# Enantioselective Michael addition to vinyl phosphonates *via* hydrogen bond-enhanced halogen bond catalysis†

**DOI:** 10.1039/d1sc01029h

**Published:** 2021-04-26

**Authors:** Mikk Kaasik, Jevgenija Martõnova, Kristin Erkman, Andrus Metsala, Ivar Järving, Tõnis Kanger

**Affiliations:** Department of Chemistry and Biotechnology, Tallinn University of Technology Akadeemia tee 15 12618 Tallinn Estonia tonis.kanger@taltech.ee

## Abstract

An asymmetric Michael addition of malononitrile to vinyl phosphonates was accomplished by hydrogen bond-enhanced bifunctional halogen bond (XB) catalysis. NMR titration experiments were used to demonstrate that halogen bonding, with the support of hydrogen-bonding, played a key role in the activation of the Michael acceptors through the phosphonate group. This is the first example of the use of XBs for the activation of organophosphorus compounds in synthesis. In addition, the iodo-perfluorophenyl group proved to be a better directing unit than different iodo- and nitro-substituted phenyl groups. The developed approach afforded products with up to excellent yields and diastereoselectivities and up to good enantioselectivities.

## Abstract 2

An asymmetric Michael addition of malononitrile to vinyl phosphonates was accomplished by hydrogen bond-enhanced bifunctional halogen bond (XB) catalysis.

## Graphical abstract

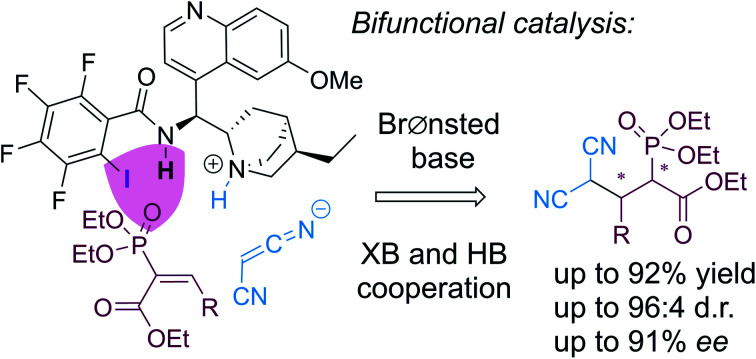

## Introduction

Halogen bonds (XBs), noncovalent interactions between electrophilic halogen atoms and Lewis bases,^1^ are exploited in areas ranging from crystal engineering to catalysis.^2^ The use of XBs in catalysis was first implemented by C. Bolm in 2008 for the reduction of quinolines.^3^ Since then several studies have demonstrated the viability of XBs to achieve catalysis.^4^ Notably, XBs have been used for the activation of imines in Mannich^5^ and aza-Diels–Alder reactions,^6^ for the activation of haloalkanes in halogen abstraction reactions,^7^ and for the activation of carbonyl compounds in Diels–Alder,^8^ Michael^9^ and Nazarov reactions.^10^ In addition, XBs have been used to activate other substrate classes, such as silyl halides,^11^ thioamides^12^ and the π-system of indoles.^13^ Yet there are no examples of the activation of phosphorus compounds by XBs. This is surprising as XBs to phosphine oxides, phosphonates and phosphates have received attention in several solution studies.^14^ Thus, organophosphorus compounds should be suitable substrates for XB catalysis.

Phosphorus compounds are an important class of molecules in organic synthesis both as valuable reagents and end targets.^15^ More specifically, (chiral) organophosphonates and their phosphonic acid derivatives can be used in the much-acclaimed Horner–Wadsworth–Emmons reaction^16^ and are also valuable biomolecules.^17^ Therefore, the asymmetric synthesis of organophosphonates has received significant attention over the years.^18^ In several instances this has been successfully achieved through the use of well-developed asymmetric hydrogen-bond catalysis, which can be compared to the less explored XB catalysis. The two major advantages of XBs compared to HBs are their high directionality,^19^ due to the formation of the XB at the elongation of the covalent bond to the halogen atom, and their high degree of variability. Namely, there is a choice among XB donor atoms, which can be ranked by the increase in their donor ability in the order of Cl < Br < I, corresponding to the increase in the polarisability of the halogen atom.^20^ Although several examples can be found for the use of XBs in asymmetric synthesis, the deliberate use of XBs for catalytic purposes is still in its infancy.^4*e*^ Notably, in 2018 the Arai group used a bifunctional alkaloid-based XB catalyst to carry out an asymmetric Mannich reaction,^5*a*^ in 2020 the Huber group carried out the first solely XB-catalysed asymmetric reaction using the Mukaiyama-aldol reaction as a model^21^ and in 2021 the Mancheño group demonstrated that a neutral tetrakis-iodo-triazole can be used as an asymmetric XB catalyst in the Reissert-type dearomatization of quinolone.^22^

We envisioned that the bifunctional alkaloid-based XB catalyst could be used to activate vinyl phosphonates in a Michael reaction with a nucleophile. Achieving this would both expand the chemical space of asymmetric XB catalysis and that of optically active phosphorus chemistry. We were also curious to see what influence the modification of the electronic properties and substitution pattern of the XB donor motif of the catalyst would have on the outcome of the reaction. So far, perfluorinated^3,5,7*b*^ and azolium-type^6*a*,7*d*,8*a*,13,21^ XB donor fragments have commonly been used in XB catalysts.

## Results and discussion

**Scheme 1:**
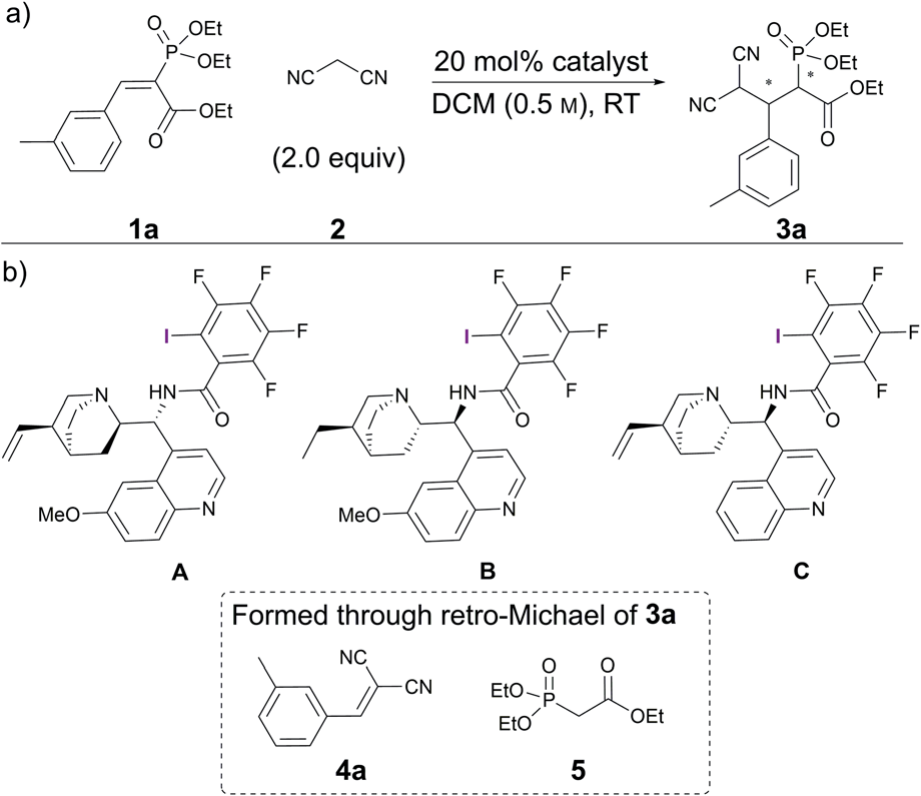
(a) Catalytic Michael reaction under study; (b) screened catalysts and decomposition products of **3a**.

Initial experiments involving vinyl phosphonate **1a** and malononitrile **2** (Scheme 1) revealed that no reaction occurred in two days at RT in DCM (Table 1, entry 1). Gratifyingly, alkaloid-based XB catalysts facilitated the reaction, resulting in the formation of enantioenriched product **3a**. Quinidine- and dihydroquinine-derived catalysts **A** and **B** provided product **3a** in similar quantities and enantioenrichment levels (Table 1, entries 2 and 3). As expected, the enantioselectivity was opposite, because the catalysts were derived from pseudo-enantiomeric alkaloids (although in addition to this difference, the vinyl group connected to the quinuclidine fragment had been reduced in catalyst **B**). It was also observed that a side reaction resulting in compounds **4a** and **5** took place that reduced the yield of the product. To hinder this, catalyst loading was dropped to 10 mol% for the following experiments. In addition, 1,3,5-trimethoxybenzene ((MeO)_3_C_6_H_3_) was used as a quantitative internal standard to determine the distribution of starting materials and products in the reaction mixture by ^1^H NMR spectroscopy. Cinchonidine-derived catalyst **C** performed much more sluggishly compared to catalysts **B**, providing product **3a** in lower conversion and enantioselectivity (Table 1, compare entries 4 and 5). In all cases, similar levels of moderate diastereoselectivity were observed. It was decided to continue with dihydroquinine-derived catalyst **B** due to its superiority in enhancing the rate of the reaction compared to **A**.

**Table 1:** Initial results of the Michael reaction^a^

Entry	Catalyst	Time (h)	NMR yield of **3a**^b^ (%)	d.r.^c^	ee^d^ (%)
1	—	48	NR	ND	ND
2	**A**	48	89	85 : 15	−39/−40
3	**B**	22	84	87 : 13	40/43
4	**C** e	24	48	77 : 23	26/28
5	**B** e	24	86	85 : 15	45/46

a Unless otherwise noted, all reactions were carried out with 0.1 mmol of **1a**, 0.2 mmol of **2**, and 0.02 mmol of catalyst in 0.2 mL of DCM at RT; NR – no reaction, ND – not determined.

b Relative amount of **3a** compared to the total amount of **1a**, **3a** and **5**; determined from the reaction mixture by ^1^H NMR spectroscopy.

c Determined from the reaction mixture by ^1^H NMR spectroscopy.

d Determined by HPLC analysis on a chiral stationary phase.

e 10 mol% of catalyst instead of 20 mol% and (MeO)_3_C_6_H_3_ was used as an internal standard.

Next, an extensive solvent screening was conducted (for full details, see the ESI†), which revealed that the reaction performed well in apolar aprotic solvents, as would be expected of noncovalent catalysis. While the solvent influenced the reactivity and enantioselectivity significantly, the diastereoselectivity was not greatly affected. For further optimisation experiments, toluene was chosen as the solvent (Table 2, entry 1). During the last stage of optimisation, other reaction conditions were varied. The reaction slowed down when run under more diluted conditions (Table 2, entry 2). Gratifyingly this also resulted in an increase in enantioselectivity. The reduction of catalyst loading under the diluted conditions had little effect on the enantioselectivity, yet resulted in a slower reaction (Table 2, entries 3 and 4). An increase of the temperature to 40 °C with a catalyst loading of 5 mol% resulted in full conversion within 2 days; however it also resulted in the increase of the retro-Michael process and did not have a positive influence on enantioselectivity (Table 2, entry 5). A decrease in temperature helped to somewhat improve the enantioselectivity at the expense of conversion (Table 2, entries 6 and 8). However, the retro-Michael process was significantly hindered at lower temperatures. As a compromise, the reaction was run at 0 °C, with 10 mol% catalyst loading and at a concentration of 0.2 M to hinder product decomposition (Table 2, entry 7). The product was formed in excellent NMR yield, with moderate diastereoselectivity and acceptable enantioselectivities for both enantiomers. These conditions were then designated as optimal.

**Table 2:** Optimisation of the reaction conditions^a^

Entry	*T* (°C)	*C* (M)	**B** (mol%)	Time (h)	Conv.^b^ (%)	**5** c (%)	ee^d^ (%)
1	RT	0.5	10	16	100	20	64/66
2	RT	0.1	10	39	87	7	76/77
3	RT	0.1	7.5	48	94	10	76/76
4	RT	0.1	5	48	76	5	79/76
5	40	0.1	5	48	100	25	76/73
6	0	0.1	10	48	81	0	79/81
7	0	0.2	10	48	97	2	76/76
8	−20	0.2	10	72	86	0	79/78

a Unless otherwise noted, all reactions were carried out with 0.1 mmol of **1a**, 0.2 mmol of **2**, and 0.05 mmol of (MeO)_3_C_6_H_3_ in toluene; d.r. was determined from the reaction mixture by ^1^H NMR spectroscopy and varied between 81 : 19 to 87 : 13; for full details see the ESI.

b Conversion depicts the amount of reacted **1a** based on ^1^H qNMR measurements using (MeO)_3_C_6_H_3_ as an internal standard. Generally, the total amount of the formed **3a** and retro-Michael product **5** corresponded to the amount of **1a** depleted.

c Describes the extent of product decomposition through the retro-Michael reaction determined by ^1^H qNMR, using the methylene proton signal of triethyl phosphonoacetate **5**.

d Determined by HPLC analysis on a chiral stationary phase.

**Fig. 1:**
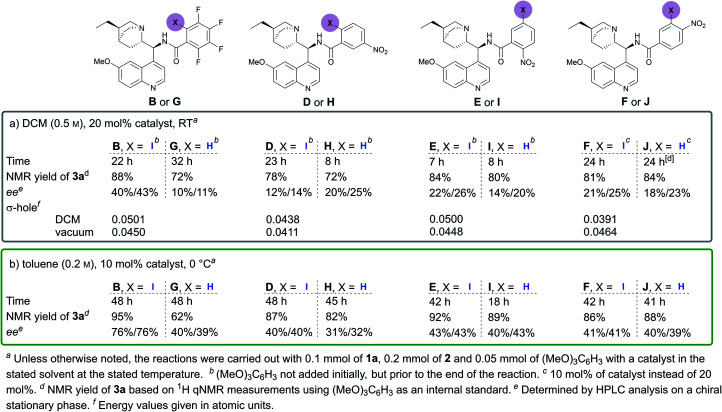
Comparison of different XB/HB catalysts in (a) DCM and (b) toluene.

Throughout the studies, we were interested in changing the XB donor fragment of the catalyst. Usually, perfluorinated haloalkyl or -aryl groups are used for this purpose in neutral XB donors, which somewhat limits catalyst design. Yet, there are several other electron-withdrawing groups that can significantly increase the magnitude of the σ-hole on the halogen atom. To explore this, we synthesised catalysts **D–F** using benzoic acids with different iodo- and nitro-substitution patterns (Fig. 1). This would have also permitted us to explore the importance of the position of the iodine atom relative to the linker substituent. As references, the non-halogenated analogues **G–J** were also synthesised (Fig. 1). During the initial studies in DCM and under the optimised conditions in toluene, catalyst **B** always performed significantly better than its non-halogenated analogue **G**, in terms of both reactivity and selectivity (Fig. 1). These results demonstrate the positive effect of the iodine atom on catalyst activity and support XB involvement in the catalytic process. In contrast, catalysts **D–F**, containing nitro-substituted XB donor motifs, did not altogether demonstrate better performance compared to their non-halogenated analogues (Fig. 1, catalysts **H**, **I** and **J**). Moreover, all of the nitro-substituted catalysts were inferior to catalyst **B** in terms of enantioinduction, although some demonstrated better performance in terms of catalytic activity. Generally, the iodinated and non-halogenated catalysts resulted in products with similar diastereoselectivities, which overall varied between 79 : 21 and 89 : 11. Unfortunately, it was also not apparent whether the 1,2- or the 1,3- substitution pattern between the iodine atom and the linker site was more beneficial for enantioinduction and catalytic activity. It can be reasoned that the steric influence of iodine in catalyst **B** results in higher levels of enantioselectivity. Catalysts **D**, **E** and **I** also contain a bulky substituent in the same position, yet these catalysts led to significantly lower levels of product enantioselectivity. Therefore, the steric influence of the large iodine atom alone cannot be used to explain the levels of enantioselectivity obtained with **B**. On the other hand, if as initially proposed the iodine acted as a directing group, then the XB donor ability of the iodine atom could provide an answer. Therefore, molecular electrostatic potentials were calculated (CAM-B3LYP functional,^23^ DEF2TZVP basis set,^24^ with the Gaussian 09 program,^25^ solvent effects with PCM^26^) for the catalysts to determine the magnitude of the σ-hole on the iodine atom (Fig. 1a). The substitution pattern influenced the magnitude of the σ-hole considerably. In addition, the XB donor ability (represented by the σ-hole value) of these compounds seems to be dependent on the environment as catalyst **B** had the largest σ-hole in DCM, while catalyst **F** had the largest σ-hole in a vacuum. Unfortunately, no clear correlation between product selectivity and the XB donor ability of the catalyst can be drawn, although the calculations support the fact that catalyst **B** was the best XB donor in DCM.

**Scheme 2:**
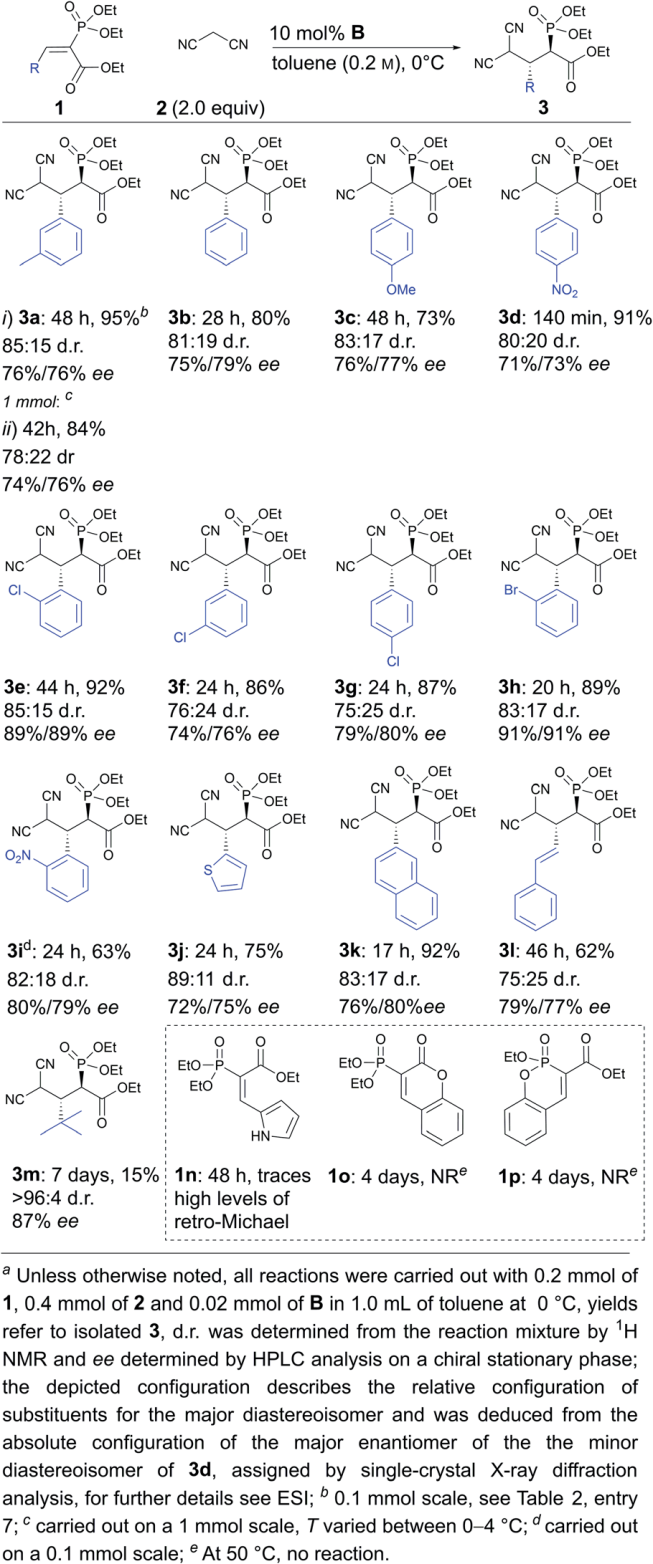
Scope of the enantioselective Michael reaction.

Next, the scope and limitations of the method were explored under the optimised conditions. Aromatic substituents containing electron-withdrawing and electron-donating substituents were tolerated (Scheme 2, compare **3c** and **3d**), as well as variations in the position of different substituents either in the *ortho*, *meta* or *para* position (Scheme 2, compare **3e**, **3f** and **3g** to **3b**). The reaction could be carried out with halogenated (**3e–h**), sterically demanding naphthyl (**3k**), heteroaromatic (**3j**) and polyconjugated substrates (**3l**). It should be noted that the substitution pattern greatly affected the speed of the reaction. For example, the reaction was complete within hours in the case of compound **3d**, while two days were needed to obtain products **3a**, **3c** and **3e**, with sterically more demanding or electron-donating substituents. In addition, *tert*-butyl-substituted substrate **1m** reacted very sluggishly, although the product was obtained in excellent diastereoselectivity and good enantioselectivity. The method was also successfully used on a 1 mmol scale (**3a**). Unfortunately, in all cases product decomposition through a retro-Michael reaction was observed despite the lower temperature. This resulted in somewhat lower yields for the products. All in all, the products were obtained in low to excellent yields (15% to 92%) and diastereoselectivities (75 : 25 to >96 : 4 d.r.) and moderate to good enantioselectivities (71% to 91% ee). It seems that *ortho*-substituents in the aromatic ring of **1** have a beneficial effect on enantioselectivity (products **3e**, **3h** and **3i**). We also observed that substrates with a *Z*-configuration of the double bond were not compatible with this catalytic system. These substrates reacted very sluggishly, leading to racemic or near racemic products, although the diastereomeric preference (with similar d.r. levels) was the same as for the corresponding *E*-isomers (for details, see the ESI†).‡‡However, it should be noted that the *Z*-isomers obtained were not as pure as the *E*-isomers. In the case of the pyrrole-containing compound **1n**, trace amounts of product were observed, along with significant levels of the retro-Michael product. This indicates that compound **1n** was less reactive than the other substrates and, at the same time, the corresponding Michael product **3n** was even more labile than the other products. Unfortunately, cyclic phosphonates **1o** and **1p** did not react even under elevated temperatures. The increased stability of **1o** and **1p** might have been caused by the existence of aromatic resonance structures.

**Scheme 3:**
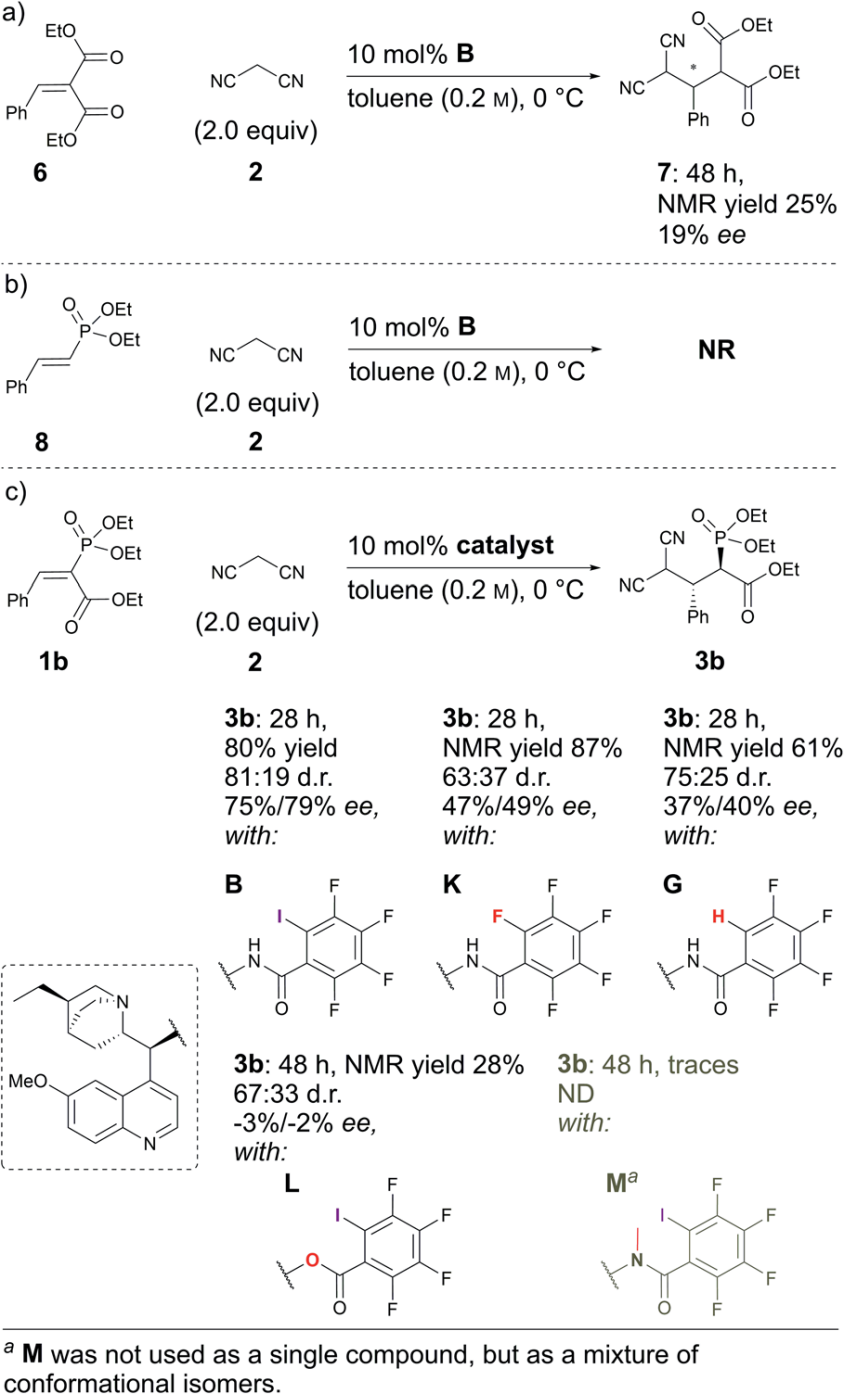
Comparative experiments using (a) **6**, (b) **8** and (c) **1b** as Michael acceptors with different catalysts.

Next, some control experiments were conducted. To ascertain the possible cause of product **3** decomposition, product **3a** and catalyst **B** were stirred at RT in toluene for seven days. It was observed that compound **3a** decomposed, resulting in the formation of retro-Michael products **4a** and **5**. Interestingly, at first the minor diastereoisomer was consumed to a greater extent than the major diastereoisomer, leading to an increase in the diastereomeric ratio. Fortunately, no racemisation took place; in contrast, a slight increase in the enantioenrichment of both enantiomers (see Fig. S3 in the ESI† for details) was observed. This confirmed that the catalyst brought about the decomposition of **3a**, which is otherwise relatively stable.§§The products were stable throughout purification by column chromatography on silica gel and subsequent solvent removal under reduced pressure. However, product decomposition was observed if left at temperatures above 50 °C for prolonged periods. Then experiments were conducted to explore the influence of the carbonyl and phosphonate groups on the reaction. A sluggish reaction took place with substrate **6**, in which the phosphonate group was exchanged for an ethoxycarbonyl group (Scheme 3a). In two days, 50% conversion was obtained, although by NMR only 25% of product **7** had formed, along with 25% of the corresponding retro-Michael products. The stereoselectivity of the reaction was also poor: only 19% ee. This example highlights the importance of the phosphonate group both for reactivity and selectivity (Scheme 3, compare (a) and (c)). The result was also expected, as an ethoxycarbonyl group should be a weaker XB acceptor than a phosphonate group. Unfortunately, compound **8** in which the ethoxycarbonyl group had been omitted did not react with malononitrile under the optimal conditions (Scheme 3b). The presence of the electron-withdrawing ethoxycarbonyl group must therefore have been critical for reactivity.^27^

In addition, control experiments were conducted with catalyst analogues (**G**, **K**, **L** and **M**, Scheme 3c). As with substrate **1a**, the hydrogen analogue of catalyst **B** (catalyst **G**) also performed sluggishly with substrate **1b**. Pentafluorophenyl substituted catalyst **K** was as active as catalyst **B**, but caused the formation of the product with diminished diastereoselectivity and enantioselectivity. These results again demonstrate the importance of the iodine atom. Next, catalyst analogues were used in which the amidic hydrogen atom was omitted. Unfortunately, the *N*-methylated analogue **M** could not be isolated as a single compound and as a result the corresponding ester **L** was also synthesised. In both instances, the catalysts demonstrated poor catalytic activity and no enantioinduction. Therefore, the amidic hydrogen atom was also a critical design element of the catalyst.

To probe the participation of XBs in the reaction, ^1^H NMR titration experiments in toluene-d8 were carried out between the substrates of the reaction and catalyst analogues **9** to **13** of the XB donor motif (Table 3). In these achiral analogues the alkaloid moiety was omitted, although a possible hydrogen bond donor site in amides **9**, **10** and **13** was still present. Compounds **1b** and **9** formed complex [**1b–9**], with an association constant value of *K*_obs_ = 9.4 M^−1^ (Table 3, entry 1), which was in the expected range based on the literature.^14*a*^ Surprisingly, the non-halogenated analogue **10** also formed a relatively strong complex with compound **1b** (Table 3, entry 2), yet the corresponding association constant of *K*_obs_ = 2.5 M^−1^ is about four times smaller. When the amidic hydrogen atom was omitted from the analogue, the interaction was more than an order of magnitude weaker for analogues **11** and **12** (Table 3, compare entries 1, 3 and 4). Based on the control experiments, association constant values of *K*_obs_ = 0.5 M^−1^ and *K*_obs_ = 0.4 M^−1^ do not correspond to a sufficiently strong XB with the substrate to achieve a significant interaction with the substrate. Analogue **13** interacted strongly with substrate **1b**, which most likely was due to hydrogen-bonding between the two and reflects the increase of acidity of the amidic hydrogen atom in **13** by the perfluorophenyl group (Table 3, entry 5). Titration experiments were also carried out between catalyst **B** and compound **1b**. The association constant values of *K*_obs_ = 3.4 M^−1^ signifies a favourable interaction between the two (Table 3, entry 6). In this instance, the change in the chemical shift of the fluorine atom *ortho* to the iodine atom was used as input for the calculation of the association constant value. The observed upfield shift upon the formation of complex [**1b–B**] is indicative of halogen bonding.^29^

**Table 3:** Association constants for XB donor and Michael reaction substrate pairs

Entry	XB acceptor	XB donor	*K*_obs_ a (M^−1^)
1	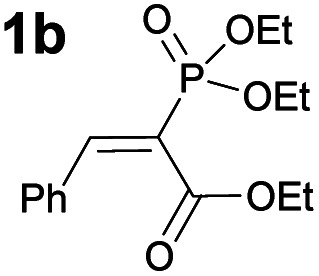	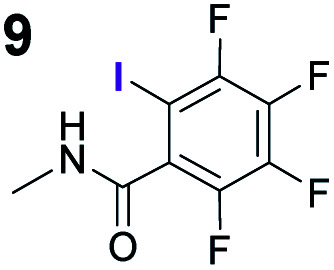	9.4
2	**1b**	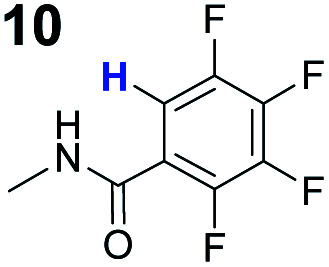	2.5
3	**1b**	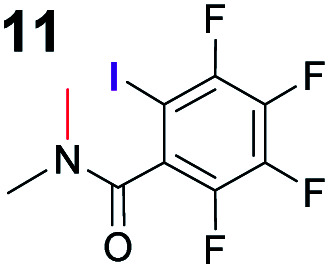	0.5
4	**1b**	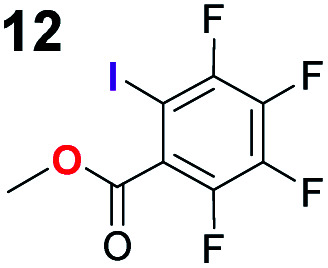	0.4
5	**1b**	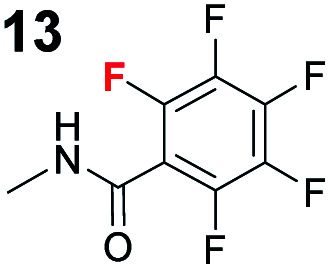	12.6
6^b^	**1b**	**B**	3.4
7	**6**	**9**	1.3
8	**8**	**9**	14.0

a The *K*_obs_ was measured in toluene-d8 and determined by fitting the ^1^H NMR titration data of the *N*- or *O*-methyl protons to the 1 : 1 binding isotherm of BindFit.^28^ The given *K*_obs_ is the calculated approximate mean value of two parallel experiments; in all cases the estimated standard errors fall below 10%. Full details are given in the ESI.

b Determined by fitting the ^19^F NMR titration data of the fluorine atom *ortho* to the iodine atom to the 1 : 1 binding isotherm of BindFit.

Malonic ester-derived compound **6** and phosphonate **8** also formed complexes with XB donor **9**. The association constant corresponding to complex [**8–9**] (Table 3, entry 8) was larger than that for the [**1b–9**] complex. On the other hand, the association constant corresponding to complex [**6–9**] (Table 3, entry 7) was almost an order of magnitude smaller than that for the [**1b–9**] complex. Thus, the phosphonate group should be the primary interaction site in the substrates and not the carbonyl group. This is also supported by the fact that when ethyl acetate was used as a solvent the reaction was not significantly hindered, as 87% conversion was reached in 24 hours and the ee of the product was 55% (this should be compared to entry 1 in Table 2; for further details, see the ESI†). The increase in the strength of the observable association constant in the series [**6–9**] < [**1b–10**] < [**1b–9**] < [**1b–13**] also aligns with the increase in reactivity in the corresponding catalytic reactions (comparison of results with catalysts **B**, **G** and **K** and reactions with substrates **1b** and **6** in Scheme 3). The higher binding potential of the amide fragment also seems to correspond to increased levels of product enantioselectivity, although an iodine atom needs to be present for the best results. Therefore, the amide fragment acted as both a coordinating and an activating unit.

**Fig. 2:**
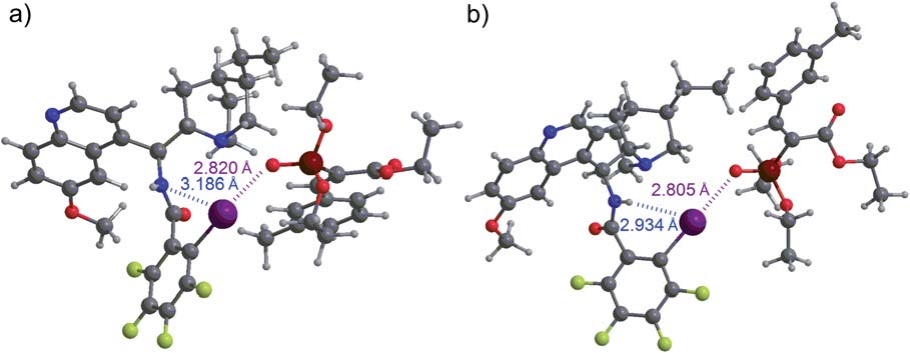
XB formation from **B** to the oxygen atom of the phosphonate group of **1a**: (a) without HB enhancement and (b) with HB enhancement.

Based on these observations, we supposed that an XB is formed to substrate **1b** and the interaction is strengthened by the participation of a hydrogen bond, either directly with the substrate or through hydrogen-bond-assisted halogen bonding.^14*b*,30^ In the latter case, the 1,3-substitution pattern between the iodine atom and the linker substituent would not be favourable for catalytic purposes, which could be one reason for the poor performance of catalysts **E** and **F** (Fig. 1). To evaluate the interaction between catalyst **B** and substrate **1a**, computations were performed (see the ESI† for details). These revealed that while an XB is formed to the oxygen atom of the phosphonate group, hydrogen-bonding to other acceptor sites in the substrate is not preferred. The calculated lowest energy conformers had an energy difference of 1.2 kcal mol^−1^ (Fig. 2). It was observed that a stronger HB from the amide group to the iodine atom also resulted in the shortening of the XB to the oxygen atom of the phosphonate group. NCI analysis also confirmed the presence of hydrogen-bonding to the iodine atom (see Fig. S2 in the ESI† for details).

**Fig. 3:**
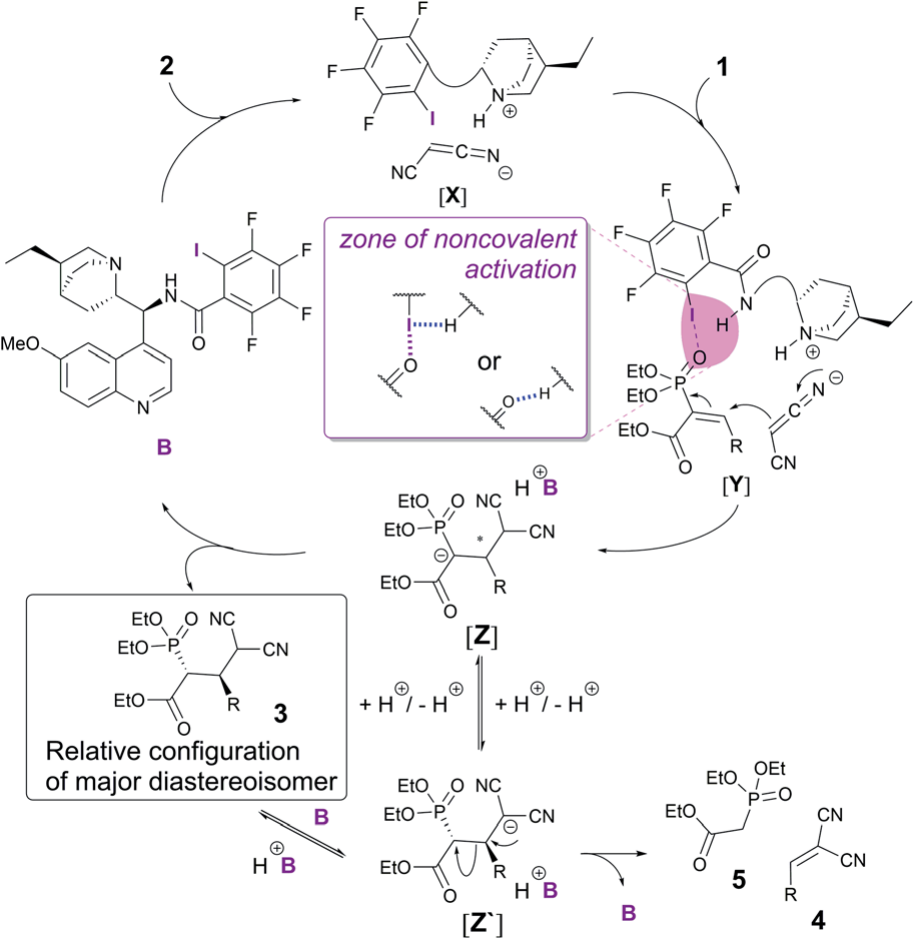
Possible catalytic cycle for the enantioselective Michael reaction.

With the obtained knowledge, we now propose a catalytic cycle similar to the one proposed by the Arai group to describe the XB catalytic Mannich reaction.^5^ First, malononitrile **2** is deprotonated and activated by the base in catalyst **B** (Fig. 3). Electrophile **1** is activated through an XB formed between the iodine atom in catalyst **B** and the oxygen atom of the phosphonate group in **1** (Fig. 3, [**Y**]). A hydrogen bond from the amide group of the catalyst to the iodine atom would enhance the XB to the phosphonate group (Fig. 3, middle). Direct activation of the phosphonate by hydrogen-bonding is also plausible and cannot be ruled out with the current experimental data. Next, a Michael reaction between **1** and **2** would result in the formation of intermediate [**Z**]. Protonation of this species leads to product **3**. This in turn could be deprotonated by the catalyst, which could lead to a retro-Michael reaction through [**Z′**]. Alternatively, it is possible that proton transfer in intermediate [**Z**] could also lead to intermediate [**Z′**], which in turn could be protonated to give product **3** or lead again to a retro-Michael reaction giving **4** and **5**.

## Conclusions

A new asymmetric organocatalytic methodology was developed that utilises a hydrogen bond-enhanced bifunctional XB catalyst for the activation of vinyl phosphonates in a Michael reaction. This approach describes the first use of organophosphorus compounds as substrates in XB catalysis and furthermore expands the field of asymmetric XB catalysis. Comparative experiments with catalysts containing different XB donor units and their hydrogen-analogues confirmed the superiority of the iodoperfluoro group as an activating and enantioinducing unit. Furthermore, NMR titration experiments and computations demonstrated that this was primarily the result of halogen bonding, supported by hydrogen-bonding, with the phosphonate group.

## Author contributions

MK conceived the project. MK, KE conducted the synthesis and analysis. JM performed crystal analysis, IJ massspectrometric analysis, AM theoretical calculations. All authors contributed to the discussion. MK and TK wrote the manuscript with contributions from all authors. TK supervised the project.

## Conflicts of interest

There are no conflicts to declare.

## Supplementary Material

SC-012-D1SC01029H-s001

SC-012-D1SC01029H-s002
