# Supplementary material for: Enantioselective Michael addition to vinyl phosphonates via hydrogen bond-enhanced halogen bond catalysis
Source: Chem Sci. 2021 Apr 26;12(21):7561–8. doi: 10.1039/d1sc01029h (PMC8171314; doi:10.1039/d1sc01029h)
Supplement: SC-012-D1SC01029H-s001 [file SC-012-D1SC01029H-s001.pdf]

Electronic Supplementary Material (ESI) for Chemical Science.
This journal is © The Royal Society of Chemistry 2021

Supporting Information

Enantioselective Michael Addition to Vinyl Phosphonates via Hydrogen Bond-Enhanced Halogen Bond Catalysis

Mikk Kaasik, Jevgenija Martõnova, Kristin Erkman, Andrus Metsala, Ivar Järving and Tõnis Kanger*

Department of Chemistry and Biotechnology, Tallinn University of Technology, Akadeemia tee 15, 12618 Tallinn, Estonia.
E-mail: tonis.kanger@taltech.ee

Table of Contents

Table of Contents S2
1. General Information S3
2. Solvent screening S3
3. Optimisation of the reaction conditions S4
4. Experiments with *Z*-isomers S4
5. Computational information S5
6. Determination of product **3a** stability in the presence of catalyst **B** S12
7. Single Crystal X-ray diffraction data S12
8. ^{1}H NMR titration experiments S14
   8.1. ^{1}H NMR titration data of **[1b-9]** complex S15
   8.2. ^{1}H NMR titration data of **[1b-10]** complex S17
   8.3 ^{1}H NMR titration data of **[1b-11]** complex S19
   8.4 ^{1}H NMR titration data of **[1b-12]** complex S21
   8.5 ^{1}H NMR titration data of **[1b-13]** complex S23
   8.6 ^{19}F NMR titration data of **[1b-B]** complex S25
   8.7 ^{1}H NMR titration data of **[6-9]** complex S27
   8.8 ^{1}H NMR titration data of **[8-9]** complex S29
9 Synthetic procedures for catalysts and model compounds S31
   9.1 Genera procedure A S33
10 Synthetic procedures for Michael reaction substrates S39
   10.1 Genera procedure B S39
   10.2 Genera procedure C S39
11 Synthetic procedures for asymmetric XB-assisted Michael reactions S43
   11.1 General procedure D S43
   11.2 General procedure E S43
12 NMR spectra S48
13 HPLC chromatograms of Michael reaction products S184
14 References S208

1. General Information

NMR spectra were measured on a Bruker Avance III 400 MHz instrument. The spectra are reported in parts per million (δ) referenced to the TMS signal or in some instances to the residual solvent signal [TMS δ = 0.00 ppm, [D$_6$]DMSO δ = 2.50 ppm (for ^{1}H NMR), TMS δ = 0.00 ppm, [D$_6$]DMSO δ = 39.52 ppm (for ^{13}C NMR)]). High resolution mass spectra were recorded using an Agilent Technologies 6540 UHD Accurate-Mass Q-TOF LC/MS spectrometer by using AJ-ESI ionization. Optical rotations were obtained using an Anton Paar GWB Polarimeter MCP 500. IR spectra were recorded on a Bruker Tensor 27 FT-IR spectrophotometer. IR absorption frequencies in wavenumbers are listed, with the relative strength in parentheses (w = weak, m = medium, s = strong). Precoated silica gel 60 F_{254} plates from Merck were used for TLC, whereas for column chromatography silica gel Kieselgel 40-63 µm was used. The measured melting points are uncorrected. Purchased chemicals and solvents were used as received. Acetone and ethyl acetate were distilled over phosphorous pentoxide and MeOH was dried by distillation over sodium metal. Petroleum ether (PE) has a boiling point of 40-60°C. In several instances general procedures were used, deviations from the general procedures are described with the characterisation data of the corresponding compound. The reactions were performed without additional moisture elimination unless stated otherwise.

Commercial malononitrile **1** and diethyl 2-benzylidenemalonate **6** were used in the catalytic and titration experiments, hydrochloric salts of the amino-alkaloids were at hand and previously prepared according to known procedures.[1]

2. Solvent screening

Table S1. Solvent screening.[a]

Entry	Solvent	Time	Conversion (%)[b]	**5** (%)[c]	d.r.[d]	*ee* (%)[e]
1	DCM[f]	24h	95	6	85:15	44/47
2	DCM	24h	92	6	85:15	45/46
3	$CHCl_3$	24h	96	6	84:16	51/53
4	1,2-dichloroethane	24h	97	9	85:15	42/44
5	*o*-dichlorobenzene	19h	100	18	84/16	44/46
6	toluene	16h	100	20	84:16	64/66
7	*p*-xylene	16h	100	19	83:17	64/65
8	Et_2O	24h	100	21	84:16	60/61
9	MTBE	16h	100	16	83:17	58/59
10	CPME	21h	100	18	84:16	58/61
11	THF	24h	83	7	83:17	51/53
12	hexane[g]	150 min	99	5	78:22	32/34
13	neat	35 min	86	1	71:29	27/28
14	Et_2CO_3	24h	91	9	82:18	60/63
15	EtOAc	24h	87	9	83:17	55/56
16	MeCN	24h	74	7	80:20	20/21
17	acetone	24h	7	-	-	-
18	Et_2CO_3/hexane (2/3)	16h	100	16	82:18	62/63

[a] Unless otherwise noted, all reactions were carried out with 0.1 mmol of **1a**, 0.2 mmol of **2**, 0.01 mmol of catalyst **B** and 0.05 mmol of $(MeO)_3C_6H_3$ in the stated solvent (0.2 mL) at RT. [b] Conversion depicts the amount of reacted **1a** based on ^{1}H qNMR measurements using $(MeO)_3C_6H_3$ as an internal standard. Generally, the total amount of formed **3a** and retro-Michael product **5** corresponded to the amount of **1a** depleted. MTBE - methyl *tert*-butyl ether; CPME - cyclopentyl methyl ether. [c] Describes the extent of product decomposition through the retro-Michael reaction, determined by ^{1}H qNMR, using the methylene proton signal of triethyl phosphonoacetate **5**. [d] Determined from the reaction mixture by ^{1}H NMR spectroscopy. [e] Determined by HPLC analysis on a chiral stationary phase. [f] $(MeO)_3C_6H_3$ was not added at the start of the reaction, but prior to taking the NMR sample. [g] Catalyst **B** and substrate **1a** did not dissolve in hexane and a biphasic mixture formed.

3. Optimisation of the reaction conditions

Table S2. Optimisation of the reaction conditions.[a]

Entry	T (°C)	C (M)	**B** (mol%)	Time (h)	Conversion (%)[b]	**5** (%)[c]	d.r.[d]	*ee* (%)[e]
1	RT	0.5	10	16	100	20	84:16	64/66
2	RT	0.1	10	39	87	7	87:13	76/77
3	RT	0.1	7.5	48	94	10	86:14	76/76
4	RT	0.1	5	48	76	5	84:16	79/76
5	40	0.1	5	48	100	25	85:15	76/73
6	0	0.1	10	48	81	0	85:15	79/81
7	0	0.2	10	48	97	2	85:15	76/76
8	-20	0.2	10	72	86	0	81:19	79/78

[a] Unless otherwise noted, all reactions were carried out with 0.1 mmol of **1a**, 0.2 mmol of **2**, and 0.05 mmol of $(MeO)_3C_6H_3$ in toluene. [b] Conversion depicts the amount of reacted **1a** based on ^{1}H qNMR measurements using $(MeO)_3C_6H_3$ as an internal standard. Generally, the total amount of formed **3a** and retro-Michael product **5** corresponded to the amount of **1a** depleted. [c] Describes the extent of product decomposition through the retro-Michael reaction determined by ^{1}H qNMR, using the methylene proton signal of triethyl phosphonoacetate **5**. [d] Determined from the reaction mixture by ^{1}H NMR spectroscopy. [e] Determined by HPLC analysis on a chiral stationary phase.

4. Experiments with *Z*-isomers

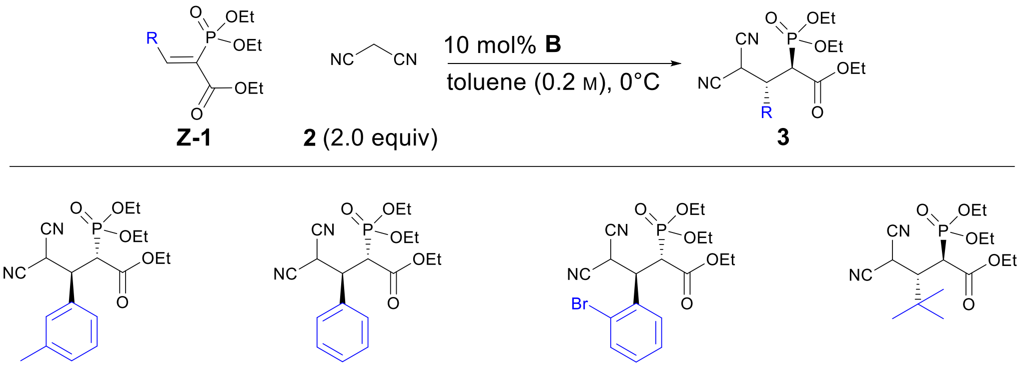

3a: 0.1 mmol
7 days,
59% NMR yield
85:15 dr
-2%/-1% *ee*

3b: 0.16 mmol
5 days,
46% NMR yield
83:17 d.r.
-4%/-3% *ee*

3h: 0.1 mmol
7 days,
22% NMR yield
81:19 d.r.
-9%/-10% *ee*

3m: 0.2 mmol
48 h,
62% yield[b]
>96:4 d.r.
3% *ee*

[a] NMR yield refers to the relative amount of **3** compared to the total amount of **1**, **3** and **5**; determined from the reaction mixture by ^{1}H NMR spectroscopy;· d.r. was determined from the reaction mixture by ^{1}H NMR and *ee* determined by HPLC analysis on a chiral stationary phase for a sample prepared by preparative TLC; the depicted configuration describes the relative configuration of substituents for the major diastereoisomer and was deduced from the absolute configuration of the major enantiomer of the the minor diastereoisomer of **3d**, assigned by single-crystal X-ray diffraction analysis; [b] Isolated yield. [c] The purity was estimated to be 79% for ***Z*-1a**, 82% for ***Z*-1b** and 86% ***Z*-1a.**

Figure S1. Experiments with *Z*-isomers.

5. Computational information

In order to find ESP values, an extensive calculation procedure was performed. Initially, conformational analysis was performed with the program Vega.[2,3] All the torsional angles were scanned. About 70 generated lowest energy conformers were used as input for quantum chemical calculations. The quantum chemistry calculation was performed with the CAM-B3LYP functional[4] with the DEF2TZVP basis set[5] on the program Gaussian09[6]. In order to model the solvent (DCM), the Polarizable Continuum Model (PCM)[7] was used. All the generated structures were verified with frequency calculations in order to be sure that imaginary frequencies were not present. The latter step also gave us Gibbs free energy values; this was helpful in order to obtain a minimum energy structure (most stable structure). The energy window about 2 kcal/mol was used for further analysis of ESP values. These values were calculated with the help of the Multiwfn program;[8] visualisation was made with the MOLEKEL[9] program. Lowest energy (<0.5) conformers were selected as a most probable source of significant ESP influence. For example, in Table S2 the second lowest conformer (0.299 kcal/mol) in contrast to the lowest energy conformer (0.0 kcal/mol) gave the most positive σ-hole value for iodine (Table S3; comparing MK831_tzvp_s_7.fchk to MK831_tzvp_s_2.fchk). This is because: a) in the experimental situation any stronger ESP value is dominating; b) another thing is a methodological, (computational) error which is assumed to be about 0.5 kcal/mol.

Table S3. Calculated lowest energy conformers in DCM.

File	E kcal/mol	E AU
MK831_tzvp_s_2.out	0	-2056.177552
MK831_tzvp_s_7.out	0.299	-2056.177075
MK831_tzvp_s_16.out	0.513	-2056.176735
MK831_tzvp_s_12.out	1.542	-2056.175095
MK843_tzvp_s_2.out	0	-1863.678914
MK843_tzvp_s_1.out	1.682	-1863.676234
MK843_tzvp_s_28.out	2.142	-1863.6755
MK843_tzvp_s_20.out	2.142	-1863.6755
MK852_tzvp_s_15.out	0	-1863.676073
MK852_tzvp_s_3.out	0.774	-1863.674839
MK852_tzvp_s_1.out	1.061	-1863.674382
MK852_tzvp_s_12.out	2.029	-1863.672839
MK863_tzvp_s_1.out	0	-1863.674645
MK863_tzvp_s_4.out	0.708	-1863.673517
MK863_tzvp_s_76.out	0.751	-1863.673448
MK863_tzvp_s_13.out	1.399	-1863.672416

Table S4. Most positive ESP values for the calculated conformers in DCM.

File	ESP AU
MK831_tzvp_s_2.fchk	0.04156251
MK831_tzvp_s_16.fchk	0.03784048
MK831_tzvp_s_7.fchk	0.05010309
MK843_tzvp_s_2.fchk	0.04383222
MK852_tzvp_s_1.fchk	0.0481847
MK852_tzvp_s_3.fchk	0.04758427
MK852_tzvp_s_15.fchk	0.04997786
MK863_tzvp_s_4.fchk	0.03888314
MK863_tzvp_s_1.fchk	0.03905414

Table S5. Calculated lowest energy conformers in vacuum.

File	E kcal/mol	E AU
MK831_tzvp_2.out	0	-2056.16248
MK831_tzvp_16.out	0.302	-2056.161999
MK831_tzvp_7.out	0.434	-2056.161788
MK831_tzvp_12.out	1.016	-2056.160861
MK831_tzvp_36.out	1.753	-2056.159686
MK831_tzvp_33.out	1.810	-2056.159595
MK831_tzvp_14.out	1.903	-2056.159447
MK831_tzvp_6.out	1.912	-2056.159433
MK843_tzvp_2.out	0	-1863.660766
MK843_tzvp_1.out	1.411	-1863.658518
MK852_tzvp_15.out	0	-1863.658778
MK852_tzvp_1.out	1.074	-1863.657067
MK852_tzvp_22.out	1.166	-1863.65692
MK852_tzvp_3.out	1.166	-1863.65692
MK863_tzvp_1.out	0.000	-1863.656522
MK863_tzvp_76.out	0.427	-1863.655841
MK863_tzvp_4.out	0.748	-1863.655329
MK863_tzvp_13.out	1.239	-1863.654547
MK863_tzvp_3.out	1.405	-1863.654282
MK863_tzvp_70.out	1.445	-1863.654218
MK863_tzvp_12.out	1.505	-1863.654123

Table S6. Most positive ESP values for the calculated conformers in vacuum.

File	ESP AU
MK831_tzvp_12.fchk	0.03989496
MK831_tzvp_16.fchk	0.03794391
MK831_tzvp_2.fchk	0.03951913
MK831_tzvp_7.fchk	0.04499813
MK843_tzvp_2.fchk	0.04106143
MK852_tzvp_15.fchk	0.04483053
MK852_tzvp_1.fchk	0.04272381
MK852_tzvp_3.fchk	0.04261754
MK852_tzvp_22.fchk	0.04275176
MK863_tzvp_1.fchk	0.03942275
MK863_tzvp_76.fchk	0.04641929
MK863_tzvp_4.fchk	0.03956242

Table S7. The Cartesian coordinates for the two lowest energy complexes in DCM

MK831_tzvp_s_2				MK831_tzvp_s_7				MK843_tzvp_s_2				MK843_tzvp_s_1			
C	-2.16783	-3.05380	2.26714	C	2.86319	-0.43514	-2.16827	C	-3.63624	2.41507	1.92682	C	2.54375	-1.06177	2.15799
C	-3.12763	-2.69976	3.23845	C	2.96651	-0.82948	-3.51794	C	-3.95899	3.70547	1.44459	C	2.69639	-2.07139	3.13039
C	-3.25226	-1.39608	3.61563	C	1.84536	-0.86998	-4.29235	C	-3.15883	4.30858	0.53275	C	1.72899	-3.02195	3.26697
C	-2.44613	-0.38505	3.05574	C	0.57935	-0.52840	-3.77773	C	-1.99510	3.66696	0.04101	C	0.57548	-3.02470	2.45786
C	-1.48705	-0.73789	2.06969	C	0.47261	-0.13322	-2.41791	C	-1.67670	2.36722	0.50857	C	0.42060	-2.01080	1.47596
C	-1.37160	-2.09260	1.70144	C	1.64504	-0.09659	-1.63858	C	-2.52661	1.76134	1.46979	C	1.43266	-1.03830	1.35525
N	-2.62648	0.88688	3.49633	N	-0.48494	-0.59842	-4.62014	N	-1.23813	4.34073	-0.85692	N	-0.33925	-4.00827	2.66492
C	-1.87229	1.81797	2.98476	C	-1.65407	-0.27787	-4.14631	C	-0.15959	3.75573	-1.30383	C	-1.40264	-4.01375	1.91418
C	-0.89918	1.57617	1.99951	C	-1.86709	0.12959	-2.81653	C	0.23929	2.47024	-0.91382	C	-1.64722	-3.06091	0.90828
C	-0.69559	0.31409	1.52487	C	-0.82871	0.20312	-1.93612	C	-0.50654	1.75646	-0.01731	C	-0.75959	-2.05320	0.67336
C	0.32559	0.08733	0.42643	C	-1.13243	0.57438	-0.49757	C	-0.07657	0.34570	0.34203	C	-1.10356	-1.02148	-0.38361
C	-0.18542	0.66674	-0.90578	C	-1.00071	-0.65021	0.43183	C	-0.31806	-0.60321	-0.84581	C	-1.48587	0.32789	0.25805
N	0.89273	0.75431	-1.89866	N	-0.98512	-0.24769	1.84401	N	0.35482	-1.89251	-0.64426	N	-1.51830	1.40131	-0.74336
C	0.43685	1.60723	-2.99911	C	-0.52504	-1.38720	2.64177	C	0.36679	-2.61118	-1.92164	C	-1.56419	2.68663	-0.04031
C	-0.85917	1.06179	-3.66037	C	-1.42262	-2.63916	2.43766	C	-1.07078	-2.85478	-2.46068	C	-2.80640	2.79171	0.88847
C	-1.17595	-0.28082	-2.98216	C	-2.59276	-2.20888	1.53799	C	-2.04507	-2.30363	-1.40870	C	-3.63179	1.51255	0.68207
C	-1.40086	-0.09420	-1.47859	C	-2.08589	-1.71816	0.17820	C	-1.81302	-0.80767	-1.17504	C	-2.81504	0.26758	1.04156
C	1.23581	-0.56880	-2.43176	C	-2.32345	0.14487	2.30053	C	-0.33987	-2.70840	0.35724	C	-2.69587	1.28595	-1.61087
C	0.03116	-1.20305	-3.16559	C	-3.31078	-1.04220	2.21892	C	-1.78680	-3.02961	-0.08783	C	-4.00537	1.41613	-0.79749
C	-1.99080	2.08651	-3.61996	C	-0.62165	-3.83406	1.92464	C	-1.29031	-2.28157	-3.85786	C	-2.44244	3.05558	2.34697
C	-3.26058	1.65234	-4.33719	C	-1.42316	-5.12073	1.79303	C	-0.50694	-3.01801	-4.93602	C	-1.86473	4.44535	2.57744
N	1.59966	0.72715	0.71419	N	-0.30139	1.63811	0.04287	N	1.33613	0.27222	0.67357	N	-0.04478	-0.77946	-1.34911
C	3.77156	0.96049	1.71834	C	0.50271	3.87753	0.37291	C	3.31626	0.61700	1.98959	C	1.40874	-1.23402	-3.20424
C	2.39598	0.33191	1.71453	C	-0.30737	2.88781	-0.43468	C	1.84866	0.86394	1.76449	C	0.37936	-1.71343	-2.21463
O	2.08183	-0.47408	2.57012	O	-0.88908	3.24454	-1.44202	O	1.20797	1.58906	2.50509	O	0.01301	-2.87541	-2.20084
C	4.72450	0.44773	0.86248	C	1.86550	3.94261	0.16928	C	4.20039	0.98137	0.98636	C	2.63591	-0.81112	-2.71895
C	6.02308	0.91816	0.85126	C	2.65503	4.86637	0.82652	C	5.55732	0.83898	1.18886	C	3.62330	-0.43431	-3.60572
C	6.37638	1.93030	1.71632	C	2.06737	5.74445	1.71066	C	6.07189	0.32910	2.36247	C	3.42719	-0.45141	-4.97099
C	5.43163	2.45700	2.57782	C	0.70299	5.68920	1.92721	C	5.19214	-0.05054	3.35677	C	2.19954	-0.85748	-5.45520
C	4.13573	1.98188	2.59080	C	-0.08461	4.76809	1.26678	C	3.82430	0.09558	3.17767	C	1.19729	-1.25098	-4.58067
I	2.77990	2.84118	3.91902	I	-2.13133	4.75140	1.66088	I	2.58048	-0.55604	4.72855	I	-0.64287	-1.80660	-5.40687
F	4.40695	-0.53724	0.01775	F	2.45812	3.10673	-0.68504	O	-4.49846	1.92342	2.84481	O	3.45492	-0.08006	1.95705
F	6.92185	0.40470	0.02147	F	3.96345	4.91217	0.61200	C	-4.23848	0.64691	3.39919	C	4.64959	-0.08212	2.71977
F	7.61850	2.39689	1.72003	F	2.81030	6.63809	2.35124	N	6.47716	1.24107	0.12134	N	4.91949	0.00025	-3.07736
F	5.81875	3.43758	3.39083	F	0.18021	6.55404	2.79418	O	7.66765	1.12011	0.32276	O	5.06578	0.02080	-1.87282
O	-1.97540	-4.32619	1.84375	O	3.92693	-0.36371	-1.33218	O	6.00085	1.67239	-0.90845	O	5.77889	0.31869	-3.87309
C	-2.75640	-5.36532	2.40784	C	5.21586	-0.67817	-1.83029	H	-4.84681	4.19006	1.82782	H	3.56841	-2.09931	3.76564
H	-3.75958	-3.44978	3.68886	H	3.92144	-1.09595	-3.94453	H	-3.38340	5.29767	0.15766	H	1.82512	-3.80535	4.00637
H	-3.97743	-1.10412	4.36290	H	1.90165	-1.16656	-5.33080	H	-2.28763	0.78487	1.85422	H	1.36395	-0.25446	0.61885
H	-0.64624	-2.41723	0.97129	H	1.61752	0.22132	-0.60937	H	0.44157	4.30960	-2.01732	H	-2.12461	-4.80558	2.08510
H	-2.02152	2.82906	3.34946	H	-2.49611	-0.33181	-4.82864	H	1.14735	2.05401	-1.32857	H	-2.54648	-3.14822	0.31361
H	-0.30902	2.40432	1.63151	H	-2.86874	0.39361	-2.50517	H	-0.64600	0.00164	1.20371	H	-1.97055	-1.41172	-0.92202
H	0.50369	-0.98068	0.31413	H	-2.17019	0.91606	-0.48170	H	0.17983	-0.15132	-1.70611	H	-0.68327	0.59983	0.94367
H	-0.47249	1.70022	-0.70175	H	-0.02066	-1.09070	0.24860	H	0.89904	-3.55048	-1.77033	H	-1.56136	3.47706	-0.79112
H	1.24815	1.67827	-3.72417	H	-0.51276	-1.07849	3.68748	H	0.94888	-2.02180	-2.63243	H	-0.64292	2.78648	0.53654
H	0.26805	2.61062	-2.60322	H	0.50558	-1.60756	2.35704	H	-1.24470	-3.93329	-2.52302	H	-3.42575	3.62919	0.55340
H	-0.65360	0.84829	-4.71358	H	-1.84952	-2.92655	3.40316	H	-3.07181	-2.47236	-1.73629	H	-4.53254	1.55487	1.29593
H	-2.05691	-0.73480	-3.43448	H	-3.28686	-3.03682	1.39817	H	-2.44908	-0.47453	-0.35314	H	-3.38884	-0.62275	0.77590
H	-1.50907	-1.07544	-1.01346	H	-2.92475	-1.30052	-0.38286	H	-2.09743	-0.21845	-2.04574	H	-2.62702	0.20803	2.11267
H	-2.32311	0.44892	-1.27508	H	-1.68733	-2.53569	-0.42142	H	0.24061	-3.62009	0.49701	H	-2.62314	2.06565	-2.36883
H	2.08555	-0.44310	-3.10237	H	-2.22894	0.51113	3.32260	H	-0.32630	-2.18134	1.31072	H	-2.65084	0.33312	-2.13743
H	1.57712	-1.19965	-1.61171	H	-2.66520	0.98594	1.69829	H	-1.92236	-4.10428	-0.21909	H	-4.56339	2.30356	-1.10032
H	0.24597	-1.32761	-4.22813	H	-3.64307	-1.34005	3.21473	H	-2.50615	-2.70452	0.66570	H	-4.65384	0.55488	-0.96620
H	-0.18948	-2.19326	-2.76399	H	-4.20116	-0.76661	1.65161	H	-2.35674	-2.32631	-4.09596	H	-3.33748	2.93291	2.96336
H	-2.22357	2.34155	-2.58207	H	-0.16106	-3.58790	0.96362	H	-1.01744	-1.22249	-3.87209	H	-1.72685	2.30442	2.69318
H	-1.62167	3.00994	-4.07554	H	0.20926	-4.00050	2.61624	H	-0.68553	-2.58353	-5.92007	H	-1.61518	4.59987	3.62765
H	-3.99706	2.45653	-4.34740	H	-0.77950	-5.95341	1.50747	H	0.56804	-2.97973	-4.75117	H	-0.95411	4.60534	1.99747
H	-3.05056	1.38065	-5.37388	H	-1.90336	-5.38085	2.73878	H	-0.79723	-4.06967	-4.97764	H	-2.58059	5.21695	2.28724
H	-3.72437	0.79021	-3.85682	H	-2.20466	-5.03747	1.03708	H	1.82360	-0.50429	0.24384	H	0.10619	0.20082	-1.55681
H	1.98909	1.23989	-0.06804	H	0.03006	1.45942	0.98451					H	2.82947	-0.78627	-1.65711
H	-2.42327	-6.28228	1.93064	H	5.89999	-0.53917	-0.99833					H	4.21754	-0.14913	-5.64015
H	-2.59750	-5.44223	3.48502	H	5.26894	-1.71415	-2.17030					H	2.02473	-0.86493	-6.52074
H	-3.81907	-5.21991	2.20453	H	5.50077	-0.01069	-2.64557					H	5.22731	0.76920	2.37219
												H	4.44190	0.03454	3.78504
												H	5.22289	-0.99649	2.55674

Table S8. The Cartesian coordinates for the two lowest energy complexes in DCM

MK852_tzvp_s_3				MK852_tzvp_s_15				MK863_tzvp_s_1				MK863_tzvp_s_4			
C	-3.50785	-1.58121	-2.12756	C	1.22669	-2.92498	-2.22978	C	-2.55975	-0.15783	-4.02292	C	-2.58825	-2.77055	0.32007
C	-4.67911	-0.81304	-2.29527	C	1.13113	-4.33059	-2.10707	C	-2.62420	0.94814	-4.90299	C	-3.89368	-2.96969	-0.18585
C	-4.79047	0.38926	-1.66322	C	0.35575	-4.87433	-1.13792	C	-2.03437	2.11974	-4.56431	C	-4.39737	-2.11958	-1.11284
C	-3.75900	0.89167	-0.84511	C	-0.36580	-4.05845	-0.23217	C	-1.34256	2.26364	-3.33612	C	-3.64022	-1.02053	-1.58778
C	-2.57466	0.12479	-0.68394	C	-0.27288	-2.64932	-0.34970	C	-1.26244	1.15401	-2.45766	C	-2.33406	-0.81168	-1.07845
C	-2.48621	-1.11836	-1.34029	C	0.53944	-2.10487	-1.37869	C	-1.89450	-0.05976	-2.83322	C	-1.82604	-1.72215	-0.11494
N	-3.95475	2.09348	-0.24347	N	-1.11137	-4.68089	0.71287	N	-0.79314	3.47048	-3.06251	N	-4.21820	-0.21674	-2.51295
C	-3.00267	2.55087	0.51933	C	-1.77636	-3.93434	1.55107	C	-0.16603	3.60949	-1.92557	C	-3.52560	0.79695	-2.95437
C	-1.78976	1.87502	0.73888	C	-1.75185	-2.53228	1.52146	C	-0.02374	2.57516	-0.99107	C	-2.22780	1.09339	-2.51383
C	-1.55211	0.67003	0.14546	C	-1.00641	-1.86818	0.58830	C	-0.55526	1.34008	-1.23939	C	-1.61504	0.31307	-1.57399
C	-0.21522	-0.01488	0.36108	C	-0.96902	-0.35277	0.64860	C	-0.33649	0.22744	-0.25093	C	-0.24457	0.73793	-1.08112
C	0.89444	0.72108	-0.41296	C	0.40242	0.15513	1.13843	C	1.12997	-0.24055	-0.26251	C	-0.32055	1.30006	0.35336
N	2.22688	0.28307	0.02183	N	0.55413	1.59555	0.89612	N	1.44316	-1.08786	0.89555	N	1.01950	1.42983	0.94256
C	3.21535	1.23412	-0.49175	C	1.96414	1.94722	1.08012	C	2.89996	-1.21916	0.99089	C	0.87034	1.63658	2.38550
C	3.16960	1.34283	-2.04110	C	2.47385	1.58429	2.50242	C	3.50806	-1.84832	-0.29407	C	0.00421	2.88594	2.70697
C	2.13738	0.31444	-2.53060	C	1.26313	1.05940	3.29115	C	2.33540	-2.19327	-1.22431	C	-0.30591	3.57196	1.36651
C	0.75250	0.60995	-1.94684	C	0.67120	-0.18406	2.62083	C	1.52818	-0.94083	-1.57994	C	-1.08894	2.63553	0.44120
C	2.54226	-1.05529	-0.49009	C	-0.25974	2.38215	1.83088	C	0.85011	-2.42247	0.75800	C	1.74373	2.57122	0.37030
C	2.56998	-1.06760	-2.03645	C	0.18810	2.14799	3.29235	C	1.40461	-3.15610	-0.48634	C	1.02288	3.90251	0.68414
C	2.91808	2.77713	-2.50121	C	3.67000	0.63611	2.45150	C	4.56848	-0.96672	-0.94755	C	-1.22102	2.52733	3.54530
C	2.95922	2.97225	-4.00970	C	4.27963	0.31652	3.80855	C	5.84116	-0.84902	-0.12002	C	-2.06105	3.72068	3.97605
N	0.17171	-0.02724	1.76182	N	-1.27894	0.30270	-0.61193	N	-0.62105	0.65865	1.12687	N	0.75877	-0.31175	-1.09606
C	0.11917	-0.74786	4.05581	C	-2.71173	1.16787	-2.34243	C	-2.04385	1.24935	2.99168	C	2.36101	-1.81624	-2.11329
C	-0.47805	-0.76654	2.67173	C	-2.52180	0.30790	-1.11766	C	-1.87248	0.94102	1.53067	C	1.20442	-0.86142	-2.23986
O	-1.52144	-1.35793	2.45990	O	-3.43894	-0.37721	-0.70372	O	-2.82789	0.95959	0.77087	O	0.71257	-0.62093	-3.33069
C	0.60188	-1.88173	4.69351	C	-3.49858	2.31098	-2.36398	C	-1.01035	1.71730	3.79016	C	2.72678	-2.41729	-0.91731
C	1.01327	-1.86495	6.01012	C	-3.75691	3.00174	-3.53066	C	-1.24761	2.00069	5.11882	C	3.79093	-3.29369	-0.88837
C	0.96299	-0.68177	6.72221	C	-3.20807	2.55916	-4.71844	C	-2.50523	1.78691	5.65053	C	4.50139	-3.54675	-2.04647
C	0.49338	0.46218	6.10054	C	-2.41604	1.42374	-4.71575	C	-3.55906	1.32874	4.87050	C	4.14950	-2.96838	-3.25875
C	0.07114	0.43203	4.78038	C	-2.17170	0.73002	-3.54102	C	-3.30986	1.08027	3.53175	C	3.06208	-2.11194	-3.27271
I	0.40894	2.25844	7.16310	I	-1.58141	0.73799	-6.50313	I	-5.52362	1.08775	5.54040	I	5.06960	-3.37977	-5.08927
O	-3.32212	-2.78811	-2.71409	O	2.02893	-2.49882	-3.23174	O	-3.19754	-1.26614	-4.46200	O	-2.19243	-3.68591	1.23367
C	-4.35048	-3.32359	-3.52857	C	2.20748	-1.10580	-3.40802	C	-3.19862	-2.41614	-3.63639	C	-0.89195	-3.57570	1.78016
N	0.73504	-3.14185	3.95889	N	-4.05777	2.84738	-1.12114	N	-2.65047	2.06923	7.08313	N	5.64542	-4.45533	-1.90910
O	0.90811	-3.08047	2.75948	O	-5.04106	3.55306	-1.19892	O	-3.25406	1.26887	7.76234	O	5.48753	-5.44628	-1.22732
O	0.68859	-4.17473	4.59314	O	-3.49478	2.57182	-0.08162	O	-2.12874	3.07988	7.50479	O	6.68195	-4.15210	-2.45669
H	-5.48875	-1.16945	-2.91352	H	1.68628	-4.94698	-2.80115	H	-3.15771	0.83141	-5.83661	H	-4.46510	-3.81140	0.18142
H	-5.68236	0.99083	-1.77392	H	0.26730	-5.94638	-1.02737	H	-2.07853	2.97895	-5.21942	H	-5.39128	-2.25620	-1.51650
H	-1.61685	-1.74889	-1.23425	H	0.59828	-1.03754	-1.49456	H	-1.86274	-0.90651	-2.16951	H	-0.82542	-1.59069	0.25655
H	-3.17295	3.50925	0.99911	H	-2.37365	-4.44408	2.29977	H	0.26302	4.58380	-1.71591	H	-3.99599	1.42938	-3.69997
H	-1.05133	2.32199	1.39071	H	-2.34109	-1.98189	2.24242	H	0.50542	2.76962	-0.06808	H	-1.71341	1.94628	-2.93534
H	-0.27826	-1.04593	0.01702	H	-1.72979	-0.05666	1.37450	H	-0.99329	-0.60823	-0.46666	H	0.08559	1.53497	-1.75215
H	0.82804	1.77139	-0.12149	H	1.16698	-0.32062	0.52431	H	1.74054	0.65569	-0.13496	H	-0.83179	0.55670	0.96538
H	4.19889	0.91052	-0.14968	H	2.06966	3.01496	0.88563	H	3.12315	-1.82096	1.87209	H	1.86877	1.72971	2.81383
H	3.01866	2.20698	-0.03680	H	2.54772	1.42105	0.32187	H	3.31541	-0.22499	1.16561	H	0.41712	0.73987	2.81290
H	4.14065	1.03951	-2.44386	H	2.80813	2.49963	2.99995	H	3.99252	-2.79248	-0.02744	H	0.60416	3.58700	3.29476
H	2.08798	0.31620	-3.61892	H	1.55145	0.82638	4.31559	H	2.71484	-2.66000	-2.13434	H	-0.87185	4.48786	1.53254
H	0.07350	-0.19752	-2.22480	H	-0.25246	-0.45808	3.13492	H	0.64865	-1.23844	-2.15316	H	-1.17937	3.10221	-0.54198
H	0.33057	1.52572	-2.35964	H	1.33871	-1.04106	2.70383	H	2.09838	-0.26482	-2.21567	H	-2.10388	2.47120	0.80124
H	3.50721	-1.34654	-0.07567	H	-0.16007	3.43073	1.55092	H	1.06984	-2.97472	1.67142	H	2.75287	2.56135	0.78163
H	1.81025	-1.76169	-0.10051	H	-1.30733	2.12073	1.68868	H	-0.23355	-2.32186	0.70748	H	1.84203	2.41949	-0.70412
H	3.57093	-1.29800	-2.40486	H	0.58812	3.06515	3.72771	H	1.95047	-4.05488	-0.19530	H	1.63156	4.52851	1.33861
H	1.89867	-1.83267	-2.42960	H	-0.65628	1.84017	3.91107	H	0.59321	-3.47179	-1.14428	H	0.84471	4.47071	-0.23008
H	1.95877	3.12907	-2.11083	H	3.38682	-0.29270	1.94796	H	4.81643	-1.37932	-1.92949	H	-1.84750	1.81395	3.00219
H	3.67717	3.41439	-2.03831	H	4.43348	1.09549	1.81692	H	4.16102	0.03192	-1.12906	H	-0.87248	1.99654	4.43596
H	2.85851	4.02658	-4.26949	H	5.17806	-0.29173	3.69882	H	6.57611	-0.21308	-0.61442	H	-2.86791	3.40978	4.64048
H	3.90487	2.61652	-4.42427	H	4.55937	1.23061	4.33661	H	5.64477	-0.41913	0.86376	H	-1.45358	4.45379	4.51100
H	2.15474	2.43354	-4.51140	H	3.58730	-0.23482	4.44554	H	6.29708	-1.82911	0.03339	H	-2.51588	4.22658	3.12382
H	1.14093	0.21970	1.92281	H	-0.66338	1.07986	-0.82466	H	0.07132	0.35243	1.79916	H	1.35419	-0.29103	-0.27619
H	1.38427	-2.77025	6.46612	H	-4.37317	3.88732	-3.50188	H	-0.02508	1.89057	3.38224	H	2.17994	-2.23108	-0.00460
H	1.29336	-0.66002	7.74996	H	-3.39832	3.10197	-5.63225	H	-0.46392	2.38335	5.75563	H	4.08414	-3.78408	0.02792
H	-0.31580	1.32337	4.30790	H	-1.57307	-0.16923	-3.55010	H	-4.10552	0.75294	2.87937	H	2.73400	-1.65930	-4.19626
H	-3.98480	-4.28451	-3.87844	H	2.89222	-0.99140	-4.24304	H	-3.76691	-3.17173	-4.17060	H	-0.78769	-4.39718	2.48273
H	-5.27021	-3.47416	-2.96031	H	1.26254	-0.61317	-3.64526	H	-2.18283	-2.77934	-3.46609	H	-0.13123	-3.66538	1.00205
H	-4.55284	-2.68140	-4.38792	H	2.64148	-0.64924	-2.51595	H	-3.67801	-2.21132	-2.67696	H	-0.76450	-2.62831	2.30828

Modelling the XB between 1a and catalyst B

Calculations were performed to explore the possible hydrogen and halogen bonds between binding modes of **1a** and catalyst **B** in the precatalytic complex. To clarify this question an extensive conformational search was performed with the help of Vega program to ascertain the presence of hydrogen bonds in addition to XBs. All the significant rotational degrees of freedom were taken into account in this scan. The minimum energy structures (~100) from molecular mechanical calculations were submitted to the quantum mechanical (QM) procedure. The latter QM procedure was also performed with the Gaussian program and with the DFT method as described above (CAM-B3LYP functional), but with lower DEF2SVP basis set; this is because of the very large number of conformers generated with MM methods. Both electronic and Gibb's energies were calculated. No N-H--O hydrogen bonds were detected. In order to verify the these result an additional molecular dynamical simulation (program YASARA, AMBER99 force field) with higher temperature was performed (T = 500˚C) with subsequent QM calculations. A complexes with a N-H--O hydrogen bond was detected, but with higher energy (12.3 kcal/mol), and with wrong chirality. In an attempt to correct the chirality issue manually, some atoms become strongly overlapped. Thus the simultaneous presence of an XB and a HB from catalyst B to the substrate does not occure. Some alternative halogen bonding possibilities were also explored: I--O=C, I--O-C, in addition to the I--O=P in a similar way. From each possible halogen bonding scheme (I--O=P, I--O=C, I---O-C1, I---O-C2, I---O-C3) a minimum energy structure was determined Table S9.

From this table we can see that the initially guessed I--O=P complex is a most probable one. A critical difference between entries 1 and 2 is the presence of a stronger N-H--I (the HB in entry 2 is borderline) hydrogen bond that also results in the reduction of the length of the I--O=P XB in entry 1. Confirmation of the presence of the HB to the iodine atom was obtained with NCI analysis with NCIPLOT and can be observed in Figure S2.[10]

The Cartesian coordinates of the complexes in the table are also included in Table S10 and Table S11.

Table S9 Minimum energy complexes for possible XB interaction modes.

Entry	File	E, kcal/mol	E, AU	XB topology
1	f_min_mk_min_noopt_svp_z_33.out	0	-3393.209566	I--O=P
2	f_min_mk_min_noopt_svp_z_25.out	1.247	-3393.207579	I--O=P
3	f2_I-CO_yas_noopt_hf_svp_z_45.out	2.934	-3393.20489	I--O=C
4	f2_I-OCC1_noopt_hf_svp_z_10.out	2.979	-3393.204818	I--O-C1
5	f2_I-OCC2_noopt_hf_svp_z_7.out	3.043	-3393.204717	I--O-C2
6	f2_I-OCC3_noopt_hf_svp_z_9.out	3.832	-3393.203459	I--O-C3

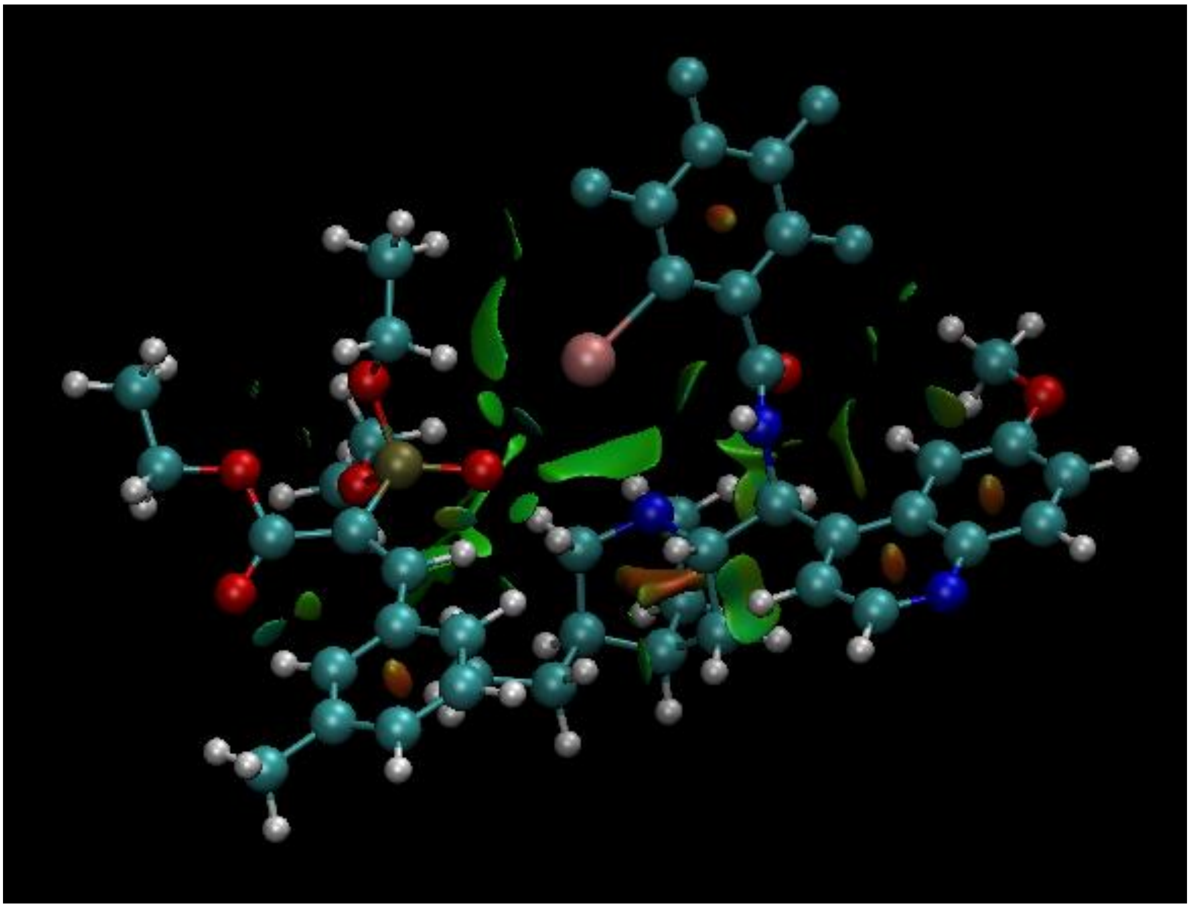

Figure S2. NCI analysis for complex [**1a** – **B**] mode f_min_mk_min_noopt_svp_z_33

Table S10 The Cartesian coordinates for the complexes

f_min_mk_min_noopt_svp_z_33				f_min_mk_min_noopt_svp_z_25				f2_I-CO_yas_noopt_hf_svp_z_45			
C	7.229	1.959	-0.567	C	5.866	0.890	-4.442	C	1.07933	6.18675	2.09234
C	7.627	2.903	-1.555	C	6.079	0.588	-5.816	C	1.10742	7.35975	1.28968
C	6.700	3.704	-2.153	C	5.041	0.176	-6.598	C	1.13817	7.25609	-0.06875
C	5.322	3.618	-1.807	C	3.728	0.041	-6.065	C	1.15424	5.98569	-0.71225
C	4.923	2.684	-0.810	C	3.509	0.358	-4.695	C	1.14873	4.80595	0.08851
C	5.905	1.853	-0.200	C	4.607	0.780	-3.893	C	1.10217	4.94070	1.50685
N	4.449	4.431	-2.452	N	2.745	-0.393	-6.892	N	1.17705	5.97097	-2.06720
C	3.184	4.345	-2.142	C	1.543	-0.530	-6.401	C	1.18866	4.81122	-2.66711
C	2.677	3.457	-1.164	C	1.210	-0.237	-5.058	C	1.19946	3.58128	-1.97539
C	3.532	2.625	-0.480	C	2.176	0.221	-4.193	C	1.19423	3.55210	-0.60024
C	3.049	1.618	0.550	C	1.890	0.505	-2.728	C	1.33806	2.20316	0.08650
C	1.707	1.953	1.224	C	0.446	0.916	-2.401	C	2.76003	2.03743	0.67138
N	1.331	0.921	2.192	N	0.299	1.243	-0.980	N	2.82859	0.88913	1.58546
C	-0.071	1.105	2.552	C	-1.125	1.294	-0.653	C	4.06207	0.98185	2.36527
C	-0.316	2.504	3.200	C	-1.877	2.347	-1.524	C	5.32151	0.99425	1.44545
C	1.057	3.200	3.293	C	-0.809	3.078	-2.361	C	4.82137	0.80970	-0.00131
C	1.690	3.351	1.902	C	-0.067	2.093	-3.275	C	3.86888	1.94493	-0.40346
C	2.147	0.964	3.405	C	0.907	2.532	-0.642	C	2.80772	-0.38245	0.85607
C	1.988	2.323	4.138	C	0.219	3.694	-1.406	C	4.04014	-0.50739	-0.07657
C	-1.381	3.325	2.470	C	-3.009	1.739	-2.355	C	6.19997	2.23210	1.63940
C	-2.787	2.752	2.615	C	-4.188	1.271	-1.509	C	6.90260	2.27345	2.99215
N	3.025	0.301	-0.068	N	2.320	-0.654	-1.958	N	0.39707	1.94236	1.15919
C	3.375	-2.103	-0.221	C	3.505	-1.915	-0.273	C	-1.70579	1.36919	2.17665
C	3.574	-0.789	0.509	C	3.112	-0.579	-0.871	C	-0.93668	1.85458	0.96091
O	4.254	-0.750	1.518	O	3.560	0.461	-0.423	O	-1.50311	2.14874	-0.06904
C	4.534	-2.841	-0.469	C	4.870	-2.197	-0.253	C	-2.12651	2.30991	3.10605
C	4.492	-4.099	-1.058	C	5.366	-3.373	0.296	C	-2.87891	1.95111	4.21866
C	3.267	-4.643	-1.413	C	4.481	-4.294	0.838	C	-3.22584	0.61874	4.39710
C	2.103	-3.917	-1.180	C	3.116	-4.026	0.825	C	-2.80621	-0.33477	3.47274
C	2.136	-2.653	-0.599	C	2.613	-2.851	0.274	C	-2.04929	0.02407	2.36275
I	0.267	-1.696	-0.341	I	0.527	-2.541	0.392	I	-1.45568	-1.47680	1.01415
F	5.734	-2.359	-0.171	F	5.747	-1.354	-0.786	F	-1.79639	3.59287	2.95728
F	5.608	-4.769	-1.288	F	6.665	-3.621	0.293	F	-3.25926	2.86340	5.09761
F	3.208	-5.839	-1.971	F	4.935	-5.418	1.365	F	-3.94342	0.25782	5.44758
F	0.955	-4.484	-1.537	F	2.312	-4.933	1.364	F	-3.15517	-1.59664	3.69450
O	8.225	1.214	-0.055	O	6.964	1.274	-3.765	O	1.02402	6.41071	3.42128
C	7.918	0.248	0.923	C	6.846	1.591	-2.399	C	0.91979	5.31596	4.29396
H	8.687	2.954	-1.811	H	7.092	0.694	-6.208	H	1.08962	8.32550	1.79709
H	6.977	4.434	-2.916	H	5.178	-0.067	-7.653	H	1.14757	8.13646	-0.71266
H	5.607	1.123	0.549	H	4.454	1.002	-2.839	H	1.03961	4.04613	2.11710
H	2.492	5.006	-2.677	H	0.763	-0.889	-7.082	H	1.19584	4.81494	-3.76372
H	1.605	3.452	-0.967	H	0.179	-0.377	-4.731	H	1.20555	2.64824	-2.54257
H	3.805	1.555	1.342	H	2.538	1.327	-2.405	H	1.18738	1.44328	-0.69937
H	0.928	1.917	0.446	H	-0.195	0.034	-2.565	H	2.94710	2.92017	1.30020
H	-0.350	0.295	3.243	H	-1.215	1.538	0.414	H	4.08487	0.13604	3.06818
H	-0.687	0.973	1.649	H	-1.552	0.287	-0.778	H	4.01582	1.89955	2.97269
H	-0.671	2.364	4.236	H	-2.333	3.100	-0.859	H	5.94392	0.11461	1.68338
H	0.939	4.190	3.759	H	-1.286	3.863	-2.967	H	5.68183	0.78043	-0.68701
H	2.711	3.751	2.011	H	0.770	2.616	-3.765	H	3.44381	1.72445	-1.39506
H	1.140	4.082	1.294	H	-0.717	1.734	-4.084	H	4.39818	2.90267	-0.50180
H	1.823	0.130	4.045	H	0.813	2.663	0.445	H	2.78124	-1.19361	1.59798
H	3.191	0.747	3.145	H	1.985	2.482	-0.842	H	1.86585	-0.45456	0.29491
H	1.566	2.187	5.146	H	-0.280	4.387	-0.711	H	4.68486	-1.34568	0.22928
H	2.961	2.823	4.265	H	0.953	4.286	-1.975	H	3.73044	-0.70500	-1.11420
H	-1.368	4.359	2.856	H	-3.361	2.484	-3.090	H	6.95671	2.26152	0.83735
H	-1.126	3.397	1.399	H	-2.624	0.889	-2.945	H	5.59206	3.14457	1.51507
H	-3.527	3.355	2.068	H	-4.970	0.808	-2.130	H	7.56124	1.40049	3.12412
H	-2.853	1.724	2.227	H	-3.882	0.532	-0.752	H	7.52169	3.17676	3.09537
H	-3.096	2.726	3.672	H	-4.647	2.116	-0.971	H	6.18445	2.27214	3.82706
H	2.453	0.182	-0.894	H	1.997	-1.565	-2.261	H	0.82552	1.46458	1.95344
H	7.491	0.712	1.829	H	6.171	2.450	-2.240	H	0.86881	5.72635	5.31008
H	7.205	-0.503	0.548	H	6.471	0.738	-1.812	H	0.01013	4.72676	4.09460
H	8.862	-0.245	1.183	H	7.851	1.857	-2.051	H	1.79973	4.65259	4.22182
O	-2.192	-0.380	-0.043	O	-2.176	-1.852	0.803	O	-5.60157	-3.81453	-1.28604
P	-3.513	-0.955	0.362	P	-3.136	-1.657	1.932	P	-4.28089	-4.47573	-1.16662
C	-4.895	0.035	-0.239	C	-2.586	-0.421	3.128	C	-2.92293	-3.53916	-1.88123
C	-6.267	-0.347	0.202	C	-3.508	-0.042	4.234	C	-1.53646	-4.05302	-1.67114
C	-4.542	1.068	-1.042	C	-1.345	0.075	2.883	C	-3.22104	-2.35240	-2.43562
C	-5.266	2.105	-1.783	C	-0.421	1.013	3.524	C	-2.30440	-1.40096	-3.07878
O	-7.172	0.404	0.464	O	-3.510	1.004	4.833	O	-0.72508	-3.53603	-0.94026
O	-6.383	-1.673	0.297	O	-4.376	-1.024	4.495	O	-1.30472	-5.17538	-2.34623
O	-3.670	-2.495	-0.062	O	-4.594	-1.148	1.515	O	-3.69865	-4.73450	0.30434
O	-3.800	-1.034	1.931	O	-3.488	-3.036	2.682	O	-4.24140	-5.97031	-1.77376
C	-6.595	2.503	-1.558	C	0.858	1.078	2.932	C	-2.58412	-0.03503	-2.97698
C	-7.192	3.499	-2.328	C	1.870	1.895	3.431	C	-1.75026	0.93238	-3.54321
C	-6.444	4.105	-3.344	C	1.577	2.678	4.553	C	-0.63070	0.49377	-4.25479
C	-5.123	3.737	-3.574	C	0.317	2.642	5.145	C	-0.34844	-0.86575	-4.38610
C	-4.534	2.752	-2.793	C	-0.684	1.820	4.641	C	-1.17505	-1.81363	-3.79974
C	-8.611	3.930	-2.065	C	3.236	1.931	2.799	C	-2.05298	2.39310	-3.35922
C	-7.620	-2.185	0.799	C	-5.383	-0.778	5.480	C	-0.07060	-5.86395	-2.09499
C	-7.512	-3.689	0.846	C	-6.551	-0.006	4.902	C	-0.17972	-6.73309	-0.86069
C	-3.533	-2.925	-1.420	C	-5.444	-1.941	0.676	C	-4.50225	-5.34904	1.31935
C	-3.000	-4.338	-1.440	C	-6.710	-1.166	0.409	C	-3.89516	-5.04175	2.66682
C	-2.994	-1.839	2.805	C	-2.467	-3.943	3.100	C	-5.05708	-6.35368	-2.88425
C	-3.296	-1.443	4.229	C	-3.129	-5.160	3.700	C	-4.31178	-6.20822	-4.19404
H	-3.461	1.114	-1.216	H	-0.895	-0.363	1.988	H	-4.27435	-2.05471	-2.38835
H	-7.157	2.034	-0.753	H	1.061	0.470	2.046	H	-3.46710	0.28691	-2.41900
H	-6.903	4.886	-3.957	H	2.349	3.334	4.965	H	0.02443	1.22972	-4.72847
H	-4.548	4.224	-4.364	H	0.108	3.272	6.012	H	0.52164	-1.18618	-4.96346
H	-3.494	2.467	-2.969	H	-1.673	1.814	5.091	H	-0.96835	-2.87717	-3.93141
H	-8.646	4.963	-1.685	H	3.264	1.405	1.834	H	-1.85993	2.68077	-2.31444
H	-9.213	3.903	-2.986	H	3.566	2.967	2.632	H	-1.43397	3.02146	-4.01434
H	-9.095	3.283	-1.321	H	3.982	1.460	3.460	H	-3.10978	2.61033	-3.57496
H	-7.804	-1.750	1.793	H	-5.691	-1.776	5.817	H	0.10031	-6.46487	-2.99760
H	-8.437	-1.846	0.144	H	-4.932	-0.237	6.323	H	0.73633	-5.12509	-1.99542
H	-6.677	-4.000	1.489	H	-7.345	0.093	5.656	H	-1.02838	-7.42605	-0.95122
H	-8.441	-4.121	1.246	H	-6.237	1.002	4.599	H	0.74143	-7.32033	-0.73067
H	-7.345	-4.103	-0.159	H	-6.965	-0.526	4.026	H	-0.32532	-6.11592	0.03660
H	-4.526	-2.866	-1.895	H	-4.909	-2.169	-0.260	H	-5.52881	-4.95706	1.24410
H	-2.850	-2.246	-1.955	H	-5.656	-2.892	1.190	H	-4.53551	-6.43435	1.12999
H	-1.981	-4.378	-1.029	H	-7.386	-1.755	-0.227	H	-2.86747	-5.42847	2.72629
H	-3.648	-5.004	-0.852	H	-7.232	-0.937	1.350	H	-3.86407	-3.95783	2.84461
H	-2.967	-4.711	-2.475	H	-6.488	-0.219	-0.102	H	-4.48995	-5.51248	3.46363
H	-3.231	-2.898	2.618	H	-1.842	-4.212	2.234	H	-5.33336	-7.40310	-2.70539
H	-1.931	-1.679	2.565	H	-1.819	-3.445	3.841	H	-5.97813	-5.75088	-2.87578
H	-3.042	-0.388	4.402	H	-3.751	-4.877	4.561	H	-4.92317	-6.59680	-5.02240
H	-4.363	-1.583	4.454	H	-3.770	-5.658	2.958	H	-4.08120	-5.15387	-4.40551
H	-2.709	-2.062	4.924	H	-2.367	-5.878	4.038	H	-3.36403	-6.76416	-4.16439

Table S11 The Cartesian coordinates for the complexes

f2_I-OCC1_noopt_hf_svp_z_10			
C	-2.51149	-1.70694	5.75509
C	-1.35883	-2.21350	6.40782
C	-0.14410	-2.15664	5.77476
C	-0.00558	-1.59605	4.48108
C	-1.16344	-1.07119	3.82954
C	-2.40764	-1.15340	4.49731
N	1.23326	-1.58733	3.92363
C	1.35966	-1.07648	2.72831
C	0.28405	-0.52297	1.99173
C	-0.97974	-0.49764	2.52949
C	-2.11256	0.16900	1.75986
C	-1.74595	1.62153	1.40645
N	-2.67755	2.18444	0.41764
C	-2.08375	3.40377	-0.12698
C	-1.82275	4.45920	0.99284
C	-2.35678	3.86065	2.31012
C	-1.64044	2.54435	2.64415
C	-3.98050	2.48991	1.00680
C	-3.84827	3.55227	2.12981
C	-0.35932	4.89977	1.06923
C	0.08495	5.73262	-0.12832
N	-2.44305	-0.50385	0.51457
C	-3.56262	-2.14468	-0.86458
C	-3.03779	-1.71247	0.49474
O	-3.19431	-2.41864	1.46753
C	-4.94419	-2.13570	-1.02424
C	-5.54785	-2.58062	-2.19474
C	-4.75279	-3.04774	-3.23225
C	-3.36867	-3.05983	-3.08998
C	-2.76718	-2.61087	-1.91806
I	-0.67116	-2.67246	-1.80946
F	-5.72587	-1.67557	-0.05469
F	-6.86328	-2.55348	-2.32715
F	-5.31051	-3.47498	-4.35329
F	-2.64915	-3.51201	-4.10811
O	-3.74940	-1.73695	6.30482
C	-3.93735	-2.33398	7.55801
H	-1.42884	-2.65273	7.40226
H	0.76060	-2.54396	6.24561
H	-3.32863	-0.81741	4.02637
H	2.36136	-1.06821	2.28351
H	0.48084	-0.09836	1.00478
H	-3.01064	0.17015	2.39387
H	-0.77879	1.58843	0.88854
H	-2.75955	3.79921	-0.90086
H	-1.14227	3.11948	-0.62068
H	-2.42434	5.36132	0.78486
H	-2.21729	4.58538	3.12707
H	-2.11598	2.08754	3.52710
H	-0.58876	2.71401	2.91332
H	-4.64134	2.84241	0.20070
H	-4.42610	1.55757	1.38306
H	-4.39490	4.47234	1.87063
H	-4.27381	3.18366	3.07580
H	-0.20680	5.48381	1.99281
H	0.28927	4.01187	1.15337
H	-0.51556	6.65146	-0.22113
H	1.14017	6.03322	-0.04102
H	-0.02087	5.17350	-1.07099
H	-2.53030	0.12819	-0.28012
H	-3.64798	-3.39929	7.55688
H	-3.37463	-1.81558	8.35416
H	-5.00895	-2.26205	7.78341
O	0.84759	1.34545	-0.76710
P	1.92866	1.29812	-1.78721
C	2.49728	-0.35813	-2.23244
C	2.83375	-1.18757	-1.03911
C	2.60079	-0.73905	-3.51887
C	3.15196	-1.98522	-4.07497
O	3.35012	-0.72551	-0.04972
O	2.46411	-2.46063	-1.15144
O	1.57244	1.89996	-3.22982
O	3.22344	2.16146	-1.39207
C	4.29231	-2.60171	-3.54215
C	4.81969	-3.76534	-4.09710
C	4.17477	-4.32100	-5.20936
C	3.05198	-3.71478	-5.76011
C	2.54957	-2.54083	-5.20800
C	6.05792	-4.40895	-3.53147
C	2.80942	-3.34787	-0.07077
C	2.60990	-4.76635	-0.54430
C	1.09344	3.24041	-3.37876
C	1.10580	3.59316	-4.84621
C	3.63104	2.39813	-0.03577
C	4.11674	3.82246	0.08776
H	2.24215	-0.02064	-4.26151
H	4.79858	-2.14484	-2.68881
H	4.56992	-5.23699	-5.65639
H	2.56654	-4.15639	-6.63278
H	1.67252	-2.05957	-5.64627
H	6.40606	-3.89100	-2.62737
H	5.87489	-5.46236	-3.26938
H	6.88098	-4.39676	-4.26294
H	3.85026	-3.14844	0.22216
H	2.17170	-3.10667	0.79217
H	1.56161	-4.94993	-0.81909
H	3.24129	-4.98279	-1.41796
H	2.87745	-5.46352	0.26274
H	0.07605	3.30323	-2.96139
H	1.74269	3.91919	-2.80323
H	0.46444	2.90607	-5.41679
H	0.73159	4.61703	-4.99177
H	2.12607	3.53453	-5.25138
H	2.78069	2.20290	0.63338
H	4.42025	1.67342	0.20769
H	3.30531	4.53480	-0.12303
H	4.48107	4.00764	1.10920
H	4.94082	4.01766	-0.61380

f2_I-OCC2_noopt_hf_svp_z_7			
C	1.40120	-0.55533	-6.43380
C	1.21290	-1.49006	-7.48975
C	0.37555	-2.55285	-7.32338
C	-0.32655	-2.75190	-6.10042
C	-0.13912	-1.81930	-5.04091
C	0.74309	-0.71795	-5.23451
N	-1.14426	-3.82823	-6.00516
C	-1.79550	-4.00794	-4.88685
C	-1.69089	-3.14514	-3.77279
C	-0.86400	-2.04838	-3.82856
C	-0.65869	-1.12897	-2.63666
C	-1.79141	-1.09904	-1.59642
N	-3.10113	-0.89627	-2.21611
C	-3.22424	0.44052	-2.79473
C	-3.08514	1.55934	-1.71810
C	-2.79504	0.84775	-0.38387
C	-1.53914	-0.02619	-0.49926
C	-4.13957	-1.08180	-1.20500
C	-3.98043	-0.06277	-0.04439
C	-2.06119	2.63609	-2.08467
C	-2.46870	3.47752	-3.28974
N	0.61053	-1.48252	-1.99666
C	2.93368	-1.24536	-1.36662
C	1.62946	-0.61840	-1.82082
O	1.56424	0.57539	-2.05855
C	3.94767	-1.29477	-2.31901
C	5.20820	-1.79989	-2.02319
C	5.46412	-2.27043	-0.74286
C	4.46175	-2.22684	0.22159
C	3.19896	-1.72072	-0.07511
I	1.80211	-1.66117	1.49530
F	3.72563	-0.86784	-3.55746
F	6.14935	-1.84203	-2.94803
F	6.65497	-2.75538	-0.44197
F	4.75526	-2.68009	1.43081
O	2.24439	0.45643	-6.71414
C	2.46620	1.44892	-5.74197
H	1.75567	-1.32112	-8.42110
H	0.21004	-3.28408	-8.11575
H	0.90450	-0.00440	-4.43013
H	-2.45757	-4.88010	-4.82984
H	-2.29225	-3.34878	-2.88826
H	-0.51237	-0.10637	-3.00093
H	-1.82823	-2.09475	-1.12377
H	-2.46724	0.55817	-3.58512
H	-4.19814	0.49332	-3.30347
H	-4.05934	2.06730	-1.60779
H	-2.65504	1.58549	0.41994
H	-1.34537	-0.48847	0.47640
H	-0.66356	0.59029	-0.74727
H	-5.11597	-0.97591	-1.70177
H	-4.08315	-2.11850	-0.83721
H	-4.89449	0.53888	0.07964
H	-3.78241	-0.55638	0.91744
H	-1.91575	3.29734	-1.21351
H	-1.07776	2.17409	-2.27614
H	-3.42465	3.99448	-3.10913
H	-1.71387	4.24473	-3.51840
H	-2.59442	2.86259	-4.19472
H	0.77606	-2.46397	-1.80441
H	2.89539	1.02772	-4.81979
H	3.17443	2.16313	-6.17958
H	1.53264	1.97883	-5.48636
O	-2.03076	-0.09930	2.77349
P	-1.08144	-0.18393	3.91504
C	0.15460	1.12433	3.95317
C	0.87644	1.36318	5.23506
C	0.32270	1.78582	2.78345
C	1.23254	2.83910	2.32000
O	1.21086	2.43436	5.67647
O	1.09260	0.21961	5.88555
O	-1.80808	-0.17303	5.35572
O	-0.21599	-1.54242	3.97551
C	1.47655	2.86322	0.93467
C	2.34378	3.78435	0.35006
C	2.95673	4.72330	1.18546
C	2.70670	4.73442	2.55475
C	1.85504	3.79990	3.13152
C	2.58653	3.77823	-1.13594
C	1.69333	0.31240	7.17830
C	1.75265	-1.07830	7.76121
C	-3.11001	0.40633	5.52292
C	-4.18438	-0.65657	5.48724
C	-0.84265	-2.81050	4.21260
C	0.20469	-3.78712	4.69104
H	-0.31647	1.40772	1.97817
H	0.99395	2.12108	0.29422
H	3.63798	5.46125	0.75319
H	3.18624	5.48493	3.18695
H	1.66101	3.80988	4.20111
H	1.89663	4.47015	-1.64680
H	3.60917	4.10637	-1.37297
H	2.42795	2.77738	-1.56045
H	2.69297	0.76163	7.07496
H	1.09634	0.99772	7.79927
H	2.35513	-1.74330	7.12581
H	2.20780	-1.04962	8.76170
H	0.74284	-1.50409	7.85025
H	-3.08802	0.92171	6.49390
H	-3.28589	1.15633	4.73719
H	-5.17080	-0.20589	5.67364
H	-4.00443	-1.42170	6.25695
H	-4.20702	-1.14009	4.50028
H	-1.63150	-2.68174	4.96927
H	-1.31731	-3.15039	3.27820
H	0.99442	-3.92009	3.93783
H	0.66965	-3.42630	5.61917
H	-0.25533	-4.76716	4.88590

f2_I-OCC3_noopt_hf_svp_z_9			
C	2.88156	-0.47708	-1.43647
C	4.21118	-0.47804	-0.94208
C	4.52974	-1.24575	0.14626
C	3.55914	-2.04600	0.79851
C	2.22362	-2.05008	0.29719
C	1.91237	-1.24681	-0.82546
N	3.94928	-2.77105	1.87989
C	3.05671	-3.50959	2.47651
C	1.70725	-3.60419	2.05377
C	1.27104	-2.88805	0.96638
C	-0.17280	-2.92792	0.49008
C	-0.91862	-4.22984	0.83336
N	-2.33845	-4.14485	0.48812
C	-3.03828	-5.26666	1.10568
C	-2.49168	-6.63845	0.60041
C	-1.39839	-6.32405	-0.44019
C	-0.27468	-5.48416	0.18400
C	-2.56062	-4.18024	-0.95902
C	-2.03018	-5.50390	-1.57111
C	-2.02272	-7.55768	1.72974
C	-3.15874	-8.06789	2.60989
N	-0.90583	-1.77780	0.99708
C	-2.57152	-0.03751	0.93922
C	-1.61605	-0.95185	0.20400
O	-1.54353	-0.93808	-1.01238
C	-3.92574	-0.33608	0.82026
C	-4.89748	0.44756	1.43245
C	-4.50823	1.55534	2.17335
C	-3.15731	1.86425	2.29564
C	-2.18163	1.08315	1.68402
I	-0.18605	1.71988	1.82837
F	-4.32271	-1.39081	0.12145
F	-6.17864	0.14359	1.31551
F	-5.41955	2.31311	2.75840
F	-2.83661	2.94253	3.00173
O	2.49338	0.25949	-2.50161
C	3.47192	0.90260	-3.29313
H	4.96782	0.15392	-1.40213
H	5.54127	-1.25991	0.55451
H	0.90263	-1.20512	-1.23415
H	3.38576	-4.08684	3.34903
H	1.03635	-4.26021	2.60924
H	-0.17506	-2.81462	-0.59982
H	-0.90020	-4.34151	1.92994
H	-4.11167	-5.15894	0.89075
H	-2.92487	-5.18516	2.19912
H	-3.29829	-7.16932	0.06598
H	-0.98545	-7.26409	-0.83740
H	0.44336	-5.19707	-0.60113
H	0.29672	-6.06446	0.92185
H	-3.64143	-4.06542	-1.12583
H	-2.09315	-3.29969	-1.41787
H	-2.84212	-6.07812	-2.04361
H	-1.28217	-5.30671	-2.35477
H	-1.48805	-8.41820	1.29298
H	-1.28369	-7.02994	2.35735
H	-3.70629	-7.24208	3.09041
H	-3.88793	-8.64539	2.02001
H	-2.78470	-8.72295	3.41070
H	-1.08343	-1.74268	1.99393
H	3.97766	1.69517	-2.72270
H	4.20831	0.17772	-3.67971
H	2.93520	1.34504	-4.14207
O	3.83037	2.72730	-0.70741
P	3.01060	3.70252	0.05725
C	1.43868	4.10437	-0.71208
C	0.74224	5.35770	-0.28297
C	0.95528	3.23706	-1.62084
C	-0.29642	3.33546	-2.38577
O	1.22698	6.45960	-0.35901
O	-0.47334	5.11895	0.19707
O	2.55637	3.24990	1.53873
O	3.75876	5.07912	0.40881
C	-0.99014	2.15291	-2.66816
C	-2.20805	2.16559	-3.35283
C	-2.70737	3.39685	-3.78560
C	-2.00898	4.57907	-3.54537
C	-0.81030	4.55566	-2.84489
C	-2.93809	0.87839	-3.62870
C	-1.26062	6.25077	0.57895
C	-2.65936	5.76572	0.87256
C	3.50632	3.07319	2.60467
C	4.18333	1.72162	2.54090
C	4.40420	5.86301	-0.60188
C	5.30510	6.86618	0.07599
H	1.54313	2.33116	-1.80576
H	-0.58677	1.19890	-2.32057
H	-3.65561	3.43015	-4.32856
H	-2.40575	5.52850	-3.91186
H	-0.25741	5.48213	-2.67784
H	-2.56658	0.41062	-4.55539
H	-2.78221	0.15816	-2.81359
H	-4.01661	1.04872	-3.75797
H	-0.79213	6.72599	1.45496
H	-1.23982	6.98896	-0.23712
H	-3.28946	6.60959	1.18942
H	-3.10771	5.31596	-0.02517
H	-2.65775	5.01342	1.67332
H	4.23662	3.89518	2.56390
H	2.92425	3.18816	3.52999
H	4.87778	1.61158	3.38751
H	3.44772	0.90583	2.59242
H	4.74509	1.61027	1.60377
H	4.97501	5.19244	-1.26379
H	3.62351	6.37025	-1.18862
H	6.07935	6.36008	0.67089
H	5.80093	7.49748	-0.67608
H	4.72023	7.51520	0.74310

6. Determination of product 3a stability in the presence of catalyst B

Compound **3** (18.7 mg, 0.048 mmol) and internal standard $(MeO)_3C_6H_3$ (4.1 mg, 0.024 mmol) were dissolved in toluene (0.24 mL) and catalyst **B** (3.0 mg, 0.005 mmol) was added. The mixture was stirred at RT for seven days. At the stated time intervals samples were taken for ^{1}H qNMR analysis. Also, samples taken on the first and seventh day were additionally purified by preparative TLC (R_f=0.43 (major), 0.31 (minor); DCM/EtOAc 2:0.3) and the enantiomeric excess of **3** was determined by HPLC analysis on a chiral stationary phase.

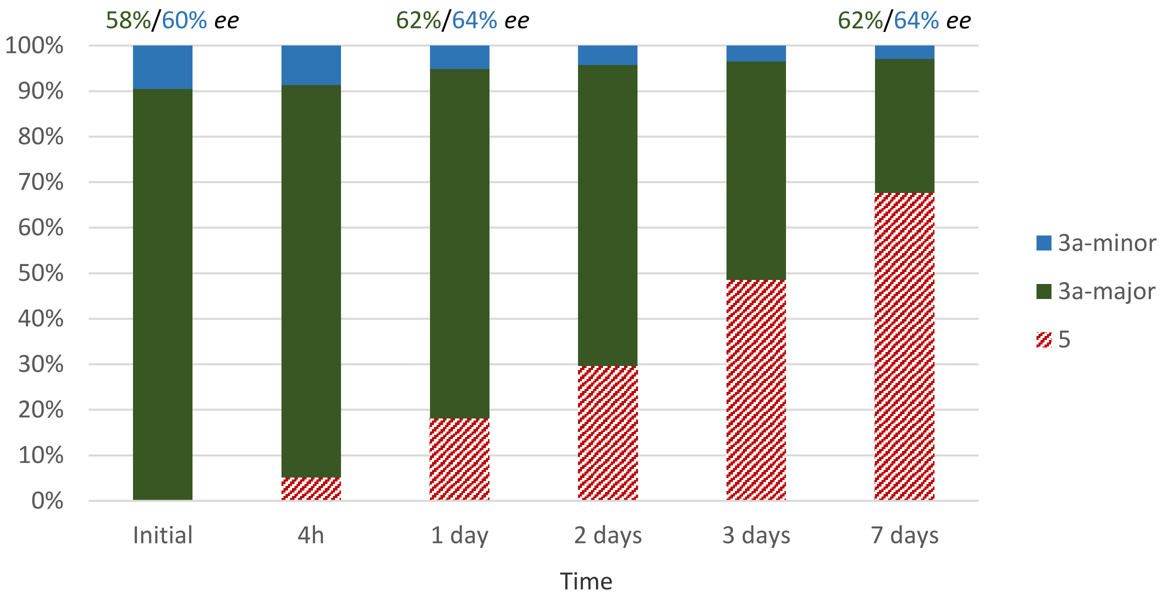

Figure S3. Catalyst **B** facilitated retro-Michael reaction of **3a**.

7. Single Crystal X-ray diffraction data

Single crystal X-ray diffraction data was collected at 123K on Rigaku Compact HomeLab diffractometer, equipped with a Saturn 944 HG CCD detector and Oxford Cryostream cooling system using monochromatic Cu-*Kα* radiation (1.54178Å) from a MicroMax^{TM}-003 sealed tube microfocus X-ray source. The strategy of data collections was calculated and implemented through the program package *HKL-3000*.[11] Data was collected using *ω*-scans. CrysAlisPro[12] was used for data reduction and empirical absorption correction using spherical harmonics implemented in *SCALE3 ABSPACK* scaling algorithm.[13] The structures were solved using *SHELXT*[14] and refined by full-matrix least-squares method against F^2 with *SHELXL-2016*[15] through *OLEX2*[16] program package. All non-hydrogen atoms were refined with anisotropic atomic displacement parameters. Hydrogen atoms attached to carbon atoms were treated as riding atoms, using isotropic displacement parameters U_{iso}(H) = 1.2; U_{iso}(C) for CH and CH_2; U_{iso}(H) = 1.5; U_{iso}(C or O) for CH_3 and OH. Appropriate restraints were applied to the geometry and thermal displacement parameters of the atoms involved in the disordered parts of the structures. Restrain SADI was used to fix the distance between two carbon atoms (C13-C13a bond distance was fixed to be the same as C13-C13b) to be equal within the standard uncertainty s value 0.02. Restrain SIMU restrains the anisotropic displacement parameters of adjacent atoms (C13, C13a and C13b) to be similar. The default values for the standard deviations are 0.04 SIMU (0.08 for terminal atoms, which tend to move more strongly). The absolute configuration was assigned based on the anomalous dispersion effects – absolute configuration is S at C7 and S at C11 as shown on Figure S2. The figures were drawn using the programs Mercury CSD 2.0.[17] The crystallographic data is deposited with the Cambridge Crystallographic Data Centre (CCDC 2063122) and can be obtained free of charge via www.ccdc.cam.ac.uk/data_request/cif.

Single Crystal X-ray diffraction analysis

Crystallographic details for the compound **3d** (major enantiomer of minor diastereoisomer).

CCDC 2063122 : $C_{18}H_{21.53}N_3O_7P$, monoclinic, space group $P2_1$, a = 10.0914(10) Å, b = 9.9851(10) Å, c = 10.5204(10) Å, α = 90°, β = 92.720(10)°, γ = 90°, V = 1058.879(18) Å^3, Z = 2, T = 123.0 K, μ(CuKα) = 1.541 mm^{-1}, D_{calc} = 1.326 g/cm^3, 18922 reflections measured (8.414 ° ≤ 2Θ ≤ 134.592°), of which 3740 unique (3738 with $I > 2\sigma(I)$), R_{int} = 0.0254, $R_1[F^2 > 2\sigma(F^2)]$ = 0.0351 and wR_2 (all data) = 0.0940, S = 1.046, Flack x = 0.011(5); absolute configuration determined by anomalous diffraction effects using 1718 quotients [(I+)-(I-)]/[(I+)+(I-)].

Colourless crystals of compound were obtained from a mixture of methyl *tert*-butyl ether and hexane. The structure crystallized in the monoclinic space group $P2_1$. One ethyl group was found to be disordered in the crystal structure. The two disorder components were modelled, using the SHELXL. The relative occupancy of the respective disorder components was allowed to refine freely and is indicated on Figure S4.

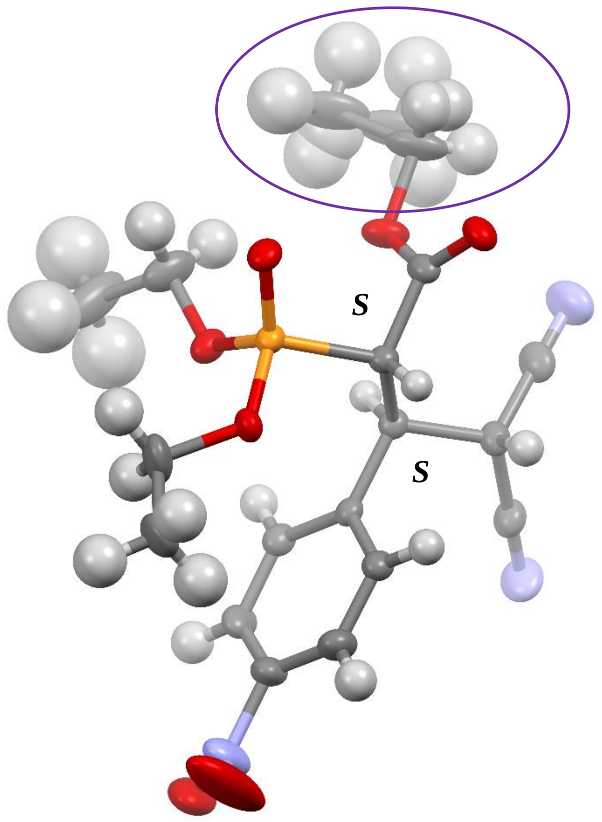

Figure S4. Crystal structure. of major enantiomer of minor diatereoisomer of **3d**. Colour-scheme of elements: C – dark grey, N – blue, O – red, P – orange, H – light grey.

8. ^{1}H NMR titration experiments

^{1}H NMR titration experiments were performed on a Bruker AVANCE III 400 MHz spectrometer. First, a 5 mM host solution of XB donor was prepared in toluene-d8. Then, the guest solution was prepared by dissolving the XB donor (final concentration: 5 mM) in a solution of XB acceptor (1250 mM) in toluene-d8 (this was done to ensure the concentration of XB donor would not change throughout the titration experiment). All the solutions were prepared using Hamilton® Gastight syringes and samples were weighed on a microbalance with an accuracy of 6 µg. During the titration experiment small aliquots from the guest solution were added increasingly (from 0 to 100 µl) to the NMR tube containing 500 µl of the host (XB donor) stock solution. After every addition the sample was thoroughly shaken and then the spectrum was measured. For the measurements 16 scans (32 scans for the ^{19}F measurements) were collected with a 2 s relaxation delay and acquisition time set to 2 s at 296 K. The precision of the chemical shifts was red within 0.0005 ppm (SW = 8224 with an AC = 16446 for ^{1}H, SW = 89286 with an AC = 178566 for ^{19}F). The signals belonging to the methyl group protons of the XB donor were used as input for the fitting calculations. All the NMR experiments showed downfield ^{1}H shifts upon addition of the guest solution. For ^{19}F measurements, the signals belonging to the fluorine atom *ortho* to the iodine atom in catalyst **B** was used as input for the fitting calculations and it demonstrated an upfield chemical shift. The chemical shifts were referenced based on the toluene-d8 residual peak at 2.09 ppm or the fluorine signal of 1,4-bis(trifluoromethyl)benzene at -63.15. K_{obs} values were determined using nonlinear regression analysis. For the fitting of the binding data 1:1 binding isotherm of BindFit was used (freely available at http://supramolecular.org).[18,19] Herein, the given standard error after each trial depicts error coming from curve fit calculations. Also, the standard error based on unbiased estimation of standard deviation is given for the calculated mean value of the two parallel experiments (or the largest curve fitting error, if this is larger).

8.1. ^{1}H NMR titration data of [1b-9] complex

Trial 1

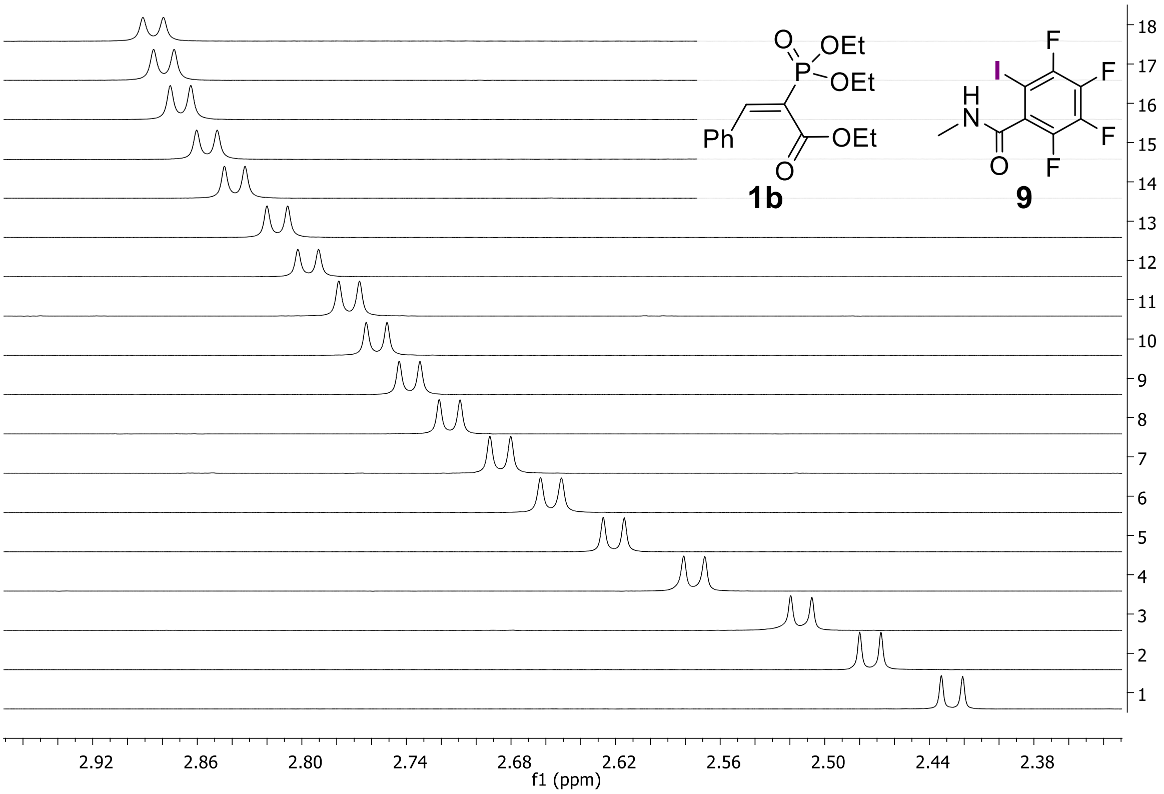

Figure S5. Outtakes of ^{1}H NMR spectra (toluene-d8, 400 MHz) of **9** (5.0 mM) titration with **1b** at 296 K. Showing the collected 18 data points of **9** methyl protons (*N*-CH_3) after reaching 0.0, 2.0, 4.0, 7.8, 11.5, 15.1, 18.6, 22.8, 26.9, 30.8, 34.6, 41.9, 48.6, 60.9, 71.8, 85.9, 98.0 and 108.0 equiv. of **1b**.

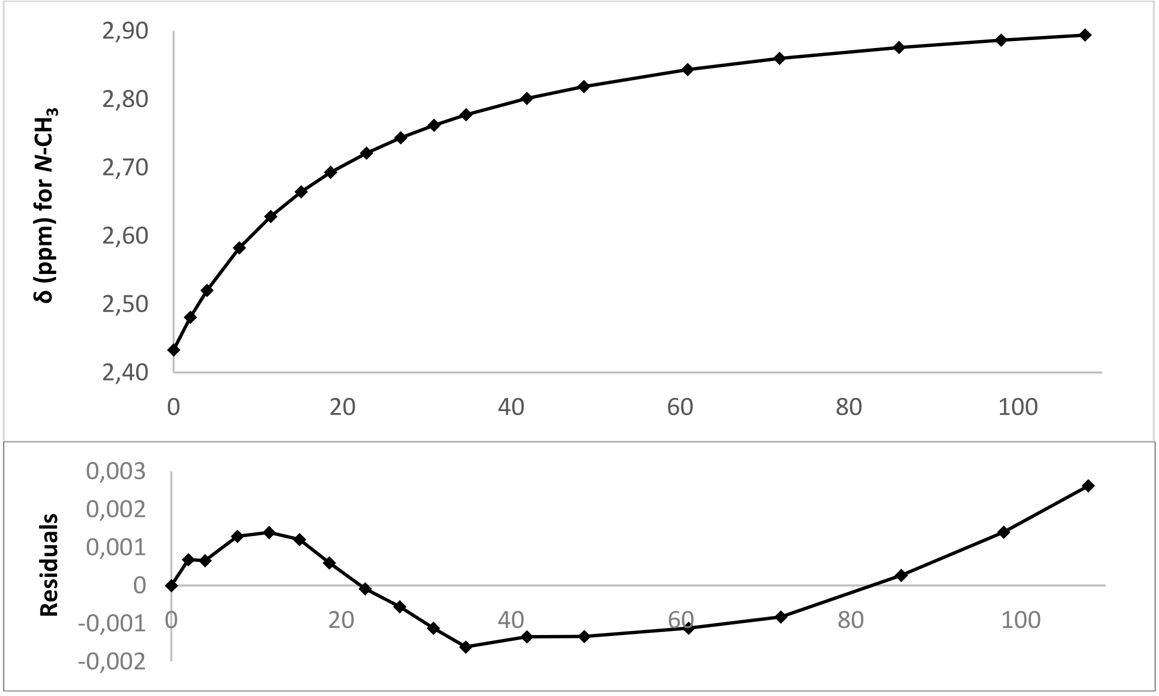

Figure S6. Calculated chemical shift values and residuals of the binding isotherm for the [**1b-9**] complex obtained with BindFit. The fitting of the methyl protons (*N*-CH_3) gave an average value of K_{obs} = 10.02 ± 0.06 M^{-1}.

For full details see: http://app.supramolecular.org/bindfit/view/e21b1954-b22e-49a9-ac94-25a622eb85f8

Trial 2

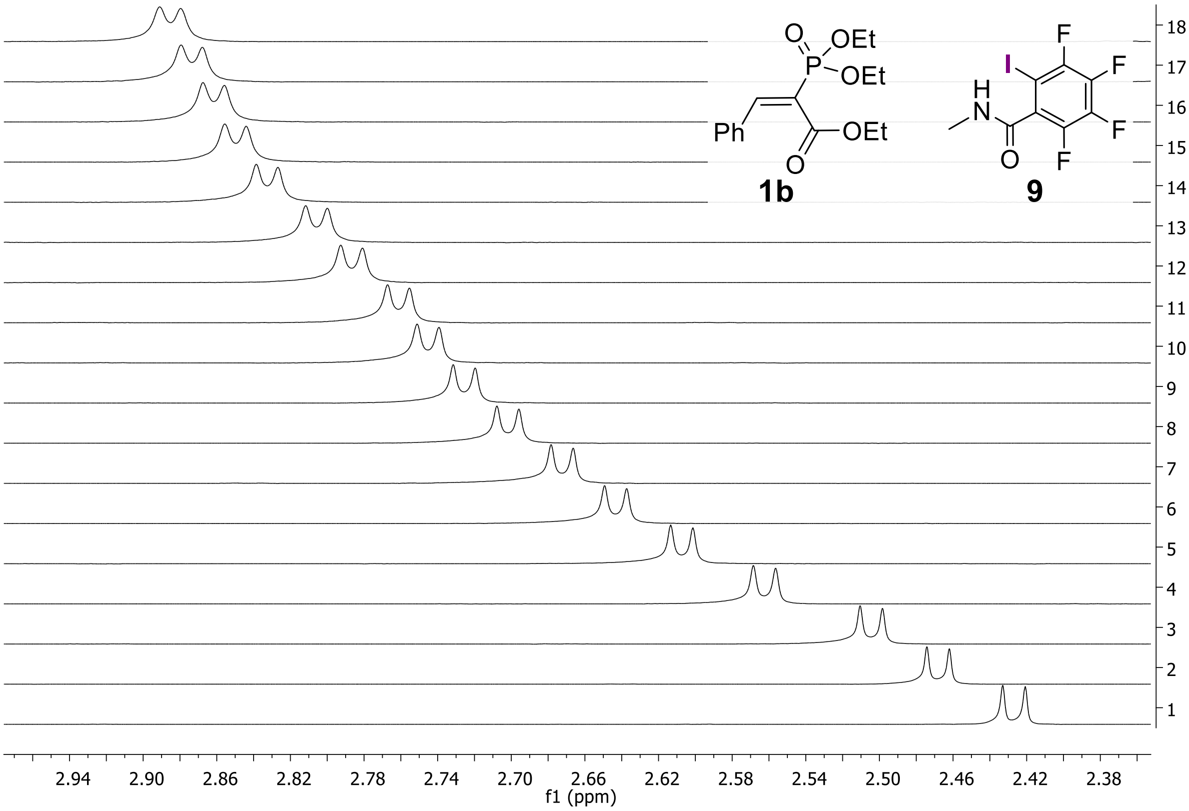

Figure S7. Outtakes of ^{1}H NMR spectra (toluene-d8, 400 MHz) of **9** (5.0 mM) titration with **1b** at 296 K. Showing the collected 18 data points of **9** methyl protons (*N*-CH_3) after reaching 0.0, 2.0, 3.9, 7.8, 11.5, 15.1, 18.6, 22.8, 26.8, 30.8, 34.5, 41.7, 48.5, 60.7, 71.6, 81.2, 93.9 and 111.3 equiv. of **1b**.

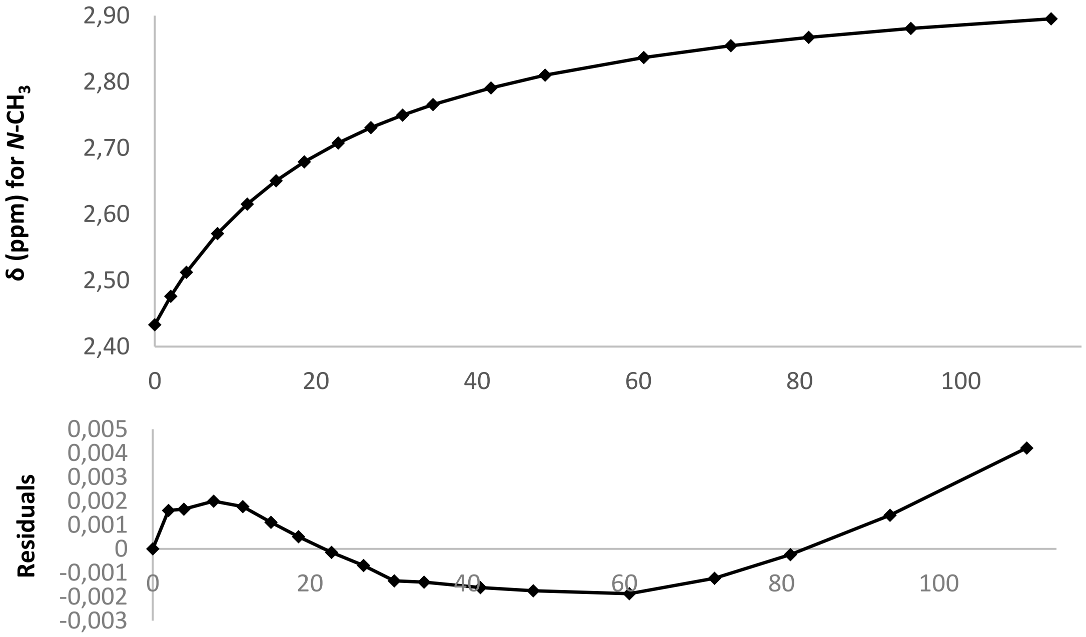

Figure S8. Calculated chemical shift values and residuals of the binding isotherm for the [**1b**-**9**] complex obtained with BindFit. The fitting of the methyl protons (*N*-CH_3) gave an average value of K_{obs} = 8.69 ± 0.08 M^{-1}.

For full details see: http://app.supramolecular.org/bindfit/view/3ef43570-8d35-4831-ab9c-5ae401d0b069

Mean value of trial 1 and 2: 9.4 ± 0.7 M^{-1}

8.2. 1H NMR titration data of [1b-10] complex

Trial 1

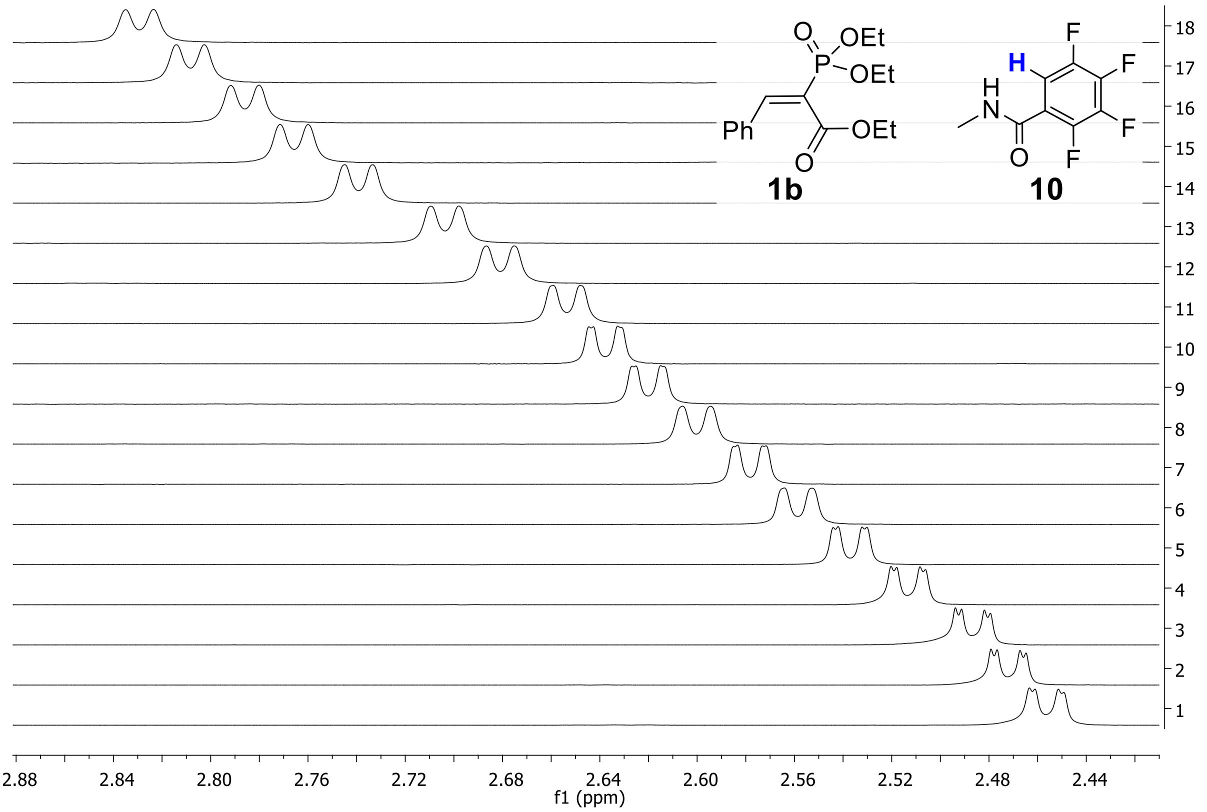

Figure S9. Outtakes of ^{1}H NMR spectra (toluene-d8, 400 MHz) of **10** (5.0 mM) titration with **1b** at 296 K. Showing the collected 18 data points of **10** methyl protons (*N*-CH_3) after reaching 0.0, 2.0, 3.9, 7.8, 11.5, 15.0, 18.5, 22.7, 26.8, 30.7, 34.5, 41.7, 48.4, 60.7, 71.5, 81.1, 93.8 and 108.0 equiv. of **1b**.

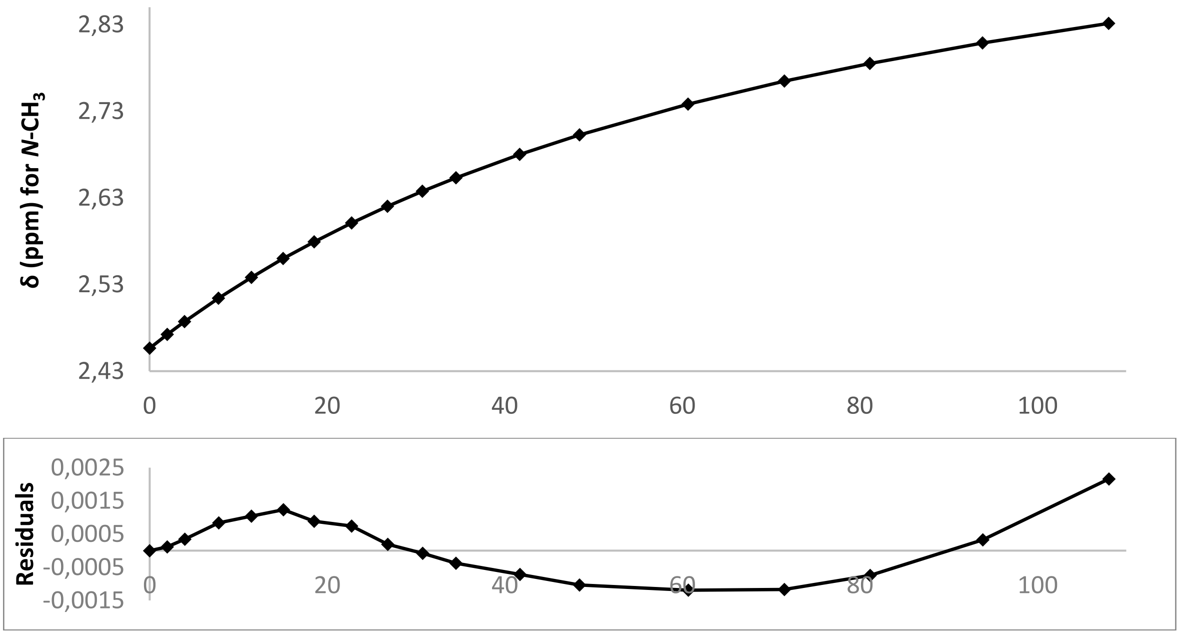

Figure S10. Calculated chemical shift values and residuals of the binding isotherm for the [**1b-10**] complex obtained with BindFit. The fitting of the methyl protons (*N*-CH_3) gave an average value of K_{obs} = 2.54 ± 0.01 M^{-1}.

For full details see: http://app.supramolecular.org/bindfit/view/b3386de7-e1bb-4390-bd49-9f2a96afa268

Trial 2

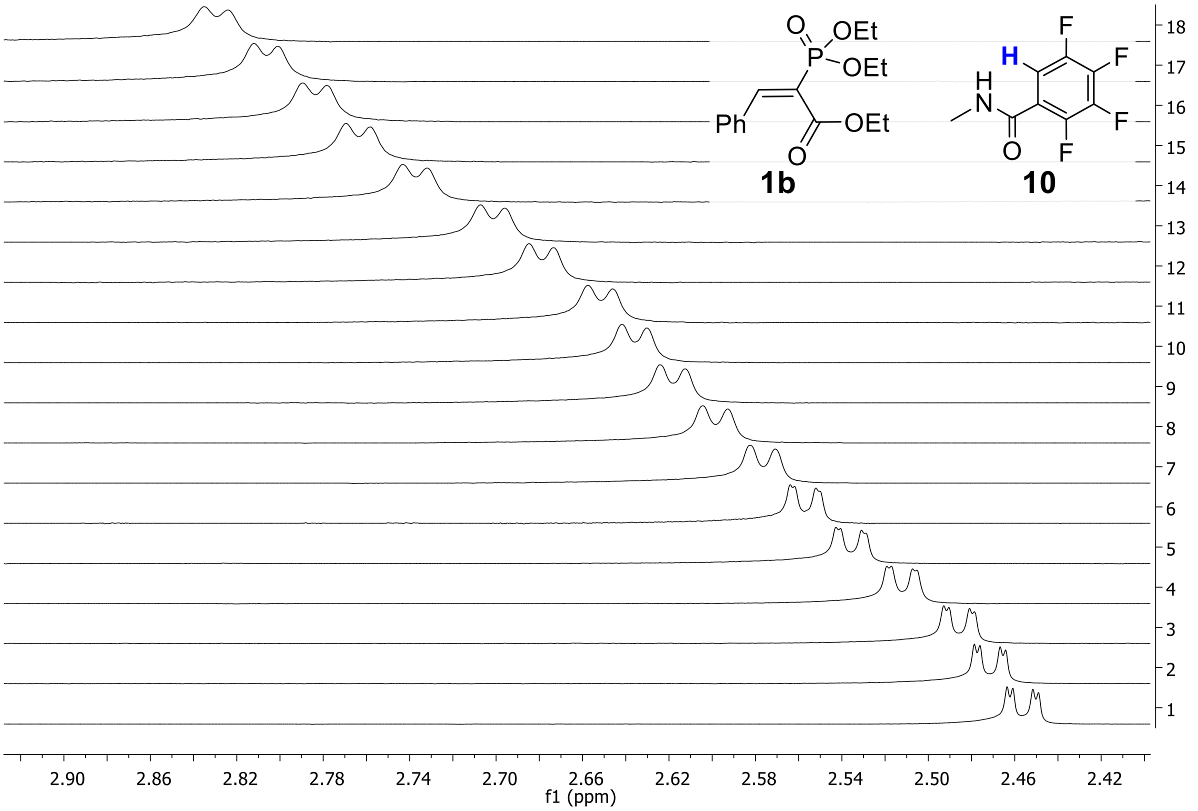

Figure S11. Outtakes of ^{1}H NMR spectra (toluene-d8, 400 MHz) of **10** (5.0 mM) titration with **1b** at 296 K. Showing the collected 18 data points of **10** methyl protons (*N*-CH_3) after reaching 0.0, 2.0, 3.9, 7.8, 11.5, 15.1, 18.5, 22.7, 26.8, 30.7, 34.5, 41.7, 48.4, 60.7, 71.5, 81.1, 93.8 and 109.6 equiv. of **1b**.

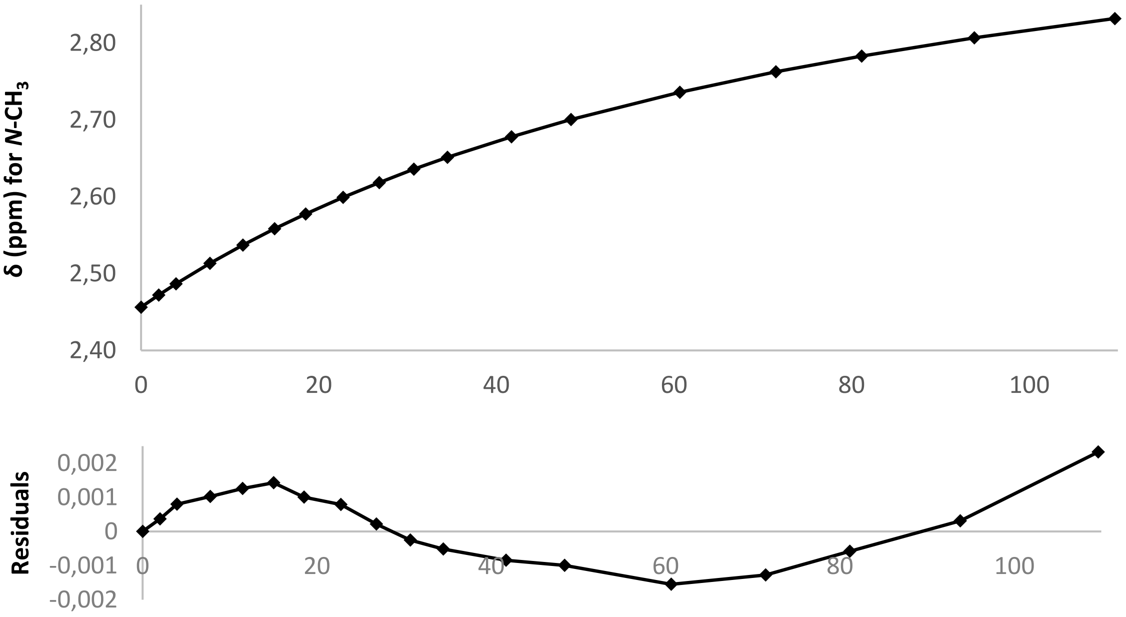

Figure S12. Calculated chemical shift values and residuals of the binding isotherm for the [**1b-10**] complex obtained with BindFit. The fitting of the methyl protons (*N*-CH_3) gave an average value of K_{obs} = 2.51 ± 0.01 M⁻¹.

For full details see: http://app.supramolecular.org/bindfit/view/713702d3-8915-4012-9ddf-c048d3cf1b80

Mean value of trial 1 and 2: 2.52 ± 0.02 M⁻¹

8.3 ^{1}H NMR titration data of [1b-11] complex

Trial 1

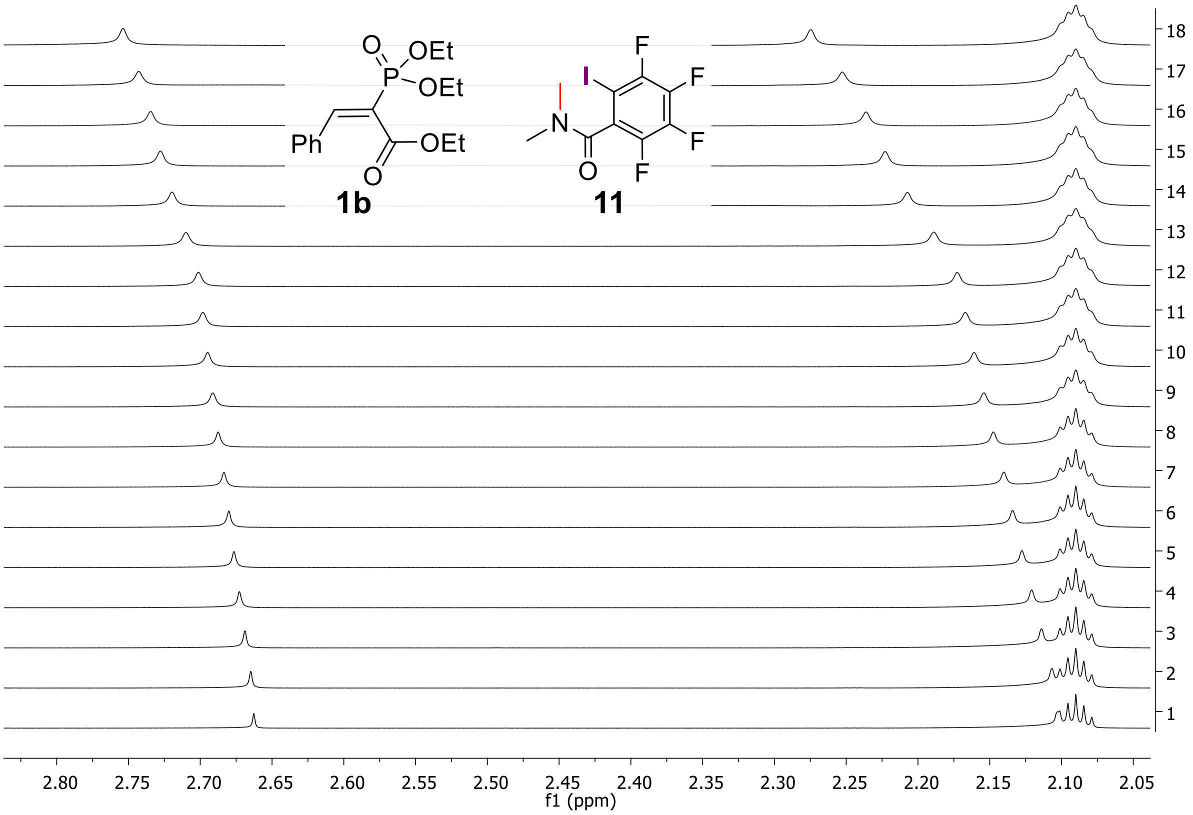

Figure S13. Outtakes of ^{1}H NMR spectra (toluene-d8, 400 MHz) of **11** (5.0 mM) titration with **1b** at 296 K. Showing the collected 18 (x2) data points of **11** methyl protons (*N*-CH_3) after reaching 0.0, 2.0, 5.9, 9.6, 13.3, 16.8, 18.5, 20.3, 24.4, 28.4, 32.3, 36.1, 39.7, 49.8, 61.9, 72.6, 82.2, 94.8 and 112.0 equiv. of **1b**.

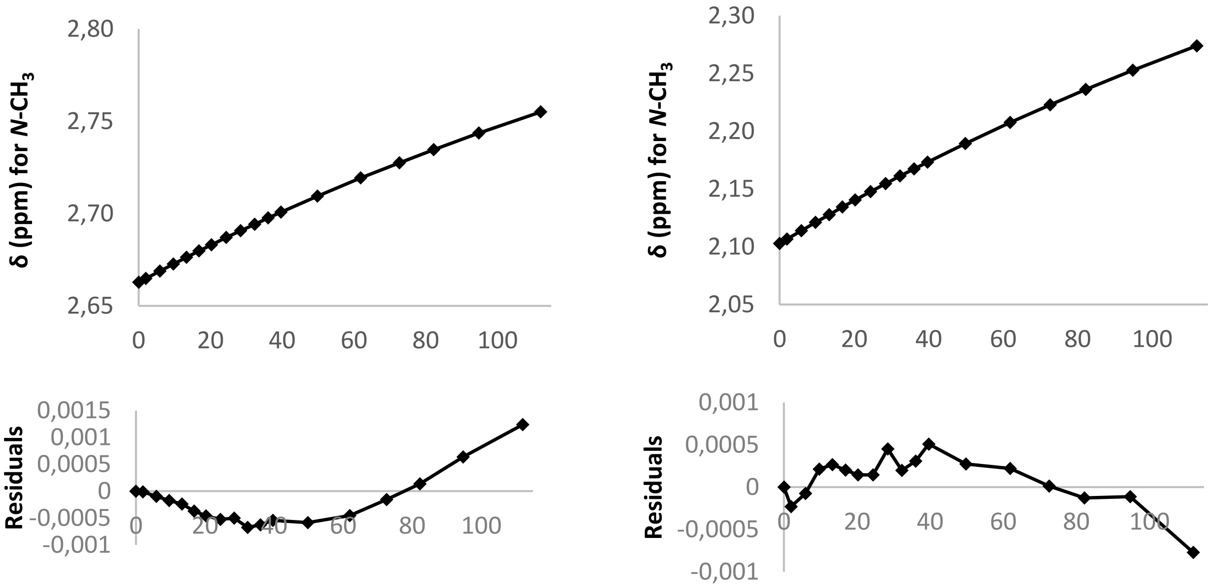

Figure S14. Calculated chemical shift values and residuals of the binding isotherm for the [**1b-11**] complex obtained with BindFit. The global fitting of the methyl protons (*N*-CH_3) gave an average value of K_{obs} = 0.480 ± 0.001 M⁻¹.

For full details see: http://app.supramolecular.org/bindfit/view/b77cd9c3-8da1-470c-9626-93a45d335a02

Trial 2

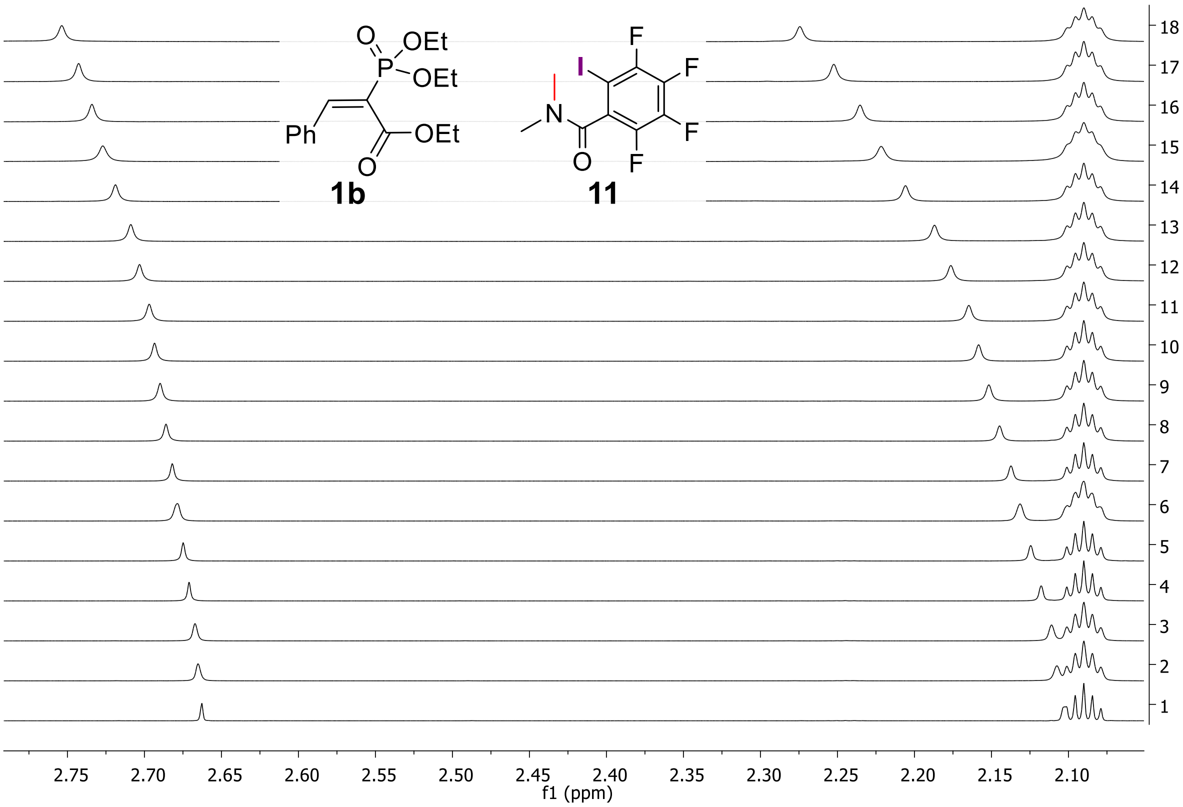

Figure S15. Outtakes of ^{1}H NMR spectra (toluene-d8, 400 MHz) of **11** (5.0 mM) titration with **1b** at 296 K. Showing the collected 18 (x2) data points of **11** methyl protons (*N*-CH_3) after reaching 0.0, 2.0, 3.9, 7.8, 11.5, 15.0, 18.5, 22.7, 26.8, 30.7, 34.5, 41.7, 48.4, 60.7, 71.5, 81.1, 93.8 and 111.2 equiv. of **1b**.

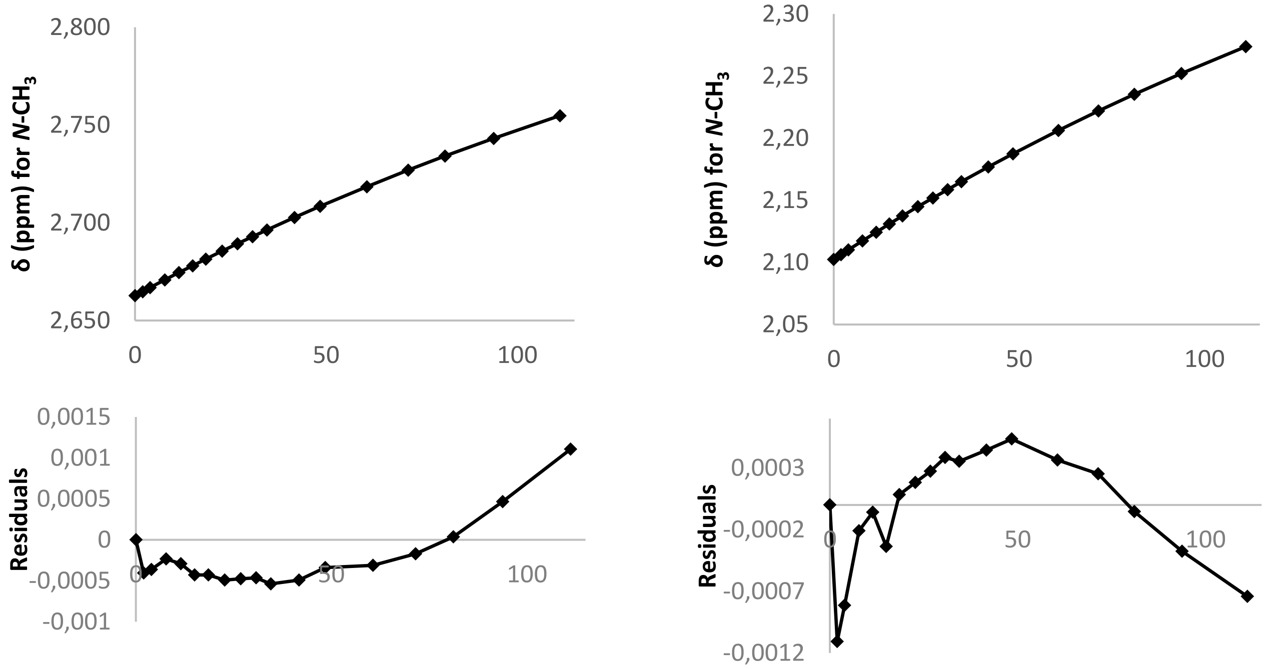

Figure S16. Calculated chemical shift values and residuals of the binding isotherm for the [**1b-11**] complex obtained with BindFit. The global fitting of the methyl protons (*N*-CH_3) gave an average value of K_{obs} = 0.488 ± 0.001 M^{-1}.

For full details see: http://app.supramolecular.org/bindfit/view/2b7d0f4a-f176-4193-b928-5c98a28103c5

Mean value of trial 1 and 2: 0.484 ± 0.004 M^{-1}

8.4 ^{1}H NMR titration data of [1b-12] complex

Trial 1

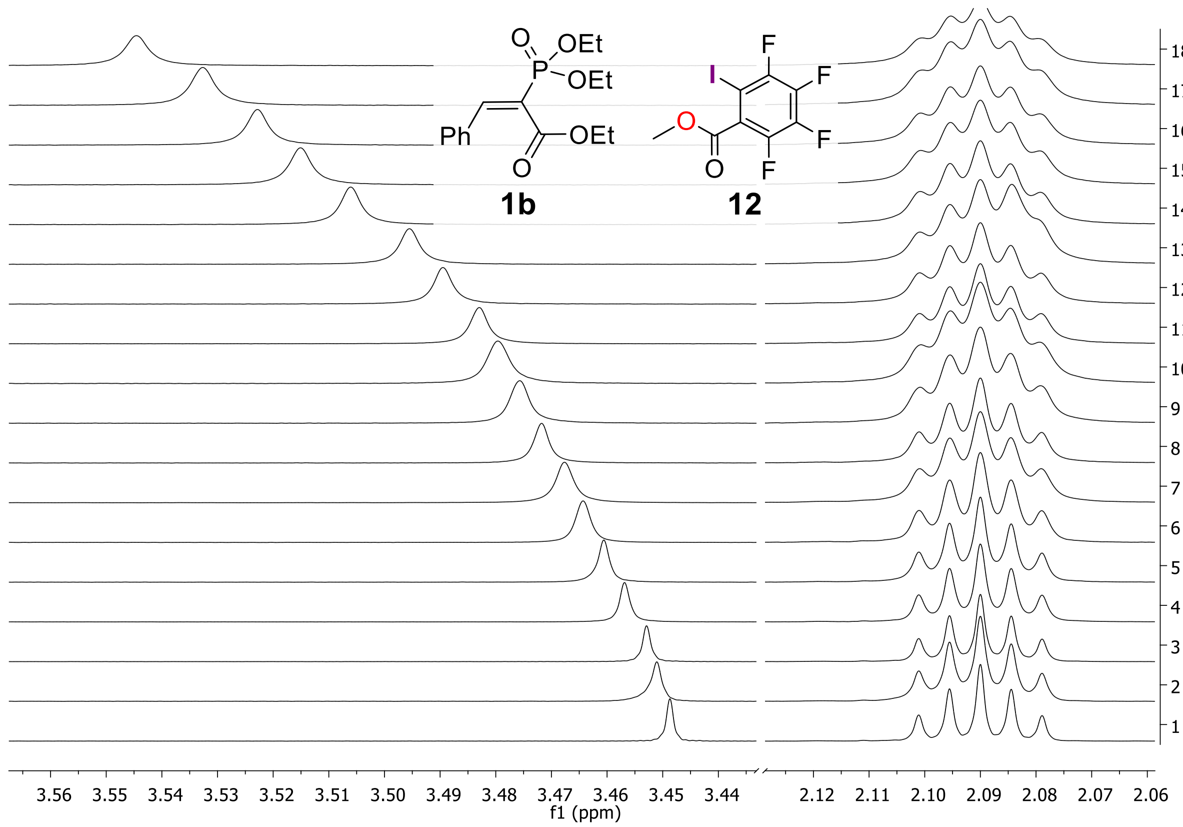

Figure S17. Outtakes of ^{1}H NMR spectra (toluene-d8, 400 MHz) of **12** (5.0 mM) titration with **1b** at 297 K. Showing the collected 18 data points of **12** methyl protons (*O*-CH_3) after reaching 0.0, 2.0, 4.0, 7.8, 11.5, 15.1, 18.6, 22.9, 26.9, 30.9, 34.7, 42.0, 48.8, 61.1, 72.1, 81.9, 94.8 and 111.2 equiv. of **1b**.

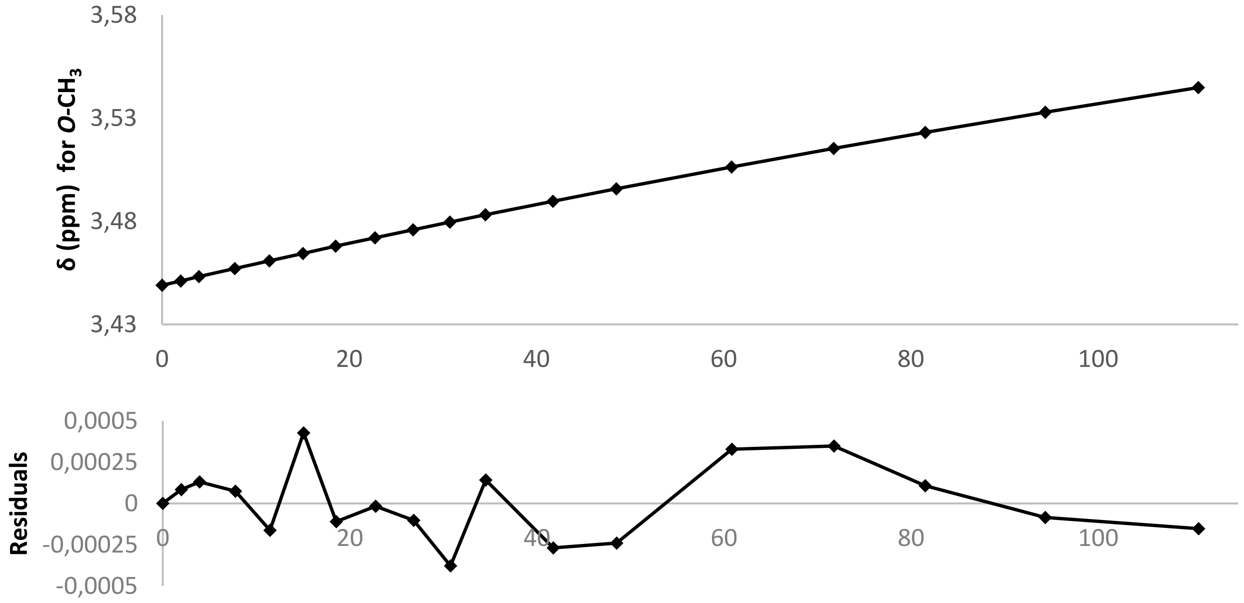

Figure S18. Calculated chemical shift values and residuals of the binding isotherm for the [**1b-12**] complex obtained with BindFit. The fitting of the methyl protons (*O*-CH_3) gave an average value of K_{obs} = 0.400 ± 0.001 M^{-1}.

For full details see: http://app.supramolecular.org/bindfit/view/bfcf1c3b-a54d-4d01-9d55-91ab734a903b

Trial 2

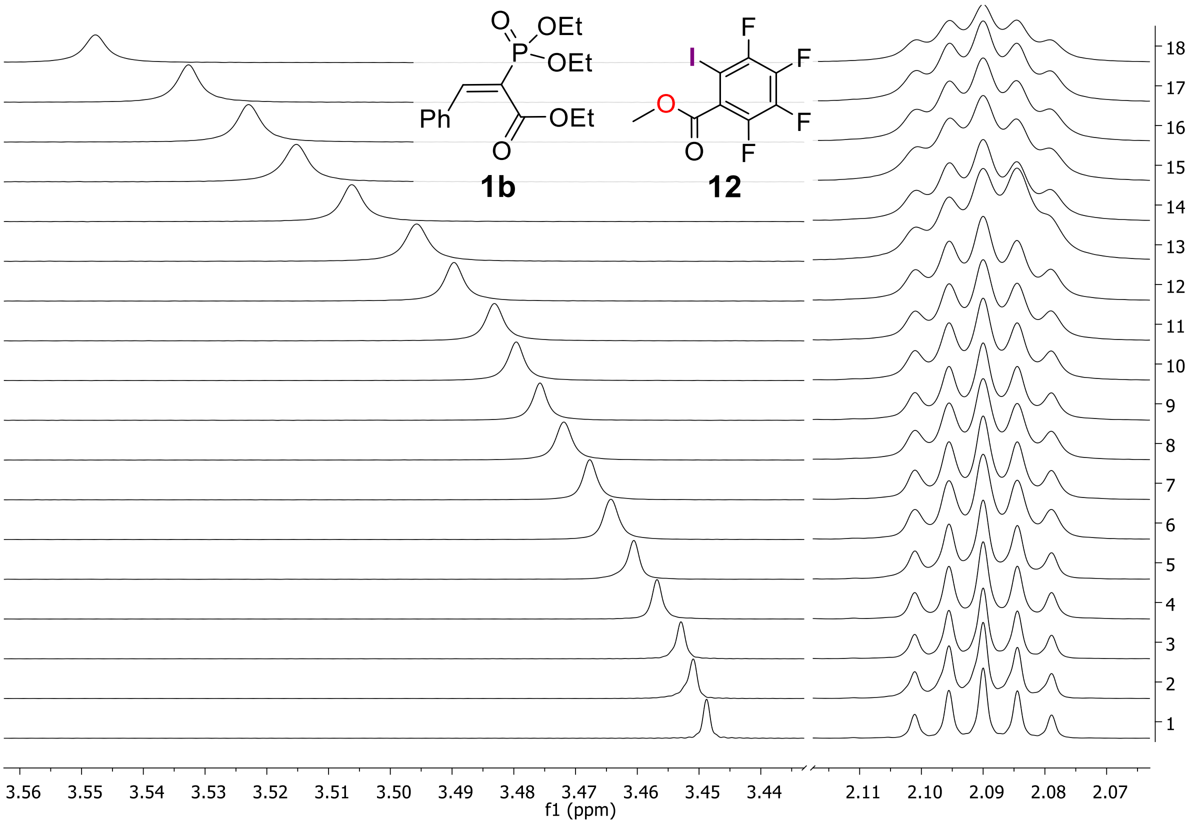

Figure S19. Outtakes of ^{1}H NMR spectra (toluene-d8, 400 MHz) of **12** (5.0 mM) titration with **1b** at 297 K. Showing the collected 18 data points of **12** methyl protons (*O*-CH_3) after reaching 0.0, 2.0, 4.0, 7.8, 11.5, 15.1, 18.6, 22.9, 26.9, 30.9, 34.7, 42.0, 48.8, 61.1, 72.1, 81.9, 94.8 and 115.6 equiv. of **1b**.

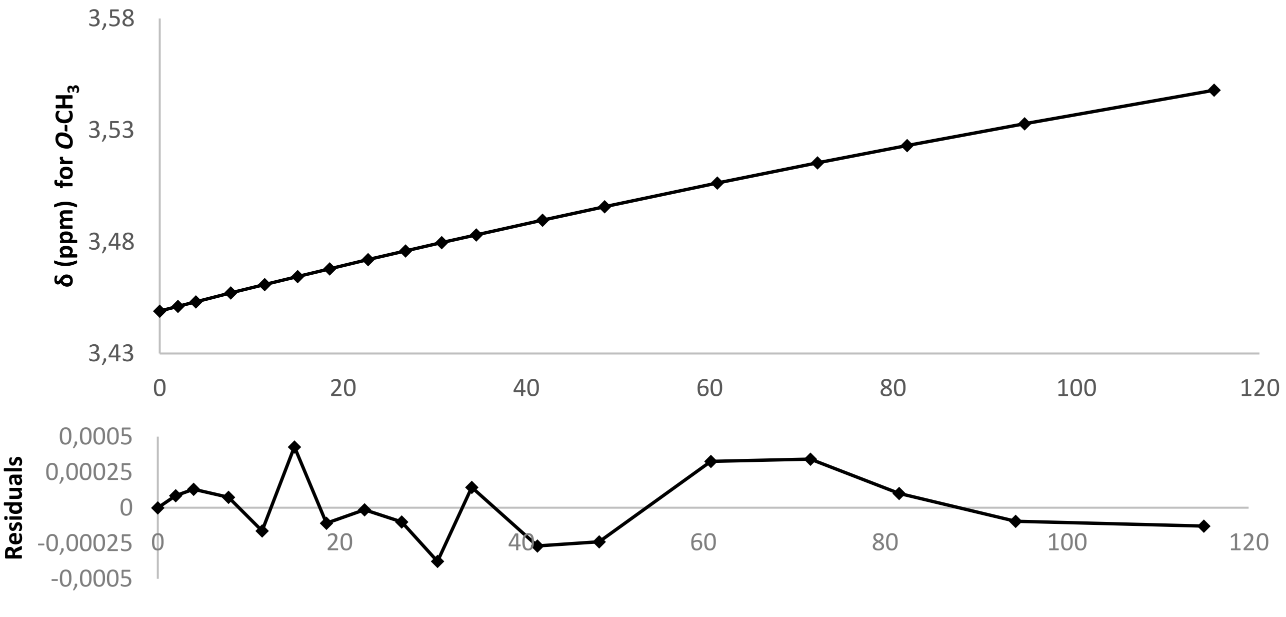

Figure S20. Calculated chemical shift values and residuals of the binding isotherm for the [**1b-12**] complex obtained with BindFit. The fitting of the methyl protons (*O*-CH_3) gave an average value of K_{obs} = 0.401 ± 0.001 M^{-1}.

For full details see: http://app.supramolecular.org/bindfit/view/515bac01-7ca9-463b-aea5-4b43e89418ed

Mean value of trial 1 and 2: 0.401 ± 0.001 M^{-1}

8.5 ^{1}H NMR titration data of [1b-13] complex

Trial 1 (concentration of **1b** was 1200 mM)

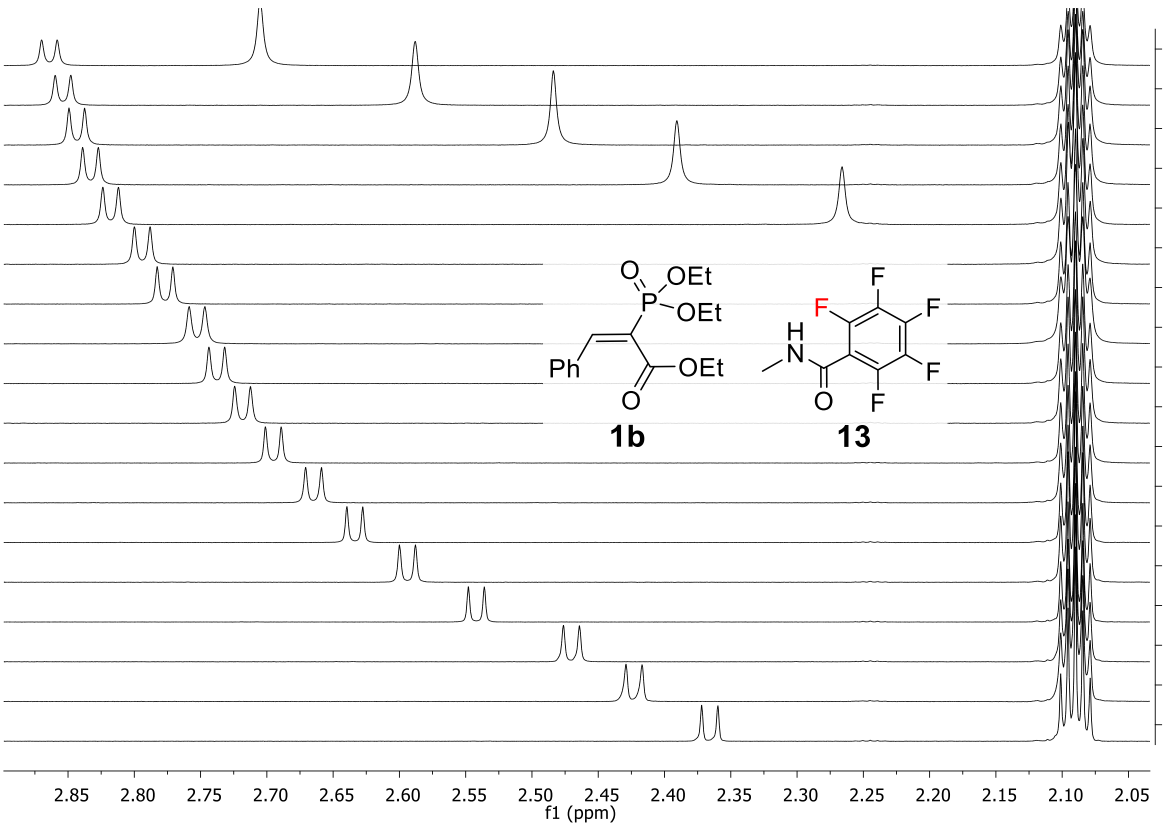

Figure S21. Outtakes of ^{1}H NMR spectra (toluene-d8, 400 MHz) of **13** (5.0 mM) titration with **1b** at 297 K. Showing the collected 18 data points of **13** methyl protons (N-CH_3) after reaching 0.0, 1.9, 3.8, 7.4, 10.9, 14.4, 17.7, 21.7, 25.6, 29.3, 32.9, 39.8, 46.2, 57.9, 68.2, 77.4, 89.5 and 106.1 equiv. of **1b**.

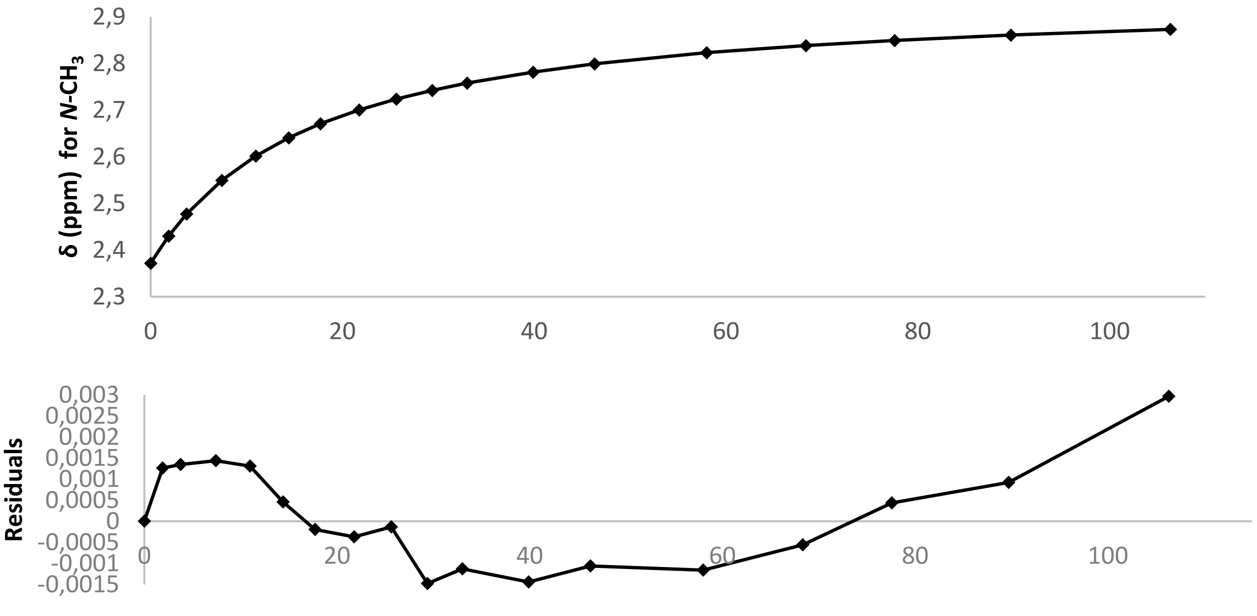

Figure S22. Calculated chemical shift values and residuals of the binding isotherm for the [**1b-13**] complex obtained with BindFit. The fitting of the methyl protons (N-CH_3) gave an average value of K_{obs} = 12.49 ± 0.07 M^{-1}.

For full details see: http://app.supramolecular.org/bindfit/view/892dd596-8b81-4357-b8fb-f2bf543915cd

Trial 2 (concentration of **1b** was 1200 mM)

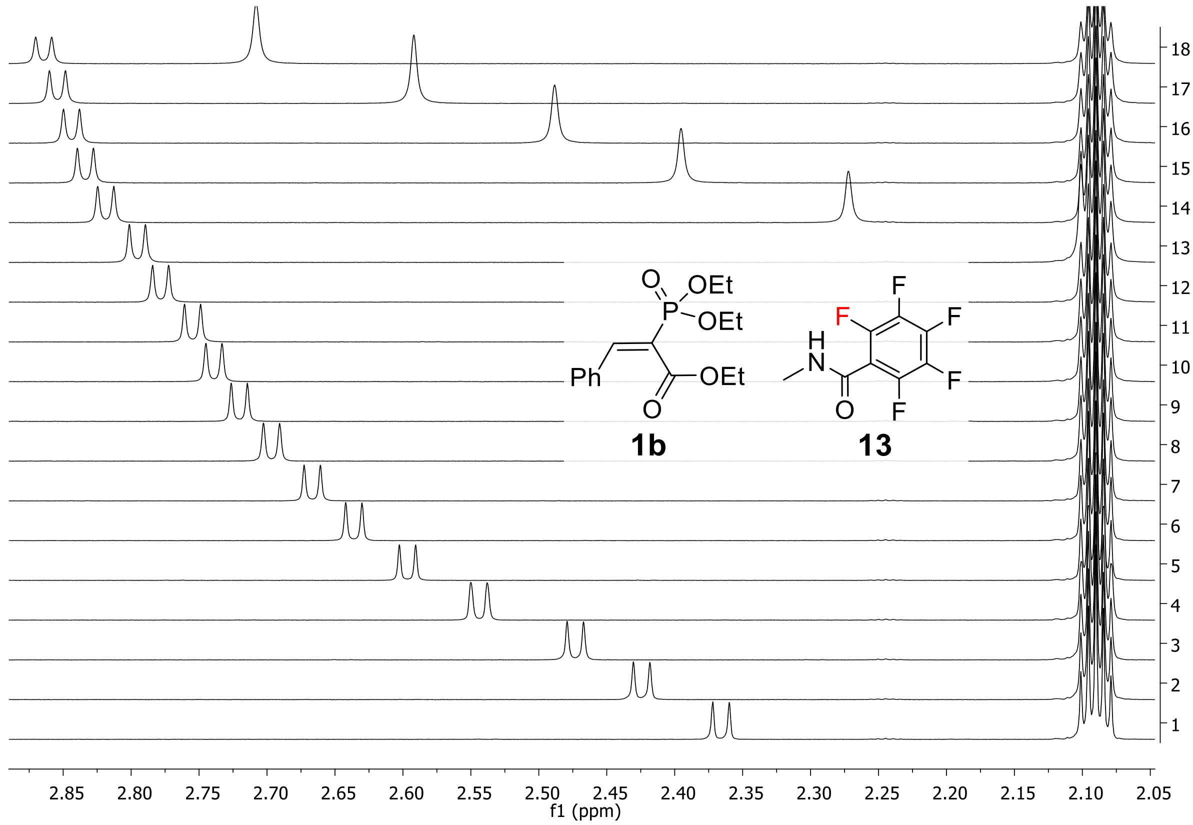

Figure S23. Outtakes of ^{1}H NMR spectra (toluene-d8, 400 MHz) of **13** (5.0 mM) titration with **1b** at 297 K. Showing the collected 18 data points of **13** methyl protons (*N*-CH_3) after reaching 0.0, 1.9, 3.8, 7.4, 10.9, 14.4, 17.7, 21.7, 25.6, 29.3, 32.9, 39.8, 46.2, 57.9, 68.1, 77.3, 89.4 and 106.0 equiv. of **1b**.

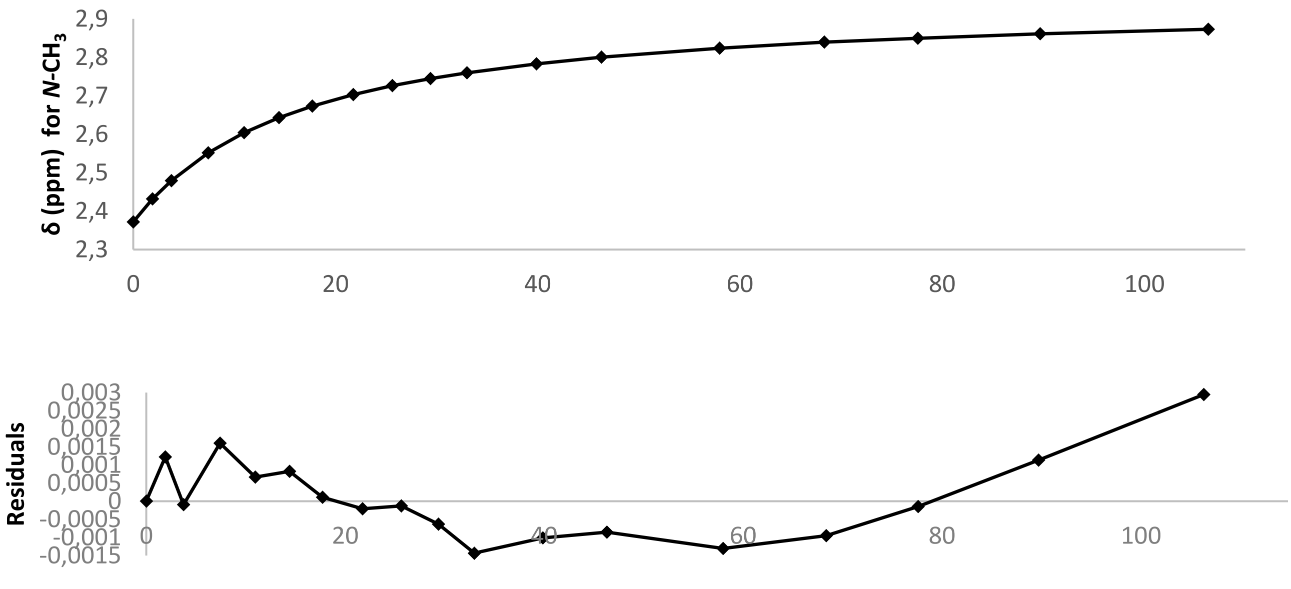

Figure S24. Calculated chemical shift values and residuals of the binding isotherm for the [**1b**-**13**] complex obtained with BindFit. The fitting of the methyl protons (*N*-CH_3) gave an average value of K_{obs} = 12.77 ± 0.07 M^{-1}.

For full details see: http://app.supramolecular.org/bindfit/view/859fcb60-d745-41fc-9ab9-a5fa6db87c93

Mean value of trial 1 and 2: 12.6 ± 0.2 M^{-1}

8.6 ^{19}F NMR titration data of [1b-B] complex

Trial 1

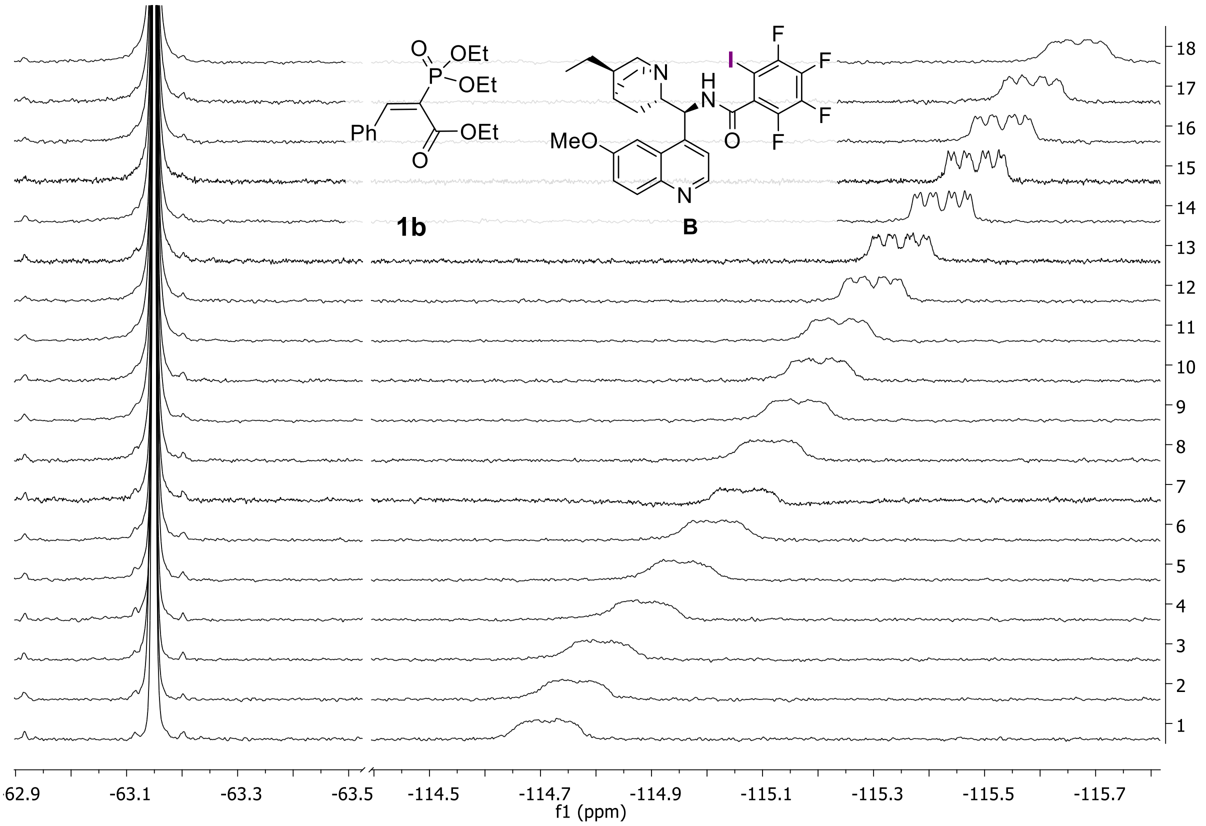

Figure S25. Outtakes of ^{19}F NMR spectra (toluene-d8, 376 MHz) of **B** (5.0 mM) titration with **1b** at 298 K. Showing the collected 18 data points of **B** flourine atom *ortho* to the iodine atom after reaching 0.0, 2.1, 4.1, 8.1, 12.0, 15.7, 19.0, 23.7, 28.0, 32.1, 36.0, 43.6, 50.6, 63.5, 74.9, 85.1, 98.5 and 116.9 equiv. of **1b**.

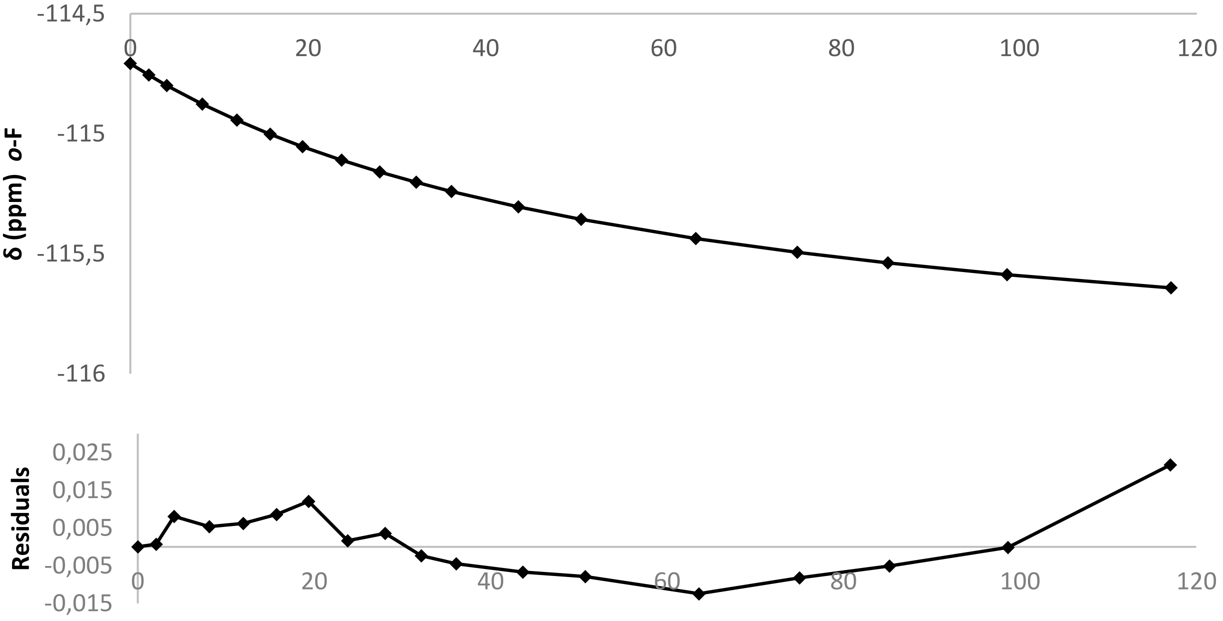

Figure S26. Calculated chemical shift values and residuals of the binding isotherm for the [**1b-B**] complex obtained with BindFit. The fitting of the ortho fluorine atom to iodine gave an average value of K_{obs} = 3.42 ± 0.06 M^{-1}.

For full details see: http://app.supramolecular.org/bindfit/view/138f0cef-7b25-4cf7-9a15-3399de37c55d

Trial 2

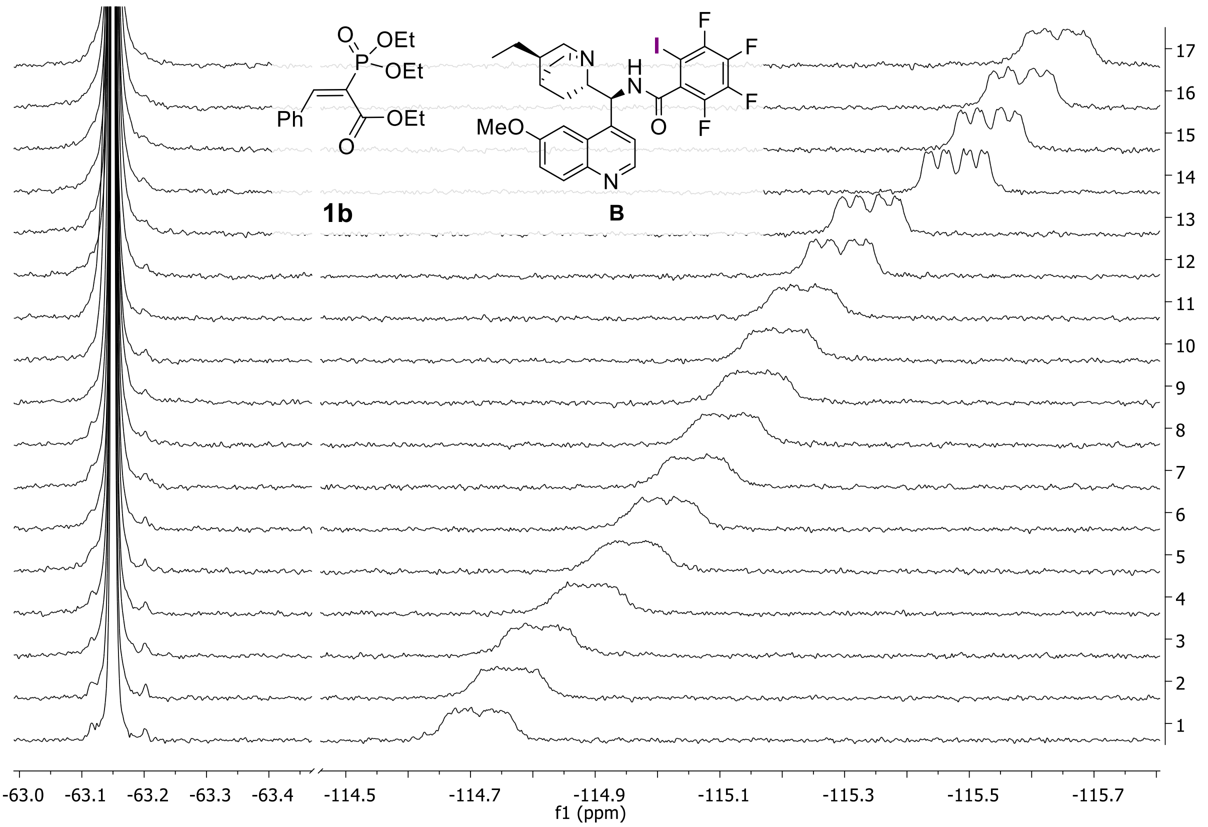

Figure S27. Outtakes of ^{19}F NMR spectra (toluene-d8, 376 MHz) of **B** (5.0 mM) titration with **1b** at 298 K. Showing the collected 17 data points of **B** flourine atom *ortho* to the iodine atom after reaching 0.0, 2.1, 4.1, 8.0, 11.8, 15.5, 19.1, 23.5, 27.7, 31.4, 35.6, 43.1, 50.1, 74.1, 84.1, 97.4 and 113.9 equiv. of **1b**.

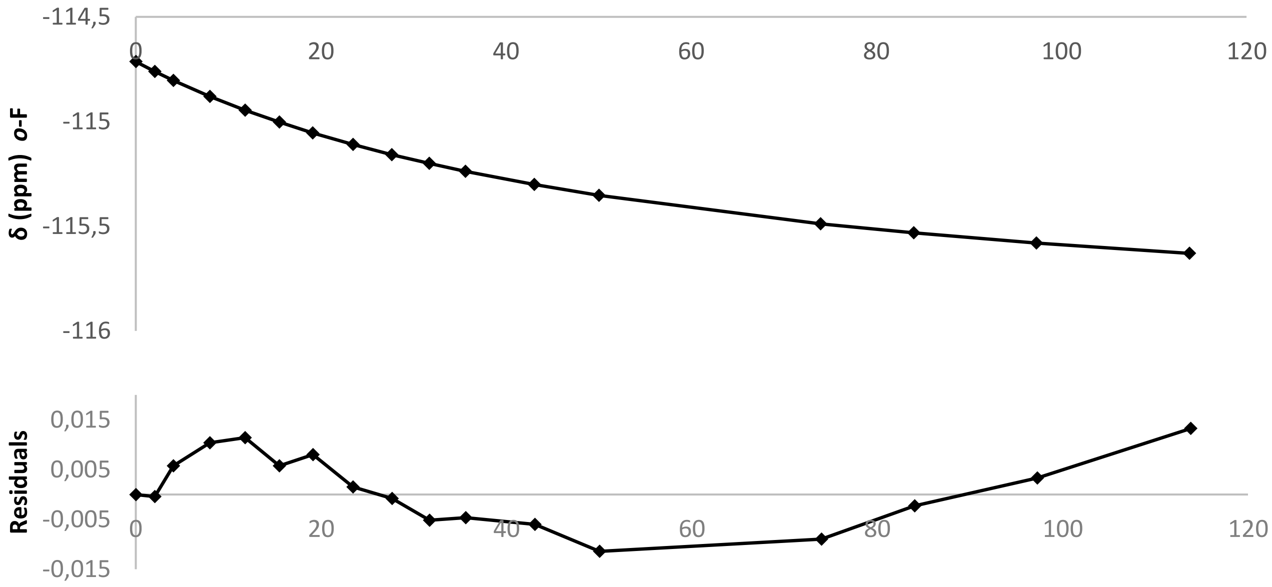

Figure S28. Calculated chemical shift values and residuals of the binding isotherm for the [**1b-B**] complex obtained with BindFit. The fitting of the ortho fluorine atom to iodine gave an average value of K_{obs} = 3.45 ± 0.05 M^{-1}.

For full details see: http://app.supramolecular.org/bindfit/view/ad7575d9-aada-46ac-9533-66d2496cab1b

Mean value of trial 1 and 2: 3.44 ± 0.06 M^{-1}

8.7 ^{1}H NMR titration data of [6-9] complex

Trial 1

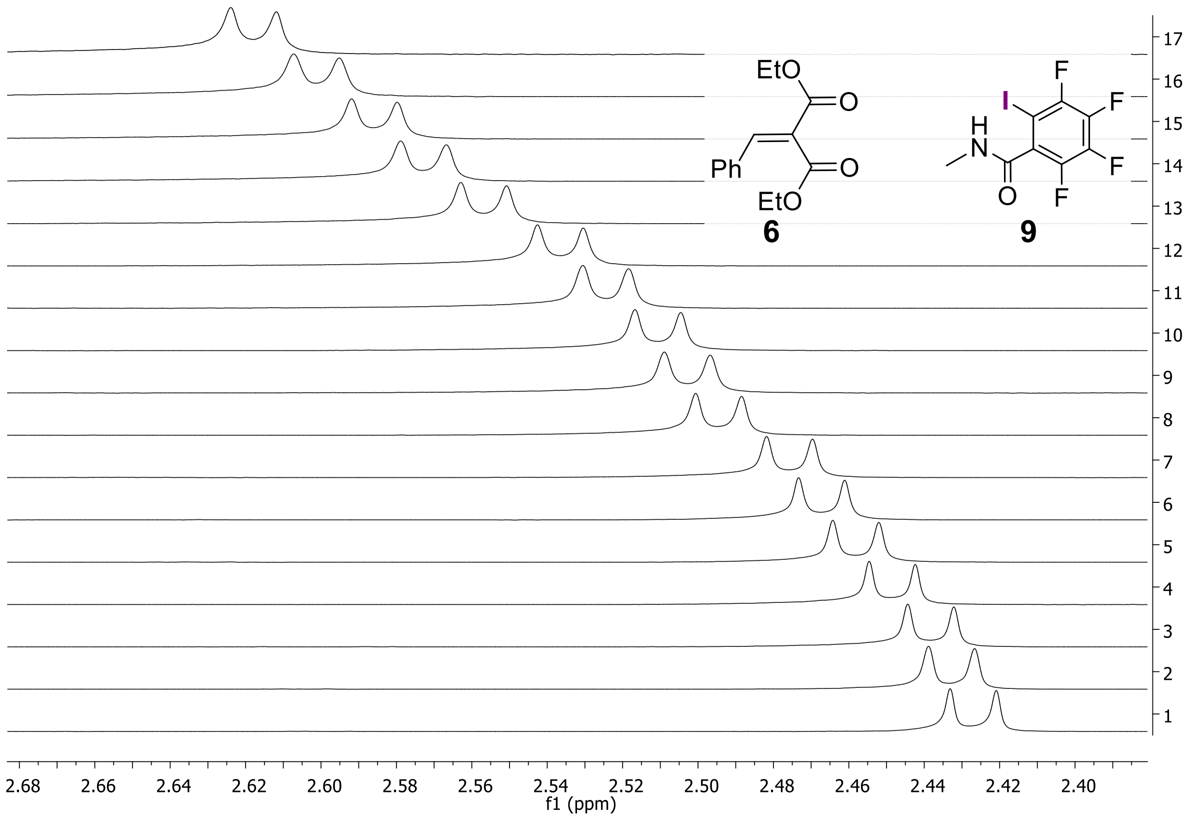

Figure S29. Outtakes of ^{1}H NMR spectra (toluene-d8, 400 MHz) of **9** (5.0 mM) titration with **6** at 296 K. Showing the collected 17 data points of **9** methyl protons (*N*-CH_3) after reaching 0.0, 2.0, 3.9, 7.7, 11.4, 15.0, 18.5, 26.8, 30.7, 34.5, 41.6, 48.3, 60.6, 71.4, 81.0, 93.7 and 109.3 equiv. of **6**.

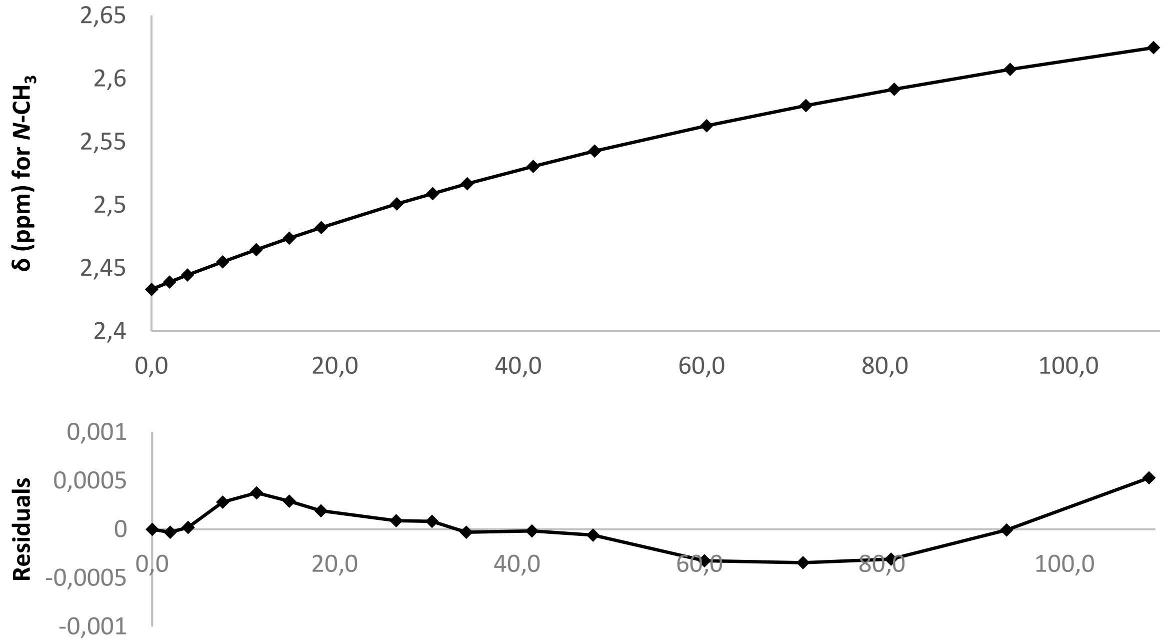

Figure S30. Calculated chemical shift values and residuals of the binding isotherm for the [**6-9**] complex obtained with BindFit. The fitting of the methyl protons (*N*-CH_3) gave an average value of K_{obs} = 1.261 ± 0.004 M^{-1}.

For full details see: http://app.supramolecular.org/bindfit/view/508d2878-ae8d-4ccb-b9bc-fea8c12b6f4e

Trial 2

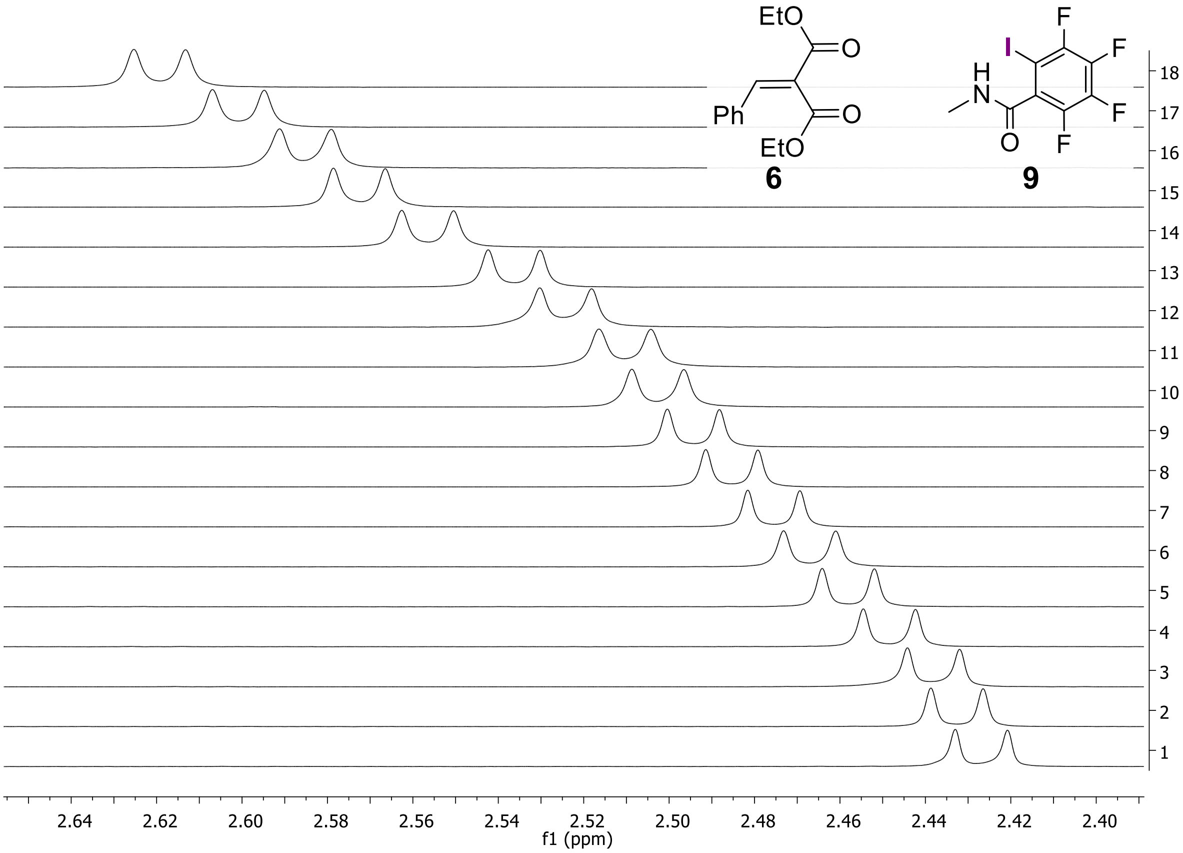

Figure S31. Outtakes of ^{1}H NMR spectra (toluene-d8, 400 MHz) of **9** (5.0 mM) titration with **6** at 296 K. Showing the collected 18 data points of **9** methyl protons (*N*-CH_3) after reaching 0.0, 2.0, 3.9, 7.7, 11.4, 15.0, 18.5, 26.8, 30.7, 34.5, 41.6, 48.3, 60.6, 71.4, 81.0, 93.7 and 111.0 equiv. of **6**.

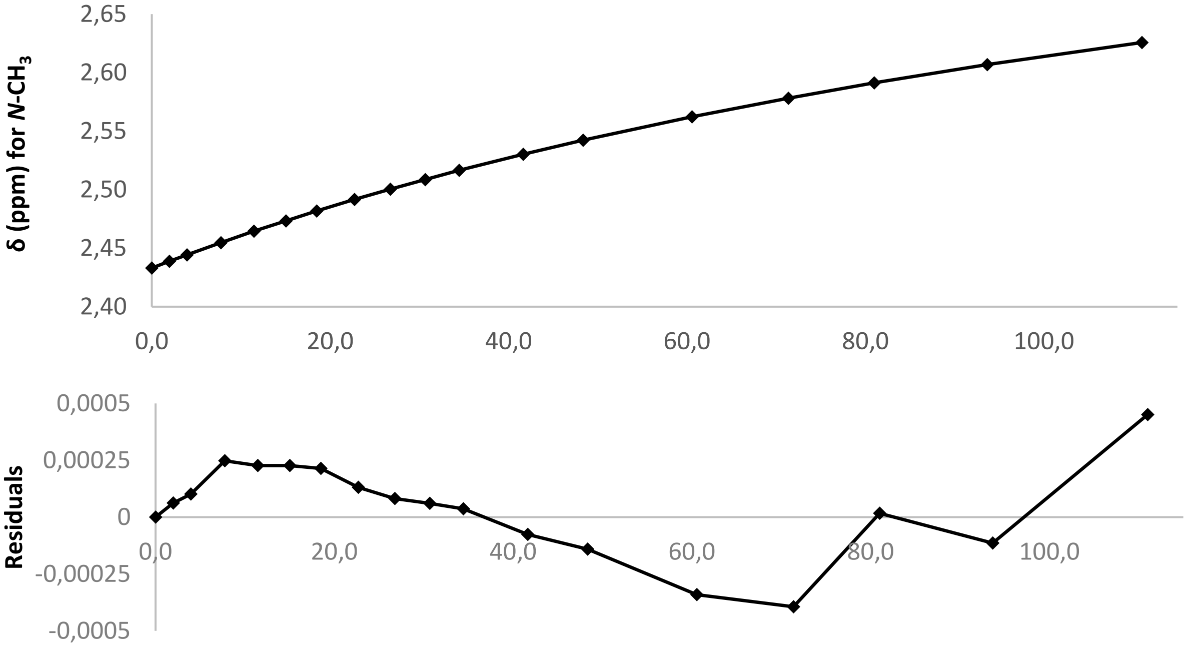

Figure S32. Calculated chemical shift values and residuals of the binding isotherm for the [**6-9**] complex obtained with BindFit. The global fitting of the methyl protons (*N*-CH_3) gave an average value of K_{obs} = 1.285 ± 0.003 M^{-1}.

For full details see: http://app.supramolecular.org/bindfit/view/28d9588a-2531-48f0-98b6-85743d338ccd

Mean value of trial 1 and 2: 1.27 ± 0.01 M^{-1}

8.8 ^{1}H NMR titration data of [8-9] complex

Trial 1

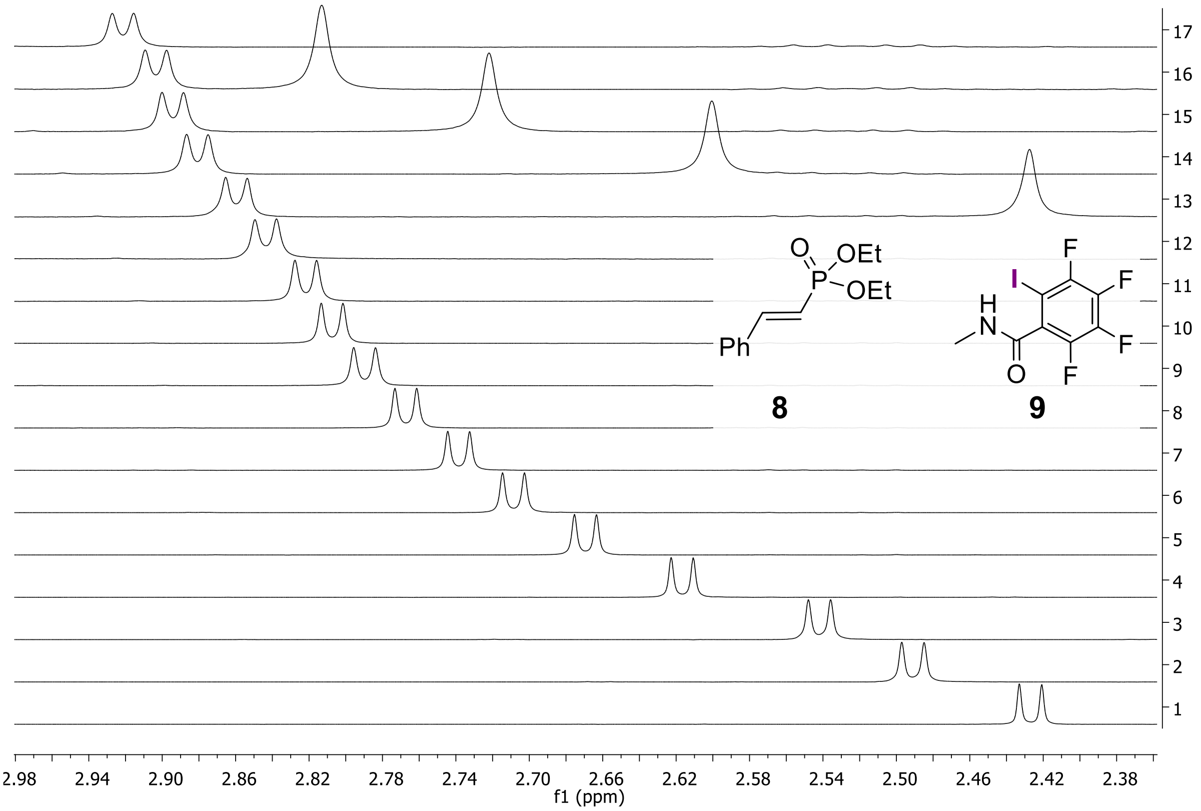

Figure S33. Outtakes of ^{1}H NMR spectra (toluene-d8, 400 MHz) of **9** (5.0 mM) titration with **8** at 296 K. Showing the collected 17 data points of **9** methyl protons (*N*-CH_3) after reaching 0.0, 2.0, 3.9, 7.8, 11.5, 15.1, 18.6, 26.8, 30.8, 34.6, 41.8, 48.5, 60.7, 71.6, 81.3 and 111.3 equiv. of **8**.

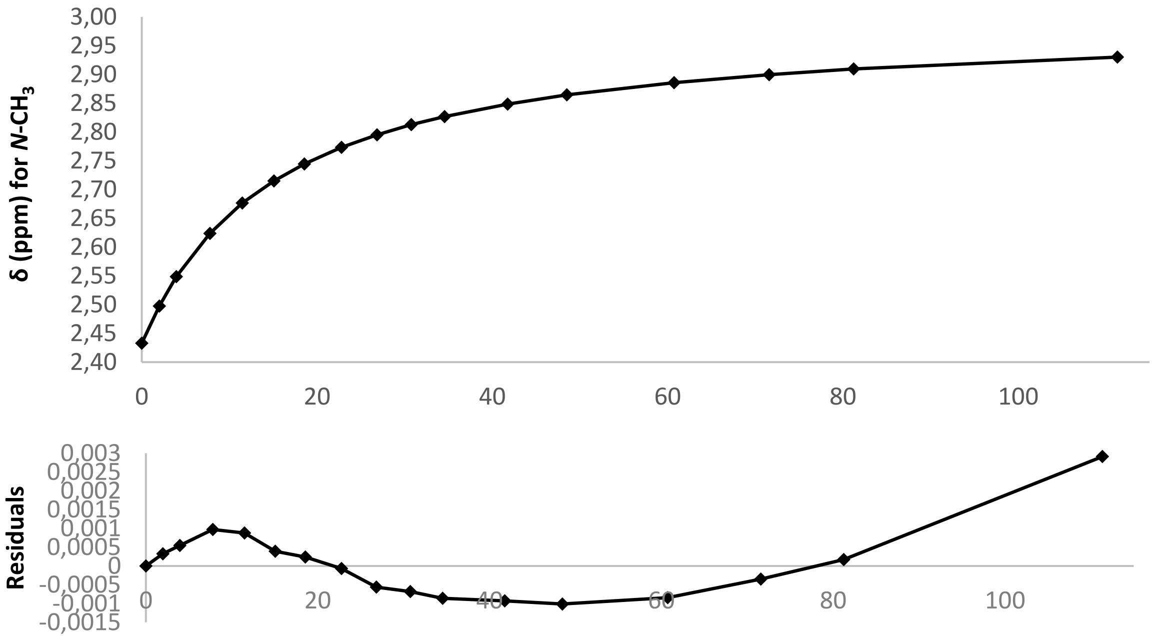

Figure S34. Calculated chemical shift values and residuals of the binding isotherm for the [**8**-**9**] complex obtained with BindFit. The global fitting of the methyl protons (*N*-CH_3) gave an average value of K_{obs} = 13.83 ± 0.08 M^{-1}.

For full details see: http://app.supramolecular.org/bindfit/view/4ba9f4f0-a752-45cd-9afe-6d4d86dec0eb

Trial 2

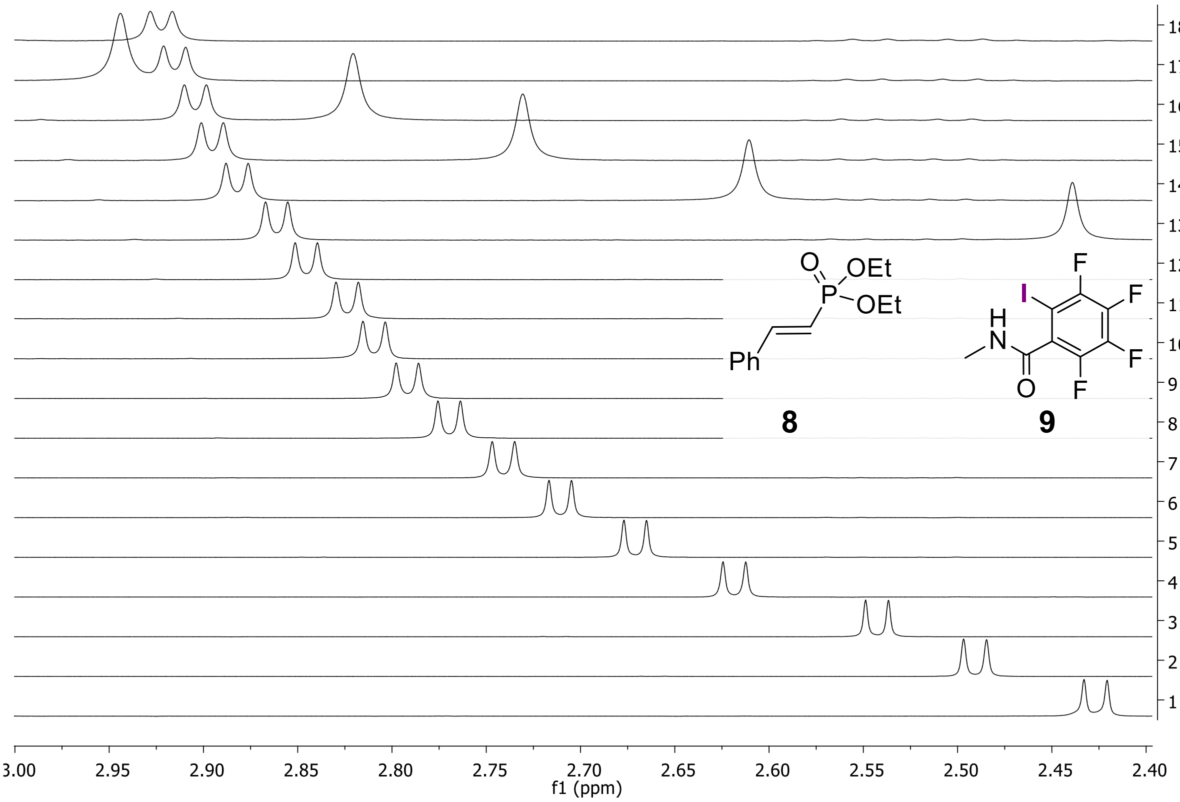

Figure S35. Outtakes of ^{1}H NMR spectra (toluene-d8, 400 MHz) of **9** (5.0 mM) titration with **8** at 296 K. Showing the collected 18 data points of **9** methyl protons (*N*-CH_3) after reaching 0.0, 2.0, 3.9, 7.8, 11.5, 15.1, 18.6, 26.8, 30.8, 34.6, 41.8, 48.5, 60.7, 71.6, 81.3, 97.8 and 112.9 equiv. of **8**.

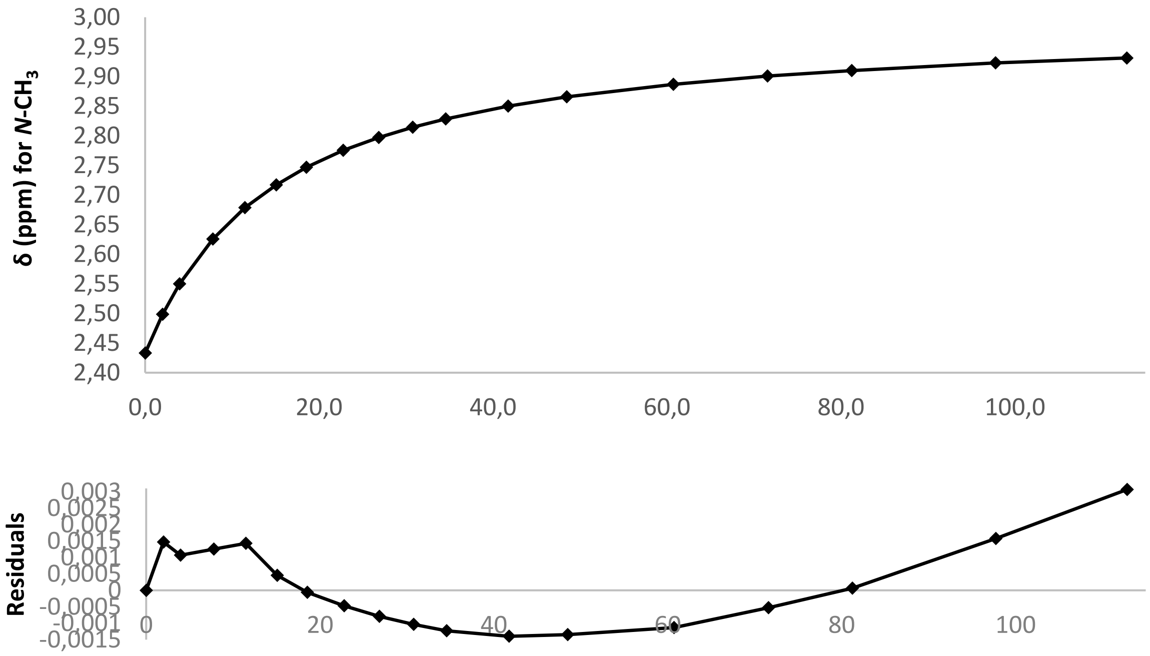

Figure S36. Calculated chemical shift values and residuals of the binding isotherm for the [**8**-**9**] complex obtained with BindFit. The global fitting of the methyl protons (*N*-CH_3) gave an average value of K_{obs} = 14.1 ± 0.1 M^{-1}.

For full details see: http://app.supramolecular.org/bindfit/view/29897c23-24ff-415f-b0d0-50e8c2a1581d

Mean value of trial 1 and 2: 14.0 ± 0.1 M^{-1}

9 Synthetic procedures for catalysts and model compounds

(1*S*)-((2*S*,4*S*,5*R*)-5-ethylquinuclidin-2-yl)(6-methoxyquinolin-4-yl)methanamine (CAS nr. 168960-95-0)

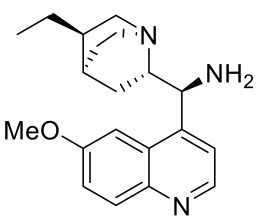

The corresponding hydrochloride salt (0.52 g, 1.40 mmol) was dissolved in DCM (20 mL), then an aqueous solution of NaOH (10 mL, 1 M) was added and the mixture was stirred for 10 minutes. The mixture was transferred to a separatory funnel with DCM (10 mL), then the phases were separated, and the aqueous phase was extracted with DCM (2 x 20 mL). The combined organic phase was dried over anhydrous K_2CO_3, concentrated under reduced pressure, and purified with column chromatography on silica gel (starting from 2% of NH_3/MeOH in DCM) to provide the desired product as a yellow resin (0.33 g, 72 %). ^{1}H NMR (400 MHz, $CDCl_3$) *δ* 8.76 (d, *J* = 4.5 Hz, 1H; Ar-H), 8.04 (d, *J* = 9.2 Hz, 1H; Ar-H), 7.66 (bs, 1H; Ar-H), 7.48 (d, *J* = 4.5 Hz, 1H; Ar-H), 7.39 (dd, *J* = 9.2, 2.7 Hz, 1H; Ar-H), 4.60 (d, *J* = 9.3 Hz, 1H; CH_2), 3.97 (s, 3H; CH_3), 3.26 (dd, *J* = 13.6, 9.8 Hz, 1H; CH_2), 3.22 – 3.16 (m, 1H; CH_2), 3.06 (dd, *J* = 17.0, 9.3 Hz, 1H; CH), 2.85 – 2.73 (m, 1H; CH_2), 2.53 (ddd, *J* = 13.6, 4.6, 2.4 Hz, 1H; CH_2), 2.02 (bs, 2H; *N*H_2), 1.64 – 1.23 (m, 7H; CH and CH_2), 0.83 (t, *J* = 7.3 Hz, 3H; CH_3), 0.79 – 0.71 (m, 1H; CH_2); ^{13}C{^{1}H} NMR (101 MHz, $CDCl_3$) *δ* 157.6, 147.9, 147.3, 144.8, 131.8, 128.8, 121.2, 120.0, 102.1, 61.8, 58.0, 55.6, 53.4, 41.1, 37.5, 28.9, 27.6, 25.8, 25.3, 12.1.

(1*S*)-quinolin-4-yl((2*S*,4*S*,5*R*)-5-vinylquinuclidin-2-yl)methanamine (CAS nr. 850409-61-9)

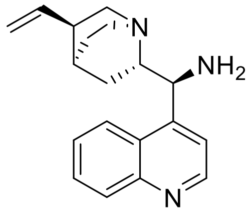

The corresponding trihydrochloride salt (0.55 g, 1,37 mmol) was dissolved in DCM (20 mL), then an aqueous solution of NaOH (10 mL, 1 M) was added and the mixture was stirred for 10 minutes. The mixture was transferred to a separatory funnel with DCM (10 mL), then the phases were separated, and the aqueous phase was extracted with DCM (2 x 10 mL). The combined organic phase was dried over anhydrous Na_2SO_4, concentrated under reduced pressure, and purified with column chromatography on silica gel (starting from 3% of NH_3/MeOH in DCM) to provide the desired product as a yellow resin (0.37 g, 92%). ^{1}H NMR (400 MHz, $CDCl_3$) *δ* 8.91 (d, *J* = 4.5 Hz, 1H; Ar-H), 8.37 (bs, 1H; Ar-H), 8.14 (dd, *J* = 8.4, 0.9 Hz, 1H; Ar-H), 7.73 (ddd, *J* = 8.3, 6.8, 1.3 Hz, 1H; Ar-H), 7.60 (ddd, *J* = 8.3, 6.8, 1.3 Hz, 1H; Ar-H), 7.53 (d, *J* = 4.5 Hz, 1H; Ar-H), 5.81 (ddd, *J* = 17.5, 10.3, 7.6 Hz, 1H; =CH), 5.04 – 4.95 (m, 2H; =CH_2), 4.72 (d, *J* = 8.6 Hz, 1H; CH), 3.28 (dd, *J* = 13.8, 10.0 Hz, 1H; CH_2), 3.25 – 3.17 (m, 1H; CH_2), 3.08 (dd, *J* = 16.4, 8.6 Hz, 1H; CH), 2.89 – 2.76 (m, 1H; CH_2), 2.35 – 2.25 (m, 1H; CH), 2.01 (s, 2H, *N*H_2) 1.66 – 1.60 (m, 1H; CH_2), 1.60 – 1.50 (m, 2H; CH and CH_2), 1.47 – 1.37 (m, 1H; CH_2), 0.80 – 0.70 (m, 1H; CH_2); ^{13}C{^{1}H} NMR (101 MHz, $CDCl_3$) *δ* 150.4, 148.8, 148.6, 141.8, 130.5, 129.0, 127.9, 126.5, 114.3, 62.0, 56.3, 53.4, 41.0, 39.9, 28.1, 27.6, 26.1. (signals corresponding to the carbon atoms 3' and 5' in the quinoline core could not be detected due to the low intensity of the signals).

(1*R*)-((2*S*,4*S*,5*R*)-5-ethylquinuclidin-2-yl)(6-methoxyquinolin-4-yl)methyl methanesulfonate

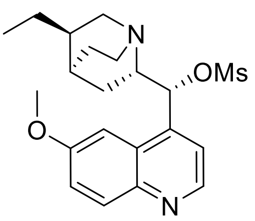

Based on minor modifications to the literature procedure.[1] Under argon atmosphere, dihydroquinine (1.00 g, 3.06 mmol) and triethylamine (1.70 mL, 12.3 mmol) were dissolved in THF (4.5 mL) and the reaction mixture was cooled to 0 °C. Mesyl chloride (0.60 mL, 7.75 mmol) in THF (2.0 mL) was added dropwise and then the mixture was stirred for at RT for 2 h. The mixture was cooled to 0 °C and quenched with the addition of a saturated aqueous solution of $NaHCO_3$ (3.5 mL). The mixture was transferred to a separatory funnel with EtOAc (10 mL), then the phases were separated, and the aqueous phase was extracted with EtOAc (4 x 10 mL). The combined organic phase was dried over anhydrous Na_2SO_4, concentrated under reduced pressure, and purified with column chromatography on silica gel (starting from 5% MeOH in EtOAc:NH_3/MeO 500:5) to provide the desired product as a yellow resin that solidifies in the fridge (1.05 g, 85%). $[\alpha]^D_{20}$=-59.5 (*c*=0.23 in $CHCl_3$); IR (film): $\tilde{\nu}$=2932 (s), 2868 (s), 1736 (m), 1621 (s), 1509 (s), 1358 (s), 1172 (s), 1030 (m), 940 (m) cm^{-1}; ^{1}H NMR (400 MHz, $CDCl_3$) δ 8.81 (d, *J* = 4.4 Hz, 1H, Ar-H), 8.06 (d, *J* = 9.2 Hz, 1H, Ar-H), 7.47 (bs, 1H, Ar-H), 7.42 (dd, *J* = 9.2, 2.6 Hz, 1H, Ar-H), 7.39 (bs, 1H, Ar-H), 6.24 (s, 1H, CH), 3.98 (s, 3H, CH_3), 3.39 (bs, 1H, CH), 3.14 (bs, 1H, CH_2), 3.06 – 2.94 (m, 1H, CH_2), 2.71 – 2.66 (m, 1H, CH_2), 2.63 (s, 3H, CH_3), 2.29 (d, *J* = 11.4 Hz, 1H, CH_2), 2.01 – 1.93 (m, 1H, CH_2), 1.88 (bs, 1H, CH), 1.83 – 1.74 (m, 1H, CH_2), 1.72 – 1.60 (m, 1H, CH_2), 1.56 – 1.41 (m, 2H, CH, CH_2), 1.42 – 1.23 (m, 2H, CH_2), 0.86 (t, *J* = 7.3 Hz, 3H, CH_3); ^{13}C{^{1}H} NMR (101 MHz, $CDCl_3$) δ 158.5, 147.5, 145.0, 141.5, 132.2, 126.5, 122.3, 119.2, 100.8, 77.3, 59.7, 58.2, 55.8, 42.3, 39.2, 37.3, 28.1, 27.7, 25.0, 24.5, 12.1; HRMS (ESI): *m/z* calcd for $C_{21}H_{29}N_2O_4S^+$: 405.1843 [*M*+H]$^+$; found: 405.1848.

(1*S*)-((2*S*,4*S*,5*R*)-5-ethylquinuclidin-2-yl)(6-methoxyquinolin-4-yl)methanol

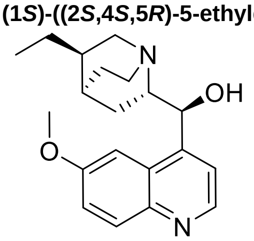

Mesyl dihydroquinine (0.30 g, 0.74 mmol) and L-tartaric acid (0.12 g, 0.81 mmol) were dissolved in water (3 mL) and then heated at 100 °C for 30 minutes. After cooling to RT, a saturated aqueous solution of $NaHCO_3$ (6 mL) was added. The mixture was transferred to a separatory funnel with DCM (10 mL), then the phases were separated, and the aqueous phase was extracted with DCM (2 x 10 mL). The combined organic phase was dried over anhydrous Na_2SO_4, concentrated under reduced pressure, and purified with column chromatography on silica gel (EtOAc/MeOH/NH_3:MeOH 100:5:2) to provide the desired product as a pale-yellow resin (0.18 g, 75%). $[\alpha]^D_{20}$=8.7 (*c*=0.10 in $CHCl_3$); IR (film): $\tilde{\nu}$=2931 (s), 2866 (m), 1621 (s), 1590 (w), 1508 (s), 1240 (s), 1032 (m); ^{1}H NMR (400 MHz, $CDCl_3$) *δ* 8.75 (d, *J* = 4.5 Hz, 1H, Ar-H), 8.03 (d, *J* = 9.2 Hz, 1H, Ar-H), 7.66 (d, *J* = 2.7 Hz, 1H, Ar-H), 7.42 (d, *J* = 4.5 Hz, 1H, Ar-H), 7.38 (dd, *J* = 9.2, 2.7 Hz, 1H, Ar-H), 5.02 (d, *J* = 9.6 Hz, 1H, CH), 3.95 (s, 3H, CH_3), 3.24 (dd, *J* = 13.6, 9.9 Hz, 1H, CH_2), 3.22 – 3.14 (m, 1H, CH_2), 3.08 (dd, *J* = 18.2, 9.6 Hz, 1H, CH), 2.83 – 2.72 (m, 1H, CH_2), 2.49 (ddd, *J* = 13.6, 4.1, 2.6 Hz, 1H, CH_2), 1.73 – 1.65 (m, 1H, CH), 1.66 – 1.44 (m, 3H, CH, CH_2), 1.44 – 1.35 (m, 1H), 1.33 – 1.21 (m, 2H, CH_2), 1.00 – 0.91 (m, 1H, CH_2), 0.81 (t, *J* = 7.4 Hz, 3H, CH_3); ^{13}C{^{1}H} NMR (101 MHz, $CDCl_3$) *δ* 157.4, 147.6,

144.9, 144.6, 131.7, 128.2, 121.3, 120.2, 102.7, 71.4, 61.5, 57.6, 55.5, 40.9, 37.7, 28.7, 27.7, 24.9, 24.9, 12.1; HRMS (ESI): *m/z* calcd for $C_{20}H_{27}N_2O_2^+$: 327.2067 [*M*+H]$^+$; found: 327.2071.

methyl ((1*S*)-((2*S*,4*S*,5*R*)-5-ethylquinuclidin-2-yl)(6-methoxyquinolin-4-yl)methyl)carbamate

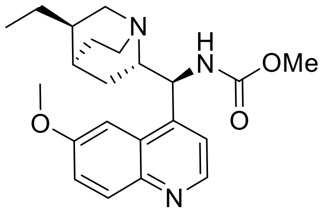

To a solution of the (1*S*)-((2*S*,4*S*,5*R*)-5-ethylquinuclidin-2-yl)(6-methoxyquinolin-4-yl)methanamine (0.32 g, 0.99 mmol) in DCM (10 mL) was added triethylamine (0.140 mL, 0.99 mmol) and ClCOOMe (76 µL, 0.99 mmol) under argon atmosphere. The mixture was stirred at RT for 18 h, then water (15 mL) was added. The mixture was extracted with DCM (3 x 10 mL) and the organic phase was dried over Na_2SO_4. After filtration, the residue was purified with column chromatography on silica gel (starting from 1% NH_3/MeOH in DCM), affording compound methyl ((1*S*)-((2*S*,4*S*,5*R*)-5-ethylquinuclidin-2-yl)(6-methoxyquinolin-4-yl)methyl)carbamate as a white foam (0.229 mg, 75%).[α]$^D_{20}$=-37.6 (*c*=0.13 in $CHCl_3$); m.p. 72–79°C; IR (film): $\tilde{\nu}$=2930 (s), 2861 (m), 1714 (s), 1622 (s), 1508 (s), 1242 (s), 1032 (m); ^{1}H NMR (400 MHz, $CDCl_3$) δ 8.74 (d, J = 4.6 Hz, 1H, Ar-H), 8.03 (d, J = 9.2 Hz, 1H, Ar-H), 7.66 – 7.57 (m, 1H, Ar-H), 7.43 – 7.35 (m, 2H, Ar-H), 6.18 (bs, 1H), 5.06 (bs, 1H), 3.97 (s, 3H, CH_3), 3.55 (bs, 3H, CH_3), 3.22 (dd, J = 13.7, 9.9 Hz, 1H, CH_2), 3.18 – 3.04 (m, 1H, CH_2), 3.04 – 2.87 (m, 1H, CH), 2.79 – 2.65 (m, 1H, CH_2), 2.39 (ddd, J = 13.8, 5.0, 2.5 Hz, 1H, CH_2), 1.66 – 1.55 (m, 2H, CH, CH_2), 1.56 – 1.47 (m, 1H, CH_2), 1.46 – 1.37 (m, 1H, CH), 1.36 – 1.13 (m, 2H for Et CH_2, 1H for CH_2), 0.90 (dd, J = 13.6, 7.0 Hz, 1H, CH_2), 0.79 (t, J = 7.3 Hz, 3H, CH_3); ^{13}C{^{1}H} NMR (101 MHz, $CDCl_3$) δ 157.8, 157.0, 147.8, 144.9, 132.0, 121.6, 101.9, 77.5, 77.2, 76.8, 57.8, 55.7, 52.2, 41.1, 37.4, 28.8, 27.6, 25.9, 25.2, 12.1; HRMS (ESI): *m/z* calcd for $C_{22}H_{30}N_3O_3^+$: 384.2282 [*M*+H]$^+$; found: 384.2291.

(1*S*)-1-((2*S*,4*S*,5*R*)-5-ethylquinuclidin-2-yl)-1-(6-methoxyquinolin-4-yl)-N-methylmethanamine

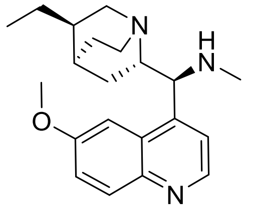

To a suspension of $LiAlH_4$ (0.10 g, 2.66 mmol) in THF (2.7 mL) was dropwise added compound methyl ((1*S*)-((2*S*,4*S*,5*R*)-5-ethylquinuclidin-2-yl)(6-methoxyquinolin-4-yl)methyl)carbamate (0.260 mg, 0.66 mmol) in THF (4 mL) under argon atmosphere at 0 °C. The mixture was refluxed for 8 h and then cooled to 0 °C. An aqueous KOH (20% w/w, 0.500 mL) was added dropwise and the obtained mixture was filtered through a pad of silica gel. The solvent was evaporated and the residue was purified with column chromatography on silica gel (starting from 1% NH_3/MeOH in DCM), affording compound the desired product as a yellow oil (0.12 g, 54%). [α]$^D_{20}$=73.8 (*c*=0.09 in $CHCl_3$); IR (film): $\tilde{\nu}$=2935 (s), 2862 (m), 1714 (s), 1620 (s), 1508 (s), 1229 (s), 1034 (m), 793 (s); ^{1}H NMR (400 MHz, $CDCl_3$) δ 8.93 – 8.46 (m, 1H, Ar-H), 8.16 – 7.93 (m, 1H, Ar-H), 7.76 – 7.59 (m, 1H, Ar-H), 7.61 – 7.46 (m, 1H, Ar-H), 7.47 – 7.32 (m, 1H, Ar-H), 4.33 (s, 1H, CH), 3.95 (s, 3H, CH_3), 3.30 – 3.01 (m, 2H, CH_2), 3.01 – 2.82 (m, 1H, CH), 2.83 – 2.63 (m, 1H, CH_2), 2.65 – 2.37 (m, 1H, CH_2), 2.19 (s, 3H, CH_3), 1.68 – 1.52 (m, 2H, CH, CH_2), 1.52 – 1.44 (m, 1H, CH_2), 1.43 – 1.33 (m, 1H, CH), 1.33 – 1.02 (m, 2H, CH_2), 0.87 – 0.67 (m, 5H, CH_2, CH_3); ^{13}C{^{1}H} NMR (101 MHz, $CDCl_3$) δ 157.6, 148.4, 132.0, 121.0, 120.1, 101.5, 62.3, 60.1, 57.9, 55.7, 53.5, 41.4, 37.7, 34.6, 29.1, 27.7, 25.3, 12.2; ^{1}H NMR (400 MHz, MeOD) δ 8.70 (s, 1H, Ar-H), 7.98 (d, *J* = 9.1 Hz, 1H, Ar-H), 7.68 (s, 1H, Ar-H), 7.84 – 7.26 (m, 1H, Ar-H), 7.47 (d, *J* = 7.7 Hz, 1H, Ar-H), 4.67 – 4.43 (m, 1H, CH), 4.00 (s, 3H, CH_3), 3.36 – 3.31 (m, 1H, CH_2), 3.25 (dd, *J* = 13.6, 9.9 Hz, 1H, CH_2), 3.12 – 2.98 (m, 1H, CH), 2.84 – 2.71 (m, 1H, CH_2), 2.54 – 2.47 (m, 1H, CH_2), 2.17 (s, 3H, CH_3), 1.71 – 1.61 (m, 1H, CH_2), 1.59 – 1.53 (m, 2H, CH, CH_2), 1.51 – 1.43 (m, 1H, CH), 1.36 – 1.17 (m, 3H, CH_2), 0.82 (t, *J* = 7.3 Hz, 3H, CH_3), 0.80 – 0.72 (m, 1H, CH_2); ^{13}C{^{1}H} NMR (101 MHz, MeOD) δ 148.5, 131.6, 123.3, 121.2, 102.6, 62.9, 60.6, 58.7, 56.3, 42.0, 38.7, 33.9, 29.6, 28.6, 26.5, 26.2, 12.4 (the quaternary carbon atoms of the quinoline core was not detected due to the low intensity of the signals); HRMS (ESI): *m/z* calcd for $C_{21}H_{30}N_3O^+$: 340.2383 [*M*+H]$^+$; found: 340.2387.

2,3,4,5-tetrafluoro-6-iodobenzoic acid (CAS nr. 110625-15-5)

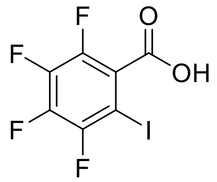

Synthesised according to the literature procedure on a 12.9 mmol scale and obtained as colourless crystals (3.20 g, 78%).[20] ^{13}C{^{1}H} NMR (400 MHz, [D$_6$]DMSO) δ 164.0, 148.6 – 145.9 (m), 145.1 – 142.2 (m), 141.2 – 138.5 (m), 140.9 – 138.1 (m), 125.7 – 125.3 (m), 77.2 – 76.6 (m); ^{19}F NMR (376 MHz, [D$_6$]DMSO) δ -115.5 (dd, *J* = 23.2, 8.8 Hz; 1F), -140.1 (dd, *J* = 20.6, 9.1 Hz; 1F), -152.0 (t, *J* = 22.8 Hz; 1F), -153.8 (t, *J* = 21.8 Hz; 1F).

5-iodo-2-nitrobenzoic acid (CAS nr. 35674-28-3)

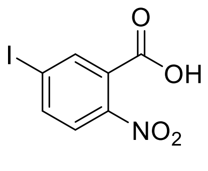

The nitro-acid (1.10 g, 5.92 mmol) was suspended in an aqueous solution of sulfuric acid (10 mL, 1.5 M), then stirred for 1 h and cooled to 0 °C. Then an aqueous solution of $NaNO_2$ (15 mL, 0.56 m) was added dropwise (caution: an exothermic reaction was observed) and the resulting dark mixture was stirred at 0 °C for 30 minutes and at RT for 30 minutes. Then the solid particles were removed by filtration and washed with water (30 mL). The combined yellow solution was cooled to 0 °C and an aqueous solution of KI (6 mL, 3.0 M) was added dropwise, which resulted in a vigorous reaction and a colour change to brown-red. After 10 minutes, the temperature of the reaction mixture was raised to RT and stirred for 2 h. The reaction mixture was transferred to a separatory funnel with EtOAc (80 mL), diluted with a saturated solution of $NaHSO_3$ (30 mL), the phases were separated, and the aqueous phase was extracted with EtOAc (80 mL). The combined organic phase was concentrated under reduced pressure. The resulting solid was dissolved in water (20 mL) and basified with an aqueous solution of NaOH (5 mL, 1 M), which resulted in the formation of a precipitate. Then a saturated aqueous solution of $NaHSO_3$ (10 mL) was added and the mixture was acidified with an aqueous solution of HCl (10 mL, 1 M). The solid was separated by filtration and the crystals were washed with water. The product was dried under reduced pressure

and obtained as beige crystals (0.96 g, 55%). ^{1}H NMR (400 MHz, [D$_8$]DMSO) *δ* 14.08 (bs, 1H; COOH), 8.19 – 8.13 (m, 2H; Ar-H), 7.78 (d, *J* = 8.3 Hz, 1H; Ar-H); ^{13}C{^{1}H} NMR (101 MHz, [D$_8$]DMSO) *δ* 164.8, 147.4, 140.9, 137.9, 129.1, 125.4, 101.2.

2-iodo-5-nitrobenzoic acid (CAS nr. 19230-50-3)

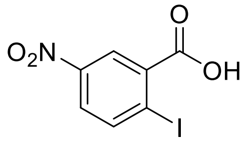

The nitro-acid (1.10 g, 5.92 mmol) was dissolved in a 30% aqueous solution of sulfuric acid (30 mL), then stirred for 1 h and cooled to 0 °C. Then an aqueous solution of $NaNO_2$ (15 mL, 0.56 M) was added dropwise (caution: an exothermic reaction was observed) and the mixture was stirred at 0 °C for 2 h. Then the solid particles were removed by filtration and washed with water (30 mL). The combined solution phase was cooled to 0 °C and an aqueous solution of KI (6 mL, 3.0 M) was added dropwise, which resulted in a vigorous reaction. After 15 minutes, the temperature of the reaction mixture was raised to RT and stirred for 90 minutes before quenching the reaction by filtration. The solid phase was washed with water, then transferred to a round bottom flask, suspended in a saturated aqueous solution of $NaHSO_3$ (10 mL) and concentrated HCl (15 mL). The product was triturated for 4 h, then filtered and washed with water. The product was dried under reduced pressure and obtained as mustard yellow crystals (1.33 g, 77%). ^{1}H NMR (400 MHz, [D$_8$]DMSO) *δ* 13.93 (bs, 1H; COOH), 8.40 (d, *J* = 2.6 Hz, 1H; Ar-H), 8.28 (d, *J* = 8.6 Hz, 1H; Ar-H), 8.00 (dd, *J* = 8.6, 2.6 Hz, 1H; Ar-H); ^{13}C{^{1}H} NMR (101 MHz, [D$_8$]DMSO) *δ* 166.5, 147.3, 142.4, 138.1, 126.1, 124.1, 103.8.

3-iodo-4-nitrobenzoic acid (CAS nr. 1086210-19-6)

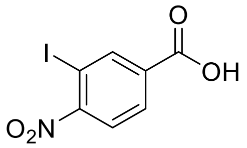

The nitro-acid (0.19 g, 1.00 mmol) was dissolved in MeOH (2 mL) and cooled to 0 °C. Thionyl chloride (0.11 mL, 1.50 mmol) was then added dropwise. The reaction mixture was subsequently warmed to 60 ° and stirred for 3 h. Next, the mixture was cooled to RT, the volatiles were removed under reduced pressure and the product was purified with column chromatography on silica gel (DCM) to provide the methyl ester (0.17 g). ^{1}H NMR (400 MHz, $CDCl_3$) *δ* 8.68 (d, *J* = 1.7 Hz, 1H; Ar-H), 8.13 (dd, *J* = 8.4, 1.7 Hz, 1H; Ar-H), 7.85 (d, *J* = 8.4 Hz, 1H; Ar-H), 3.98 (s, 3H; CH_3); ^{13}C{^{1}H} NMR (101 MHz, $CDCl_3$) *δ* 163.94, 155.67, 142.95, 134.16, 130.21, 125.05, 85.78, 53.05. The ester was dissolved in MeCN (4.0 mL), then *p*-toluenesulfonic acid monohydrate (0.58 g, 3.00 mmol) was added and the reaction mixture was cooled to 0 °C. Next, a solution of KI (0.41 g, 2.47 mmol) and $NaNO_2$ (0.14 g, 2.03 mmol) in water (0.6 mL) was added dropwise, which resulted in a vigorous reaction and the formation of an orange-brown suspension. The mixture was stirred at 0 °C for 90 minutes and then at RT for 2.5 h before quenching the reaction with the addition of water (16 mL), a saturated aqueous solution of $NaHCO_3$ (3 mL) and a saturated aqueous solution of $Na_2S_2O_3$ (6 mL). A brown precipitate formed, which was separated by filtration and suspended in a mixture of DCM (5 mL) and water (5 mL). The suspension was cooled to 0 °C, basified with an aqueous solution of NaOH (1 mL, 1 M), warmed to RT and stirred for 3 days. Then, the mixture was transferred to a separatory funnel with DCM (5 mL) and water (10 mL). The phases were separated, and the aqueous phase was washed with DCM (10 mL). Then the aqueous phase was acidified with an aqueous solution of HCl (3 mL, 1 M) and extracted with DCM (4 x 15 mL). The organic phase was dried over anhydrous $MgSO_4$, concentrated under reduced pressure to provide the desired product as a yellow solid (0.095 g, 32%). ^{1}H NMR (400 MHz, [D$_8$]DMSO) *δ* 13.78 (bs, 1H; COOH), 8.49 (s, 1H; Ar-H), 8.09 (d, *J* = 8.1 Hz, 1H; Ar-H), 8.02 (d, *J* = 8.3 Hz, 1H; Ar-H); ^{13}C{^{1}H} NMR (101 MHz, [D$_8$]DMSO) *δ* 164.7, 155.9, 141.5, 134.9, 130.3, 125.0, 88.0.

9.1 Genera procedure A

Based on minor modifications to the literature procedure:[21] The corresponding acid (0.5 mmol), 1-(3-dimethylaminopropyl)-3-ethylcarbodiimide hydrochloride (EDCI·HCl) (0.12 g, 0.60 mmol), 1-hydroxybenzotriazole (HOBt) (0.016 g, 0.10 mmol) and the corresponding amine (0.5 mmol) were dissolved in DCM (5.6 mL). The mixture was stirred overnight and quenched with the addition of a saturated solution of NH_4Cl (10 mL) and DCM (10mL). The phases were separated, and the aqueous phase was additionally extracted with DCM (2 x 10 mL). The combined organic phase was dried over anhydrous Na_2SO_4, concentrated, and purified with column chromatography on silica gel (starting from 1% of EtOAc in DCM) to provide the product after removal of the solvent under reduced pressure.

2,3,4,5-tetrafluoro-6-iodo-*N*-((1*R*)-(6-methoxyquinolin-4-yl)((2*R*,4*S*,5*R*)-5-vinylquinuclidin-2-yl)methyl)benzamide – catalyst A (CAS nr. 2220998-71-8)

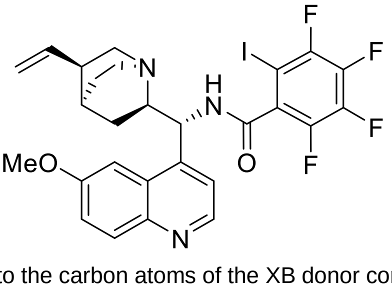

Synthesised according to the literature procedure on a 0.50 mmol scale and obtained as a colourless solid (0.18 g, 56%).[21] ^{1}H NMR (400 MHz, $CDCl_3$) *δ* 8.75 (d, *J* = 4.5 Hz, 1H; Ar-H), 8.04 (d, *J* = 9.1 Hz, 1H; Ar-H), 7.69 (bs, 1H; *N*H), 7.58 (s, 1H; Ar-H), 7.49 (d, *J* = 4.5 Hz, 1H; Ar-H), 7.40 (dd, *J* = 9.1, 2.4 Hz, 1H; Ar-H), 5.97 (ddd, *J* = 16.4, 10.4, 6.1 Hz, 1H; =CH), 5.49 (d, *J* = 9.2 Hz, 1H; CH), 5.24 – 5.07 (m, 2H; =CH_2), 4.01 (s, 3H; CH_3), 3.14 – 2.96 (m, 2H; CH_2), 2.96 – 2.81 (m, 3H; CH and CH_2), 2.34 (dd, *J* = 15.1, 7.6 Hz, 1H; CH), 1.72 (bs, 1H; CH), 1.63 – 1.55 (m, 1H; CH_2), 1.54 – 1.40 (m, 2H; CH_2), 1.17 – 1.01 (m, 1H; CH_2); ^{13}C{^{1}H} NMR *δ* 162.1, 158.0, 147.43, 145.0, 140.5, 131.8, 128.3, 126.8 (based on HMBC), 122.2, 118.7, 114.9, 101.0, 61.2, 55.6, 50.9, 49.1, 46.9, 38.9, 27.2, 26.6, 25.3 (signals corresponding to the carbon atoms of the XB donor core were not detected due to the low intensity of the signals).

***N*-((1*S*)-((2*S*,4*S*,5*R*)-5-ethylquinuclidin-2-yl)(6-methoxyquinolin-4-yl)methyl)-2,3,4,5-tetrafluoro-6-iodobenzamide – catalyst B**

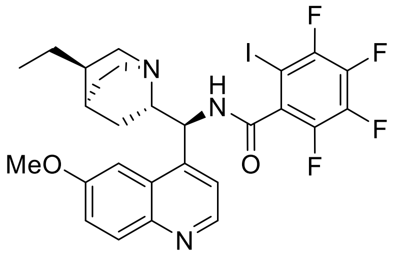

General procedure A on a 0.60 mmol scale. Column chromatography: starting from 1% of NH_3/MeOH in DCM. Obtained as a colourless solid (0.31 g, 83%). m.p. 128–130°C; $[\alpha]^D_{20}$=19.3 (*c*=0.29 in MeOH); IR (KBr): $\tilde{\nu}$=3425 (br), 1674 (s), 1623 (m), 1500 (s), 1458 (s), 1359 (m), 1241 (m) cm^{-1}; ^1H NMR (400 MHz, [D_4]methanol) *δ* 8.70 (d, *J* = 4.5 Hz, 1H; Ar-H), 7.97 (d, *J* = 9.2 Hz, 1H; Ar-H), 7.91 (bs, 1H; Ar-H), 7.62 (d, *J* = 4.5 Hz, 1H; Ar-H), 7.47 (dd, *J* = 9.2, 2.5 Hz, 1H; Ar-H), 5.89 (d, *J* = 8.5 Hz, 1H; CH), 4.04 (s, 3H; CH_3), 3.46 – 3.30 (m, 2H; CH and CH_2), 3.26 (dd, *J* = 9.9, 3.5 Hz, 1H; CH_2), 2.86 – 2.76 (m, 1H; CH_2), 2.48 (dd, *J* = 13.6, 3.5 Hz, 1H; CH_2), 1.77 – 1.67 (m, 1H; CH_2), 1.67 – 1.43 (m, 4H; CH and CH_2), 1.41 – 1.24 (m, 2H; CH_2), 0.89 – 0.82 (m, 4H; CH_2 and CH_3); $^{13}C\{^1H\}$ NMR (101 MHz, [D_4]methanol) *δ* 164.4, 159.8, 148.2, 147.0, 145.1, 131.3, 130.4, 123.8, 121.2, 103.3, 77.1 – 76.3 (m), 60.3, 58.6, 56.4, 51.8, 42.5, 38.5, 29.6, 28.4, 27.0, 26.8, 12.4 (signals corresponding to the carbon atoms of the XB donor core, besides the *C*-I signal, were not detected due to the low intensity of the signals); ^{19}F NMR (376 MHz, [D_4]methanol) *δ* -117.2 – -117.4 (m; 1F), -140.4 – -140.6 (m; 1F), -155.2 (ddd, *J* = 22.2, 18.8, 3.5 Hz; 1F), -157.0 (t, *J* = 19.4 Hz; 1F); HRMS (ESI): *m*/*z* calcd for $C_{27}H_{27}F_4IN_3O_2^+$: 628.1079 [*M*+H]$^+$; found: 628.1050.

2,3,4,5-tetrafluoro-6-iodo-*N*-((1*S*)-quinolin-4-yl((2*S*,4*S*,5*R*)-5-vinylquinuclidin-2-yl)methyl)benzamide – catalyst C (CAS nr. 2220998-67-2)

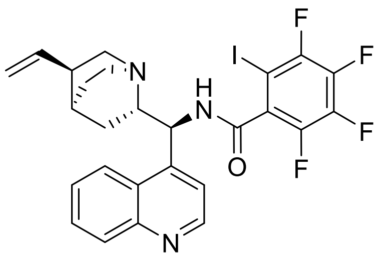

General procedure A on a 0.40 mmol scale. Column chromatography: starting from 1% of NH_3/MeOH in DCM/PE (1/1). Obtained as a fluffy colourless solid (0.21 g, 87%). ^1H NMR (400 MHz, $CDCl_3$) *δ* 8.93 (d, *J* = 4.4 Hz, 1H; Ar-H), 8.41 (bs, 1H; Ar-H), 8.16 (dd, *J* = 8.0, 0.8 Hz, 1H; Ar-H), 7.75 (t, *J* = 8.0 Hz, 1H; Ar-H), 7.69 – 7.59 (m, 2H; Ar-H and NH), 7.56 (d, *J* = 4.5 Hz, 1H; Ar-H), 5.73 – 5.60 (m, 2H; =CH and CH), 5.01 – 4.84 (m, 2H; =CH_2), 3.22 (dd, *J* = 13.9, 10.1 Hz, 1H; CH_2), 3.17 – 3.10 (m, 1H; CH_2), 3.06 – 2.90 (m, 1H; CH), 2.82 – 2.70 (m, 1H; CH_2), 2.66 (ddd, *J* = 13.9, 4.9, 2.2 Hz, 1H; CH_2), 2.36 – 2.23 (m, 1H; CH), 1.73 – 1.56 (m, 3H; CH and CH_2), 1.45 – 1.35 (m, 1H; CH_2), 1.12 – 1.03 (m, 1H; CH_2); $^{13}C\{^1H\}$ NMR *δ* 162.1, 150.0, 148.5, 146.5, 141.0, 130.5, 129.3, 127.4, 126.7, 123.4, 118.6, 114.8, 61.3, 55.8, 51.3, 40.8, 39.5, 27.8, 27.3, 25.6 (signals corresponding to the carbon atoms of the XB donor core were not detected due to the low intensity of the signals).

***N*-((1*S*)-((2*S*,4*S*,5*R*)-5-ethylquinuclidin-2-yl)(6-methoxyquinolin-4-yl)methyl)-2-iodo-5-nitrobenzamide – catalyst D**

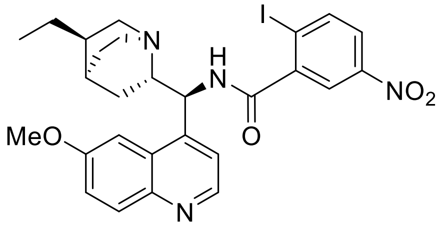

General procedure A on a 0.31 mmol scale. Column chromatography: starting from 1% of NH_3/MeOH in DCM. Obtained as a yellow solid (0.14 g, 75%); m.p. 129–132°C; $[\alpha]^D_{20}$=14.4 (*c*=0.13 in $CHCl_3$); IR (KBr): $\tilde{\nu}$=3385 (br), 1663 (s), 1621 (s), 1523 (s) 1510 (s), 1350 (s), 1240 (m) cm^{-1}; ^1H NMR (400 MHz, $CDCl_3$) *δ* 8.76 (d, *J* = 4.5 Hz, 1H; Ar-H), 8.14 (s, 1H; Ar-H), 8.06 (d, *J* = 9.2 Hz, 1H; Ar-H), 8.00 (d, *J* = 8.6 Hz, 1H; Ar-H), 7.86 (dd, *J* = 8.6, 2.6 Hz, 1H, Ar-H), 7.73 (bs, 1H; CH_2), 7.48 (d, *J* = 4.6 Hz, 1H; CH_2), 7.47 (bs, 1H; *N*H; overlap with adjacent multiples), 7.42 (dd, *J* = 9.2, 2.7 Hz, 1H; Ar-H), 5.56 (s, 1H; CH), 4.02 (s, 3H; CH_3), 3.33 – 3.06 (m, 3H; CH and CH_2), 2.84 – 2.70 (m, 1H; CH_2), 2.42 (dd, *J* = 13.7, 2.3 Hz, 1H; CH_2), 1.74 – 1.64 (m, 2H; CH and CH_2), 1.62 – 1.52 (m, 1H; CH_2), 1.51 – 1.42 (m, 2H; CH and CH_2), 1.33 – 1.18 (m, 2H; CH_2), 1.06 – 0.95 (m, 1H; CH_2), 0.81 (t, *J* = 7.3 Hz, 3H; CH_3); $^{13}C\{^1H\}$ NMR (101 MHz, $CDCl_3$) *δ* 167.1, 157.9, 147.7, 147.5, 144.8, 143.3, 141.2, 131.9, 128.6 (based on HMBC), 125.0, 123.0, 121.6, 119.0 (based on HSQC), 101.03 (based on HMBC), 100.99, 60.5 (based on HSQC), 57.7, 55.8, 51.1 (based on HMBC), 41.3, 37.2, 28.6, 27.5, 26.1, 25.0, 12.0 (the signal corresponding to the carbon atom 4` in the quinoline core was not detected due to the low intensity of the signal); HRMS (ESI): *m*/*z* calcd for $C_{27}H_{30}IN_4O_4^+$: 601.1306 [*M*+H]$^+$; found: 601.1284.

***N*-((1*S*)-((2*S*,4*S*,5*R*)-5-ethylquinuclidin-2-yl)(6-methoxyquinolin-4-yl)methyl)-5-iodo-2-nitrobenzamide – catalyst E**

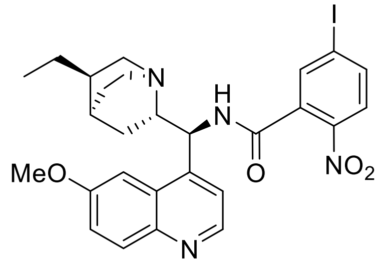

General procedure A on a 0.31 mmol scale. Column chromatography: starting from 1% of NH_3/MeOH in DCM. Obtained as a beige solid (0.077 g, 41%); m.p. ND, decomposes above 210°; $[\alpha]^D_{20}$=13.0 (*c*=0.29 in $CHCl_3$); IR (KBr): $\tilde{\nu}$=3416 (br), 1666 (s), 1621 (m), 1530 (s) 1511 (s), 1348 (s), 1240 (m) cm^{-1}; ^1H NMR (400 MHz, $CDCl_3$) *δ* 8.79 (d, *J* = 4.0 Hz, 1H, Ar-H), 8.06 (d, *J* = 9.2 Hz, 1H, Ar-H), 7.88 (dd, *J* = 8.6, 1.9 Hz, 1H; Ar-H), 7.78 (s, 1H; Ar-H), 7.75 (d, *J* = 8.6 Hz, 1H; Ar-H), 7.69 (d, *J* = 2.4 Hz, 1H; Ar-H), 7.46 (d, *J* = 4.0 Hz, 1H; Ar-H), 7.44 (bs, 1H; *N*H; overlap with adjacent multiples) 7.42 (dd, *J* = 9.2, 2.4 Hz, 1H; Ar-H), 5.49 (s, 1H; CH), 4.00 (s, 3H; CH_3), 3.31 – 3.14 (m, 1H; CH_2), 3.20 (dd, *J* = 13.6, 9.9 Hz, 1H; CH_2), 3.11 – 2.88 (m, 1H; CH), 2.81 – 2.63 (m, 1H; CH_2), 2.41 – 2.33 (m, 1H; CH_2), 1.74 – 1.61 (m, 2H; CH and CH_2), 1.61 – 1.51 (m, 1H; CH_2), 1.49 – 1.36 (m, 2H; CH and CH_2), 1.31 – 1.14 (m, 2H; CH_2), 1.10 – 0.96 (m, 1H; CH_2), 0.80 (t, *J* = 7.3 Hz, 3H; CH_3); $^{13}C\{^1H\}$ NMR (101 MHz, $CDCl_3$) *δ* 164.9, 157.9 (based on HMBC), 147.6, 145.7, 144.8 (based on HMBC), 139.4, 137.8, 134.2, 131.8, 128.4 (based on HMBC), 125.7, 121.5 (based on HSQC); 118.3 (based on HSQC), 101.7 (based on HSQC), 101.1, 60.7 (based on HSQC), 57.6, 55.7, 51.3 (based on HMBC), 40.8, 37.2, 28.6, 27.4, 25.8 (based on HSQC) ; 25.0, 12.0 (signal corresponding to the carbon atom 4` in the quinoline core was not detected due to the low intensity of the signals); HRMS (ESI): *m*/*z* calcd for $C_{27}H_{30}IN_4O_4^+$: 601.1306 [*M*+H]$^+$; found: 601.1301.

N-((1S)-((2S,4S,5R)-5-ethylquinuclidin-2-yl)(6-methoxyquinolin-4-yl)methyl)-3-iodo-4-nitrobenzamide – catalyst F

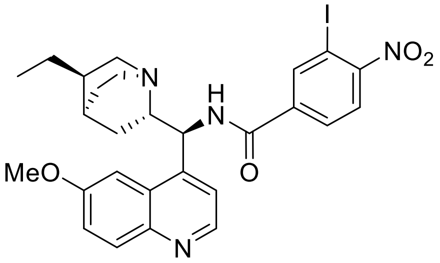

General procedure A on a 0.25 mmol scale, stirred for 6 h. Column chromatography: starting from 0.8% of NH_3/MeOH in DCM. Obtained as a pale-yellow solid (0.10 g, 67%); m.p. 118–119°C; $[\alpha]^D_{20}$=-168.7 (*c*=0.38 in $CHCl_3$); IR (KBr): $\tilde{\nu}$=3416 (br), 1622 (m), 1530 (s) 1509 (s), 1343 (m), 1241 (m) cm^{-1}; ^1H NMR (400 MHz, $CDCl_3$) *δ* 8.72 (d, *J* = 4.6 Hz, 1H; Ar-H), 8.39 (d, *J* = 1.4 Hz, 1H; Ar-H), 8.04 (d, *J* = 9.4 Hz, 1H; Ar-H), 7.93 (bs, 1H; *N*H), 7.88 – 7.77 (m, 2H; Ar-H), 7.68 (d, *J* = 2.6 Hz, 1H; Ar-H), 7.41 (dd, *J* = 9.4, 2.6 Hz, 1H; Ar-H), 7.39 (d, *J* = 4.6 Hz, 1H; Ar-H), 5.38 (bs, 1H; CH), 4.00 (s, 3H; CH_3), 3.27 (dd, *J* = 13.7, 9.9 Hz, 1H; CH_2), 3.21 – 3.13 (m, 1H; CH), 3.12 – 3.02 (m, 1H; CH_2), 2.80 – 2.67 (m, 1H; CH_2), 2.48 (ddd, *J* = 13.7, 4.9, 2.2 Hz, 1H; CH_2), 1.71 – 1.61 (m, 2H; CH and CH_2), 1.60 – 1.54 (m, 1H; CH_2), 1.53 – 1.46 (m, 1H; CH), 1.45 – 1.39 (m, 1H; CH_2), 1.31 – 1.21 (m, 2H; CH_2), 1.01 (dd, *J* = 13.6, 6.7 Hz, 1H; CH_2), 0.82 (t, *J* = 7.3 Hz, 3H; CH_3); $^{13}C\{^1H\}$ NMR (101 MHz, $CDCl_3$) *δ* 163.9, 157.9, 154.6, 147.6, 144.8, 140.9, 140.1, 138.2, 132.0, 128.2 (based on HMBC), 127.8, 125.3, 121.5, 119.0 (based on HSQC), 101.8, 86.3, 60.2 (based on HSQC), 57.7, 55.7, 41.0, 37.2, 28.5, 27.4, 25.9, 25.0, 12.0 (signals corresponding to the carbon atoms 4\` and 9 of the alkaloid fragment were not detected due to the low intensity of the signals); HRMS (ESI): *m/z* calcd for $C_{27}H_{30}IN_4O_4^+$: 601.1306 $[M+H]^+$; found: 601.1307.

N-((1S)-((2S,4S,5R)-5-ethylquinuclidin-2-yl)(6-methoxyquinolin-4-yl)methyl)-2,3,4,5-tetrafluorobenzamide – catalyst G

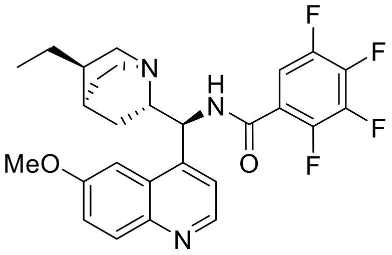

General procedure A on a 0.50 mmol scale using the corresponding hydrochloride salt instead of the amine and an additional amount of Et_3N (0.17 mL, 1.25 mmol), stirred for 5 h, extracted once with DCM. Dried with K_2CO_3. Column chromatography: starting from 2% of NH_3/MeOH in DCM. Obtained as a colourless solid (0.14 g, 55%); m.p. 82–84°C; $[\alpha]^D_{20}$=-153.9 (*c*=0.28 in $CHCl_3$); IR (KBr): $\tilde{\nu}$=3346 (br), 1673 (m), 1622 (m), 1510 (s) 1480 (s), 1362 (m), 1241 (m), 1034 (m) cm^{-1}; ^1H NMR (400 MHz, $CDCl_3$) *δ* 8.74 (d, *J* = 4.5 Hz, 1H; Ar-H), 8.20 (bs, 1H; *N*H), 8.04 (d, *J* = 9.2 Hz, 1H; Ar-H), 7.62 (bs; 1H, Ar-H), 7.57 (dddd, *J* = 10.6, 8.6, 6.5, 2.4 Hz, 1H; Ar-H), 7.44 – 7.36 (m, 2H; Ar-H), 5.35 (s, 1H; CH), 3.97 (s, 3H; CH_3), 3.27 (dd, *J* = 13.7, 9.9 Hz, 1H; CH_2), 3.16 – 3.01 (m, 1H; CH), 2.80 – 2.66 (m, 1H; CH_2), 2.47 (ddd, *J* = 13.7, 4.7, 2.2 Hz, 1H; CH_2), 1.69 – 1.65 (m, *J* = 2.3 Hz, 1H; CH), 1.62 (ddd, *J* = 13.6, 5.4, 3.1 Hz, 1H; CH_2), 1.59 – 1.52 (m, 1H; CH_2), 1.52 – 1.44 (m, 1H; CH), 1.40 (ddd, *J* = 13.4, 10.0, 3.4 Hz, 1H; CH_2), 1.32 – 1.15 (m, 2H; CH_2), 1.01 (dd, *J* = 13.4, 7.0 Hz, 1H; CH_2), 0.81 (t, *J* = 7.3 Hz, 3H; CH_3); $^{13}C\{^1H\}$ NMR (101 MHz, $CDCl_3$) *δ* 160.4, 158.0, 147.8, 145.9, 145.0, 132.2, 128.0 (based on HMBC), 121.7, 117.8 – 117.5 (m), 113.1 – 112.6 (m), 101.7, 57.8, 55.7, 41.1, 37.5, 28.8, 27.6, 26.1, 25.2, 12.1 (signals corresponding to the carbon atoms in positions 4\`, 8, 9 of the alkaloid fragment and the *C*F carbon atoms of the fluorinated core were not detected due to the low intensity of the signals); ^{19}F NMR (376 MHz, $CDCl_3$) *δ* -136.9 (bs; 1F), -138.7 (bs; 1F), -149.1 – -149.4 (m; 1F), -154.0 – -154.1 (m; 1F); HRMS (ESI): *m/z* calcd for $C_{27}H_{27}F_4N_3O_2+Na^+$: 524.1932 $[M+Na]^+$; found: 524.1925.

N-((1S)-((2S,4S,5R)-5-ethylquinuclidin-2-yl)(6-methoxyquinolin-4-yl)methyl)-3-nitrobenzamide – catalys H

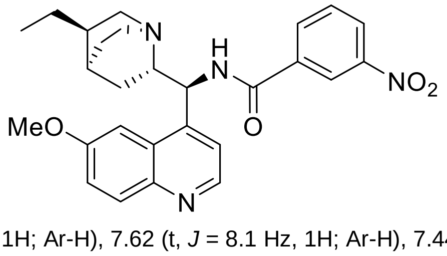

General procedure A on a 0.50 mmol scale using the corresponding hydrochloride salt instead of the amine and an additional amount of Et_3N (0.18 mL, 1.30 mmol), stirred for 2.5 h, extracted once with DCM. Column chromatography: starting from 1% of NH_3/MeOH in DCM. Obtained as a pale-yellow solid (0.11 g, 46%), m.p. 102–105°C; $[\alpha]^D_{20}$=-177.8 (*c*=0.31 in $CHCl_3$); IR (KBr): $\tilde{\nu}$=3346 (br), 1656 (m), 1621 (m), 1530 (s) 1510 (s), 1351 (s), 1240 (m) cm^{-1}; ^1H NMR (400 MHz, $CDCl_3$) *δ* 8.74 (d, *J* = 4.6 Hz, 1H; Ar-H), 8.62 (t, *J* = 2.0 Hz, 1H; Ar-H), 8.34 (ddd, *J* = 8.1, 2.0, 1.0 Hz, 1H; Ar-H), 8.16 – 8.12 (m, 1H; Ar-H), 8.04 (d, *J* = 9.2 Hz, 1H; Ar-H), 7.93 (bs, 1H; *N*H), 7.70 (d, *J* = 2.6 Hz, 1H; Ar-H), 7.62 (t, *J* = 8.1 Hz, 1H; Ar-H), 7.44 (d, *J* = 4.6 Hz, 1H; Ar-H), 7.41 (dd, *J* = 9.2, 2.6 Hz, 1H; Ar-H), 5.38 (s, 1H; CH), 4.01 (s, 3H; CH_3), 3.28 (dd, *J* = 13.7, 9.9 Hz, 1H; CH_2), 3.23 – 3.15 (m, 1H; CH), 3.13 – 3.04 (m, 1H; CH_2), 2.79 – 2.69 (m, 1H; CH_2), 2.50 (ddd, *J* = 13.7, 4.9, 2.2 Hz, 1H; CH_2), 1.72 – 1.62 (m, 2H; CH and CH_2), 1.61 – 1.54 (m, 1H; CH_2), 1.54 – 1.39 (m, 2H; CH and CH_2), 1.34 – 1.20 (m, 2H; CH_2), 1.04 (dd, *J* = 13.6, 6.8 Hz, 1H; CH_2), 0.83 (t, *J* = 7.3 Hz, 3H; CH_3); $^{13}C\{^1H\}$ NMR (101 MHz, $CDCl_3$) *δ* 165.0, 157.9, 148.2, 147.6, 144.8, 135.7, 133.4, 132.0, 129.8, 128.3 (based on HMBC), 126.2, 122.1, 121.6, 119.4 (based on HSQC), 101.8, 60.1, 57.7, 55.6, 41.0, 37.3, 28.6, 27.5, 26.0, 25.0, 12.0 (signals corresponding to the carbon atoms 4\`and 9 of the alkaloid fragment were not detected due to the low intensity of the signals); HRMS (ESI): *m/z* calcd for $C_{27}H_{31}N_4O_4^+$: 475.2340 $[M+H]^+$; found: 475.2334.

N-((1S)-((2S,4S,5R)-5-ethylquinuclidin-2-yl)(6-methoxyquinolin-4-yl)methyl)-2-nitrobenzamide – catalyst I

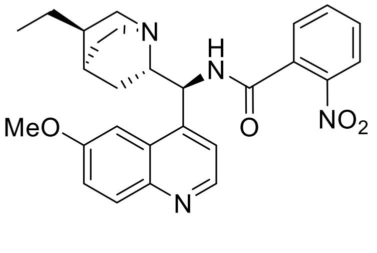

General procedure A on a 0.40 mmol scale using the corresponding hydrochloride salt instead of the amine and an additional amount of Et_3N (0.14 mL, 1.00 mmol), stirred for 5.5 h. Column chromatography: starting from 1% of NH_3/MeOH in DCM /PE(1/1). Obtained as a colourless solid (0.10 g, 55%); m.p. 224–227°C; $[\alpha]^D_{20}$=32.0 (*c*=0.16 in $CHCl_3$); IR (KBr): $\tilde{\nu}$=3397 (br), 1673 (s), 1621 (m), 1534 (s) 1510 (s), 1355 (m), 1230 (m) cm^{-1}; ^1H NMR (400 MHz, $CDCl_3$) *δ* 8.79 (d, *J* = 4.5 Hz, 1H; Ar-H), 8.08 – 8.02 (m, 1H; Ar-H), 7.71 (s, 1H; Ar-H), 7.64 (dt, *J* = 7.7, 1.0 Hz, 1H; Ar-H), 7.55 (td, *J* = 7.7, 1.5 Hz, 1H; Ar-H), 7.48 (d, *J* = 4.5 Hz, 1H; Ar-H), 7.51 – 7.43 (m, 1H; Ar-H), 7.41 (dd, *J* = 9.2, 2.7 Hz, 1H; Ar-H), 7.34 (bs, 1H; *N*H; overlap with adjacent multiples), 5.52 (bs, 1H; CH), 4.00 (s, 3H; CH_3), 3.24 (bs, 1H; CH_2), 3.19 (dd, *J* = 13.8, 9.9 Hz,

1H; CH_2), 3.01 (bs, 1H, CH), 2.78 – 2.68 (m, 1H; CH_2), 2.36 (dd, *J* = 13.8, 3.0 Hz, 1H; CH_2), 1.74 – 1.62 (m, 2H; CH and CH_2), 1.61 – 1.52 (m, 1H; CH_2), 1.50 – 1.36 (m, 2H; CH and CH_2), 1.28 – 1.16 (m, 2H; CH_2), 1.10 – 0.99 (m, 1H; CH_2), 0.79 (t, *J* = 7.3 Hz, 3H; CH_3); $^{13}C\{^1H\}$ NMR (101 MHz, $CDCl_3$) *δ* 157.9 (based on HMBC), 147.6, 146.3, 144.8 (based on HMBC), 133.7, (based on HSQC), 130.3, 128.9, 124.5, 121.7 (based on HSQC), 118.8 (based on HSQC), 101.8, 60.5 (based on HSQC), 57.7, 55.7, 40.8, 37.2, 28.6, 27.4, 26.0 (based on HSQC), 25.0, 12.0 (signals corresponding to the carbon atoms at position 4`, 9` and 9 of the alkaloid fragment, also the carbonyl carbon and the ipso-carbon of the amide fragment were not detected due to the low intensity of the signals); HRMS (ESI): *m*/*z* calcd for $C_{27}H_{31}N_4O_4^+$: 475.2340 [*M*+H]$^+$; found: 475.2331.

***N*-((1*S*)-((2*S*,4*S*,5*R*)-5-ethylquinuclidin-2-yl)(6-methoxyquinolin-4-yl)methyl)-4-nitrobenzamide – catalyst J**

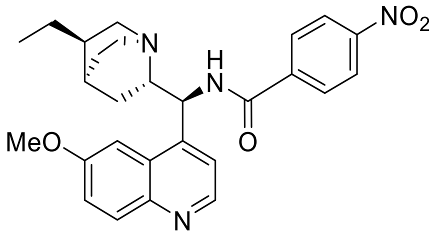

General procedure A on a 0.22 mmol scale, stirred for 4 h. Column chromatography: starting from 1% of NH_3/MeOH in DCM. Obtained as a pale-yellow solid (0.09 g, 86%); m.p. 109–111°C; $[\alpha]^D_{20}$=-205.9 (*c*=0.28 in $CHCl_3$); IR (KBr): $\tilde{\nu}$=3417 (br), 1659 (m), 1622 (m), 1525 (s) 1510 (s), 1344 (m), 1241 (m) cm^{-1}; ^1H NMR (400 MHz, $CDCl_3$) *δ* 8.75 (d, *J* = 4.6 Hz, 1H, Ar-H), 8.33 – 8.22 (m, 2H, Ar-H), 8.05 (d, *J* = 9.2 Hz, 1H, Ar-H), 8.00 – 7.90 (m, 3H; Ar-H and *N*H), 7.68 (d, *J* = 2.6 Hz, 1H; Ar-H), 7.49 – 7.35 (m, 2H; Ar-H), 5.35 (s, 1H; CH), 3.99 (s, 3H; CH_3), 3.28 (dd, *J* = 13.6, 9.9 Hz, 1H; CH_2), 3.21 – 3.00 (m, 2H; CH and CH_2), 2.81 – 2.68 (m, 1H; CH_2), 2.48 (ddd, *J* = 13.6, 4.9, 2.2 Hz, 1H; CH_2), 1.67 – 1.61 (m, 2H; CH and CH_2), 1.61 – 1.54 (m, 1H; CH_2), 1.53 – 1.38 (m, 2H; CH and CH_2), 1.33 – 1.20 (m, 2H; CH_2), 1.04 (dd, *J* = 13.6, 6.9 Hz, 1H; CH_2), 0.82 (t, *J* = 7.3 Hz, 3H; CH_3); $^{13}C\{^1H\}$ NMR (101 MHz, $CDCl_3$) *δ* 165.18, 157.88, 149.67, 147.61, 144.82, 139.49, 132.00, 128.35, 128.2 (based on HMBC), 123.78, 121.46, 119.33 (based on HSQC), 101.83, 60.4 (based on HSQC), 57.75, 55.64, 41.03, 37.25, 28.57, 27.44, 25.93, 24.99, 11.96 (signals corresponding to the carbon atoms at 4`and 9 of the alkaloid fragment were not detected due to the low intensity of the signals); HRMS (ESI): *m*/*z* calcd for $C_{27}H_{31}N_4O_4^+$: 475.2340 [*M*+H]$^+$; found: 475.2346.

***N*-((1*S*)-((2*S*,4*S*,5*R*)-5-ethylquinuclidin-2-yl)(6-methoxyquinolin-4-yl)methyl)-2,3,4,5,6-pentafluorobenzamide – catalyst K**

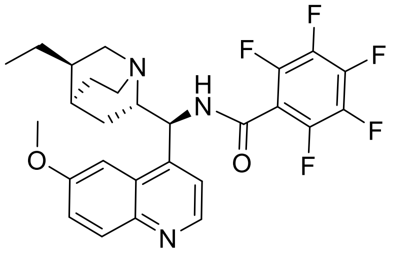

The corresponding hydrochloride salt of the amine (0.14 g, 0.40 mmol) was suspended in DCM (2.0 mL) and cooled to 0 °C. Then triethylamine (0.11 mL, 0.80 mmol) and 2,3,4,5,6-pentafluorobenzoyl chloride (0.06 mL, 0.40 mmol) were added. The mixture was stirred at RT for 6 h and quenched with the addition of water (10 mL) and DCM (10mL). The phases were separated, and the aqueous phase was additionally extracted with DCM (1 x 10 mL). The combined organic phase was dried over anhydrous Na_2SO_4, concentrated, and purified with column chromatography on silica gel (0.8% NH_3/MeOH in DCM) to provide the product after removal of the solvent under reduced pressure as a colourless solid (0.11 g, 53%). $[\alpha]^D_{20}$=-63.6 (*c*=0.14 in $CHCl_3$); m.p. 157–160°C; IR (film): $\tilde{\nu}$=2934 (w), 1682 (m), 1503 (s), 1241 (w), 991(m) cm^{-1}; ^1H NMR (400 MHz, $CDCl_3$) δ 8.77 (d, *J* = 4.5 Hz, 1H, Ar-H), 8.05 (d, *J* = 9.2 Hz, 1H, Ar-H), 7.60 (bs, 2H, Ar-H, *N*H), 7.46 – 7.38 (m, 2H, Ar-H), 5.44 (bs, 1H, CH), 3.99 (s, 3H, CH_3), 3.22 (dd, *J* = 13.7, 9.9 Hz, 1H, CH_2), 3.15 – 2.86 (m, 2H, CH, CH_2), 2.78 – 2.67 (m, 1H, CH_2), 2.40 (ddd, *J* = 13.7, 4.8, 2.2 Hz, 1H, CH_2), 1.73 – 1.61 (m, 2H, CH, CH_2), 1.60 – 1.52 (m, 1H, CH_2), 1.50 – 1.44 (m, 1H, CH), 1.44 – 1.34 (m, 1H, CH_2), 1.29 – 1.17 (m, 2H, CH_2), 1.03 (dd, *J* = 13.7, 7.0 Hz, 1H, CH_2), 0.80 (t, *J* = 7.3 Hz, 3H, CH_3); $^{13}C\{^1H\}$ NMR (101 MHz, $CDCl_3$) δ 157.9, 157.3, 147.6, 145.9– 144.7 (m), 144.8 (based on HMBC), 143.4 – 140.9 (m), 139.2 – 135.8 (m), 132.0, 128.8 (based on HMBC), 121.7, 118.4 (based on HSQC), 111.7 – 111.0 (m), 101.6, 61.1 (based on HSQC), 57.6, 55.6, 51.7 (based on HMBC), 40.7, 37.3, 28.6, 27.4, 25.8, 25.0, 12.0; ^{19}F NMR (376 MHz, $CDCl_3$) δ -140.5 (d, *J* = 17.3 Hz), -150.8 (t, *J* = 20.4 Hz), -160.1 (bs); HRMS (ESI): *m*/*z* calcd for $C_{27}H_{27}F_5N_3O_2^+$: 520.2018 [*M*+H]$^+$; found: 520.2018.

(1*S*)-((2*S*,4*S*,5*R*)-5-ethylquinuclidin-2-yl)(6-methoxyquinolin-4-yl)methyl 2,3,4,5-tetrafluoro-6-iodobenzoate – catalyst L

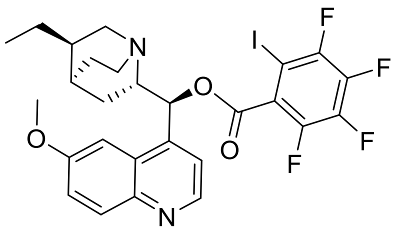

2,3,4,5-Tetrafluoro-6-iodobenzoic acid (0.10 g, 0.31 mmol) was dissolved in $SOCl_2$ (1.5 mL) and heated at 85 °C for 2 h under argon atmosphere. The mixture was cooled to RT and $SOCl_2$ was removed under reduced pressure (for 2 h). Then the crude mixture was placed under argon atmosphere, DCM (0.5 mL) was added. The mixture was cooled to 0 °C, triethylamine (0.09 mL) was added followed by the *epi*-dihydroquinine (0.090 g, 0.28 mmol) in DCM (1.1 mL). Then the mixture was stirred at RT for 2 h and quenched with the addition of a saturated solution of NH_4Cl (10 mL) and DCM (10mL). The phases were separated, and the aqueous phase was additionally extracted with DCM (1 x 10 mL). The combined organic phase was dried over anhydrous Na_2SO_4, concentrated, and purified with column chromatography on silica gel (0.75% NH_3/MeOH in DCM) to provide the product after removal of the solvent under reduced pressure as a colourless solid (0.065g, 37%). $[\alpha]^D_{20}$=50.6 (*c*=0.10 in $CHCl_3$); m.p. 76–78°C; IR (film): $\tilde{\nu}$=2932 (m), 1738 (m), 1622 (m), 1504 (s), 1460 (s), 1364 (m), 1220 (s), 966 (m) cm^{-1}; ^1H NMR (400 MHz, $CDCl_3$) δ 8.81 (d, *J* = 4.5 Hz, 1H, Ar-H), 8.06 (d, *J* = 9.2 Hz, 1H, Ar-H), 7.60 (d, *J* = 2.6 Hz, 1H, Ar-H), 7.51 (d, *J* = 4.5 Hz, 1H, Ar-H), 7.43 (dd, *J* = 9.2, 2.6 Hz, 1H, Ar-H), 6.69 (bs, 1H, CH), 4.00 (s, 3H, CH_3), 3.60 – 3.41 (m, 1H, CH), 3.38 – 3.26 (m, 1H, CH_2), 3.21 (dd, *J* = 13.7, 9.8 Hz, 1H, CH_2), 2.85 – 2.73 (m, 1H, CH_2), 2.46 (ddd, *J* = 13.8, 4.4, 2.2 Hz, 1H, CH_2), 1.68 – 1.65 (m, 1H, CH), 1.64 – 1.58 (m, 1H, CH_2), 1.57 – 1.51 (m, 1H, CH_2), 1.50 – 1.36 (m, 2H, CH, CH_2), 1.34 – 1.24 (m, 2H, CH_2), 0.91 – 0.84 (m, 1H, CH_2), 0.83 (t, *J* = 7.3 Hz, 3H, CH_3); $^{13}C\{^1H\}$ NMR (101 MHz, $CDCl_3$) δ 162.0, 158.2, 147.5, 144.9, 140.4 (based on HMBC), 132.0, 127.7, 122.1, 120.6 (based on HSQC), 101.7 (based on HSQC), 59.0 (based on HSQC), 57.7, 55.7, 41.5, 37.3, 28.7, 27.6, 25.2, 24.9, 12.1 (signals corresponding to the carbon atoms of the XB donor core were not detected due

to the low intensity of the signals); ^{19}F NMR (376 MHz, $CDCl_3$) δ -112.3 – -112.5 (m), -135.3 – -135.4 (m), -149.4 (t, *J* = 20.4 Hz), -152.4 (td, *J* = 21.3, 4.3 Hz); HRMS (ESI): *m/z* calcd for $C_{27}H_{26}F_4IN_2O_3^+$: 629.0919 [*M*+H]$^+$; found: 629.0922.

***N*-((1*S*)-((2*S*,4*S*,5*R*)-5-ethylquinuclidin-2-yl)(6-methoxyquinolin-4-yl)methyl)-2,3,4,5-tetrafluoro-6-iodo-N-methylbenzamide – catalyst M**

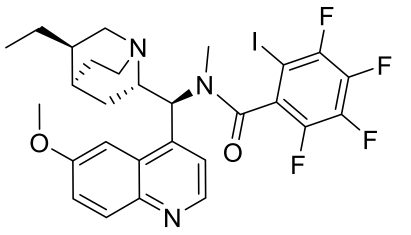

General procedure A on a 0.30 mmol scale for 3 days at 30 °C. Column chromatography: starting from 0.2% of NH_3/MeOH in DCM. Obtained as a yellow amorphous solid (0.030 g, 16%). $[\alpha]^D_{20}$=-112.8 (*c*=0.07 in $CHCl_3$); **Isomer 1**: ^{1}H NMR (400 MHz, $CDCl_3$) δ 8.79 (d, *J* = 4.5 Hz, 1H, Ar-H), 8.20 (d, *J* = 2.7 Hz, 1H, Ar-H), 8.01 (d, *J* = 9.2 Hz, 1H, Ar-H), 7.41 – 7.36 (m, 1H, Ar-H), 7.31 (d, *J* = 4.6 Hz, 1H, Ar-H), 6.47 (d, *J* = 9.1 Hz, 1H, CH), 4.09 (s, 3H, CH_3), 3.69 – 3.49 (m, 1H, CH), 3.49 – 3.36 (m, 1H, CH_2), 3.33 – 3.25 (m, 1H, CH_2), 2.79 – 2.67 (m, 1H, CH_2), 2.54 (s, 3H, CH_3), 2.56 – 2.47 (m, 1H, CH_2), 1.91 – 1.79 (m, 1H, CH_2), 1.78 – 1.66 (m, 1H, CH), 1.68 – 1.55 (m, 2H, CH_2), 1.55 – 1.40 (m, 1H for CH, 1H for CH_2), 0.98 – 0.88 (m, 3H for CH_3 and 1H for CH_2), 0.81 – 0.69 (m, 1H, CH_2); ^{13}C{1H} NMR (101 MHz, $CDCl_3$) δ 163.8, 158.4, 147.0, 145.3, 139.7, 131.5, 129.7, 123.1, 120.4, 103.7, 58.8, 56.2, 54.4, 53.6, 41.9, 37.6, 30.6, 28.6, 28.1, 27.6, 25.6, 12.3 (the atoms of the XB donor core were not detected due to the low intensity of the signals); ^{19}F NMR (376 MHz, $CDCl_3$) δ -114.3 (ddd, *J* = 21.3, 10.9, 3.6 Hz), -136.2 (dd, *J* = 20.8, 10.5 Hz), -151.7 – -152.4 (m); **Isomer 2**: ^{1}H NMR (400 MHz, $CDCl_3$) δ 8.78 (d, J = 4.6 Hz, 1H, Ar-H), 8.02 (d, J = 9.2 Hz, 1H, Ar-H), 7.87 (d, J = 2.7 Hz, 1H, Ar-H), 7.43 – 7.38 (m, 1H, Ar-H), 7.28 (d, J = 4.7 Hz, 1H, Ar-H), 6.44 (d, *J* = 9.0 Hz, 1H, CH), 3.95 (s, 3H, CH_3), 3.69 – 3.49 (m, 1H for CH, 1H for CH_2), 3.25 – 3.17 (m, 1H, CH_2), 2.79 – 2.67 (m, 1H, CH_2), 2.53 (s, 3H, CH_3), 2.56 – 2.47 (m, 1H, CH_2), 1.91 – 1.79 (m, 1H, CH_2), 1.78 – 1.66 (m, 1H, CH), 1.68 – 1.55 (m, 2H, CH_2), 1.55 – 1.40 (m, 1H for CH and 1H for CH_2), 0.98 – 0.88 (m, 3H for CH_3 and 1H for CH_2), 0.81 – 0.69 (m, 1H, CH_2). ^{13}C{1H} NMR (101 MHz, $CDCl_3$) δ 163.2, 158.7, 146.8, 145.3, 139.8, 131.6, 129.5, 123.2, 120.5, 102.6, 58.6, 56.1, 54.1, 53.7, 42.6, 37.8, 30.6, 28.7, 28.2, 28.0, 25.7, 12.4 (the atoms of the XB donor core were not detected due to the low intensity of the signals); ^{19}F NMR (376 MHz, $CDCl_3$) δ -113.2 (ddd, *J* = 22.6, 10.9, 4.5 Hz), -138.4 (ddd, *J* = 22.1, 10.9, 3.0 Hz), -151.7 – -152.4 (m); HRMS (ESI): *m/z* calcd for $C_{28}H_{29}F_4IN_3O_2^+$: 642.1235 [*M*+H]$^+$; found: 642.1242.

2,3,4,5-tetrafluoro-6-iodo-*N*-methylbenzamide - 9

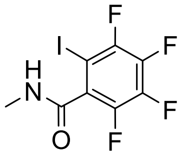

General procedure A on a 0.5 mmol scale, 2 equivalents of $MeNH_2$ HCl instead of 1 equivalent of amine, additionally Et_3N (0.21 mL, 1.50 mmol) was added. Column chromatography: starting from 3% of EtOAc in DCM. Obtained as a colourless solid (0.082 g, 49%). m.p. 176–177°C; IR (KBr): $\tilde{\nu}$= 3289 (m), 1651 (s), 1504 (s), 1459 (s), 1368 (m), 1119 (w) cm^{-1}; ^{1}H NMR (400 MHz, [D_6]DMSO) *δ* 8.67 (q, *J* = 4.5 Hz, 1H, *N*H), 2.79 (d, *J* = 4.7 Hz, 3H; CH_3); ^{13}C{^{1}H} NMR (400 MHz, [D_6]DMSO) *δ* 162.4, 148.4 – 145.4 (m), 144.8 – 141.8 (m), 141.0 – 137.9 (m), 140.5 – 137.7 (m), 128.0 (dd, *J* = 19.7, 3.6 Hz), 78.5 – 77.9 (m), 26.1; ^{19}F NMR (400 MHz, [D_8]DMSO) *δ* -113.1 (ddd, *J* = 22.7, 10.9, 4.8 Hz), -137.7 (ddd, *J* = 21.8, 10.9, 4.0 Hz), -150.6 (ddd, *J* = 22.7, 19.6, 4.0 Hz), -152.2 (ddd, *J* = 21.8, 19.6, 4.8 Hz). ^{19}F NMR (376 MHz, $CDCl_3$) *δ* -113.9 (ddd, *J* = 22.4, 11.0, 4.8 Hz; 1F), -138.0 (ddd, *J* = 21.6, 11.0, 3.4 Hz; 1F), -151.5 (ddd, *J* = 22.4, 19.4, 3.4 Hz; 1F), -152.1 (ddd, *J* = 21.6, 19.4, 4.8 Hz; 1F); HRMS (ESI): *m/z* calcd for $C_8H_5F_4INO^+$: 333.9346 [*M*+H]$^+$; found: 333.9340.

2,3,4,5-tetrafluoro-*N*-methylbenzamide – 10 (CAS nr. 129725-50-4)

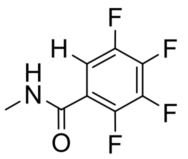

General procedure A on a 1.00 mmol scale, 2 equivalents of $MeNH_2$ HCl instead of 1 equivalent of amine, additionally Et_3N (0.42 mL, 3.00 mmol) was added. Column chromatography: starting from 3% of EtOAc in DCM. Obtained as a colourless solid (0.106 g, 51%). m.p. 99–100°C; IR (KBr): $\tilde{\nu}$= 3330 (m), 1640 (s), 1571 (m), 1479 (s), 1465 (m), 1198 (m) cm^{-1}; ^{1}H NMR (400 MHz, $CDCl_3$) *δ* 7.86 – 7.69 (m, 1H, Ar-H), 6.61 (bs, 1H; *N*H), 3.05 (dd, *J* = 4.8, 1.1 Hz, 3H; CH_3); ^{13}C{^{1}H} NMR (400 MHz, $CDCl_3$) *δ* 161.0, 148.6 – 147.1 (m), 146.1 – 144.6 (m), 144.1 – 141.5 (m), 142.0 – 139.1 (m), 117.6 – 117.3 (m), 113.1 – 112.7 (m), 27.2; ^{19}F NMR (376 MHz, $CDCl_3$) *δ* -136.8 (dddd, *J* = 21.2, 13.6, 10.6, 2.8 Hz; 1F), -139.9 – -140.1 (m; 1F), -149.3 (dddd, *J* = 21.2, 19.2, 8.2, 6.8 Hz; 1F), -154.1 (ddt, *J* = 22.4, 19.2, 2.8 Hz; 1F); HRMS (ESI): *m/z* calcd for $C_8H_6F_4NO^+$: 208.0380 [*M*+H]$^+$; found: 208.0380.

2,3,4,5-tetrafluoro-6-iodo-*N*,*N*-dimethylbenzamide – 11

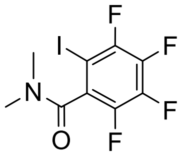

General procedure A on a 0.5 mmol scale, 2 equivalents of Me_2NH/THF solution (2 M) instead of 1 equivalent of amine. Column chromatography: starting from 1% of EtOAc in DCM). Obtained as a colourless solid (0.09 g, 52%). m.p. 126–128°C; (KBr): $\tilde{\nu}$= 1645 (s), 1496 (m), 1462 (m), 1166 (m) cm^{-1}; ^{1}H NMR (400 MHz, $CDCl_3$) *δ* 3.17 (s, 3H; CH_3), 2.90 (s, 3H; CH_3); ^{13}C{^{1}H} NMR (101 MHz, $CDCl_3$) *δ* 163.4, 149.4 – 146.7 (m), 144.8 – 142.2 (m), 142.2 – 139.2 (m), 141.6 – 138.2 (m), 127.0 – 126.7 (m), 74.7 – 74.2 (m), 37.8, 35.0; ^{19}F NMR (376 MHz, $CDCl_3$) *δ* -113.9 (ddd, *J* = 22.4, 11.0, 4.8 Hz; 1F), -138.0 (ddd, *J* = 21.6, 11.0, 3.4 Hz; 1F), -151.5 (ddd, *J* = 22.4, 19.4, 3.4 Hz; 1F), -152.1 (ddd, *J* = 21.6, 19.4, 4.8 Hz; HRMS (ESI): *m/z* calcd for $C_9H_7F_4INO^+$: 347.9503 [*M*+H]$^+$; found: 347.9505.

methyl 2,3,4,5-tetrafluoro-6-iodobenzoate – 12

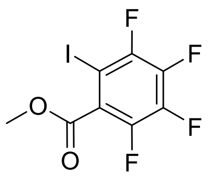

2,3,4,5-Tetrafluoro-6-iodobenzoic acid (0.20 g, 0.63 mmol) was dissolved in $SOCl_2$ (1.5 mL) and heated at 85 °C for 2 h under argon atmosphere. The mixture was cooled to RT and $SOCl_2$ was removed under reduced pressure (for 2 h). Then the crude mixture was placed under argon atmosphere and DCM (0.6 mL) was added. The mixture was cooled to 0 °C, triethylamine (0.18 mL, 1.25 mmol) was added followed by MeOH (0.63 mL). Then the mixture was stirred at RT for 4 h and quenched with the addition of a saturated solution of NH_4Cl (10 mL) and DCM (10mL). The phases were separated, and the aqueous phase was additionally extracted with DCM (1 x 10 mL). The combined organic phase was dried over anhydrous Na_2SO_4, concentrated, and purified with column chromatography on silica gel (25% DCM in hexane) to provide the product after removal of the solvent under reduced pressure as a colourless solid (0.065 g, 74%). m.p.73–74°C; IR (film): $\tilde{\nu}$=2964 (w), 1737 (s), 1624 (m), 1507 (s), 1360 (s), 1236 (s), 974 (m) cm^{-1}; ^1H NMR (400 MHz, $CDCl_3$) δ 4.00 (s, 3H, CH_3); $^{13}C\{1H\}$ NMR (101 MHz, $CDCl_3$) δ 163.1, 147.9 (dddd, J = 245.6, 11.2, 4.1, 1.8 Hz), 145.2 (dddd, J = 256.2, 11.7, 4.0, 2.1 Hz), 142.4 – 139.5 (m), 142.0 – 139.2 (m), 124.0 (dd, J = 17.1, 4.2 Hz), 74.6 (ddd, J = 26.7, 4.7, 2.8 Hz), 53.7; ^{19}F NMR (376 MHz, $CDCl_3$) δ -112.84 (ddd, J = 22.7, 10.5, 4.9 Hz), -136.73 (ddd, J = 21.0, 10.5, 4.9 Hz), -149.52 (ddd, J = 22.7, 19.6, 4.9 Hz), -152.19 (ddd, J = 21.0, 19.6, 4.9 Hz); GC-MS (EI) m/z: 334, 303, 148, 98.

2,3,4,5,6-pentafluoro-*N*-methylbenzamide – 13

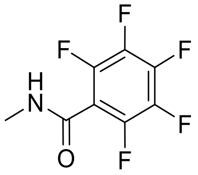

Methylamine hydrochloride (0.20 g, 3.00 mmol) was suspended in DCM (5.0 mL) and cooled 0 °C. Then triethylamine (0.56 mL, 4.00 mmol) and 2,3,4,5,6-pentafluorobenzoyl chloride (0.14 mL, 1.00 mmol) were added. The mixture was stirred at RT for 4.5 h and quenched with the addition of water (10 mL) and DCM (10mL). The phases were separated, and the aqueous phase was additionally extracted with DCM (1 x 10 mL). The combined organic phase was phased through a phase separator and purified with column chromatography on silica gel (2% EtOAc in DCM) to provide the product after removal of the solvent under reduced pressure as a colourless solid (0.12 g, 53%). m.p. 100–102°C; IR (film): $\tilde{\nu}$=3275 (m), 3113 (w), 1661 (s), 1500 (s), 1194 (m), 992 (s), 902 (w) cm^{-1}; ^1H NMR (400 MHz, $CDCl_3$) δ 6.41 (s, 1H; NH), 3.00 (d, J = 4.9 Hz, 3H, CH_3); $^{13}C\{^1H\}$ NMR (101 MHz, $CDCl_3$) δ 158.2, 144.2 (dddd, J = 252.5, 16.1, 7.9, 4.1 Hz), 143.8 – 140.7 (m), 139.1 – 136.1 (m), 111.6 (tdd, J = 20.4, 3.6, 1.7 Hz), 27.0; ^{19}F NMR (376 MHz, $CDCl_3$) δ -140.67 – -140.81 (m), -150.91 (tt, J = 20.7, 3.0 Hz), -160.17 – -160.35 (m); HRMS (ESI): m/z calcd for $C_8H_5F_5NO^+$: 226.0286 $[M+H]^+$; found: 226.0287.

10 Synthetic procedures for Michael reaction substrates

10.1 Genera procedure B

Based on modifications to the literature procedure:[22] Under argon atmosphere, $TiCl_4$ (0.13 mL, 1.20 mmol) and Et_3N (0.39 mL, 2.80 mmol) in CH_2Cl_2 (3.5 mL) were successively added to a stirred solution of triethyl phosphonoacetate **5** (0.20 mL 1.00 mmol) in DCM (3.5 mL) at 0 °C. After 15 min, the corresponding aldehyde (1.20 mmol) was added and the reaction mixture was stirred for 2 h. Then, a saturated solution of NH_4Cl (10 mL) was added to the mixture, which was extracted once with DCM (10 mL). The combined organic phase was dried over anhydrous Na_2SO_4, concentrated, and purified with column chromatography on silica gel (starting from 10% of EtOAc in CH_2Cl_2) to provide the product after removal of the solvent under reduced pressure.

10.2 Genera procedure C

Based on minor modifications to the literature procedure:[23] Under argon atmosphere, triethyl phosphonoacetate **5** (1.60 mL 8.00 mmol), the corresponding aldehyde (1.13 mmol), piperidine (0.48 mL, 4.80 mmol) and acetic acid (0.07 mL, 1.20 mmol) were successively added to a stirred solution of toluene (40 mL) in a three-necked round bottom flask connected to a Dean-Stark trap with activated 3 Å molecular sieves. The mixture was refluxed for 3 days in an oil bath. After completion of the reaction the solvent was removed under reduced pressure and the crude product was purified with column chromatography on silica gel to provide the product after removal of the solvent under reduced pressure.

ethyl (*E*)-2-(diethoxyphosphoryl)-3-(3-methylphenyl)acrylate – 1a (CAS nr. 107846-67-3)

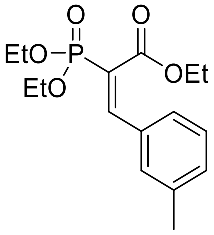

Genera procedure C on an 8 mmol scale. Column chromatography: starting from 30% of EtOAc in PE. Obtained as a yellow oil (0.90 g, 35%). IR (film): $\tilde{\nu}$= 1722 (s), 1615 (m), 1292 (s), 1256 (s), 1022 (s), 973 (s) cm^{-1}; ^1H NMR (400 MHz, $CDCl_3$) δ 7.62 (d, J = 24.2 Hz, 1H; =CH), 7.26 – 7.17 (m, 4H; Ar-H), 4.28 (q, J = 7.1 Hz, 2H; CH_2), 4.24 – 4.14 (m, 4H; CH_2), 2.34 (s, 3H; CH_3), 1.41 – 1.34 (m, 6H; CH_3), 1.25 (t, J = 7.1 Hz, 3H; CH_3); $^{13}C\{^1H\}$ NMR (101 MHz, $CDCl_3$) δ 166.6 (d, J = 12.7 Hz), 148.4 (d, J = 6.3 Hz), 138.3, 133.6 (d, J = 20.0 Hz), 131.2, 129.9, 128.6, 126.3, 124.2 (d, J = 178.8 Hz), 62.7 (d, J = 5.1 Hz), 61.7, 21.3, 16.2 (d, J = 6.7 Hz), 13.9.

1a was also prepared according to general procedure B on a 3.0 mmol scale with a reaction time of 3 h (0.88 g, 90%). Column chromatography: starting from 10% of EtOAc in DCM.

ethyl (*Z*)-2-(diethoxyphosphoryl)-3-(3-methylphenyl)acrylate – *Z*-1a

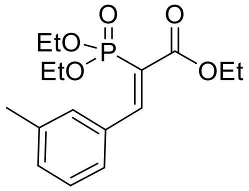

Obtained as a side product in the synthesis of **1a** (General procedure B) as a yellow oil (~0.05 g, impure). IR (film): $\tilde{\nu}$=2983 (m), 1716 (s), 1599 (w), 1228 (s), 1025 (s) cm^{-1}; ^1H NMR (400 MHz, $CDCl_3$) δ 8.17 (d, J = 43.8 Hz, 1H, =CH), 7.46 – 7.41 (m, 2H, Ar-H), 7.28 (t, J = 7.5 Hz, 1H, Ar-H), 7.20 (d, J = 7.6 Hz, 1H, Ar-H), 4.33 (q, J = 7.1 Hz, 2H, CH_2), 4.10 – 3.90 (m, 4H, CH_2), 2.37 (s, 3H, CH_3), 1.38 (t, J = 7.1 Hz, 3H, CH_3), 1.11 (td, J = 7.0, 0.4 Hz, 6H, CH_3); $^{13}C\{1H\}$ NMR (101 MHz, $CDCl_3$) δ 166.5 (d, J = 15.3 Hz), 154.8 (d, J = 4.7 Hz), 137.6, 134.0 (d, J = 6.5 Hz), 130.9, 130.7 (d, J = 1.5 Hz), 127.9, 127.3, 124.2 (d, J = 188.5 Hz), 62.5 (d, J = 6.2 Hz), 61.8, 16.0 (d, J = 6.7 Hz), 14.2

ethyl (*E*)-2-(diethoxyphosphoryl)-3-phenylacrylate – 1b (CAS nr. 13507-49-8)

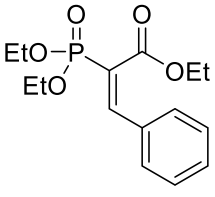

General procedure B on a 5.00 mmol scale. Obtained as a yellow oil (1.24 g, 79%). ^1H NMR (400 MHz, $CDCl_3$) δ 7.65 (d, J = 24.2 Hz, 1H; =C*H*), 7.46 – 7.34 (m, 5H; Ar-H), 4.28 (q, J = 7.1 Hz, 2H; CH_2), 4.25 – 4.14 (m, 4H; CH_2), 1.37 (td, J = 7.1, 0.5 Hz, 6H; CH_3), 1.25 (t, J = 7.1 Hz, 3H; CH_3); $^{13}C\{^1H\}$ NMR (101 MHz, $CDCl_3$) δ 166.5 (d, J = 12.6 Hz), 148.2 (d, J = 6.3 Hz), 133.7 (d, J = 20.2 Hz), 130.4, 129.2, 128.7, 124.5 (d, J = 178.7 Hz), 62.7 (d, J = 5.1 Hz), 61.7, 16.2 (d, J = 6.7 Hz), 13.9.

1b was also prepared according to general procedure C on a 4.5 mmol scale with a reaction time of 2 days (0.35 g, 25%).

ethyl (*Z*)-2-(diethoxyphosphoryl)-3-phenylacrylate – *Z*-1b (CAS nr. 1086271-77-3)

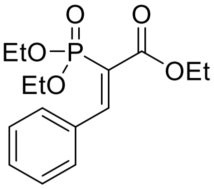

Obtained as a side product in the synthesis of **1b** (General procedure B) as a yellow oil (~0.07 g, impure). ^1H NMR (400 MHz, $CDCl_3$) δ 8.20 (d, J = 43.6 Hz, 1H; =CH), 7.66 – 7.60 (m, 2H; Ar-H), 7.42 – 7.36 (m, 3H; Ar-H), 4.34 (q, J = 7.1 Hz, 2H; CH_2), 4.08 – 3.91 (m, 4H; CH_2), 1.38 (t, J = 7.1 Hz, 3H; CH_3), 1.10 (t, J = 7.1 Hz, 6H; CH_3); $^{13}C\{^1H\}$ NMR 101 MHz, $CDCl_3$) δ 166.5 (d, J = 15.2 Hz), 154.5 (d, J = 4.5 Hz), 134.1 (d, J = 6.4 Hz), 130.1, 130.1, 128.0, 124.5 (d, J = 187.9 Hz), 62.5 (d, J = 6.2 Hz), 61.8, 16.0 (d, J = 6.9 Hz), 14.2; HRMS (ESI): *m*/*z* calcd for $C_{15}H_{22}O_5P^+$: 313.1199 $[M+H]^+$; found: 313.1196.

ethyl (*E*)-2-(diethoxyphosphoryl)-3-(4-methoxyphenyl)acrylate – 1c (CAS nr. 14656-25-8)

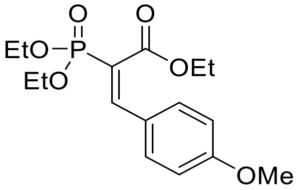

General procedure C on a 3.0 mmol scale, refluxed for 1 day instead of 3 days. Column chromatography: starting from 40% of EtOAc in PE. Obtained as a yellow oil (0.56 g, *E*/*Z* 97/3, 55%). ^{1}H NMR (400 MHz, $CDCl_3$) *δ* 7.58 (d, *J* = 24.4 Hz, 1H; =CH), 7.46 – 7.38 (m, 2H; Ar-H), 6.91 – 6.85 (m, 2H; Ar-H), 4.31 (q, *J* = 7.1 Hz, 2H; CH_2), 4.23 – 4.12 (m, 4H; CH_2), 3.83 (s, 3H; CH_3), 1.36 (td, *J* = 7.0, 0.5 Hz, 6H; CH_3), 1.29 (t, *J* = 7.1 Hz, 3H; CH_3); ^{13}C{^{1}H} NMR (101 MHz, $CDCl_3$) *δ* 166.8 (d, *J* = 12.6 Hz), 161.5, 148.0 (d, *J* = 6.7 Hz), 131.5 (d, *J* = 1.0 Hz), 126.1 (d, *J* = 20.6 Hz), 121.0 (d, *J* = 180.2 Hz), 114.1, 62.6 (d, *J* = 5.1 Hz), 61.6, 55.4, 16.2 (d, *J* = 6.8 Hz), 14.0.

ethyl (E)-2-(diethoxyphosphoryl)-3-(4-nitrophenyl)acrylate – 1d (CAS nr. 18896-71-4)

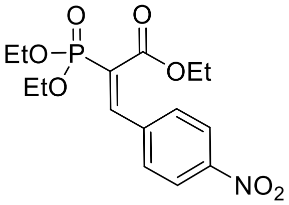

General procedure B on 1 mmol scale; stirred for 3 h instead of 2 h; extracted three times instead of once with DCM. Obtained as a yellow solid (0.18 g, 50%). ^{1}H NMR (400 MHz, $CDCl_3$) *δ* 8.27 – 8.18 (m, 2H; Ar-H), 7.70 (d, *J* = 23.7 Hz, 1H; =CH), 7.57 (d, *J* = 8.8 Hz, 2H; Ar-H), 4.31 – 4.16 (m, 6H; CH_2), 1.41 – 1.39 (td, *J* = 7.1, 0.4 Hz, 6H; CH_3), 1.25 (t, *J* = 7.1 Hz, 3H; CH_3); ^{13}C{^{1}H} NMR (101 MHz, $CDCl_3$) *δ* 165.4 (d, *J* = 11.8 Hz), 148.3, 145.3 (d, *J* = 6.5 Hz), 140.0 (d, *J* = 20.4 Hz), 129.7 (d, *J* = 1.3 Hz), 129.2 (d, *J* = 177.3 Hz), 123.8, 63.1 (d, *J* = 5.4 Hz), 62.1, 16.3 (d, *J* = 6.6 Hz), 13.9.

ethyl (*E*)-3-(2-chlorophenyl)-2-(diethoxyphosphoryl)acrylate – 1e (CAS nr. 53235-78-2)

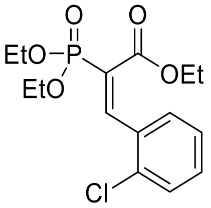

General procedure B on 1 mmol scale; extracted three times instead of once with DCM. Obtained as a yellow oil (0.25 g, 73%). ^{1}H NMR (400 MHz, $CDCl_3$) *δ* 7.91 (d, *J* = 23.3 Hz, 1H; =CH), 7.42 (dd, *J* = 7.9, 0.6 Hz, 1H; Ar-H), 7.37 (dd, *J* = 7.7, 1.2 Hz, 1H; Ar-H), 7.31 (td, *J* = 7.7, 1.7 Hz, 1H; Ar-H), 7.23 (ddd, *J* = 7.5, 1.2, 0.6 Hz, 1H; Ar-H), 4.28 – 4.14 (m, 6H; CH_2), 1.39 (t, *J* = 7.1 Hz, 6H; CH_3), 1.13 (t, *J* = 7.1 Hz, 3H; CH_3); ^{13}C{^{1}H} NMR (101 MHz, $CDCl_3$) *δ* 165.4 (d, *J* = 13.4 Hz), 146.1 (d, *J* = 7.4 Hz), 133.9, 133.0 (d, *J* = 20.0 Hz), 130.9, 129.7, 129.4 (d, *J* = 2.1 Hz), 127.6 (d, *J* = 179.3 Hz), 126.6, 63.0 (d, *J* = 5.4 Hz), 61.6, 16.3 (d, *J* = 6.6 Hz), 13.8.

ethyl (*E*)-3-(3-chlorophenyl)-2-(diethoxyphosphoryl)acrylate – 1f (CAS nr. 1512847-01-6)

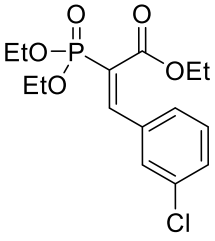

General procedure B on 1 mmol scale. Obtained as a pale-yellow oil (0.25 g, 73%). ^{1}H NMR (400 MHz, $CDCl_3$) *δ* 7.59 (d, *J* = 23.9 Hz, 1H; =CH), 7.41 (bs, 1H; Ar-H), 7.36 (dt, *J* = 7.1, 2.1 Hz, 1H; Ar-H), 7.34 – 7.28 (m, 2H; Ar-H), 4.29 (q, *J* = 7.1 Hz, 2H; CH_2), 4.25 – 4.14 (m, 4H; CH_2), 1.37 (td, *J* = 7.1, 0.5 Hz, 6H; CH_3), 1.26 (t, *J* = 7.1 Hz, 3H; CH_3); ^{13}C{^{1}H} NMR (101 MHz, $CDCl_3$) *δ* 166.0 (d, *J* = 12.2 Hz), 146.4 (d, *J* = 6.5 Hz), 135.5 (d, *J* = 20.4 Hz), 134.6, 130.2, 129.9, 128.8 (d, *J* = 1.3 Hz), 127.3 (d, *J* = 1.1 Hz), 126.4 (d, *J* = 178.1 Hz), 62.9 (d, *J* = 5.2 Hz), 61.9, 16.2 (d, *J* = 6.7 Hz), 13.9.

ethyl (*E*)-3-(4-chlorophenyl)-2-(diethoxyphosphoryl)acrylate – 1g (CAS nr. 13507-50-1)

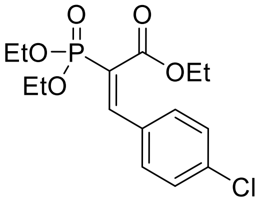

General procedure B on 1 mmol scale. Obtained as a yellow oil (0.24 g, 68%). ^{1}H NMR (400 MHz, $CDCl_3$) *δ* 7.59 (d, *J* = 24.1 Hz, 1H; =CH), 7.39 – 7.33 (m, 4H; Ar-H), 4.28 (q, *J* = 7.1 Hz, 2H; CH_2), 4.24 – 4.13 (m, 4H; CH_2), 1.37 (t, *J* = 7.1 Hz, 6H; CH_3), 1.26 (t, *J* = 7.1 Hz, 3H; CH_3); ^{13}C{^{1}H} NMR (101 MHz, $CDCl_3$) *δ* 166.2 (d, *J* = 12.3 Hz), 146.8 (d, *J* = 6.6 Hz), 136.5, 132.1 (d, *J* = 20.5 Hz), 130.5 (d, *J* = 1.2 Hz), 129.0, 125.22 (d, *J* = 178.6 Hz), 62.8 (d, *J* = 5.2 Hz), 61.8, 16.2 (d, *J* = 6.7 Hz), 13.9.

ethyl (*E*)-3-(2-bromophenyl)-2-(diethoxyphosphoryl)acrylate – 1h (CAS nr. 169602-34-0)

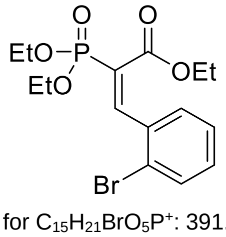

General procedure B on 2 mmol scale. Obtained as a yellow oil (0.59 g, 76%). IR (film): $\tilde{\nu}$=2984 (m), 1724 (s), 1615 (w), 1464 (m), 1340 (m), 1256 (s), 1215(s), 1024 (s) cm^{-1}; ^{1}H NMR (400 MHz, $CDCl_3$) *δ* 7.85 (d, *J* = 23.1 Hz, 1H, =CH), 7.61 (dd, *J* = 7.8, 1.3 Hz, 1H, Ar-H), 7.35 (dd, *J* = 7.6, 1.7 Hz, 1H, Ar-H), 7.28 (td, *J* = 7.5, 1.3 Hz, 1H, Ar-H), 7.23 (td, *J* = 7.6, 1.8 Hz, 1H, Ar-H), 4.29 – 4.19 (m, 4H, CH_2), 4.16 (q, *J* = 7.1 Hz, 2H, CH_2), 1.39 (t, *J* = 7.1 Hz, 6H, CH_3), 1.12 (t, *J* = 7.1 Hz, 3H, CH_3); ^{13}C{1H} NMR (101 MHz, $CDCl_3$) *δ* 165.3 (d, *J* = 13.4 Hz), 148.3 (d, *J* = 7.3 Hz), 135.0 (d, *J* = 20.0 Hz), 132.8, 130.9, 129.4 (d, *J* = 2.1 Hz), 127.6 (d, *J* = 179.1 Hz), 127.2, 123.6 (d, *J* = 1.1 Hz), 63.0 (d, *J* = 5.4 Hz), 61.6, 16.3 (d, *J* = 6.6 Hz), 13.8; HRMS (ESI): *m*/*z* calcd for $C_{15}H_{21}BrO_5P^+$: 391.0304 $[M+H]^+$; found: 391.0295.

ethyl (*Z*)-3-(2-bromophenyl)-2-(diethoxyphosphoryl)acrylate – *Z*-1h

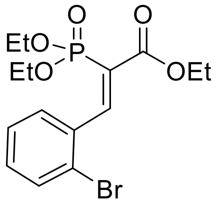

Obtained as a side product in the synthesis of **1h** (General procedure B) as a yellow oil (~0.08 g, impure). IR (film): $\tilde{\nu}$=2983 (w), 1721 (s), 1613 (w), 1465 (w), 1248 (s), 1205(m), 1025 (s) cm^{-1}; ^{1}H NMR (400 MHz, $CDCl_3$) *δ* 8.26 (d, *J* = 42.8 Hz, 1H, =CH), 7.64 (dd, *J* = 7.7, 1.2 Hz, 1H, Ar-H), 7.59 (dd, *J* = 8.0, 1.1 Hz, 1H, Ar-H), 7.37 – 7.32 (m, 1H, Ar-H), 7.27 – 7.22 (m, 1H, Ar-H), 4.36 (q, *J* = 7.1 Hz, 2H, CH_2), 4.08 – 3.89 (m, 4H, CH_2), 1.39 (t, *J* = 7.1 Hz, 3H, CH_3), 1.09 (td, *J* = 7.1, 0.5 Hz, 6H, CH_6); ^{13}C{1H} NMR (101 MHz, $CDCl_3$) *δ* 165.5 (d, *J* = 15.5 Hz), 153.5 (d, *J* = 3.5 Hz), 135.4 (d, *J* = 6.2 Hz), 132.0, 131.7 (d, *J* = 1.7 Hz), 130.9, 126.9, 126.2 (d, *J* = 187.7 Hz), 123.0 (d, *J* = 2.0 Hz), 62.7 (d, *J* = 6.3 Hz), 61.9, 16.0 (d, *J* = 6.8 Hz), 14.4;

ethyl (*E*)-2-(diethoxyphosphoryl)-3-(2-nitrophenyl)acrylate – 1i (CAS nr. 1855023-74-3)

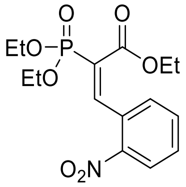

General procedure B on 1 mmol scale. Obtained as a yellow solid (0.20 g, 56%). ^{1}H NMR (400 MHz, $CDCl_3$) *δ* 8.27 – 8.16 (m, 2H, Ar-H, =CH), 7.67 – 7.62 (m, 1H, Ar-H), 7.56 (dddd, *J* = 8.2, 7.5, 1.5, 0.6 Hz, 1H, Ar-H), 7.38 – 7.35 (m, 1H, Ar-H), 4.35 – 4.19 (m, 4H, CH_2), 4.04 (q, *J* = 7.1 Hz, 2H, CH_2), 1.40 (td, *J* = 7.1, 0.5 Hz, 6H, CH_3), 1.01 (t, *J* = 7.1 Hz, 3H, CH_3); ^{13}C{1H} NMR (101 MHz, $CDCl_3$) *δ* 164.3 (d, *J* = 13.6 Hz), 149.5 (d, *J* = 8.1 Hz), 146.6, 133.7, 132.0 (d, *J* = 20.1 Hz), 129.9 (d, *J* = 2.4 Hz), 129.8, 126.9 (d, *J* = 180.4 Hz), 124.8, 63.1 (d, *J* = 5.4 Hz), 61.4, 16.3 (d, *J* = 6.5 Hz), 13.7; HRMS (ESI): *m/z* calcd for $C_{15}H_{21}NO_7P^+$: 358.1050 [*M*+H]$^+$; found: 358.1038.

ethyl (*E*)-2-(diethoxyphosphoryl)-3-(thiophen-2-yl)acrylate – 1j (CAS nr. 53235-84-0)

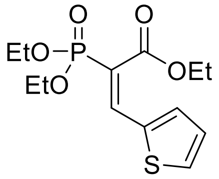

General procedure B on 1 mmol scale. Obtained as a yellow oil (0.19 g, *E*/*Z* 96/4, 59%). ^{1}H NMR (400 MHz, $CDCl_3$) *δ* 7.91 (d, *J* = 23.8 Hz, 1H; =CH), 7.58 (d, *J* = 5.0 Hz, 1H; Ar-H), 7.46 – 7.40 (m, 1H; Ar-H), 7.09 (dd, *J* = 5.1, 3.7 Hz, 1H; Ar-H), 4.37 (q, *J* = 7.1 Hz, 2H; CH_2), 4.24 – 4.08 (m, 4H; CH_2), 1.40 – 1.33 (m, 9H; CH_3); ^{13}C{^{1}H} NMR (101 MHz, $CDCl_3$) *δ* 165.7 (d, *J* = 13.5 Hz), 143.4 (d, *J* = 9.1 Hz), 136.8, 136.6, 133.1, 127.3, 117.7 (d, *J* = 183.0 Hz), 62.6 (d, *J* = 5.1 Hz), 61.7, 16.3 (d, *J* = 6.8 Hz), 14.1.

ethyl (*E*)-2-(diethoxyphosphoryl)-3-(naphthalen-2-yl)acrylate – 1k (CAS nr. 1512846-81-9)

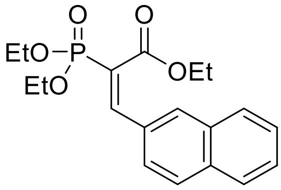

General procedure B on 1 mmol scale; stirred for 4 h instead of 2 h extracted two times instead of once with DCM. Column chromatography: starting from 8% of EtOAc in DCM. Obtained as a yellow oil (0.17 g, 48%). ^{1}H NMR (400 MHz, $CDCl_3$) *δ* 7.93 (bs, 1H; Ar-H), 7.85 – 7.81 (m, *J* = 7.6, 2.6 Hz, 3H; =CH and Ar-H), 7.79 (d, *J* = 7.5 Hz, 1H; Ar-H), 7.56 – 7.47 (m, 3H; Ar-H), 4.31 (q, *J* = 7.1 Hz, 2H; CH_2), 4.27 – 4.17 (m, 4H; CH_2), 1.39 (td, *J* = 7.1, 0.5 Hz, 6H; CH_3), 1.25 (t, *J* = 7.1 Hz, 3H; CH_3); ^{13}C{^{1}H} NMR *δ* 166.6 (d, *J* = 12.6 Hz), 148.3 (d, *J* = 6.4 Hz), 134.0, 133.0, 131.2 (d, *J* = 20.2 Hz), 130.5, 128.7, 128.3, 127.7, 127.6, 126.7, 125.3 (d, *J* = 1.0 Hz), 123.5, 62.8 (d, *J* = 5.1 Hz), 61.8, 16.3 (d, *J* = 6.7 Hz), 14.0.

ethyl (2*E*,4*E*)-2-(diethoxyphosphoryl)-5-phenylpenta-2,4-dienoate – 1l (CAS nr. 93939-12-9)

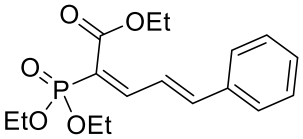

General procedure B on 1 mmol scale; extracted two times instead of once with DCM. Obtained as a yellow oil (0.15 g, 43%). ^{1}H NMR (400 MHz, $CDCl_3$) *δ* 7.84 (ddd, *J* = 15.3, 11.5, 2.0 Hz, 1H; =CH), 7.68 (dd, *J* = 21.5, 11.7 Hz, 1H; =CH), 7.57 – 7.51 (m, 2H; Ar-H), 7.44 – 7.33 (m, 3H; Ar-H), 7.09 (d, *J* = 15.4 Hz, 1H; =CH), 4.34 (q, *J* = 7.1 Hz, 2H; CH_2), 4.24 – 4.05 (m, 4H; CH_2), 1.41 – 1.31 (m, 9H; CH_3); ^{13}C{^{1}H} NMR (101 MHz, $CDCl_3$) *δ* 164.8 (d, *J* = 13.6 Hz), 155.4 (d, *J* = 7.6 Hz), 146.5, 135.6 (d, *J* = 1.5 Hz), 130.1, 128.9, 128.1, 124.3 (d, *J* = 19.4 Hz), 119.8 (d, *J* = 186.0 Hz), 62.5 (d, *J* = 5.5 Hz), 61.1, 16.4 (d, *J* = 6.5 Hz), 14.3.

ethyl (*Z*)-2-(diethoxyphosphoryl)-4,4-dimethylpent-2-enoate – *Z*-1m

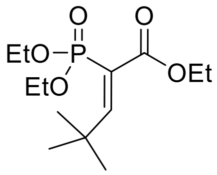

General procedure B on 3 mmol scale; stirred for 6 h instead of 2 h. Column chromatography: 18% of EtOAc in PE. Obtained as an off-white oil (0.16 g, 18%). IR (KBr): $\tilde{\nu}$= 1720 (s), 1601 (w), 1365 (m), 1255 (s), 1164 (w), 1029 (s) cm^{-1}; ^{1}H NMR (400 MHz, $CDCl_3$) *δ* 7.21 (d, *J* = 48.1 Hz, 1H; =CH), 4.23 (q, *J* = 7.1 Hz, 2H; CH_2), 4.20 – 4.07 (m, 4H; CH_2), 1.35 – 1.29 (m, 18H; CH_3); ^{13}C{^{1}H} NMR (101 MHz, $CDCl_3$) *δ* 169.2 (d, *J* = 7.4 Hz), 167.7 (d, *J* = 15.4 Hz), 124.5 (d, *J* = 187.2 Hz), 62.3 (d, *J* = 6.1 Hz), 61.6, 34.8 (d, *J* = 5.3 Hz), 29.9 (d, *J* = 1.0 Hz), 16.3 (d, *J* = 6.5 Hz), 14.1; HRMS (ESI): *m/z* calcd for $C_{13}H_{26}O_5P^+$: 293.1512 [*M*+H]$^+$; found: 293.1515.

ethyl (*E*)-2-(diethoxyphosphoryl)-4,4-dimethylpent-2-enoate – 1m (CAS nr. 53235-74-8)

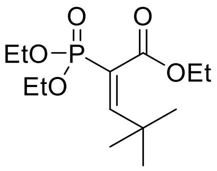

Obtained with ***Z*-1j** according to general procedure B on 3 mmol scale; stirred for 6 h instead of 2 h. Column chromatography: 18% of EtOAc in PE and after the elution of ***Z*-1j** the polarity was changed to 25% of EtOAc in PE to elute **1j**. Obtained as an off-white oil (0.32 g, 37%). IR (film): $\tilde{\nu}$=2982 (m), 1726 (s), 1662 (w), 1254 (m), 1198 (m), 1026 (s) cm^{-1}; ^1H NMR (400 MHz, $CDCl_3$) δ 6.72 (d, J = 26.4 Hz, 1H, =CH), 4.25 (q, J = 7.1 Hz, 2H, CH_2), 4.17 – 4.05 (m, 4H, CH_2), 1.36 – 1.30 (m, 9H, CH_3), 1.15 (s, 9H, CH_3); $^{13}C\{1H\}$ NMR (101 MHz, $CDCl_3$) δ 166.7 (d, J = 13.2 Hz), 160.5 (d, J = 3.7 Hz), 122.9 (d, J = 174.6 Hz), 62.5 (d, J = 5.1 Hz), 61.4, 35.7 (d, J = 17.1 Hz), 28.9 (d, J = 1.6 Hz), 16.2 (d, J = 6.8 Hz), 14.0; HRMS (ESI): m/z calcd for $C_{13}H_{26}O_5P^+$: 293.1512 $[M+H]^+$; found: 293.1506.

ethyl (*E*)-2-(diethoxyphosphoryl)-3-(1*H*-pyrrol-2-yl)acrylate – 1n

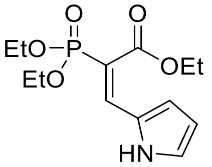

General procedure B on 1 mmol scale. Column chromatography: starting from 20% of EtOAc in DCM. Obtained as a beige solid (0.10 g, 33%). m.p. 43–45°C; IR (KBr): $\tilde{\nu}$= 1686 (m), 1574 (s), 1216 (s), 1087 (s), 1041 (s) cm^{-1}; ^1H NMR (400 MHz, $CDCl_3$) δ 12.20 (bs, 1H; NH_2), 7.90 (d, J = 23.6 Hz, 1H; =CH), 7.15 (ddd, J = 3.7, 2.4, 0.7 Hz, 1H; Ar-H), 6.81 (dt, J = 3.7, 1.8 Hz, 1H; Ar-H), 6.36 (dt, J = 3.7, 2.4 Hz, 1H; Ar-H), 4.32 (q, J = 7.1 Hz, 2H; CH_2), 4.21 – 4.03 (m, 4H; CH_2), 1.39 – 1.31 (m, 9H; CH_3); $^{13}C\{^1H\}$ NMR (101 MHz, $CDCl_3$) δ 167.7 (d, J = 12.8 Hz), 146.0 (d, J = 10.5 Hz), 128.4 (d, J = 20.8 Hz), 126.6, 124.9, 111.7, 62.1, 106.5 (d, J = 188.3 Hz), 62.1 (d, J = 5.4 Hz), 61.2, 16.4 (d, J = 6.7 Hz), 14.4; HRMS (ESI): m/z calcd for $C_{13}H_{20}NO_5P^+$: 302.1152 $[M+H]^+$; found: 302.1149.

diethyl (2-oxo-2*H*-chromen-3-yl)phosphonate – 1o (CAS nr. 104855-51-8)

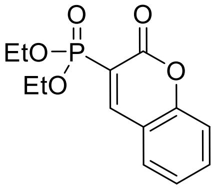

General procedure B on 1 mmol scale; stirred for 6 h total instead of 2h, additional amount of Et_3N (0.20 mL, 1.40 mmol) was added to the reaction mixture 1 h prior to quenching of the reaction; during the extraction the aqueous phase was acidified with an aqueous solution of HCl (5 mL, 2 M) prior to the separation of the phases; extracted two times with DCM. Column chromatography: starting from 25% of EtOAc in PE. Obtained as a pale-yellow oil that solidified in the fridge (0.11 g, 38%). ^1H NMR (400 MHz, $CDCl_3$) δ 8.53 (d, J = 17.2 Hz, 1H; =CH), 7.68 – 7.62 (m, 1H; Ar-H), 7.60 (dd, J = 7.7, 1.4 Hz, 1H; Ar-H), 7.40 – 7.32 (m, 2H; Ar-H), 4.37 – 4.18 (m, 4H; CH_2), 1.39 (t, J = 7.1 Hz, 6H; CH_3); $^{13}C\{^1H\}$ NMR (101 MHz, $CDCl_3$) δ 158.3 (d, J = 14.4 Hz), 155.3 (d, J = 1.0 Hz), 153.5 (d, J = 6.6 Hz), 134.3, 129.4, 124.9 (d, J = 0.9 Hz), 118.0 (d, J = 14.2 Hz), 117.9 (d, J = 196.4 Hz), 116.9 (d, J = 0.9 Hz), 63.4 (d, J = 6.0 Hz), 16.4 (d, J = 6.3 Hz).

ethyl 2-ethoxybenzo[e][1,2]oxaphosphinine-3-carboxylate 2-oxide – 1p (CAS nr. 173107-32-9)

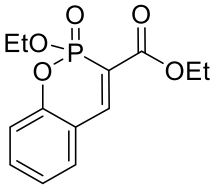

See procedure for **1m**, as **1n** was obtained as a side product. Obtained as a pale-yellow oil that solidified in the fridge (0.079 g, 28%). ^1H NMR (400 MHz, $CDCl_3$) δ 8.25 (d, J = 37.0 Hz, 1H; =CH), 7.52 – 7.45 (m, 2H; Ar-H), 7.21 (ddd, J = 11.2, 6.1, 2.3 Hz, 2H; Ar-H), 4.50 – 4.30 (m, 4H; CH_2), 1.45 – 1.37 (m, 6H; CH_3); $^{13}C\{^1H\}$ NMR (101 MHz, $CDCl_3$) δ 163.8 (d, J = 12.9 Hz), 152.7 (d, J = 8.8 Hz), 150.5 (d, J = 3.6 Hz), 133.7, 131.6 (d, J = 1.5 Hz), 124.2, 119.6 (d, J = 15.9 Hz), 118.8 (d, J = 7.5 Hz), 118.4 (d, J = 177.3 Hz), 64.9 (d, J = 6.4 Hz), 62.0, 16.5 (d, J = 6.4 Hz), 14.2.

diethyl (*E*)-styrylphosphonate – 6 (CAS nr. 20408-33-7)

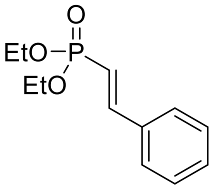

Triethyl phosphonoacetate **5** (0.64 mL, 2.50 mmol), benzaldehyde (0.31 mL, 3.00 mmol) and NaOH (0.21 g, 5.25 mmol) were ground together in a mortar for 10 minutes. The crude mixture was transferred to a separatory funnel with DCM (40 mL) and a saturated aqueous solution of NH_4Cl (30 mL). The phases were separated, and the aqueous phase was additionally extracted with DCM (2 x 50 mL). The combined organic phase was dried over anhydrous $MgSO_4$, concentrated, and purified with column chromatography on silica gel (starting from 20% of EtOAc in DCM) to provide the desired product. Obtained as a colourless oil (0.49 g, 82%). ^1H NMR (400 MHz, $CDCl_3$) δ 7.58 – 7.45 (m, 3H; =CH and Ar-H), 7.43 – 7.35 (m, 3H; Ar-H), 6.26 (t, J = 17.6 Hz, 1H; =CH), 4.21 – 4.08 (m, 4H; CH_2), 1.36 (dt, J = 7.1, 3.5 Hz, 6H; CH_3); $^{13}C\{^1H\}$ NMR (101 MHz, $CDCl_3$) δ 148.8 (d, J = 6.7 Hz), 134.9 (d, J = 23.2 Hz), 130.3, 128.9, 127.7, 114.0 (d, J = 191.3 Hz), 61.8 (d, J = 5.4 Hz), 16.4 (d, J = 6.5 Hz).

11 Synthetic procedures for asymmetric XB-assisted Michael reactions

11.1General procedure D

Phosphonate **1** (0.200 mmol) and malononitrile **2** (26.4 mg, 0.400 mmol) were weighed into the reaction vessel and then dissolved in toluene (1.0 mL). The reaction vessel was cooled to 0 °C and then catalyst **B** (12.5 mg, 0.020 mmol) was added. The progress of the reaction was monitored by TLC. The reaction was stirred for the stated amount of time, after which an NMR sample was taken to determine the d.r. and the reaction was quenched by purification with column chromatography on silica gel (10% of EtOAc in DCM) to provide the desired product as a mixture of diastereoisomers (d.r. determined by ^{1}H NMR spectroscopy of the isolated product).

11.2 General procedure E

Used during the optimisation: Phosphonate **1a** (32.6 mg, 0.100 mmol), 1,3,5-trimethoxybenzene (8.5 mg, 0.050 mmol) and malononitrile **2** (13.2 mg, 0.200 mmol) were weighed into the reaction vessel and then dissolved in the solvent. At the stated temperature the catalyst was added. The progress of the reaction was monitored by TLC. The reaction was stirred for the stated amount of time, after which an NMR sample was taken to determine the d.r. and the reaction was quenched by purification with column chromatography on silica gel (5% of EtOAc in DCM) to provide the desired product as a mixture of diastereoisomers.

ethyl (2*R,3*S**)-4,4-dicyano-2-(diethoxyphosphoryl)-3-(m-tolyl)butanoate – 3a**

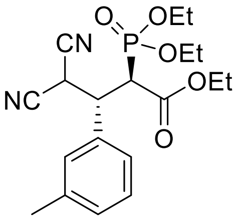

General procedure D on a 1.00 mmol scale, the temperature was not constant and varied between 0–4 °C. Reaction time: 42 h. Obtained as a yellow oil (0.33 g, 84%, 86:14 d.r., 74%/76% *ee*). *ee* determined by HPLC analysis on a chiral stationary phase; for major diastereoisomer: Chiralpak AD-H, hexane/*i*PrOH 9:1, 1 mL/min, 25 °C, 210 nm, t_R = 9.6 (major), 11.5 (minor) min; for minor diastereoisomer: Chiralpak AD-H, hexane/*i*PrOH 7:3, 0.8 mL/min, 35 °C, 210 nm, t_R = 11.2 (minor), 18.8 (major) min; IR (film): $\tilde{\nu}$= 1737 (s), 1254 (s), 1153 (m), 1050 (s), 1021 (s) cm^{-1}; ^{1}H NMR (400 MHz, $CDCl_3$) (major diastereoisomer) *δ* 7.34 – 7.27 (m, 1H; Ar-H), 7.23 – 7.16 (m, 3H; Ar-H), 5.11 (d, *J* = 3.8 Hz, 1H; CH), 4.33 – 4.19 (m, 4H; CH_2), 3.98 – 3.90 (m, 2H; CH_2), 3.82 (ddd, *J* = 12.4, 8.5, 4.0 Hz, 1H; CH), 3.71 (dd, *J* = 18.9, 12.3 Hz, 1H; CH), 2.37 (s, 3H; CH_3), 1.41 (dt, *J* = 13.1, 7.2 Hz, 6H; CH_3), 0.94 (t, *J* = 7.1 Hz, 3H; CH_3).; ^{13}C{^{1}H} NMR (101 MHz, $CDCl_3$) (major diastereoisomer) *δ* 165.9 (d, *J* = 6.2 Hz), 138.8, 134.1 (d, *J* = 15.5 Hz), 130.28, 129.1, 128.93, 125.3, 111.6, 111.2, 64.1 (d, *J* = 7.0 Hz), 64.0 (d, *J* = 6.5 Hz), 61.9, 47.3 (s, *J* = 126.6 Hz), 44.1 (d, *J* = 4.0 Hz), 28.5, 21.4, 16.4 (d, *J* = 1.2 Hz), 16.3 (d, *J* = 1.2 Hz), 13.6; ^{1}H NMR (400 MHz, $CDCl_3$) (minor diastereoisomer) *δ* 7.26 – 7.24 (m, 3H, Ar-H), 7.23 – 7.16 (m, 1H; Ar-H), 4.49 (dd, *J* = 5.0, 0.9 Hz, 1H; CH), 4.40 – 4.34 (m, 1H; CH_2), 4.20 – 4.15 (m, 1H; CH_2), 3.98 – 3.84 (m, 4H; CH, CH_2), 3.73 – 3.59 (m, 2H; CH, CH_2), 2.39 (s, 3H; CH_3), 1.38 – 1.33 (m, 3H; CH_3), 1.14 (dt, *J* = 7.0 Hz, 6H; CH_3); ^{13}C{^{1}H} NMR (101 MHz, $CDCl_3$) (minor diastereoisomer) *δ* 167.8 (d, *J* = 6.1 Hz), 138.7, 133.8 (d, *J* = 2.7 Hz), 130.3, 129.5, 128.9, 126.0, 111.3, 111.1, 63.2 (d, *J* = 6.8 Hz), 63.1 (d, *J* = 7.0 Hz), 62.7, 48.4 (d, *J* = 132.1 Hz), 44.2, 28.3, 21.5, 16.2 (d, *J* = 6.2 Hz), 16.0 (d, *J* = 6.3 Hz), 14.0; HRMS (ESI): *m*/*z* calcd for $C_{19}H_{26}N_2O_5P^+$: 393.1574 [*M*+H]$^+$; found: 393.1581.

ethyl (2*R,3*S**)-4,4-dicyano-2-(diethoxyphosphoryl)-3-phenylbutanoate – 3b**

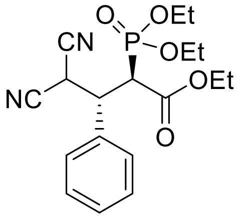

General procedure D. Reaction time: 28 h. Obtained as an amorphous yellow solid (0.061 g, 80%, 87:13 d.r., 75%/79% *ee*). *ee* determined by HPLC analysis on a chiral stationary phase; for major diastereoisomer: Chiralpak AD-H, hexane/*i*PrOH 95:5, 1 mL/min, 25 °C, 210 nm, t_R = 23.6 (major), 26.3 (minor) min; for minor diastereoisomer: Chiralpak AD-H, hexane/*i*PrOH 7:3, 0.8 mL/min, 35 °C, 210 nm, t_R = 12.5 (major), 22.7 (minor) min; IR (film): $\tilde{\nu}$= 1738 (s), 1254 (s), 1155 (m), 1050 (s), 1022 (s) cm^{-1}; ^{1}H NMR (400 MHz, $CDCl_3$) (major diastereoisomer) *δ* 7.44 – 7.28 (m, 5H; Ar-H), 5.06 (d, *J* = 4.0 Hz, 1H; CH), 4.26 – 4.12 (m, 4H; CH_2) 3.85 (q, *J* = 7.0 Hz, 2H; CH_2), 3.82 – 3.75 (m, 1H; CH), 3.66 (dd, *J* = 19.3, 12.3 Hz, 1H; CH), 1.34 (dt, *J* = 13.9, 7.1 Hz, 6H; CH_3), 1.07 0.86 (t, *J* = 7.1 Hz, 3H; CH_3); ^{13}C{^{1}H} NMR (major diastereoisomer) *δ* 165.9 (d, *J* = 6.2 Hz), 134.3 (d, *J* = 15.6 Hz), 129.5, 129.1, 128.4, 111.5, 111.2, 64.0 (d, *J* = 6.6 Hz), 63.3 (d, *J* = 6.6 Hz), 62.0, 48.4 (d, *J* = 132.1 Hz), 28.5, 16.4 (d, *J* = 1.3 Hz), 16.3 (d, *J* = 1.5 Hz), 13.6; ^{1}H NMR (400 MHz, $CDCl_3$) (minor diastereoisomer) *δ* 7.44 – 7.28 (m, 5H; Ar-H), 4.43 (dd, *J* = 5.0, 0.9 Hz, 1H; CH), 4.35 – 4.24 (m, 2H; CH_2),), 3.95 – 3.89 (m, 1H; CH), 3.88 – 3.76 (m, 3H; CH and CH_2), 3.68 – 3.53 (m, 2H; CH_2), 1.31 – 1.26 (m, 3H; CH_3), (td, *J* = 7.1, 2.8 Hz, 6H; CH_3), ^{13}C{^{1}H} NMR (101 MHz, $CDCl_3$) (minor diastereoisomer) *δ* 167.8 (d, *J* = 6.1 Hz), 133.9 (d, *J* = 2.7 Hz), 129.6, 129.1, 129.0, 111.3, 111.0, 64.2 (d, *J* = 7.0 Hz), 63.1 (d, *J* = 7.1 Hz), 62.8, 47.9 (d, *J* = 126.7 Hz), 44.4 (d, *J* = 1.4 Hz), 44.2 (d, *J* = 3.9 Hz), 28.3, 16.2 (d, *J* = 5.9 Hz), 16.1 (d, *J* = 6.4 Hz), 14.04; HRMS (ESI): *m*/*z* calcd for $C_{18}H_{24}N_2O_5P^+$: 379.1417 [*M*+H]$^+$; found: 379.1410.

ethyl (2*R,3*S**)-4,4-dicyano-2-(diethoxyphosphoryl)-3-(4-methoxyphenyl)butanoate – 3c**

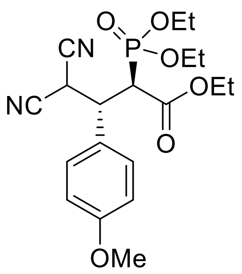

General procedure D. Reaction time: 48 h. Obtained as an amorphous yellow solid (0.060 g, 73%, 89:11 d.r., 76%/77% *ee*). *ee* determined by HPLC analysis on a chiral stationary phase; for major diastereoisomer: Chiralpak AD-H, hexane/*i*PrOH 92:8, 1 mL/min, 25 °C, 210 nm, t_R = 20.3 (minor), 22.8 (major) min; for minor diastereoisomer: Chiralpak AD-H, hexane/*i*PrOH 7:3, 0.8 mL/min, 35 °C, 210 nm, t_R = 14.2 (minor), 25.3 (major) min; IR (film): $\tilde{\nu}$= 1737 (s),1612 (m), 1516 (s), 1258 (s), 1179 (m), 1024 (s), ^{1}H NMR (400 MHz, $CDCl_3$) (major diastereoisomer) δ 7.36 – 7.30 (m, 2H; Ar-H), 6.94 – 6.89 (m, 2H; Ar-H), 5.06 (d, *J* = 3.9 Hz, 1H; CH), 4.31 – 4.19 (m, 4H; CH_2), 3.94 (q, *J* = 7.2 Hz, 2H; CH_2), 3.87 – 3.79 (m, 1H; CH), 3.81 (s, 3H; CH_3), 3.68 (dd, *J* = 19.2, 12.3 Hz, 1H; CH), 1.41 (dt, *J* = 12.6, 7.1 Hz, 6H; CH_3), 0.98 (t, *J* = 7.1 Hz, 3H; CH_3). $^{13}C\{^1H\}$ NMR (major diastereoisomer) δ 165.9 (d, *J* = 6.1 Hz), 160.3, 126.1 (d, *J* = 15.9 Hz), 129.6, 114.4, 111.7, 111.3, 64.1 (d, *J* = 7.0 Hz), 64.0 (d, *J* = 6.6 Hz), 61.9, 55.3, 48.1 (d, *J* = 126.4 Hz), 43.6 (d, *J* = 4.0 Hz), 16.4 (d, *J* = 1.2 Hz), 16.3 (d, *J* = 1.4 Hz), 13.7; ^{1}H NMR (400 MHz, $CDCl_3$) (minor diastereoisomer) δ 7.42 – 7.38 (m, 2H; Ar-H), 6.97 – 6.93 (m, 2H; Ar-H), 4.44 (dd, *J* = 4.9, 0.8 Hz, 1H; CH), 4.39 – 4.31 (m, 2H; CH_2), 3.98 – 3.90 (m, 3H; CH and CH_2), 3.89 – 3.84 (m, 1H; CH), 3.82 (s, 3H; CH_3), 3.77 – 3.68 (m, 2H; CH_2), 1.37 – 1.34 (m, 3H; CH_3), 1.16 (td, *J* = 7.1, 3.8 Hz, 6H; CH_3); $^{13}C\{^1H\}$ NMR (101 MHz, $CDCl_3$) (minor diastereoisomer) δ 160.4, 130.2, δ 126.0 (d, *J* = 14.1 Hz), 114.3, 63.3 (d, *J* = 6.8 Hz), 63.1 (d, *J* = 7.7 Hz), 62.7, 55.3, 48.5 (d, *J* = 132.4 Hz), 43.8 (d, *J* = 1.3 Hz), 28.8, 28.6, 16.2 (d, *J* = 6.0 Hz), 16.1 (d, *J* = 6.5 Hz).14.0 (the *C*O and *C*N carbon atoms were not detected due to the low intensity of the signals); m^{-1}; HRMS (ESI): *m*/*z* calcd for $C_{19}H_{25}N_2O_6P+Na^+$: 431.1342 [*M*+Na]$^+$; found: 431.1347.

ethyl (2*R,3*S**)-4,4-dicyano-2-(diethoxyphosphoryl)-3-(4-nitrophenyl)butanoate – 3d**

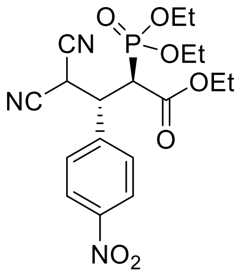

General procedure D. Reaction time: 140 min. Obtained as an off-white solid (0.077 g, 91%, 83:17 d.r., 71%/73% *ee*). *ee* determined by HPLC analysis on a chiral stationary phase; for major diastereoisomer: Chiralpak AD-H, hexane/*i*PrOH 9:1, 1 mL/min, 25 °C, 254 nm, t_R = 18.8 (minor), 23.9 (major) min; for minor diastereoisomer: Chiralpak AD-H, hexane/*i*PrOH 6:4, 0.8 mL/min, 25 °C, 254 nm, t_R = 18.3 (minor), 30.9 (major) min; IR (KBr): $\tilde{\nu}$= 1739 (s), 1609 (w), 1528 (s), 1351 (s), 1231 (m), 1150 (m), 1050 (s), 1017 (s), m^{-1}; ^{1}H NMR (400 MHz, $CDCl_3$) (major diastereoisomer) δ 8.31 – 8.27 (m, 2H; Ar-H), 7.66 – 7.59 (m, 2H; Ar-H), 5.30 (d, *J* = 4.2 Hz, 1H; CH), 4.33 – 4.20 (m, 4H; CH_2), 4.04 – 3.93 (m, 3H; CH and CH_2), 3.72 (dd, *J* = 20.4, 12.2 Hz, 1H; CH), 1.48 – 1.38 (m, 6H; CH_3), 1.02 (t, *J* = 7.1 Hz, 3H; CH_3). $^{13}C\{^1H\}$ NMR (101 MHz, $CDCl_3$) (major diastereoisomer) δ 165.5 (d, *J* = 6.7 Hz), 148.6, 141.44 (d, *J* = 15.8 Hz), 129.5, 124.3, 111.1, 110.7, 64.32 (d, *J* = 6.6 Hz), 63.61 (d, *J* = 6.6 Hz), 62.4, 47.5 (d, *J* = 126.7 Hz), 43.8 (d, *J* = 3.4 Hz), 28.0, 16.4 (d, *J* = 1.4 Hz), 16.3 (d, *J* = 1.5 Hz), 13.8; ^{1}H NMR (400 MHz, $CDCl_3$) (minor diastereoisomer) δ 8.34 – 8.30 (m, 2H; Ar-H), 7.74 – 7.68 (m, 2H; Ar-H), 4.62 (dd, *J* = 5.5, 0.9 Hz, 1H; CH), 4.40 – 4.32 (m, 2H; CH_2), 4.11 (ddd, *J* = 11.9, 10.4, 5.5 Hz, 1H; CH), 4.02 – 3.93 (m, 2H; CH_2), 3.93 – 3.84 (m, 2H; CH_2), 3.80 – 3.71 (m, 1H; CH), 1.38 – 1.34 (m, 3H; CH_3), 1.18 (dt, *J* = 11.9, 7.1 Hz, 6H; CH_3); $^{13}C\{^1H\}$ NMR (101 MHz, $CDCl_3$) (minor diastereoisomer) δ 167.2 (d, *J* = 6.5 Hz), 148.6 (based on HMBC), 140.85 (d, *J* = 2.5 Hz), 130.3, 124.00, 110.8, 110.6, 64.4 (d, *J* = 7.1 Hz), 63.5 (d, *J* = 7.0 Hz), 63.1, 47.9 (d, *J* = 131.2 Hz), 44.1 (d, *J* = 1.6 Hz), 27.8, 16.2 (d, *J* = 5.9 Hz), 16.1 (d, *J* = 6.2 Hz), 14.0; HRMS (ESI): *m*/*z* calcd for $C_{18}H_{23}N_3O_7P^+$: 424.1268 [*M*+H]$^+$; found: 424.1264.

ethyl (2*R,3*S**)-3-(2-chlorophenyl)-4,4-dicyano-2-(diethoxyphosphoryl)butanoate – 3e**

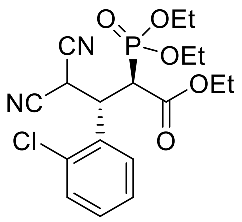

General procedure D. Reaction time: 44 h. Obtained as a colourless oil (0.076 g, 92%, 84:16 d.r., 89%/89% *ee*). *ee* determined by HPLC analysis on a chiral stationary phase; for major diastereoisomer: Chiralpak AD-H, hexane/*i*PrOH 8:2, 1 mL/min, 25 °C, 210 nm, t_R = 8.1 (major), 10.1 (minor) min; for minor diastereoisomer: Chiralpak AD-H, hexane/*i*PrOH 7:3, 0.8 mL/min, 35 °C, 210 nm, t_R = 12.5 (major), 22.7 (minor) min; IR (film): $\tilde{\nu}$= 1738 (m), 1245 (m), 1046 (s), 1020 (s), 772 (s) cm^{-1}; ^{1}H NMR (400 MHz, $CDCl_3$) (major diastereoisomer) δ 7.56 – 7.53 (m, 1H; Ar-H), 7.50 – 7.47 (m, 1H; Ar-H), 7.39 – 7.30 (m, 2H; Ar-H), 5.16 (d, *J* = 4.5 Hz, 1H; CH), 4.78 (ddd, *J* = 12.3, 10.4, 4.5 Hz, 1H; CH), 4.33 – 4.23 (m, 4H; CH_2), 3.92 (q, *J* = 7.1 Hz, 2H; CH_2), 3.70 (dd, *J* = 19.8, 12.3 Hz, 1H; CH), 1.42 (dtd, *J* = 7.6, 7.1, 0.4 Hz, 6H; CH_3), 0.96 (t, *J* = 7.1 Hz, 3H; CH_3); $^{13}C\{^1H\}$ NMR (major diastereoisomer) (101 MHz, $CDCl_3$) δ 165.44 (d, *J* = 6.7 Hz), 135.4, 132.74 (d, *J* = 16.0 Hz), 130.51, 130.4, 127.7, 127.5, 111.0 (d, *J* = 42.5 Hz), 64.3 (d, *J* = 5.7 Hz), 64.2 (d, *J* = 5.4 Hz), 62.1, 48.37 (d, *J* = 126.1 Hz), 38.1, 27.6, 16.4 (d, *J* = 3.3 Hz), 16.3 (d, *J* = 3.3 Hz), 14.0; ^{1}H NMR (400 MHz, $CDCl_3$) (minor diastereoisomer) δ 7.62 – 7.59 (m, 1H; Ar-H), 7.52 – 7.51 (m, 1H; Ar-H), 7.39 – 7.30 (m, 2H; Ar-H), 4.81 – 4.74 (m, 1H; CH), 4,51 (dd, *J* = 5.5 Hz, 0.6 Hz, 1H; CH), 4.42 – 4.33 (m, 2H; CH_2), 4.06 – 3.95 (m, 2H; CH_2), 3.94 – 3.85 (m, 2H; CH_2), 3.77 (dd, *J* = 22.5, 11.1 Hz, 1H; CH), 1.41 – 1.35 (m, 3H; CH_3), 1.24 (t, *J* = 7.0 Hz, 3H; CH_3), 1.12 (t, *J* = 7.1 Hz, 3H; CH_3); $^{13}C\{^1H\}$ NMR (101 MHz, $CDCl_3$) (minor diastereoisomer) δ 167.60 (d, *J* = 6.5 Hz), 135.2 (based on HMBC), 132.0 (d, *J* = 2.3 Hz), 130.5, 130.5, 129.0 (based on HMBC), 127.3, 110.7 (d, *J* = 33.3 Hz), 63.7 (d, *J* = 6.8 Hz), 63.1 (d, *J* = 7.1 Hz), 62.1, 48.16 (d, *J* = 131.2 Hz), 38.0, 27.7, 16.3 (d, *J* = 6.9 Hz), 16.0 (d, *J* = 6.2 Hz), 13.5; HRMS (ESI): *m*/*z* calcd for $C_{18}H_{23}ClN_2O_5P^+$: 413.1028 [*M*+H]$^+$; found: 413.1031.

ethyl (2*R,3*S**)-3-(3-chlorophenyl)-4,4-dicyano-2-(diethoxyphosphoryl)butanoate – 3f**

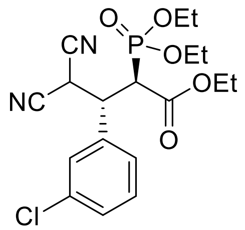

General procedure D. Reaction time: 24 h. Obtained as a yellow oil (0.076 g, 86%, 81:19 d.r., 74%/77% *ee*). *ee* determined by HPLC analysis on a chiral stationary phase; for major diastereoisomer: Chiralpak AD-H, hexane/*i*PrOH 95:5, 1 mL/min, 25 °C, 210 nm, t_R = 22.8 (major), 25.6 (minor) min; for minor diastereoisomer: Chiralcel AS-H, hexane/*i*PrOH 94:6, 1.0 mL/min, 25 °C, 210 nm, t_R = 24.3 (minor), 49.5 (major) min; IR (film): $\tilde{\nu}$= 1738 (s), 1598 (w), 1256 (s), 1157 (s), 1046 (s), 1022 (s), 796 (m) cm^{-1}; ^1H NMR (400 MHz, $CDCl_3$) (major diastereoisomer) *δ* 7.42 – 7.37 (m, 3H; Ar-H), 7.34 – 7.30 (m, 1H; Ar-H), 5.17 (d, *J* = 4.1 Hz, 1H; CH), 4.31 – 4.19 (m, 4H; CH_2), 4.01 – 3.94 (m, 2H; CH_2), 3.84 (ddd, *J* = 12.4, 8.9, 4.1 Hz, 1H; CH), 3.67 (dd, *J* = 19.6, 12.4 Hz, 1H; CH), 1.41 (dtd, *J* = 7.6, 7.1, 0.5 Hz, 6H; CH_3), 0.99 (t, *J* = 7.1 Hz, 3H; CH_3); $^{13}C\{^1H\}$ NMR (101 MHz, $CDCl_3$) (major diastereoisomer) *δ* 165.7 (d, *J* = 6.4 Hz), 136.3 (d, *J* = 15.8 Hz), 135.0, 130.4, 129.8, 128.6, 126.5, 111.3, 110.9, 64.3 (d, *J* = 7.0 Hz), 64.1 (d, *J* = 6.6 Hz), 62.1, 47.7 (d, *J* = 126.9 Hz), 43.8 (d, *J* = 3.7 Hz), 28.2, 16.4 (d, *J* = 1.4 Hz), 16.3 (d, *J* = 1.6 Hz), 13.7; ^1H NMR (400 MHz, $CDCl_3$) (minor diastereoisomer) *δ* 7.49 – 7.47 (m, 1H; Ar-H), 7.36 – 7.35 (m, 2H; Ar-H), 7.34 – 7.30 (m, 1H; Ar-H), 4.53 (dd, *J* = 5.3, 0.8 Hz, 1H; CH), 4.41 – 4.31 (m, 2H; CH_2), 3.97 – 3.89 (m, 4H; CH and CH_2), 3.83 – 3.76 (m, 1H; CH_2), 3.70 (dd, *J* = 22.5, 10.4 Hz, 1H; CH), 1.37 – 1.35 (m, 3H; CH_3), 1.17 (td, *J* = 7.5, 0.5 Hz, 6H; CH_3); $^{13}C\{^1H\}$ NMR (101 MHz, $CDCl_3$) (minor diastereoisomer) *δ* 167.49 (d, *J* = 6.1 Hz), 135.8 (based on HMBC), 134.8, 130.3, 129.81, 129.1, 127.3, 111.0, 110.8, 63.4 (d, *J* = 9.6 Hz), 63.3 (d, *J* = 6.9 Hz), 62.9, 48.2 (d, *J* = 131.8 Hz), 44.0 (d, *J* = 1.5 Hz), 28.0, 16.2 (d, *J* = 6.1 Hz), 16.1 (d, *J* = 6.1 Hz), 14.0, HRMS (ESI): *m/z* calcd for $C_{18}H_{23}ClN_2O_5P^+$: 413.1028 $[M+H]^+$; found: 413.1021.

ethyl (2*R,3*S**)-3-(4-chlorophenyl)-4,4-dicyano-2-(diethoxyphosphoryl)butanoate – 3g**

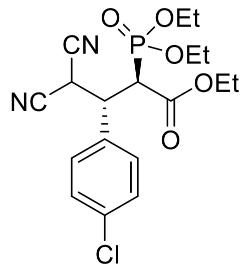

General procedure D. Reaction time: 24 h. Obtained as a pale-yellow oil (0.072 g, 87%, 79:21 d.r., 79%/80% *ee*). *ee* determined by HPLC analysis on a chiral stationary phase; for major diastereoisomer: Chiralpak AD-H, hexane/*i*PrOH 95:5, 1 mL/min, 25 °C, 210 nm, t_R = 21.1 (minor), 24.5 (major) min; for minor diastereoisomer: Chiralpak AD-H, hexane/*i*PrOH 7:3, 0.8 mL/min, 35 °C, 210 nm, t_R = 15.8 (minor), 25.4 (major) min; IR (film): $\tilde{\nu}$= 1737 (m), 1252 (m), 1046 (s), 1017 (s), 776 (w) cm^{-1}; ^1H NMR (400 MHz, $CDCl_3$) (major diastereoisomer) *δ* 7.41 – 7.38 (m, 2H; Ar-H), 7.38 – 7.34 (m, 2H; Ar-H), 5.15 (d, *J* = 4.3 Hz, 1H; CH), 4.30 – 4.18 (m, 4H; CH_2), 3.96 (q, *J* = 7.1 Hz, 2H; CH_2), 3.86 (ddd, *J* = 12.5, 9.1, 4.3 Hz, 1H; CH), 3.67 (dd, *J* = 19.7, 12.5 Hz, 1H; CH), 1.41 (dtd, *J* = 14.1, 7.1, 0.4 Hz, 6H; CH_3), 1.00 (t, *J* = 7.1 Hz, 3H; CH_3); $^{13}C\{^1H\}$ NMR (101 MHz, $CDCl_3$) (major diastereoisomer) *δ* 165.7 (d, *J* = 6.4 Hz), 135.69, 132.8 (d, *J* = 16.0 Hz), 129.7, 129.4, 111.4, 111.0, 64.3 (d, *J* = 7.0 Hz), 64.1 (d, *J* = 6.5 Hz), 62.1, 47.8 (d, *J* = 126.7 Hz), 43.7 (d, *J* = 3.8 Hz), 28.4, 16.4 (d, *J* = 1.5 Hz), 16.3 (d, *J* = 1.7 Hz), 13.7; ^1H NMR (400 MHz, $CDCl_3$) (minor diastereoisomer) *δ* 7.45 – 7.42 (m, 4H; Ar-H), 4.50 (dd, *J* = 5.2, 0.9 Hz, 1H; CH), 4.39 – 4.31 (m, 2H; CH_2), 4.01 – 3.89 (m, 4H; CH and CH_2), 3.82 – 3.75 (m, 1H; CH_2), 3.70 (dd, *J* = 22.5, 10.6 Hz, 1H; CH), 1.37 – 1.33 (m, 3H; CH_3), 1.17 (dtd, *J* = 7.1, 4.3, 0.4 Hz, 6H; CH_3); $^{13}C\{^1H\}$ NMR (101 MHz, $CDCl_3$) (minor diastereoisomer) *δ* 167.6 (d, *J* = 6.2 Hz), 135.73, 132.32 (d, *J* = 2.6 Hz), 130.4, 129.2, 111.1, 110.8, 63.4 (d, *J* = 6.7 Hz), 63.3 (d, *J* = 7.0 Hz), 62.9, 48.2 (d, *J* = 132.0 Hz), 43.9 (d, *J* = 1.4 Hz), 28.1, 16.2 (d, *J* = 6.0 Hz), 16.1 (d, *J* = 6.3 Hz), 14.0; HRMS (ESI): *m/z* calcd for $C_{18}H_{23}ClN_2O_5P^+$: 413.1028 $[M+H]^+$; found: 413.1019.

ethyl (2*S,3*S**)-3-(2-bromophenyl)-4,4-dicyano-2-(diethoxyphosphoryl)butanoate – 3h**

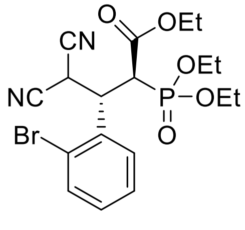

General procedure D. Reaction time: 20 h. Obtained as a colourless oil (0.081 g, 89%, 85:15 d.r., 91%/91% *ee*). *ee* determined by HPLC analysis on a chiral stationary phase; for major diastereoisomer: Chiralpak AD-H, hexane/*i*PrOH 7:3, 0.8 mL/min, 35 °C, 210 nm, t_R = 7.5 (major), 9.2 (minor) min; for minor diastereoisomer: Chiralpak AD-H, hexane/*i*PrOH 7:3, 0.8 mL/min, 35 °C, 210 nm, t_R = 11.0 (major), 23.7 (minor) min; IR (film): $\tilde{\nu}$=2985 (w), 1738 (s), 1473 (w), 1260 (m), 1021 (s), 977 (m) cm^{-1}; ^1H NMR (400 MHz, $CDCl_3$) (major diastereoisomer) *δ* 7.67 (dd, *J* = 8.0, 1.2 Hz, 1H, Ar-H), 7.54 (dd, *J* = 7.8, 1.5 Hz, 1H, Ar-H), 7.40 (td, *J* = 7.8, 1.2 Hz, 1H, Ar-H), 7.25 – 7.22 (m, 1H, Ar-H), 5.14 (d, *J* = 4.6 Hz, 1H, CH), 4.78 (ddd, *J* = 12.2, 10.6, 4.6 Hz, 1H, CH), 4.35 – 4.24 (m, 4H, CH_2), 3.96 – 3.87 (m, 2H, CH_2), 3.70 (dd, *J* = 19.8, 12.2 Hz, 1H, CH), 1.43 (qd, *J* = 7.0, 0.4 Hz, 6H, CH_3), 0.96 (t, *J* = 7.1 Hz, 3H, CH_3); $^{13}C\{^1H\}$ NMR (101 MHz, $CDCl_3$) (major diastereoisomer) *δ* 165.5 (d, *J* = 6.6 Hz), 134.4 (d, *J* = 16.0 Hz), 133.9, 130.7, 128.4, 127.6, 126.4, 111.2, 110.7, 64.3 (d, *J* = 3.9 Hz), 64.2 (d, *J* = 3.3 Hz), 62.1, 48.6 (d, *J* = 125.9 Hz), 40.7 (d, *J* = 2.2 Hz), 27.6, 16.4 (d, *J* = 4.1 Hz), 16.3 (d, *J* = 4.1 Hz), 13.6. ^1H NMR (400 MHz, $CDCl_3$) (minor diastereoisomer) *δ* 7.69 (dd, *J* = 7.6, 1.2 Hz, 1H, Ar-H), 7.59 (dd, *J* = 7.9, 1.5 Hz, 1H, Ar-H), 7.49 – 7.42 (m, 1H, Ar-H), 7.30 – 7.27 (m, 1H, Ar-H), 4.78 (ddd, *J* = 12.2, 10.6, 4.6 Hz, 2H, CH), 4.50 (d, *J* = 5.3 Hz, 1H, CH), 4.41 – 4.31 (m, 2H, CH_2), 4.25 – 4.14 (m, *J* = 21.9, 10.9, 5.3 Hz, 2H, CH_2), 4.13 – 3.96 (m, 2H, CH_2), 3.76 (dd, *J* = 22.5, 10.9 Hz, 1H, CH), 1.37 (t, *J* = 7.2 Hz, 3H, CH_3), 1.25 (t, *J* = 7.0 Hz, 3H, CH_3), 1.12 (t, *J* = 7.1 Hz, 3H, CH_3); $^{13}C\{^1H\}$ NMR ((101 MHz, $CDCl_3$) (minor diastereoisomer) *δ* 167.6 (d, *J* = 6.4 Hz), 134.4 (d, *J* = 16.0 Hz), 133.9, 130.7, 128.9 (based on HSQC), 127.6, 126.4, 110.9, 110.5, 63.7 (d, *J* = 6.6 Hz), 63.1 (d, *J* = 7.0 Hz), 62.9, 48.3 (d, *J* = 131.4 Hz), 40.7 (d, *J* = 2.2 Hz), 27.6 (s), 16.3 (d, *J* = 3.3 Hz), 16.0 (d, *J* = 6.1 Hz), 14.0; HRMS (ESI): *m/z* calcd for $C_{18}H_{23}BrN_2O_5P^+$: 457.0522 $[M+H]^+$; found: 457.0516.

ethyl (2*S,3*S**)-4,4-dicyano-2-(diethoxyphosphoryl)-3-(2-nitrophenyl)butanoate – 3i**

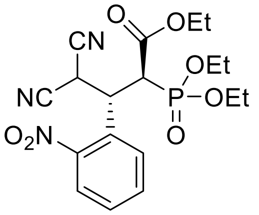

General procedure D on a 0.1 mmol scale. Reaction time: 24 h. Obtained as a pale-yellow oil (0.027 g, 63%, 74:26 d.r., 80%/79% *ee*). *ee* determined by HPLC analysis on a chiral stationary phase; for major diastereoisomer: Chiralpak OD-H, hexane/*i*PrOH 92:8, 1.0 mL/min, 25 °C, 210 nm, t_R = 16.0 (minor), 19.4 (major) min; for minor diastereoisomer: OD-H, hexane/*i*PrOH 92:8, 1.0 mL/min, 25 °C, 210 nm, t_R = 24.1 (minor), 30.1 (major) min; ; IR (film): $\tilde{\nu}$=2986 (w), 1736 (m), 1533 (s), 1351 (m), 1020 (s), 978 (m) cm^{-1}; ^1H NMR (400 MHz, $CDCl_3$) (major diastereoisomer) *δ* 8.05 (dd, *J* = 8.2, 1.2 Hz, 1H, Ar-H), 7.77 (dd, *J* = 7.9, 1.5 Hz, 1H, Ar-H), 7.72 (td, *J* = 7.6, 1.2 Hz, 1H, Ar-H), 7.59 (qd, *J* = 7.3, 1.4 Hz, 1H, Ar-H), 5.28 (d, *J* = 5.2 Hz, 1H, CH), 5.12 (ddd, *J* = 12.0, 11.3, 5.2 Hz, 1H, CH), 4.31 – 4.22 (m, 4H, CH_2), 3.96 – 3.86 (m, 2H, CH_2), 3.72 (dd, *J* = 20.4, 11.3 Hz, 1H, CH), 1.46 – 1.37 (m, 6H, CH_3), 0.95 (t, *J* = 7.1 Hz, 3H, CH_3); $^{13}C\{^1H\}$ NMR (101 MHz, $CDCl_3$) (major diastereoisomer) *δ* 165.4 (d, *J* = 7.2 Hz), 149.9; 133.9, 130.54 (d, *J* = 16.5 Hz), 130-2, 129.47 (d, *J* = 2.1 Hz), 127.7, 126.0, 111.5, 110.8, 64.36 (t, *J* = 6.8 Hz), 62.3, 48.76 (d, *J* = 125.5 Hz), 35.7, 27.6, 16.28 (d, *J* = 4.9 Hz), 13.6; ^1H NMR (400 MHz, $CDCl_3$) (minor diastereoisomer) *δ* 8.03 (dd, *J* = 8.3, 1.3 Hz, 1H, Ar-H), 7.84 (dd, *J* = 7.9, 1.1 Hz, 1H, Ar-H), 7.73 (td, *J* = 7.7, 1.4 Hz, 1H, Ar-H), 7.63 – 7.55 (m, 1H, Ar-H), 5.03 (td, *J* = 10.6, 6.2 Hz, 1H, CH), 4.68 (d, *J* = 6.1 Hz, 2H, CH), 4.41 – 4.31 (m, 2H, CH_2), 4.07 – 3.96 (m, 1H, CH_2), 3.87 – 3.79 (m, 1H, CH_2), 3.75 (dd, *J* = 21.9, 11.3 Hz, 1H, CH), 1.39 – 1.34 (m, 3H, CH_3), 1.23 (t, *J* = 7.1 Hz, 3H, CH_3), 1.05 (t, *J* = 7.1 Hz, 3H, CH_3); $^{13}C\{^1H\}$ NMR (101 MHz, $CDCl_3$) (minor diastereoisomer) *δ* 167.1 (d, *J* = 6.9 Hz), 150.2, 133.4, 130.5 (d, *J* = 16.5 Hz), 130.2, 129.9 (d, *J* = 12.1 Hz), 127.7, 125.7, 111.0, 110.5, 64.1 (d, *J* = 6.7 Hz), 63.2 (d, *J* = 7.1 Hz), 63.1, 48.7 (d, *J* = 130.2 Hz), 35.7, 27.5 (d, *J* = 17.1 Hz), 16.1 (d, *J* = 5.7 Hz), 15.9 (d, *J* = 6.3 Hz), 13.9; HRMS (ESI): *m*/*z* calcd for $C_{18}H_{23}N_3O_7P^+$: 424.1268 [*M*+H]$^+$; found: 424.1268.

ethyl (2*R,3*R**)-4,4-dicyano-2-(diethoxyphosphoryl)-3-(thiophen-2-yl)butanoate – 3j**

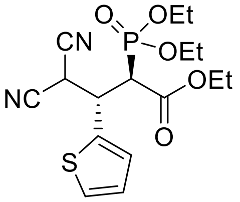

General procedure D. Reaction time: 24 h. Obtained as beige amorphous solid (0.057 g, 75%, 84:16 d.r., 72%/75% *ee*). *ee* determined by HPLC analysis on a chiral stationary phase; for major diastereoisomer: Chiralpak AD-H, hexane/*i*PrOH 95:5, 1 mL/min, 25 °C, 210 nm, t_R = 13.0 (minor), 21.3 (major) min; for minor diastereoisomer: Chiralpak AD-H, hexane/*i*PrOH 7:3, 0.8 mL/min, 35 °C, 210 nm, t_R = 11.3 (minor), 14.8 (major) min; IR (film): $\tilde{\nu}$= 1737 (s), 1258 (s), 1161 (m), 1020 (s) m^{-1}; ^1H NMR (400 MHz, $CDCl_3$) (major diastereoisomer) *δ* 7.34 (dd, *J* = 5.1, 0.8 Hz, 1H; Ar-H), 7.21 (dd, *J* = 3.6, 0.8 Hz, 1H; Ar-H), 7.03 (dd, *J* = 5.1, 3.6 Hz, 1H; Ar-H), 5.10 (d, *J* = 4.2 Hz, 1H; CH), 4.30 – 4.17 (m, 5H; CH and CH_2), 4.02 (qd, *J* = 7.1, 1.2 Hz, 2H; CH_2), 3.64 (dd, *J* = 19.4, 11.9 Hz, 1H; CH), 1.44 – 1.35 (m, 6H; CH_3), 1.06 (t, *J* = 7.1 Hz, 3H; CH_3); $^{13}C\{^1H\}$ NMR (101 MHz, $CDCl_3$) (major diastereoisomer) *δ* 165.7 (d, *J* = 6.1 Hz), 136.0 (d, *J* = 17.8 Hz), 128.0, 127.3, 126.6, 111.4, 111.1, 64.2 (d, *J* = 7.0 Hz), 64.1 (d, *J* = 6.6 Hz), 62.2, 49.5 (d, *J* = 126.1 Hz), 29.4, 16.3 (d, *J* = 6.1 Hz), 13.71; ^1H NMR (400 MHz, $CDCl_3$) (minor diastereoisomer) *δ* 7.38 (dd, *J* = 5.1, 0.8 Hz, 1H; Ar-H), 7.28 (dd, *J* = 3.6, 0.8 Hz, 1H; Ar-H), 7.06 (dd, *J* = 5.1, 3.6 Hz, 1H; Ar-H), 4.64 (dd, *J* = 5.3, 0.8 Hz, 1H; CH), 4.38 – 4.30 (m, 3H; CH and CH_2), 4.00 – 3.80 (m, 4H; CH_2), 3.68 (dd, *J* = 22.8, 9.6 Hz, 1H; CH), 1.44 – 1.35 (m, 3H; CH_3), 1.21 (dtd, *J* = 14.2, 7.1, 0.4 Hz, 6H; CH_3); $^{13}C\{^1H\}$ NMR (101 MHz, $CDCl_3$) (minor diastereoisomer) *δ* 167.4 (d, *J* = 5.9 Hz), 135.7 (d, *J* = 3.5 Hz), 128.6, 127.2, 126.8, 111.3, 110.9, 63.4 (d, *J* = 6.5 Hz), 63.3 (d, *J* = 6.8 Hz), 62.9, 49.6 (d, *J* = 132.3 Hz), 40.4 (d, *J* = 3.2 Hz), 40.2 (d, *J* = 2.5 Hz), 29.0, 16.3 (d, *J* = 7.1 Hz), 16.1 (d, *J* = 6.6 Hz), 14.0; HRMS (ESI): *m*/*z* calcd for $C_{16}H_{22}N_2O_5PS^+$: 385.0982 [*M*+ H]$^+$; found: 385.0982.

ethyl (2*R,3*S**)-4,4-dicyano-2-(diethoxyphosphoryl)-3-(naphthalen-2-yl)butanoate – 3k**

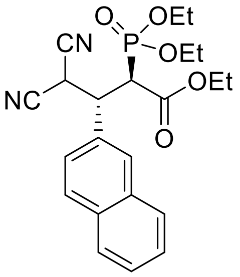

General procedure D. Reaction time: 17 h. Obtained as a colourless solid (0.079 g, 92%, 87:13 d.r., 76%/80% *ee*). *ee* determined by HPLC analysis on a chiral stationary phase; for major diastereoisomer: Chiralpak AD-H, hexane/*i*PrOH 95:5, 1 mL/min, 25 °C, 210 nm, t_R = 13.8 (minor), 22.1 (major) min; for minor diastereoisomer: Chiralcel AS-H, hexane/*i*PrOH 9:1, 1.0 mL/min, 25 °C, 230 nm, t_R = 19.5 (minor), 29.7 (major) min; IR (KBr): $\tilde{\nu}$= 1739 (s), 1254 (s), 1163 (m), 1046 (s), 1023 (s) m^{-1}; ^1H NMR (400 MHz, $CDCl_3$) (major diastereoisomer) *δ* 7.94 – 7.80 (m, 4H; Ar-H), 7.55 – 7.48 (m, 3H; Ar-H), 5.21 (d, *J* = 4.0 Hz, 1H; CH), 4.34 – 4.22 (m, 4H; CH_2), 4.05 (ddd, *J* = 12.8, 8.9, 4.0 Hz, 1H; CH), 3.93 – 3.82 (m, 3H; CH and CH_2), 1.47 – 1.38 (m, 6H; CH_3), 0.86 (t, *J* = 7.1 Hz, 3H; CH_3); $^{13}C\{^1H\}$ NMR (101 MHz, $CDCl_3$) (major diastereoisomer) *δ* 165.9 (d, *J* = 6.2 Hz), 133.55, 133.1, 131.7 (d, *J* = 15.7 Hz), 129.0, 128.32, 128.0, 127.7, 126.97, 126.7, 125.4, 111.5, 111.2, 64.2 (d, *J* = 7.0 Hz), 64.1 (d, *J* = 6.5 Hz), 62.0, 48.0 (d, *J* = 126.5 Hz), 44.3 (d, *J* = 3.9 Hz), 28.6, 16.3 (d, *J* = 0.7 Hz), 16.0 (d, *J* = 6.4 Hz), 13.6; ^1H NMR (400 MHz, $CDCl_3$) (minor diastereoisomer) *δ* 7.97 – 7.95 (m, 1H; Ar-H), 7.94 – 7.80 (m, 3H; Ar-H), 7.60 – 7.55 (m, 1H; Ar-H), 7.55 – 7.48 (m, 2H; Ar-H), 4.59 (dd, *J* = 5.1, 0.8 Hz, 1H; CH), 4.42 – 4.34 (m, 2H; CH_2), 4.17 (ddd, *J* = 16.0, 10.9, 5.1 Hz, 1H; CH), 3.93 – 3.82 (m, 3H; CH and CH_2), 3.82 – 3.76 (m, 1H; CH_2), 3.68 – 3.53 (m, 1H; CH_2), 1.41 – 1.36 (m, 3H; CH_3), 1.04 (dt, *J* = 13.3, 7.1 Hz, 6H; CH_3); $^{13}C\{^1H\}$ NMR (101 MHz, $CDCl_3$) (minor diastereoisomer) *δ* 167.8 (d, *J* = 6.2 Hz), 133.59, 133.1 (based on HMBC) , 131.23 (d, *J* = 2.6 Hz), 128.9, 128.8, 128.28, 128.0 (based on HMBC), 127.7 (based on HMBC), 127.00, 125.7, 111.3, 111.1, 63.2 (d, *J* = 6.7 Hz), 63.1 (d, *J* = 7.0 Hz), 62.8, 48.5 (d, *J* = 132.2 Hz), 44.5 (d, *J* = 1.4 Hz), 28.3, 16.4 (d, *J* = 0.6 Hz), 16.0 (d, *J* = 7.0 Hz), 14.0; HRMS (ESI): *m*/*z* calcd for $C_{22}H_{26}N_2O_5P^+$: 429.1574 [*M*+H]$^+$; found: 429.1579.

ethyl (2*R,3*R**,*E*)-3-(dicyanomethyl)-2-(diethoxyphosphoryl)-5-phenylpent-4-enoate – 3l**

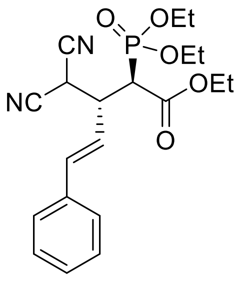

General procedure D. Reaction time: 46 h. Obtained as a yellow amorphous solid (0.050 g, 62%, 75:25 d.r., 79%/77% *ee*). *ee* determined by HPLC analysis on a chiral stationary phase; for major diastereoisomer: Chiralpak AD-H, hexane/*i*PrOH 95:5, 1 mL/min, 25 °C, 210 nm, t_R = 26.7 (minor), 38.7 (major) min; for minor diastereoisomer: Chiralpak AD-H, hexane/*i*PrOH 7:3, 0.8 mL/min, 35 °C, 210 nm, t_R = 11.3 (minor), 32.1 (major) min; IR (film): $\tilde{\nu}$= 1736 (s), 1256 (s), 1162 (m), 1023 (s) m^{-1}; ^{1}H NMR (400 MHz, $CDCl_3$) (major diastereoisomer) δ 7.38 – 7.20 (m, 5H; Ar-H), 6.71 (d, *J* = 15.6 Hz, 1H; =CH), 6.03 (dd, *J* = 15.6, 9.5 Hz, 1H; =CH), 4.78 (d, *J* = 4.3 Hz, 1H; CH), 4.19 – 4.11 (m, 4H; CH_2), 4.11 – 4.06 (m, 3H; CH and CH_2), 3.46 – 3.38 (m, 1H; CH), 3.26 (dd, *J* = 19.3, 10.6 Hz, 1H; CH), 1.31 (dt, *J* = 13.1, 7.1 Hz, 6H; CH_3), 1.13 (t, *J* = 7.1 Hz, 3H; CH_3); ^{13}C{^{1}H} NMR (101 MHz, $CDCl_3$) (major diastereoisomer) δ 166.2 (d, *J* = 5.7 Hz), 138.50 (d, *J* = 1.3 Hz), 135.1, 128.9, 128.7, 126.98, 120.8 (d, *J* = 14.8 Hz), 111.7, 111.0, 64.0 (d, *J* = 6.9 Hz), 63.9 (d, *J* = 6.5 Hz), 47.8 (d, *J* = 128.4 Hz), 62.3, 43.4 (d, *J* = 3.7 Hz), 16.4, 16.3, 14.1; ^{1}H NMR (400 MHz, $CDCl_3$) (minor diastereoisomer) δ 7.38 – 7.20 (m, 5H; Ar-H), 6.76 (d, *J* = 15.6 Hz, 1H; =CH), 6.10 (dd, *J* = 15.6, 9.8 Hz, 1H; =CH), 4.39 (d, *J* = 5.5 Hz, 1H; CH), 4.25 – 4.19 (m, 2H; CH_2), 4.06 – 4.00 (m, 4H; CH_2), 3.57 – 3.47 (m, 1H; CH), 3.33 (dd, *J* = 22.9, 7.8 Hz, 1H; CH), 1.26 (t, *J* = 7.2 Hz, 3H; CH_3), 1.24 (t, *J* = 7.1, 0.3 Hz, 3H; CH_3), 1.19 (dt, *J* = 7.1, 0.3 Hz, 3H; CH_3); ^{13}C{^{1}H} NMR (101 MHz, $CDCl_3$) (minor diastereoisomer) δ 167.0 (d, *J* = 5.5 Hz), 137.9, 135.4, 128.8, 128.7, 126.97, 121.10 (d, *J* = 4.6 Hz), 111.8, 111.0, 63.53 (d, *J* = 6.3 Hz), 63.46 (d, *J* = 6.9 Hz), 62.6, 47.56 (d, *J* = 133.8 Hz), 43.5 (d, *J* = 2.6 Hz), 27.57 (d, *J* = 11.9 Hz), 27.41 (d, *J* = 2.9 Hz), 16.32, 16.26, 14.1; HRMS (ESI): *m*/*z* calcd for $C_{20}H_{26}N_2O_5P^+$: 405.1574 [*M*+H]$^+$; found: 405.1563.

ethyl (2*R,3*S**)-3-(dicyanomethyl)-2-(diethoxyphosphoryl)-4,4-dimethylpentanoate – 3m**

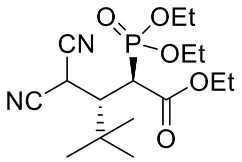

General procedure D. Reaction time: 7 days. Obtained as a yellow oil (0.011 g, 15%, >99:1 d.r., 87% *ee*). *ee* determined by HPLC analysis on a chiral stationary phase: Chiralcel AS-H, hexane/*i*PrOH 93:7, 1.0 mL/min, 25 °C, 210 nm, t_R = 7.7 (major), 9.9 (minor) min; $[\alpha]^D{}_{20}$=-28.0 (*c*=0.05 in $CHCl_3$); IR (film): $\tilde{\nu}$= 1733 (s), 1375 (m), 1264 (m), 1154 (m), 1046 (s), 1020 (s) m^{-1}; ^{1}H NMR (400 MHz, $CDCl_3$) δ 5.28 (t, *J* = 1.0 Hz, 1H; CH), 4.26 (dq, *J* = 10.8, 7.1 Hz, 1H; CH_2), 4.20 – 4.05 (m, 5H; CH_2), 3.20 (dd, *J* = 25.5, 1.0 Hz, 1H; CH), 2.40 (dt, *J* = 16.5, 1.1 Hz, 1H; CH), 1.33 – 1.23 (m, 9H; CH_3), 1.15 (s, 9H; CH_3); ^{13}C{^{1}H} NMR δ (101 MHz, $CDCl_3$) δ 167.5 (d, *J* = 3.9 Hz), 115.2, 113.3, 63.8 (d, *J* = 7.0 Hz), 63.7 (d, *J* = 6.9 Hz), 62.6, 48.1 (d, *J* = 5.1 Hz), 42.9 (d, *J* = 130.2 Hz), 36.2 (d, *J* = 12.6 Hz), 27.7, 20.9, 16.4 (d, *J* = 3.9 Hz), 16.3 (d, *J* = 4.0 Hz), 13.9; HRMS (ESI): *m*/*z* calcd for $C_{16}H_{28}N_2O_5P^+$: 359.1730 [*M*+ H]$^+$; found: 359.1722.

diethyl 2-(2,2-dicyano-1-phenylethyl)malonate – 7

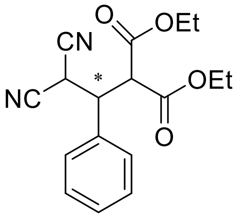

General procedure E. Reaction time: 48 h, the product was not isolated, a sample was taken for HPLC analysis after the stated time and purified by preparative TLC (R_f=0.18; DCM/EtOAc 2:0.3) (NMR yield 25%, 19% *ee*). *ee* determined by HPLC analysis on a chiral stationary phase: Chiralpak AD-H, hexane/*i*PrOH 9:1, 1 mL/min, 25 °C, 210 nm, t_R = 18.0 (major), 24.1 (minor) min; IR (film): $\tilde{\nu}$= 1732 (s), 1178 (m) m^{-1}; ^{1}H NMR (400 MHz, $CDCl_3$) δ 7.34 (s, 5H; Ar-H), 4.84 (d, *J* = 5.0 Hz, 1H; CH), 4.22 (qd, *J* = 7.1, 2.0 Hz, 2H; CH_2), 4.02 (d, *J* = 11.3 Hz, 1H; CH), 3.88 (qd, *J* = 7.1, 1.9 Hz, 2H; CH_2), 3.85 (dd, *J* = 11.3, 5.0 Hz, 1H; CH), 1.24 (t, *J* = 7.1 Hz, 3H; CH_3), 0.88 (t, *J* = 7.1 Hz, 3H; CH_3); ^{13}C{^{1}H} NMR (101 MHz, $CDCl_3$) δ 167.6, 166.0, 133.5, 129.8, 129.3, 128.7, 111.39, 111.36, 63.0, 62.3, 53.5, 45.0, 27.8, 14.0, 13.7; HRMS (ESI): *m*/*z* calcd for $C_{17}H_{19}N_2O_4^+$: 315.1339 [*M*+ H]$^+$; found: 315.1338.

12 NMR spectra

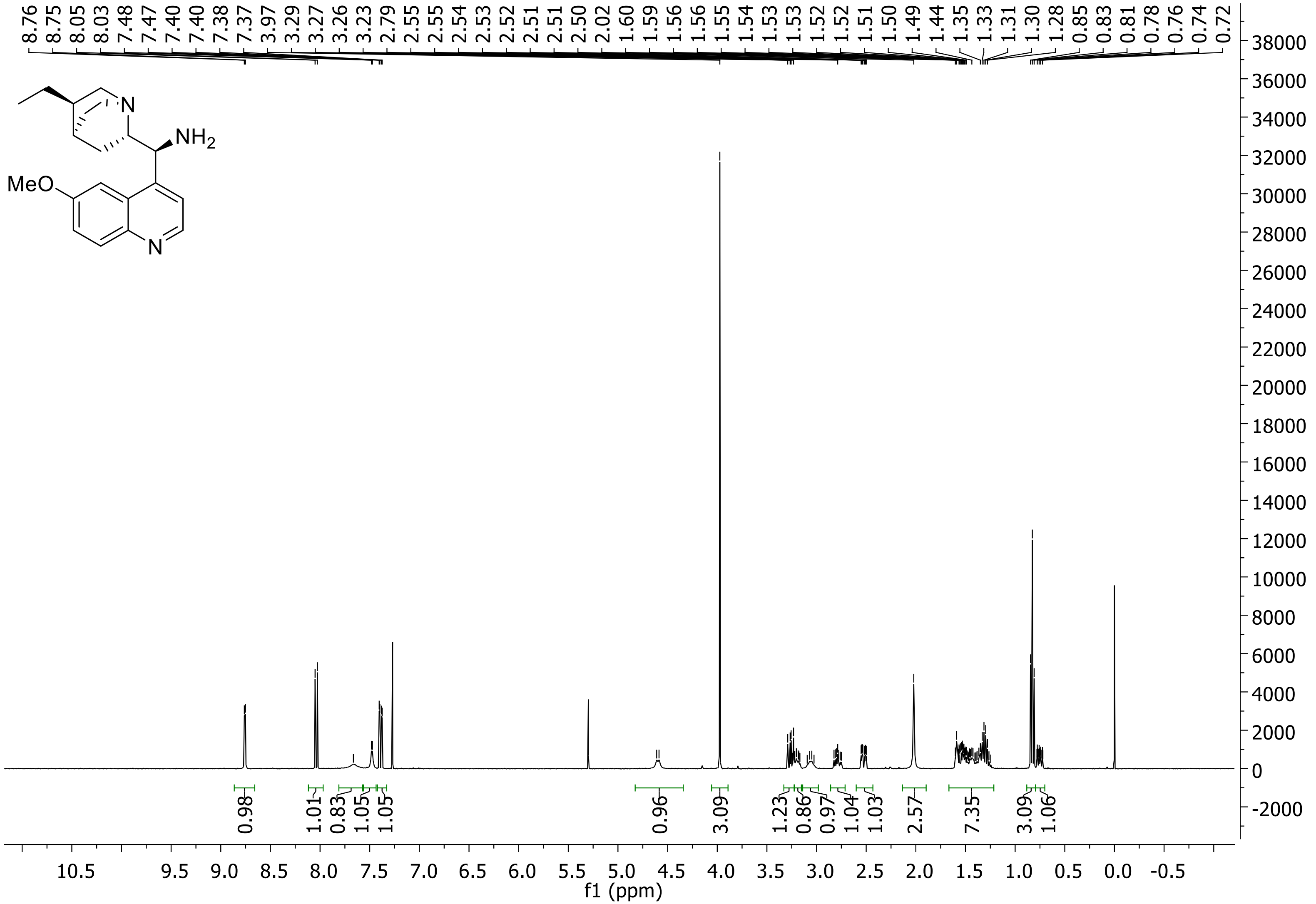

Figure S37. ^{1}H NMR spectrum of **(1*S*)-((2*S*,4*S*,5*R*)-5-ethylquinuclidin-2-yl)(6-methoxyquinolin-4-yl)methanamine** ($CDCl_3$, 400 MHz).

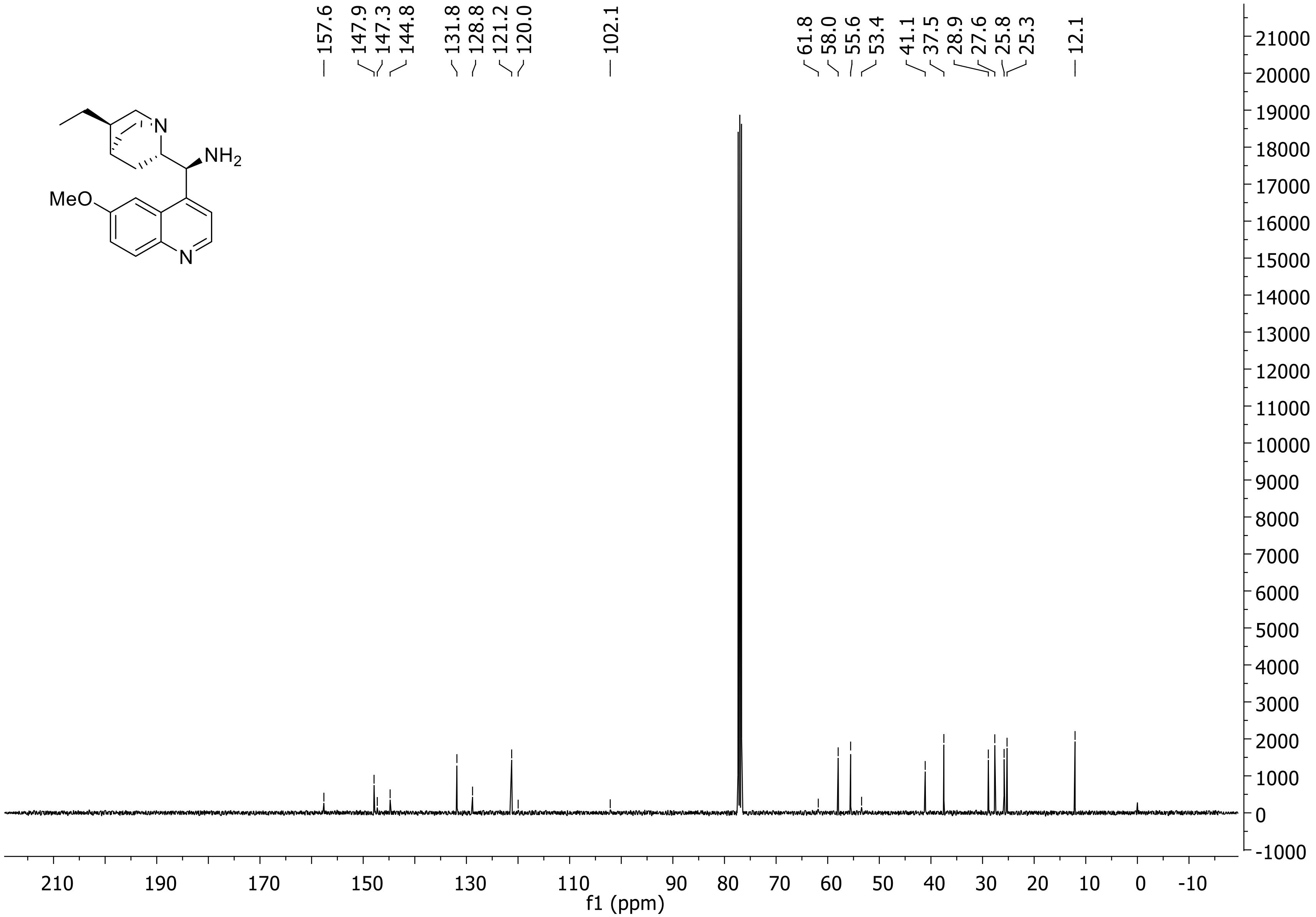

Figure S38. $^{13}C\{^{1}H\}$ NMR spectrum of **(1*S*)-((2*S*,4*S*,5*R*)-5-ethylquinuclidin-2-yl)(6-methoxyquinolin-4-yl)methanamine** ($CDCl_3$, 101 MHz).

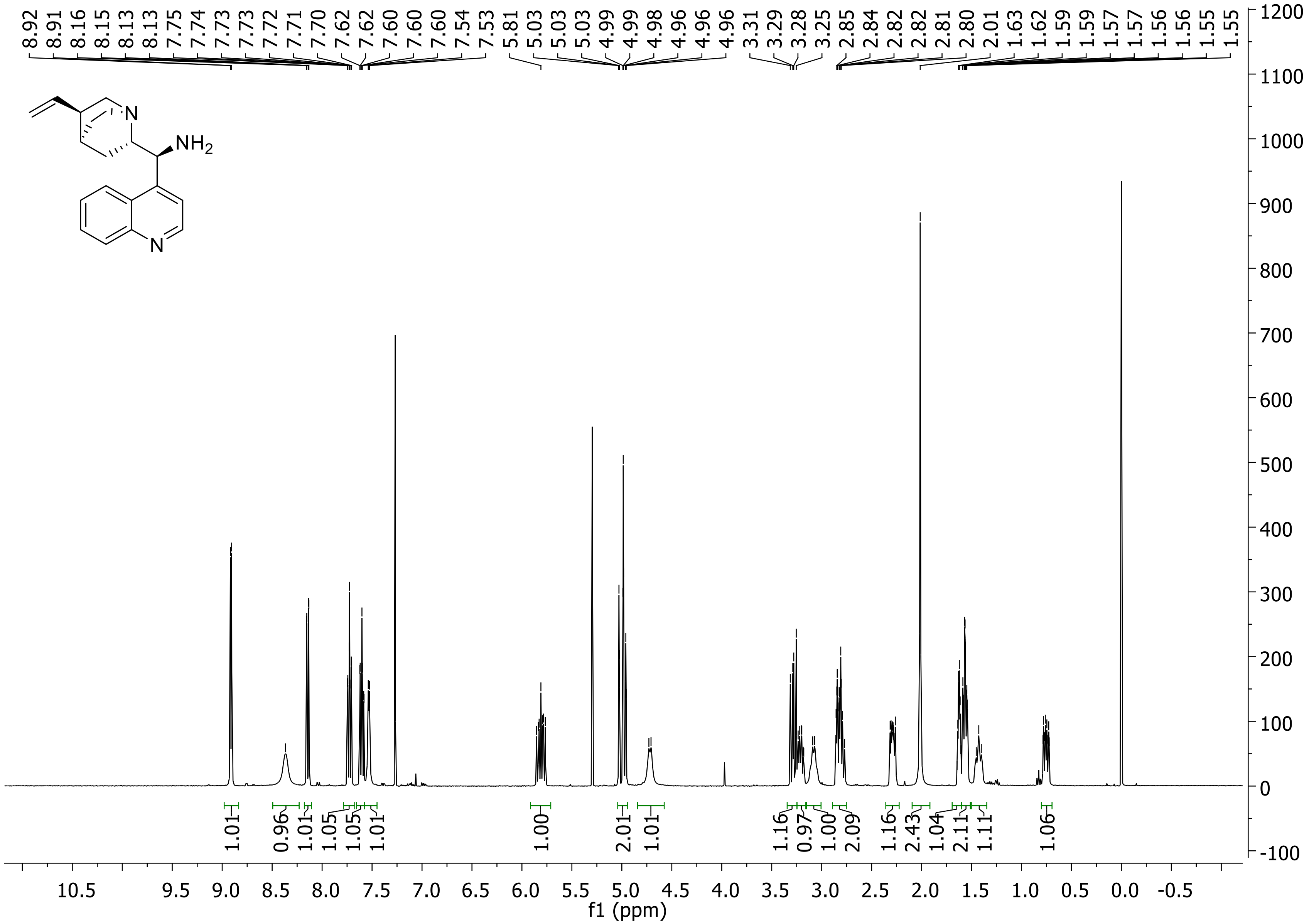

Figure S39. ^{1}H NMR spectrum of **(1*S*)-quinolin-4-yl((2*S*,4*S*,5*R*)-5-vinylquinuclidin-2-yl)methanamine** ($CDCl_3$, 400 MHz).

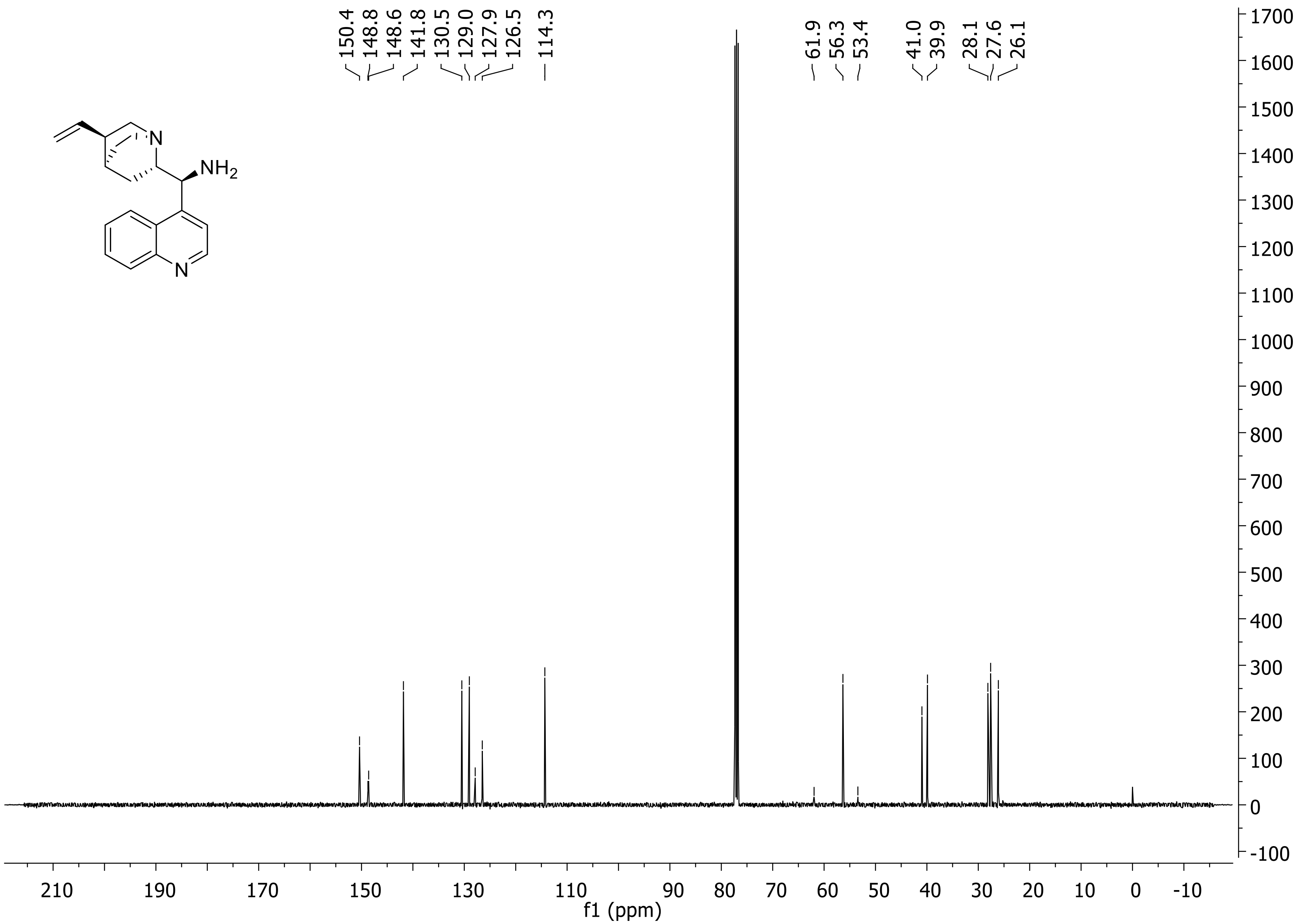

Figure S40. $^{13}C\{^{1}H\}$ NMR spectrum of **(1*S*)-quinolin-4-yl((2*S*,4*S*,5*R*)-5-vinylquinuclidin-2-yl)methanamine** ($CDCl_3$, 101 MHz).

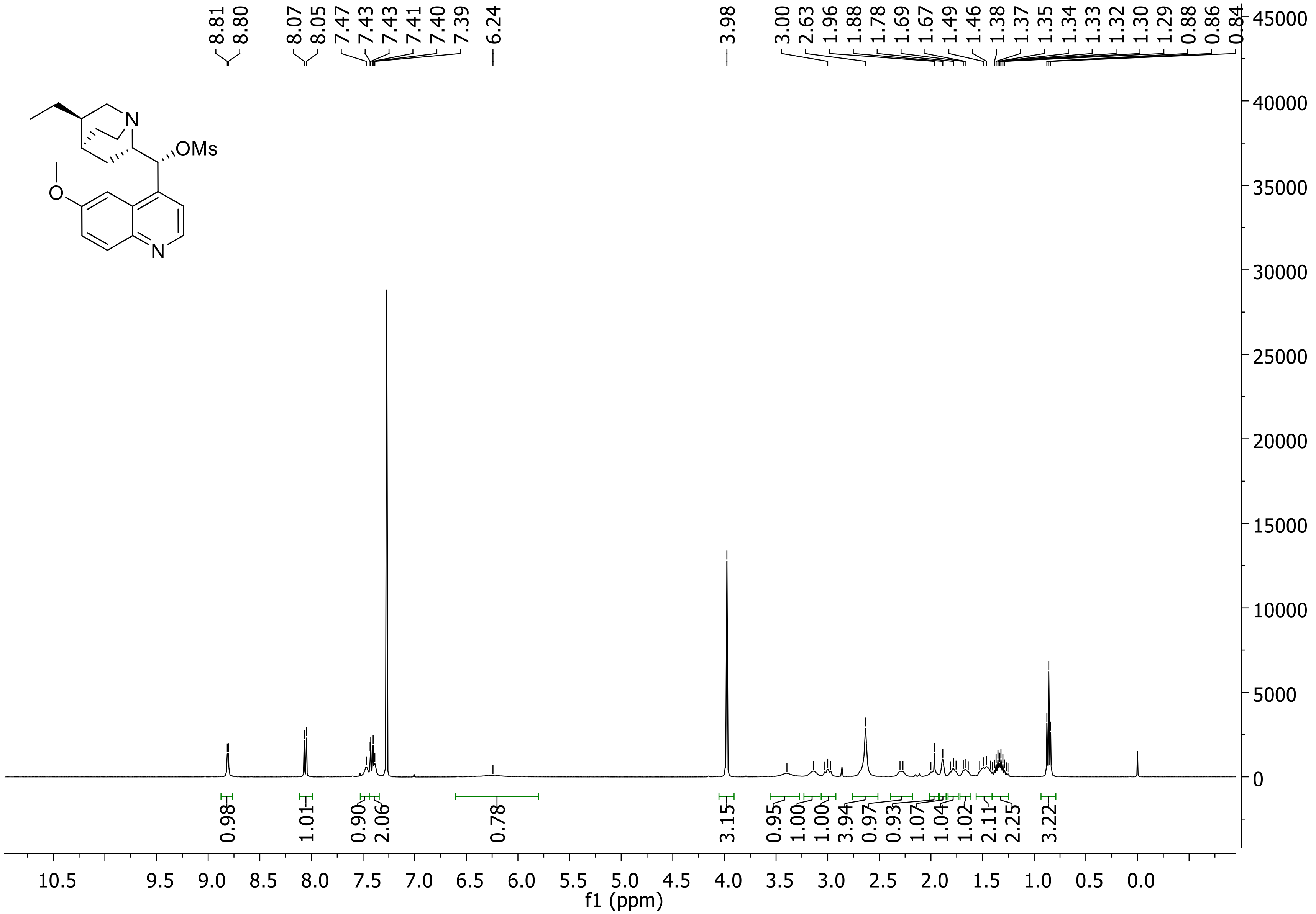

Figure S41. ^{1}H NMR spectrum of **(1*R*)-((2*S*,4*S*,5*R*)-5-ethylquinuclidin-2-yl)(6-methoxyquinolin-4-yl)methyl methanesulfonate** ($CDCl_3$, 400 MHz).

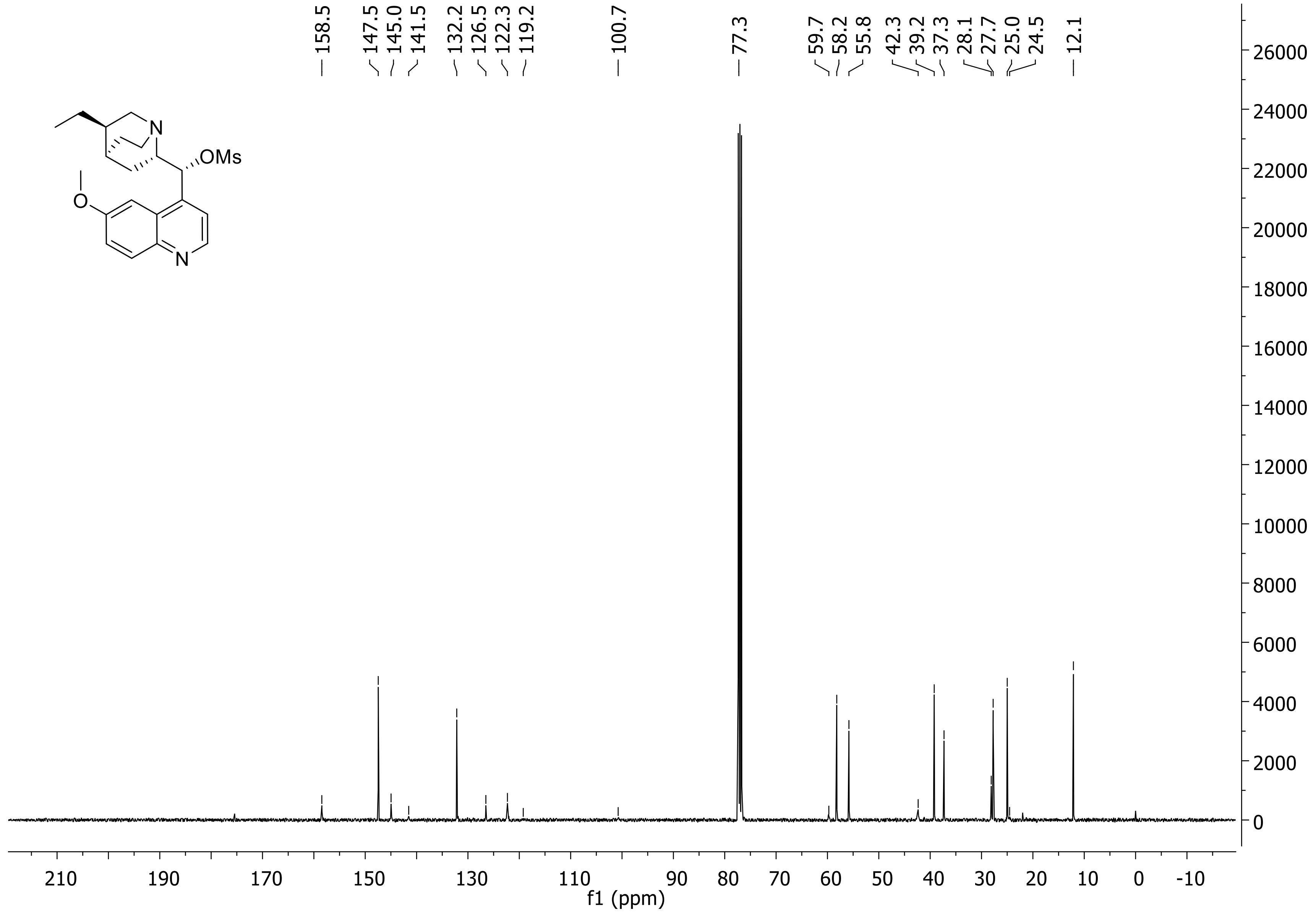

Figure S42. $^{13}C\{^{1}H\}$ NMR spectrum of **(1*R*)-((2*S*,4*S*,5*R*)-5-ethylquinuclidin-2-yl)(6-methoxyquinolin-4-yl)methyl methanesulfonate** ($CDCl_3$, 101 MHz).

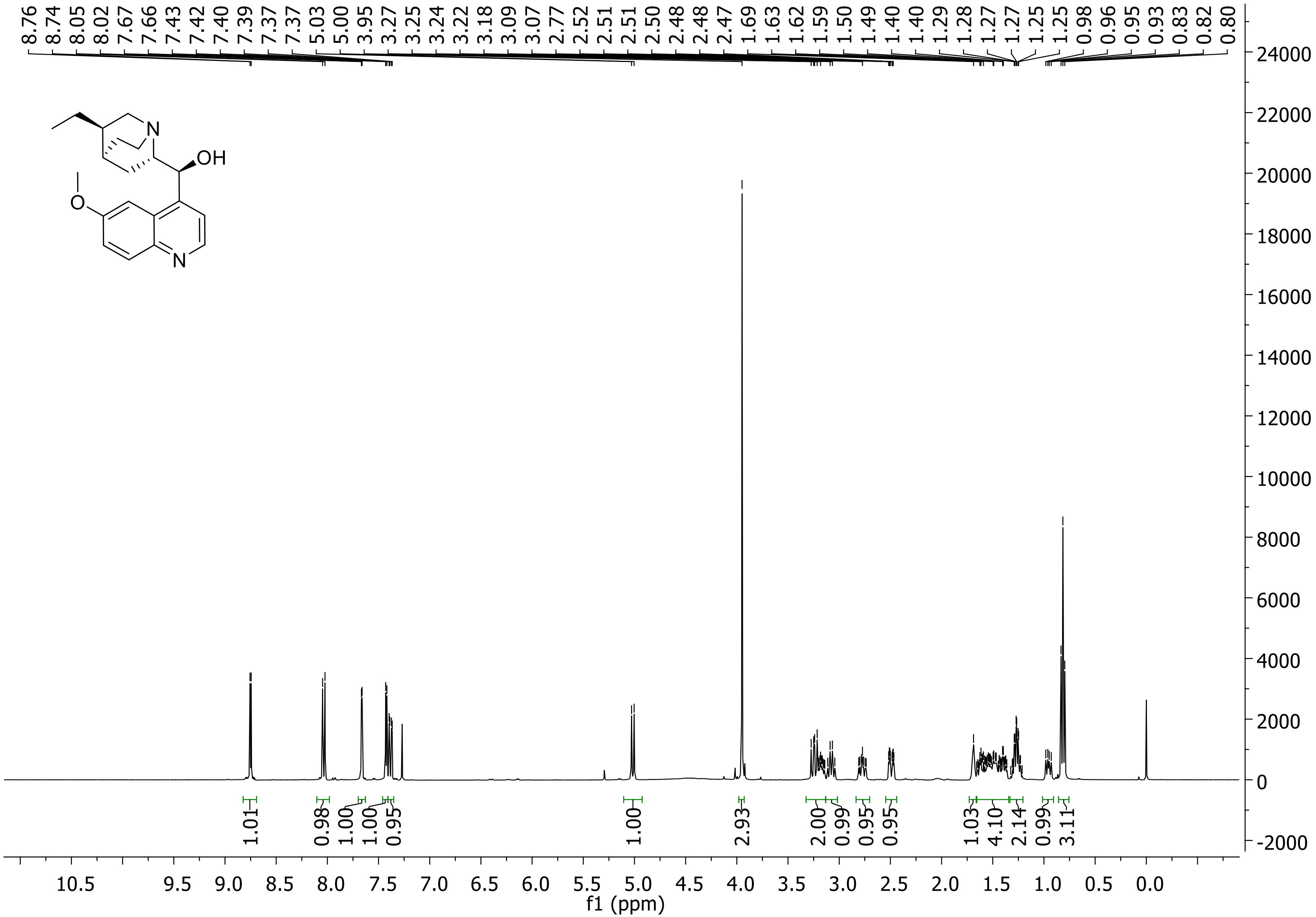

Figure S43. ^{1}H NMR spectrum of **(1*S*)-((2*S*,4*S*,5*R*)-5-ethylquinuclidin-2-yl)(6-methoxyquinolin-4-yl)methanol** ($CDCl_3$, 400 MHz).

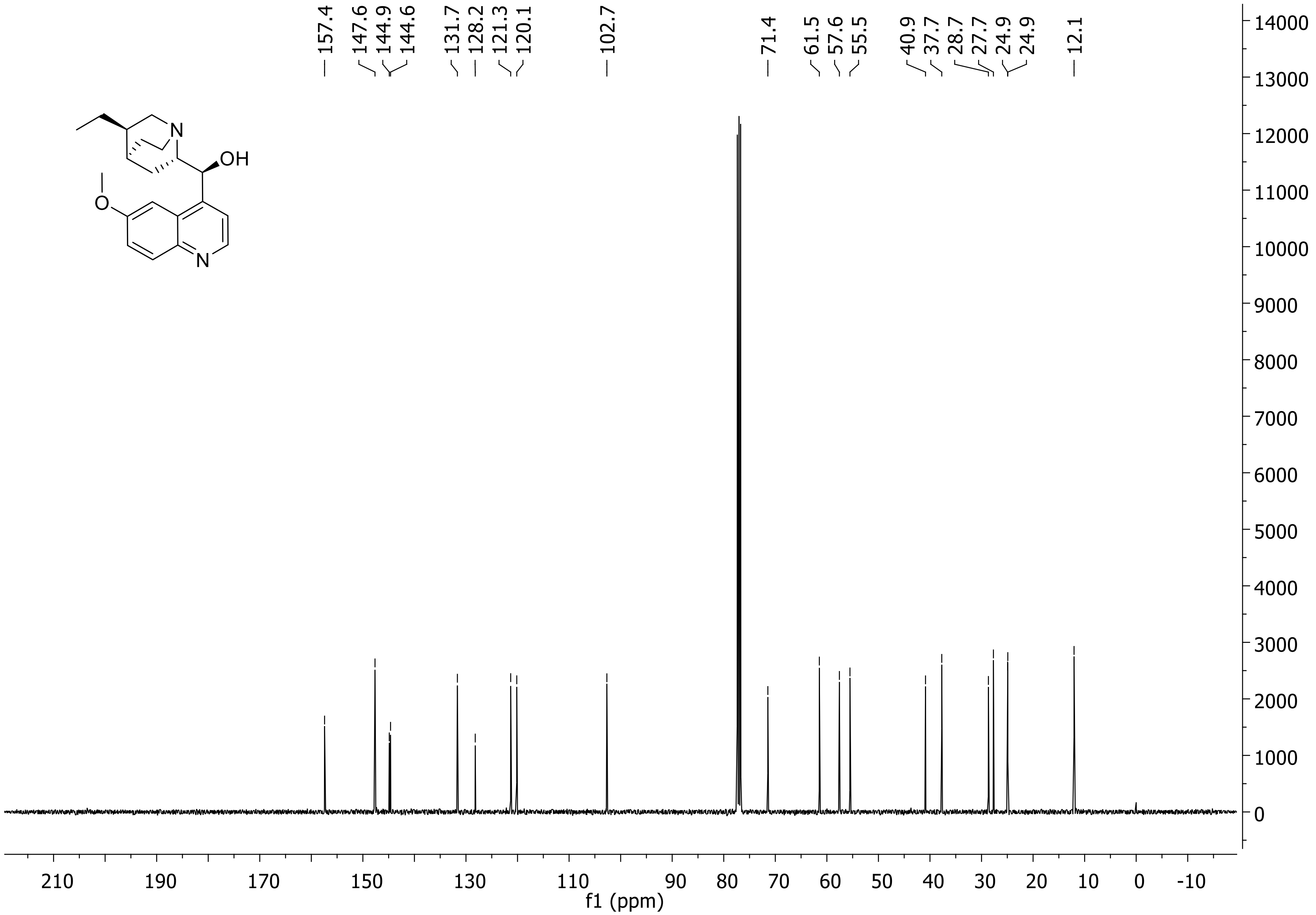

Figure S44. $^{13}C\{^1H\}$ NMR spectrum of **(1*S*)-((2*S*,4*S*,5*R*)-5-ethylquinuclidin-2-yl)(6-methoxyquinolin-4-yl)methanol** ($CDCl_3$, 101 MHz).

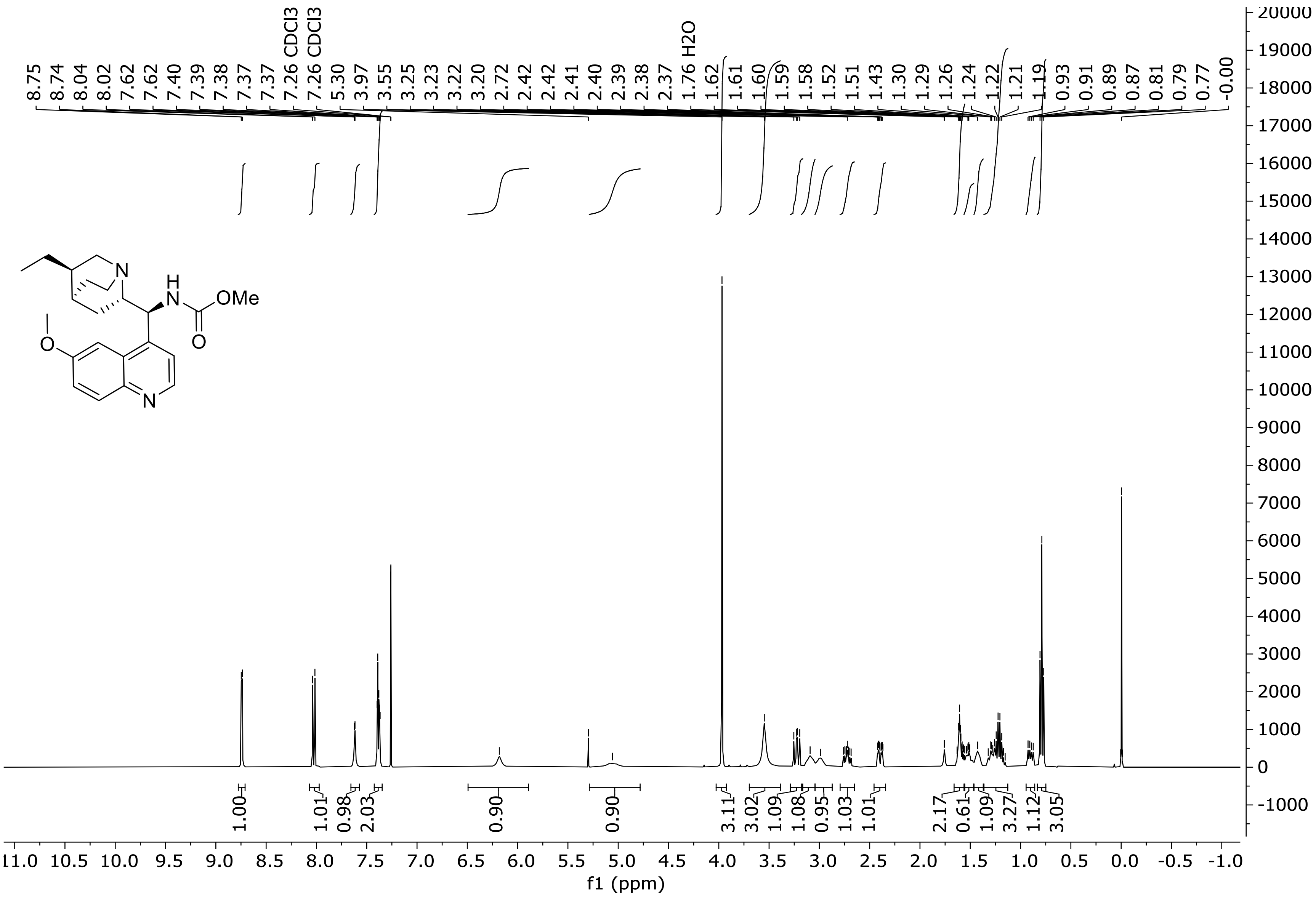

Figure S45. ^{1}H NMR spectrum of **methyl ((1*S*)-((2*S*,4*S*,5*R*)-5-ethylquinuclidin-2-yl)(6-methoxyquinolin-4-yl)methyl)carbamate** (CDCl$_3$, 400 MHz).

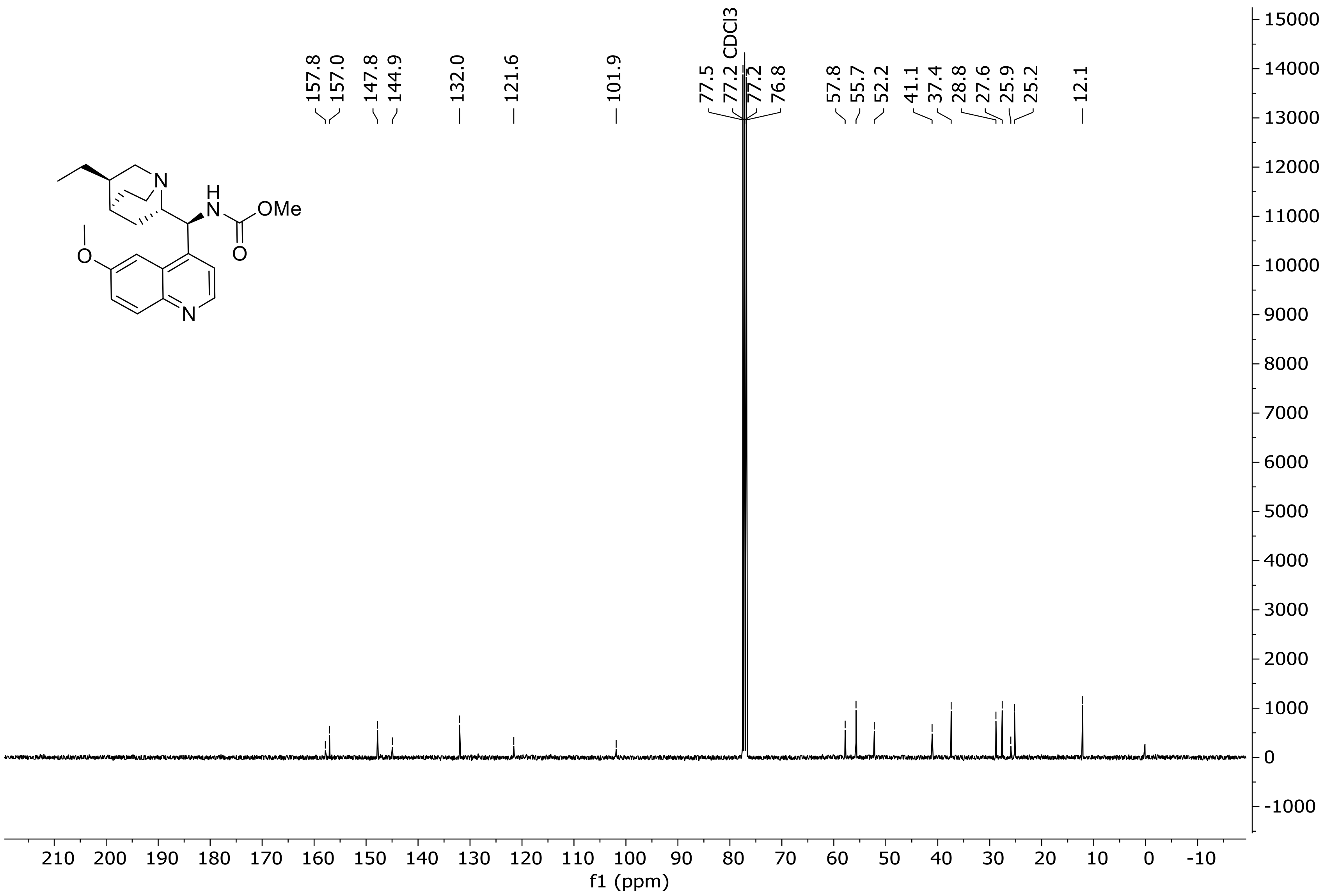

Figure S46. $^{13}C\{^{1}H\}$ NMR spectrum of **methyl ((1*S*)-((2*S*,4*S*,5*R*)-5-ethylquinuclidin-2-yl)(6-methoxyquinolin-4-yl)methyl)carbamate** ($CDCl_3$, 101 MHz).

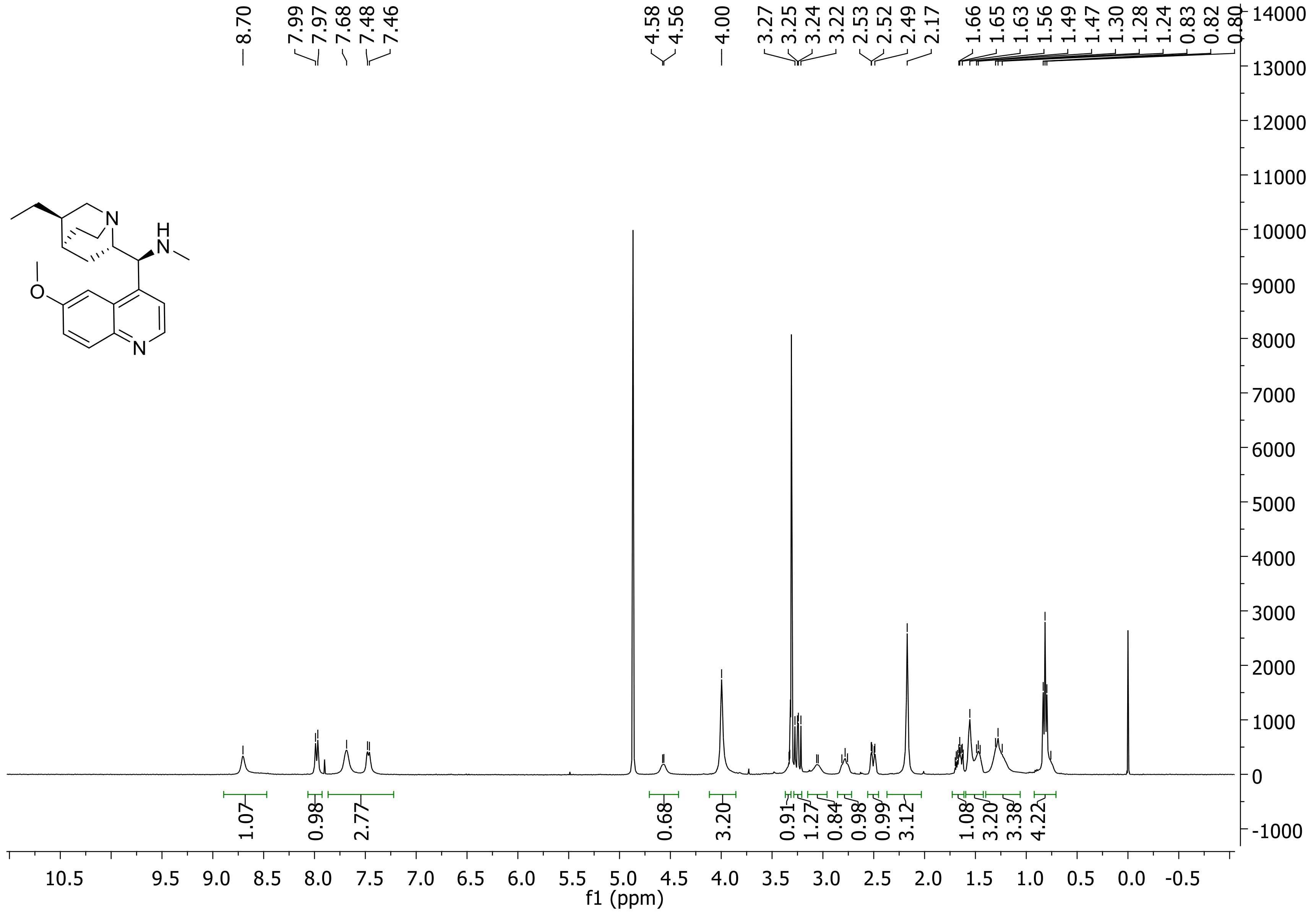

Figure S47. ^{1}H NMR spectrum of **(1*S*)-1-((2*S*,4*S*,5*R*)-5-ethylquinuclidin-2-yl)-1-(6-methoxyquinolin-4-yl)-N-methylmethanamine** (MeOD, 400 MHz).

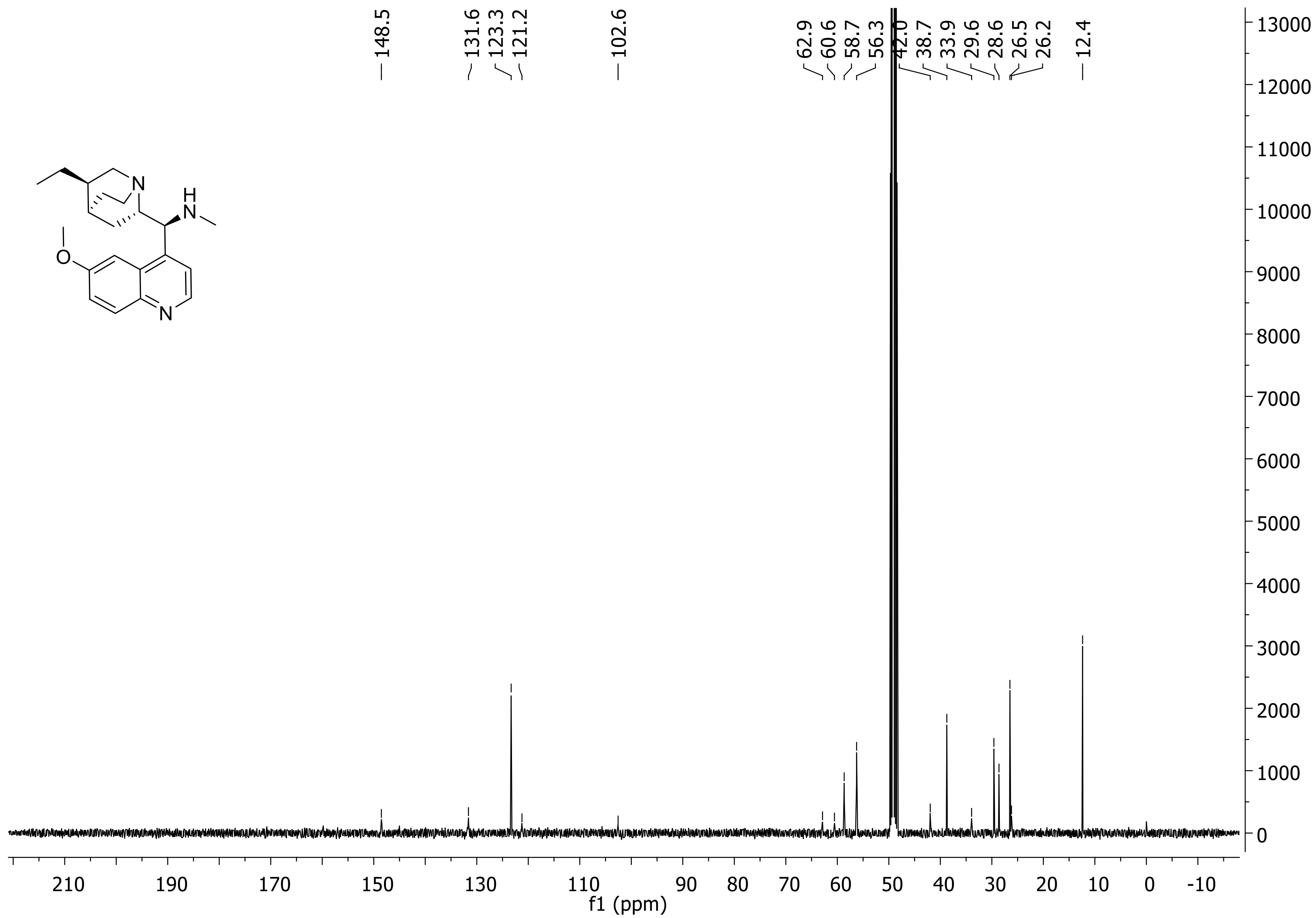

Figure S48. $^{13}C\{^{1}H\}$ NMR spectrum of **(1*S*)-1-((2*S*,4*S*,5*R*)-5-ethylquinuclidin-2-yl)-1-(6-methoxyquinolin-4-yl)-N-methylmethanamine** (MeOD, 101 MHz).

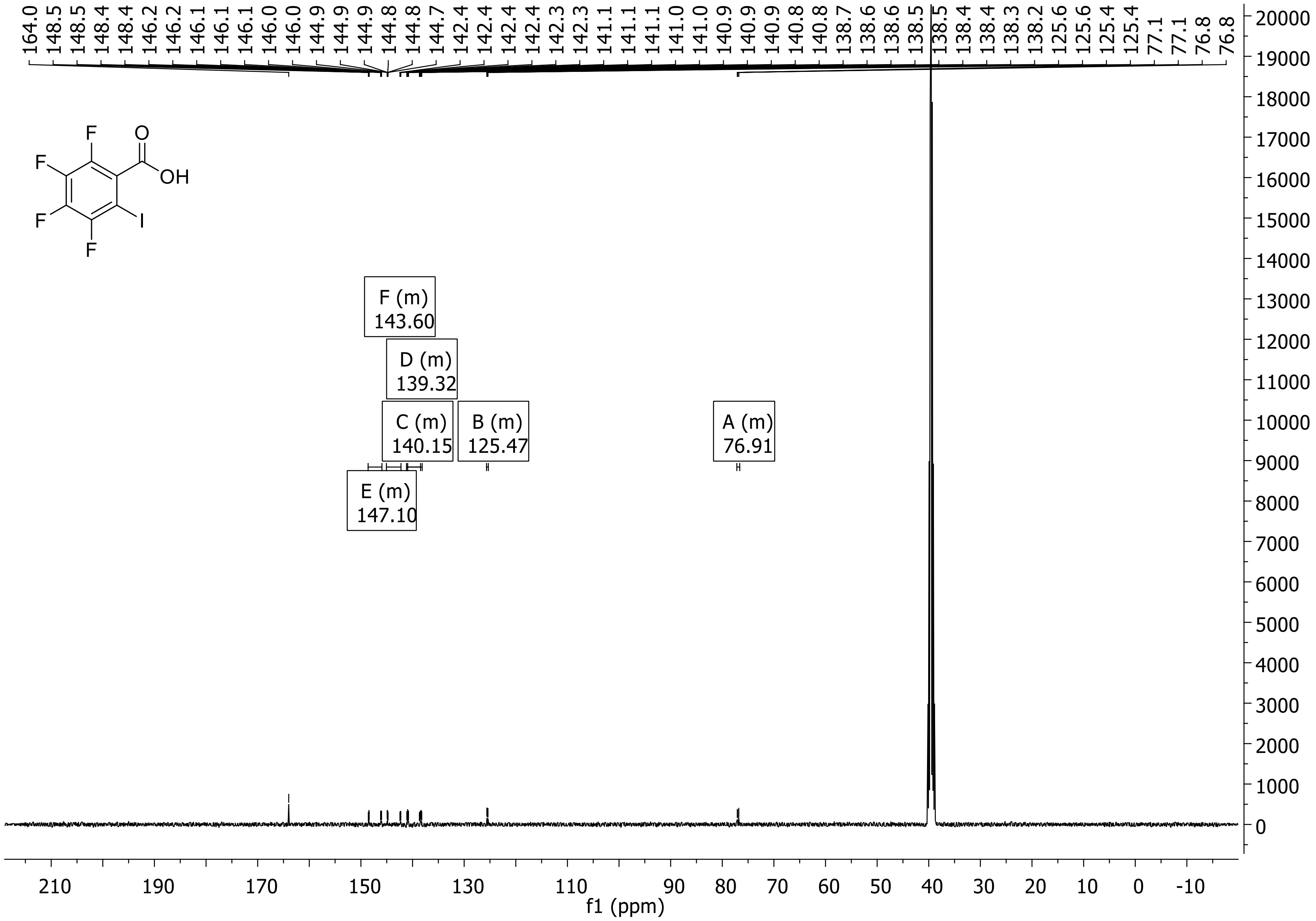

Figure S49. $^{13}C\{^1H\}$ NMR spectrum of **2,3,4,5-tetrafluoro-6-iodobenzoic acid** ([D_6]DMSO, 101 MHz).

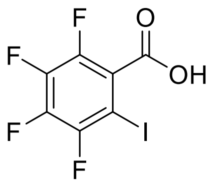

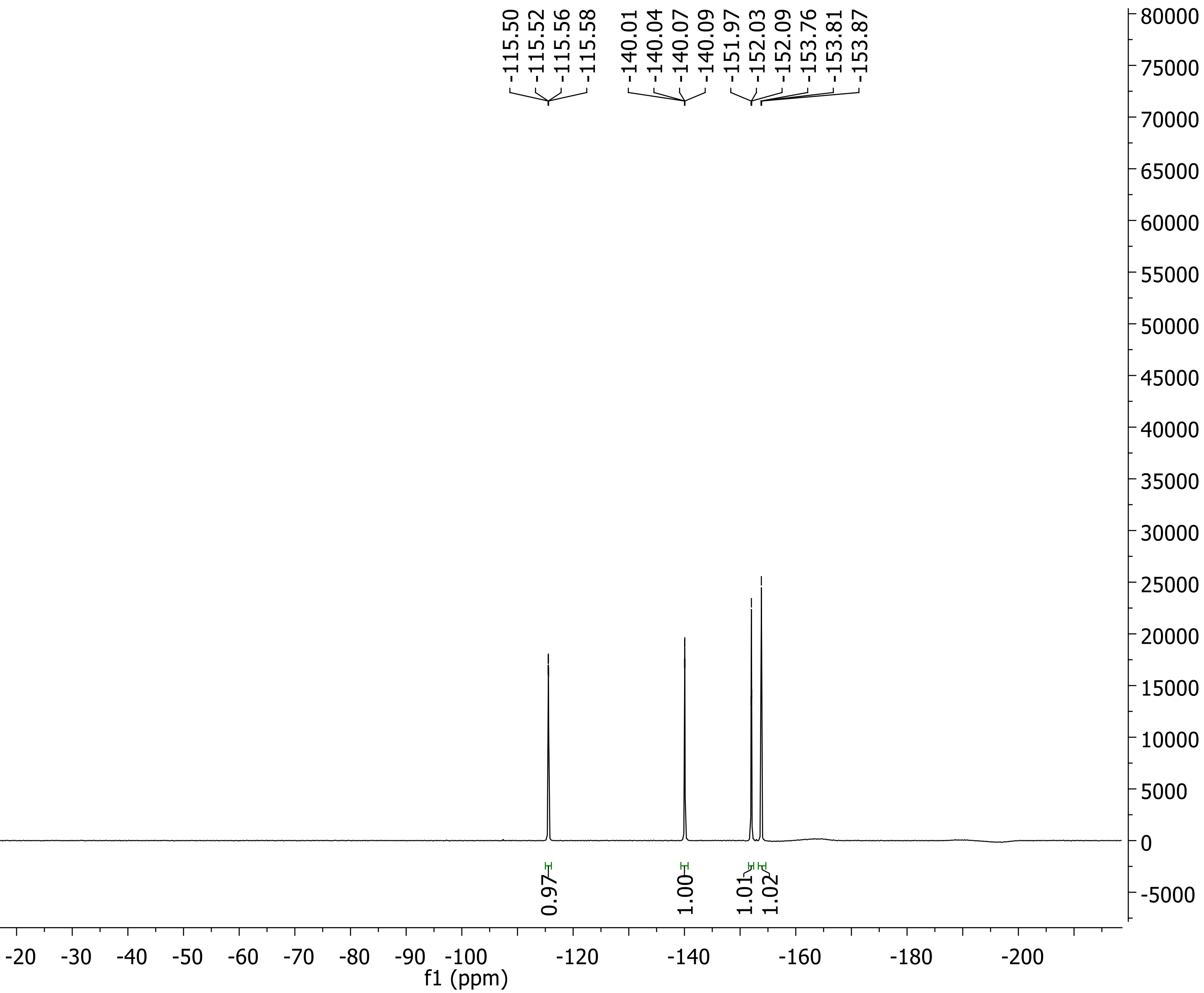

Figure S50. ^{19}F NMR spectrum of **2,3,4,5-tetrafluoro-6-iodobenzoic acid** ([D_6]DMSO, 376 MHz).

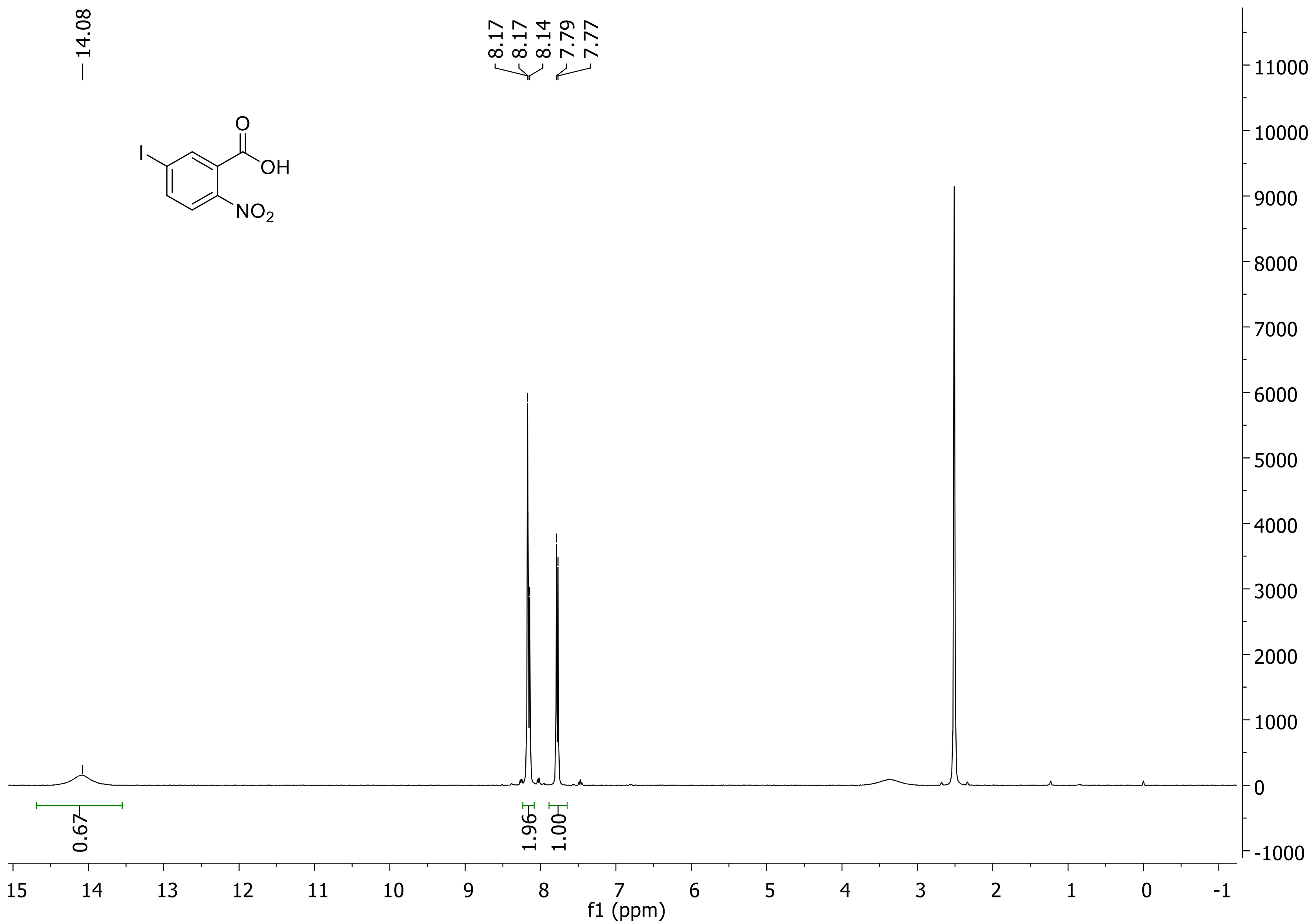

Figure S51. ^{1}H NMR spectrum of **5-iodo-2-nitrobenzoic acid** ([D$_6$]DMSO, 400 MHz).

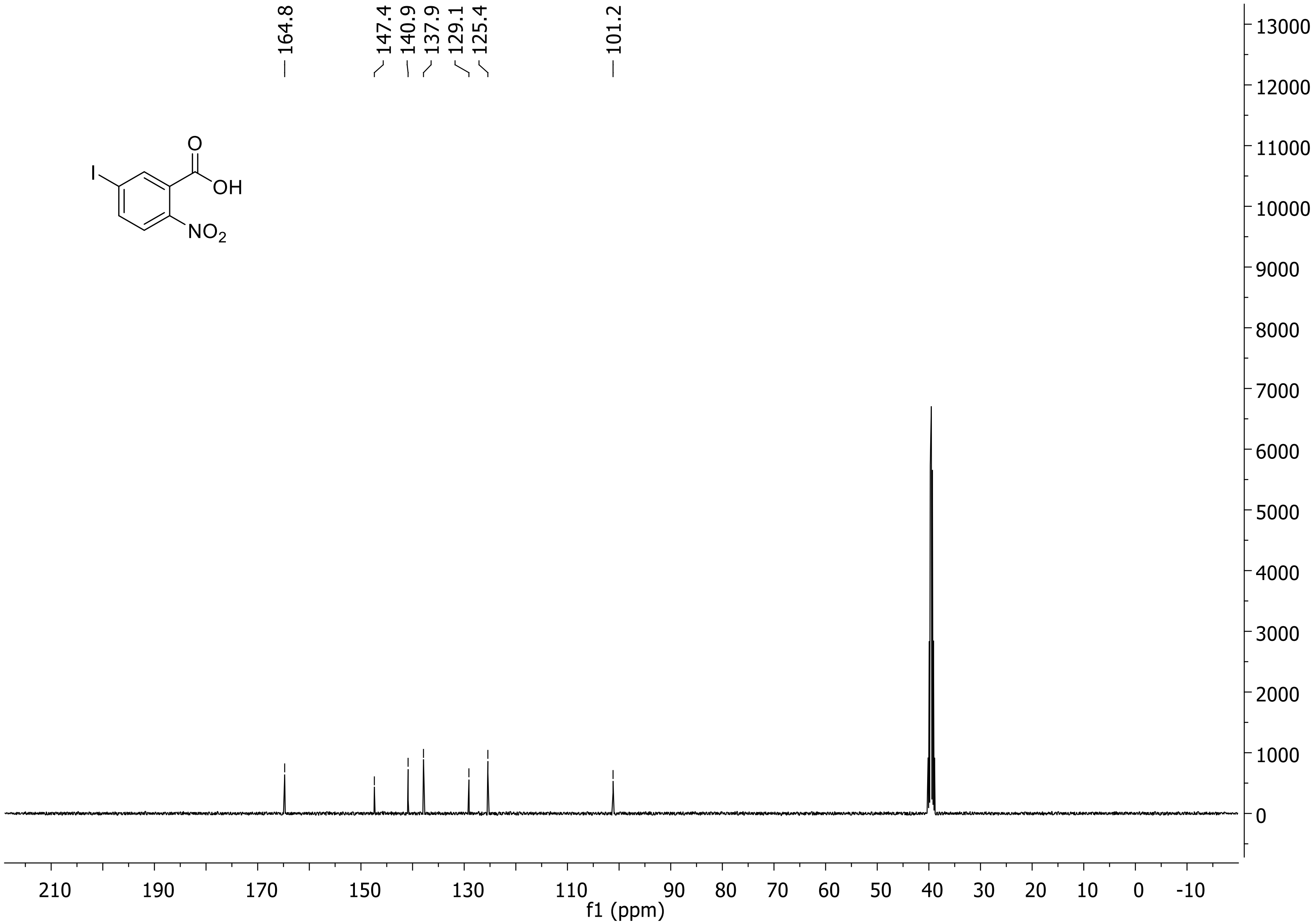

Figure S52. $^{13}C\{^{1}H\}$ NMR spectrum of **5-iodo-2-nitrobenzoic acid** ([D$_6$]DMSO, 101 MHz).

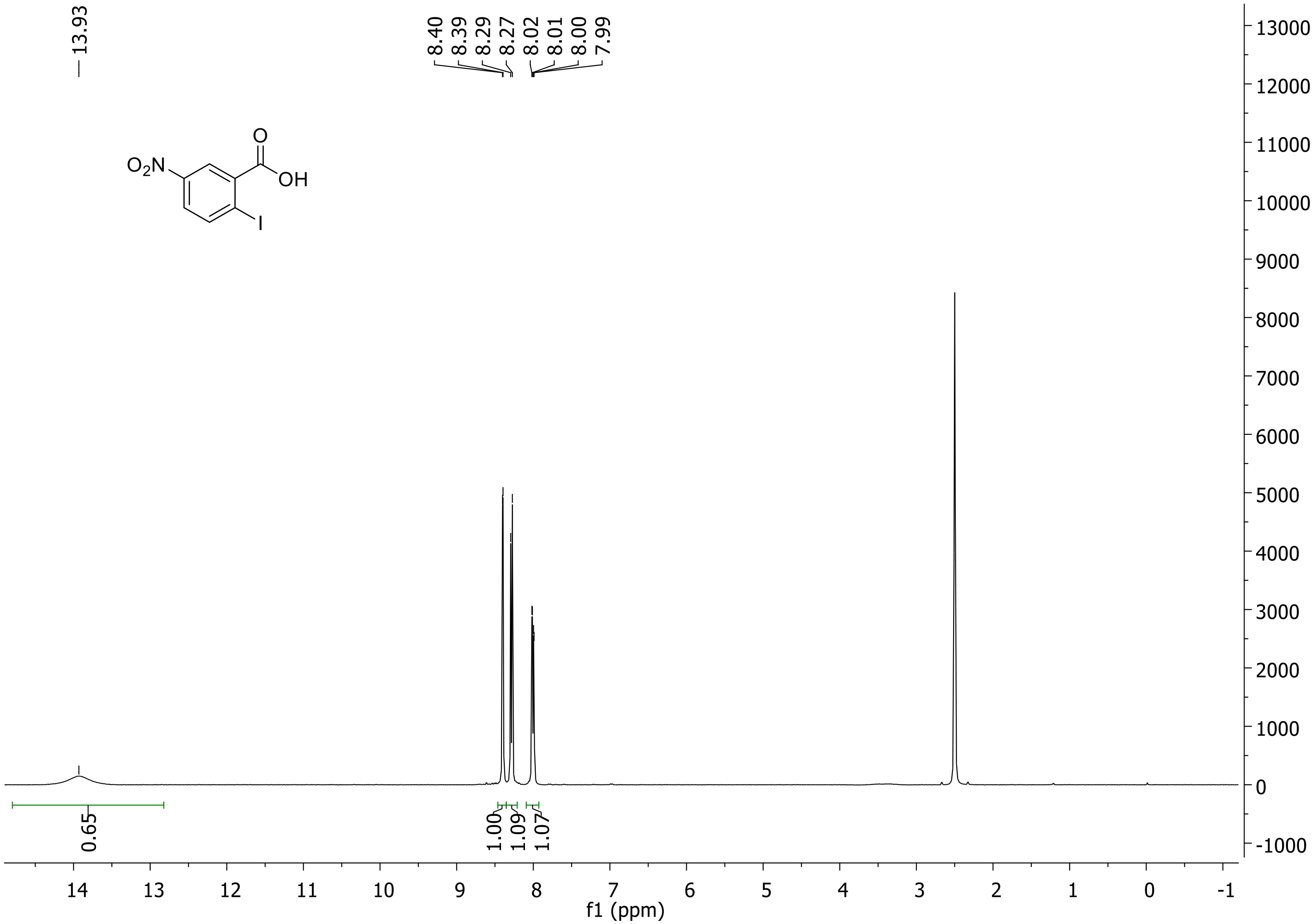

Figure S53. ^{1}H NMR spectrum of **2-iodo-5-nitrobenzoic acid** ([D$_6$]DMSO, 400 MHz).

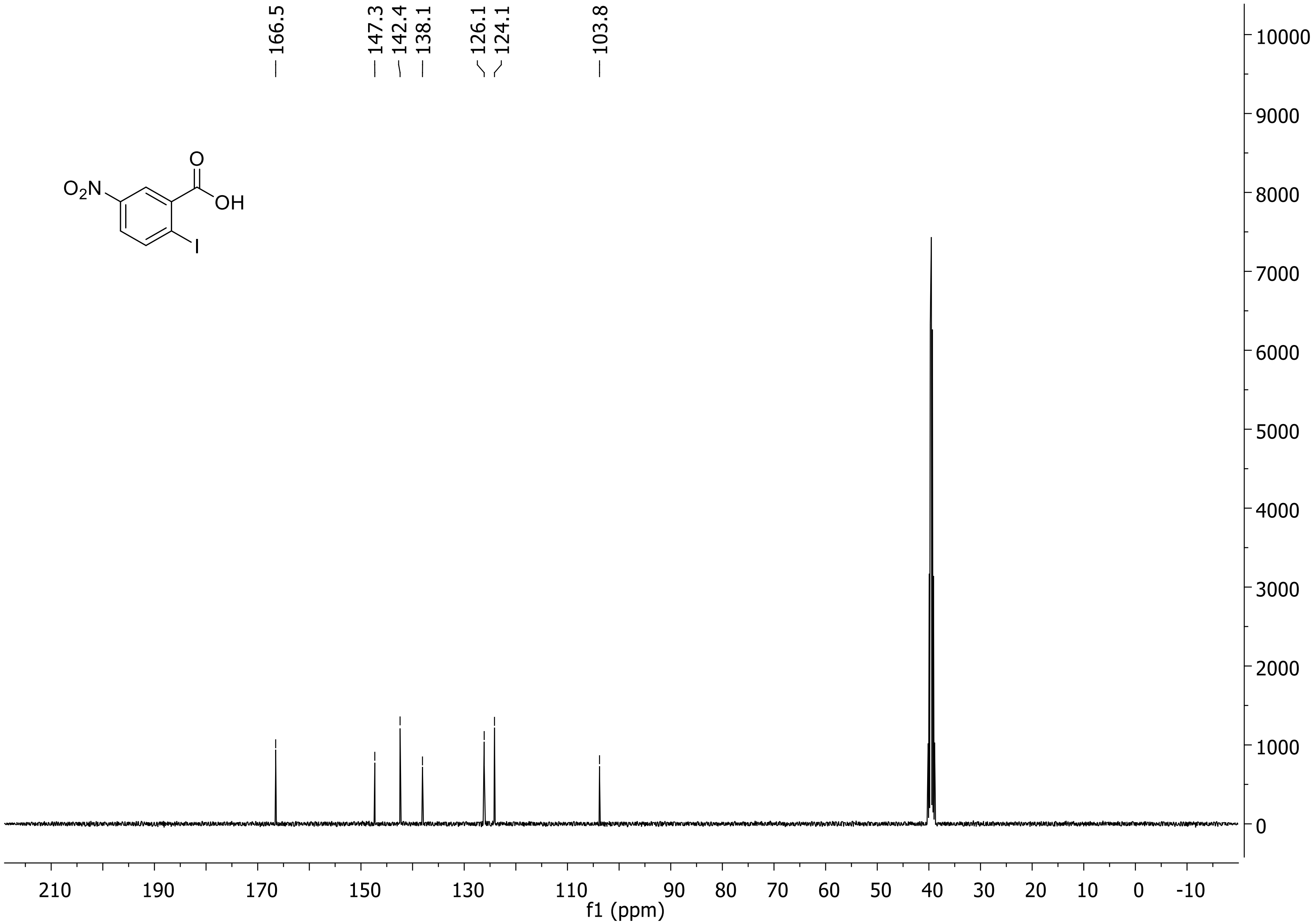

Figure S54. $^{13}C\{^{1}H\}$ NMR spectrum of **2-iodo-5-nitrobenzoic acid** ($[D_6]$DMSO, 101 MHz).

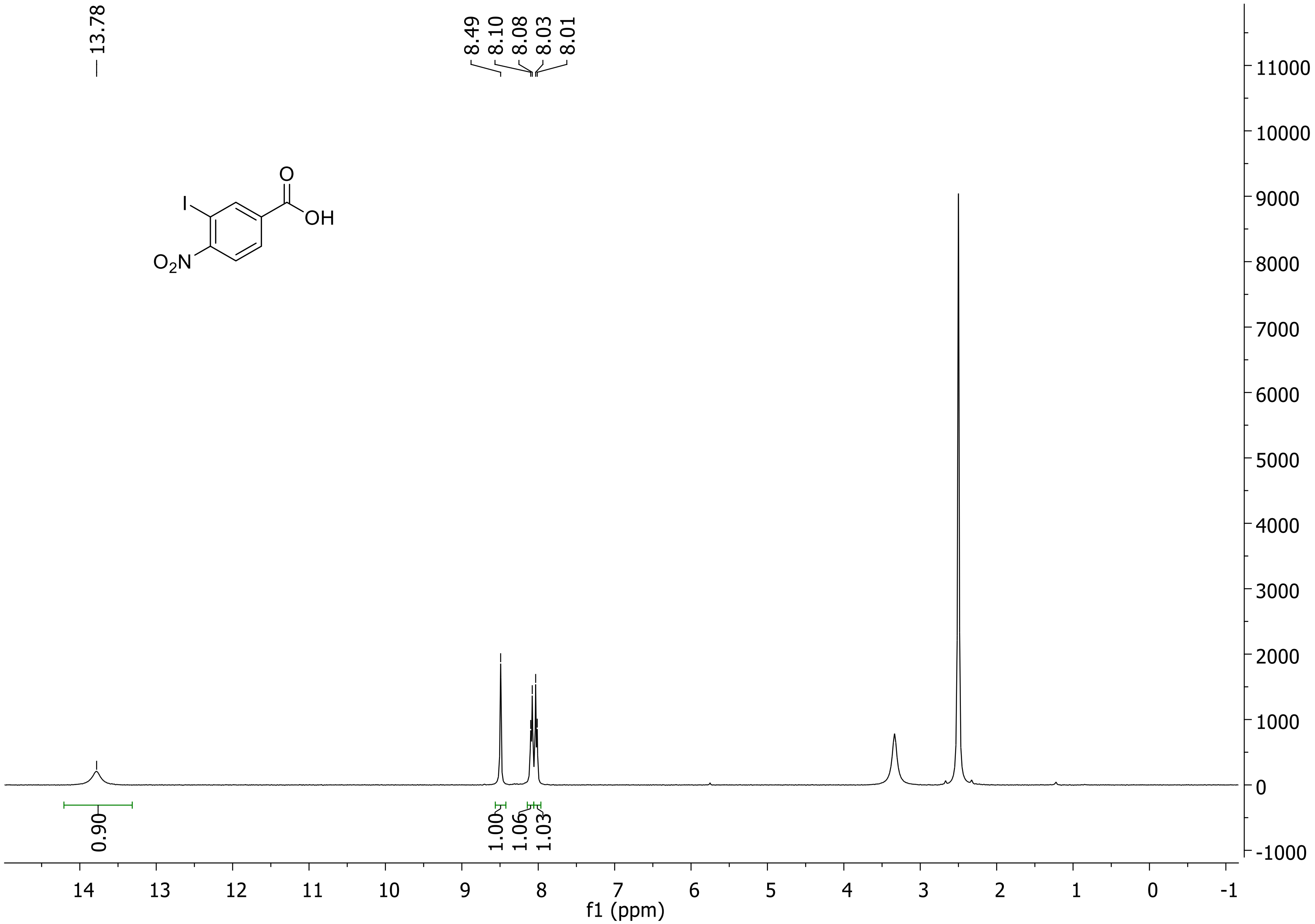

Figure S55. ^{1}H NMR spectrum of **3-iodo-4-nitrobenzoic acid** ([D$_6$]DMSO, 400 MHz).

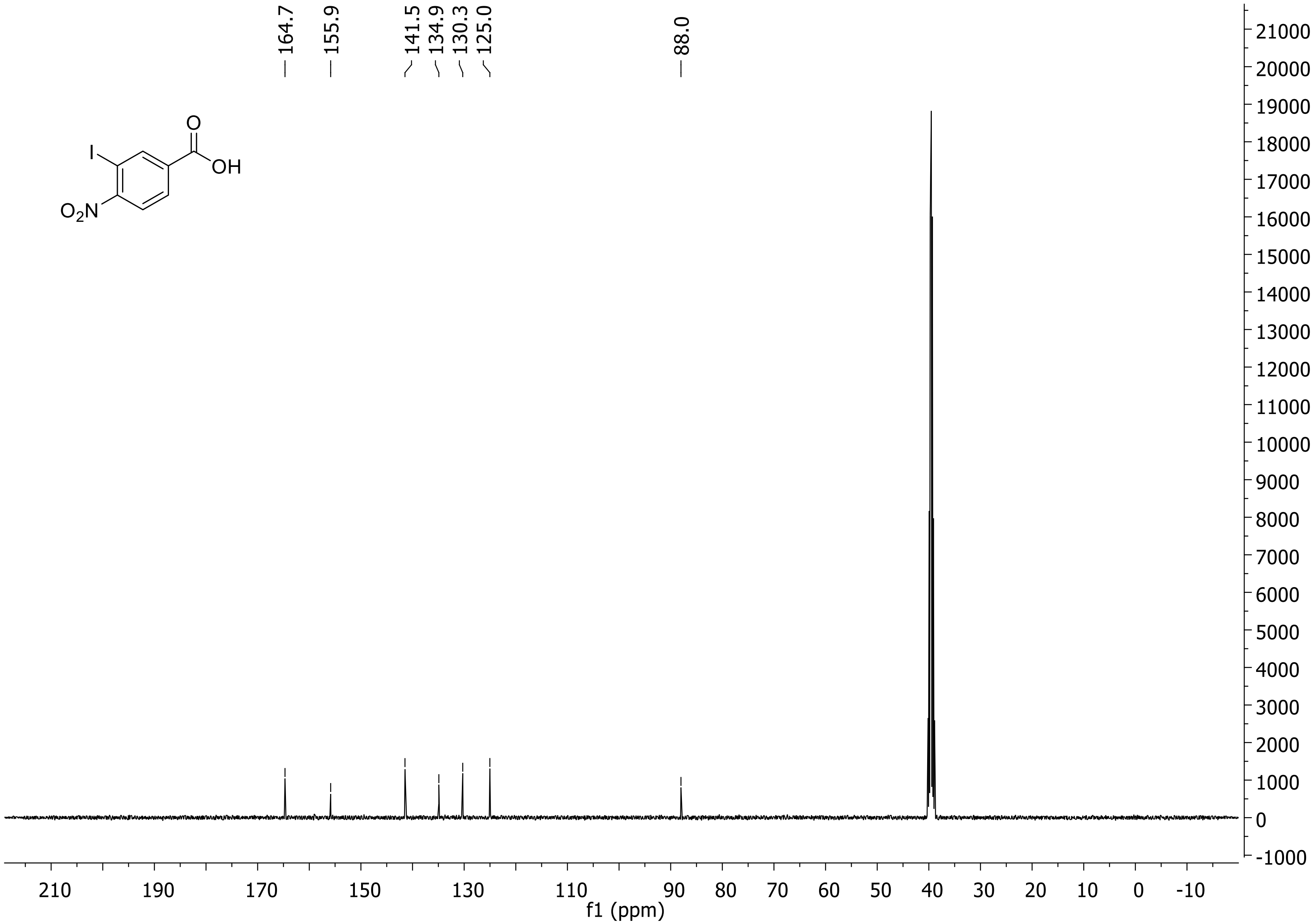

Figure S56. ${}^{13}C\{{}^{1}H\}$ NMR spectrum of **3-iodo-4-nitrobenzoic acid** ([D$_6$]DMSO, 101 MHz).

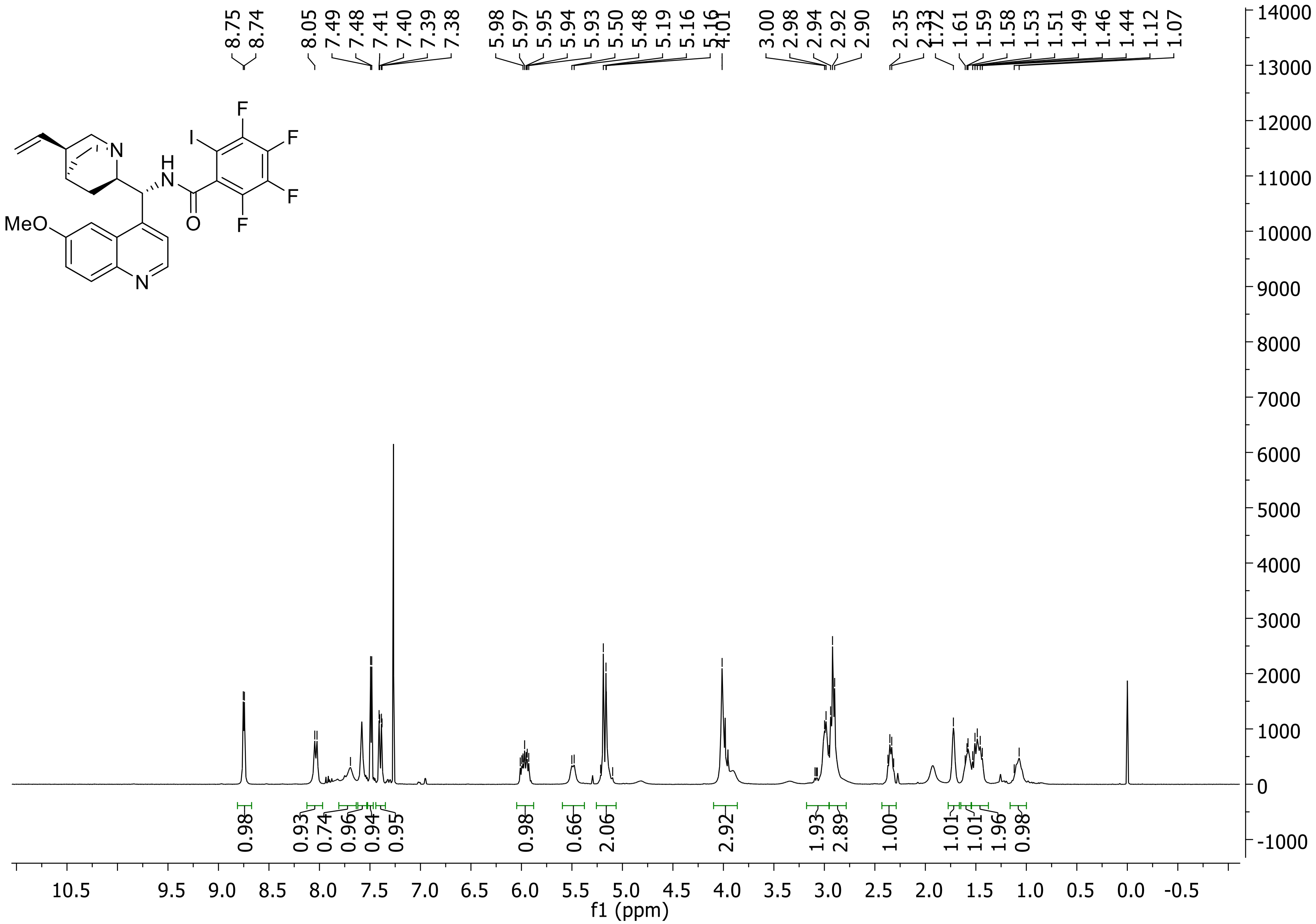

Figure S57. ^{1}H NMR spectrum of catalyst **A** ($CDCl_3$, 400 MHz).

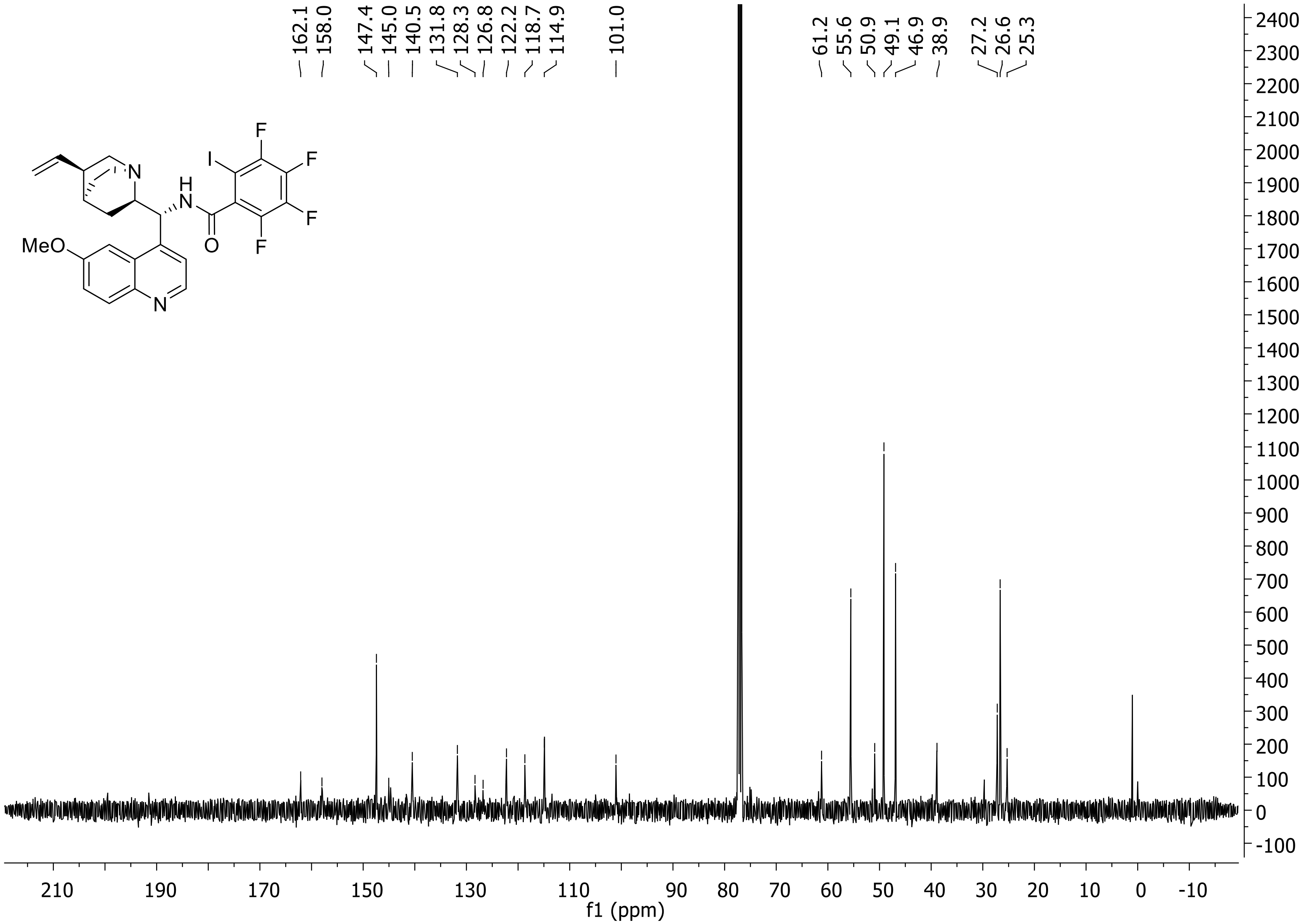

Figure S58. $^{13}C\{^{1}H\}$ NMR spectrum of catalyst **A** ($CDCl_3$, 101 MHz).

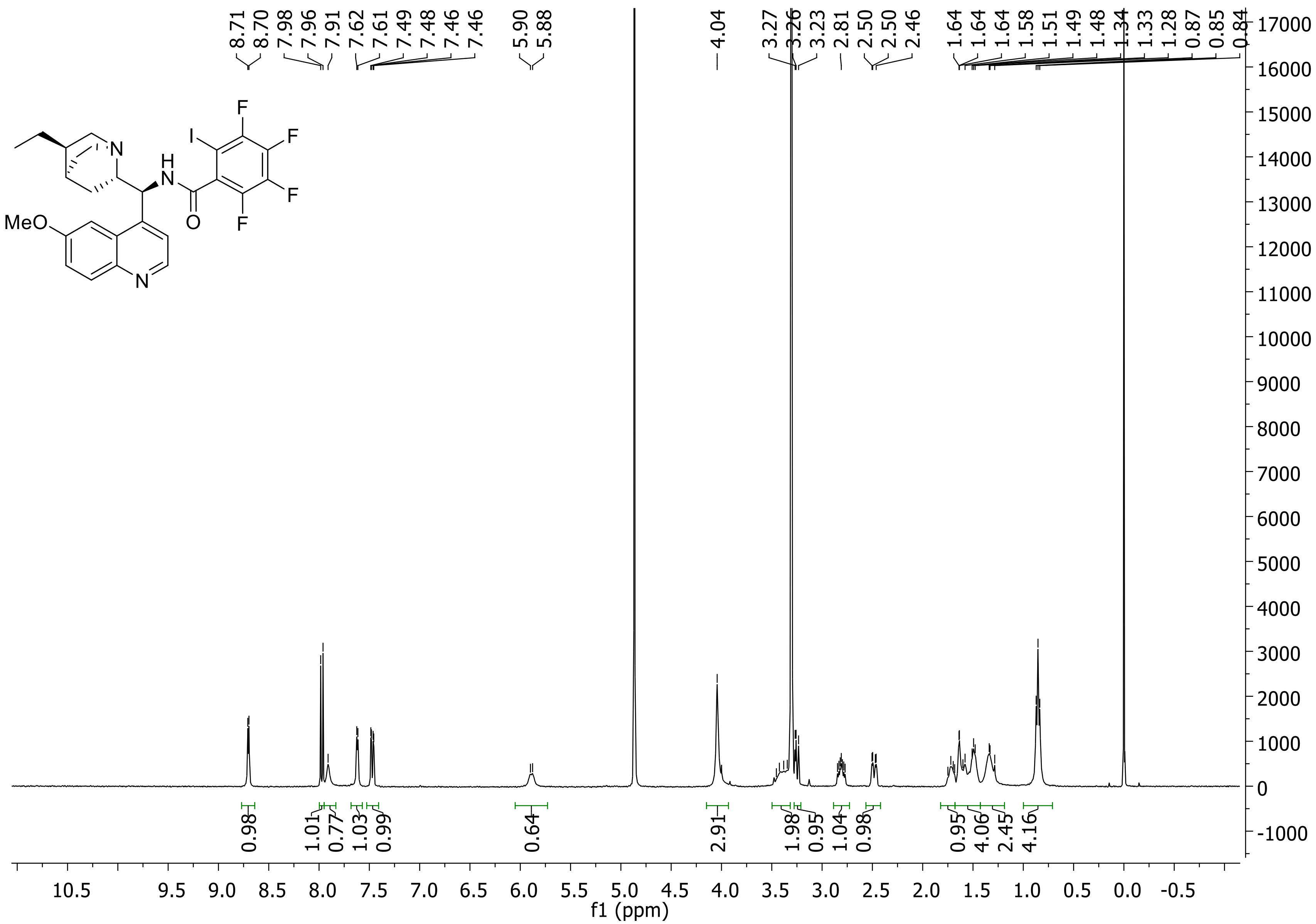

Figure S59. ^{1}H NMR spectrum of catalyst **B** ([D_4]methanol, 400 MHz).

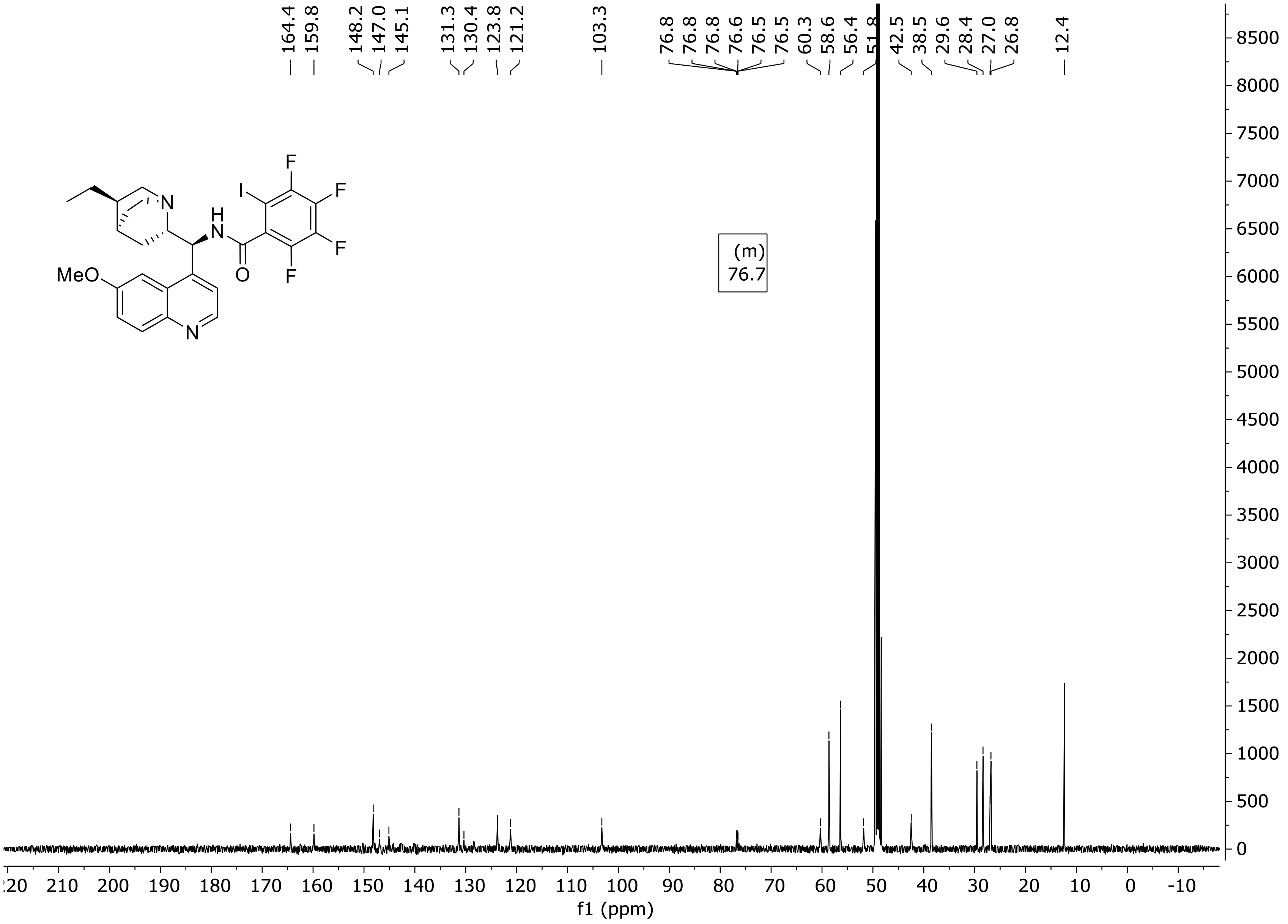

Figure S60. $^{13}C\{^{1}H\}$ NMR spectrum of catalyst **B** ([D$_4$]methanol, 101 MHz).

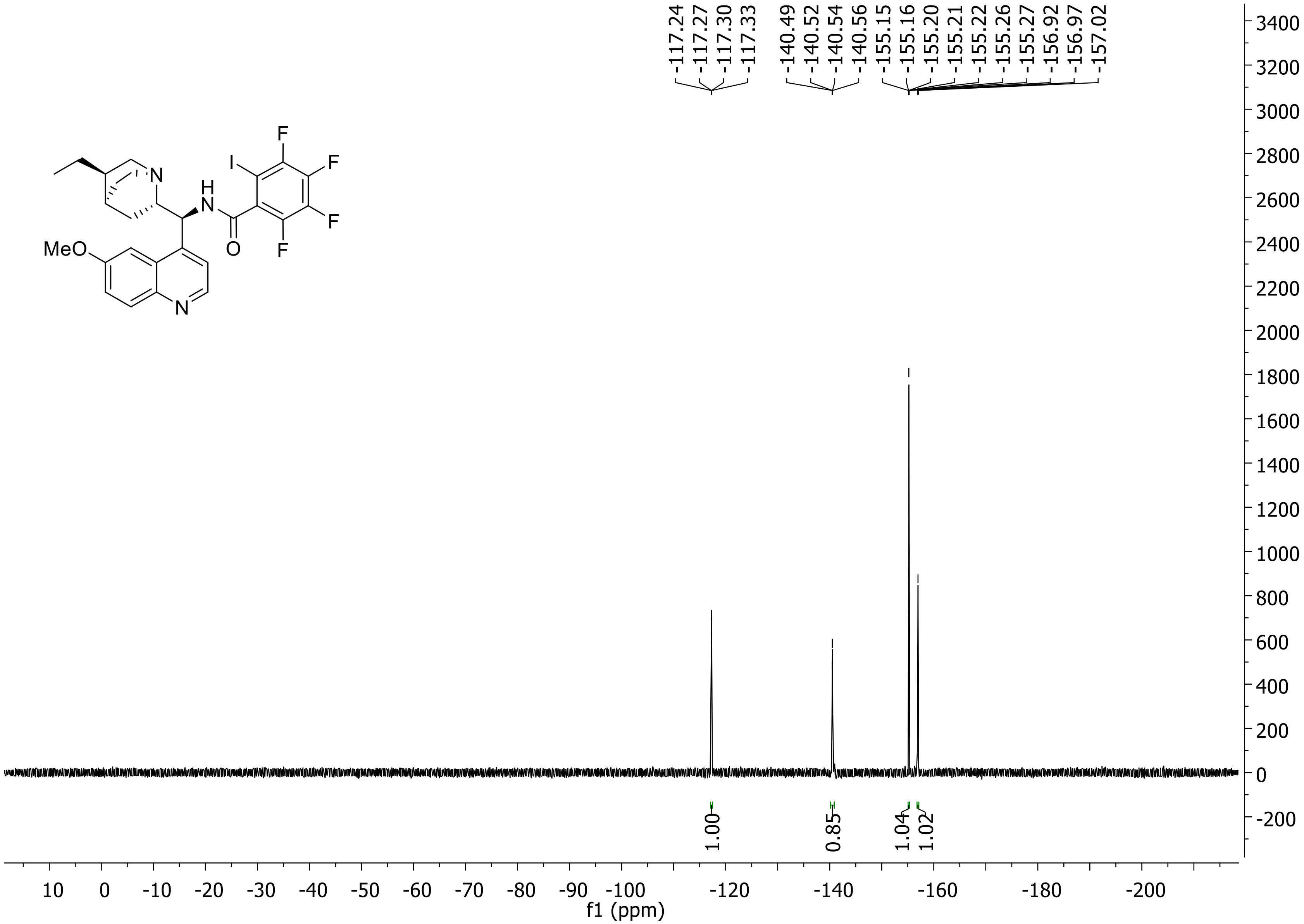

Figure S61. ^{19}F NMR spectrum of catalyst **B** ([D_4]methanol, 376 MHz).

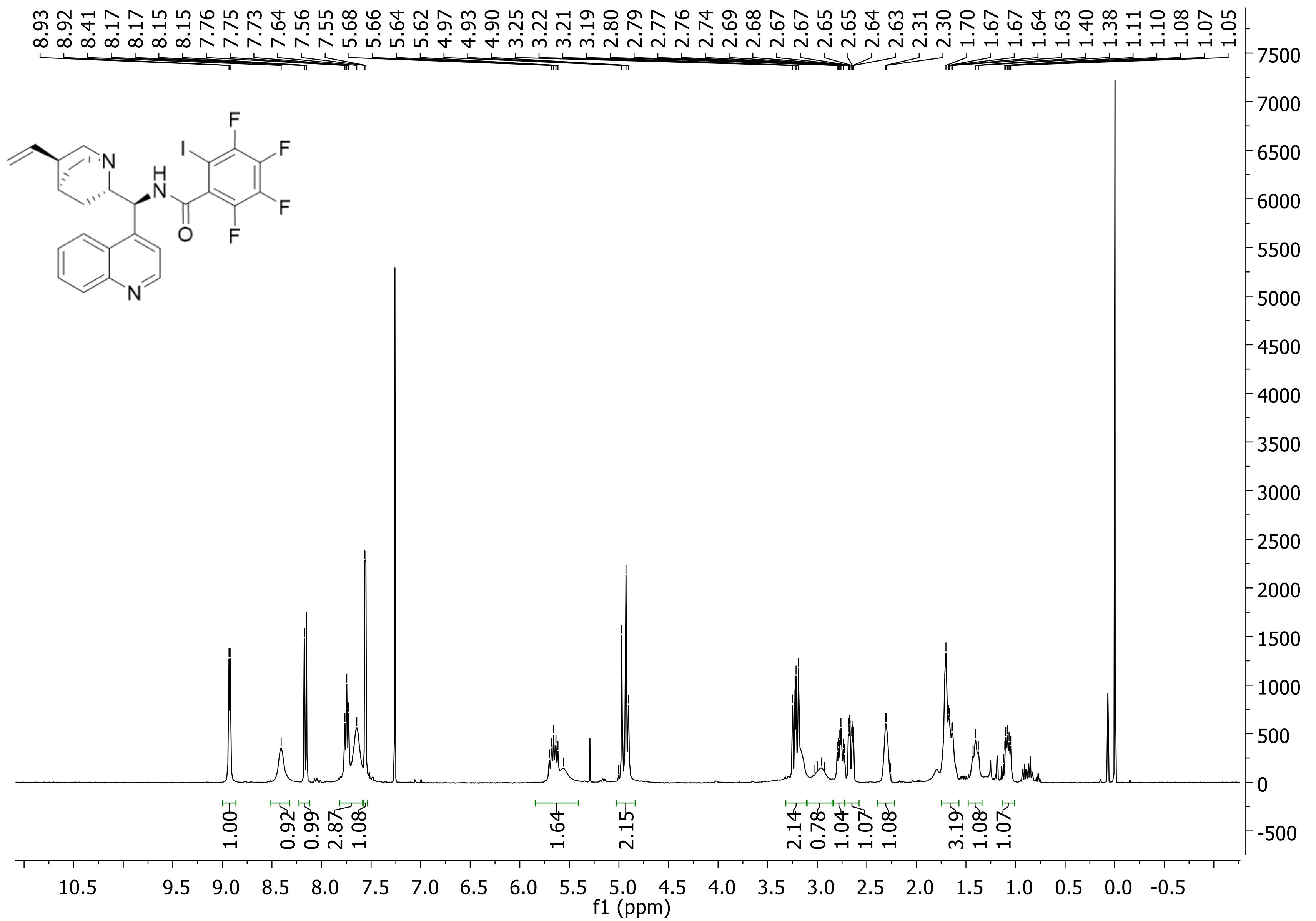

Figure S62. ^{1}H NMR spectrum of catalyst **C** ($CDCl_3$, 400 MHz).

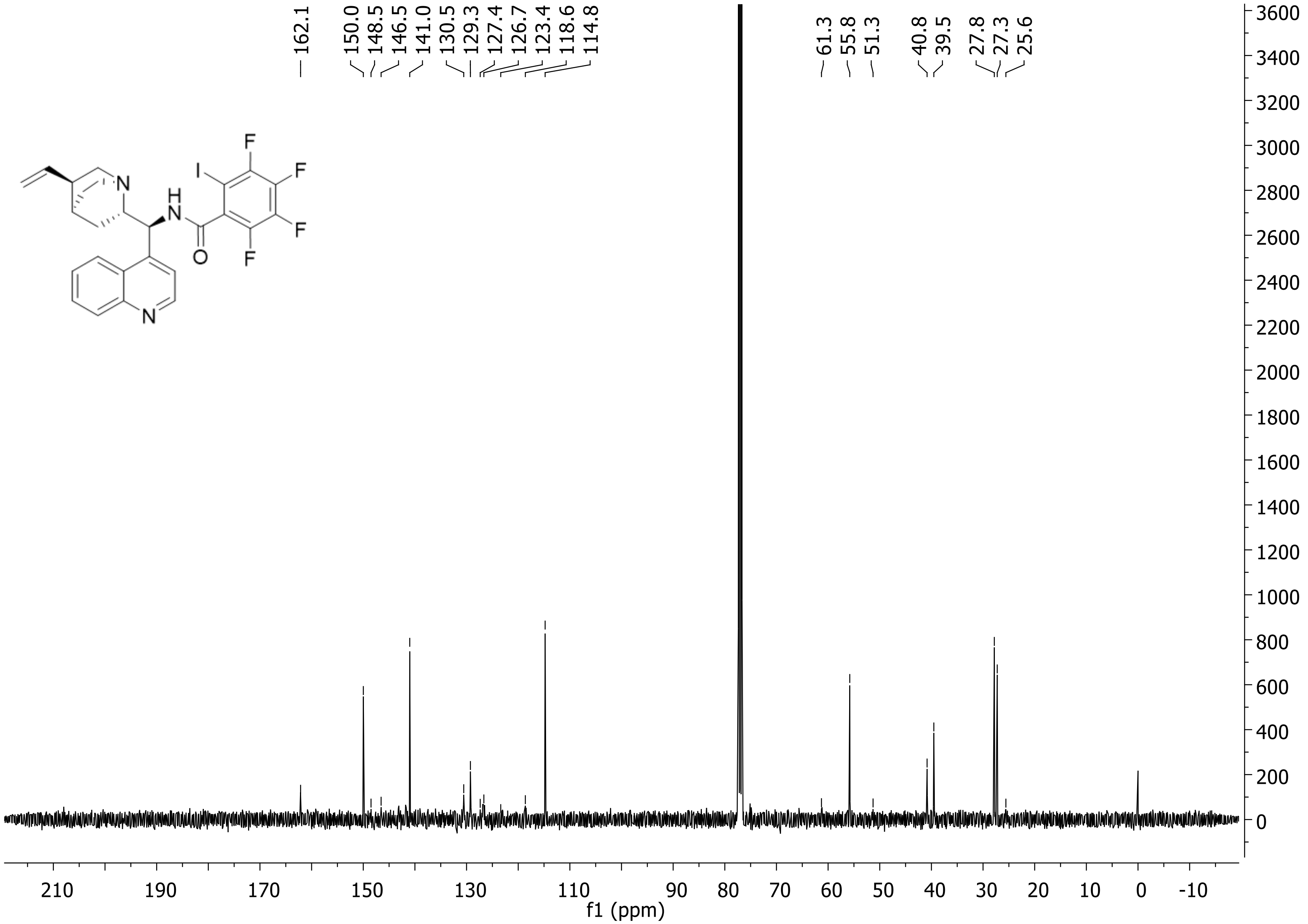

Figure S63. $^{13}C\{^{1}H\}$ NMR spectrum of catalyst **C** ($CDCl_3$, 101 MHz).

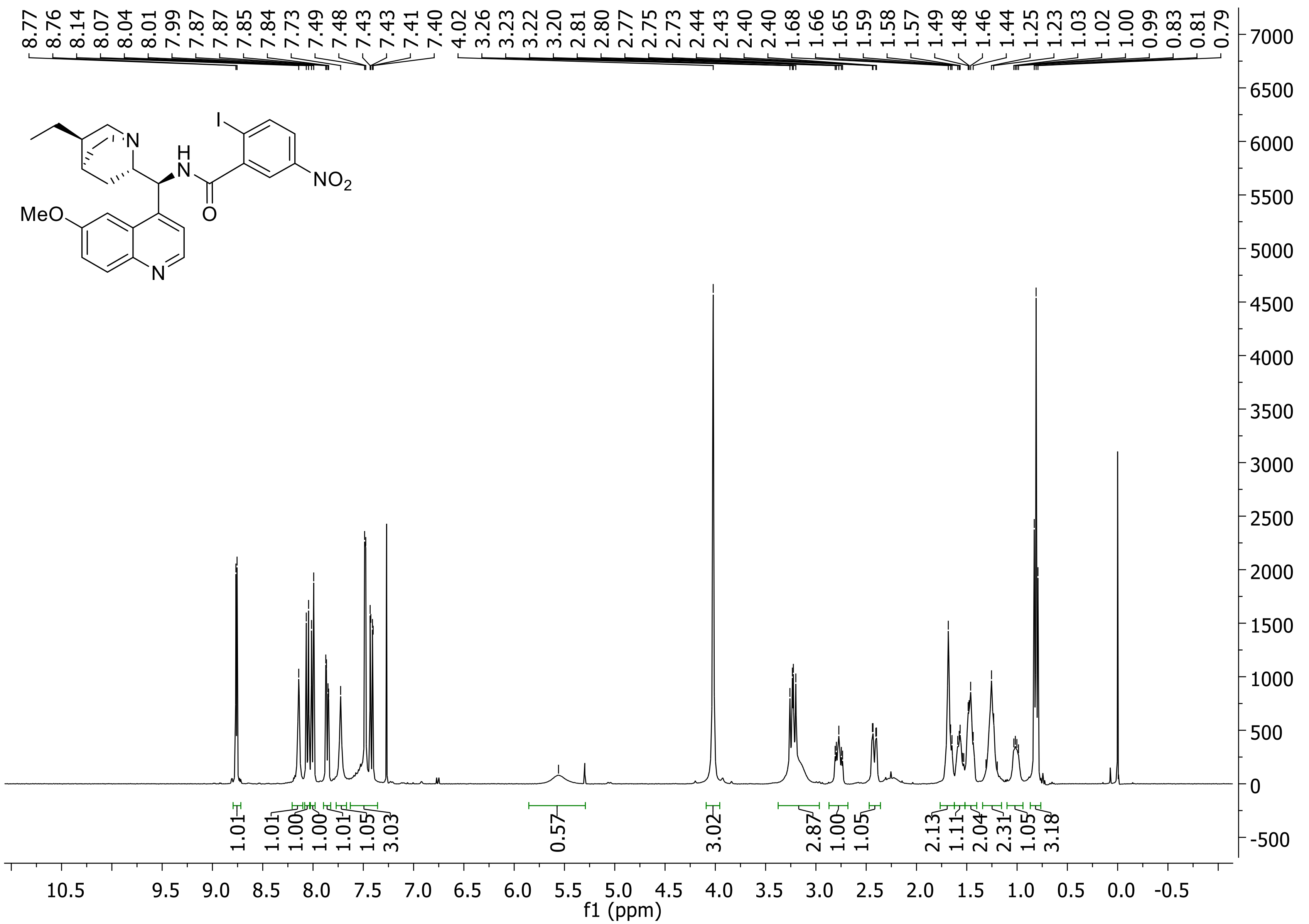

Figure S64. ^{1}H NMR spectrum of catalyst **D** ($CDCl_3$, 400 MHz).

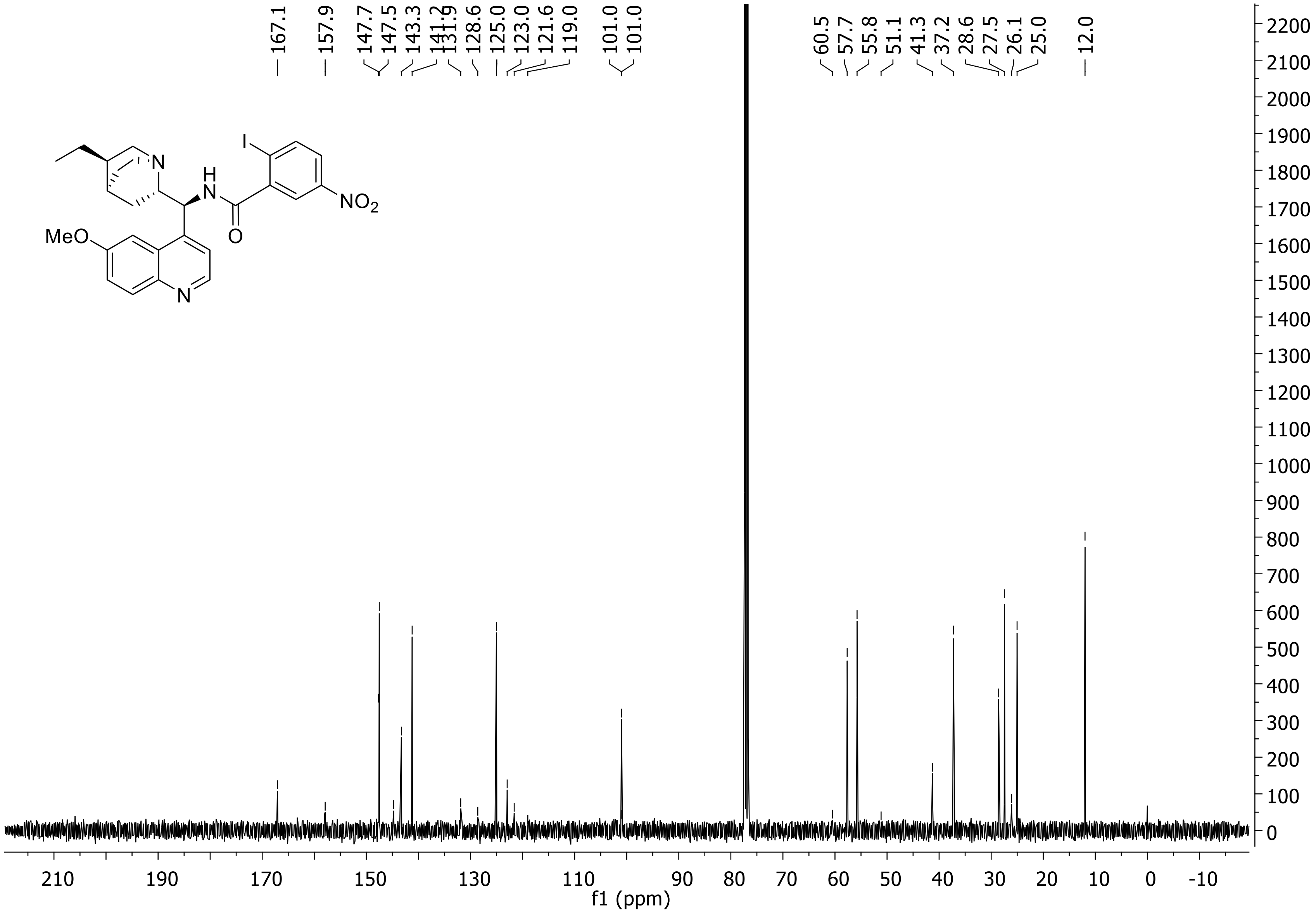

Figure S65. $^{13}C\{^1H\}$ NMR spectrum of catalyst **D** ($CDCl_3$, 101 MHz).

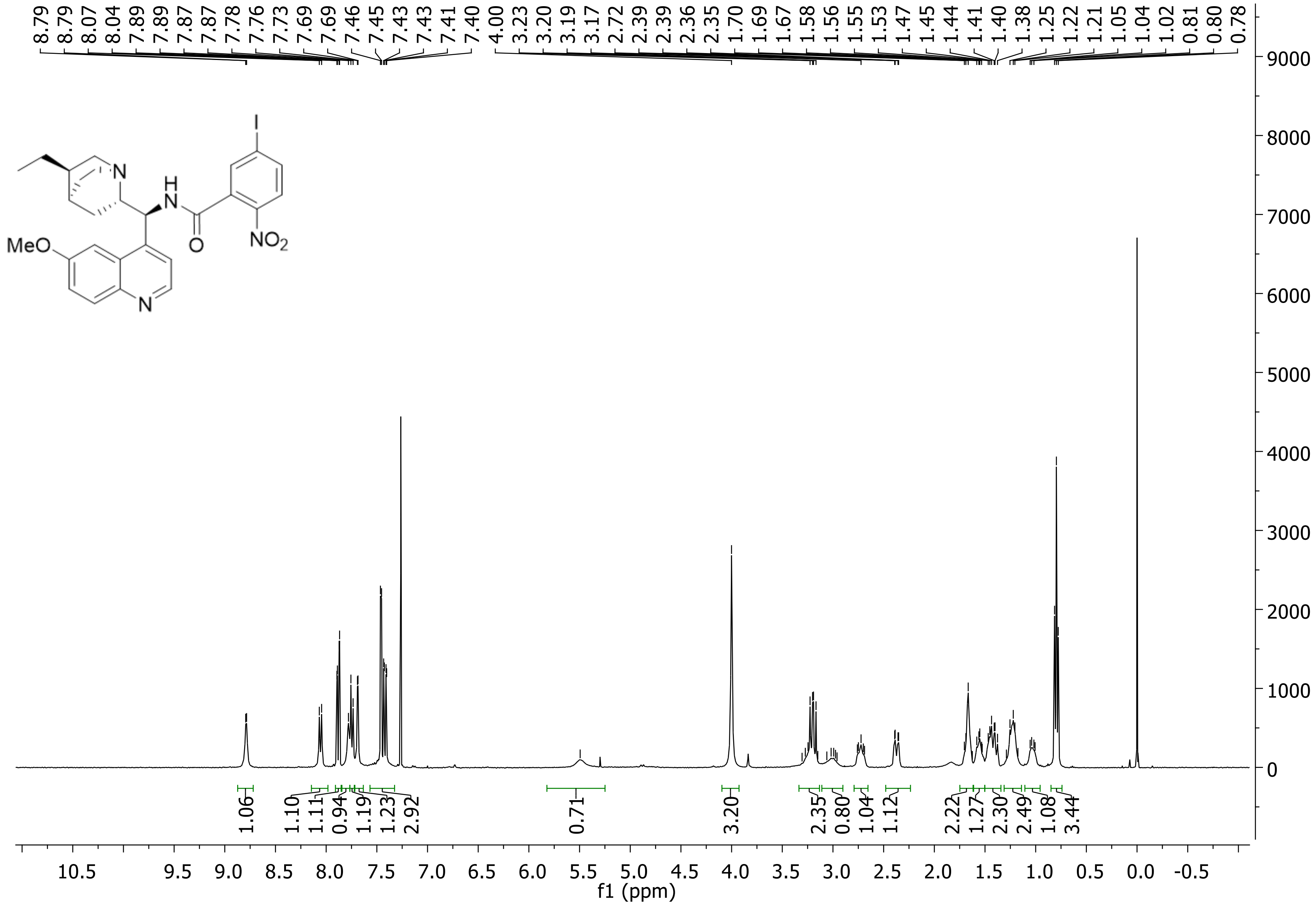

Figure S66. ^{1}H NMR spectrum of catalyst **E** ($CDCl_3$, 400 MHz).

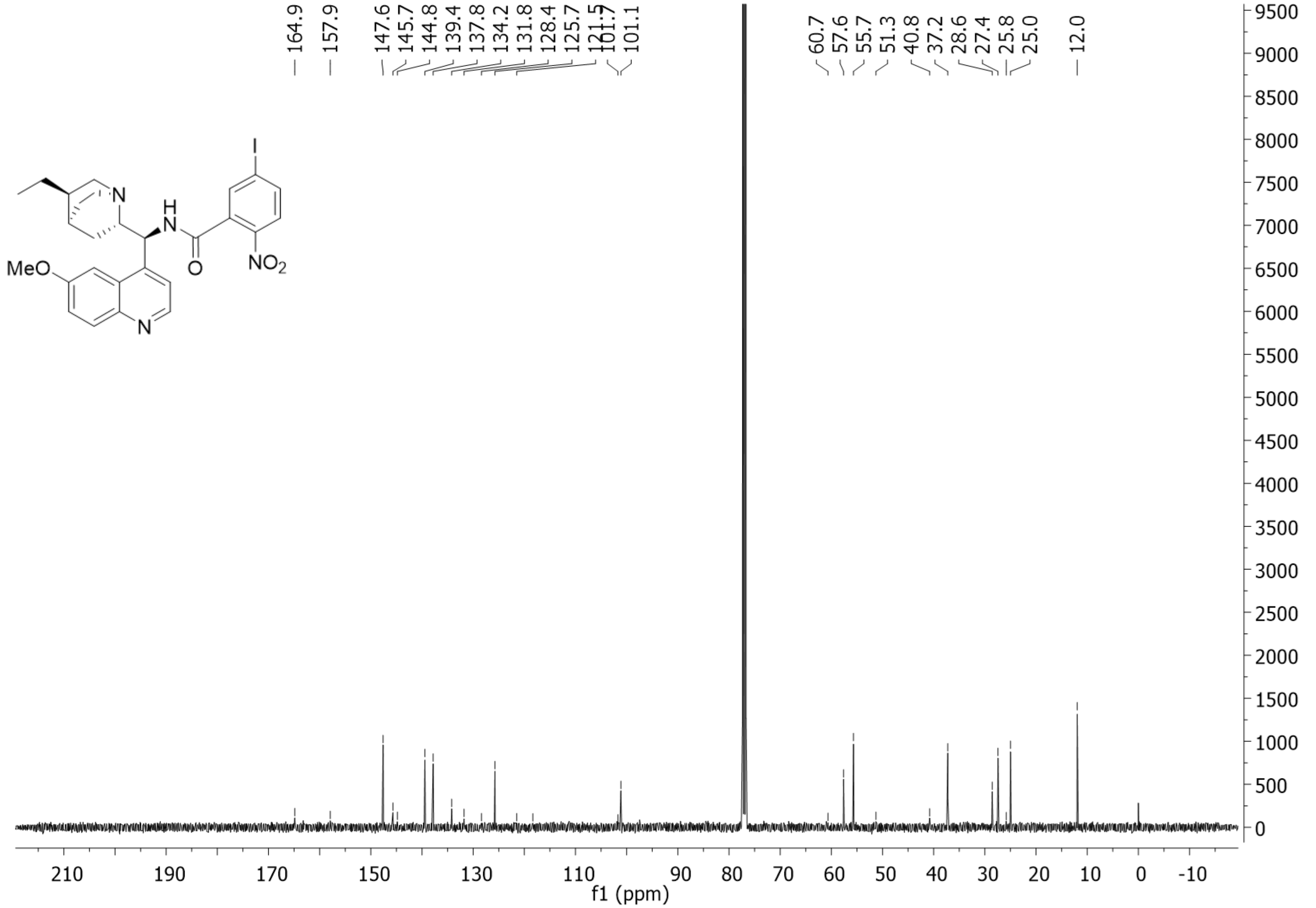

Figure S67. $^{13}C\{^{1}H\}$ NMR spectrum of catalyst **E** ($CDCl_3$, 101 MHz).

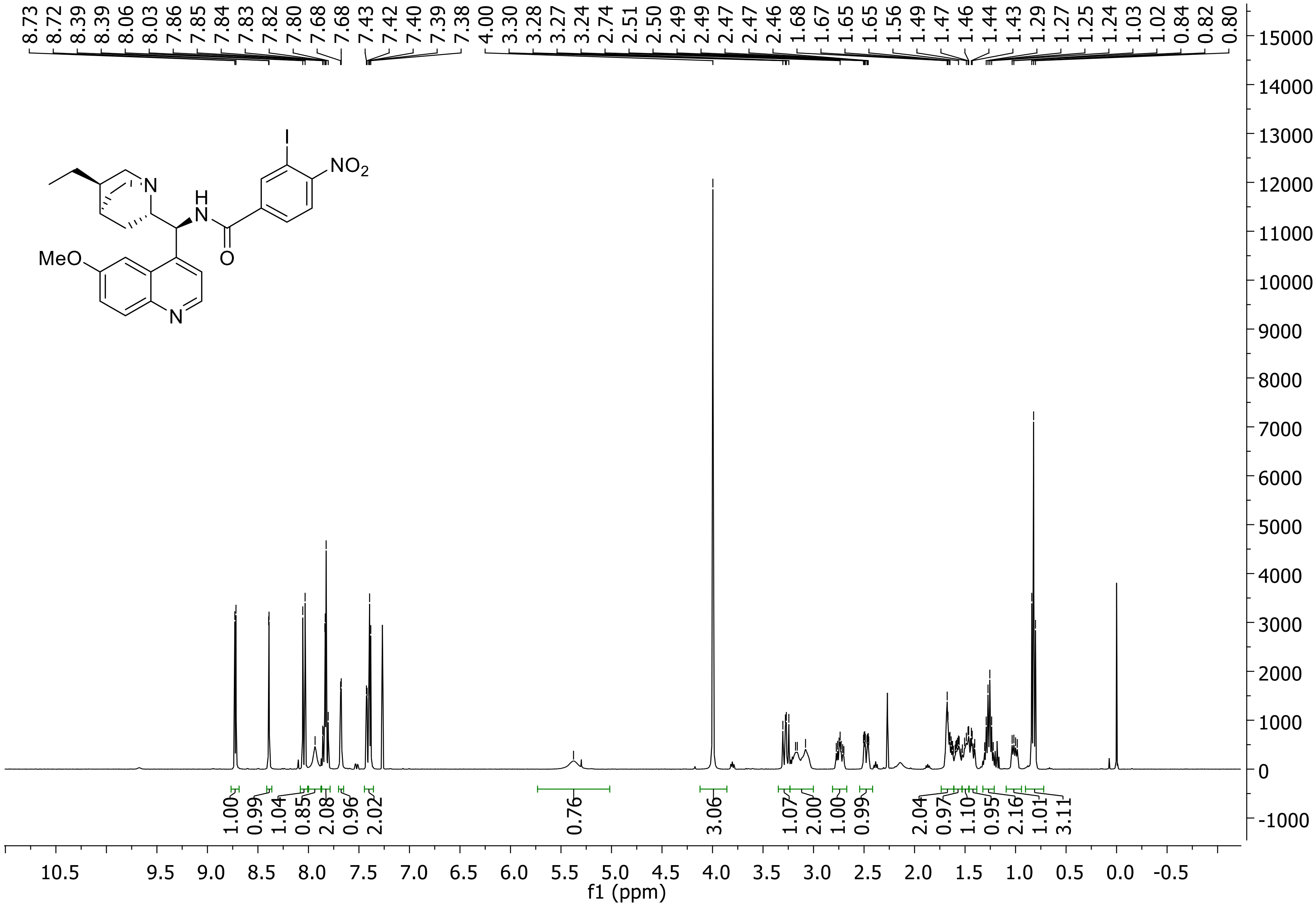

Figure S68. ¹H NMR spectrum of catalyst **F** ($CDCl_3$, 400 MHz).

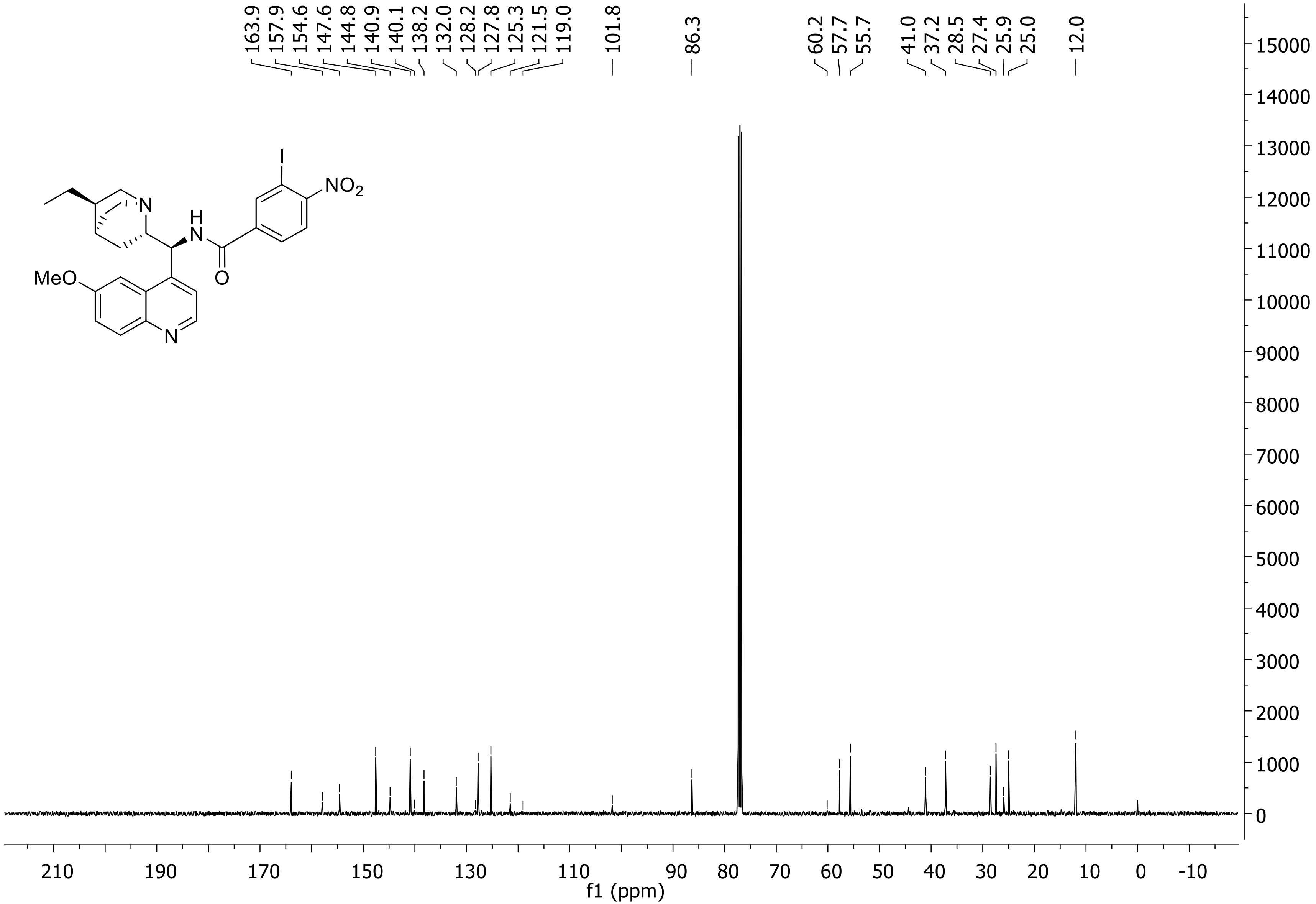

Figure S69. $^{13}C\{^1H\}$ NMR spectrum of catalyst **F** ($CDCl_3$, 101 MHz).

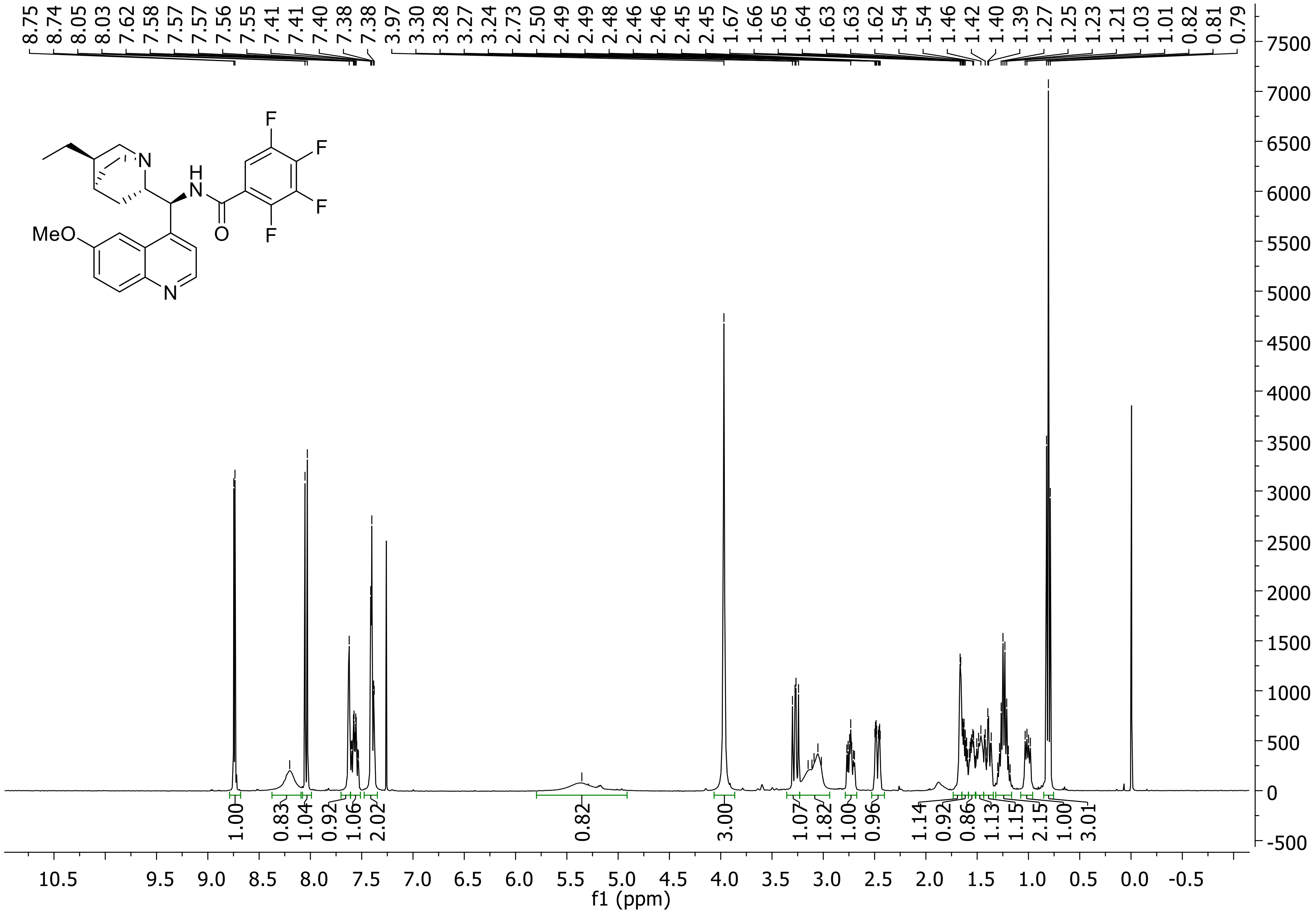

Figure S70. ^{1}H NMR spectrum of catalyst **G** ($CDCl_3$, 400 MHz).

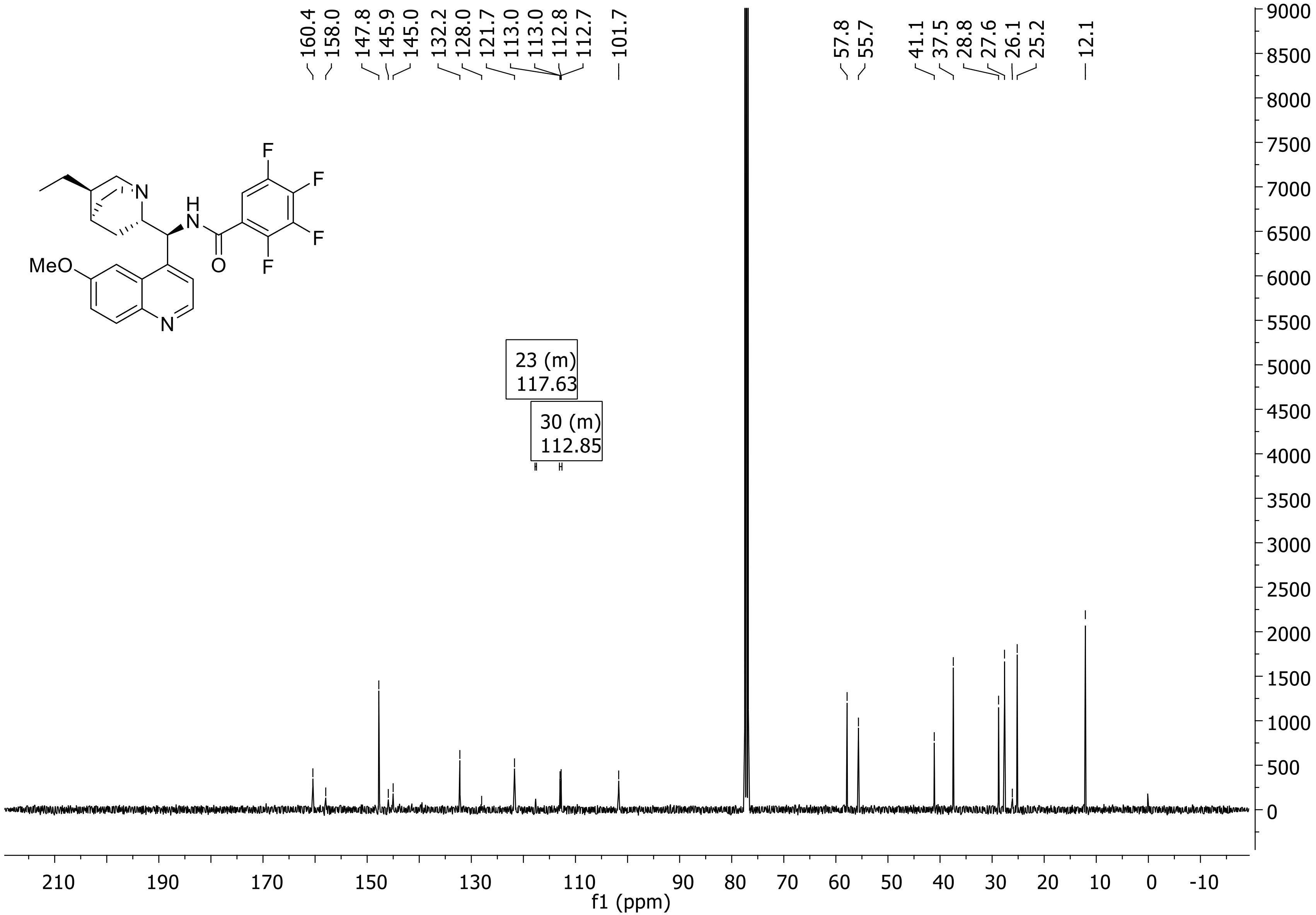

Figure S71. $^{13}C\{^1H\}$ NMR spectrum of catalyst **G** ($CDCl_3$, 101 MHz).

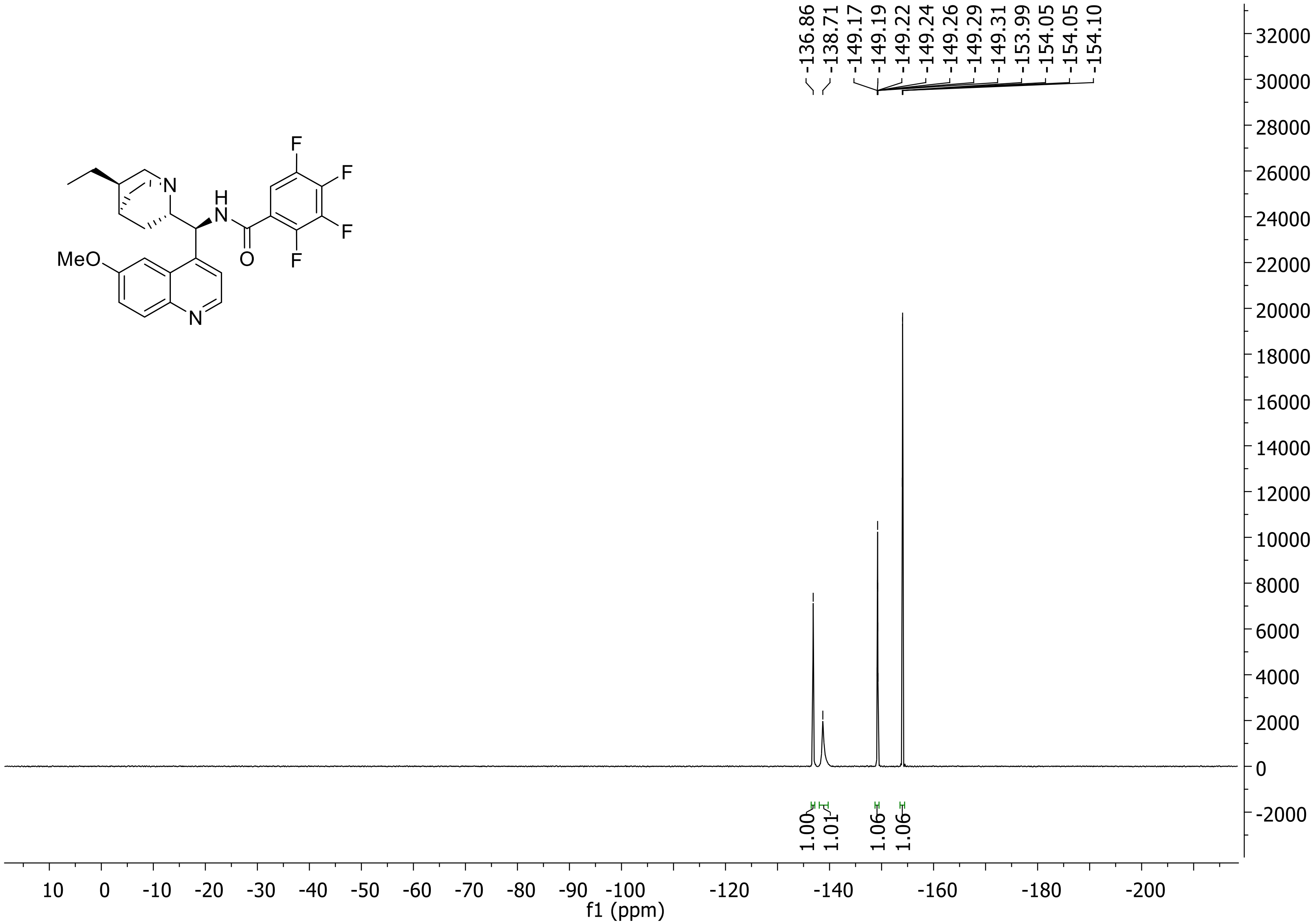

Figure S72. ^{19}F NMR spectrum of catalyst **G** ($CDCl_3$, 376 MHz).

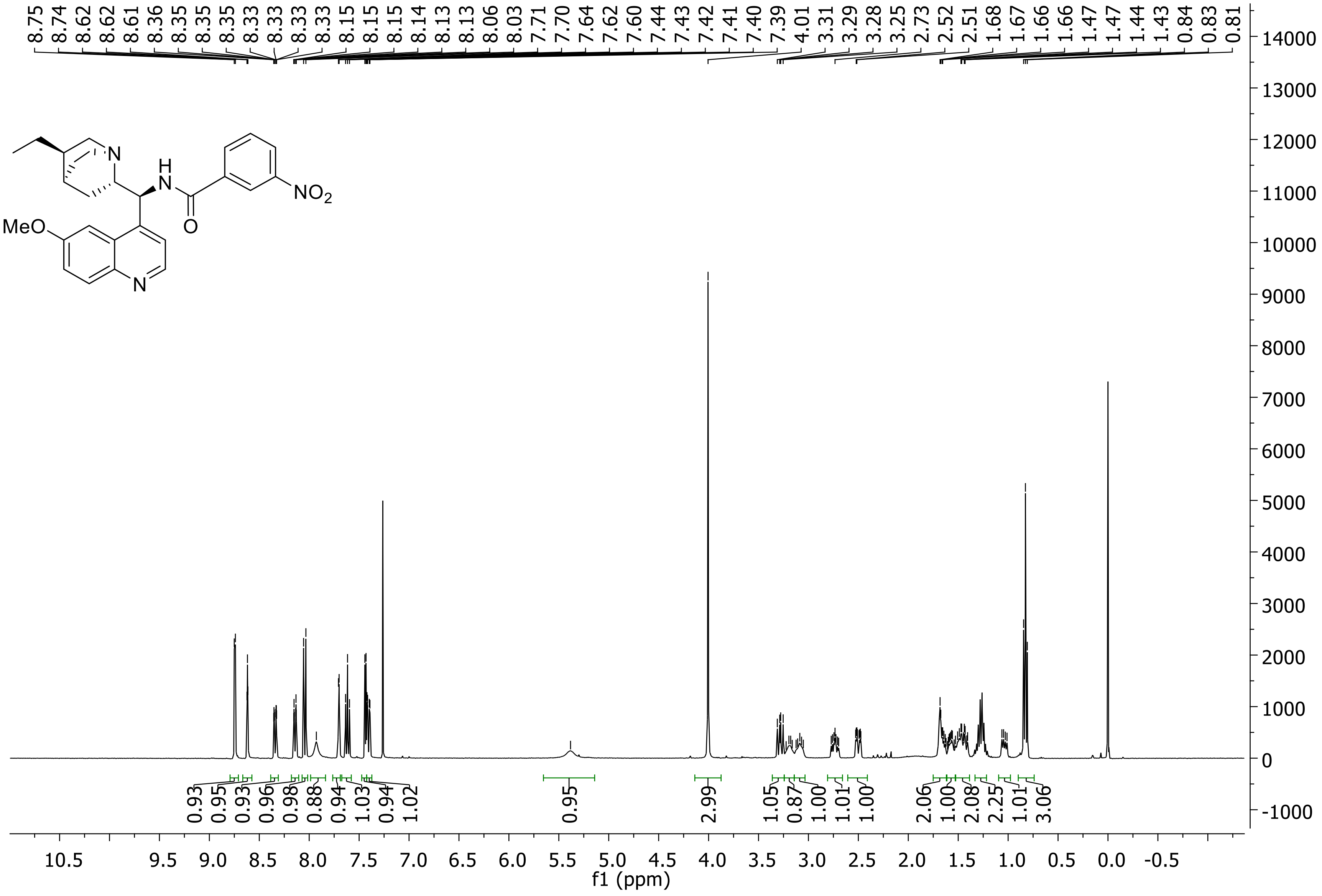

Figure S73. ^{1}H NMR spectrum of catalyst **H** ($CDCl_3$, 400 MHz).

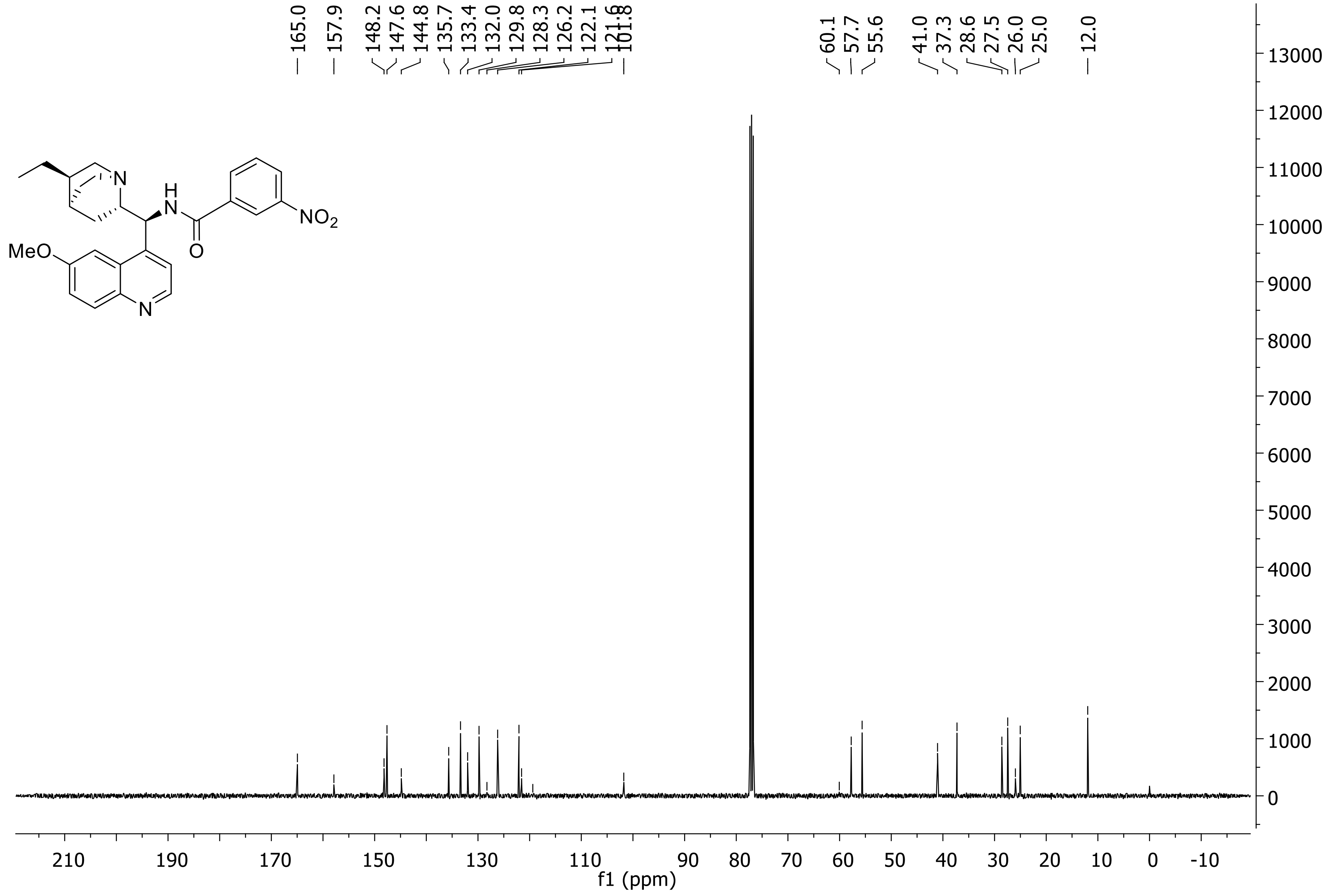

Figure S74. $^{13}C\{^{1}H\}$ NMR spectrum of catalyst **H** ($CDCl_3$, 101 MHz).

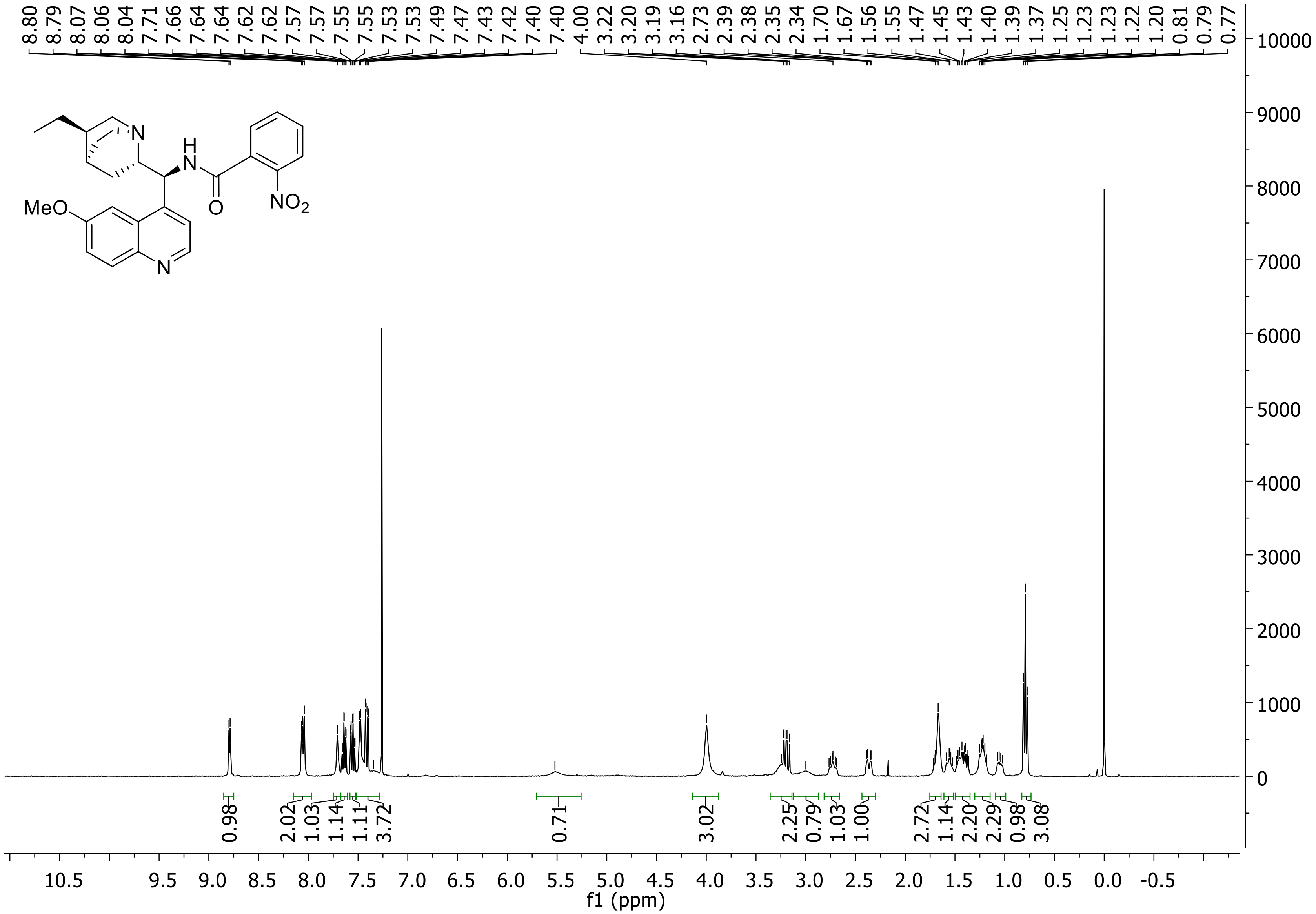

Figure S75. ^{1}H NMR spectrum of catalyst **I** ($CDCl_3$, 400 MHz).

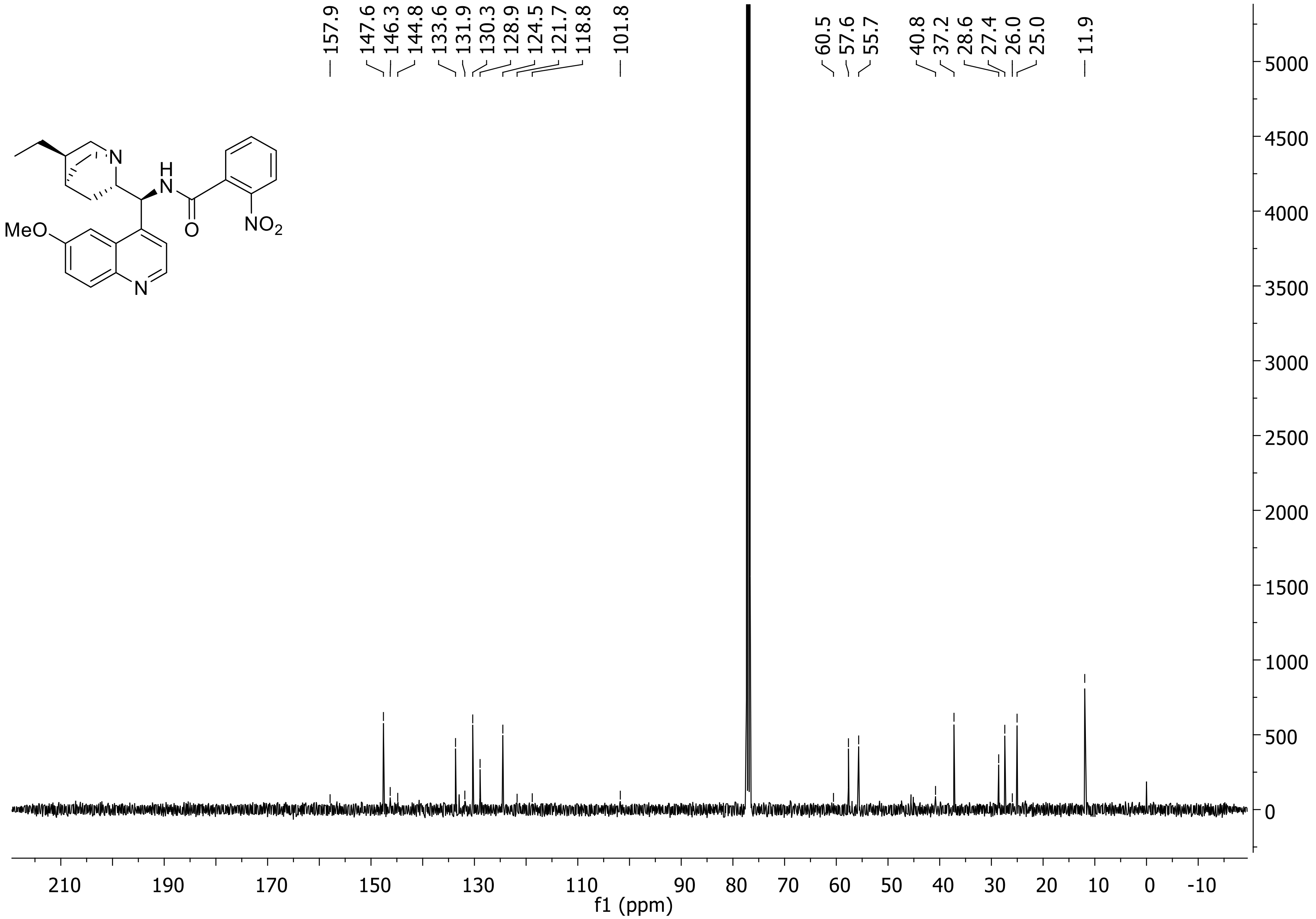

Figure S76. $^{13}C\{^1H\}$ NMR spectrum of catalyst **I** ($CDCl_3$, 101 MHz).

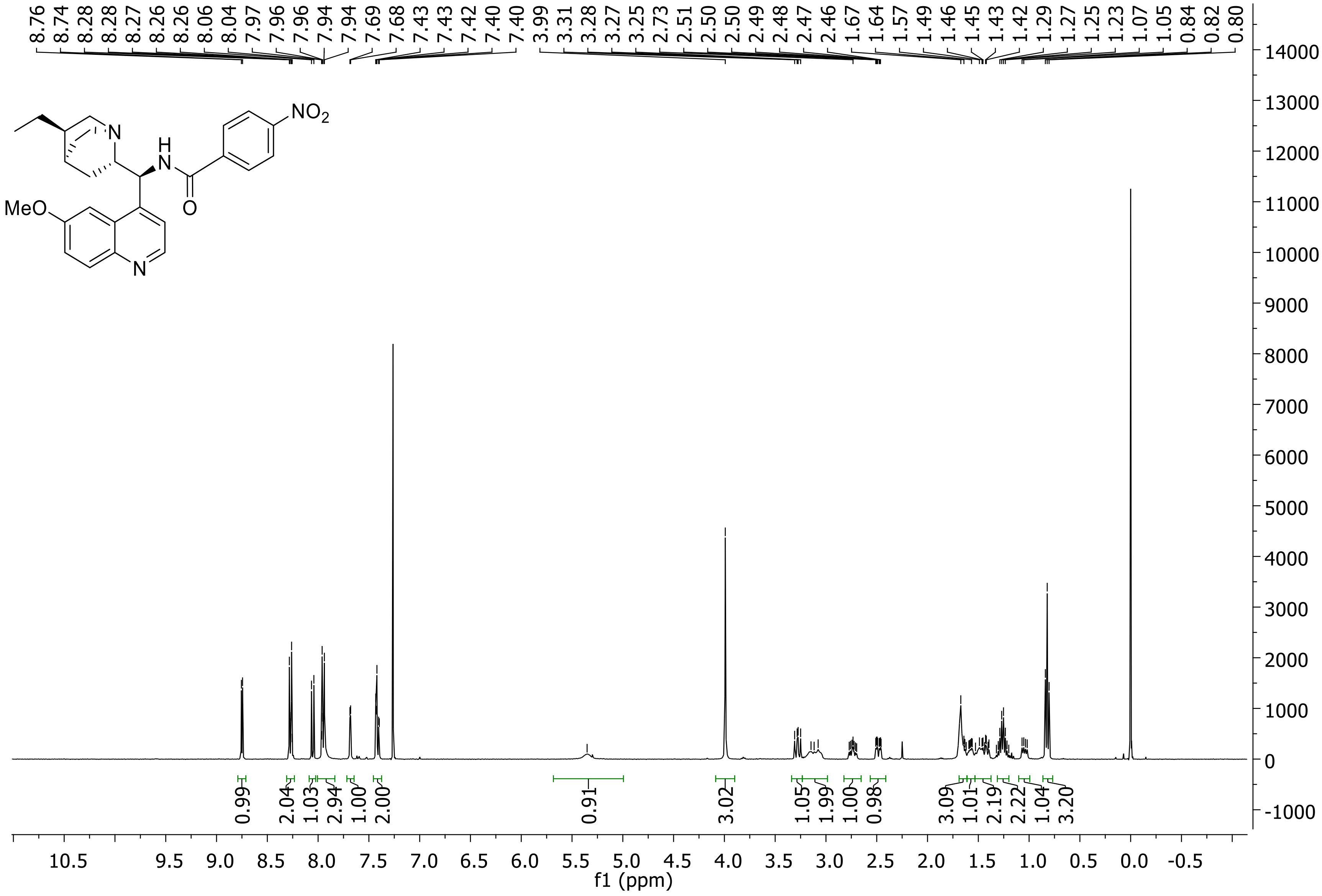

Figure S77. ^{1}H NMR spectrum of catalyst **J** ($CDCl_3$, 400 MHz).

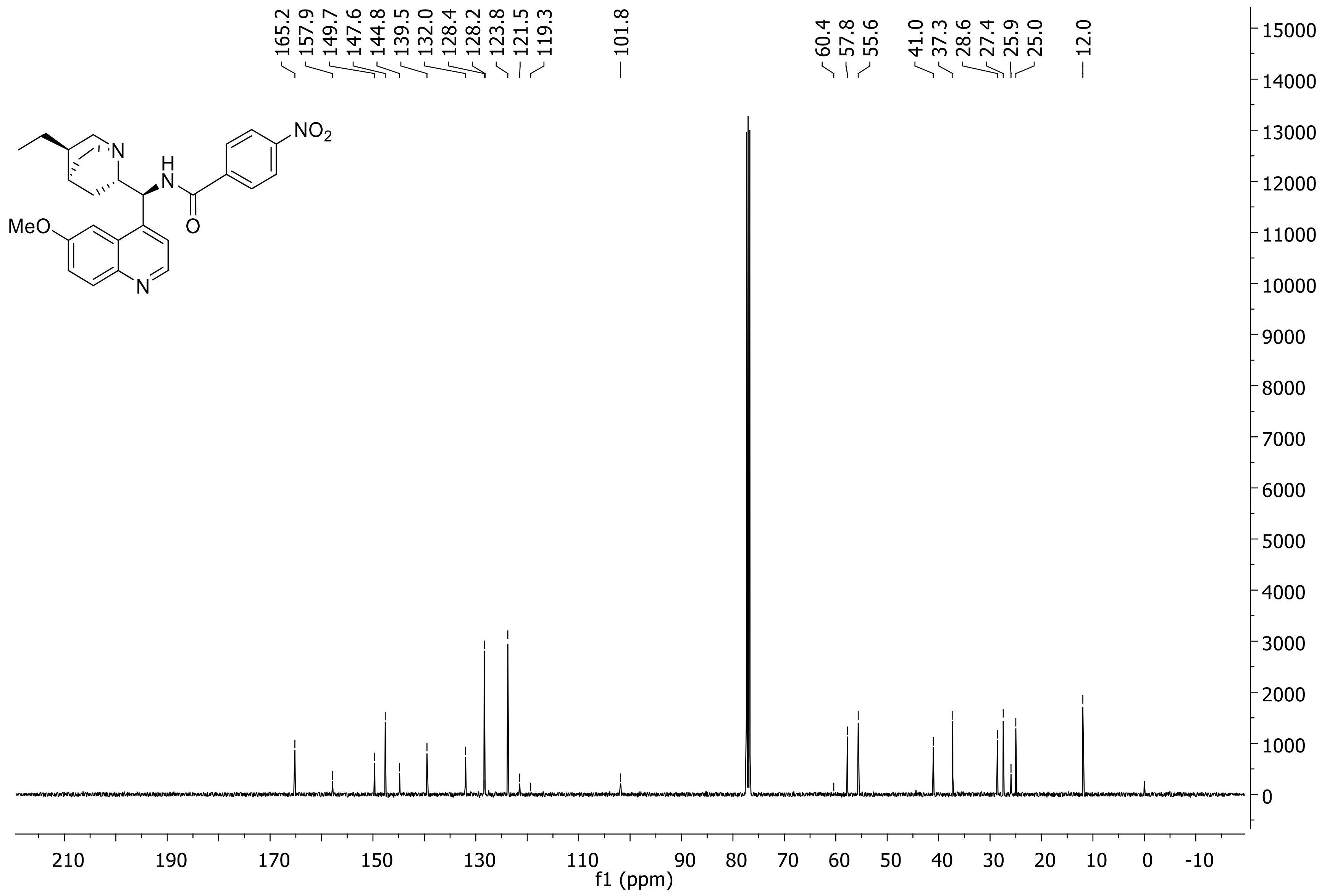

Figure S78. $^{13}C\{^1H\}$ NMR spectrum of catalyst **J** ($CDCl_3$, 101 MHz).

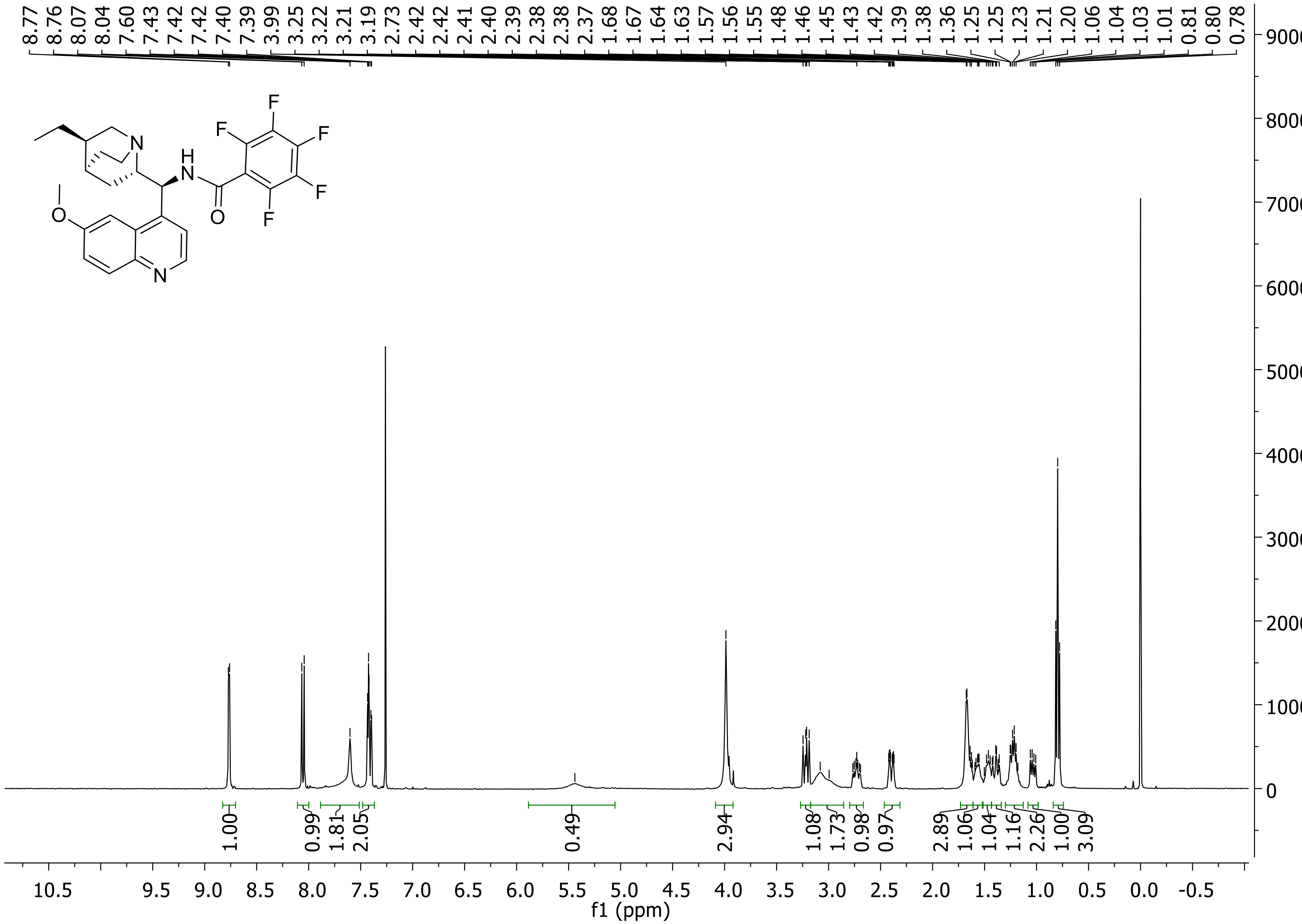

Figure S79. ^{1}H NMR spectrum of catalyst **K** ($CDCl_3$, 400 MHz).

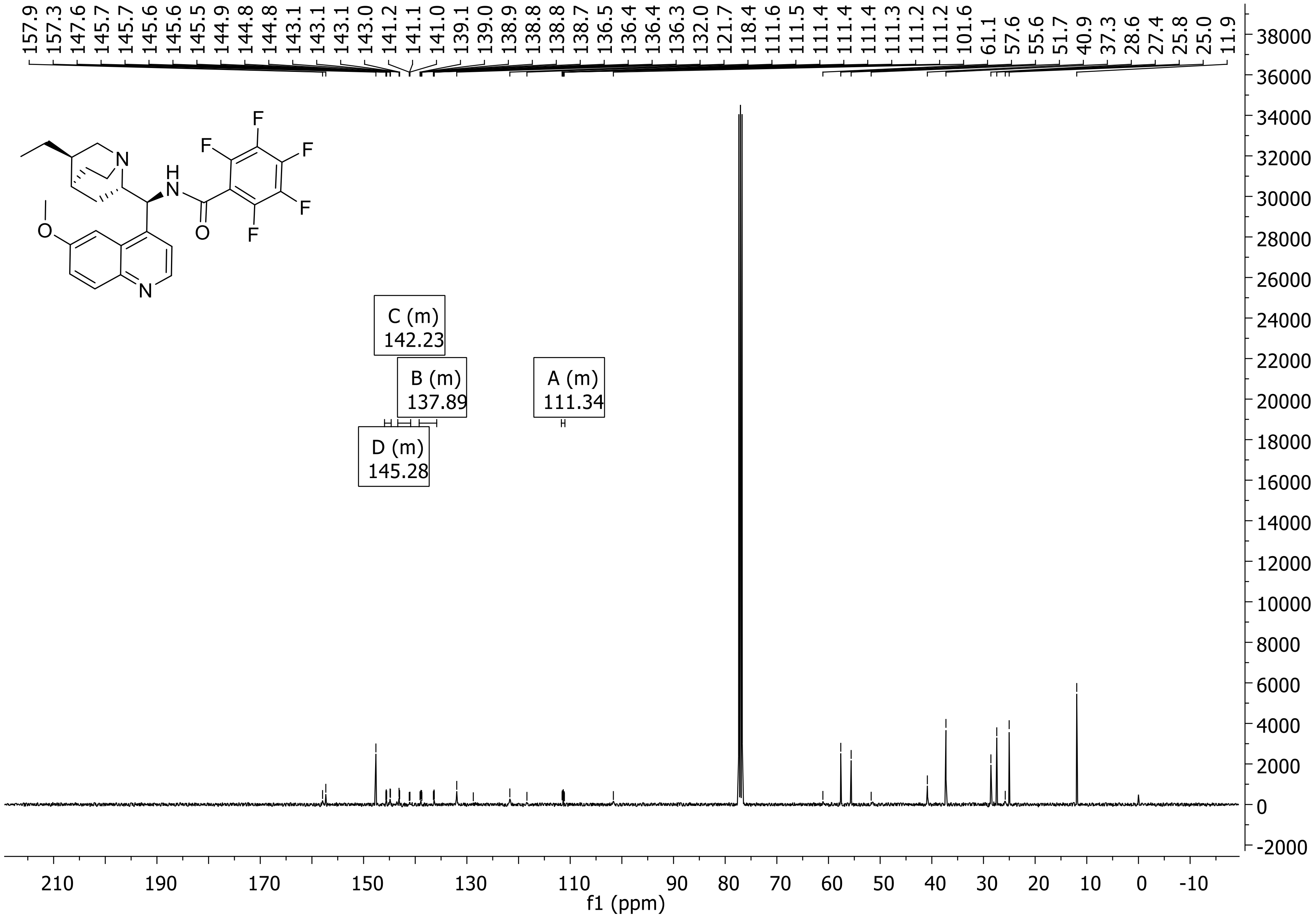

Figure S80. $^{13}C\{^1H\}$ NMR spectrum of catalyst **K** ($CDCl_3$, 101 MHz).

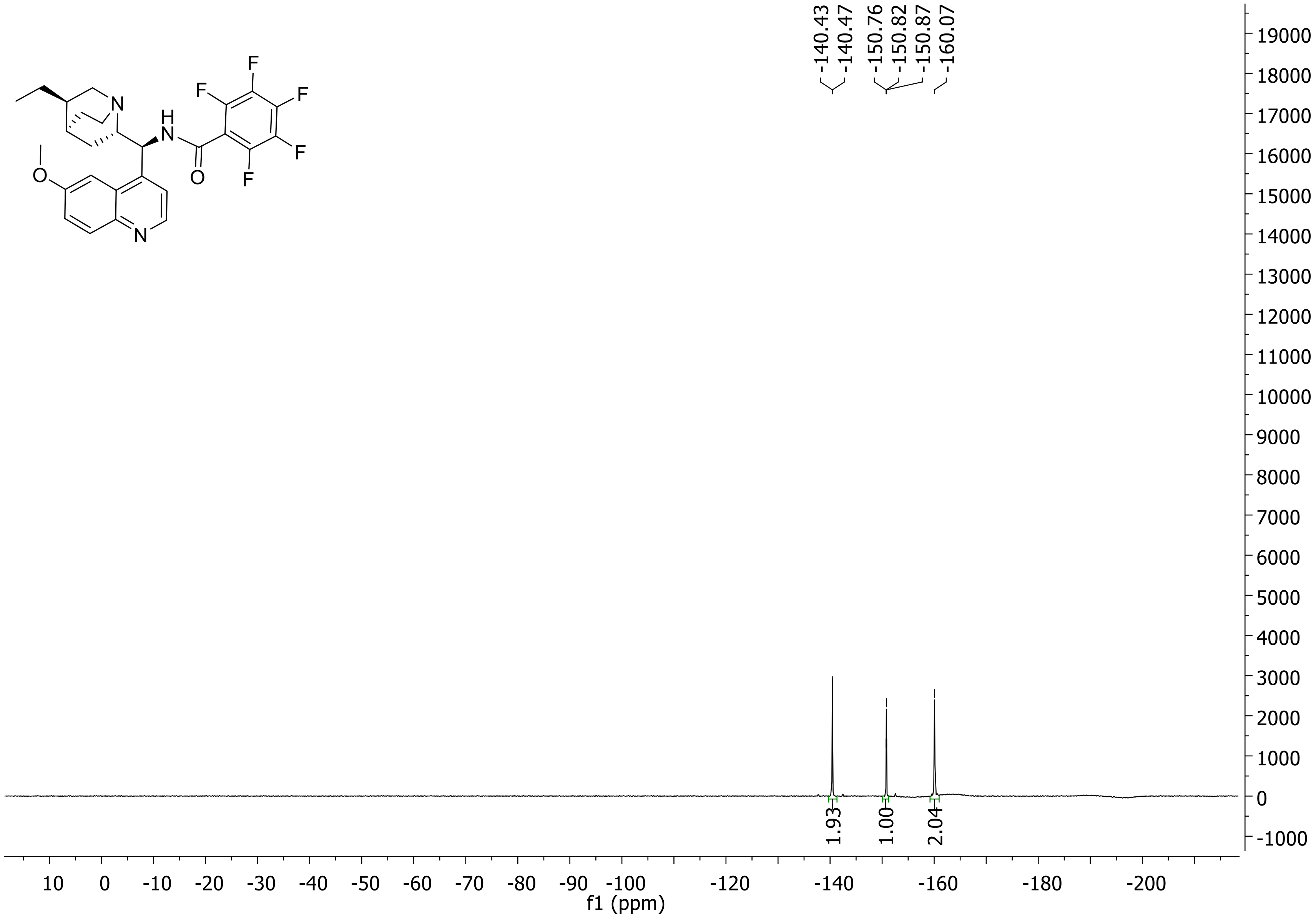

Figure S81. ^{19}F NMR spectrum of catalyst **K** ($CDCl_3$, 376 MHz).

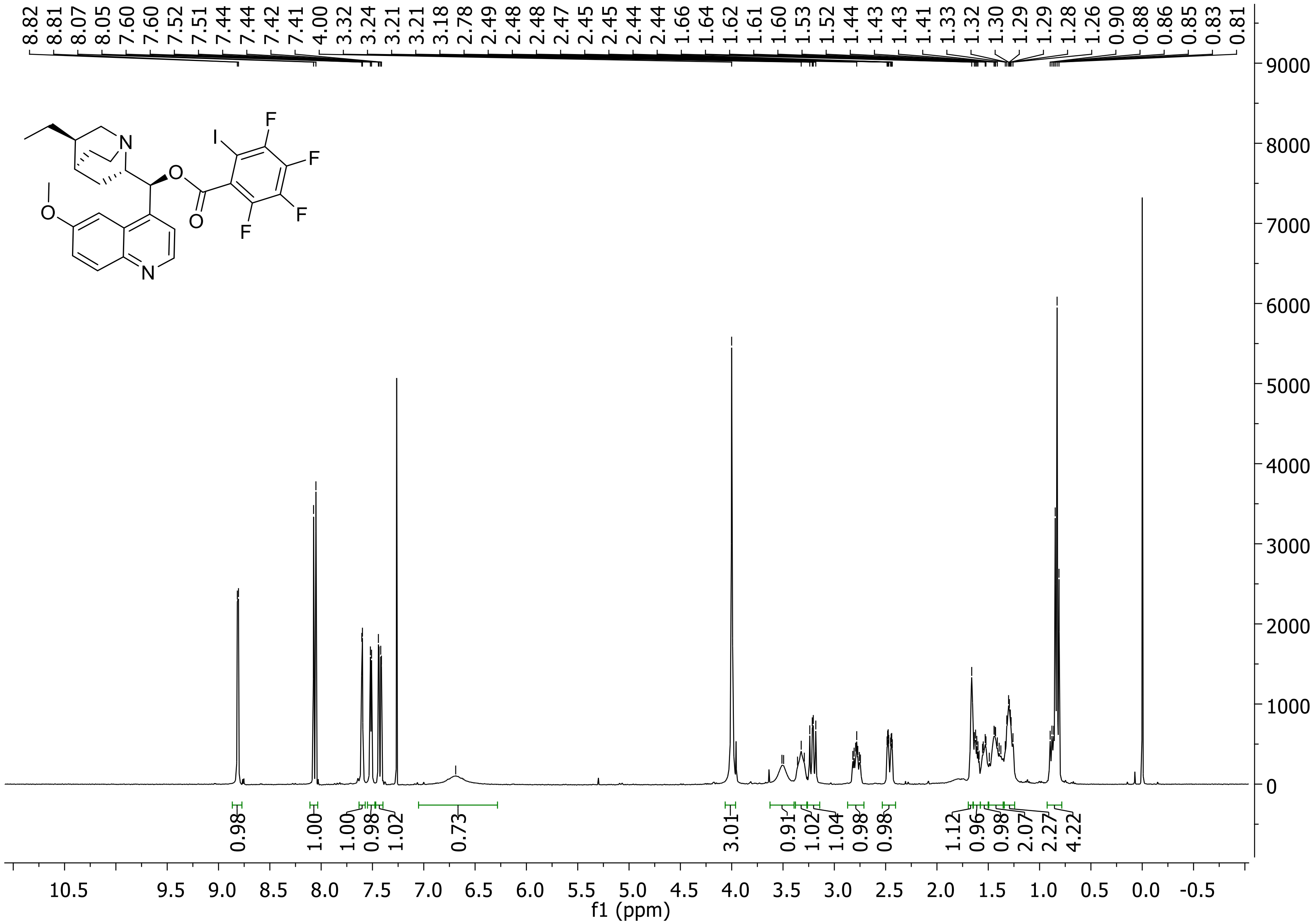

Figure S82. ^{1}H NMR spectrum of catalyst **L** ($CDCl_3$, 400 MHz).

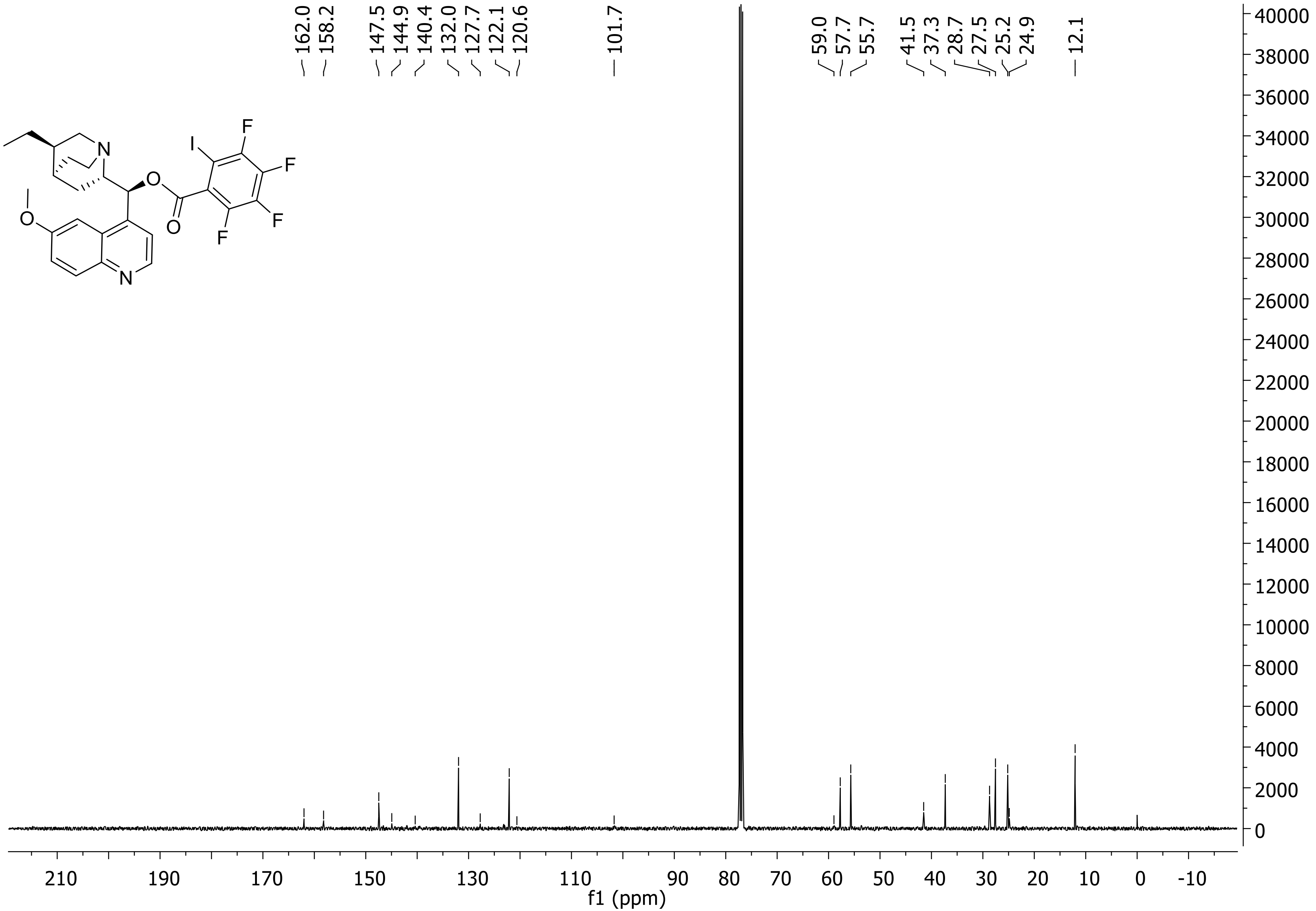

Figure S83. $^{13}C\{^1H\}$ NMR spectrum of catalyst **L** ($CDCl_3$, 101 MHz).

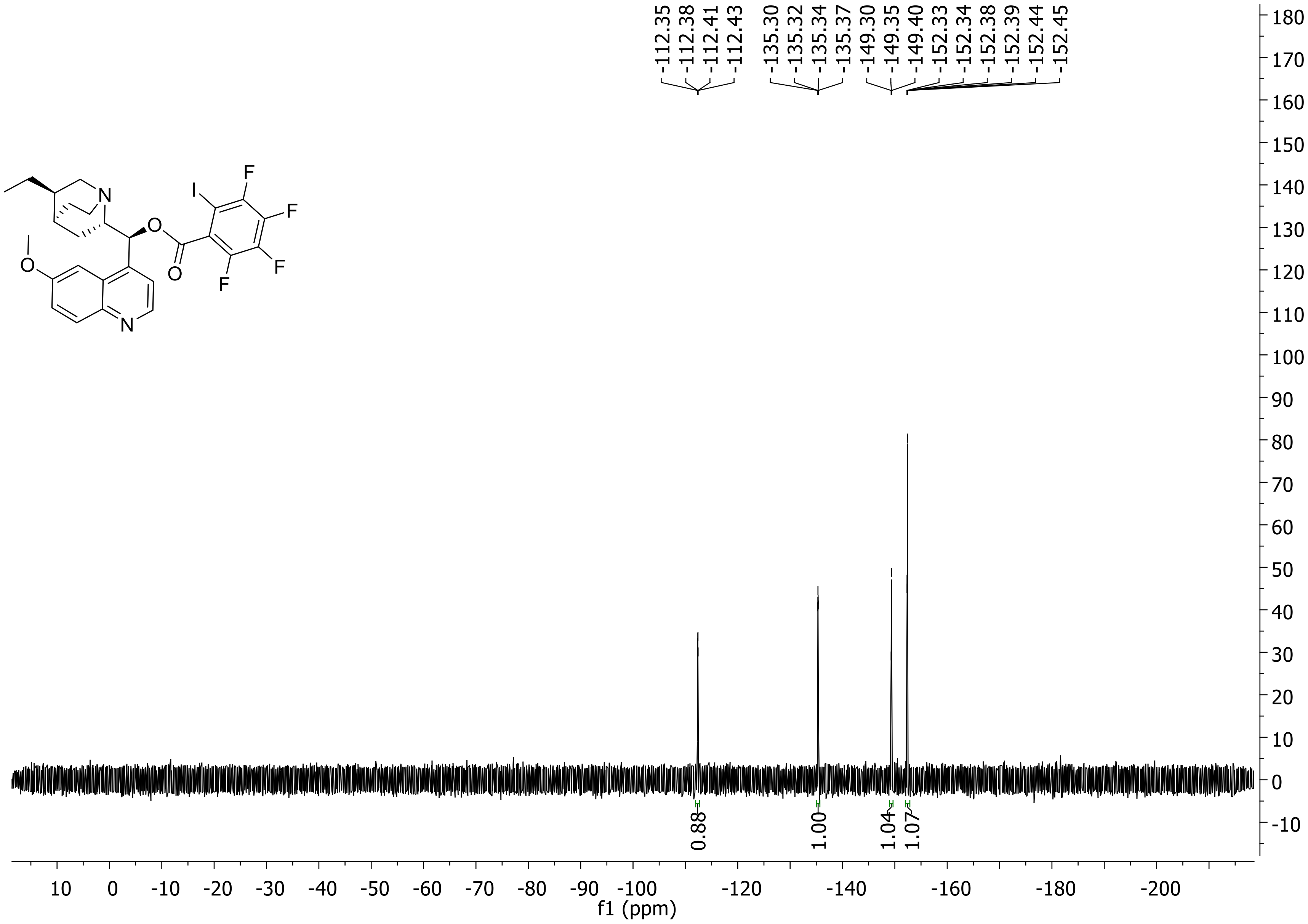

Figure S84. ^{19}F NMR spectrum of catalyst **L** ($CDCl_3$, 376 MHz).

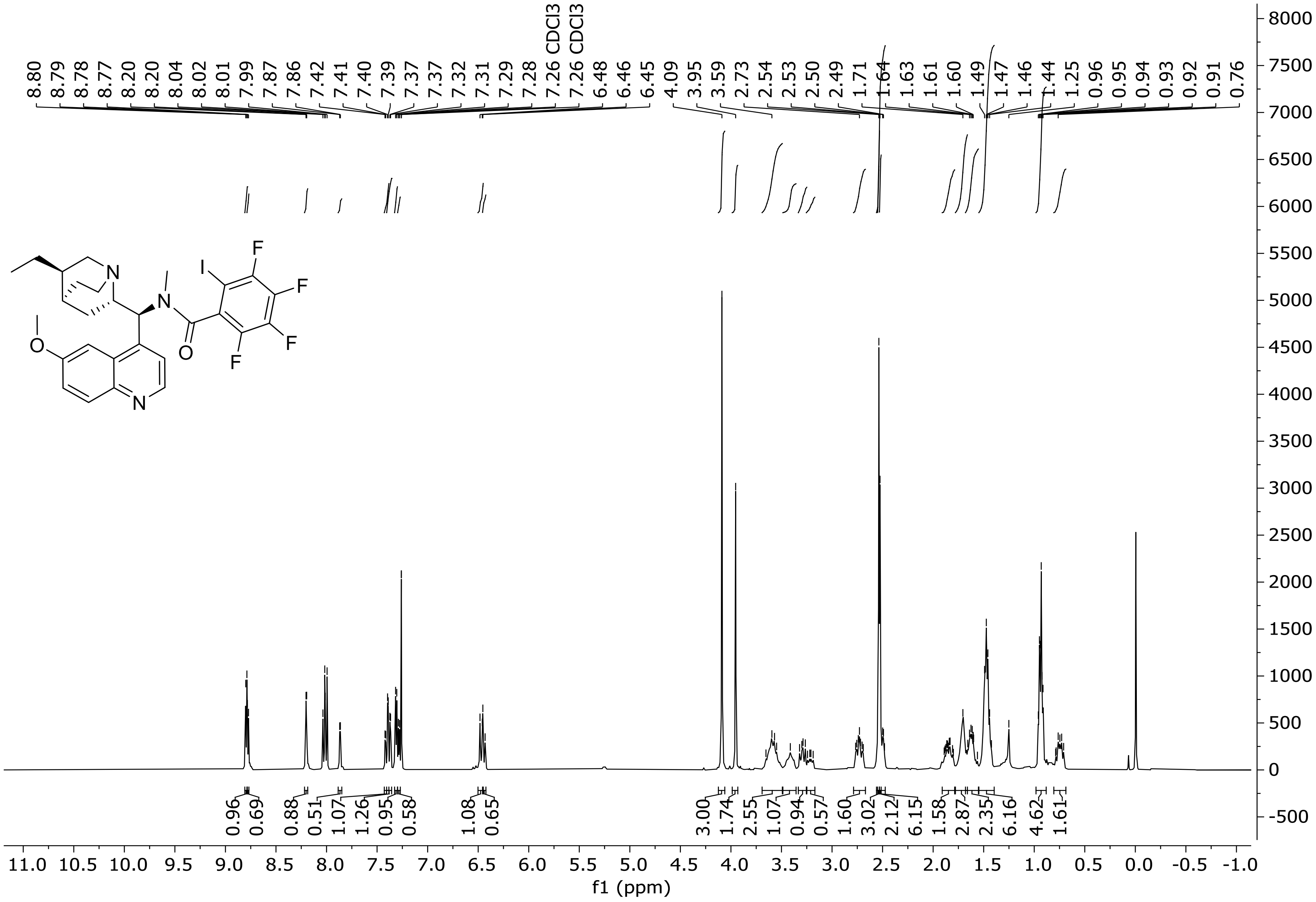

Figure S85. ^{1}H NMR spectrum of catalyst **M** ($CDCl_3$, 400 MHz).

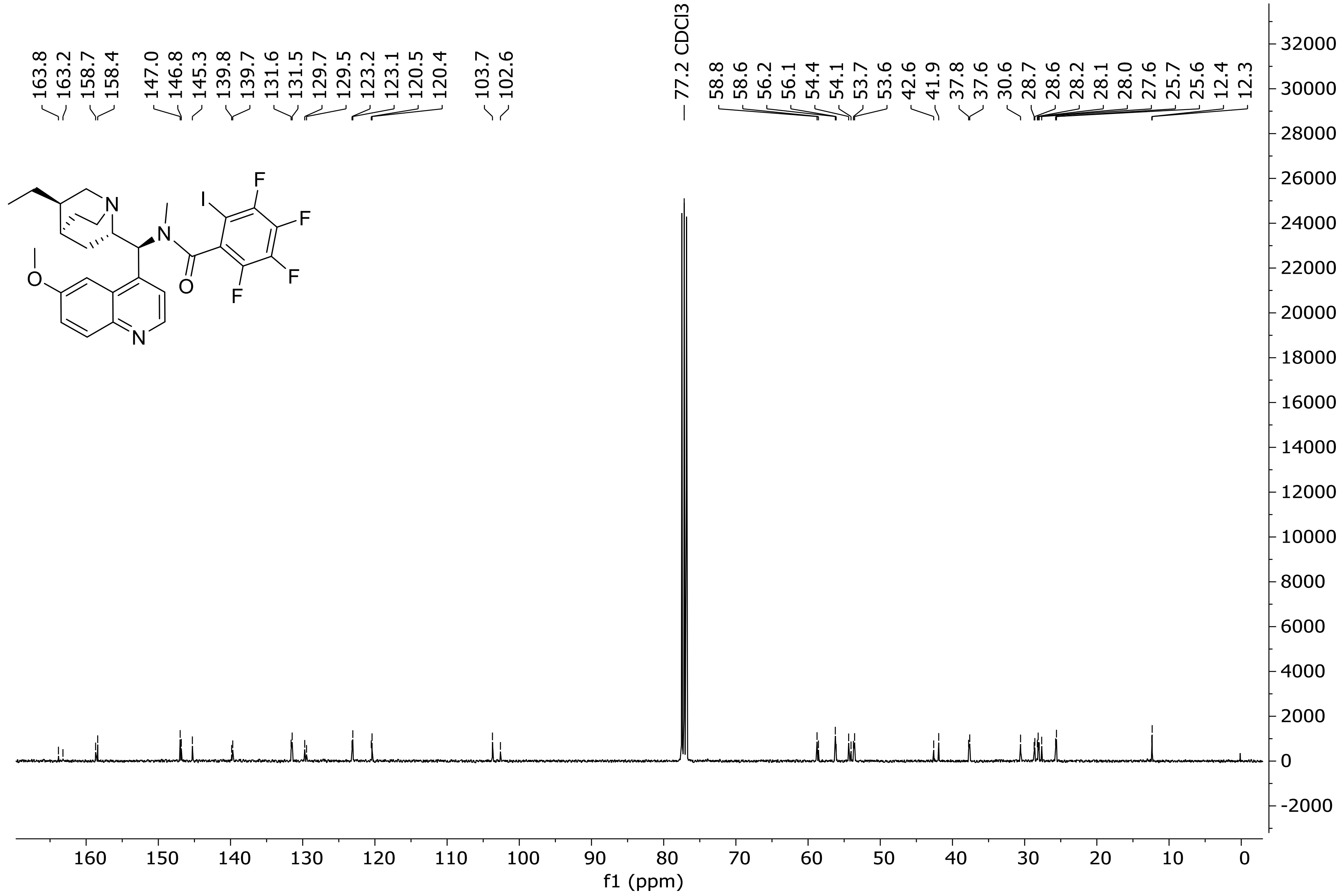

Figure S86. $^{13}C\{^1H\}$ NMR spectrum of catalyst **M** ($CDCl_3$, 101 MHz).

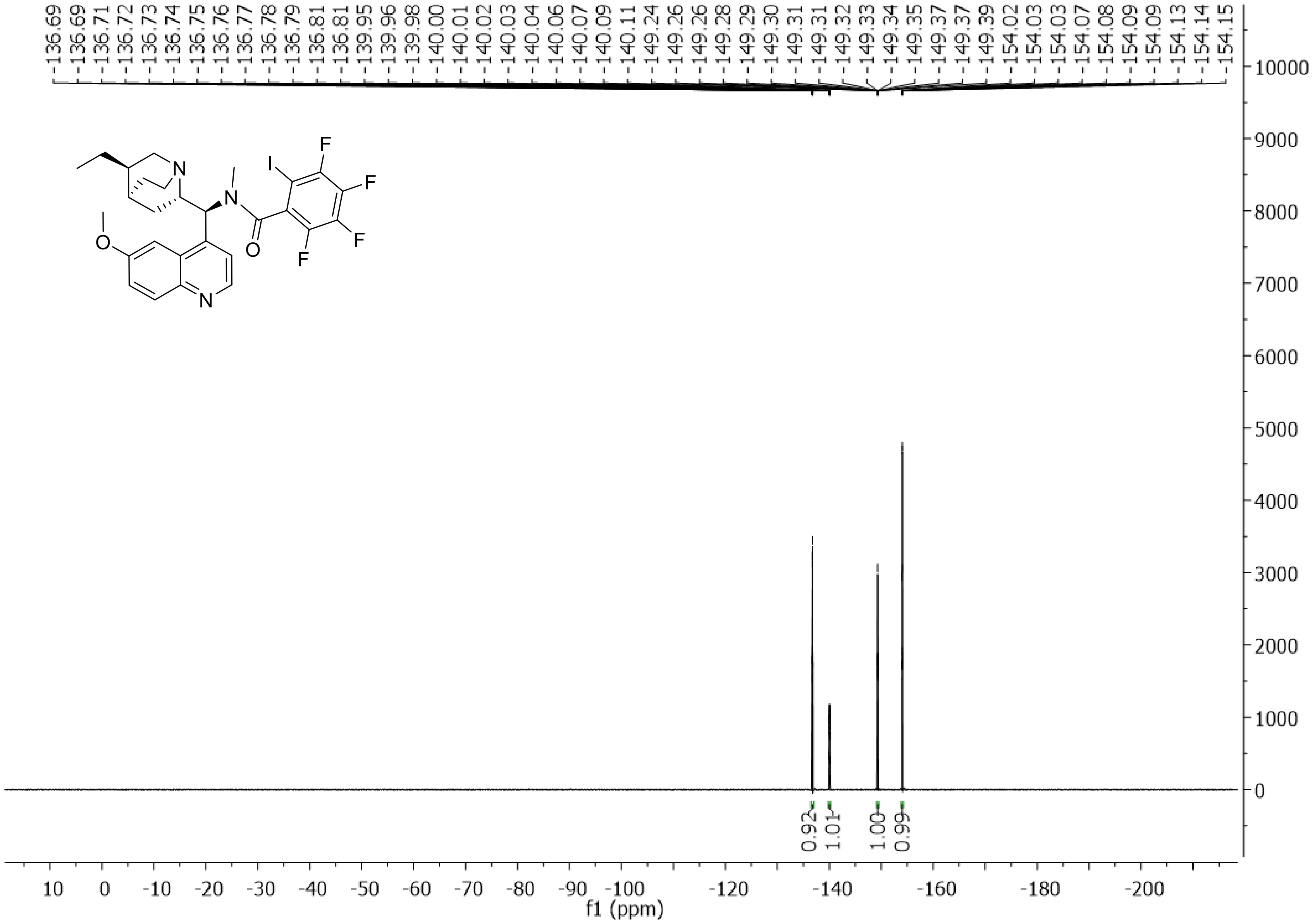

Figure S87. ^{19}F NMR spectrum of compound catalyst **M** ($CDCl_3$, 376 MHz).

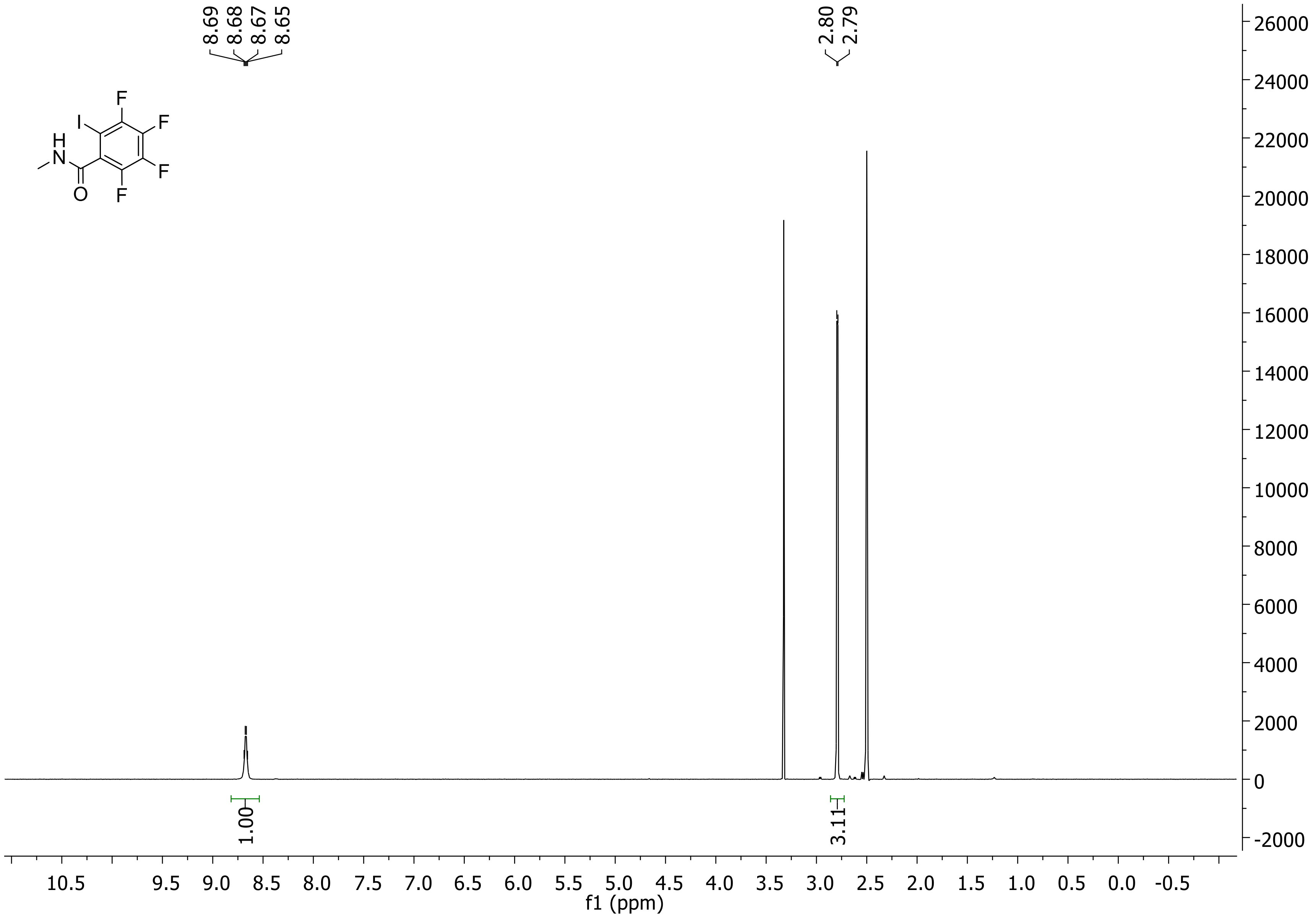

Figure S88. ^{1}H NMR spectrum of compound **9** ([D_6]DMSO, 400 MHz).

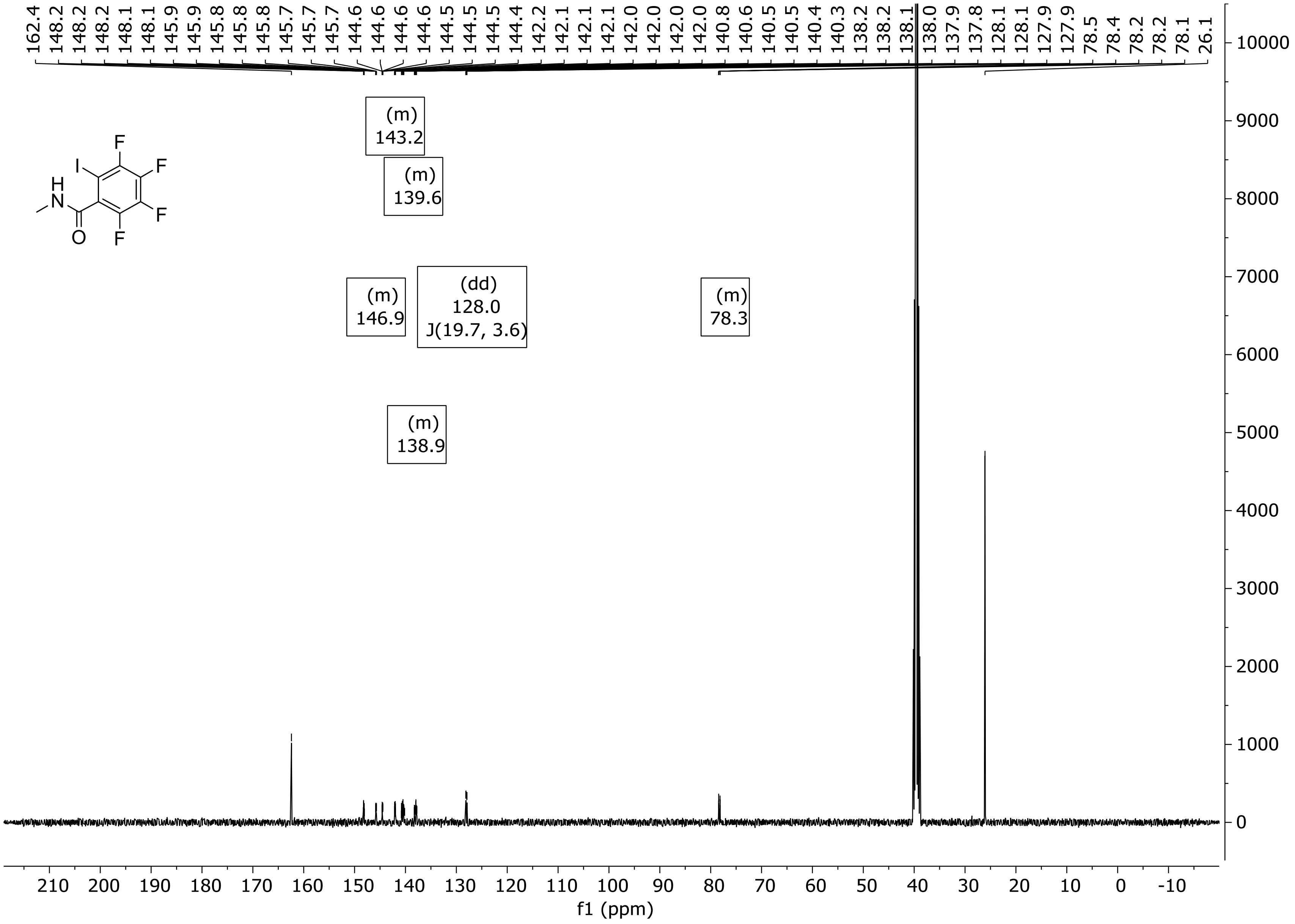

Figure S89. $^{13}C\{^{1}H\}$ NMR spectrum of compound **9** ([D_6]DMSO, 101 MHz).

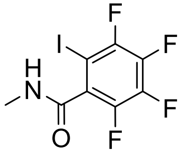

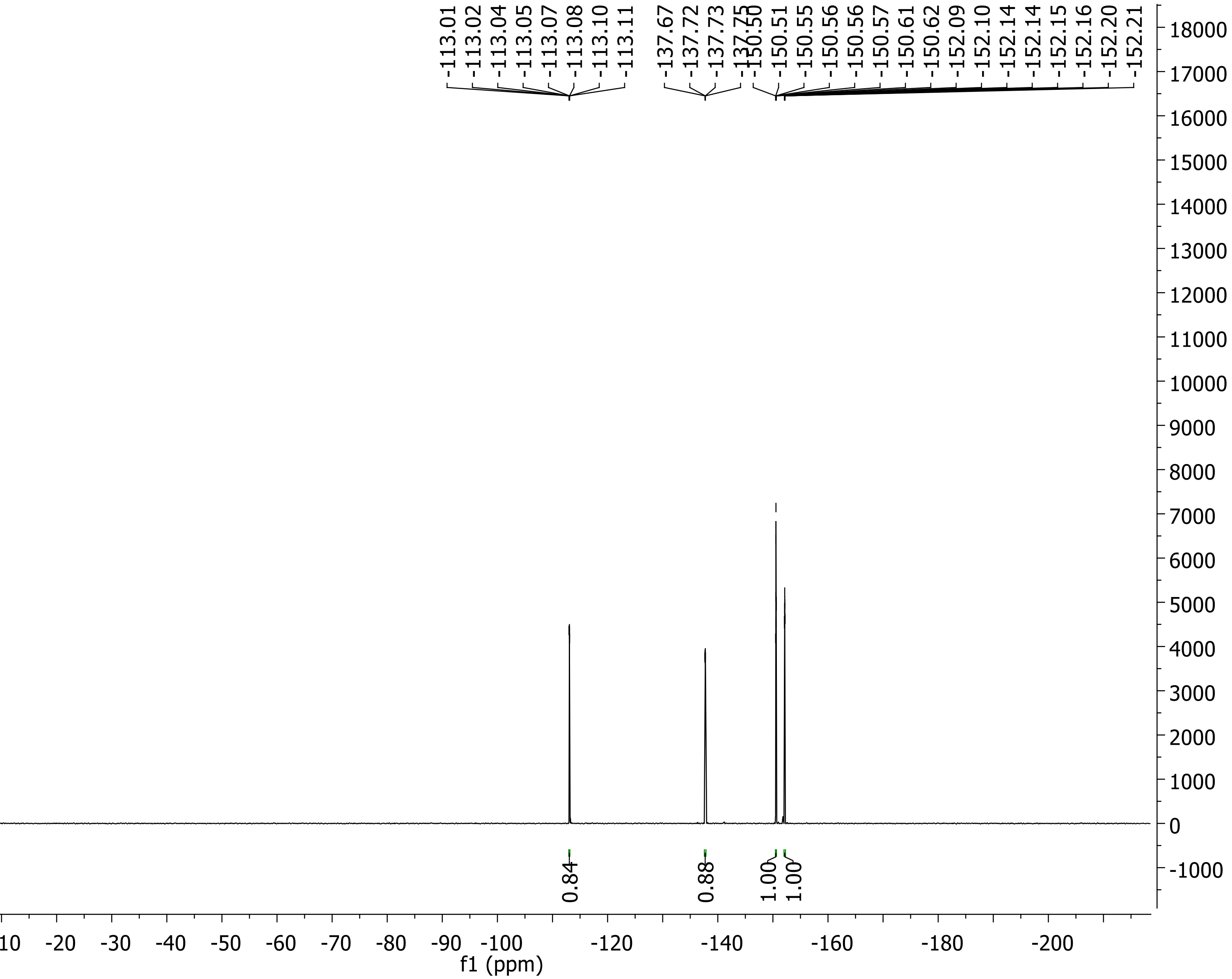

Figure S90. ^{19}F NMR spectrum of compound **9** ([D_6]DMSO, 376 MHz).

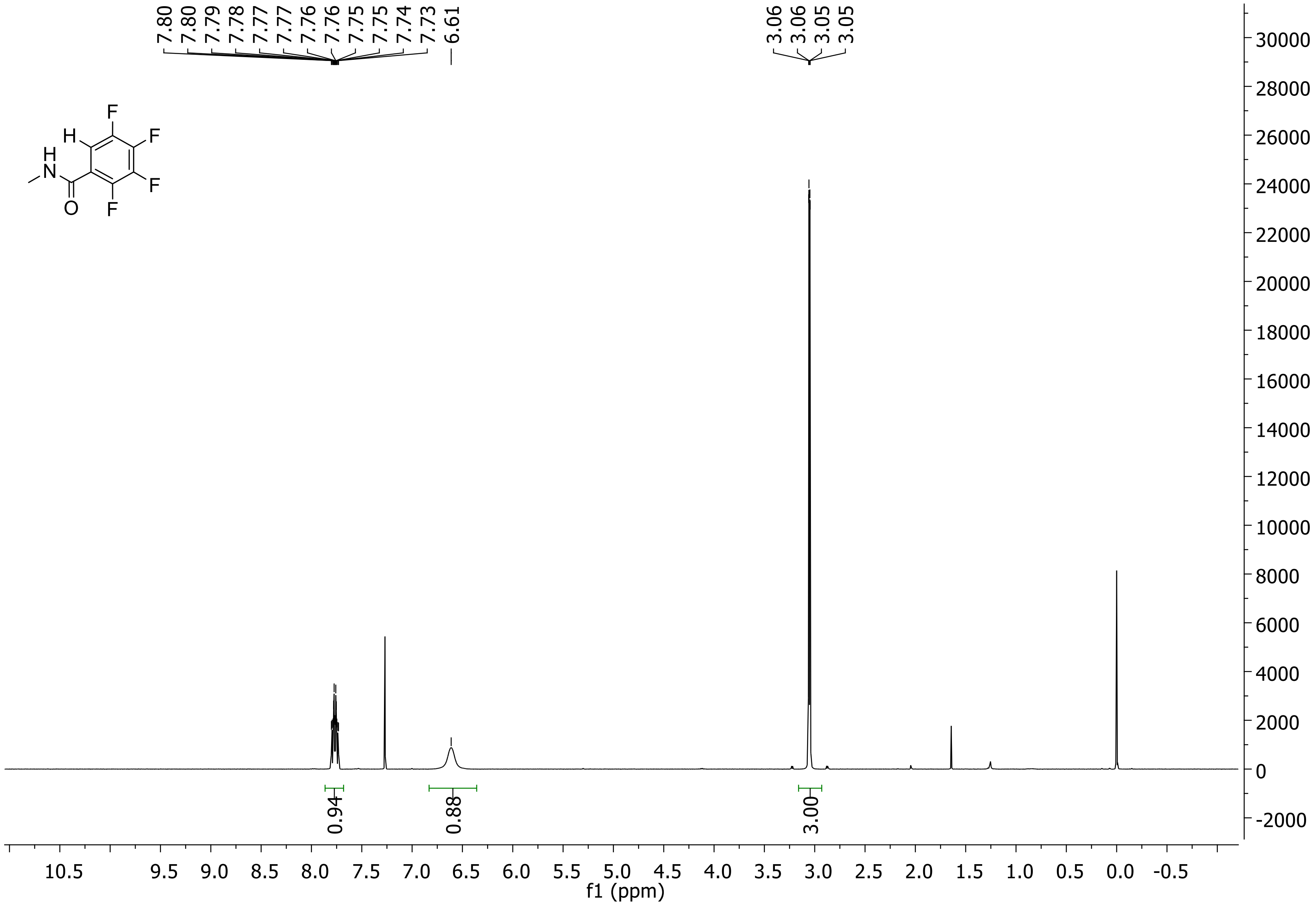

Figure S91. ^{1}H NMR spectrum of compound **10** ($CDCl_3$, 400 MHz).

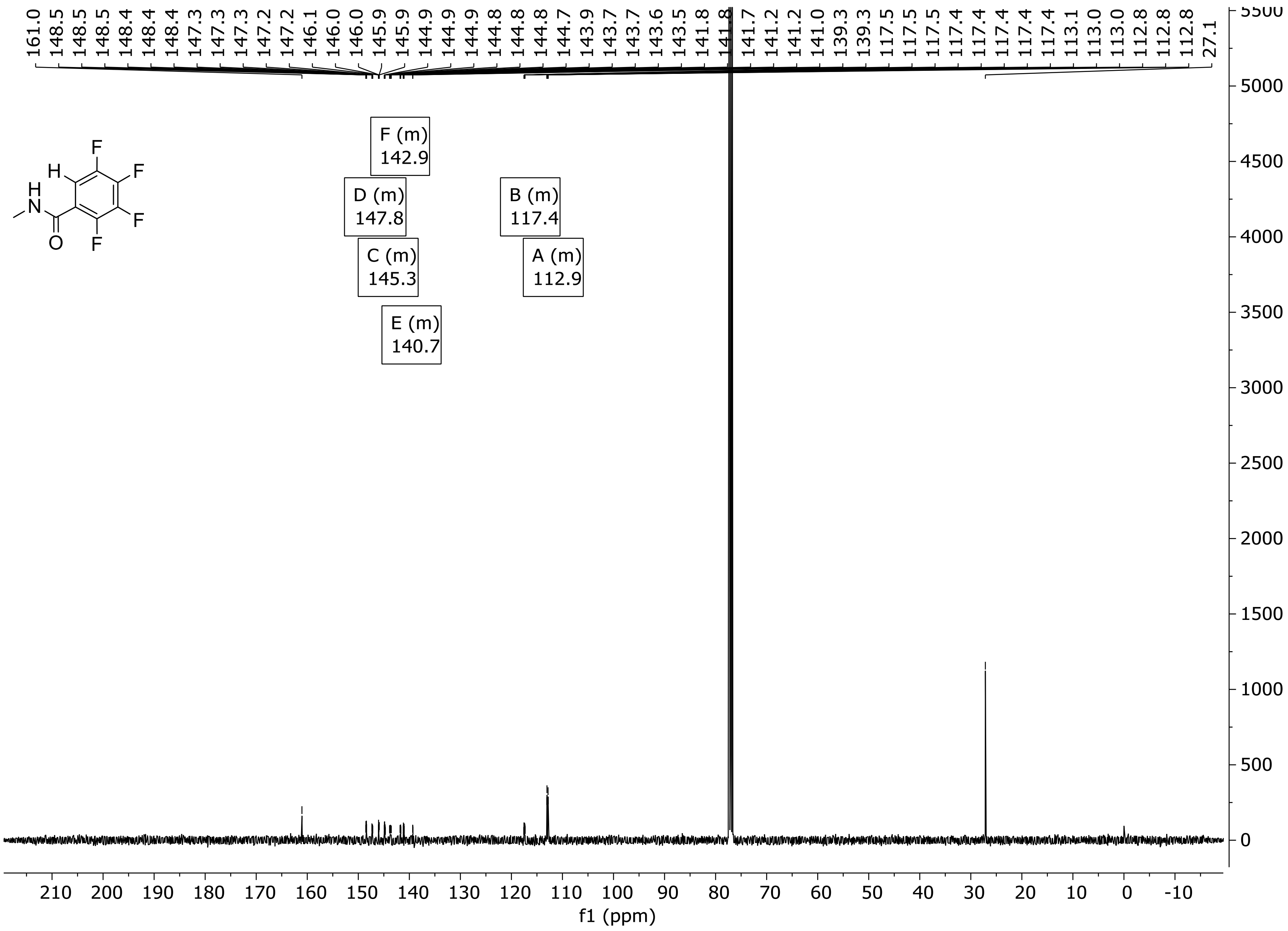

Figure S92. $^{13}C\{^{1}H\}$ NMR spectrum of compound **10** ($CDCl_3$, 101 MHz).

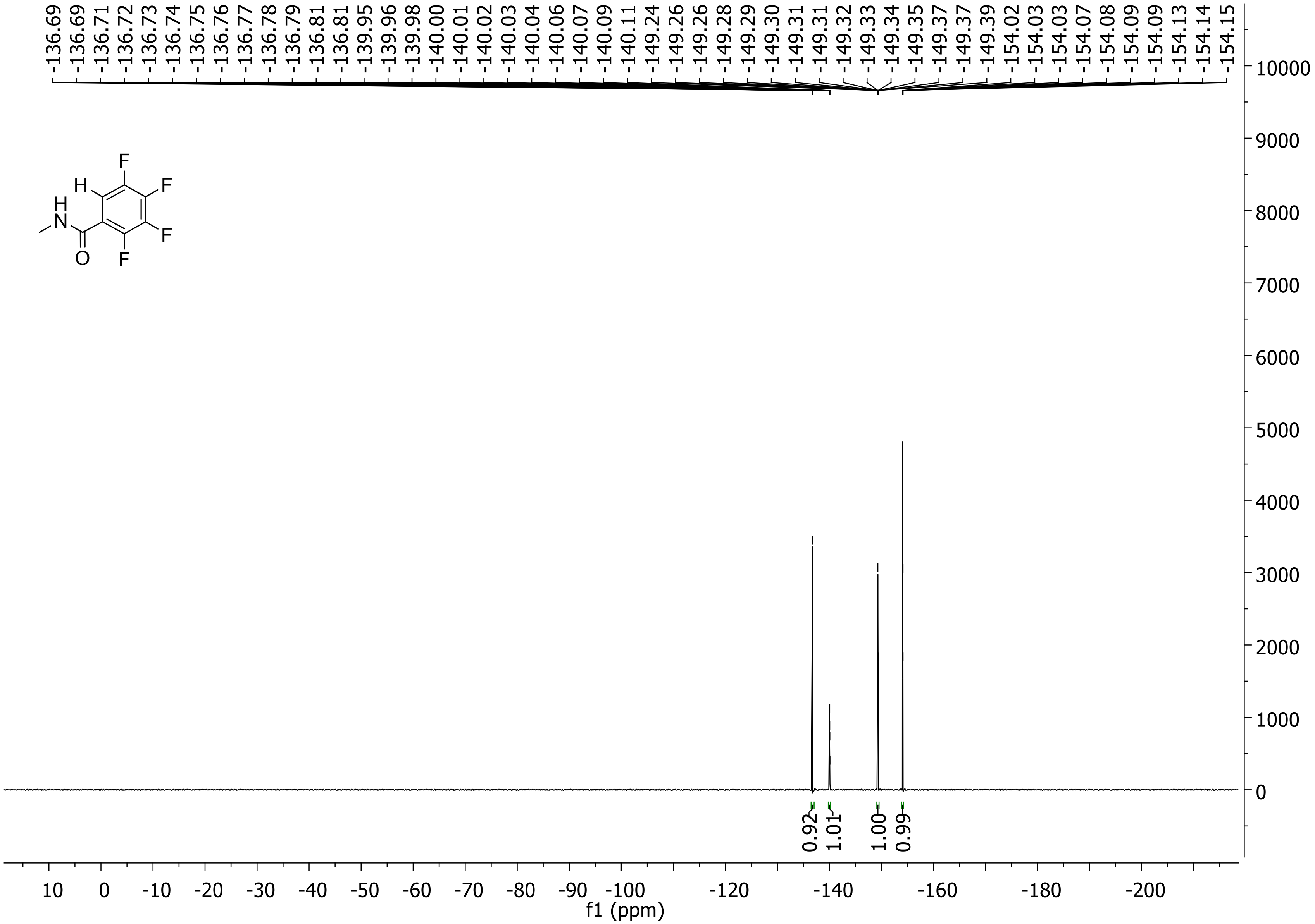

Figure S93. ^{19}F NMR spectrum of compound **10** ($CDCl_3$, 376 MHz).

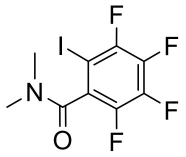

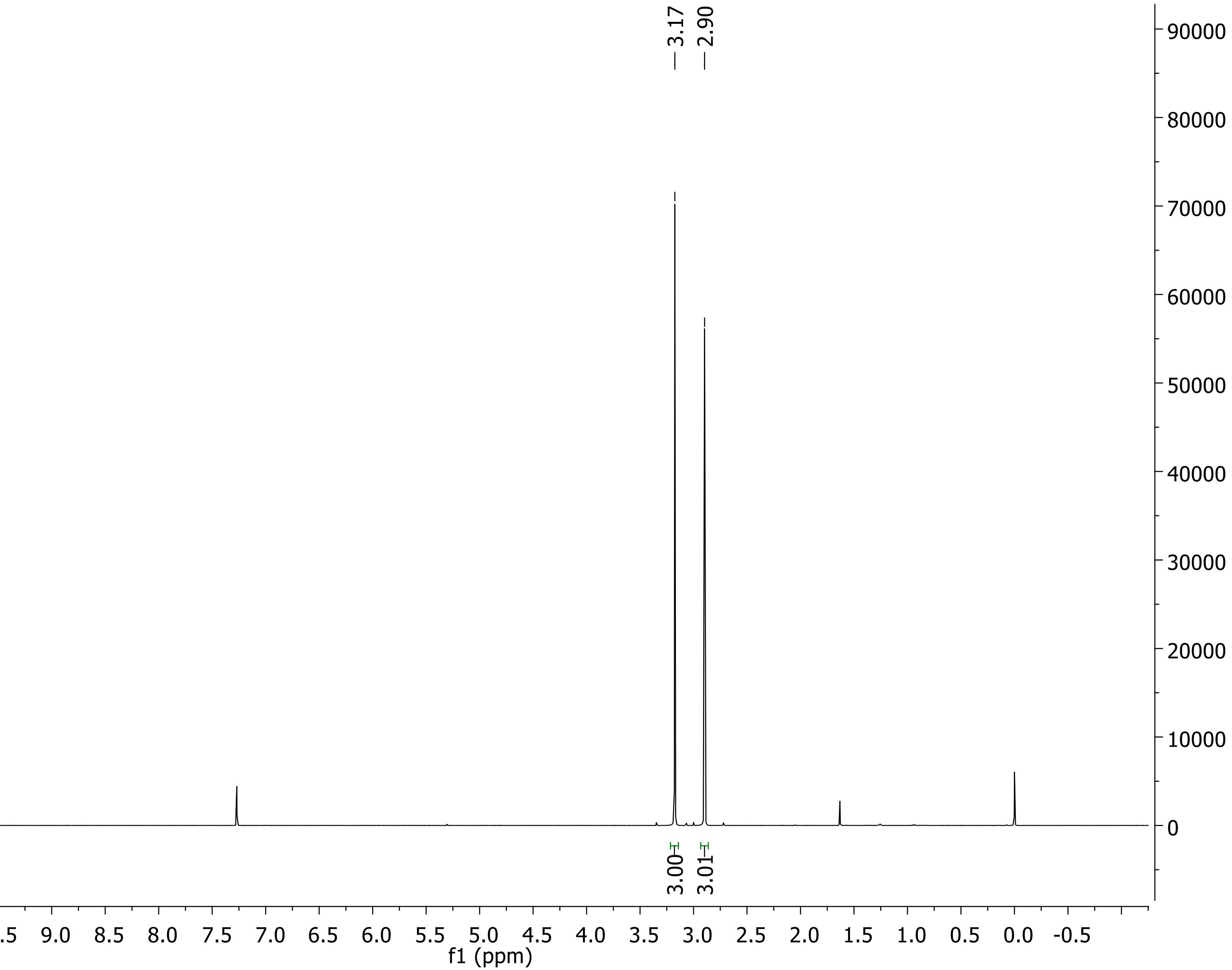

Figure S94. ^{1}H NMR spectrum of compound **11** ($CDCl_3$, 400 MHz).

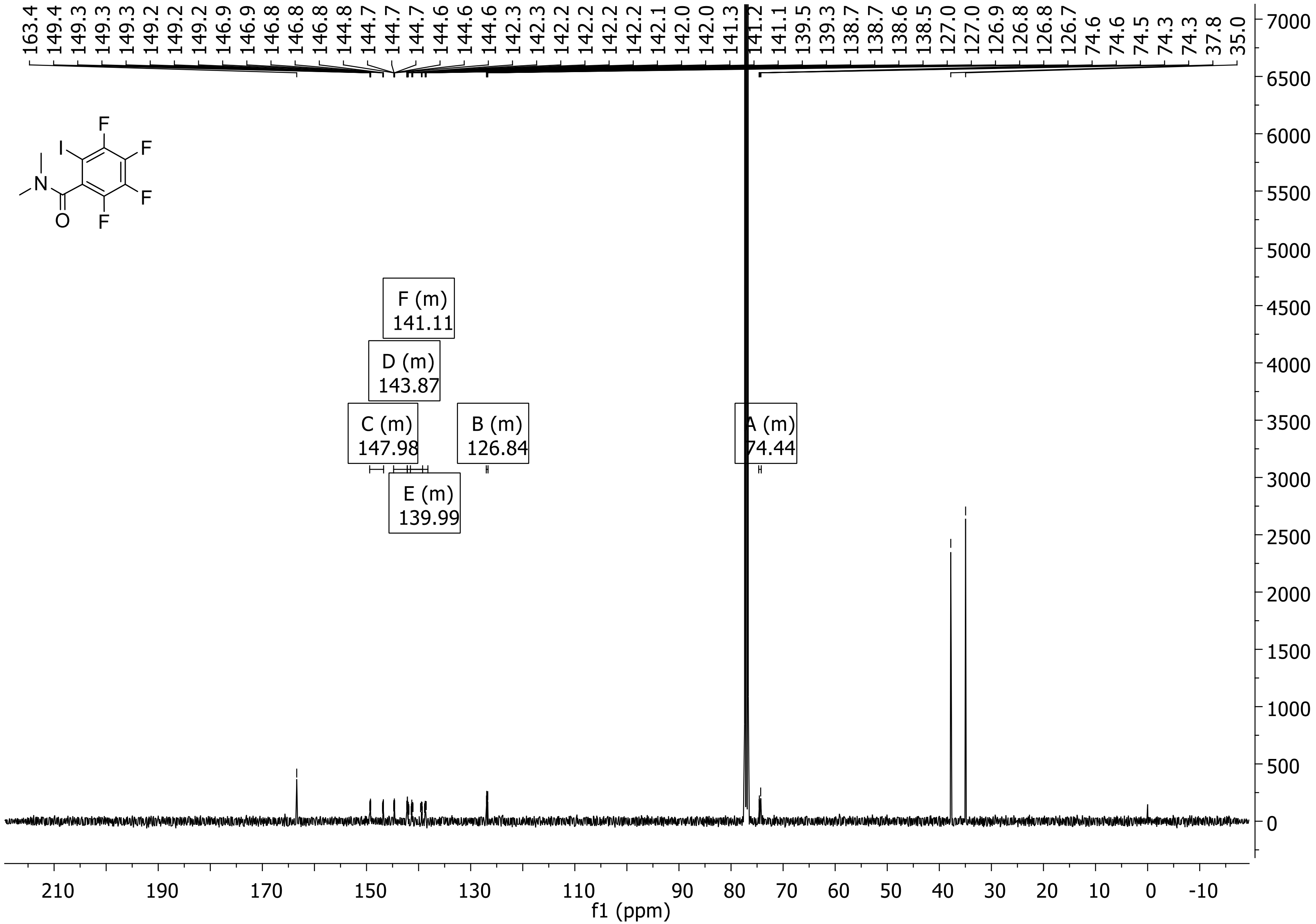

Figure S95. $^{13}C\{^{1}H\}$ NMR spectrum of compound **11** ($CDCl_3$, 101 MHz).

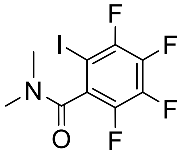

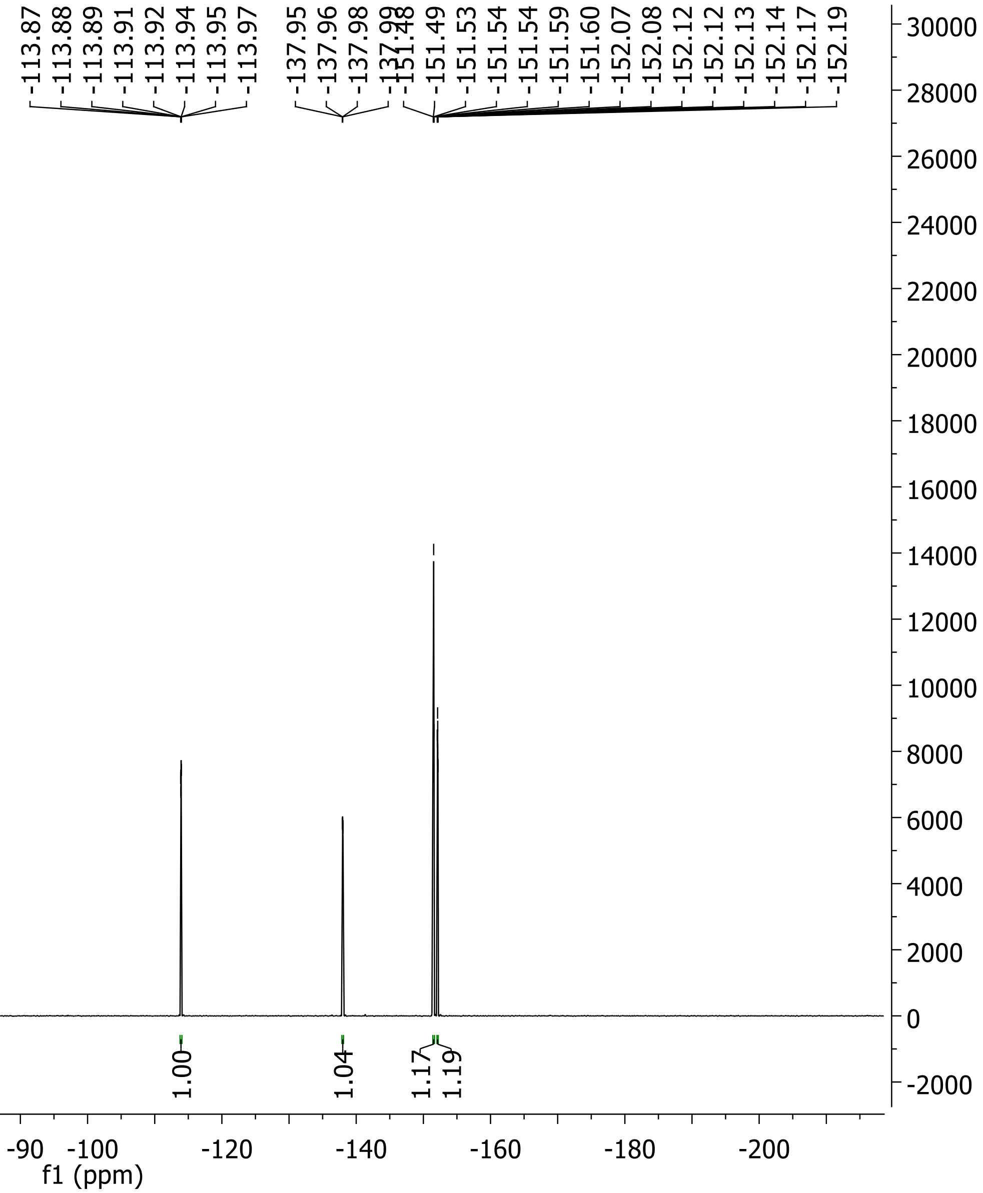

Figure S96. ^{19}F NMR spectrum of catalyst **11** ($CDCl_3$, 376 MHz).

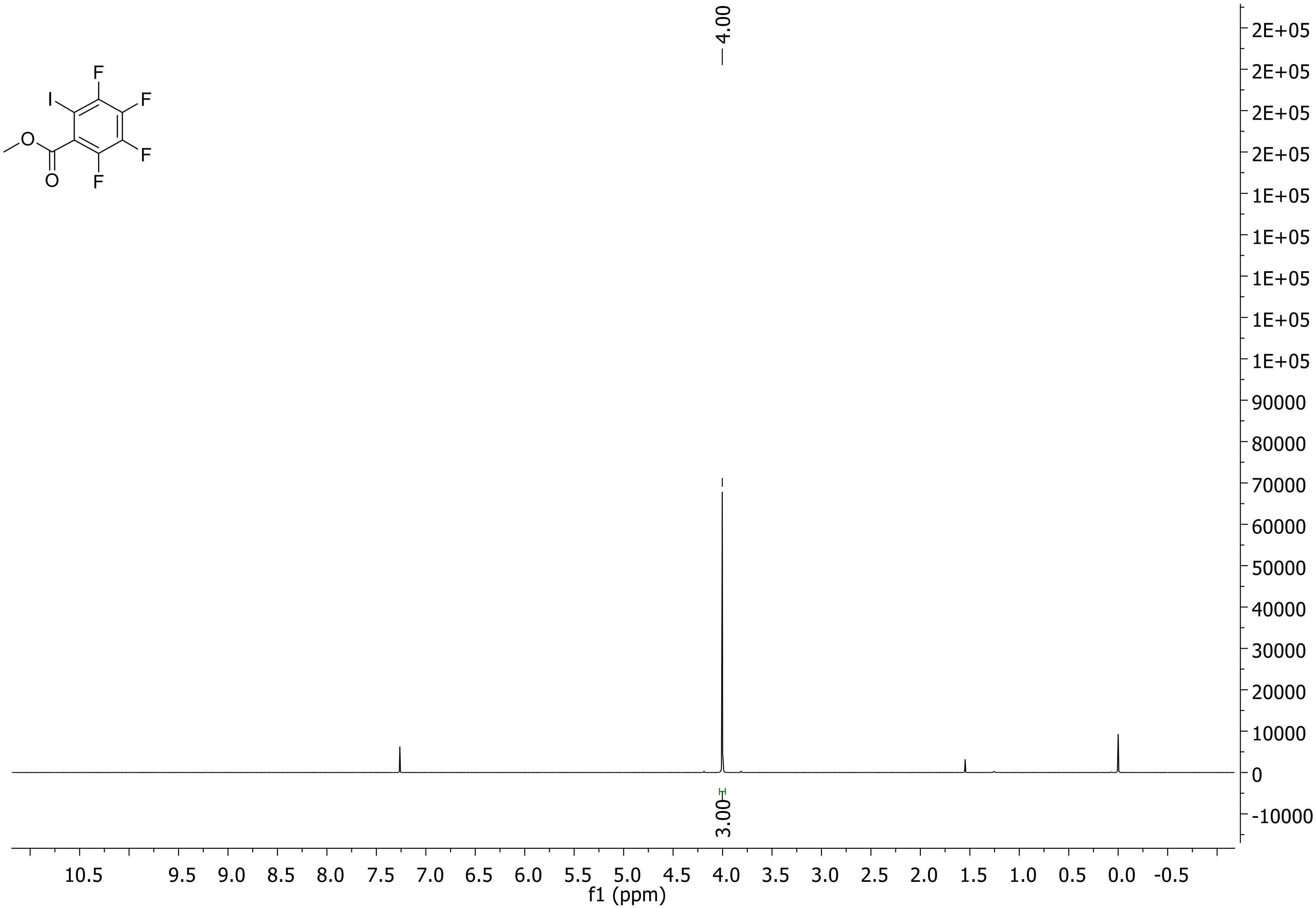

Figure S97. ^{1}H NMR spectrum of compound **12** ($CDCl_3$, 400 MHz).

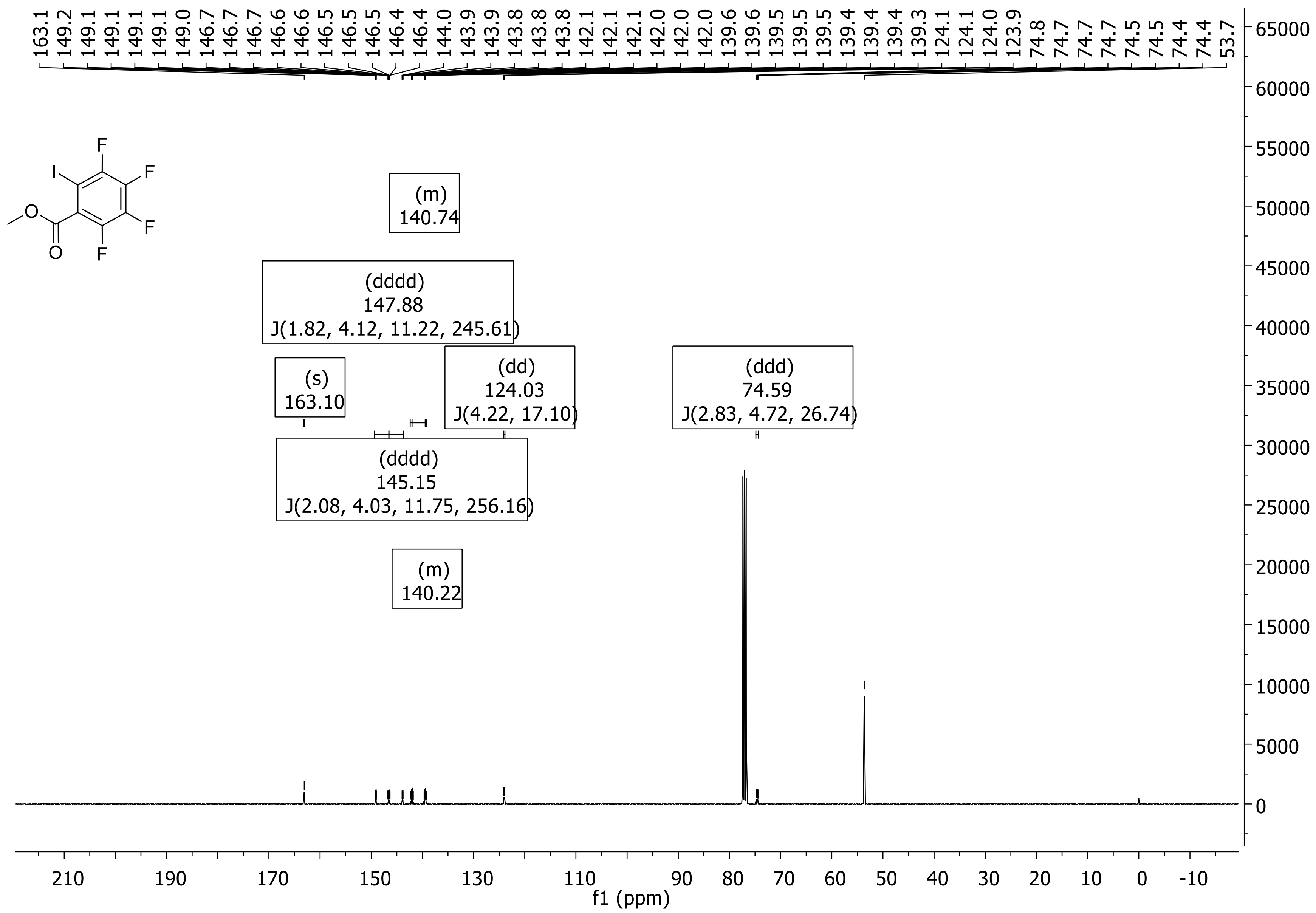

Figure S98. $^{13}C\{^{1}H\}$ NMR spectrum of compound **12** ($CDCl_3$, 101 MHz).

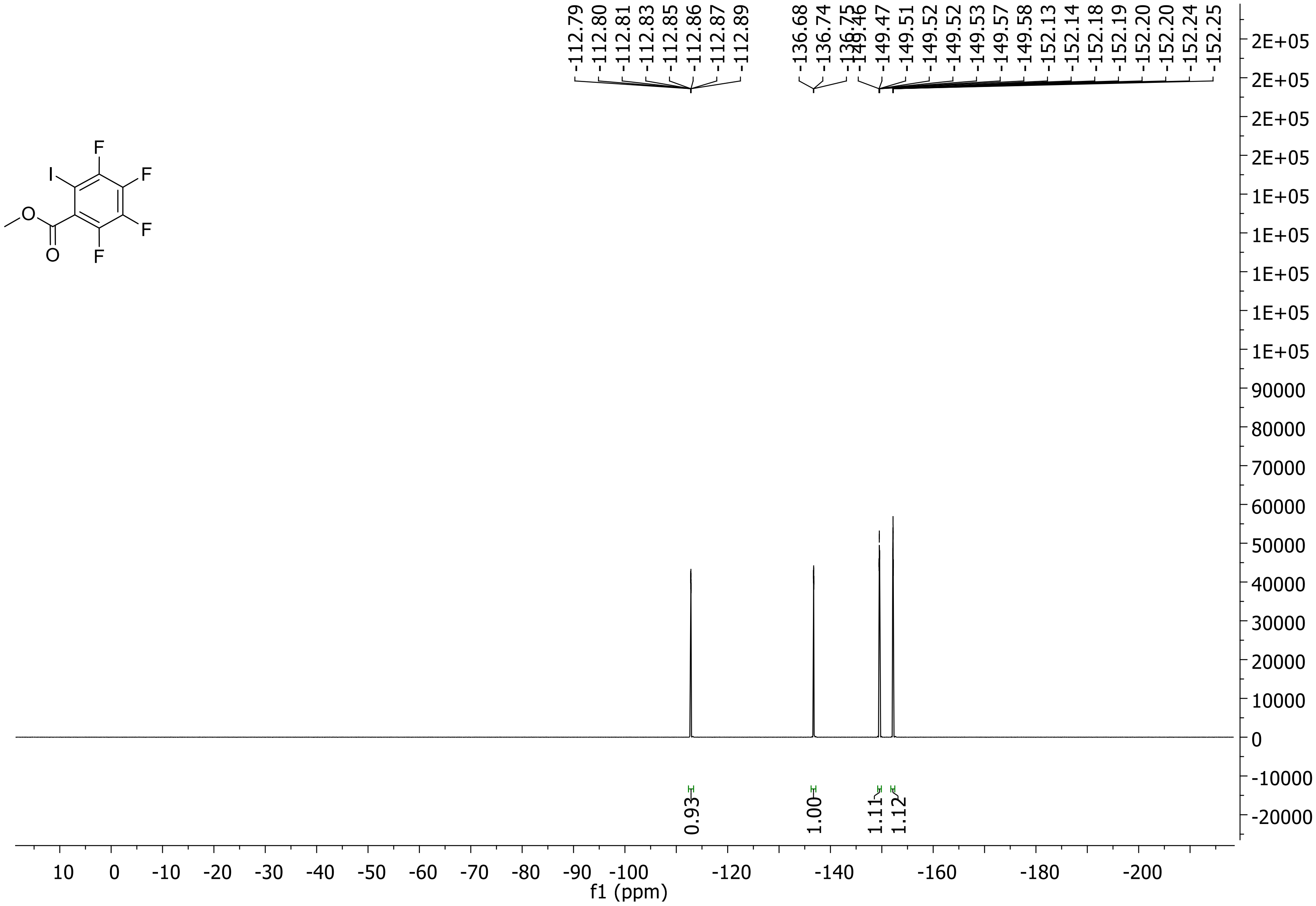

Figure S99. ^{19}F NMR spectrum of compound**12** ($CDCl_3$, 376 MHz).

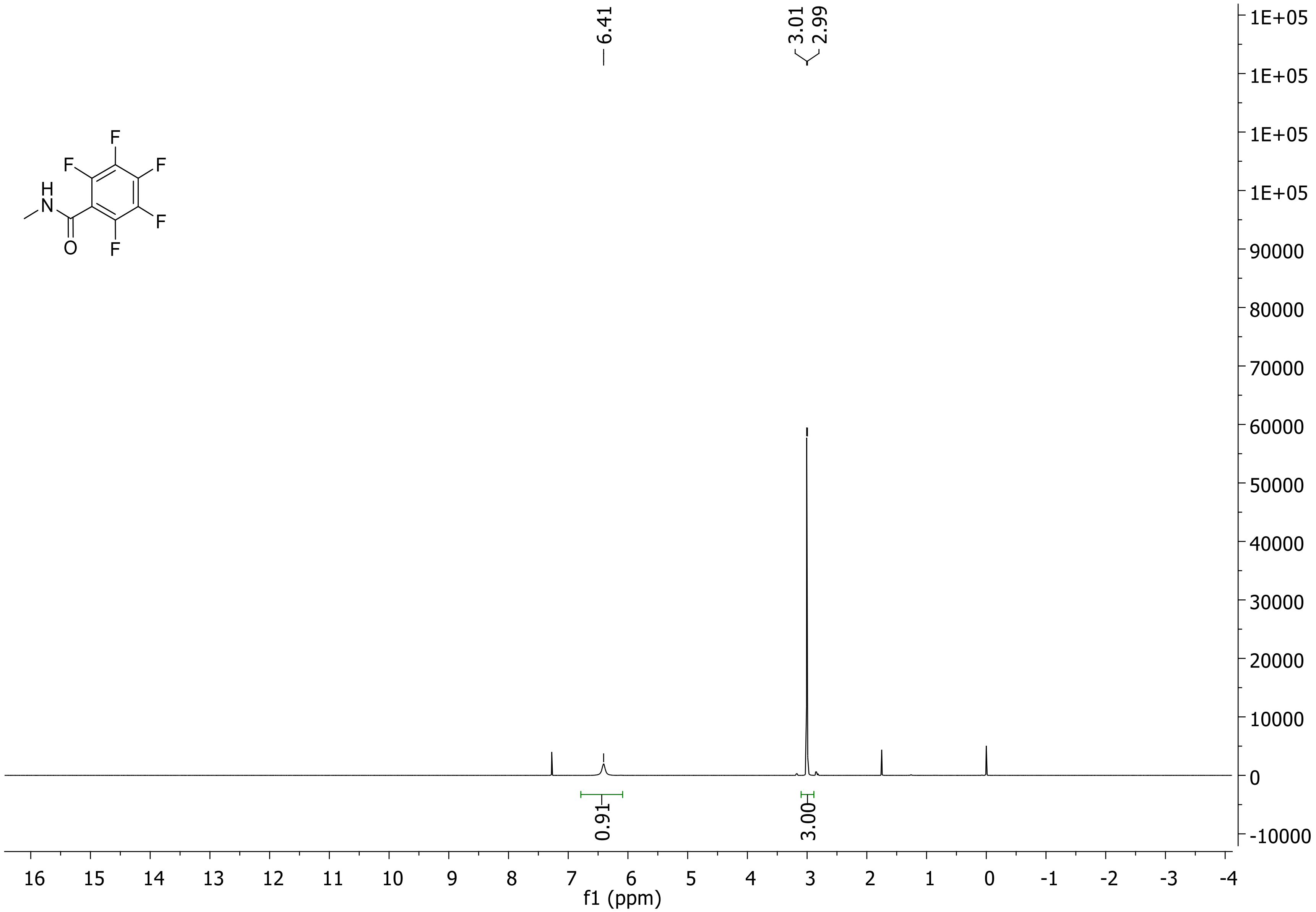

Figure S100. ^{1}H NMR spectrum of compound **13** ($CDCl_3$, 400 MHz).

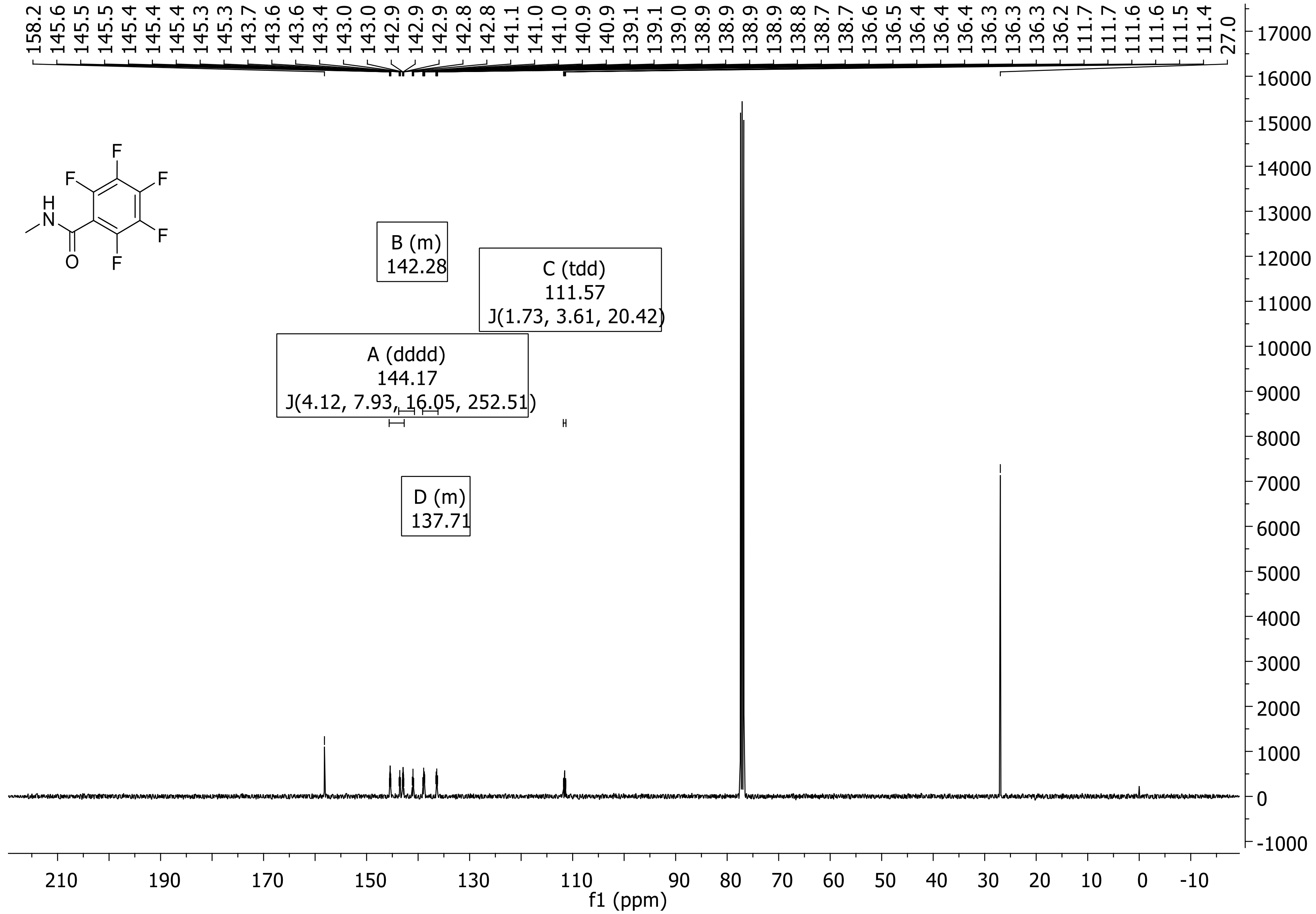

Figure S101. $^{13}C\{^1H\}$ NMR spectrum of compound **13** ($CDCl_3$, 101 MHz).

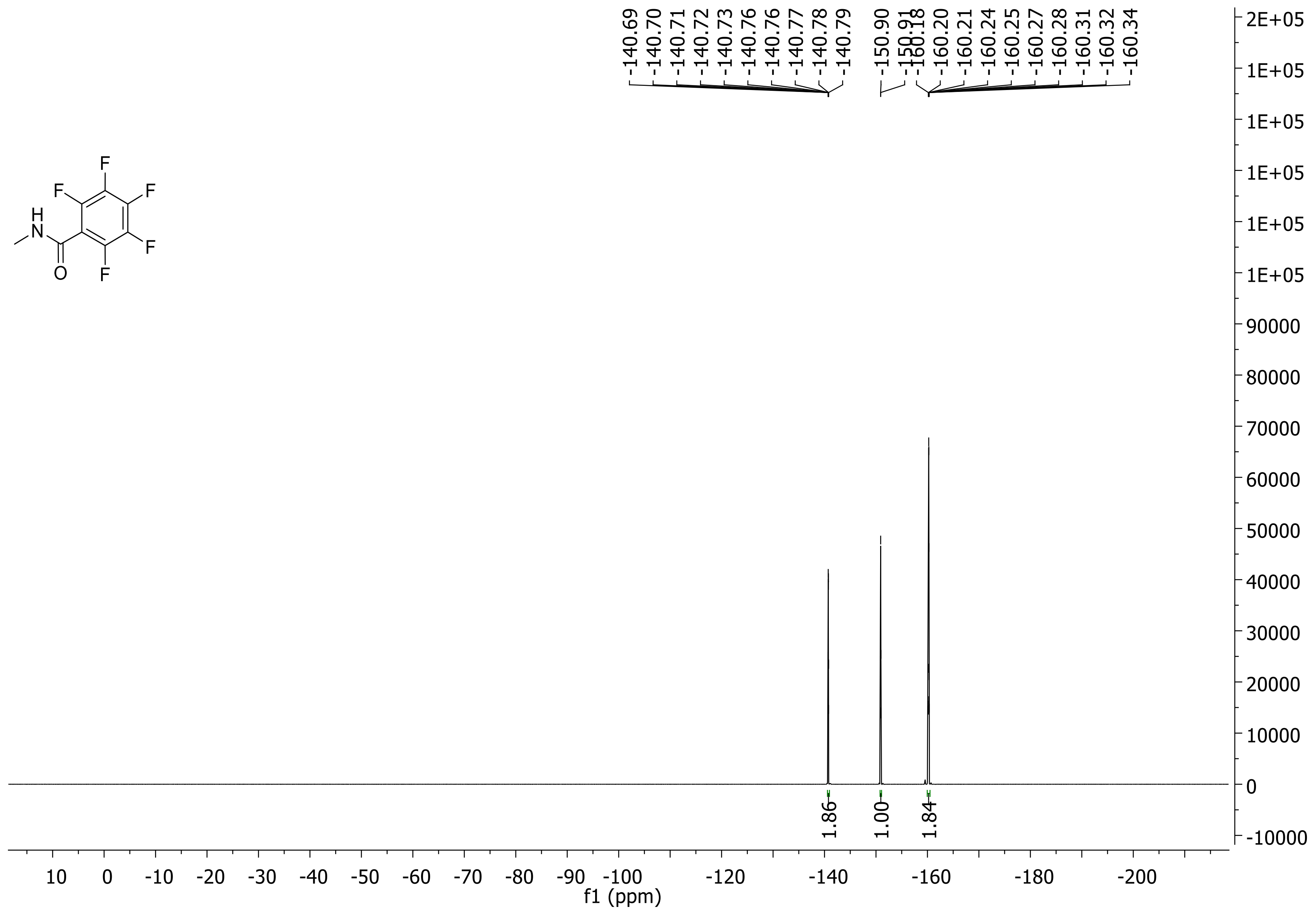

Figure S102. ^{19}F NMR spectrum of catalyst **13** ($CDCl_3$, 376 MHz).

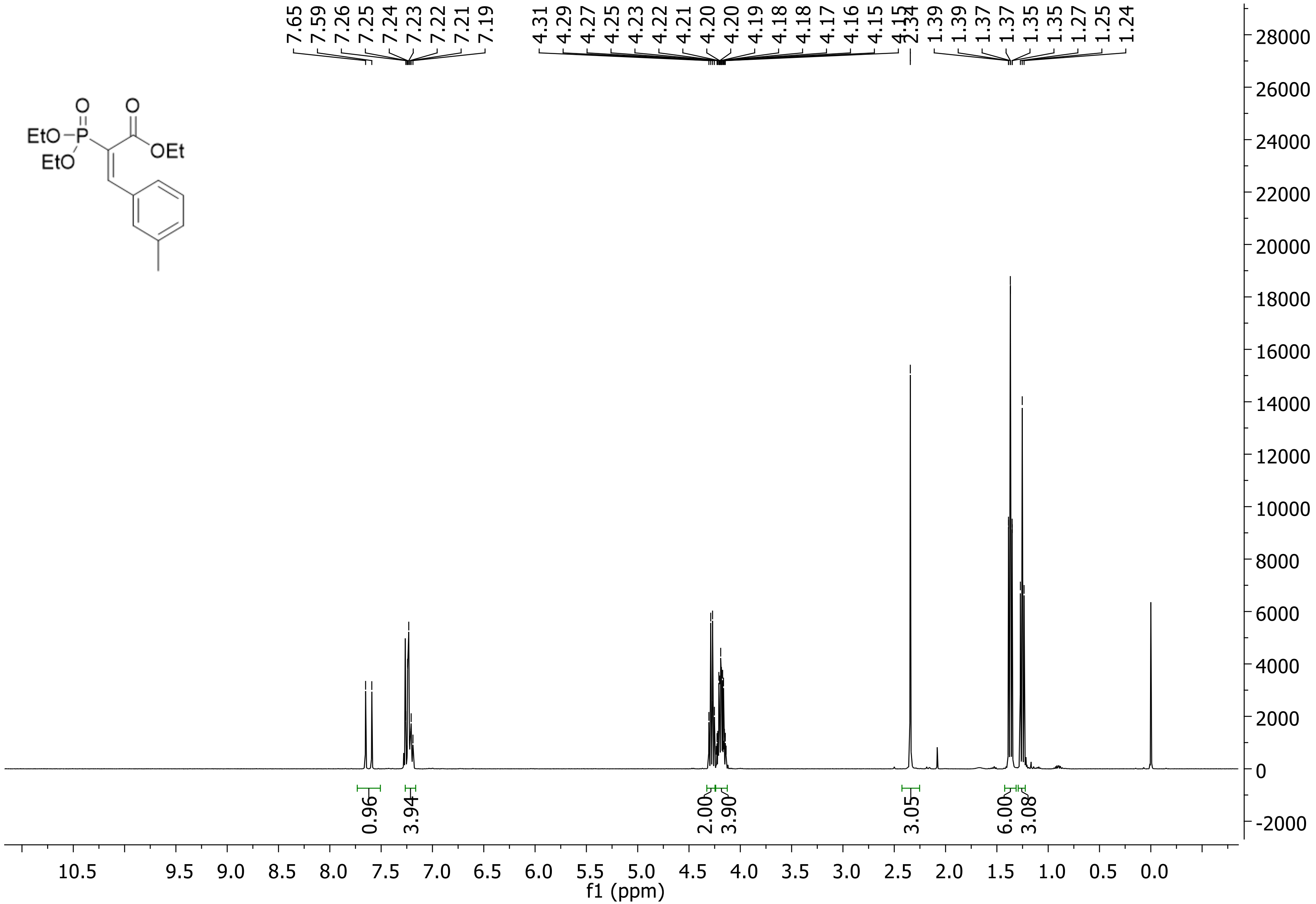

Figure S103. ^{1}H NMR spectrum of **1a** ($CDCl_3$, 400 MHz).

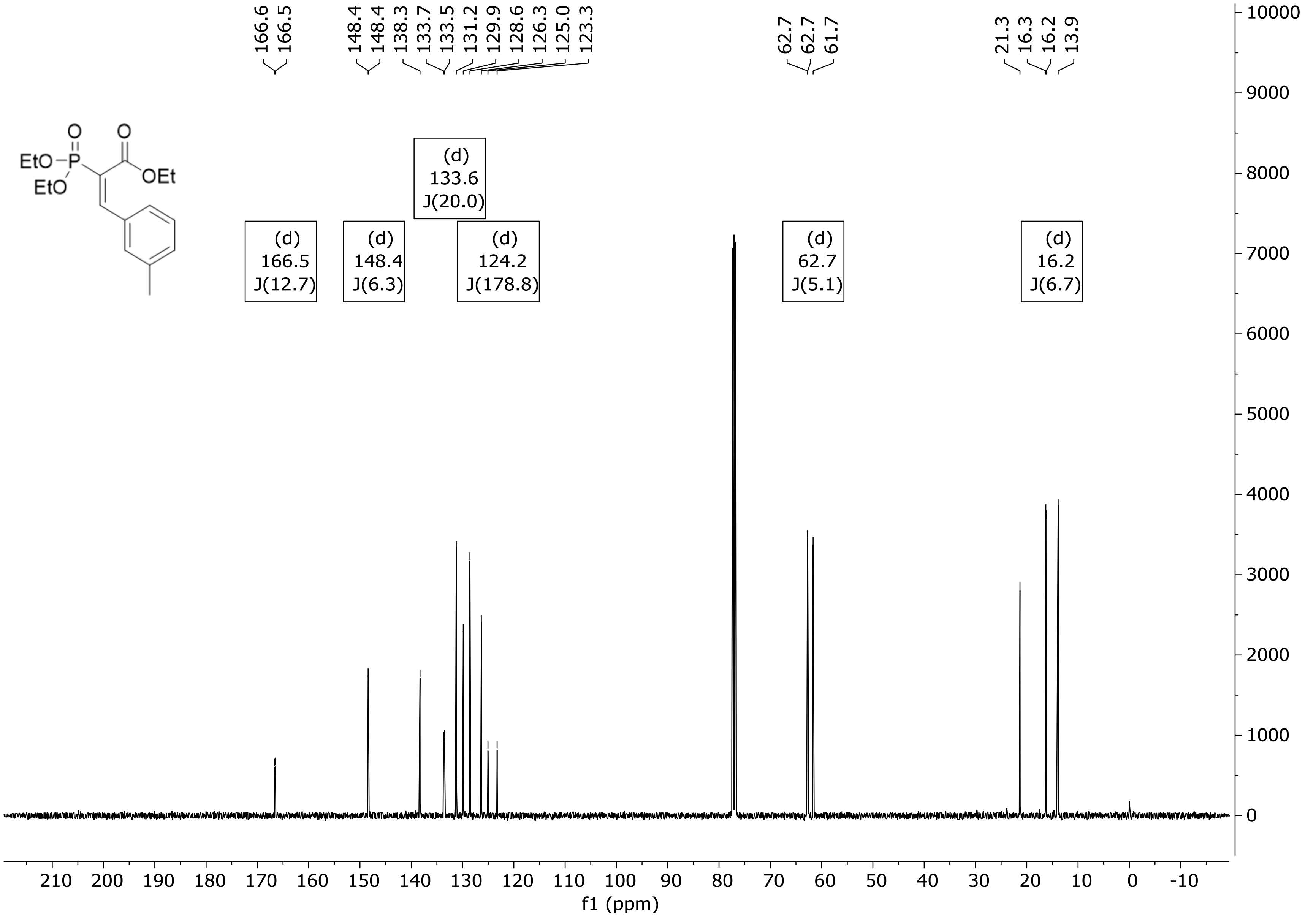

Figure S104. $^{13}C\{^{1}H\}$ NMR spectrum of **1a** ($CDCl_3$, 101 MHz).

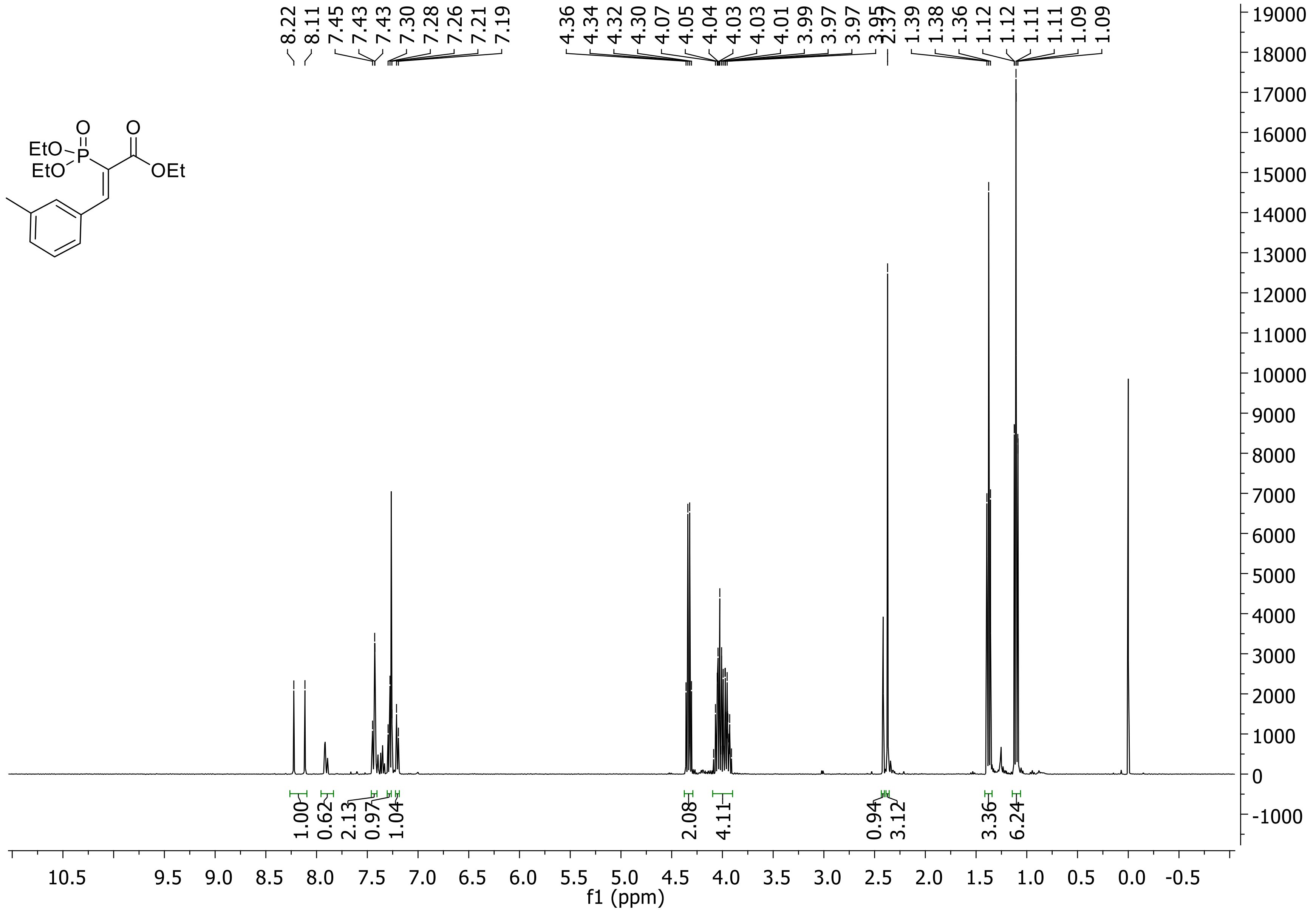

Figure S105. ^{1}H NMR spectrum of ***Z*-1a** ($CDCl_3$, 400 MHz).

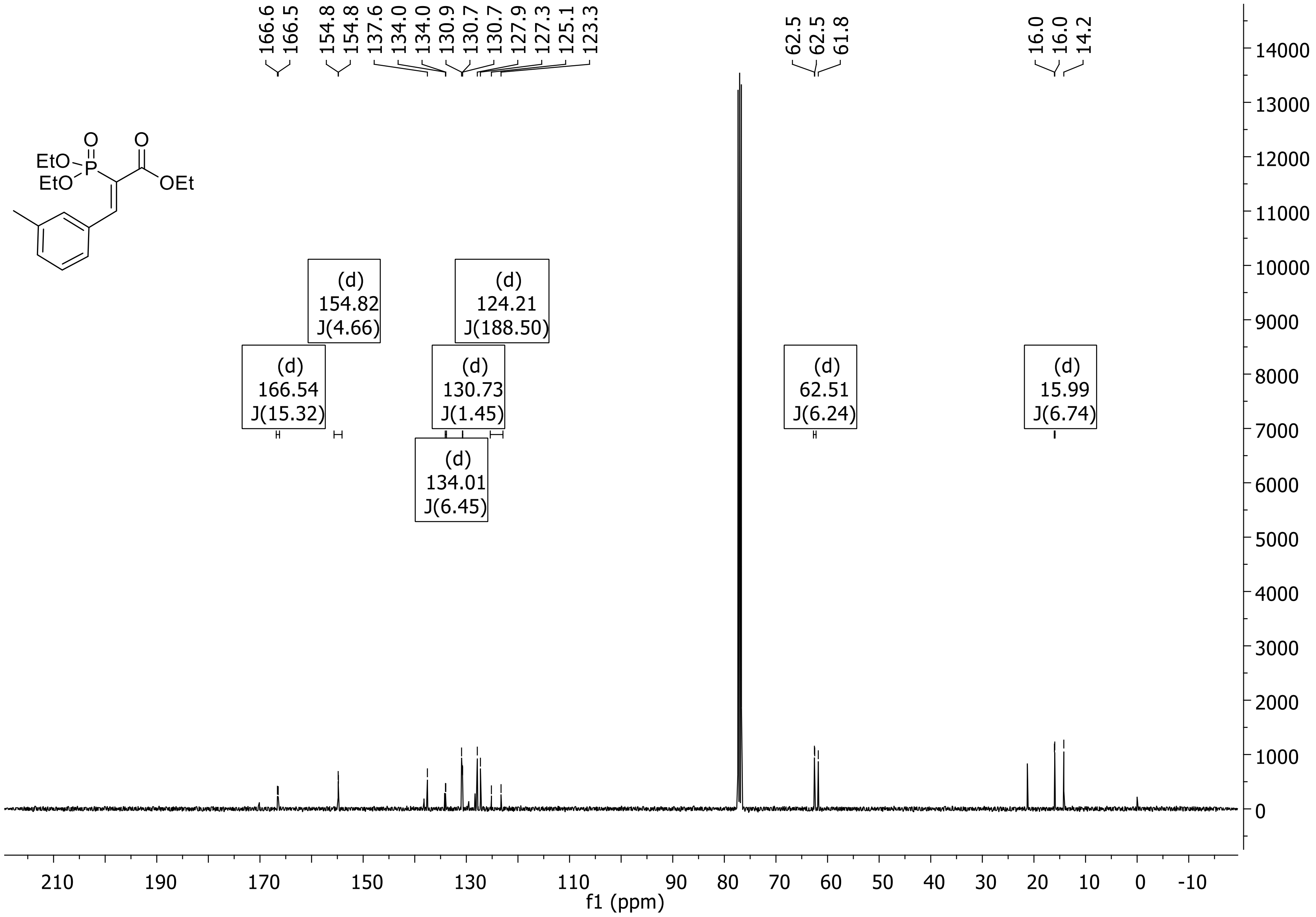

Figure S106. $^{13}C\{^1H\}$ NMR spectrum of ***Z*-1a** ($CDCl_3$, 101 MHz).

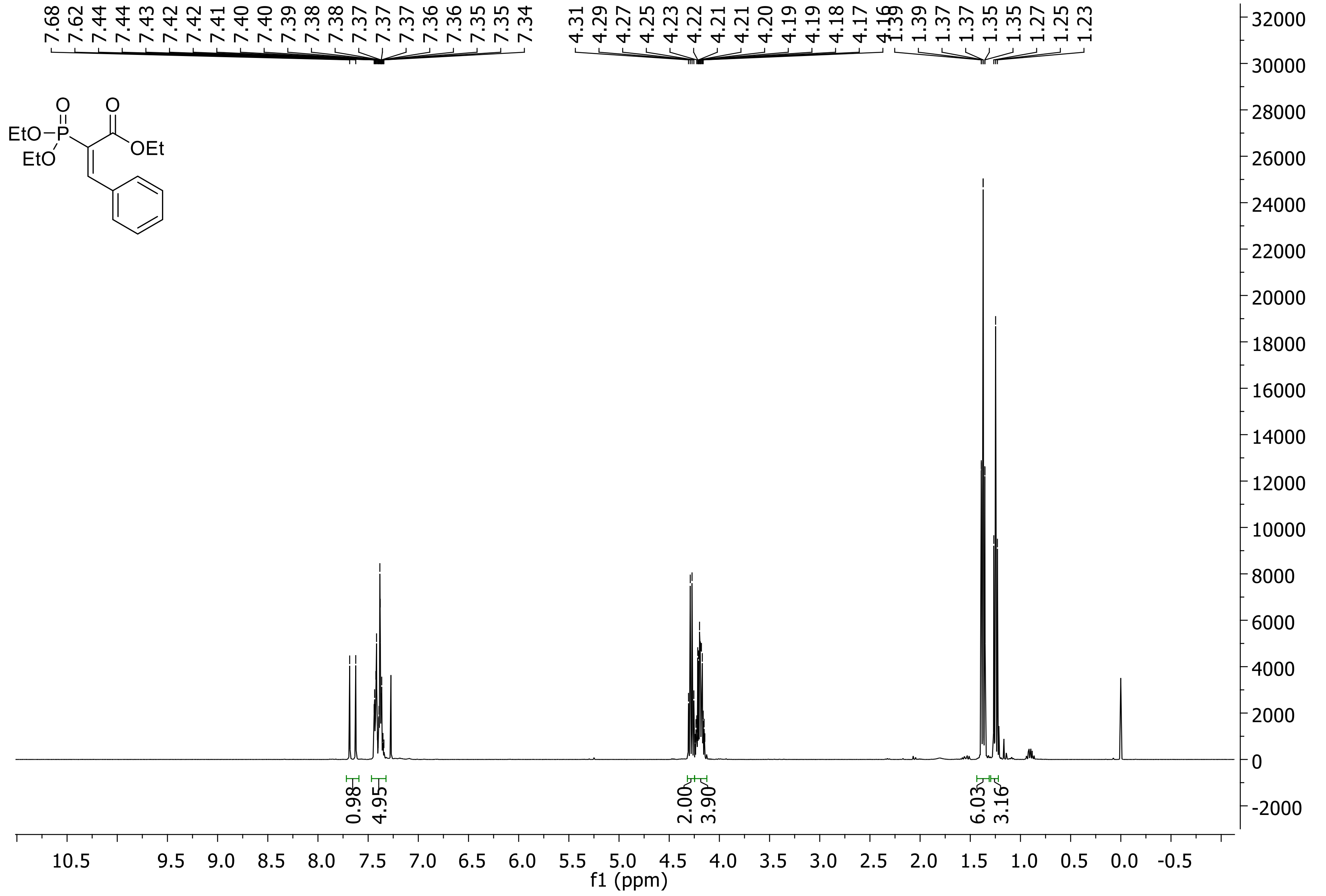

Figure S107. ^{1}H NMR spectrum of **1b** ($CDCl_3$, 400 MHz).

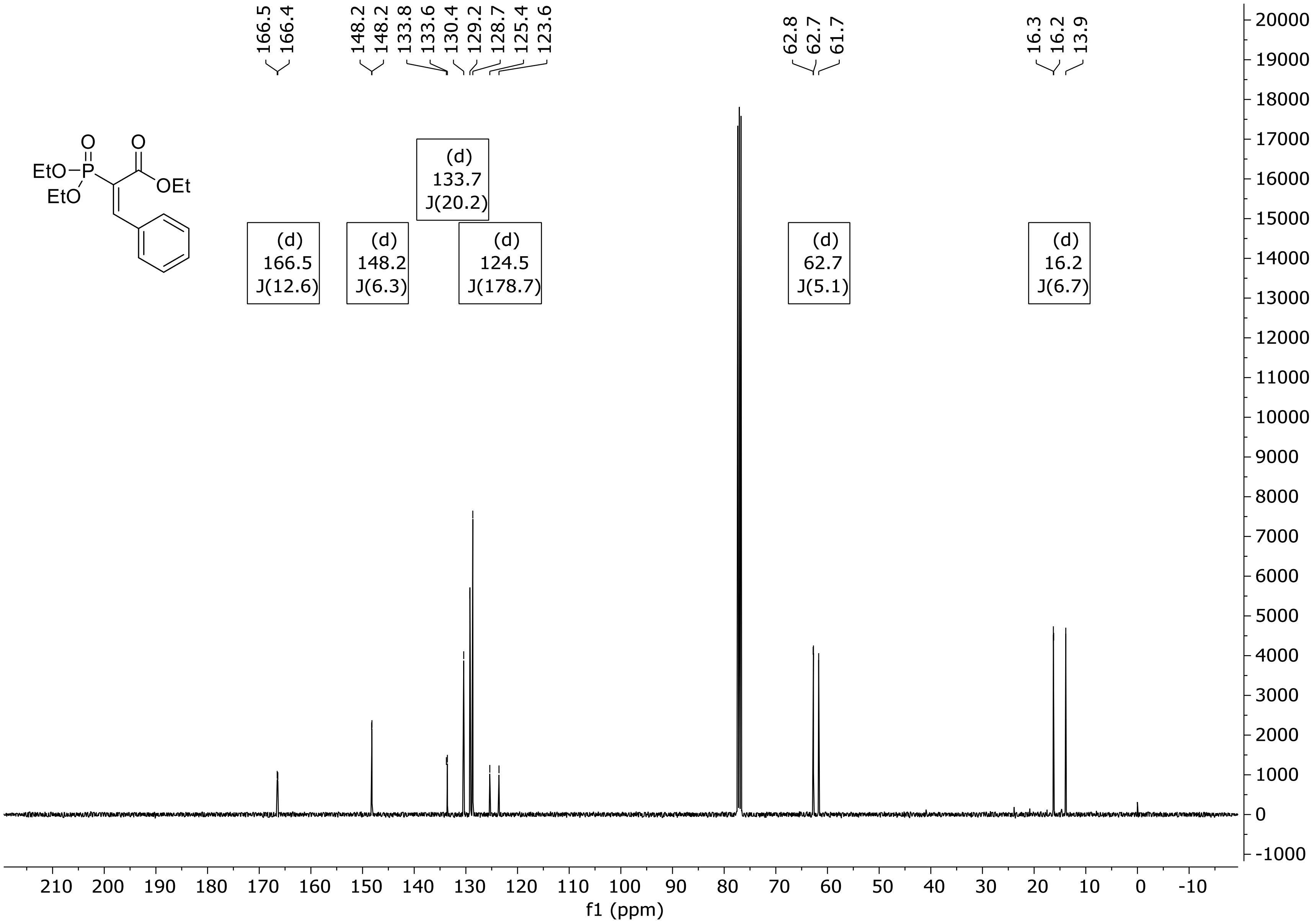

Figure S108. $^{13}C\{^1H\}$ NMR spectrum of **1b** ($CDCl_3$, 101 MHz).

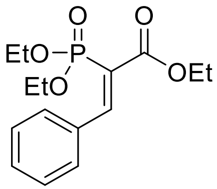

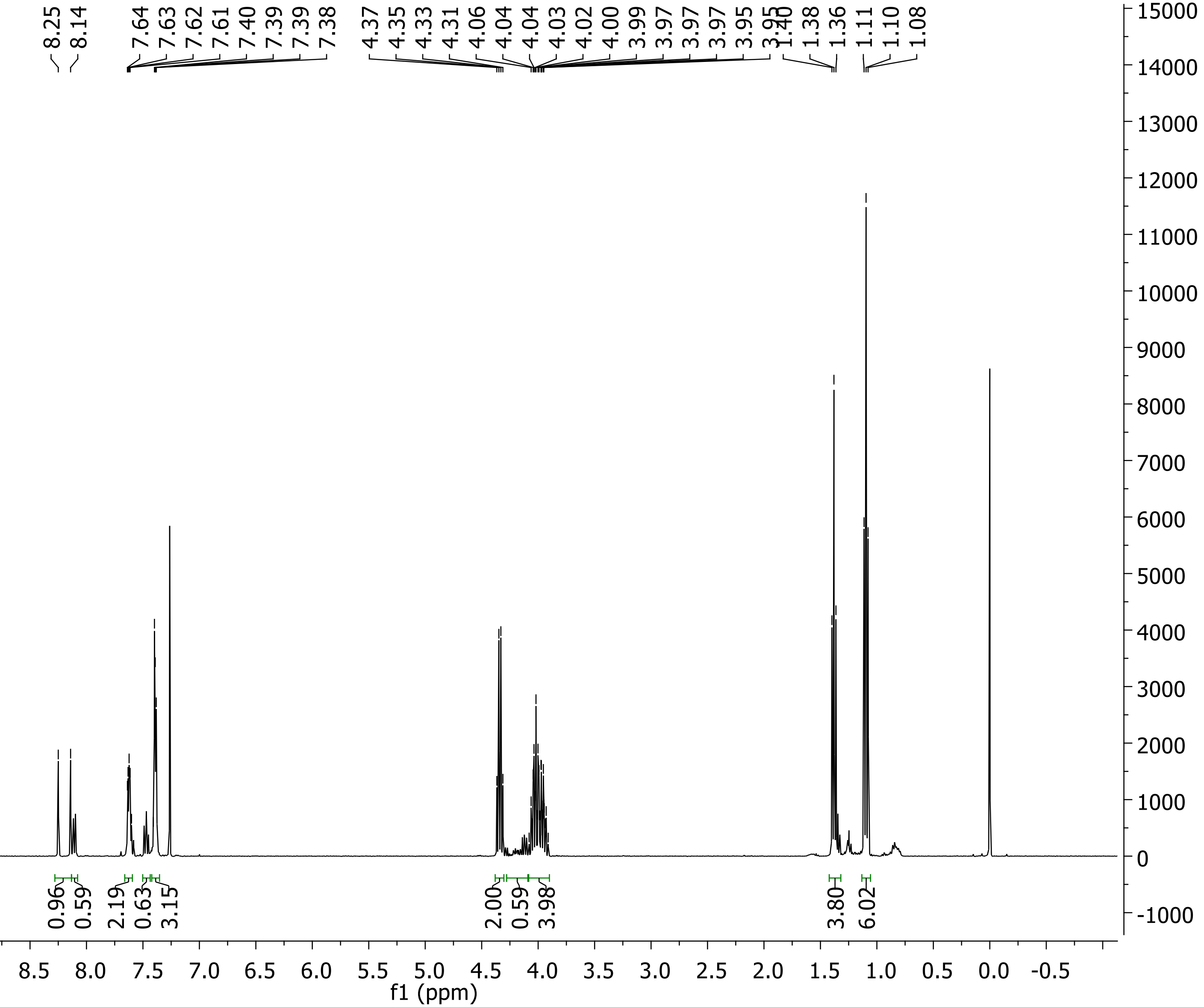

Figure S109. ^{1}H NMR spectrum of ***Z*-1b** ($CDCl_3$, 400 MHz).

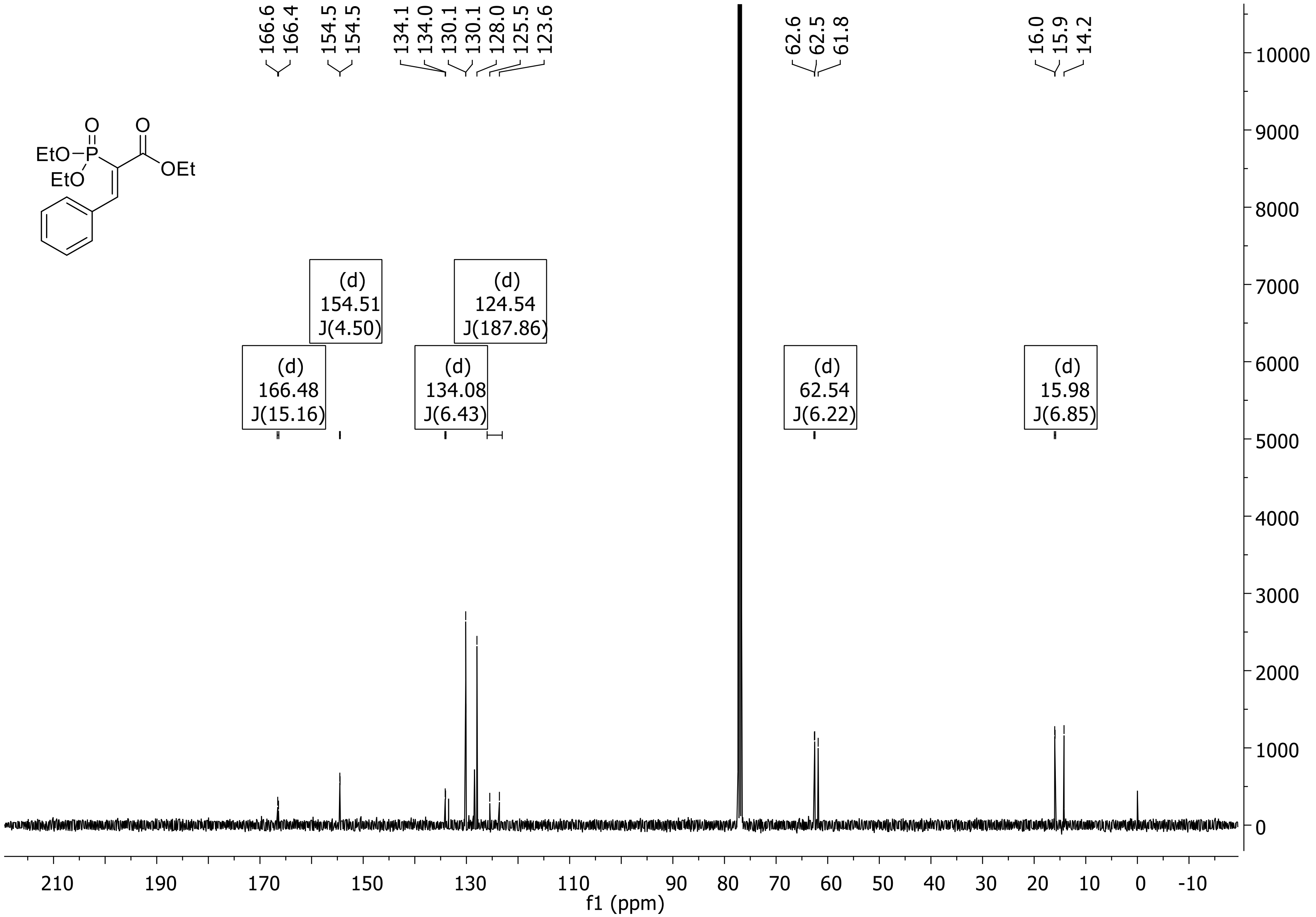

Figure S110. $^{13}C\{^{1}H\}$ NMR spectrum of ***Z*-1b** ($CDCl_3$, 101 MHz).

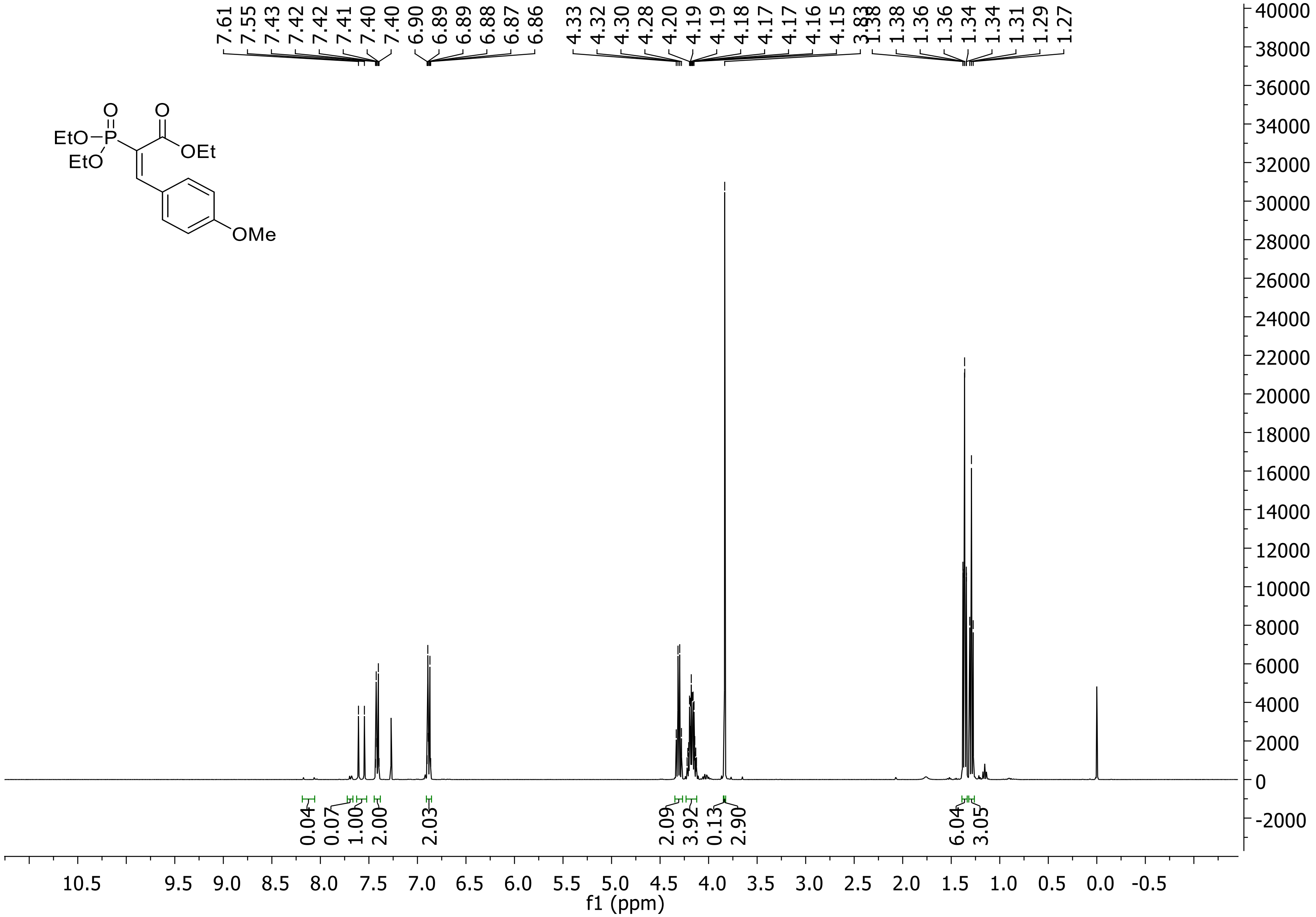

Figure S111. ^{1}H NMR spectrum of **1c** ($CDCl_3$, 400 MHz).

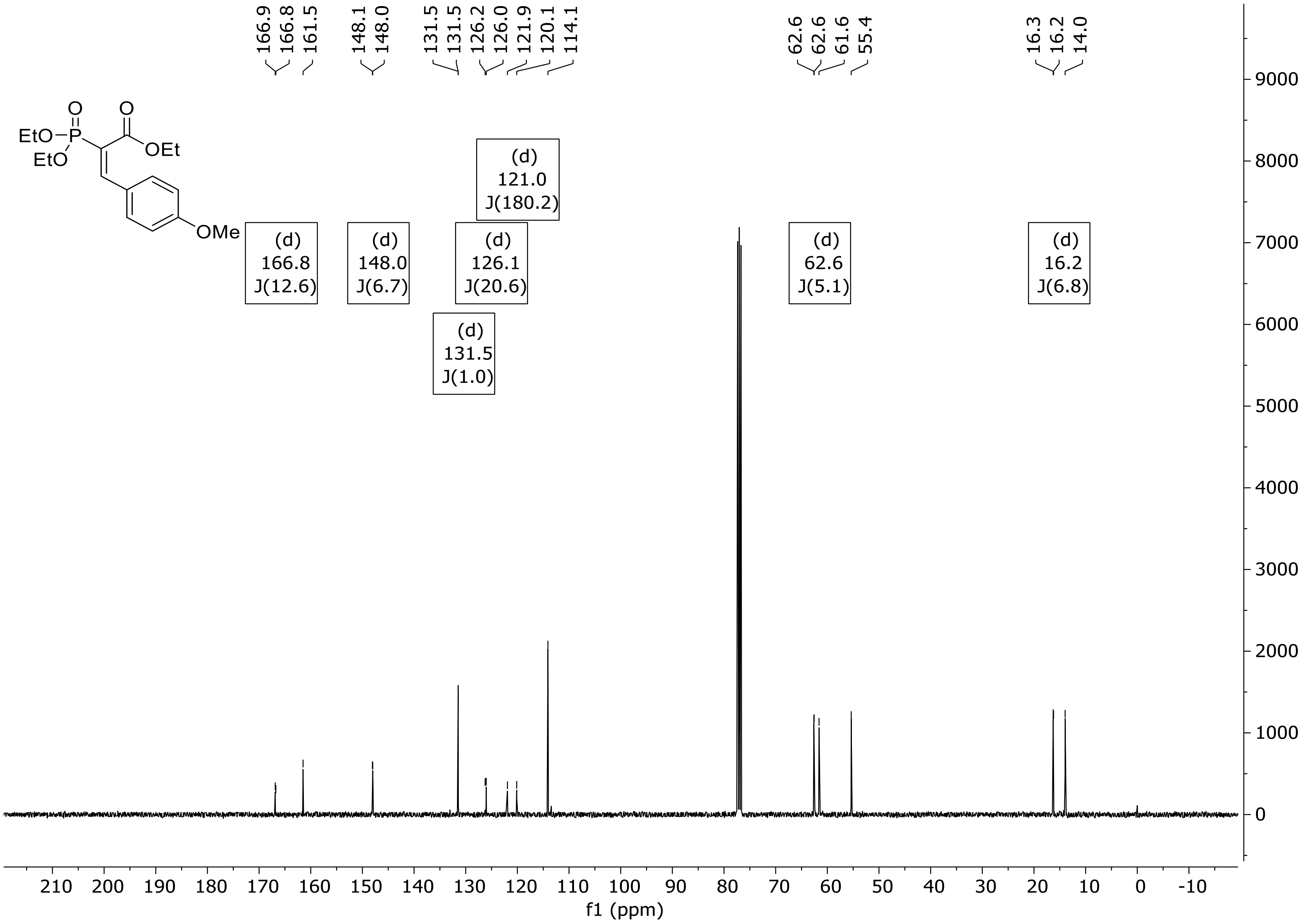

Figure S112. $^{13}C\{^1H\}$ NMR spectrum of **1c** ($CDCl_3$, 101 MHz).

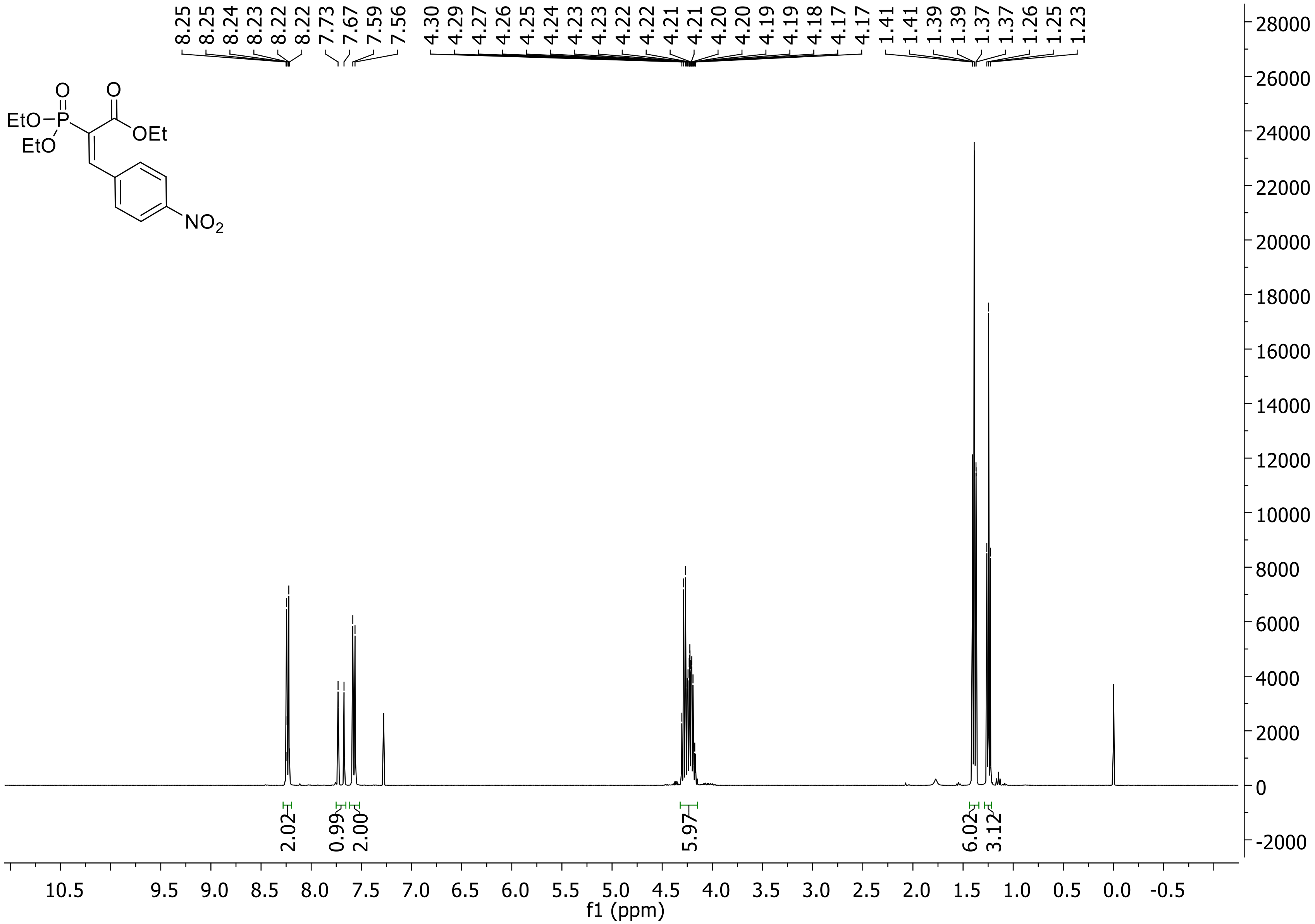

Figure S113. ^{1}H NMR spectrum of **1d** ($CDCl_3$, 400 MHz).

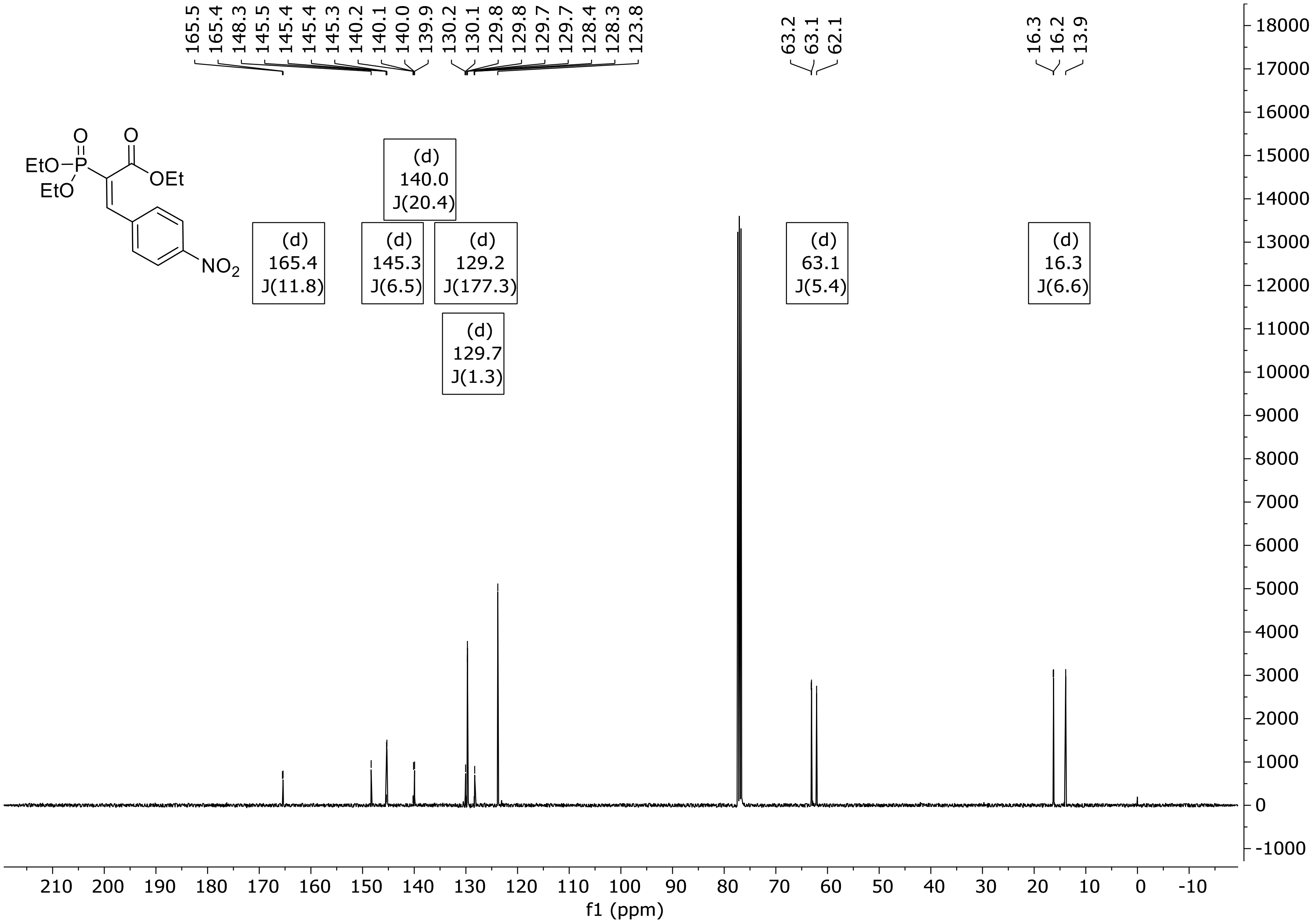

Figure S114. $^{13}C\{^{1}H\}$ NMR spectrum of **1d** ($CDCl_3$, 101 MHz).

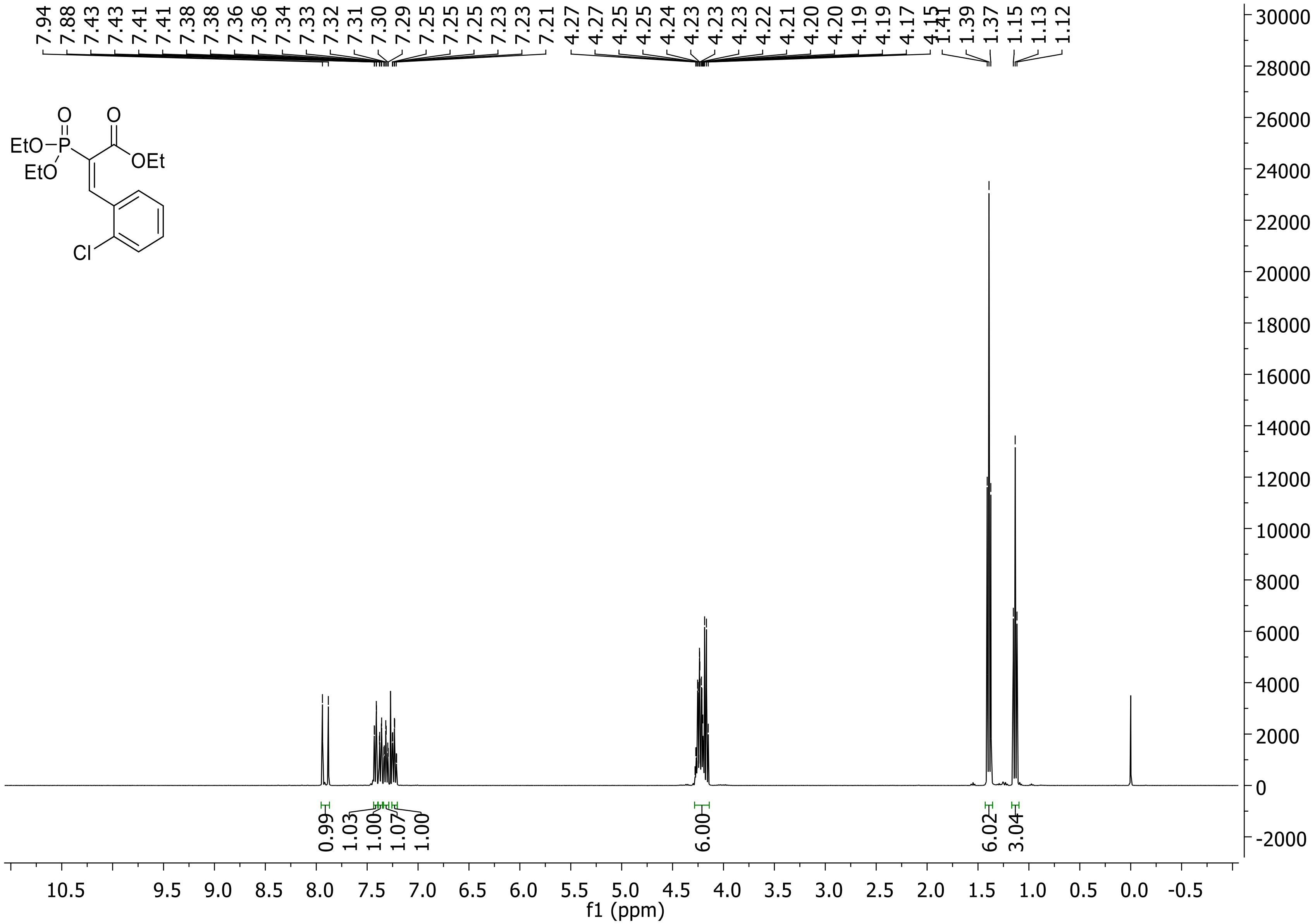

Figure S115. ^{1}H NMR spectrum of **1e** ($CDCl_3$, 400 MHz).

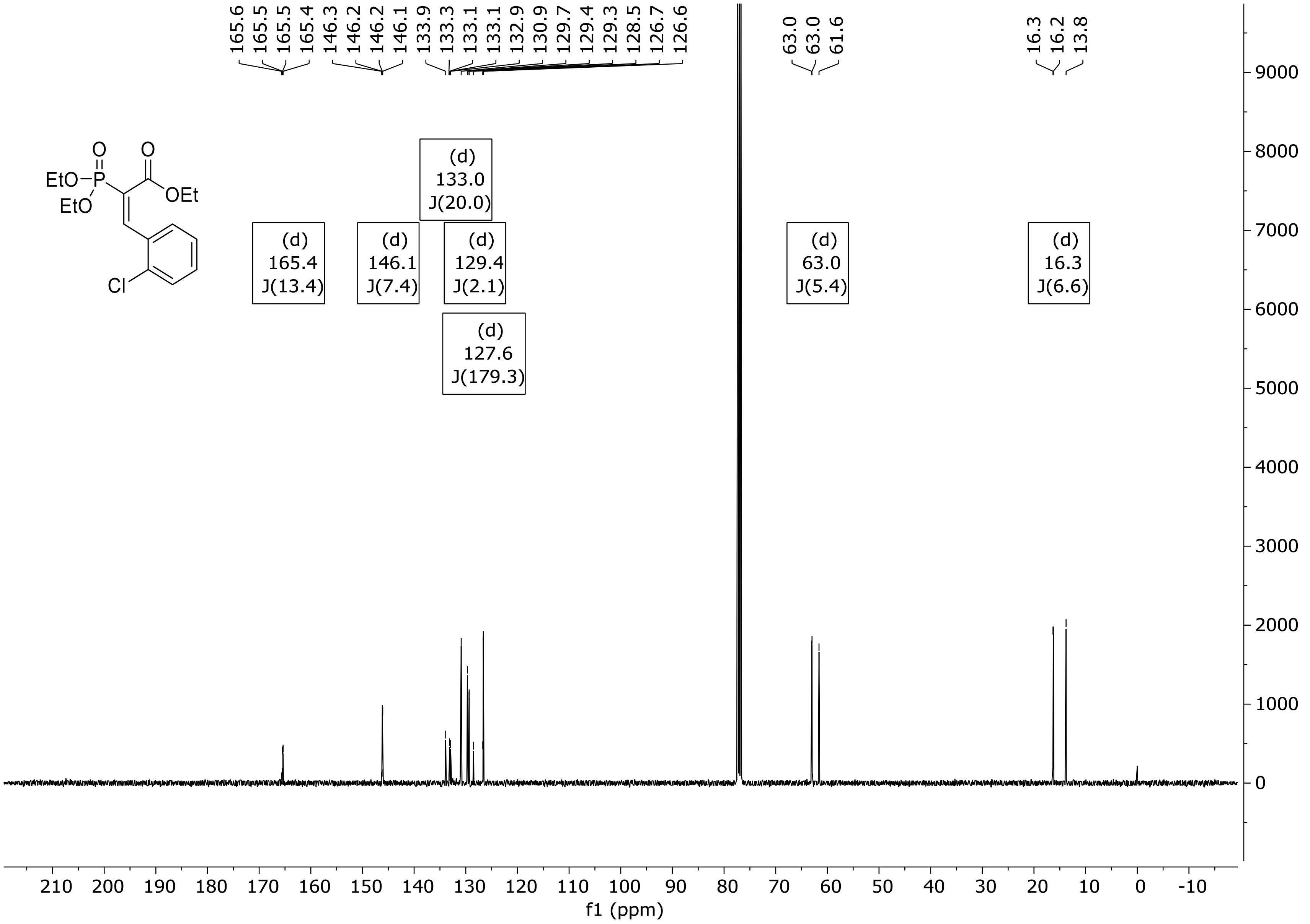

Figure S116. $^{13}C\{^{1}H\}$ NMR spectrum of **1e** ($CDCl_3$, 101 MHz).

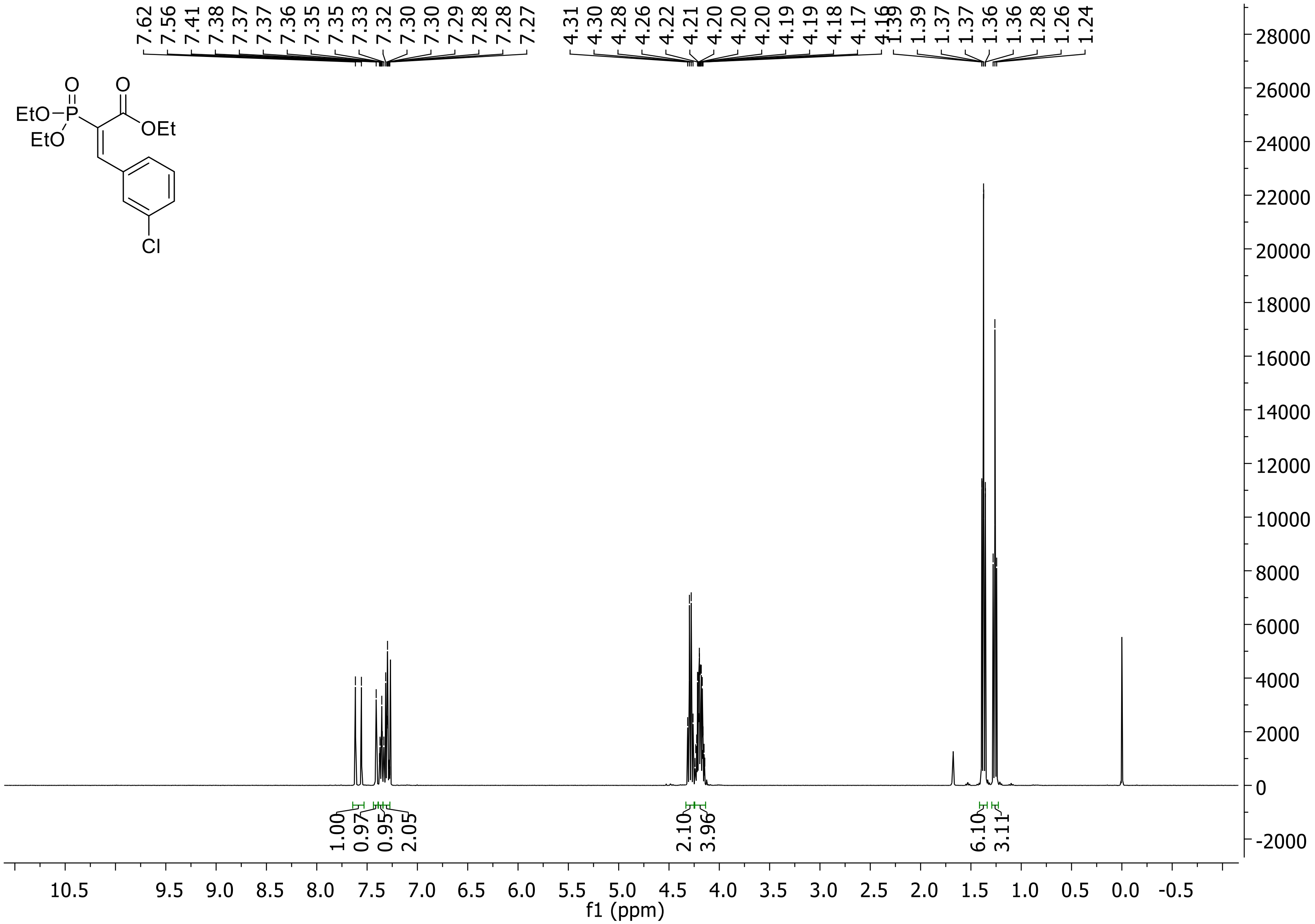

Figure S117. ^{1}H NMR spectrum of **1f** ($CDCl_3$, 400 MHz).

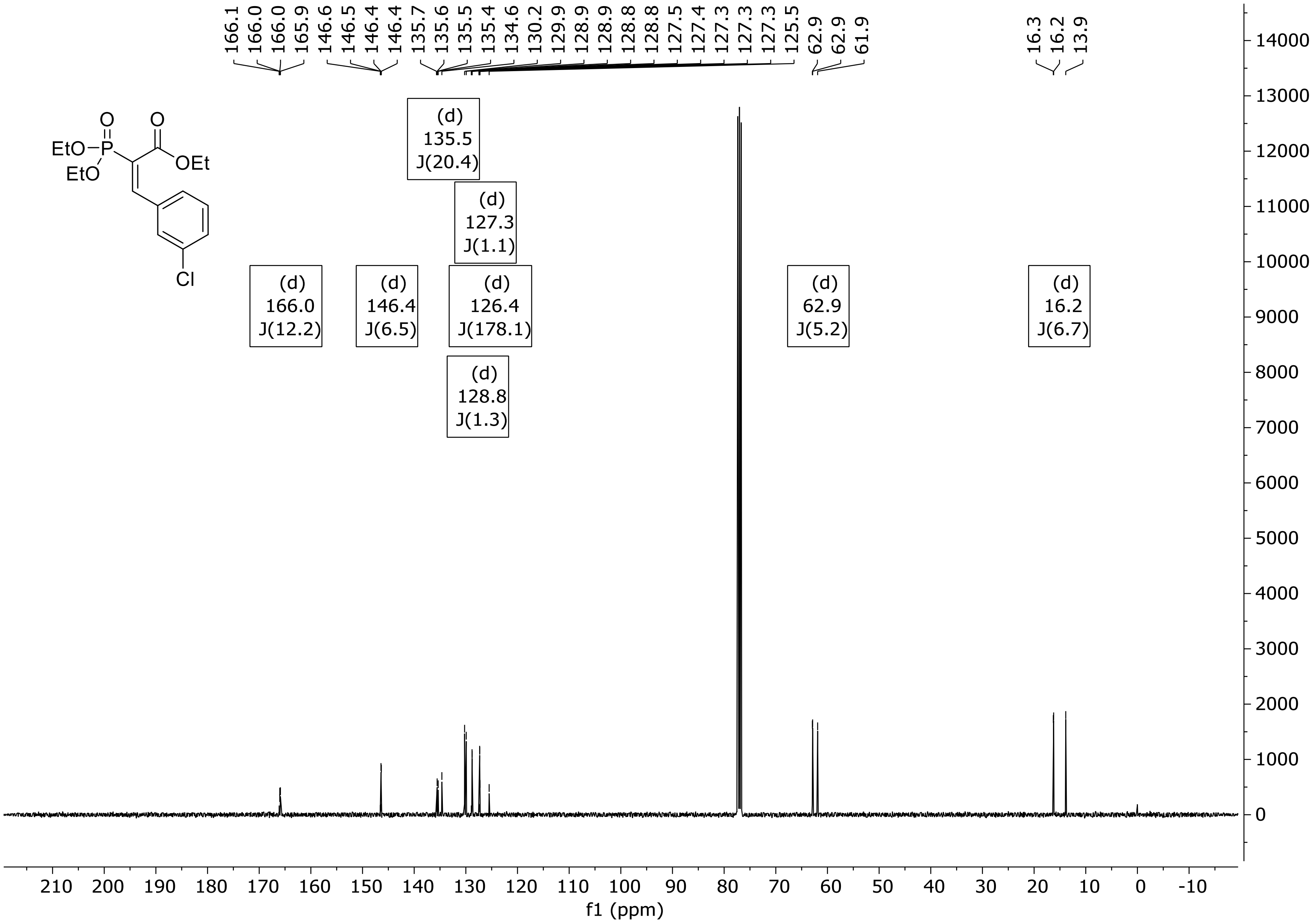

Figure S118. $^{13}C\{^1H\}$ NMR spectrum of **1f** ($CDCl_3$, 101 MHz).

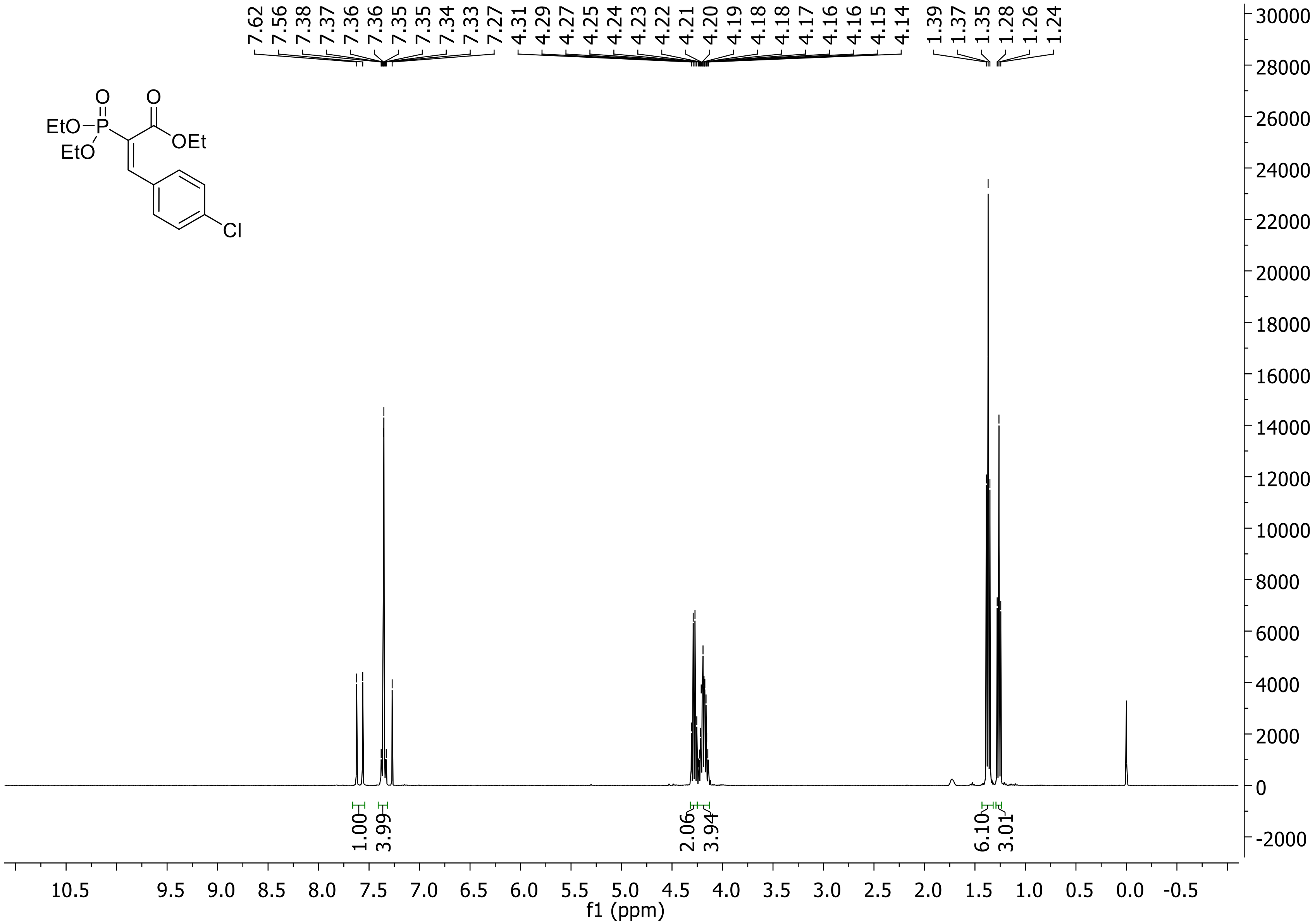

Figure S119. ^{1}H NMR spectrum of **1g** ($CDCl_3$, 400 MHz).

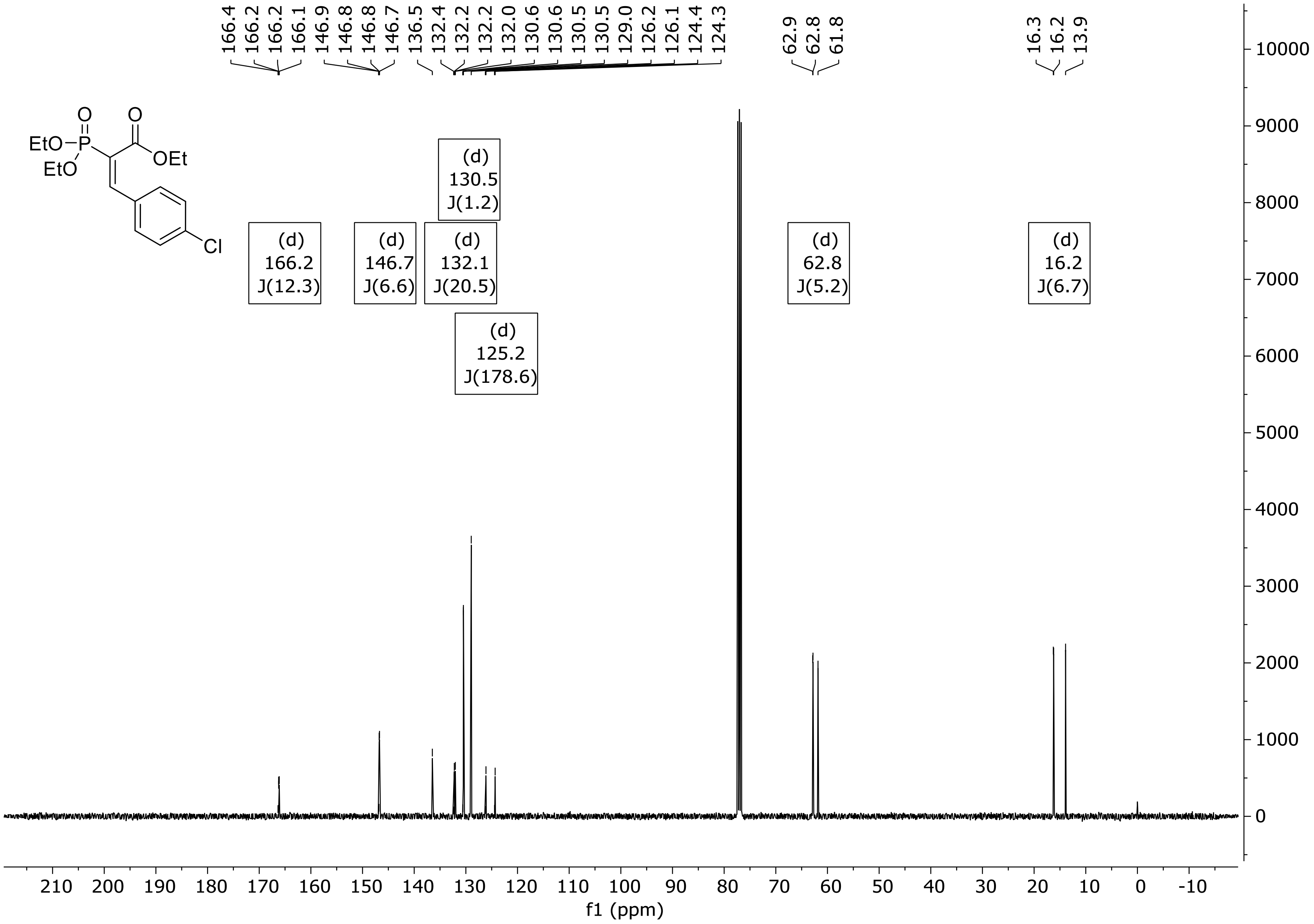

Figure S120. $^{13}C\{^{1}H\}$ NMR spectrum of **1g** ($CDCl_3$, 101 MHz).

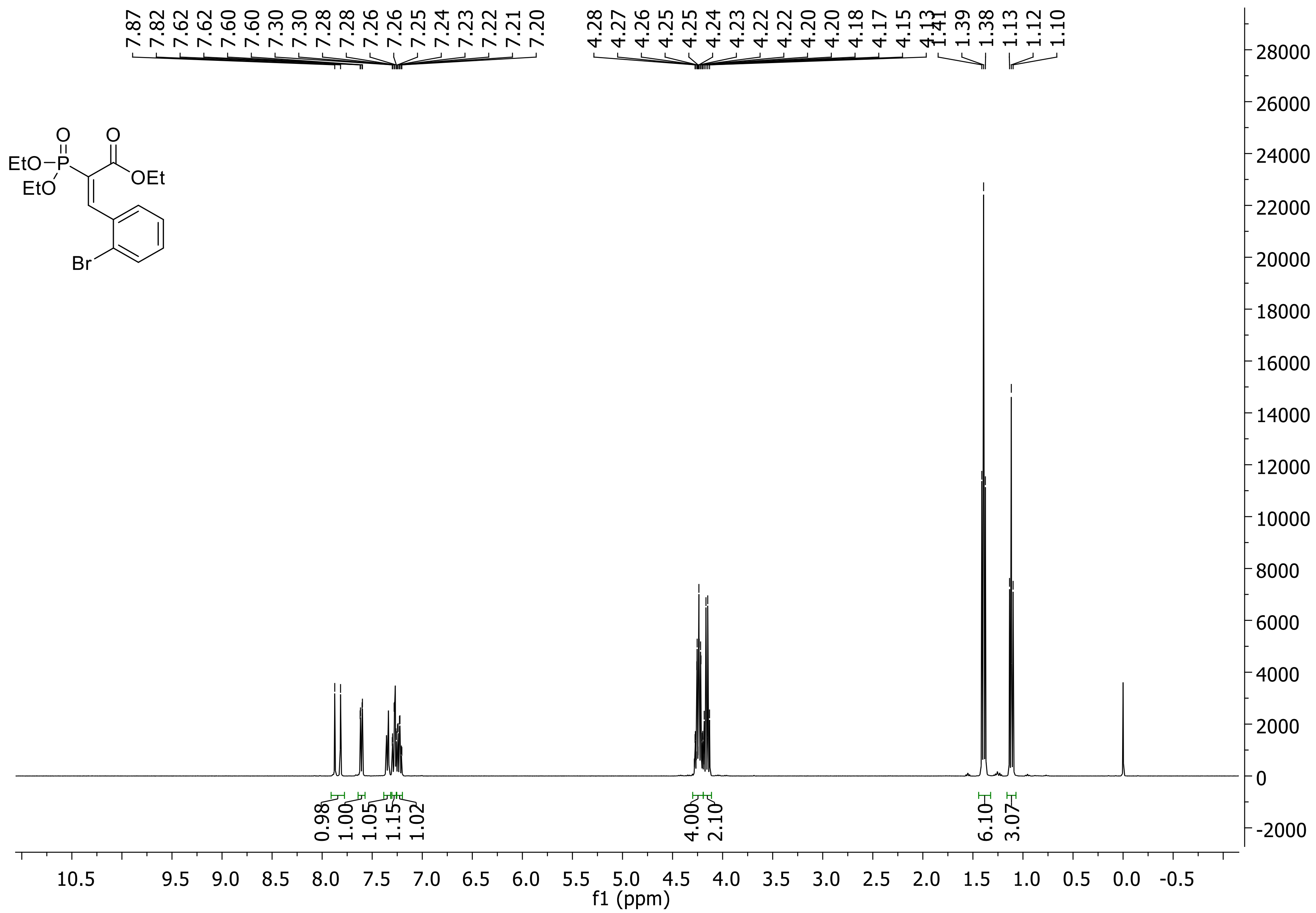

Figure S121. ^{1}H NMR spectrum of **1h** ($CDCl_3$, 400 MHz).

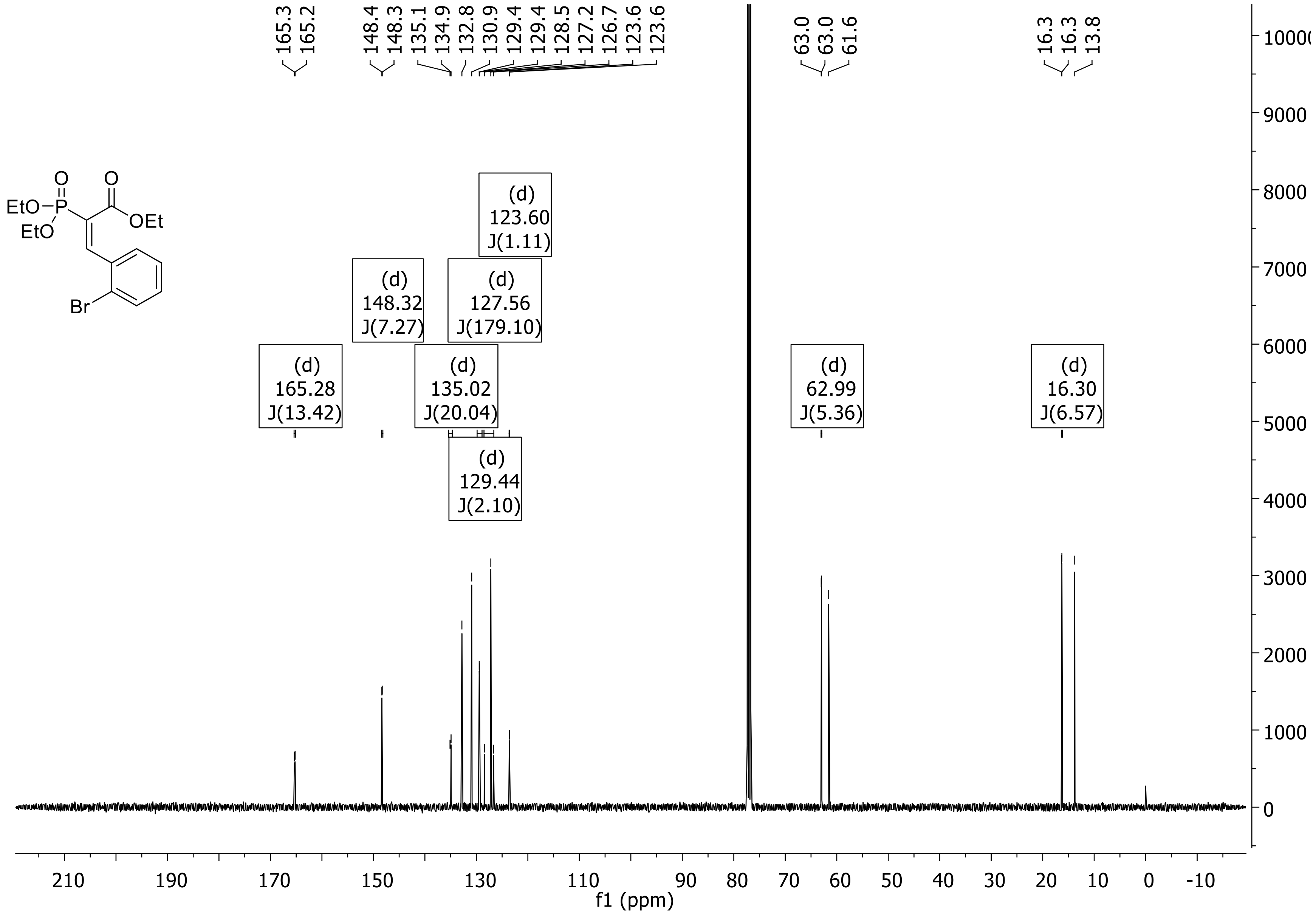

Figure S122. $^{13}C\{^{1}H\}$ NMR spectrum of **1h** ($CDCl_3$, 101 MHz).

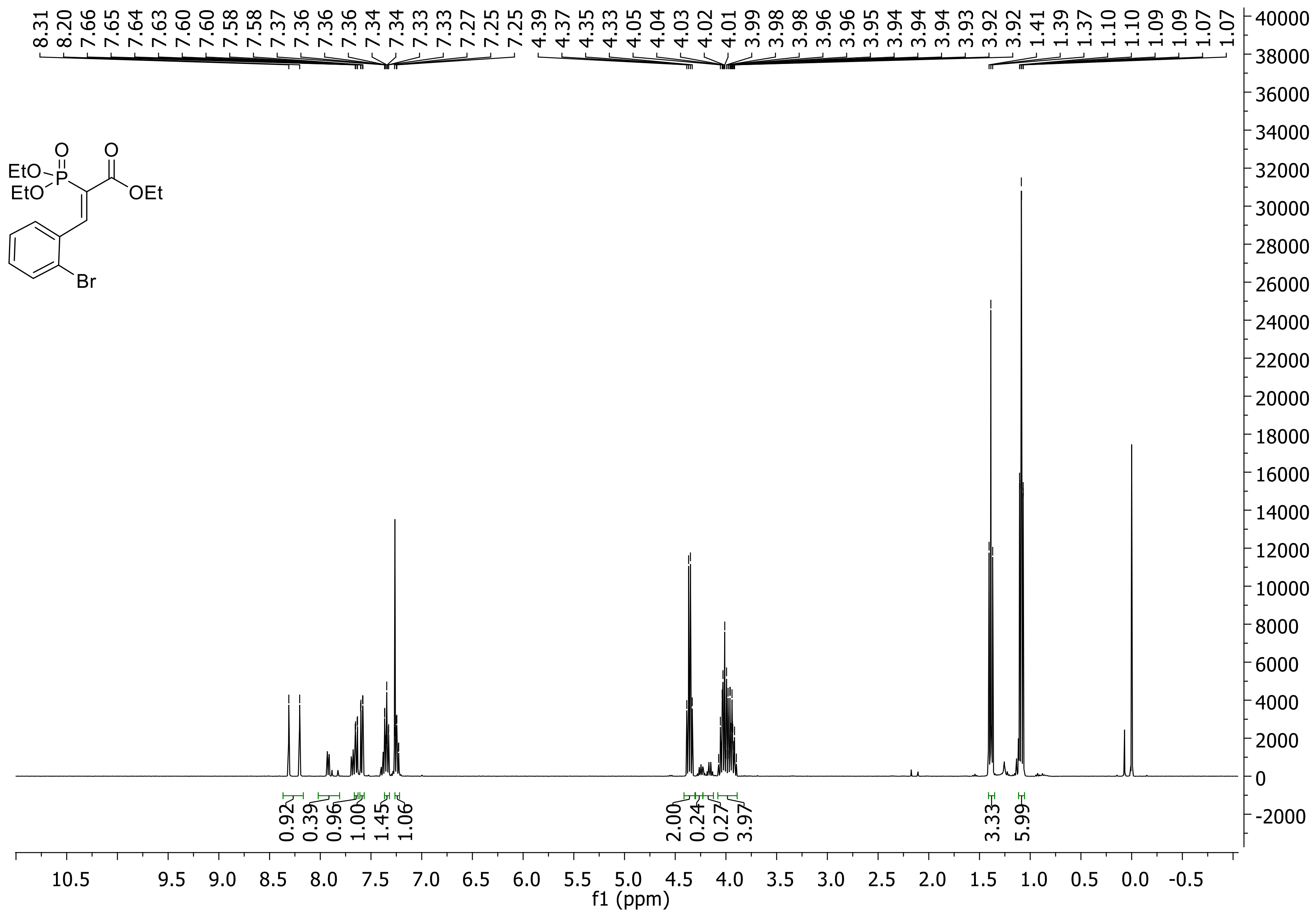

Figure S123. ^{1}H NMR spectrum of ***Z*-1h** ($CDCl_3$, 400 MHz).

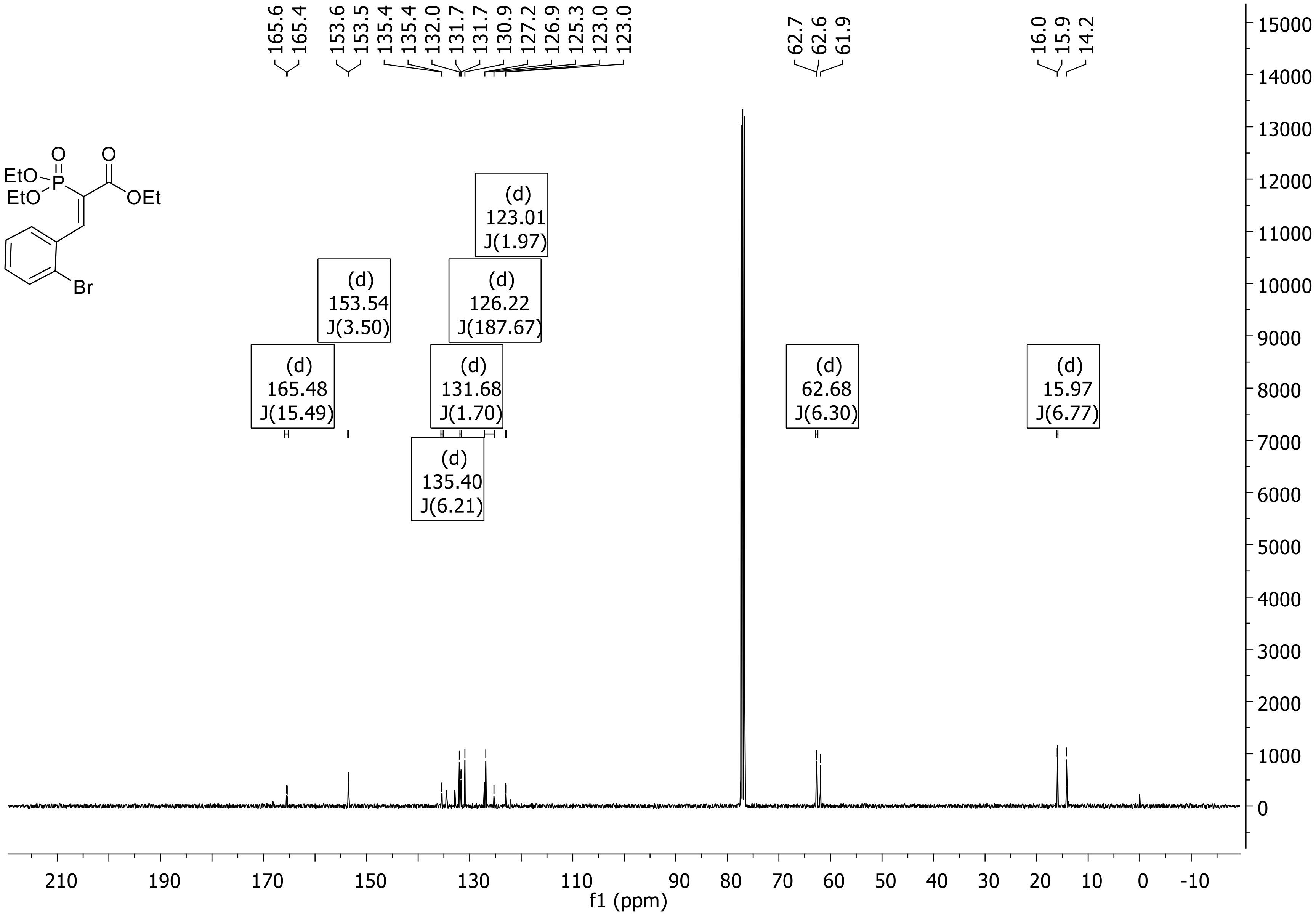

Figure S124. $^{13}C\{^{1}H\}$ NMR spectrum of ***Z*-1h** ($CDCl_3$, 101 MHz).

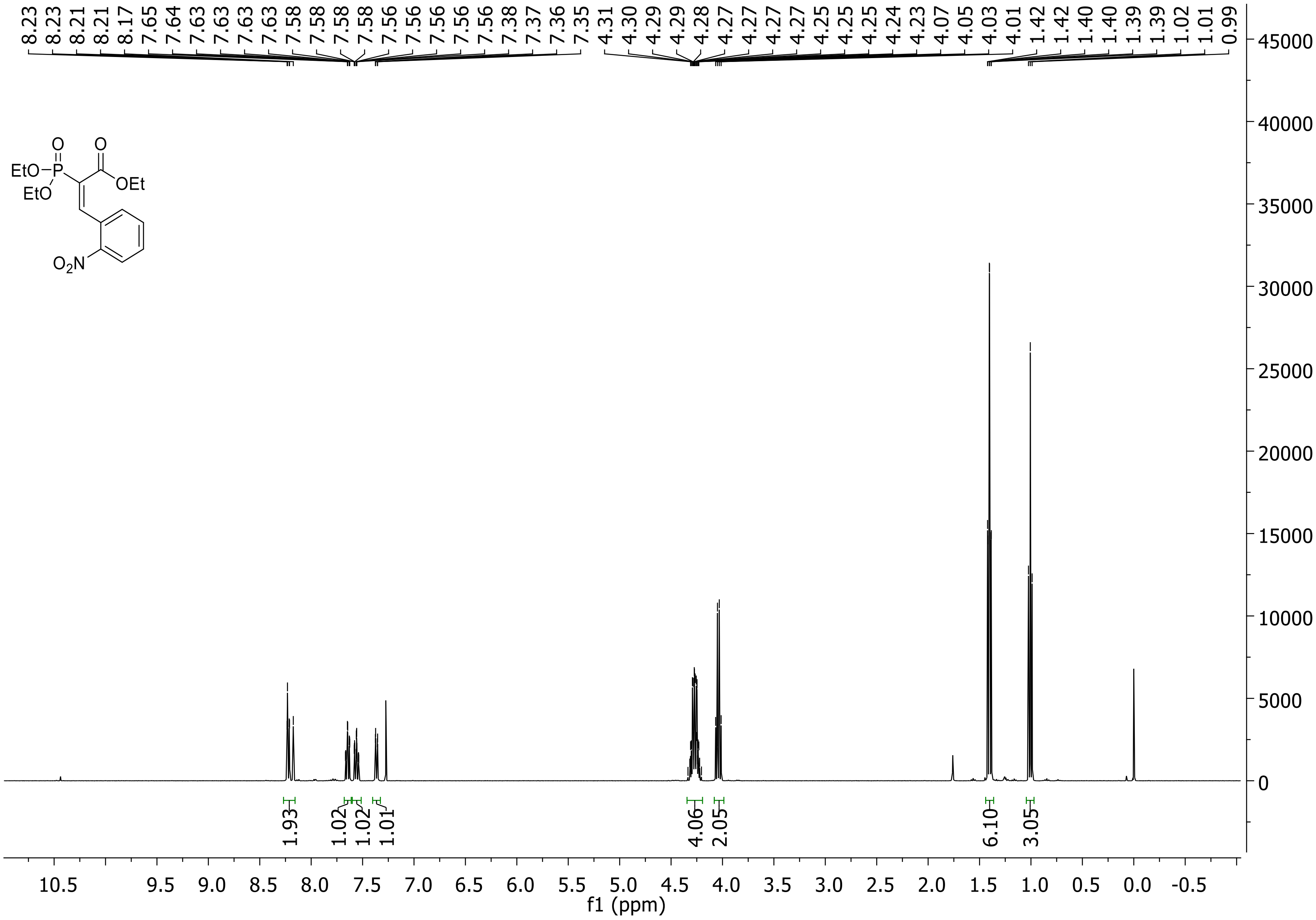

Figure S125. ^{1}H NMR spectrum of **1i** ($CDCl_3$, 400 MHz).

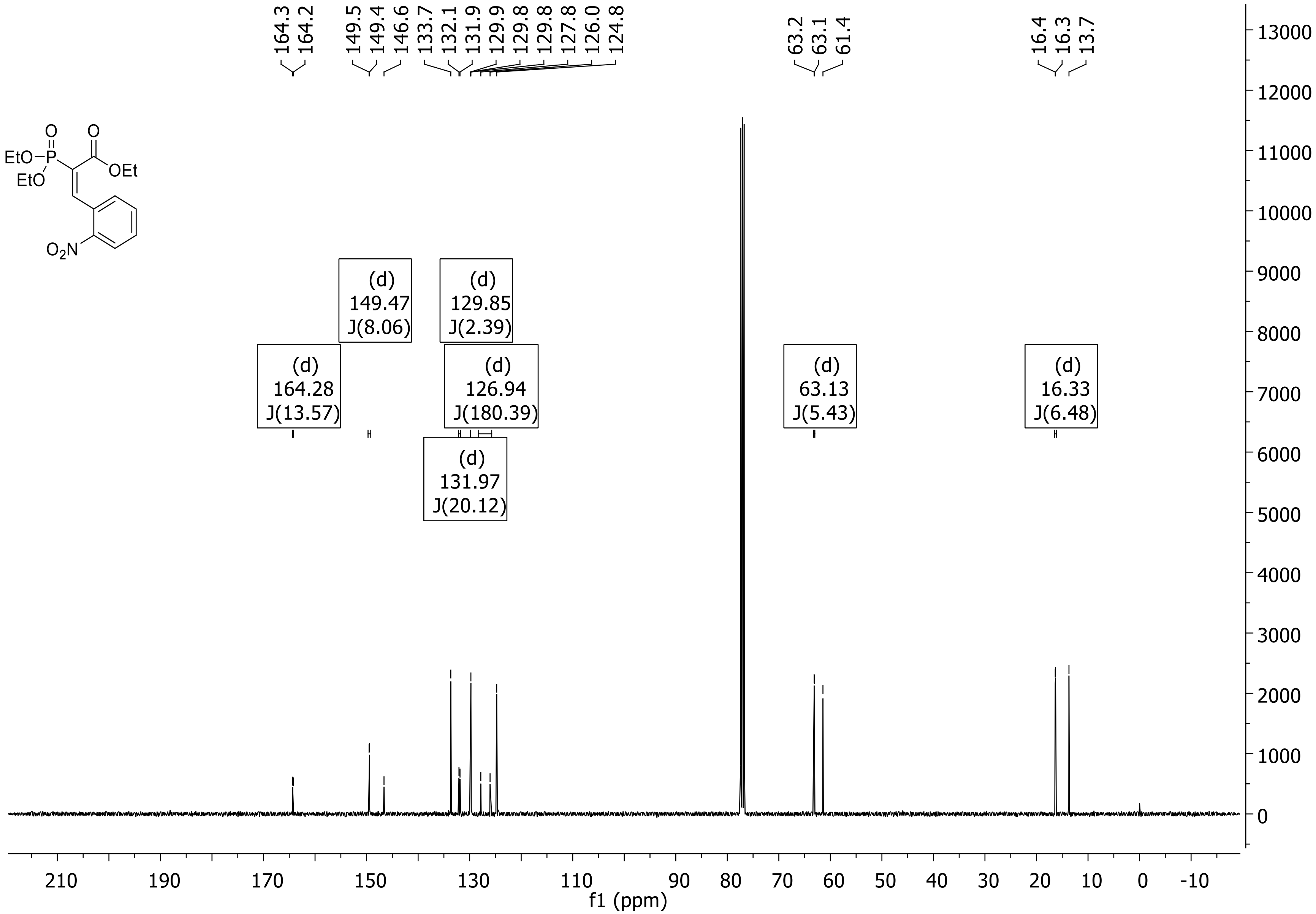

Figure S126. $^{13}C\{^1H\}$ NMR spectrum of **1i** ($CDCl_3$, 101 MHz).

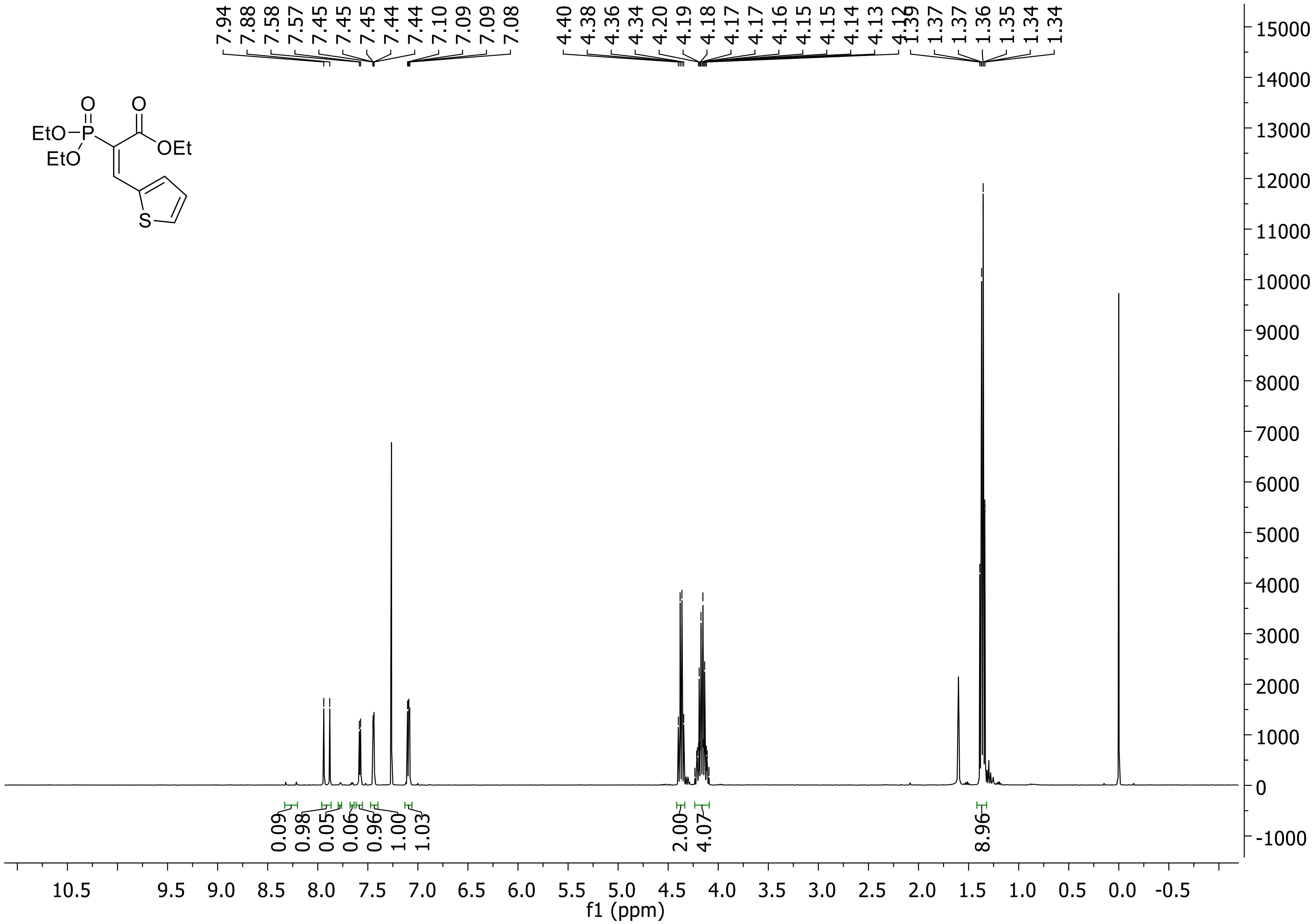

Figure S127. ^{1}H NMR spectrum of **1j** ($CDCl_3$, 400 MHz).

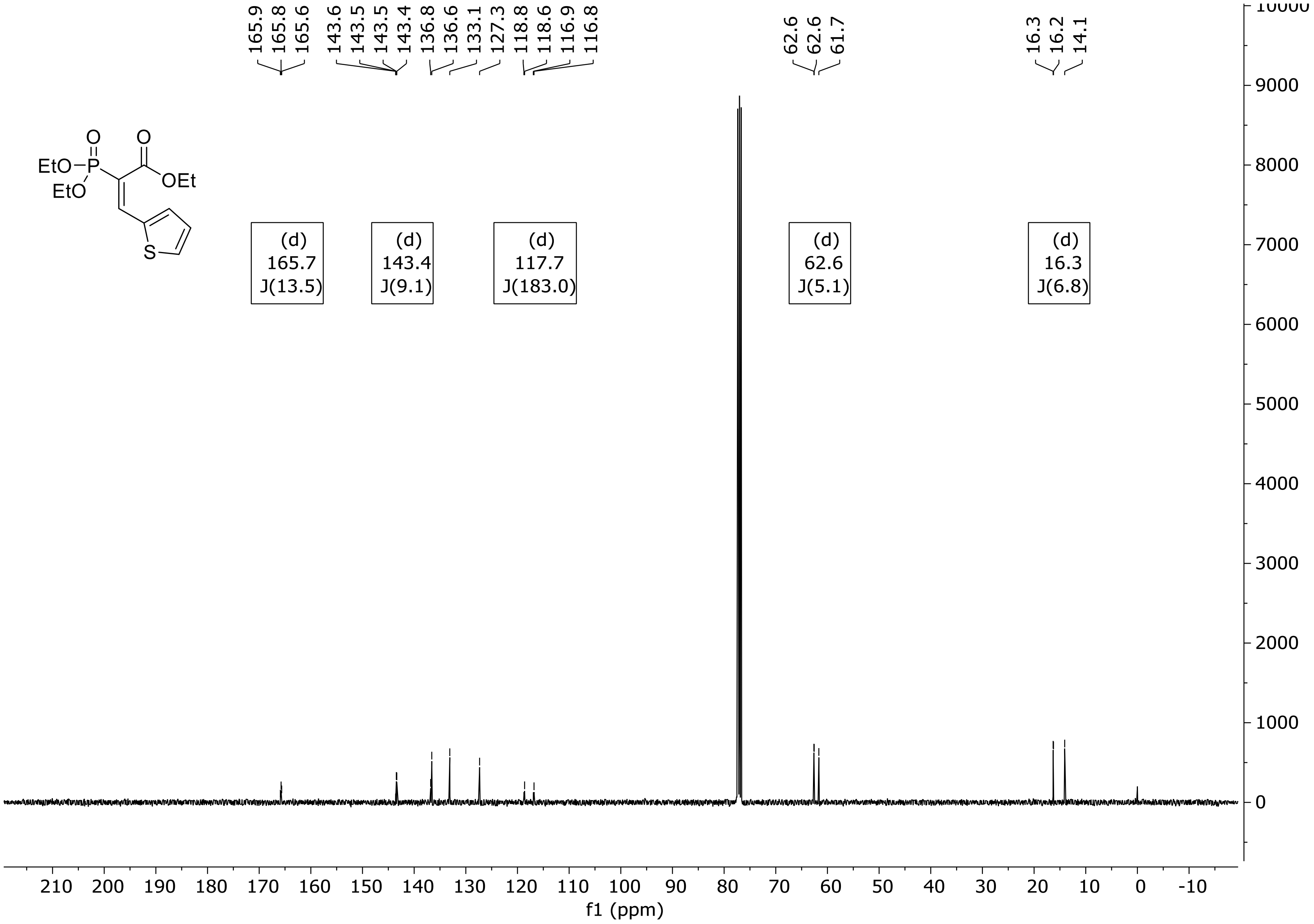

Figure S128. $^{13}C\{^1H\}$ NMR spectrum of **1j** ($CDCl_3$, 101 MHz).

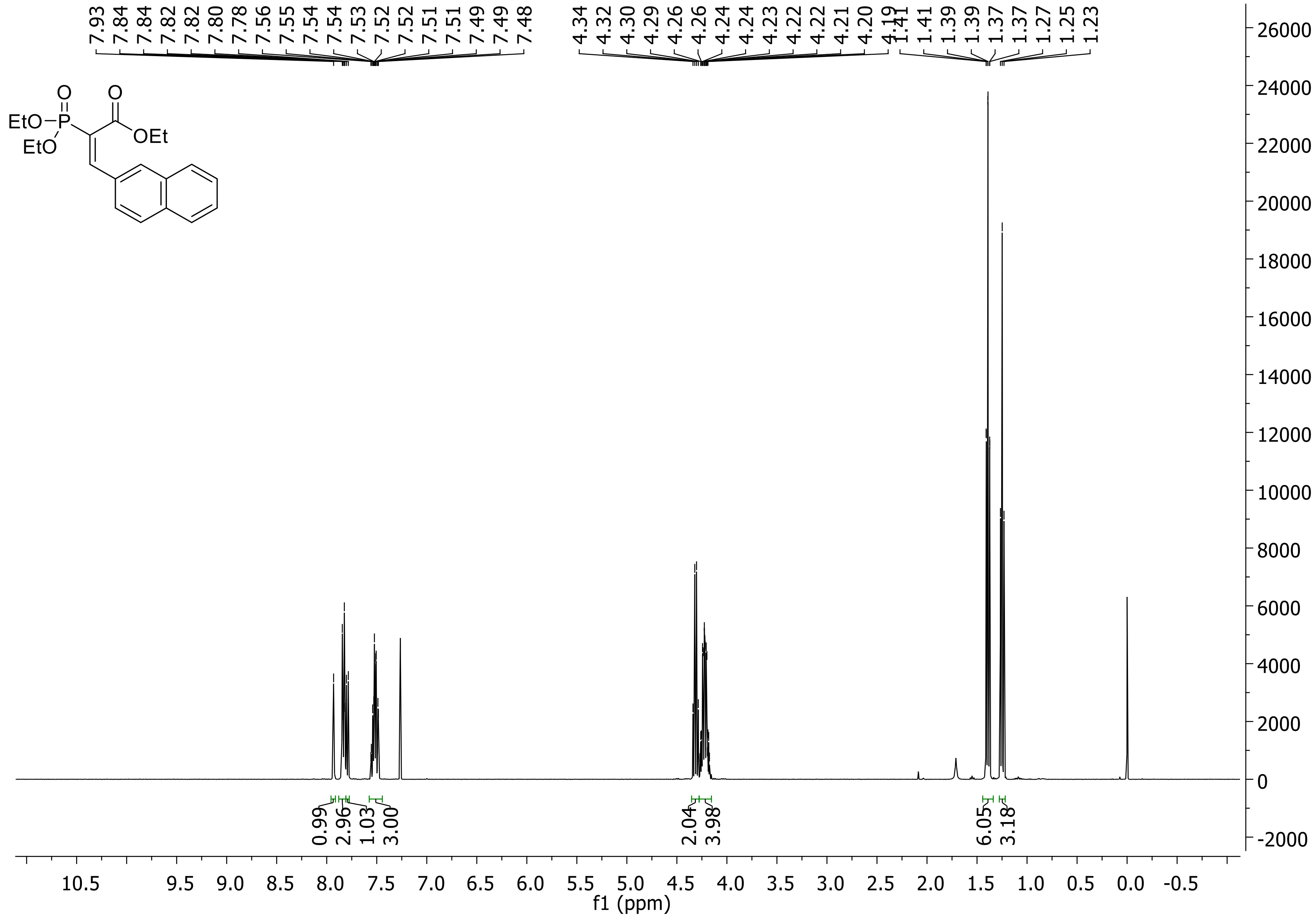

Figure S129. ^{1}H NMR spectrum of **1k** ($CDCl_3$, 400 MHz).

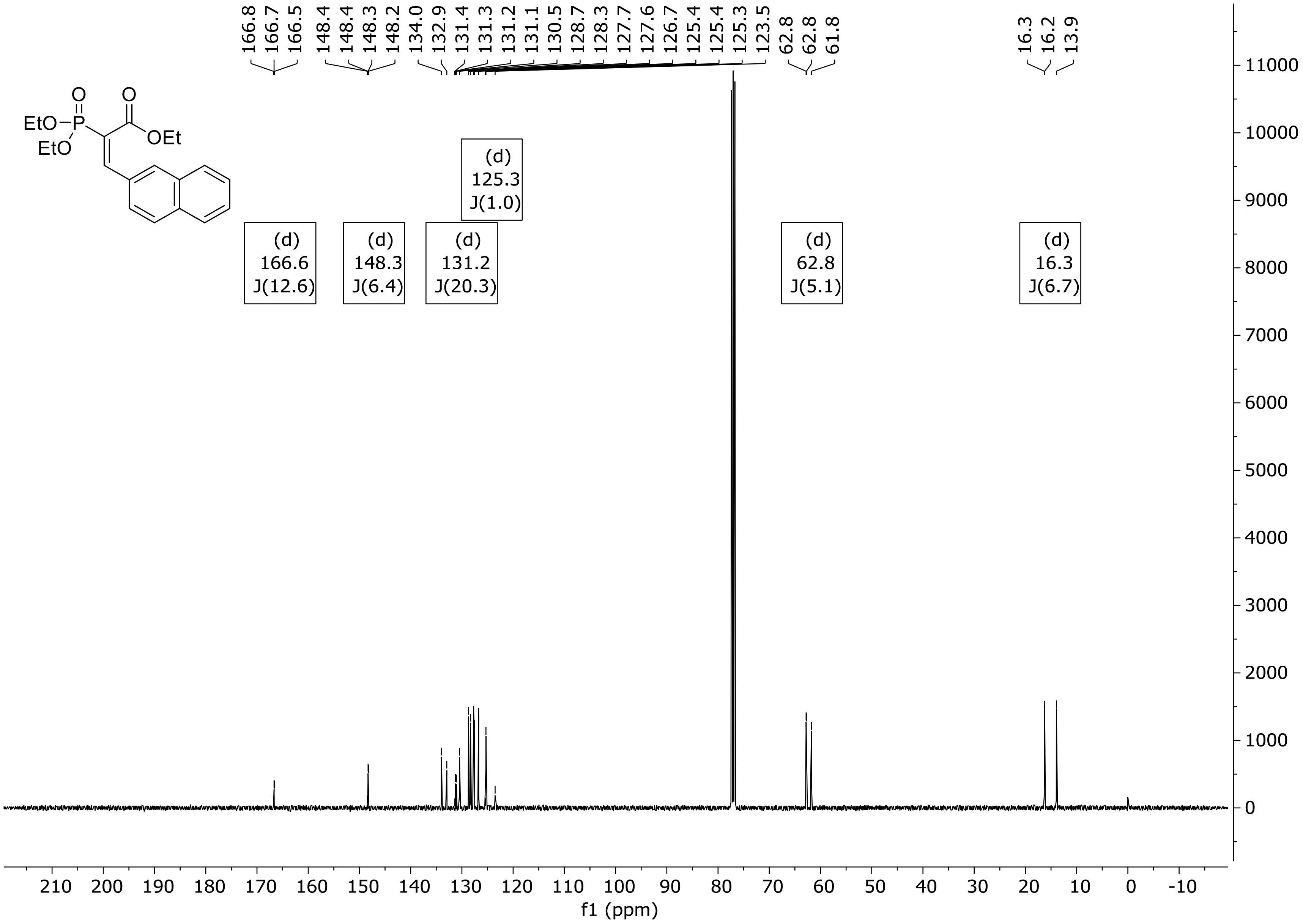

Figure S130. $^{13}C\{^{1}H\}$ NMR spectrum of **1k** ($CDCl_3$, 101 MHz).

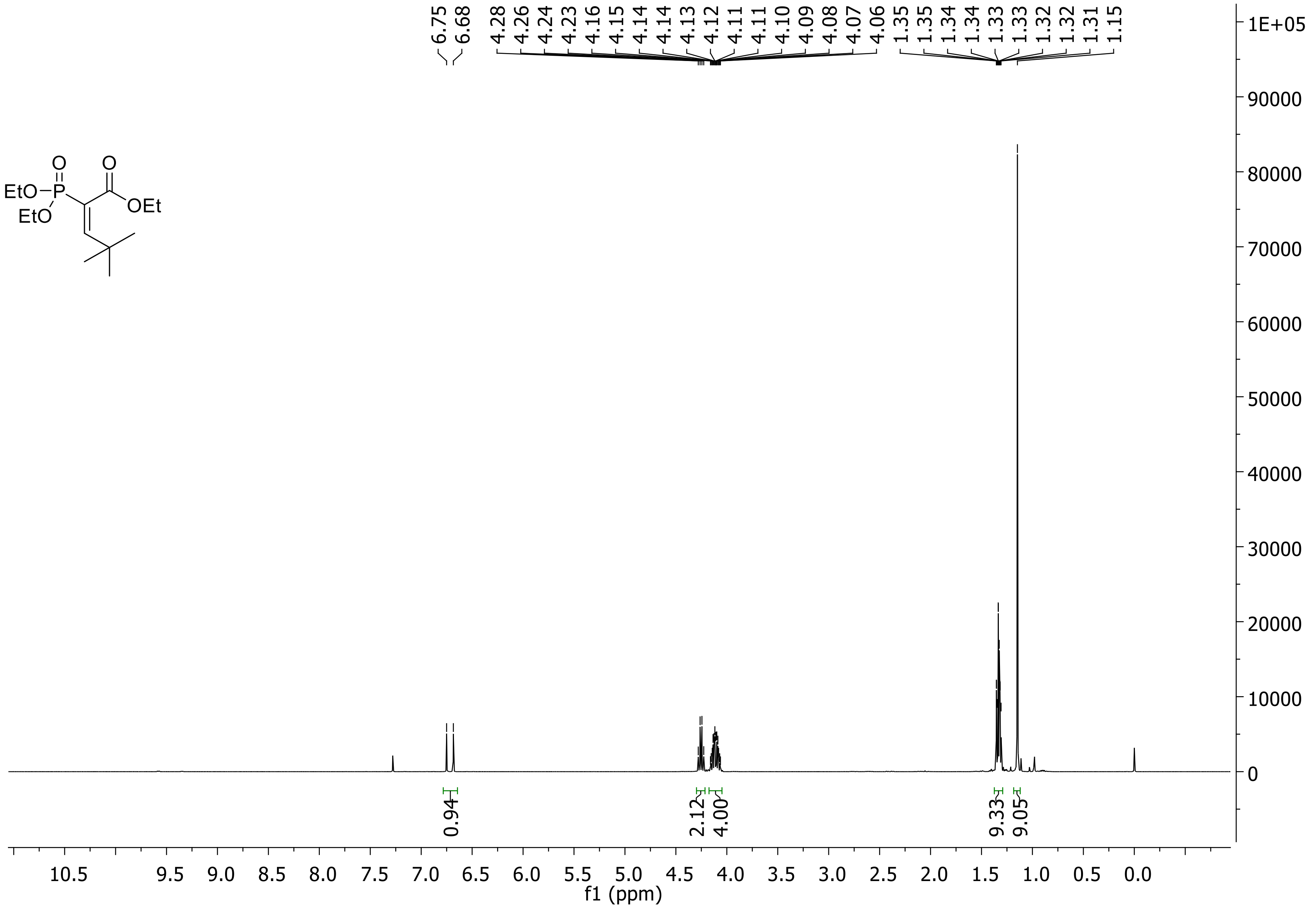

Figure S131. ^{1}H NMR spectrum of **1m** ($CDCl_3$, 400 MHz).

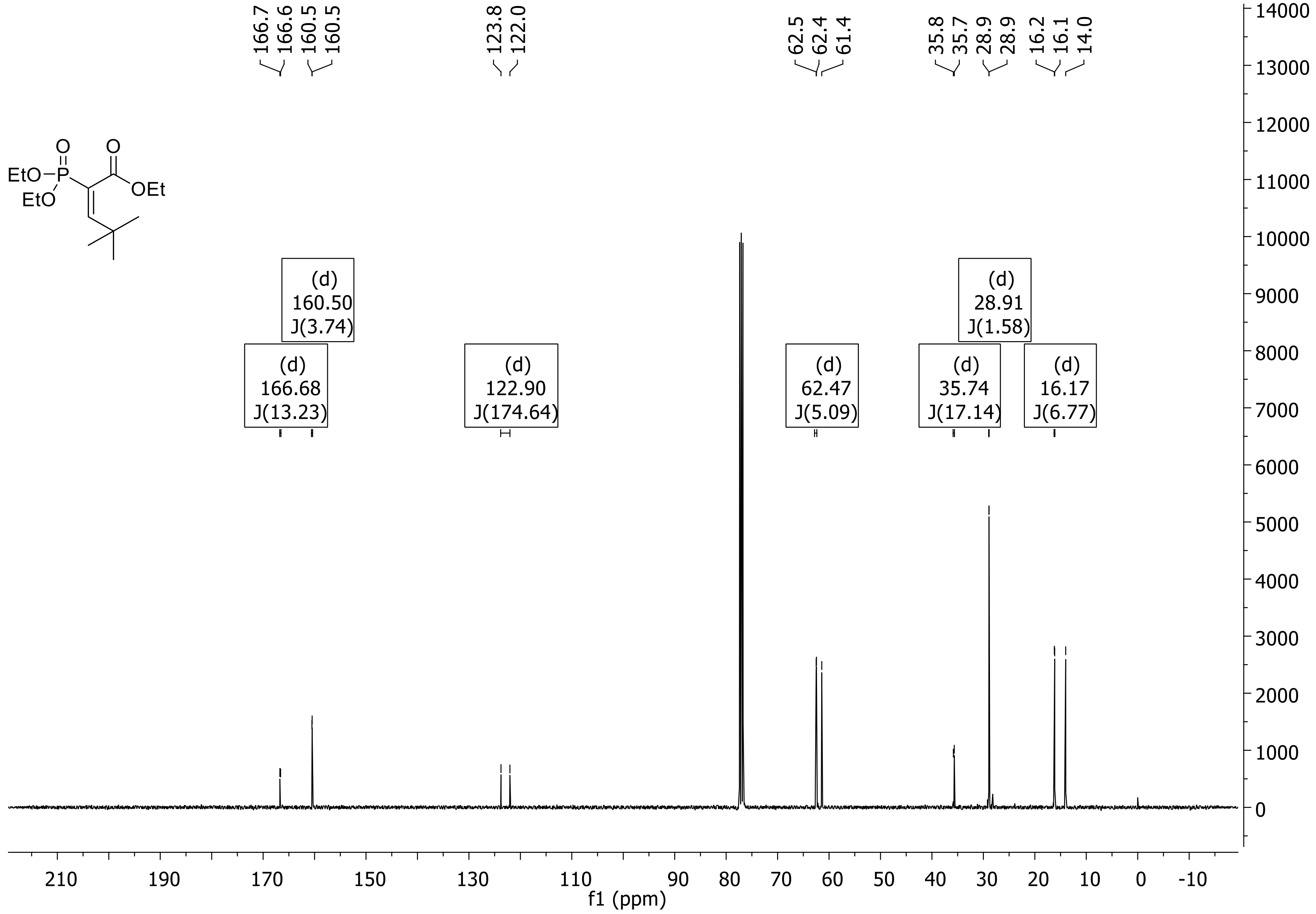

Figure S132. $^{13}C\{^{1}H\}$ NMR spectrum of **1m** ($CDCl_3$, 101 MHz).

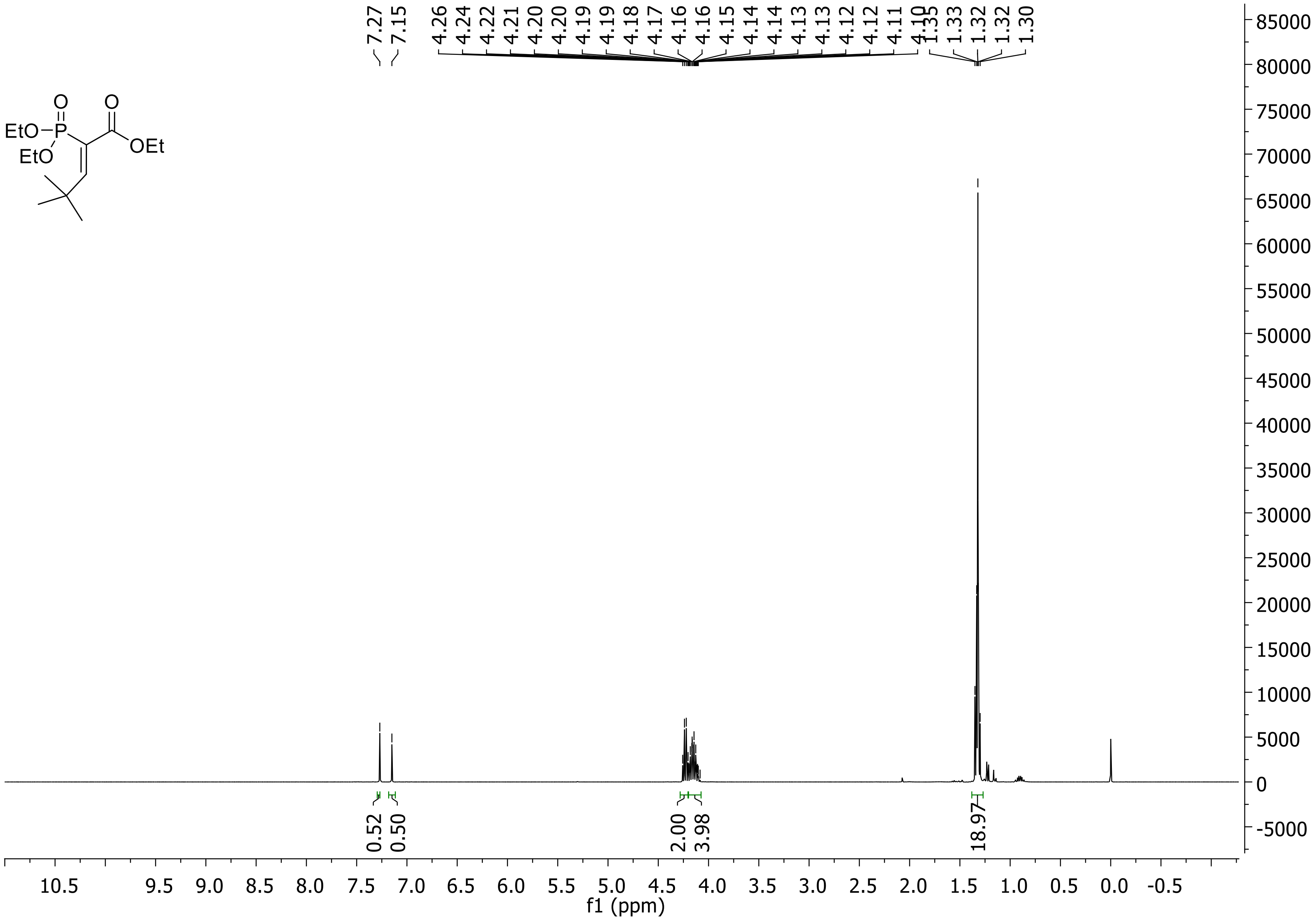

Figure S133. ^{1}H NMR spectrum of ***Z*-1m** ($CDCl_3$, 400 MHz).

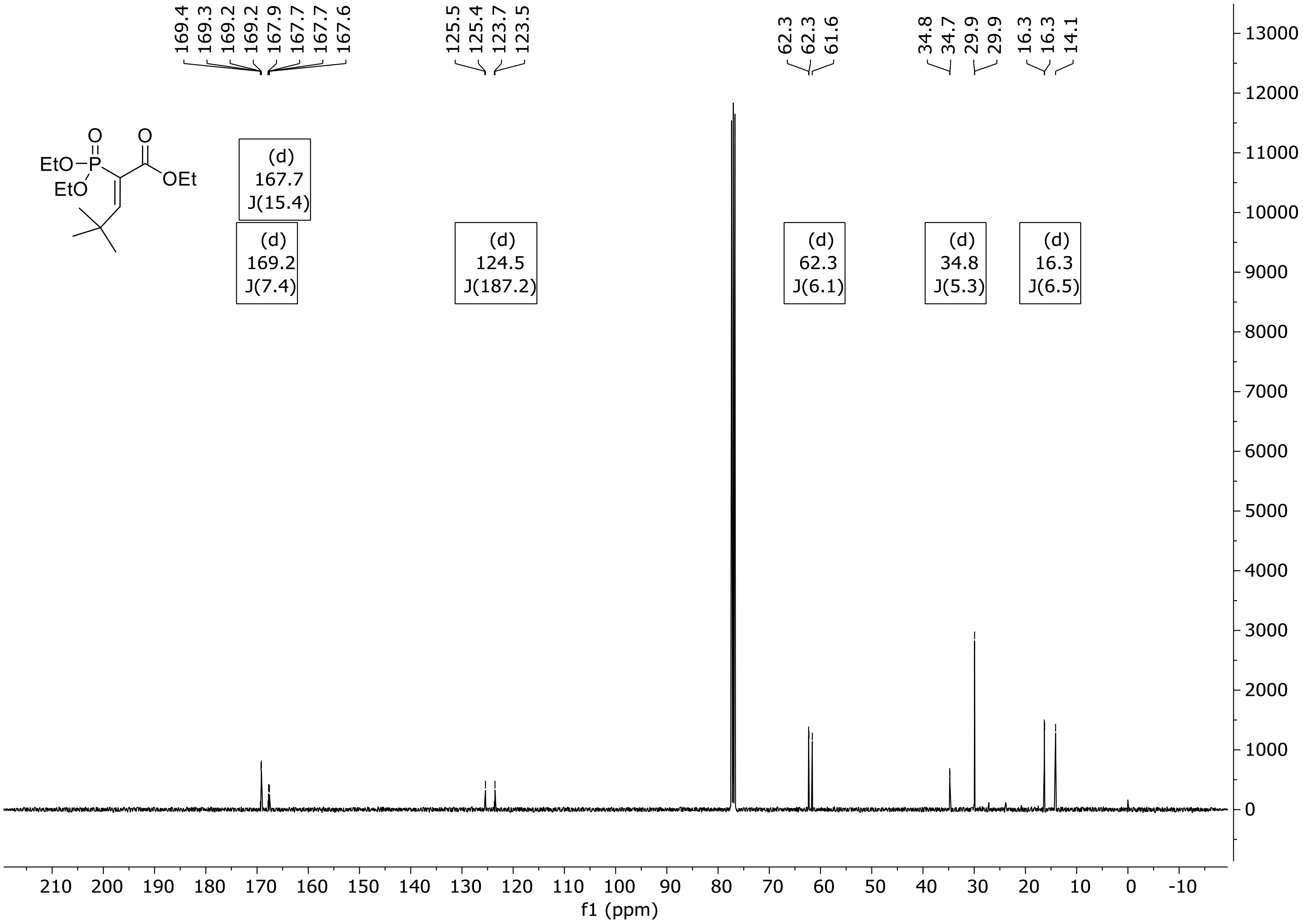

Figure S134. $^{13}C\{^{1}H\}$ NMR spectrum of ***Z*-1m** ($CDCl_3$, 101 MHz).

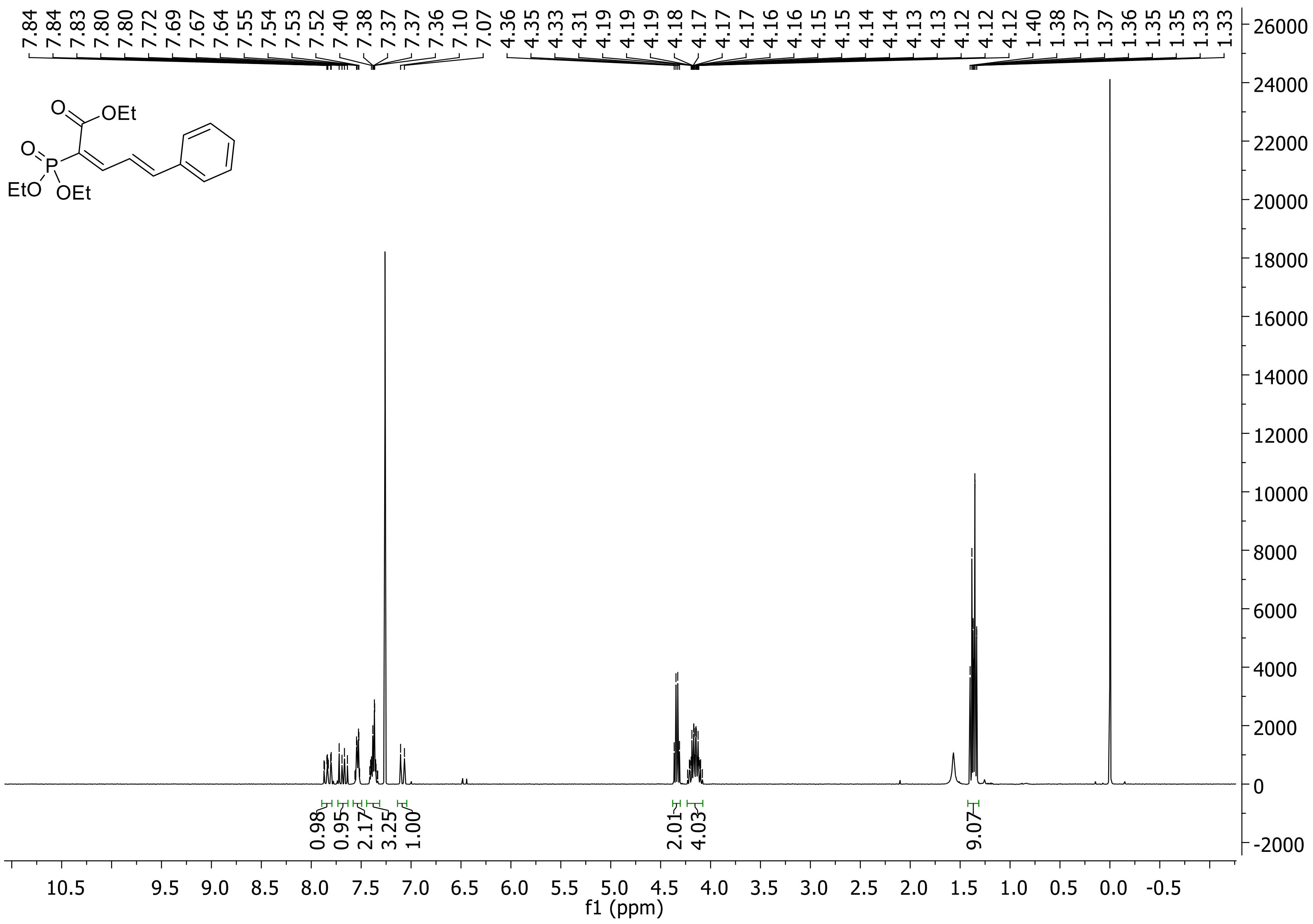

Figure S135. ^{1}H NMR spectrum of **1l** ($CDCl_3$, 400 MHz).

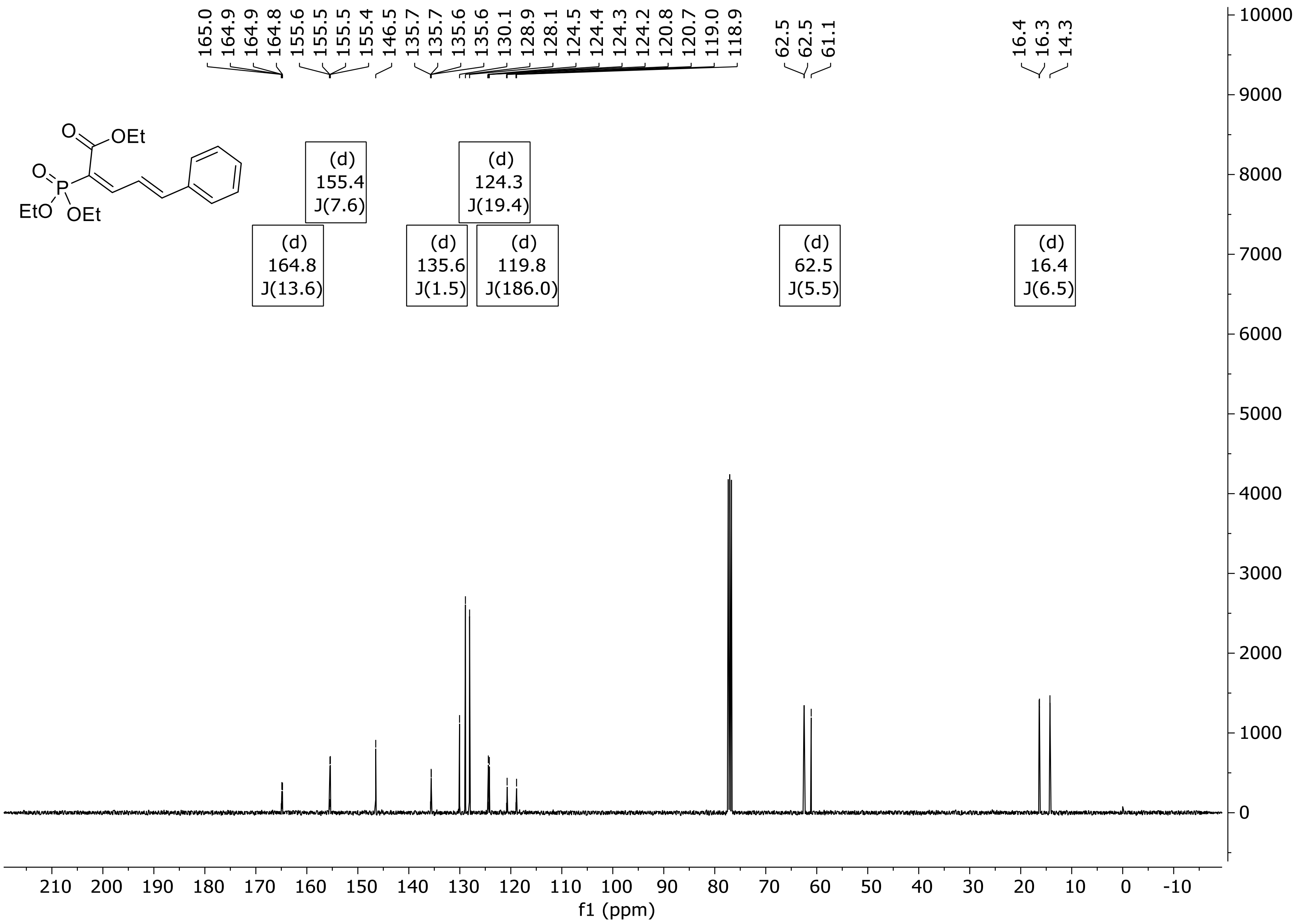

Figure S136. $^{13}C\{^{1}H\}$ NMR spectrum of **1l** ($CDCl_3$, 101 MHz).

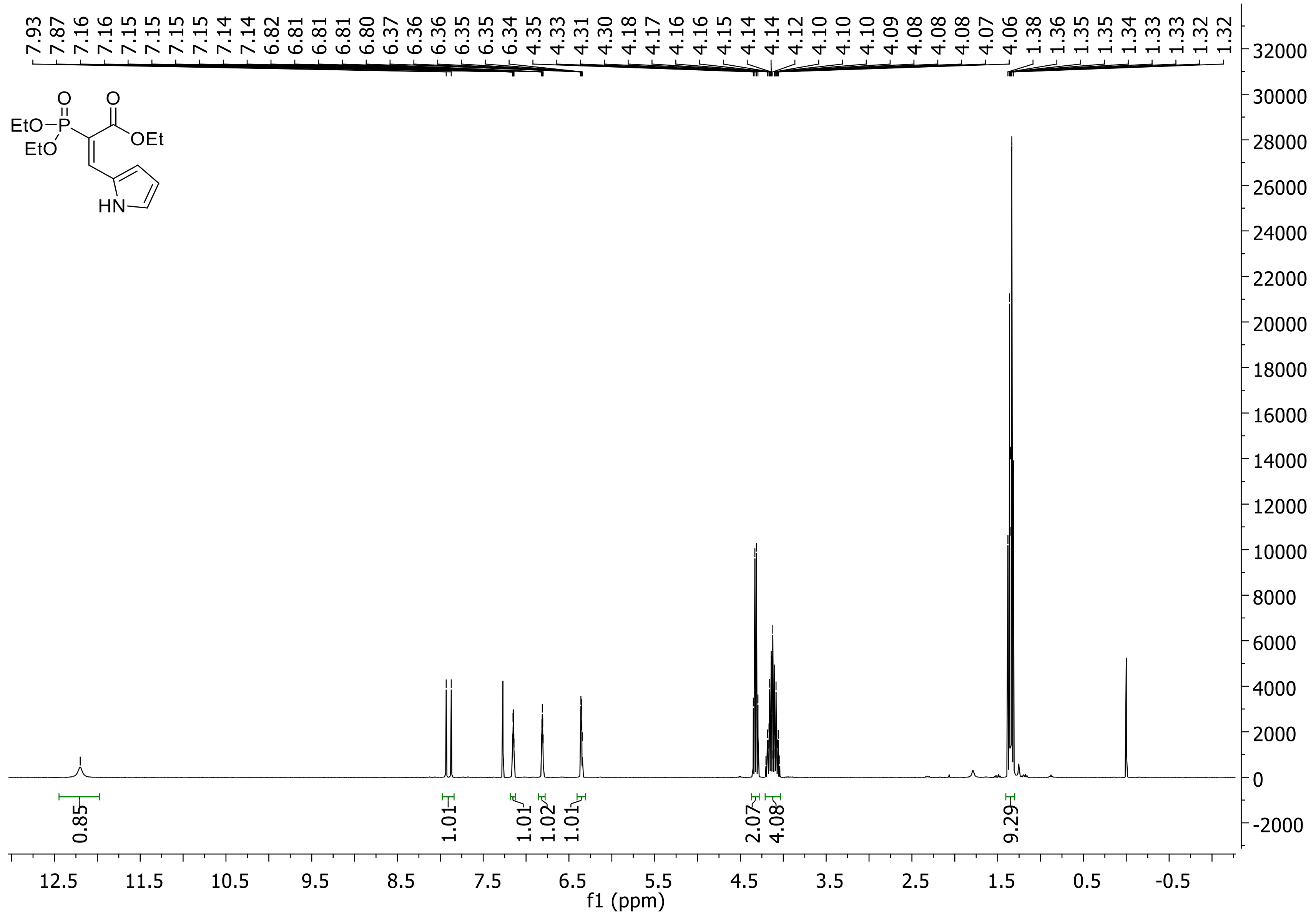

Figure S137. ^{1}H NMR spectrum of **1n** ($CDCl_3$, 400 MHz).

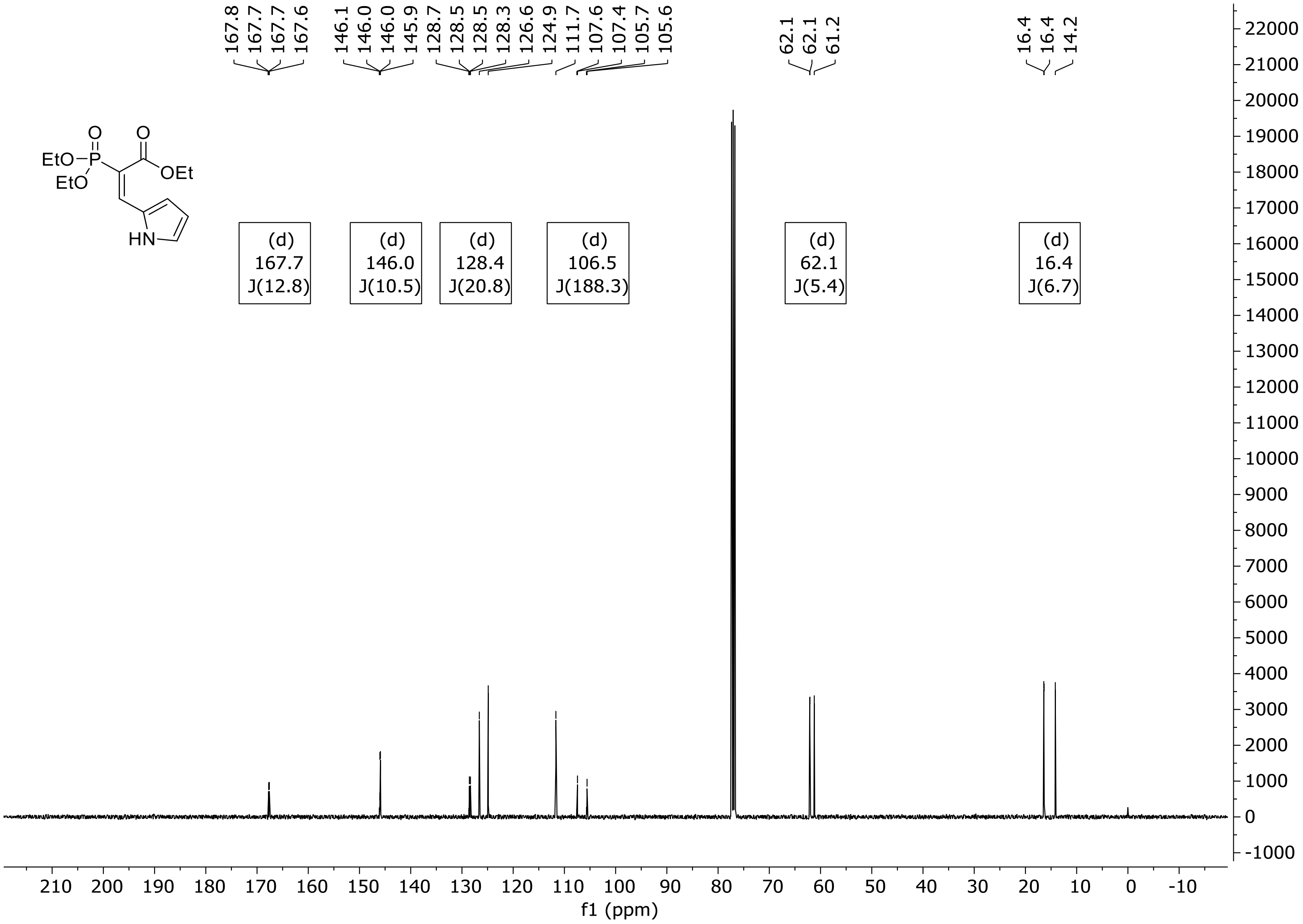

Figure S138. $^{13}C\{^{1}H\}$ NMR spectrum of **1n** ($CDCl_3$, 101 MHz).

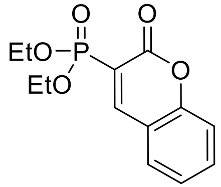

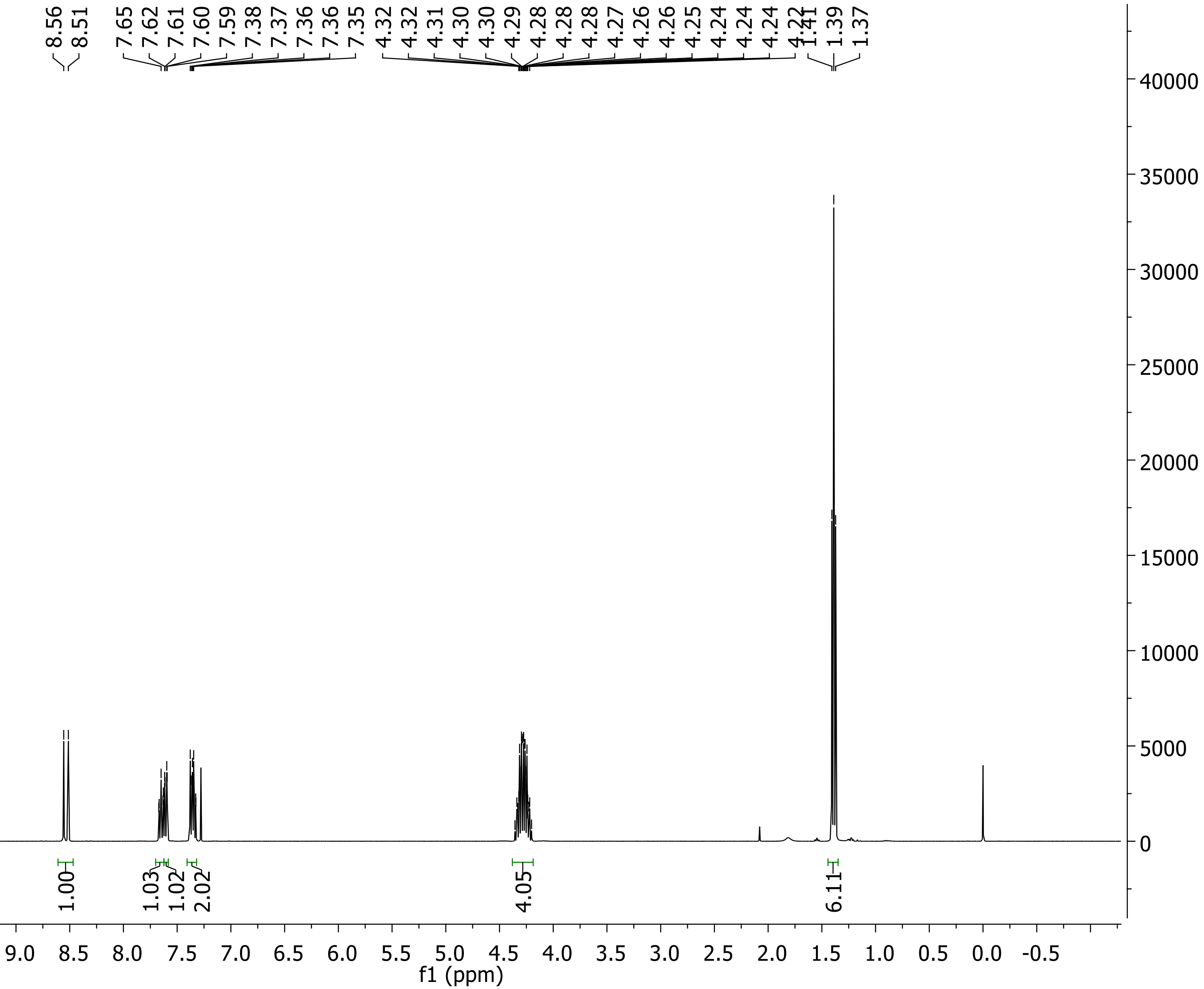

Figure S139. ^{1}H NMR spectrum of **1o** ($CDCl_3$, 400 MHz).

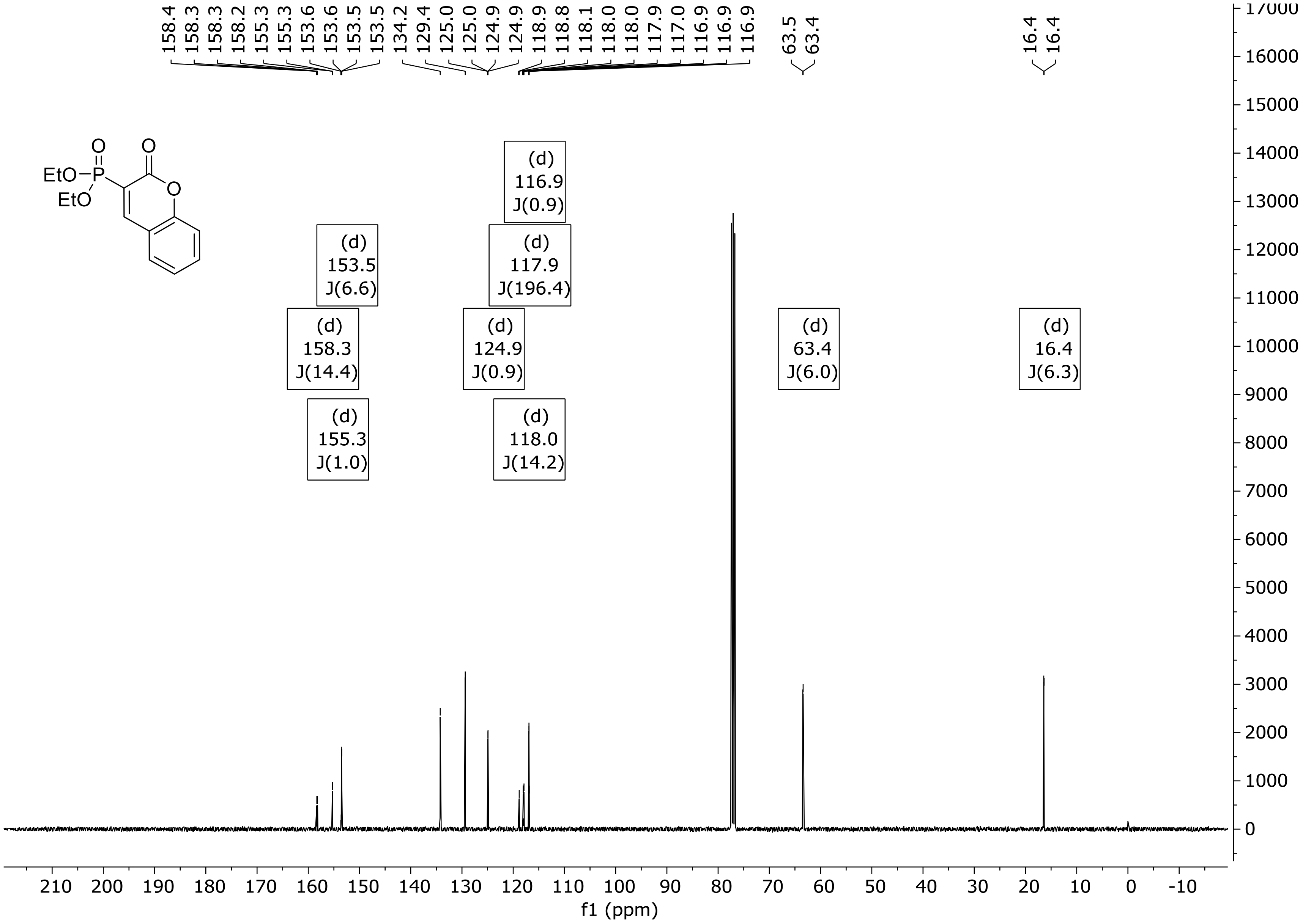

Figure S140. $^{13}C\{^{1}H\}$ NMR spectrum of **1o** ($CDCl_3$, 101 MHz).

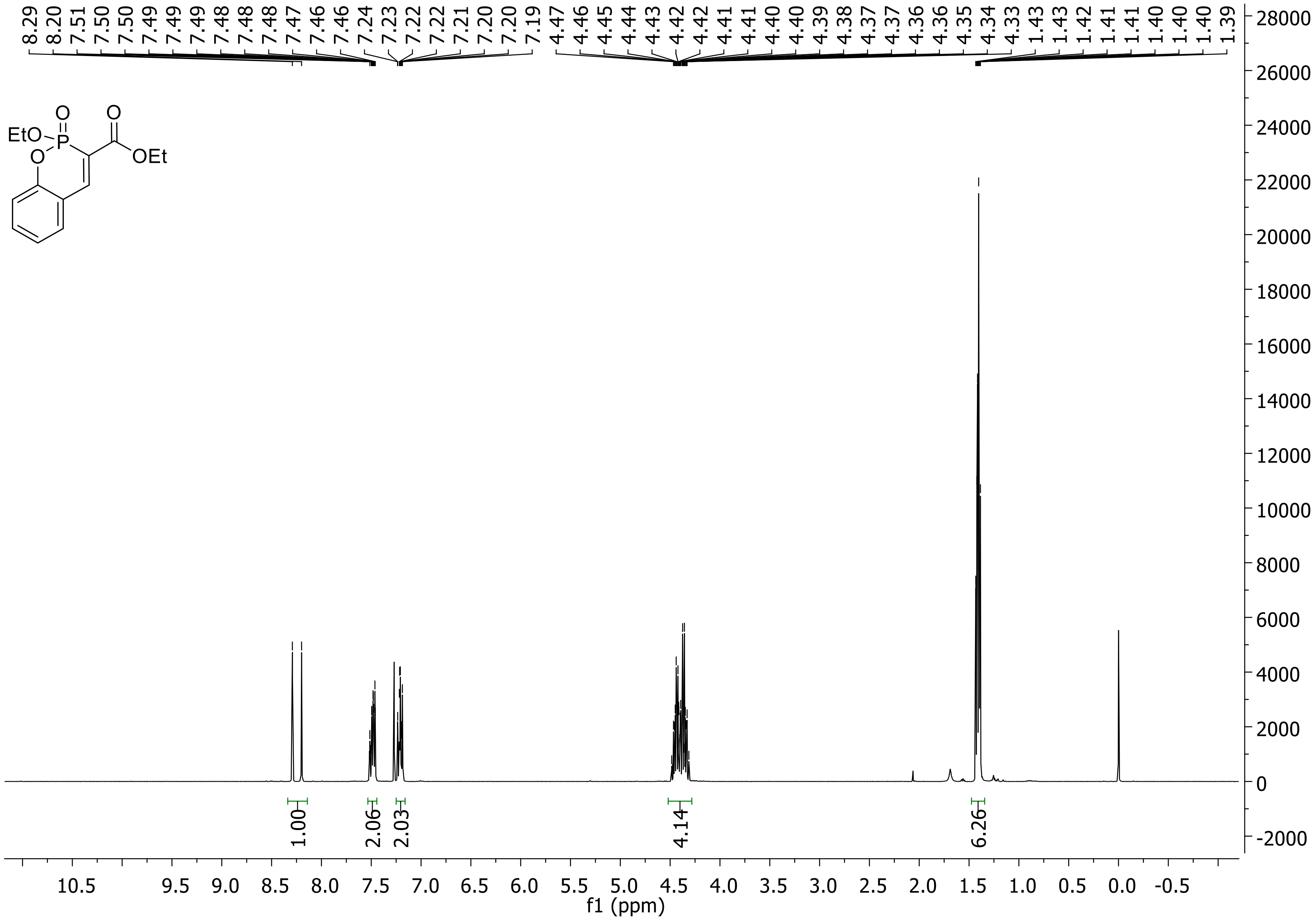

Figure S141. ^{1}H NMR spectrum of **1p** ($CDCl_3$, 400 MHz).

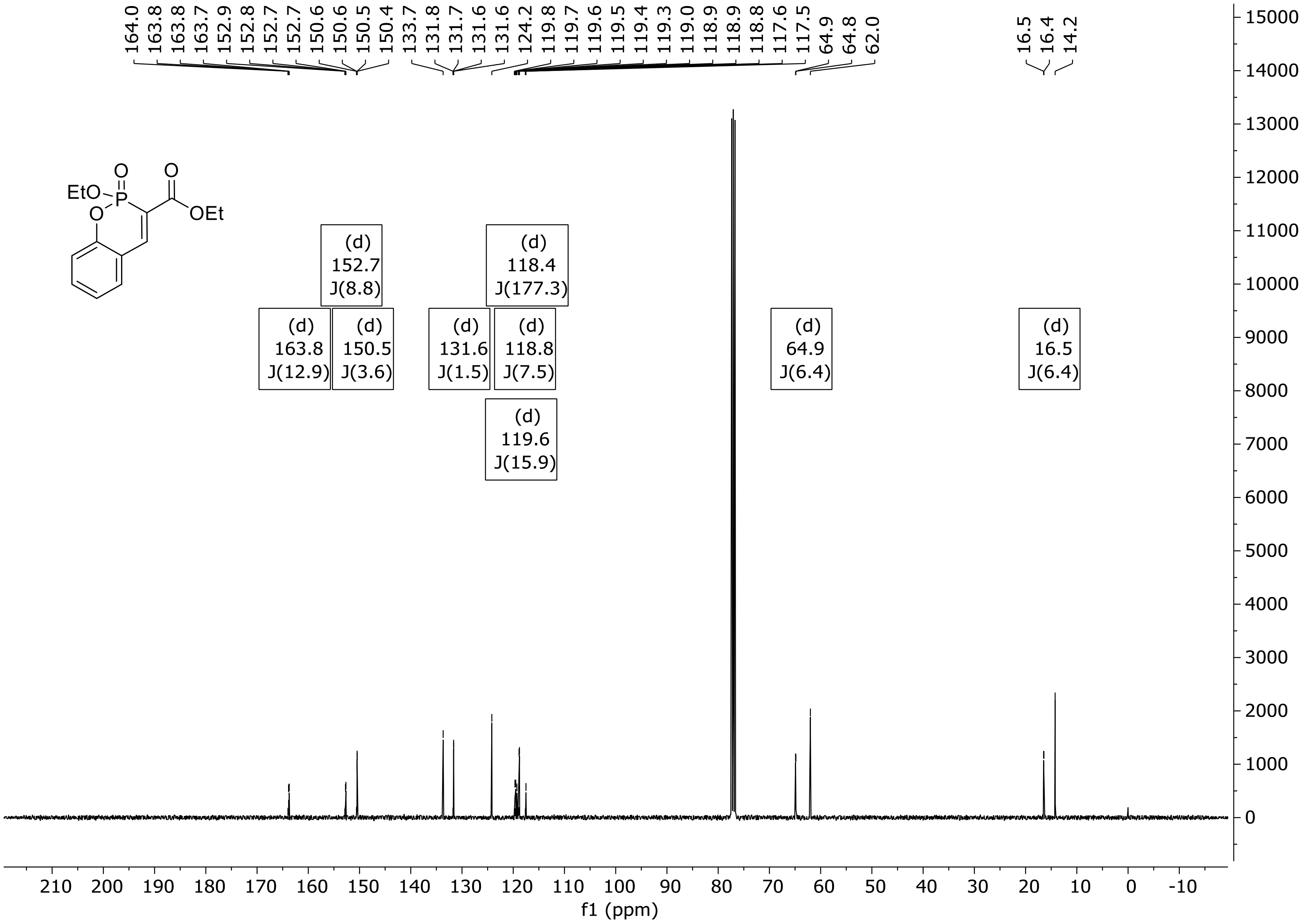

Figure S142. $^{13}C\{^{1}H\}$ NMR spectrum of **1p** ($CDCl_3$, 101 MHz).

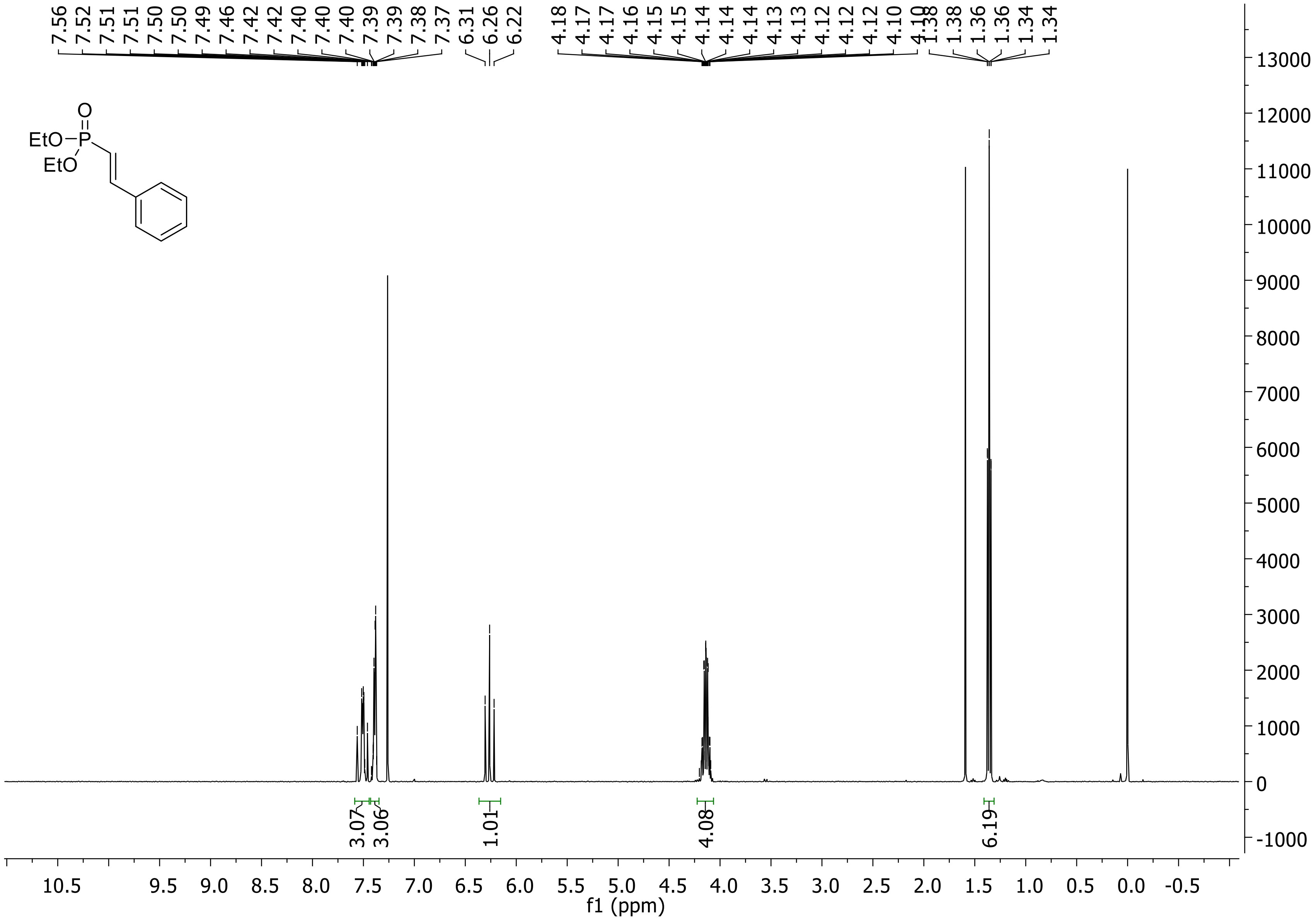

Figure S143. ^{1}H NMR spectrum of **8** ($CDCl_3$, 400 MHz).

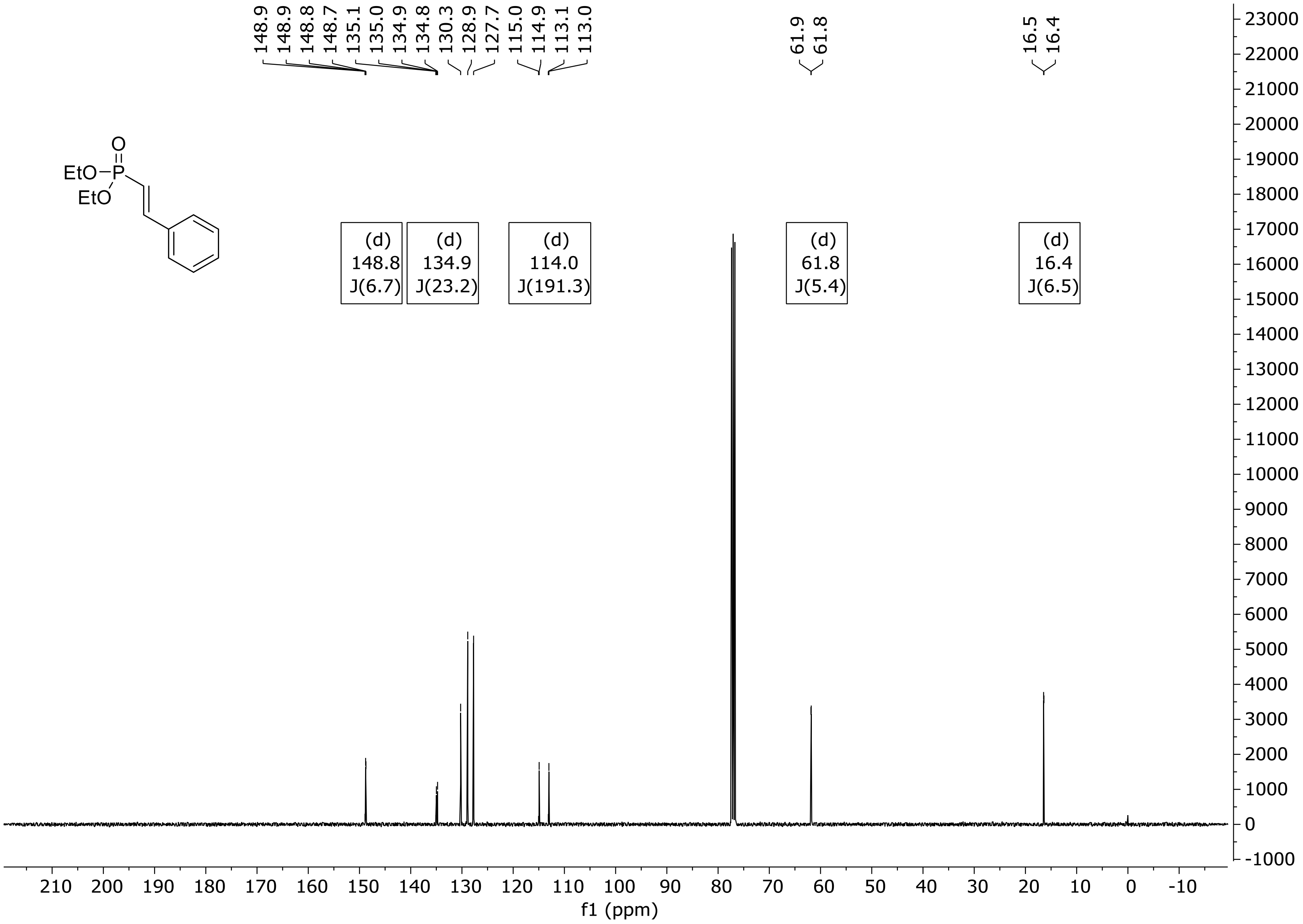

Figure S144. $^{13}C\{^1H\}$ NMR spectrum of **8** ($CDCl_3$, 101 MHz).

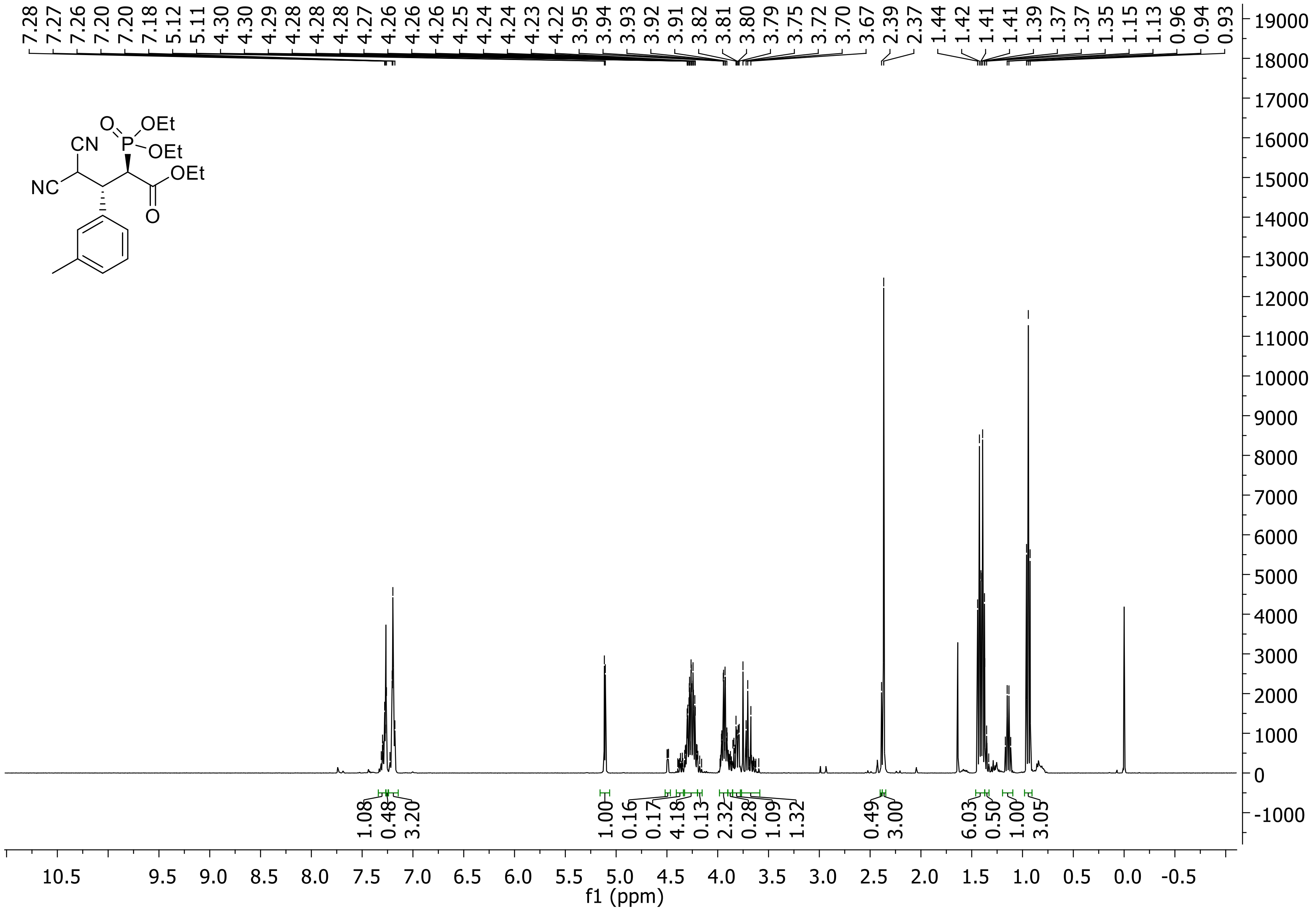

Figure S145. ^{1}H NMR spectrum of **3a** ($CDCl_3$, 400 MHz).

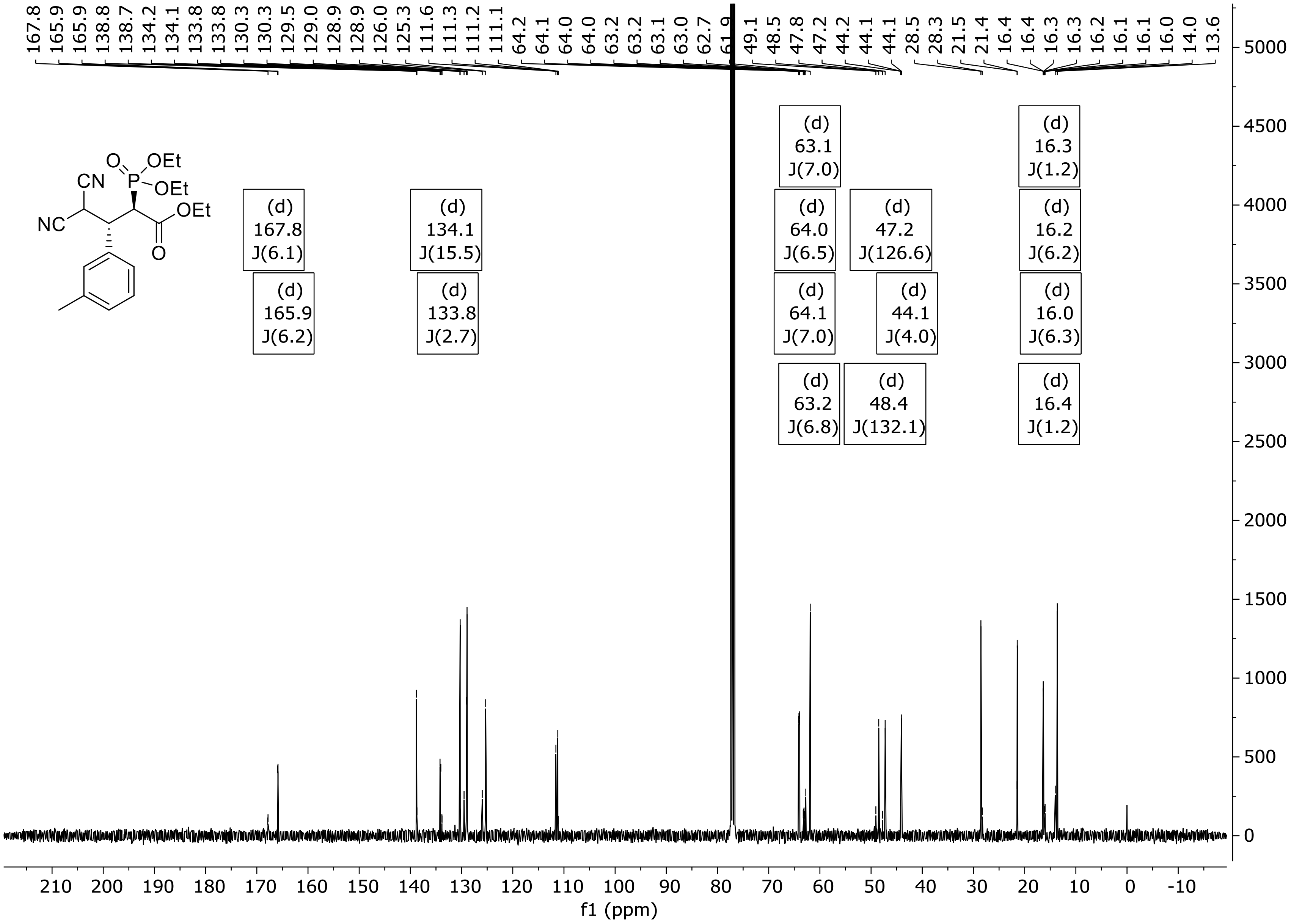

Figure S146. $^{13}C\{^{1}H\}$ NMR spectrum of **3a** ($CDCl_3$, 101 MHz).

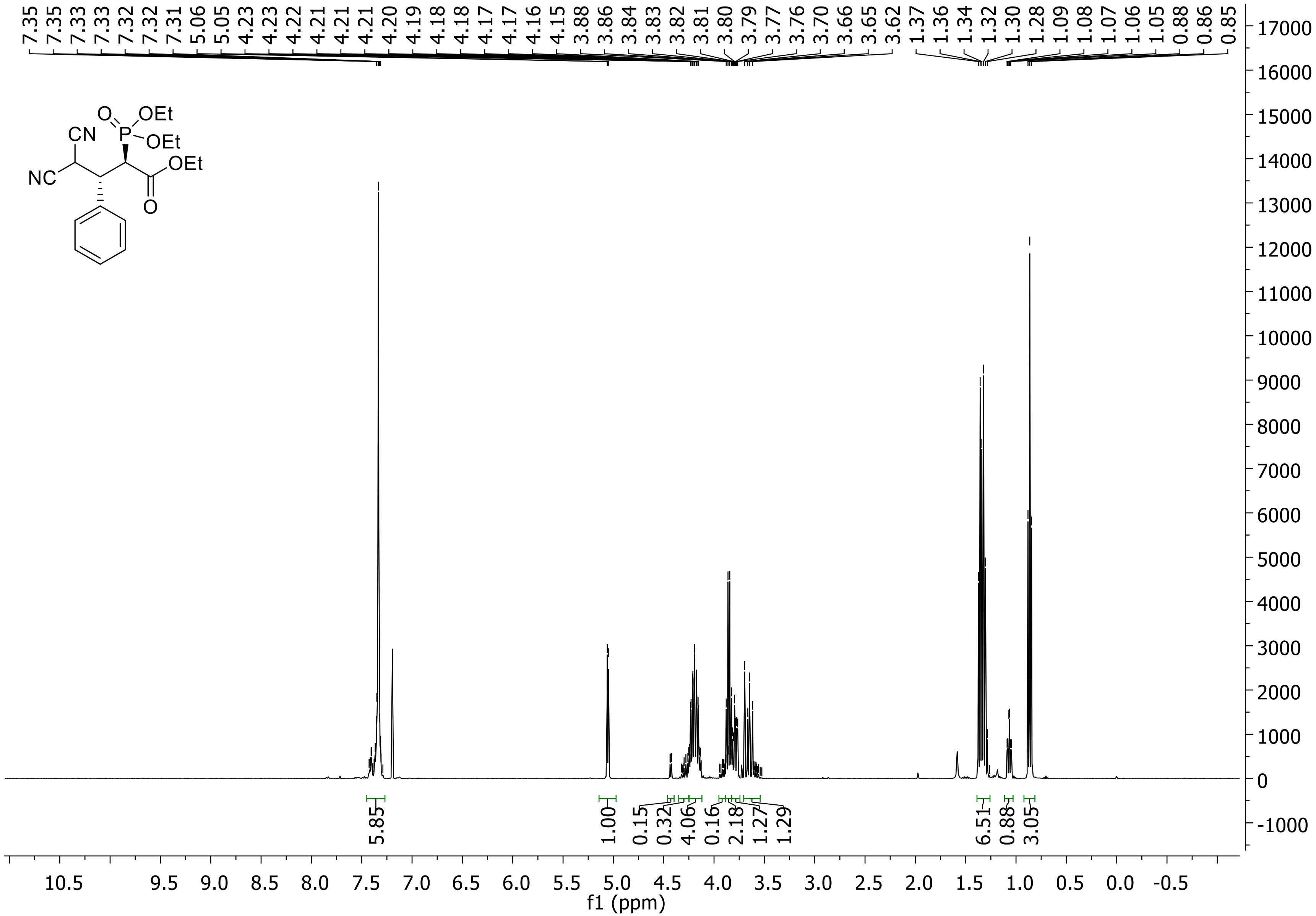

Figure S147. ^{1}H NMR spectrum of **3b** ($CDCl_3$, 400 MHz).

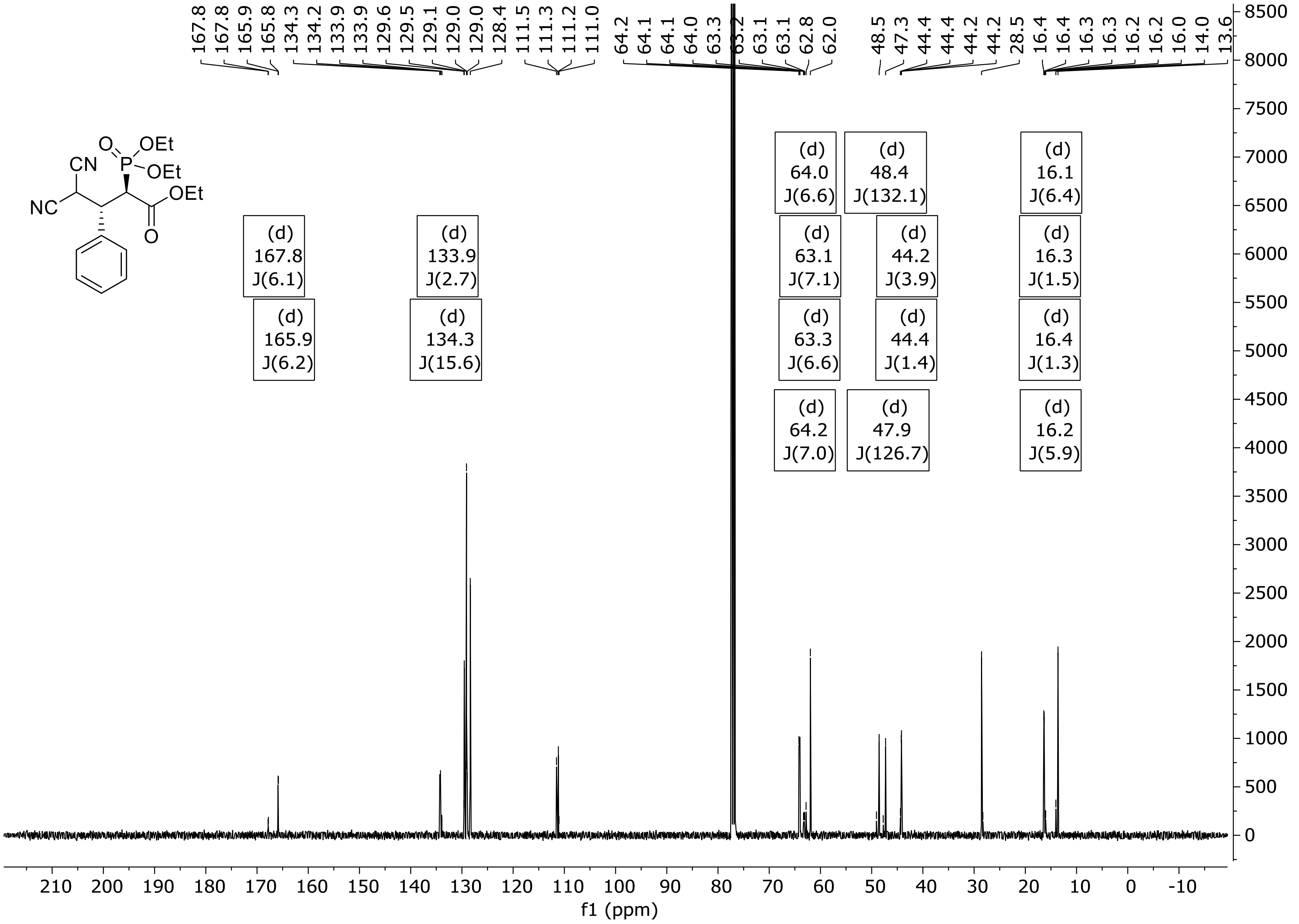

Figure S148. $^{13}C\{^{1}H\}$ NMR spectrum of **3b** ($CDCl_3$, 101 MHz).

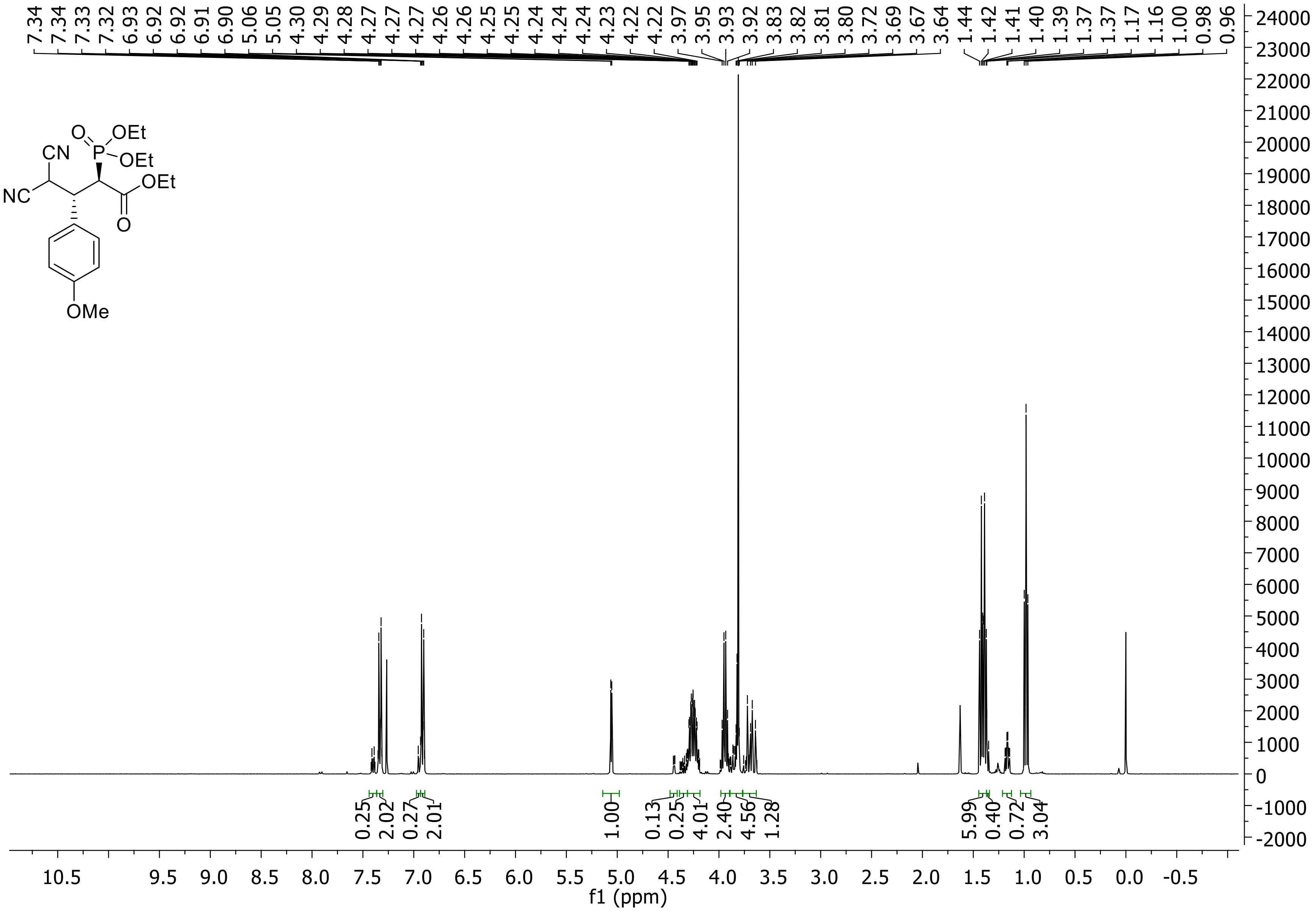

Figure S149. ^{1}H NMR spectrum of **3c** ($CDCl_3$, 400 MHz).

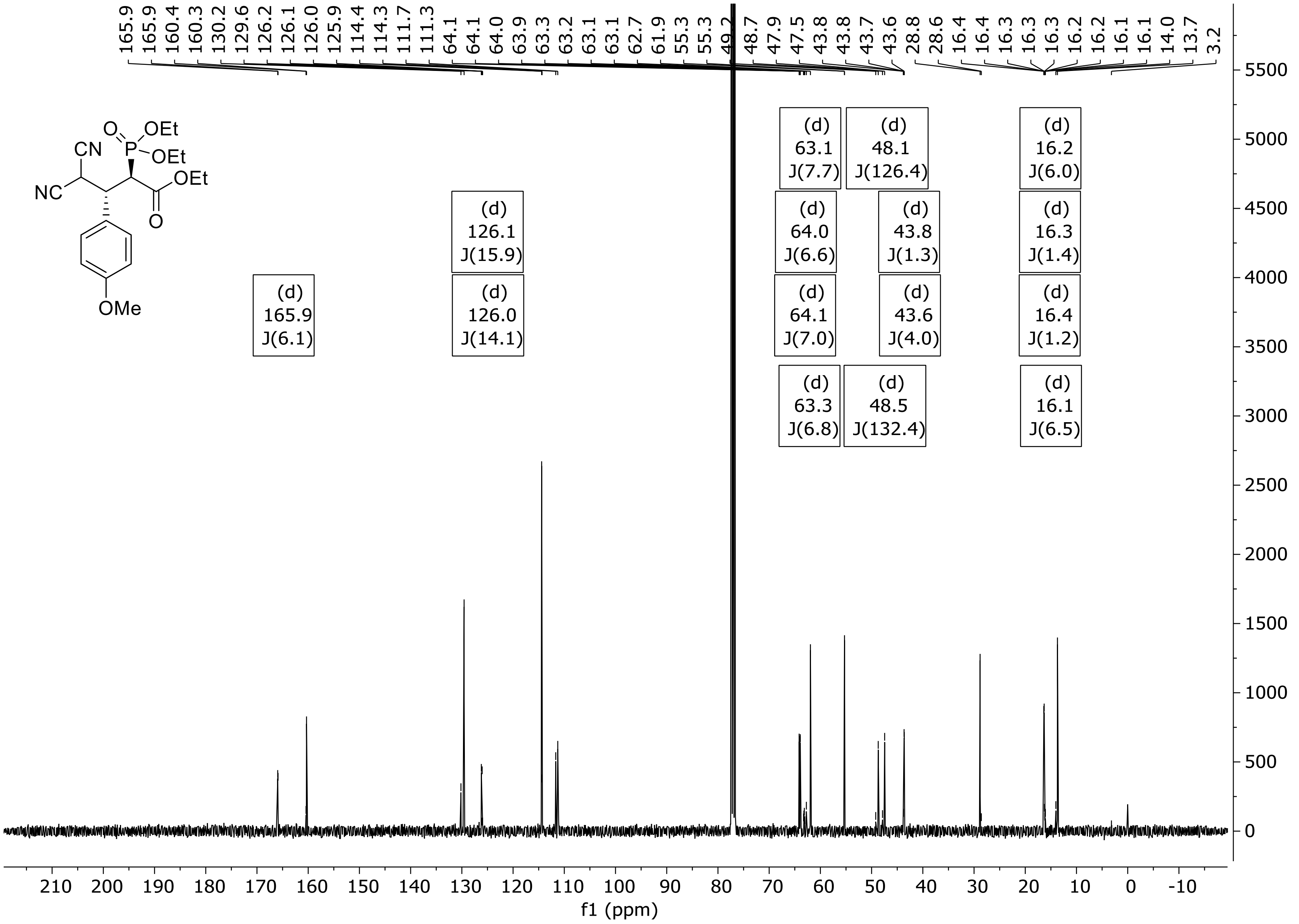

Figure S150. $^{13}C\{^{1}H\}$ NMR spectrum of **3c** ($CDCl_3$, 101 MHz).

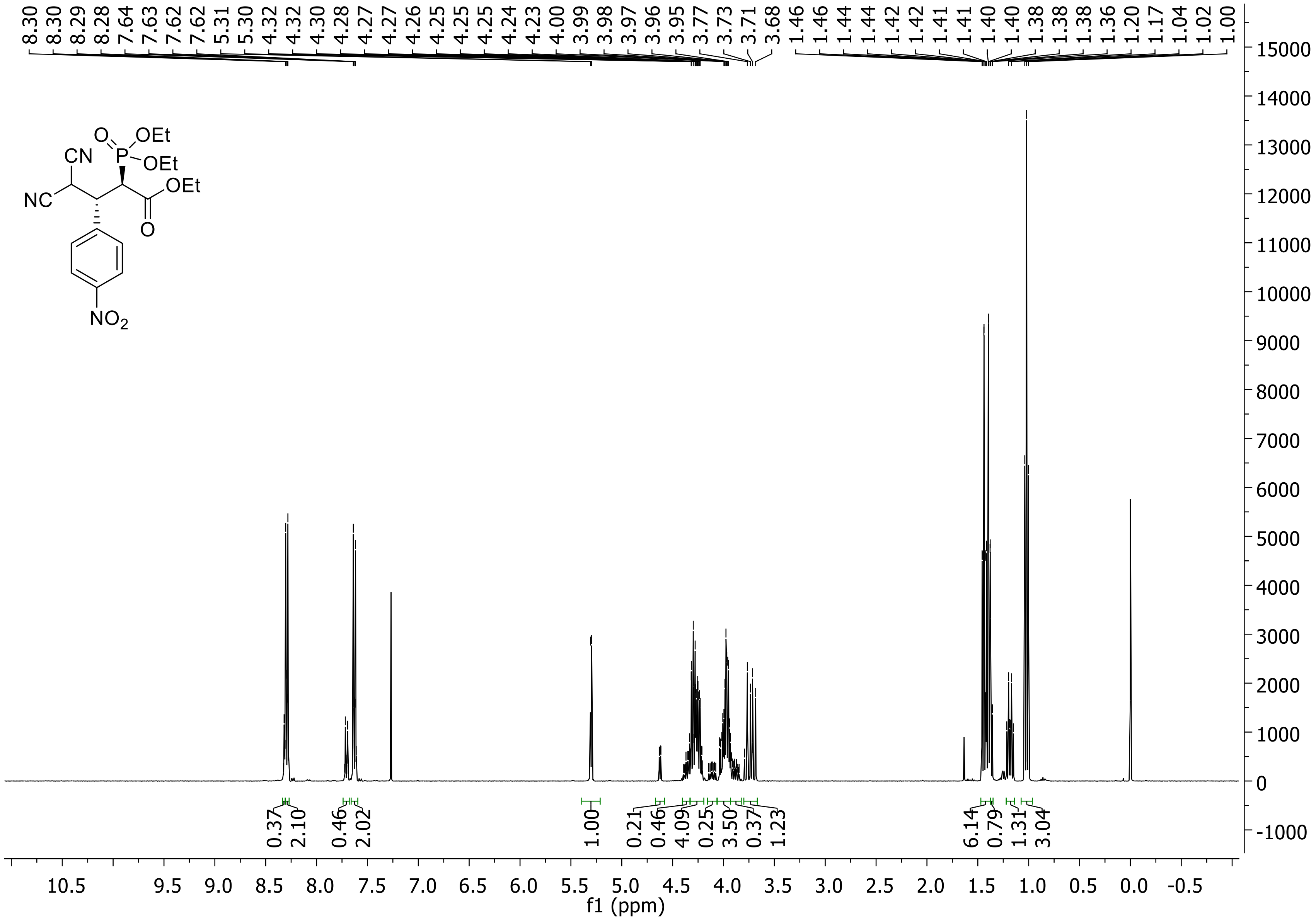

Figure S151. ^{1}H NMR spectrum of **3d** ($CDCl_3$, 400 MHz).

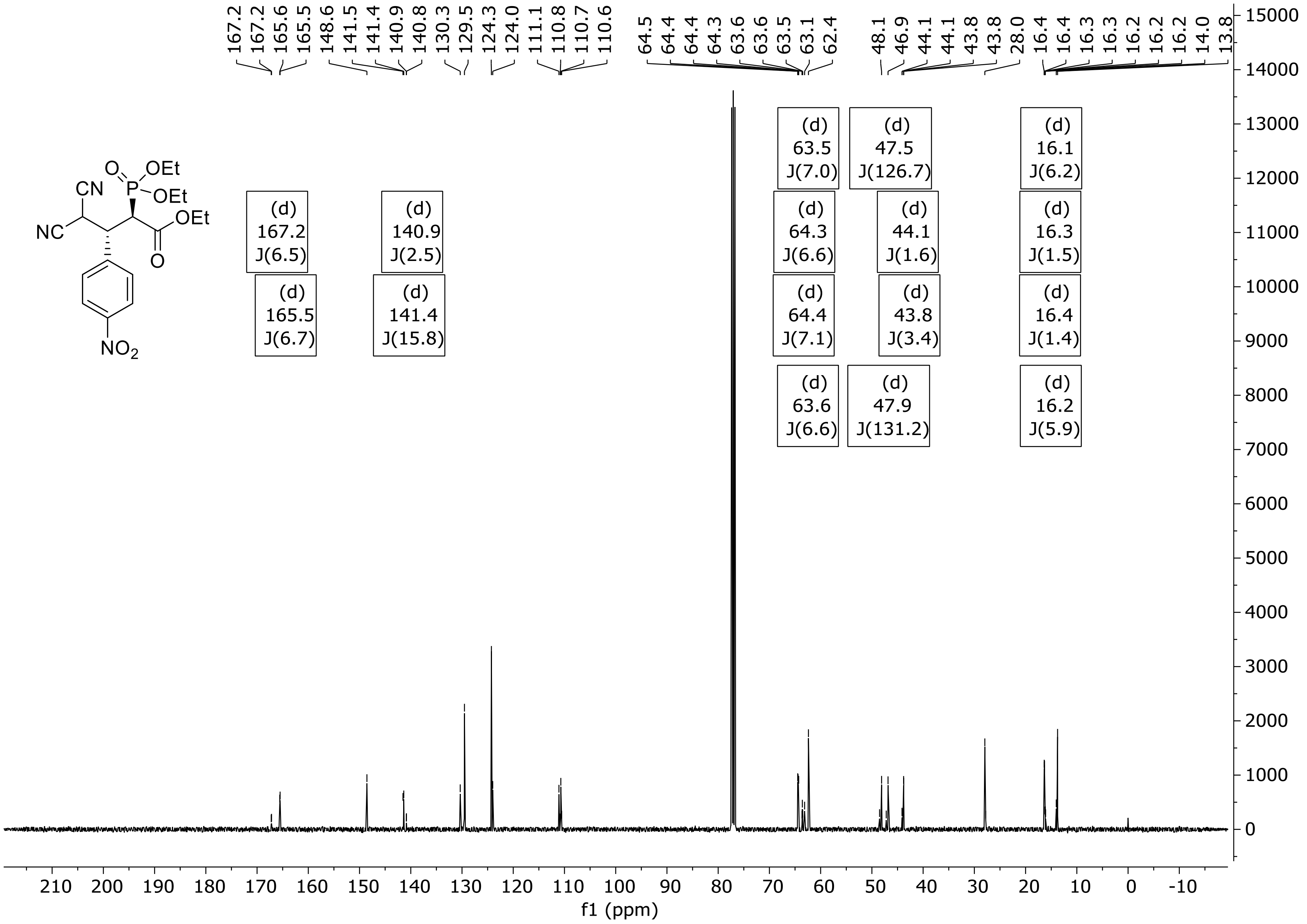

Figure S152. $^{13}C\{^{1}H\}$ NMR spectrum of **3d** ($CDCl_3$, 101 MHz).

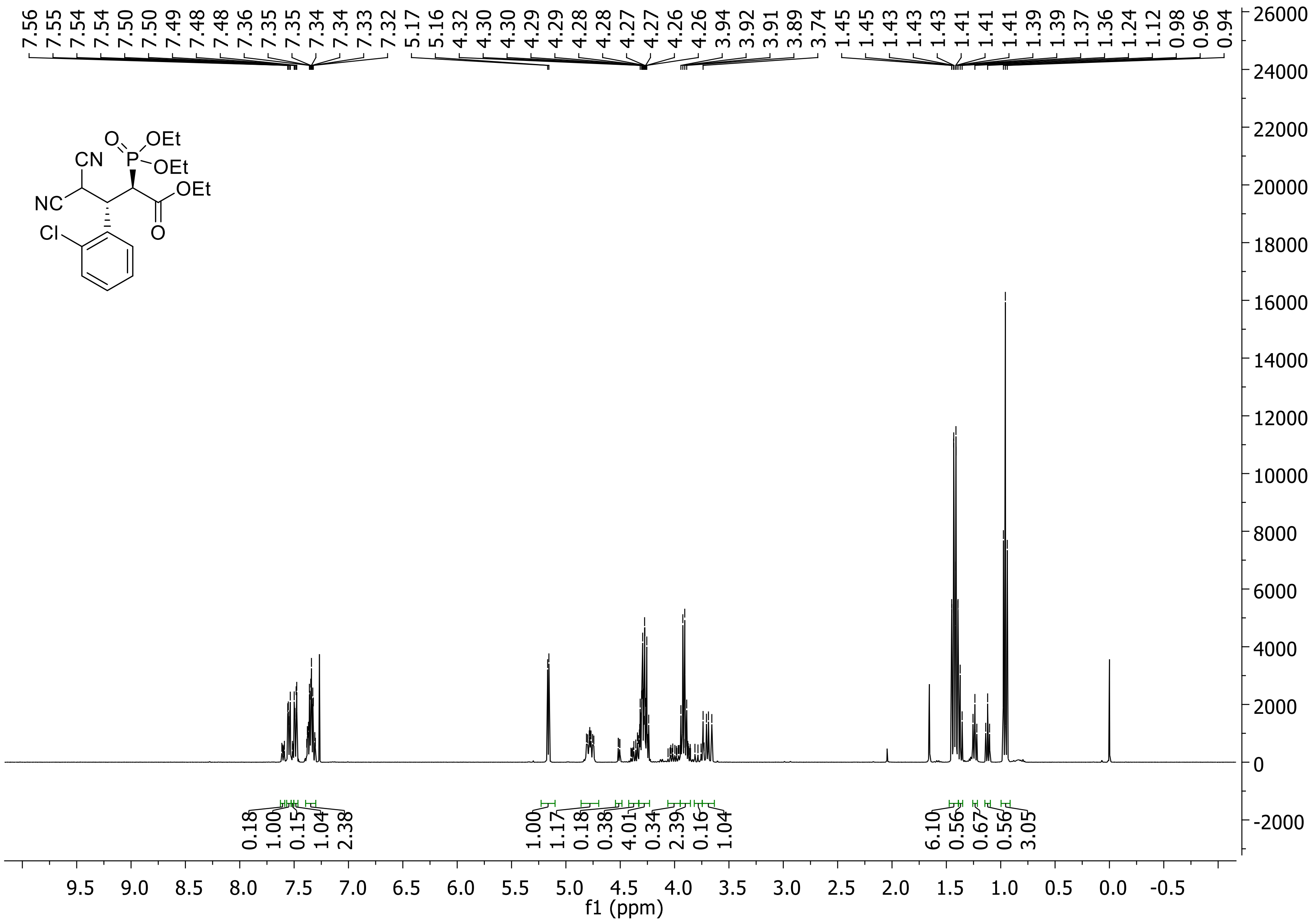

Figure S153. ^{1}H NMR spectrum of **3e** ($CDCl_3$, 400 MHz).

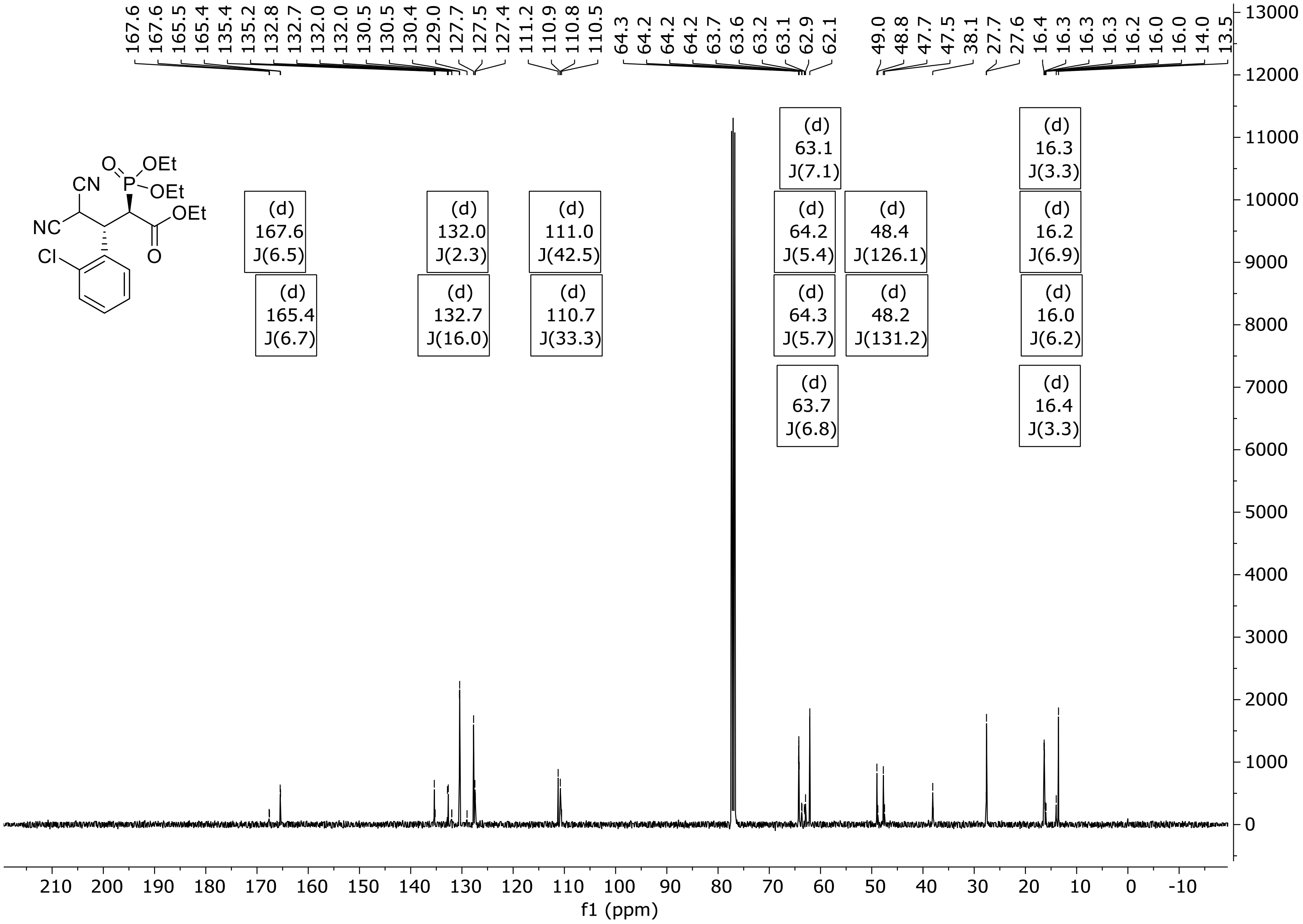

Figure S154. $^{13}C\{^1H\}$ NMR spectrum of **3e** ($CDCl_3$, 101 MHz).

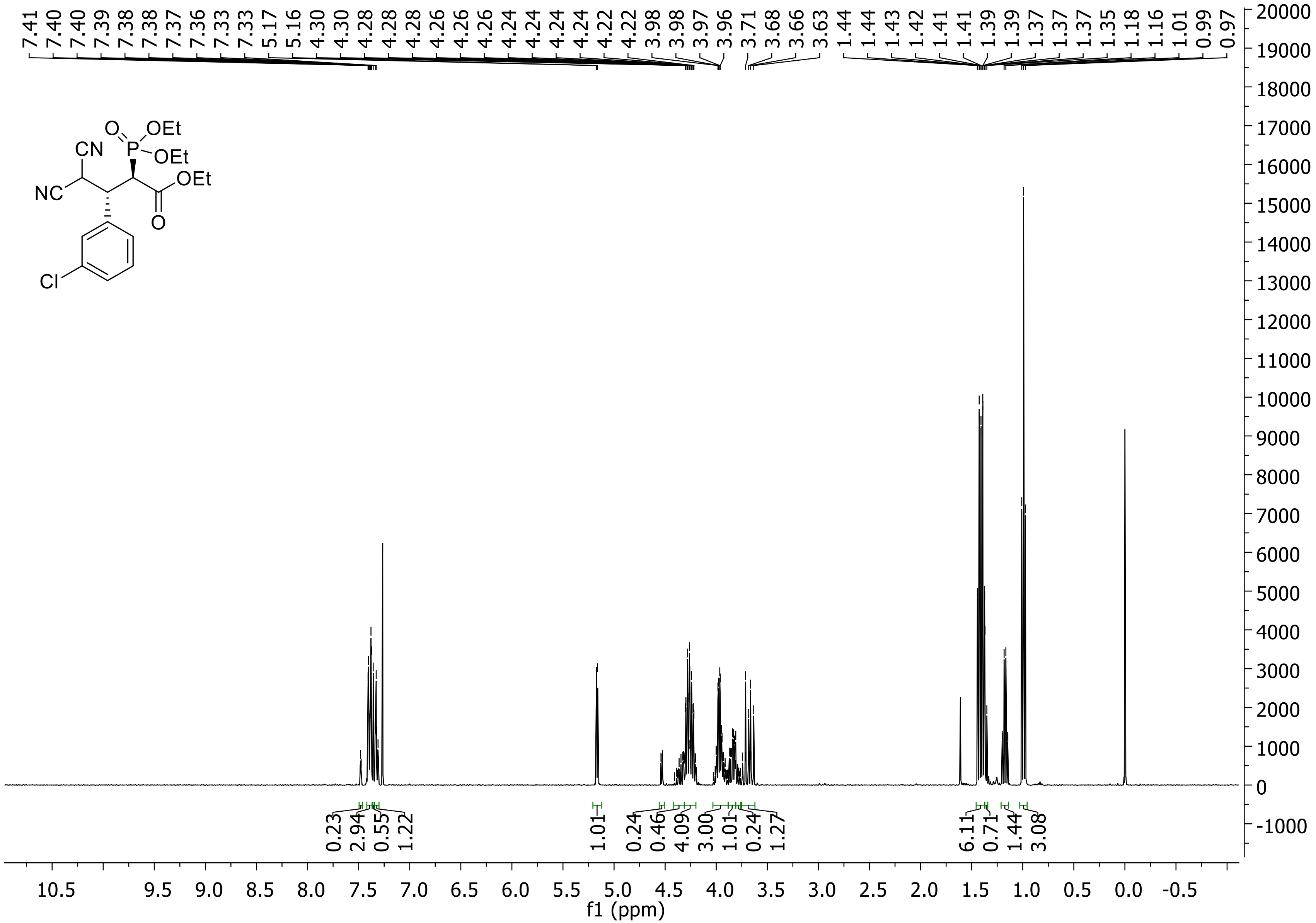

Figure S155. ^{1}H NMR spectrum of **3f** ($CDCl_3$, 400 MHz).

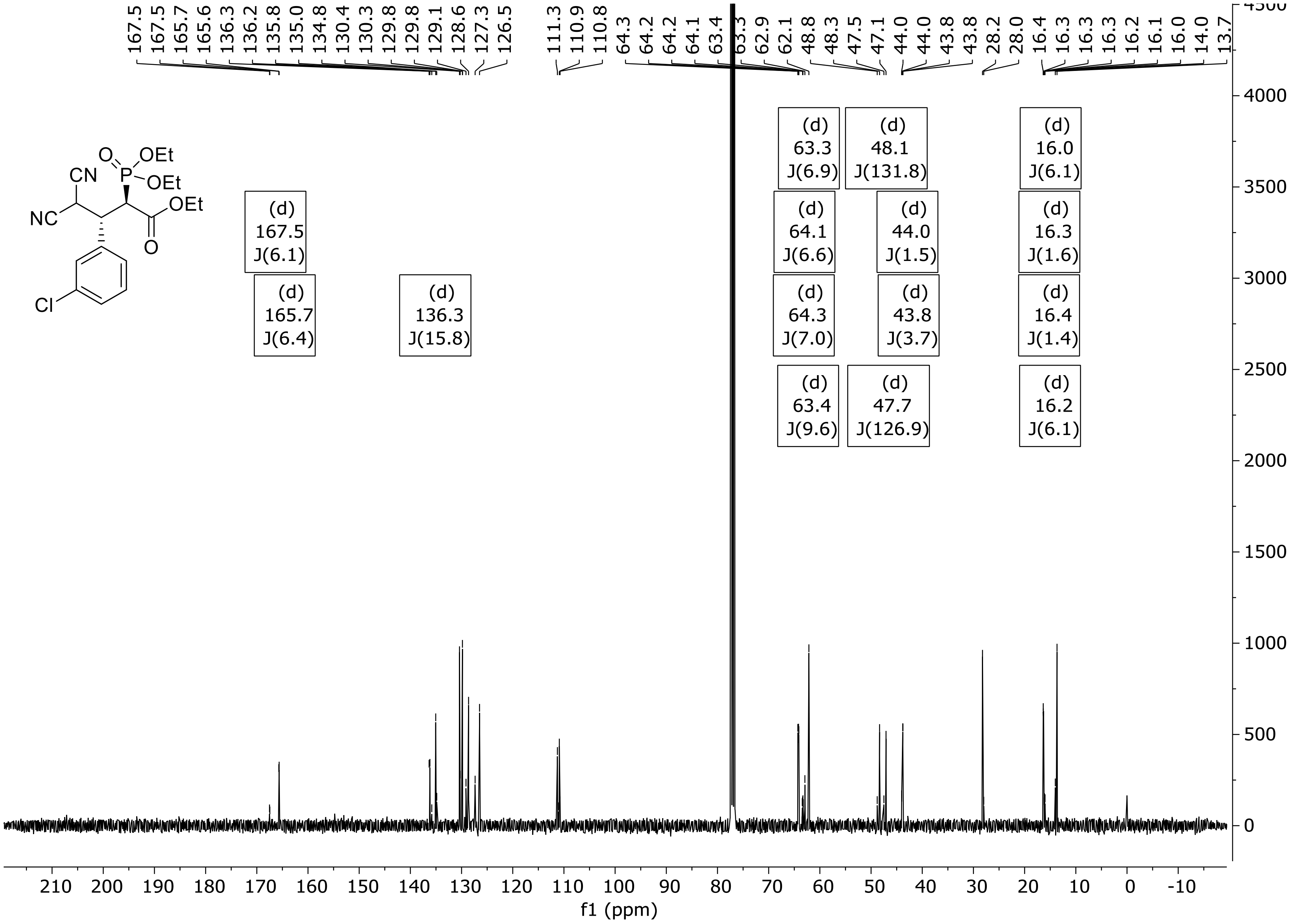

Figure S156. $^{13}C\{^{1}H\}$ NMR spectrum of **3f** ($CDCl_3$, 101 MHz).

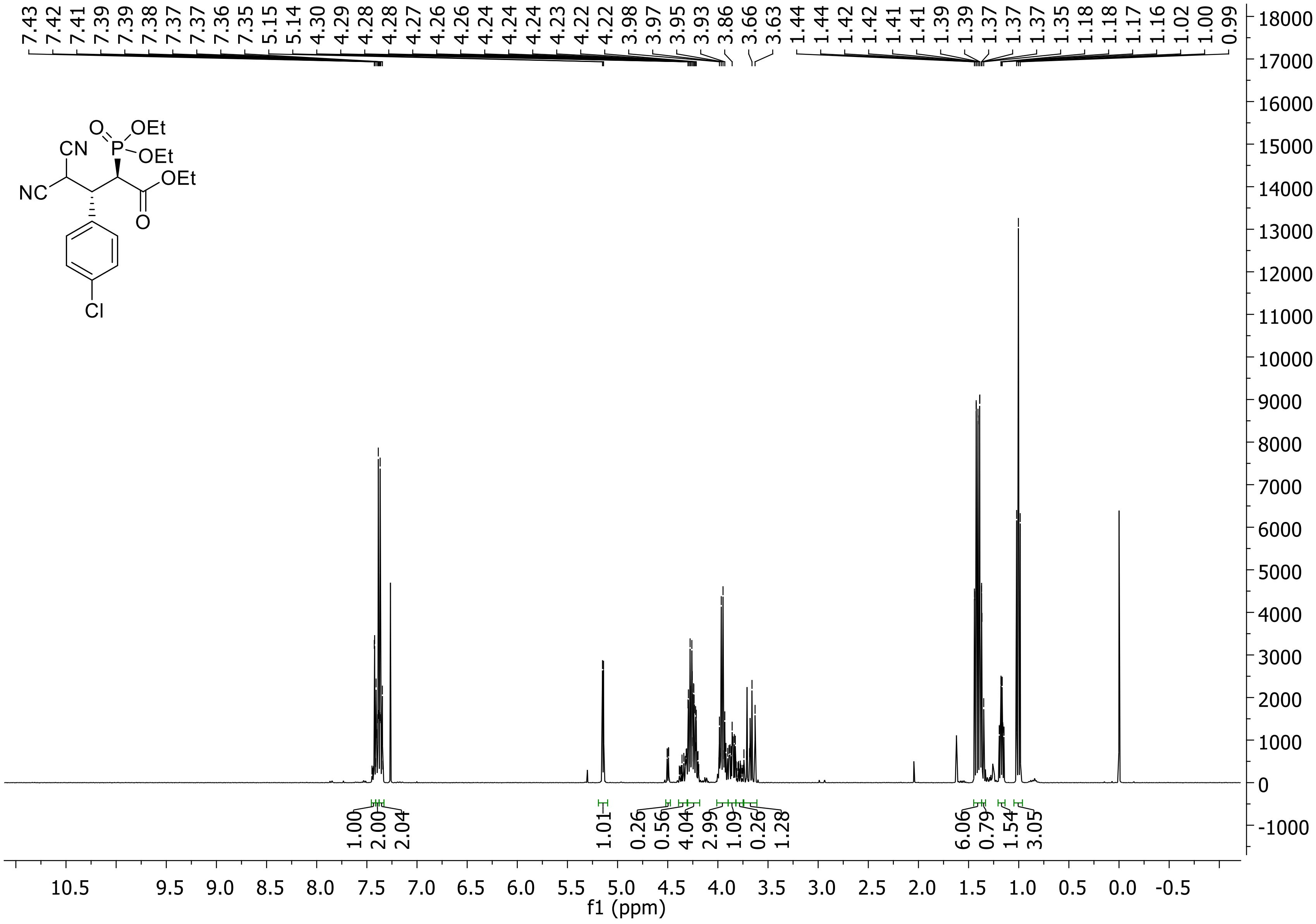

Figure S157. ^{1}H NMR spectrum of **3g** ($CDCl_3$, 400 MHz).

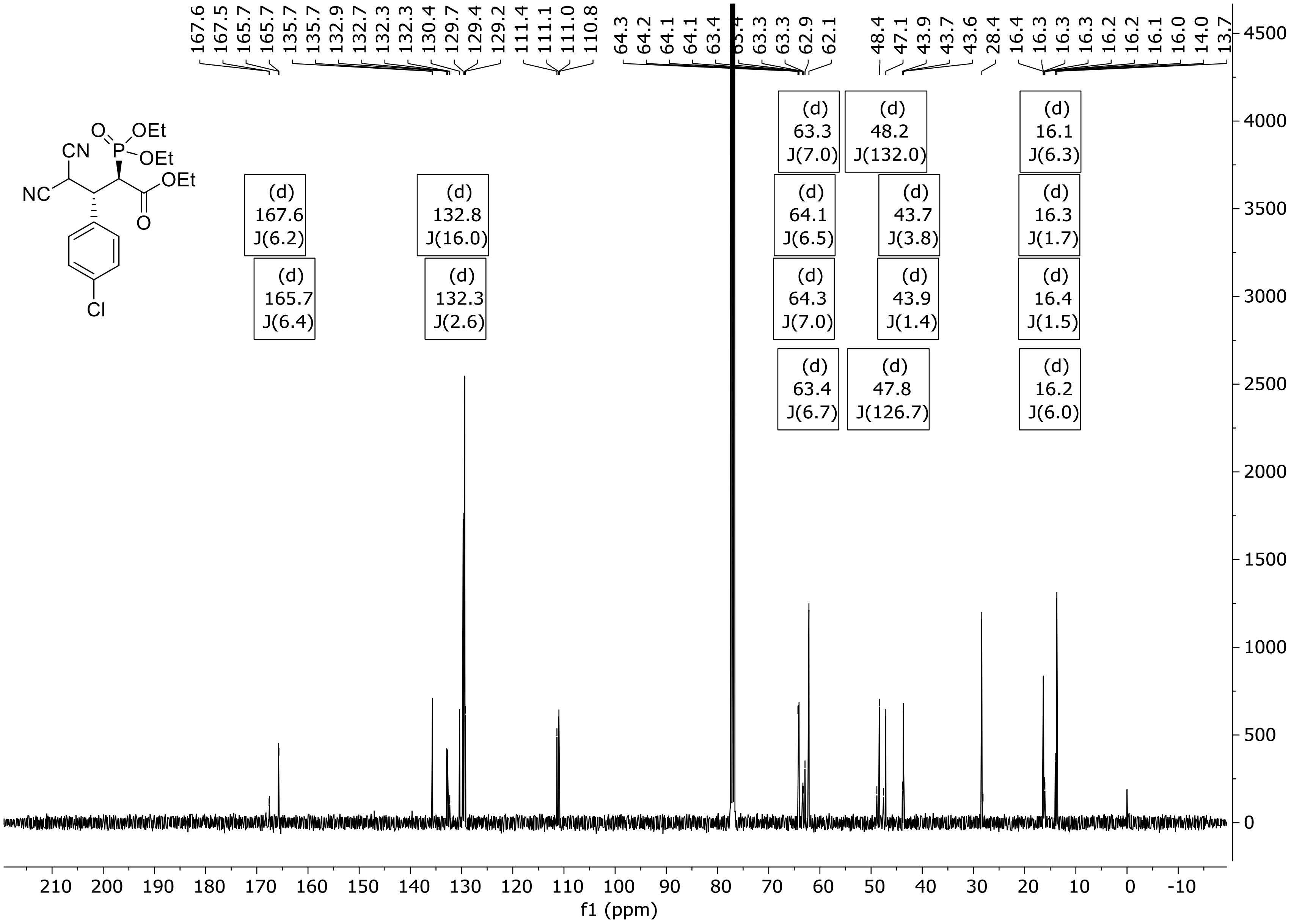

Figure S158. $^{13}C\{^{1}H\}$ NMR spectrum of **3g** ($CDCl_3$, 101 MHz).

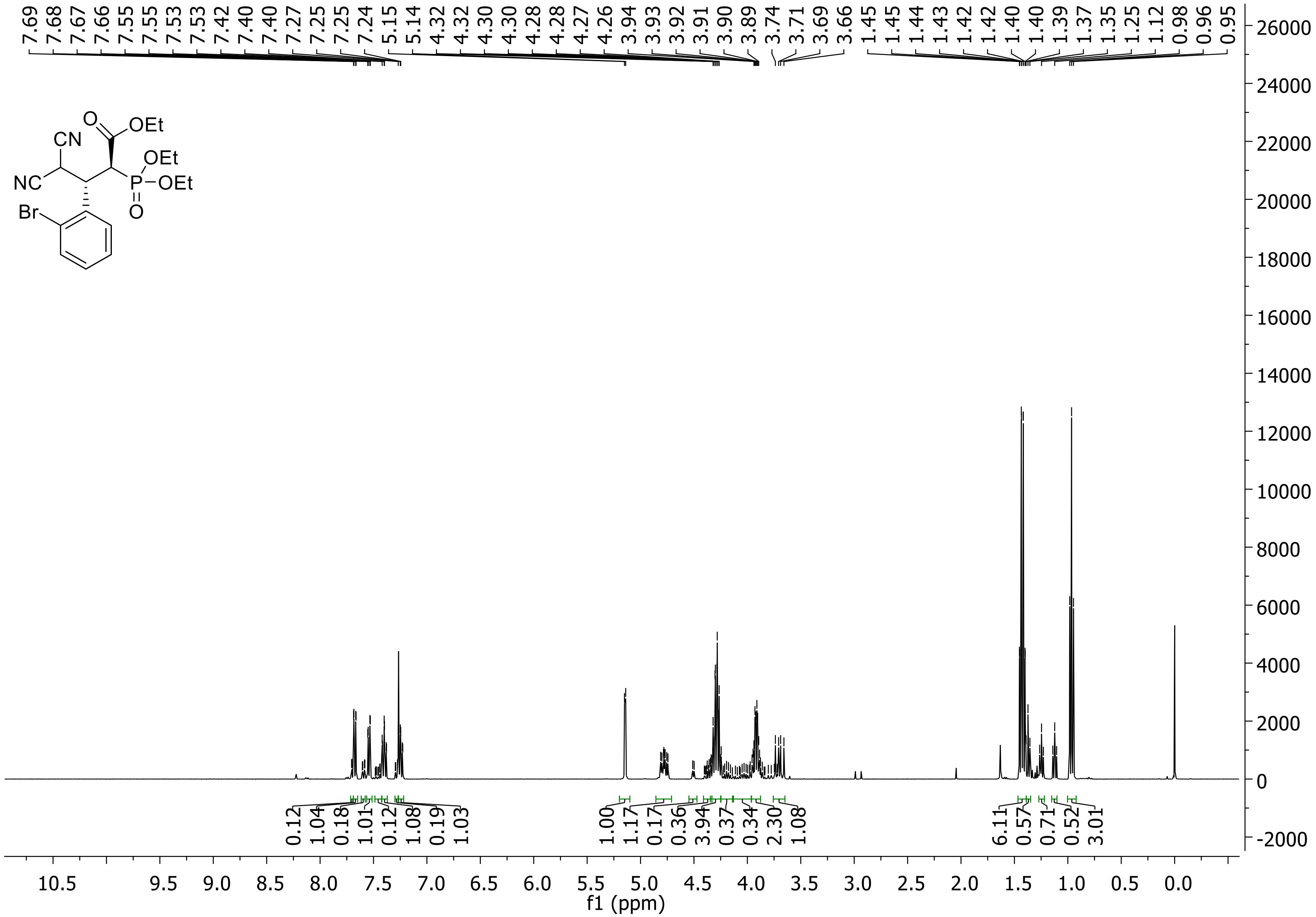

Figure S159. ^{1}H NMR spectrum of **3h** ($CDCl_3$, 400 MHz).

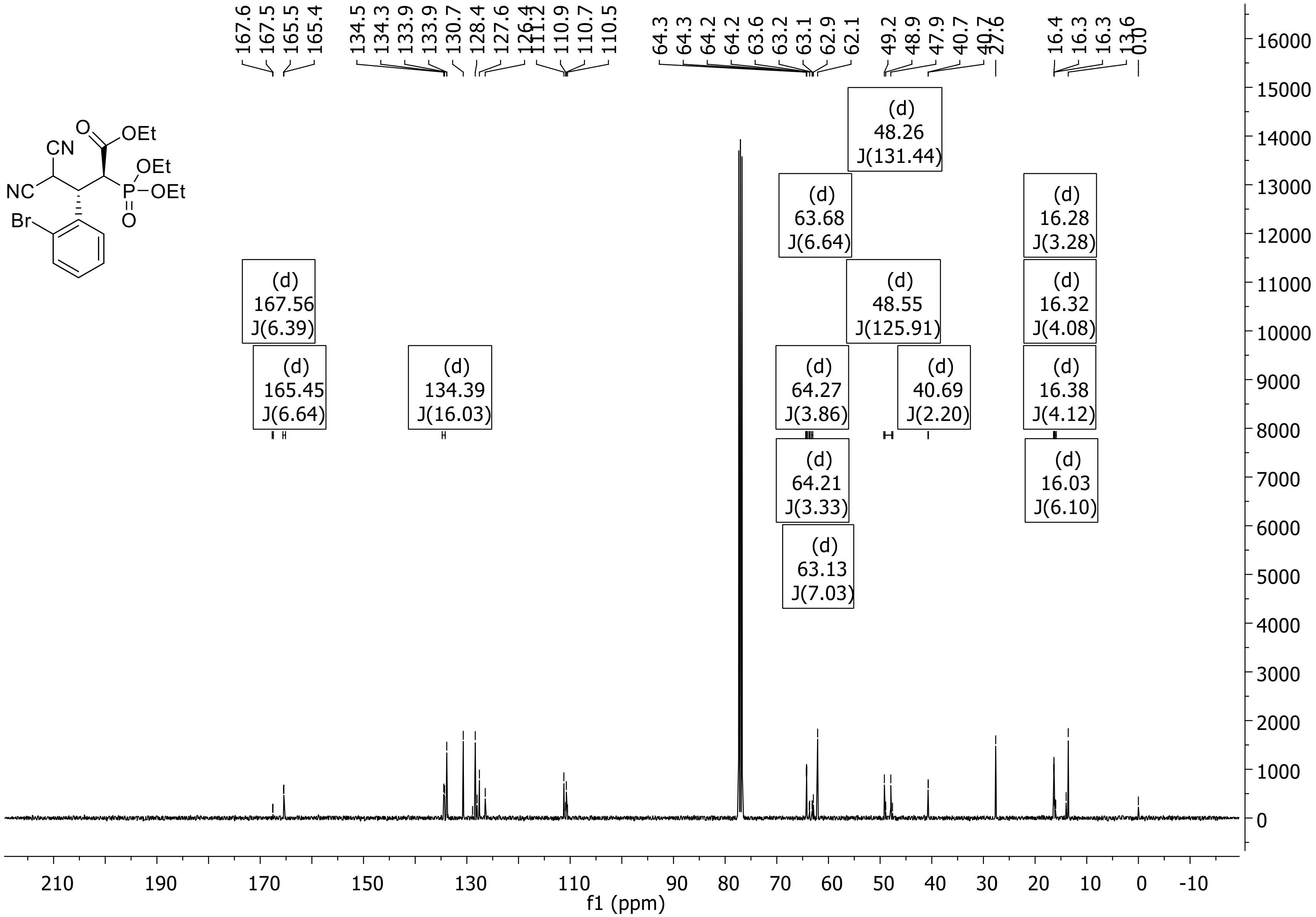

Figure S160. $^{13}C\{^{1}H\}$ NMR spectrum of **3h** ($CDCl_3$, 101 MHz).

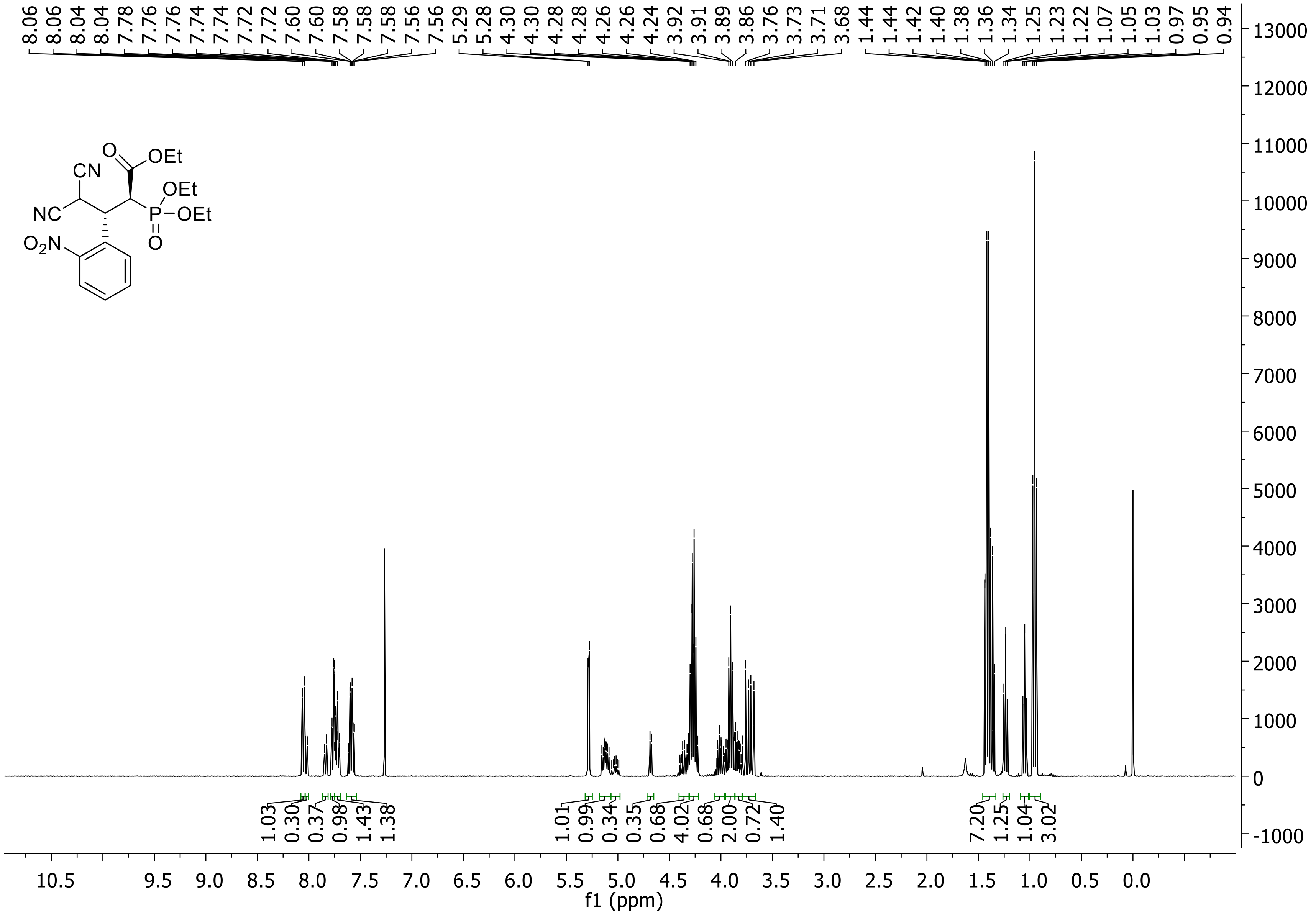

Figure S161. ^{1}H NMR spectrum of **3i** ($CDCl_3$, 400 MHz).

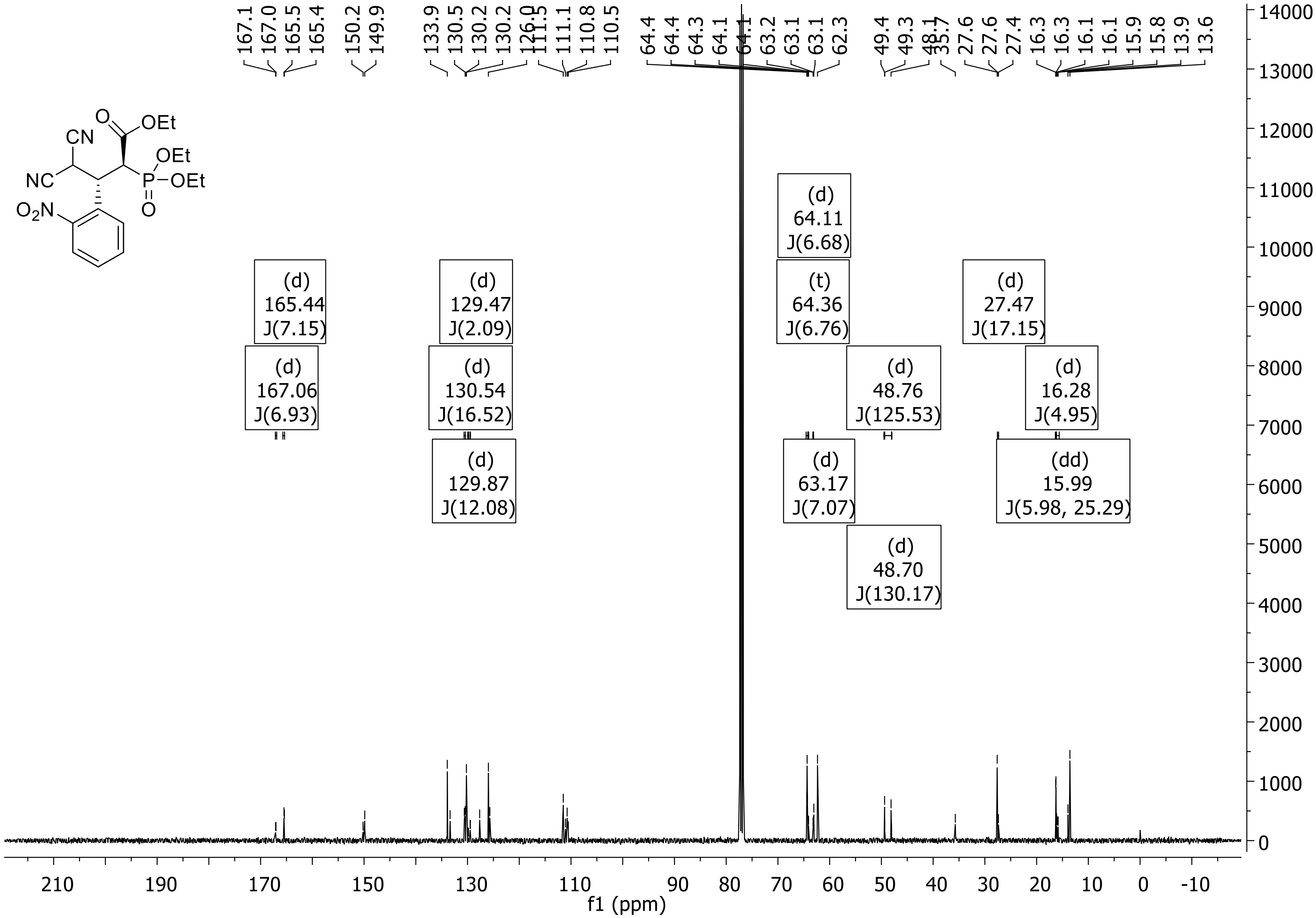

Figure S162. $^{13}C\{^1H\}$ NMR spectrum of **3i** ($CDCl_3$, 101 MHz).

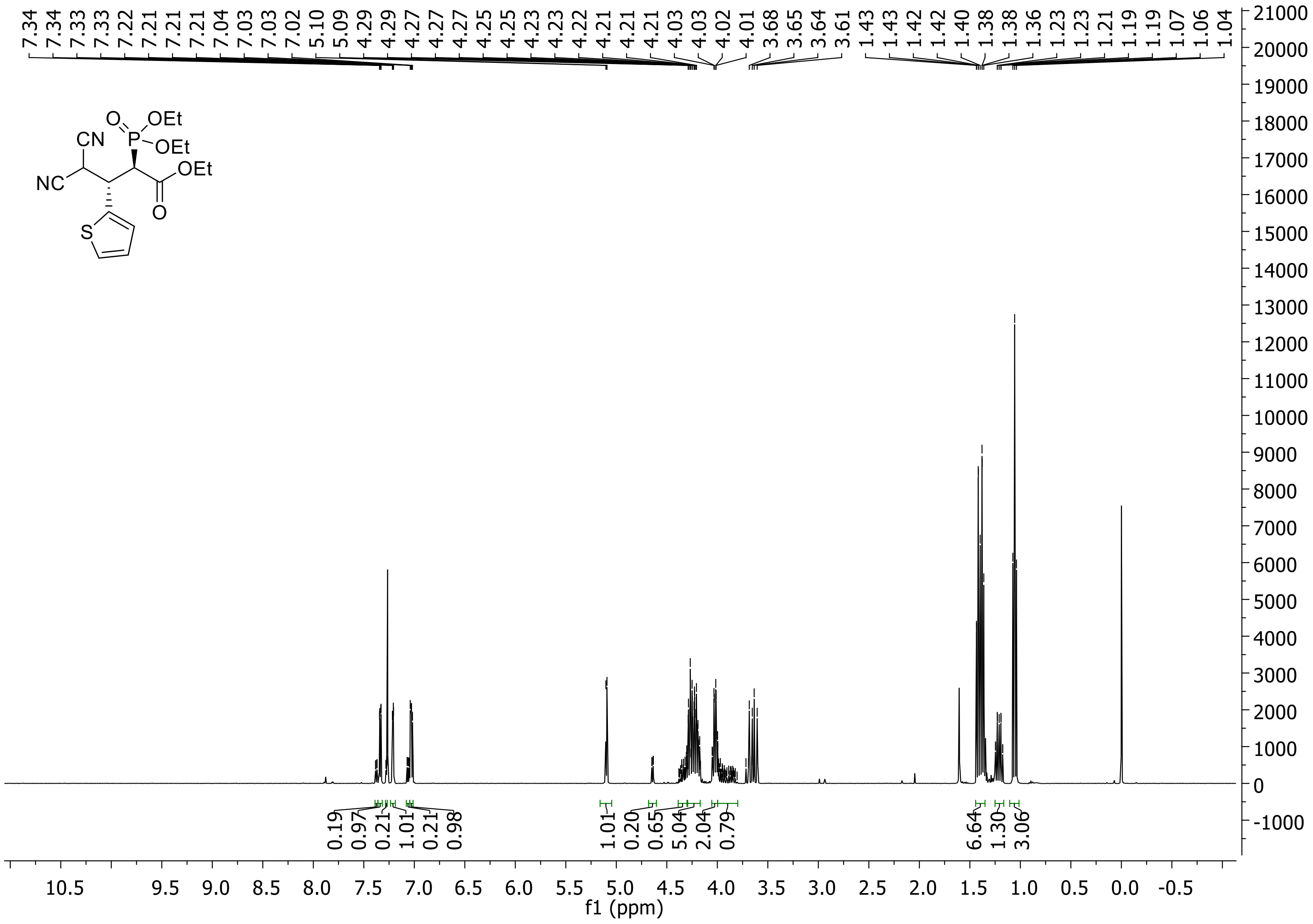

Figure S163. ^{1}H NMR spectrum of **3j** ($CDCl_3$, 400 MHz).

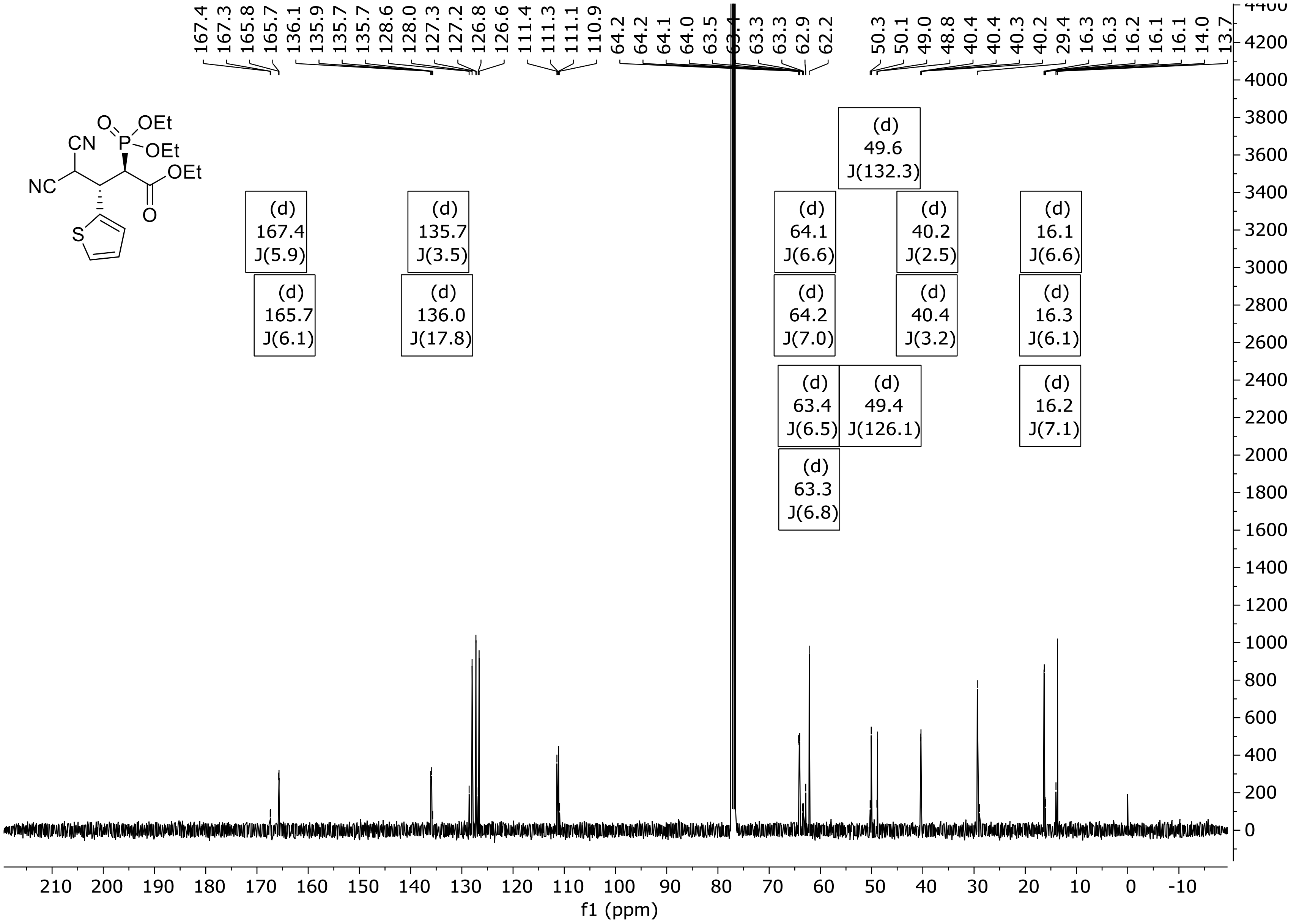

Figure S164. $^{13}C\{^{1}H\}$ NMR spectrum of **3j** ($CDCl_3$, 101 MHz).

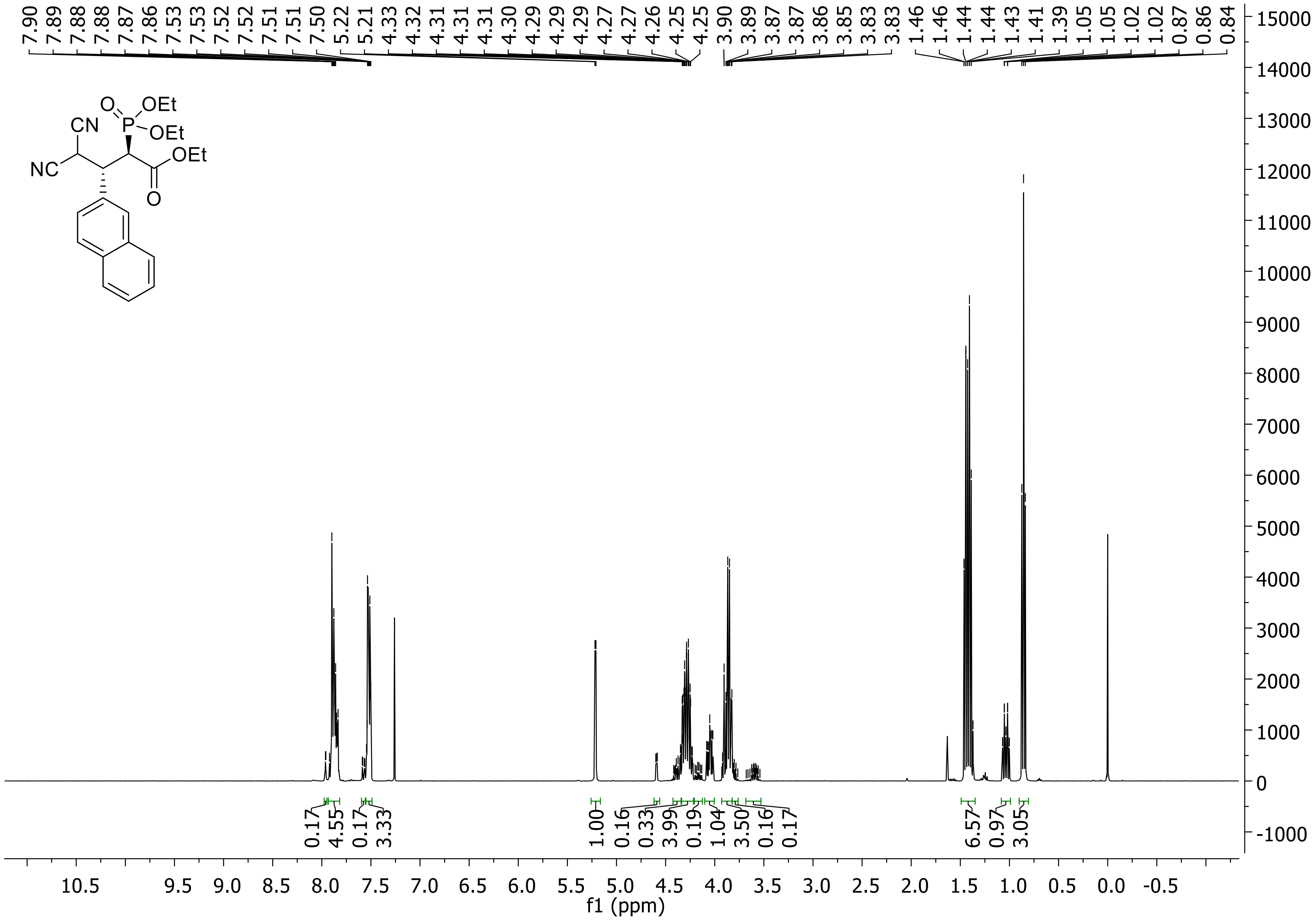

Figure S165. ^{1}H NMR spectrum of **3k** ($CDCl_3$, 400 MHz).

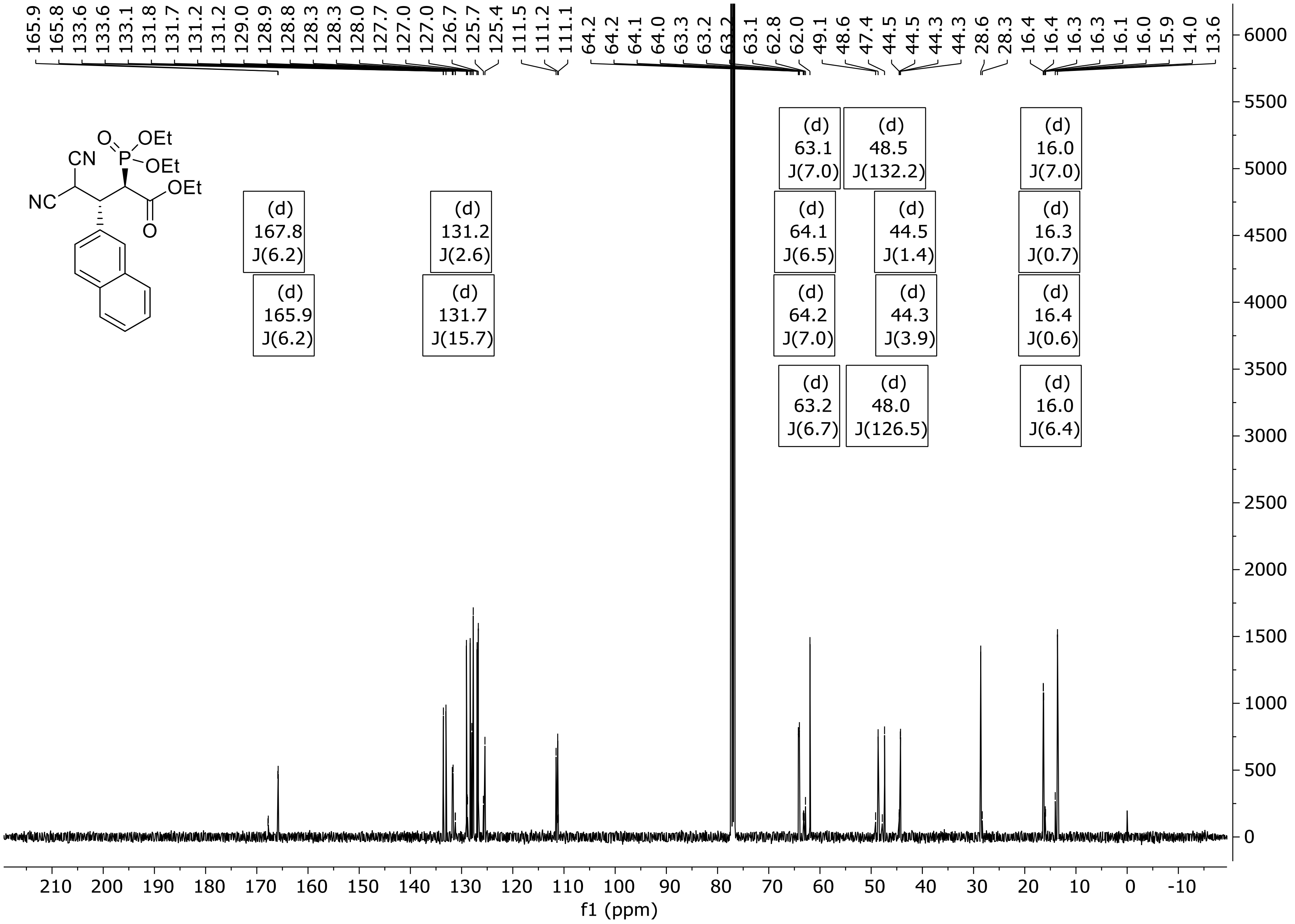

Figure S166. $^{13}C\{^{1}H\}$ NMR spectrum of **3k** ($CDCl_3$, 101 MHz).

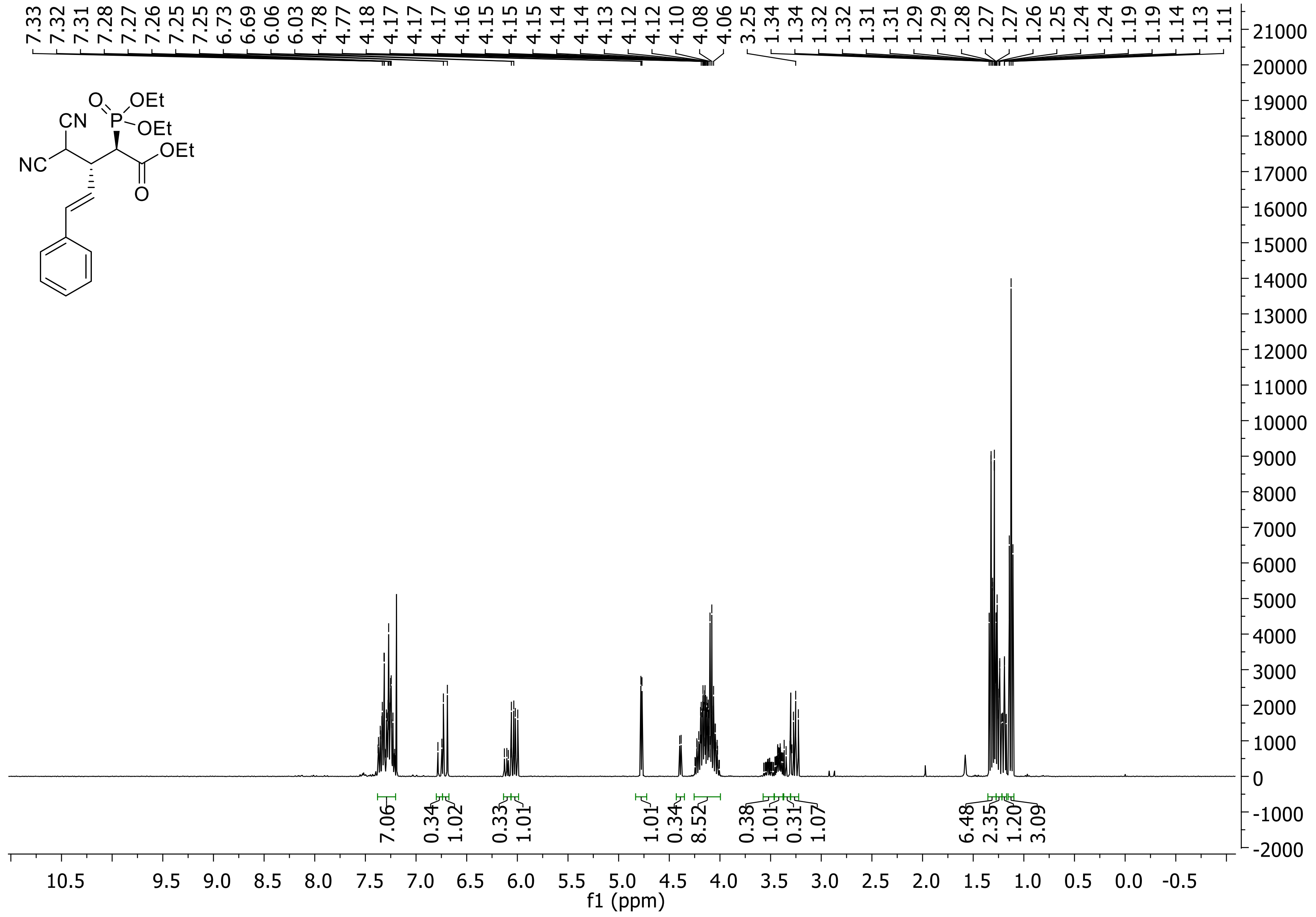

Figure S167. ^{1}H NMR spectrum of **3l** ($CDCl_3$, 400 MHz).

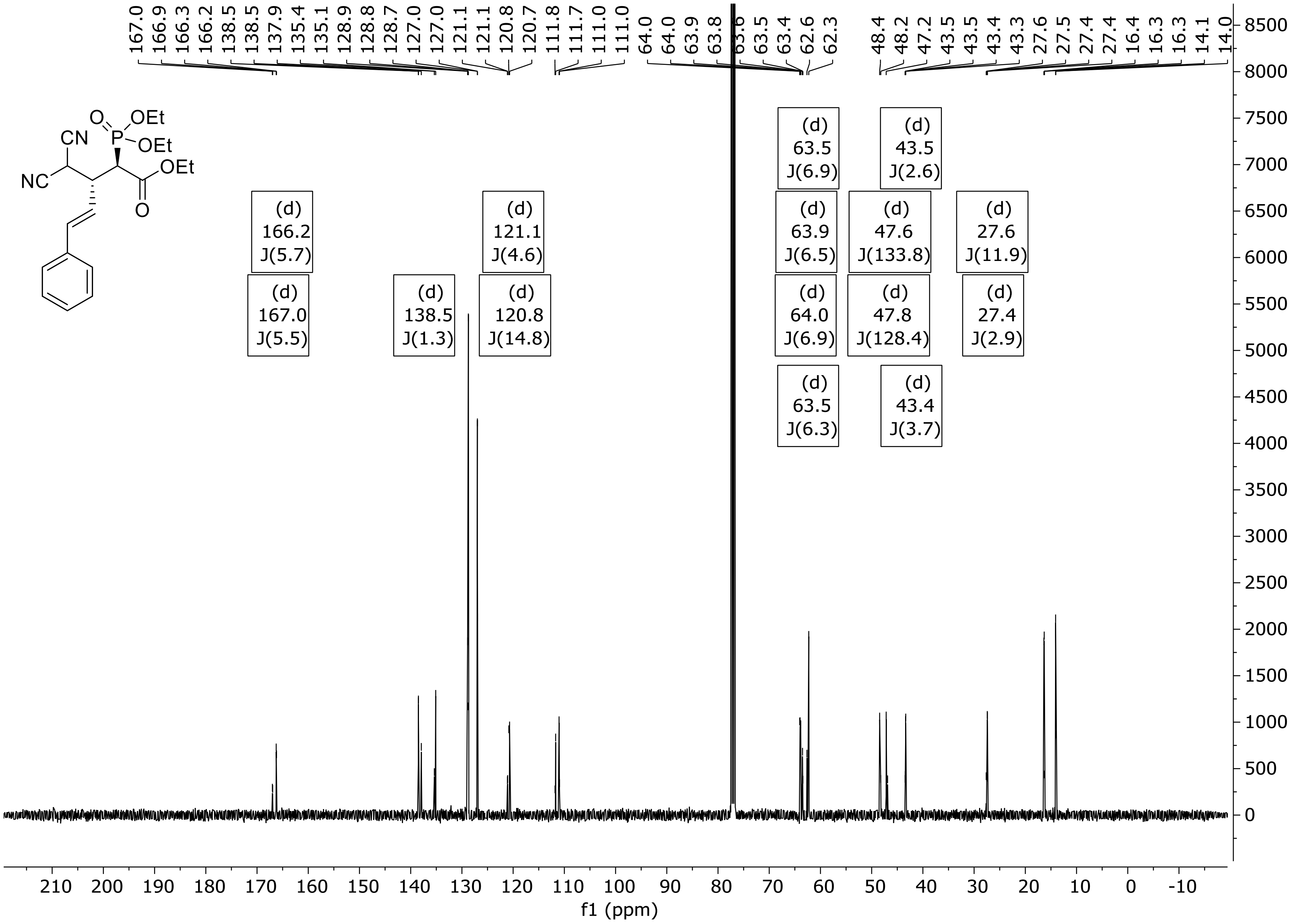

Figure S168. $^{13}C\{^1H\}$ NMR spectrum of **3l** ($CDCl_3$, 101 MHz).

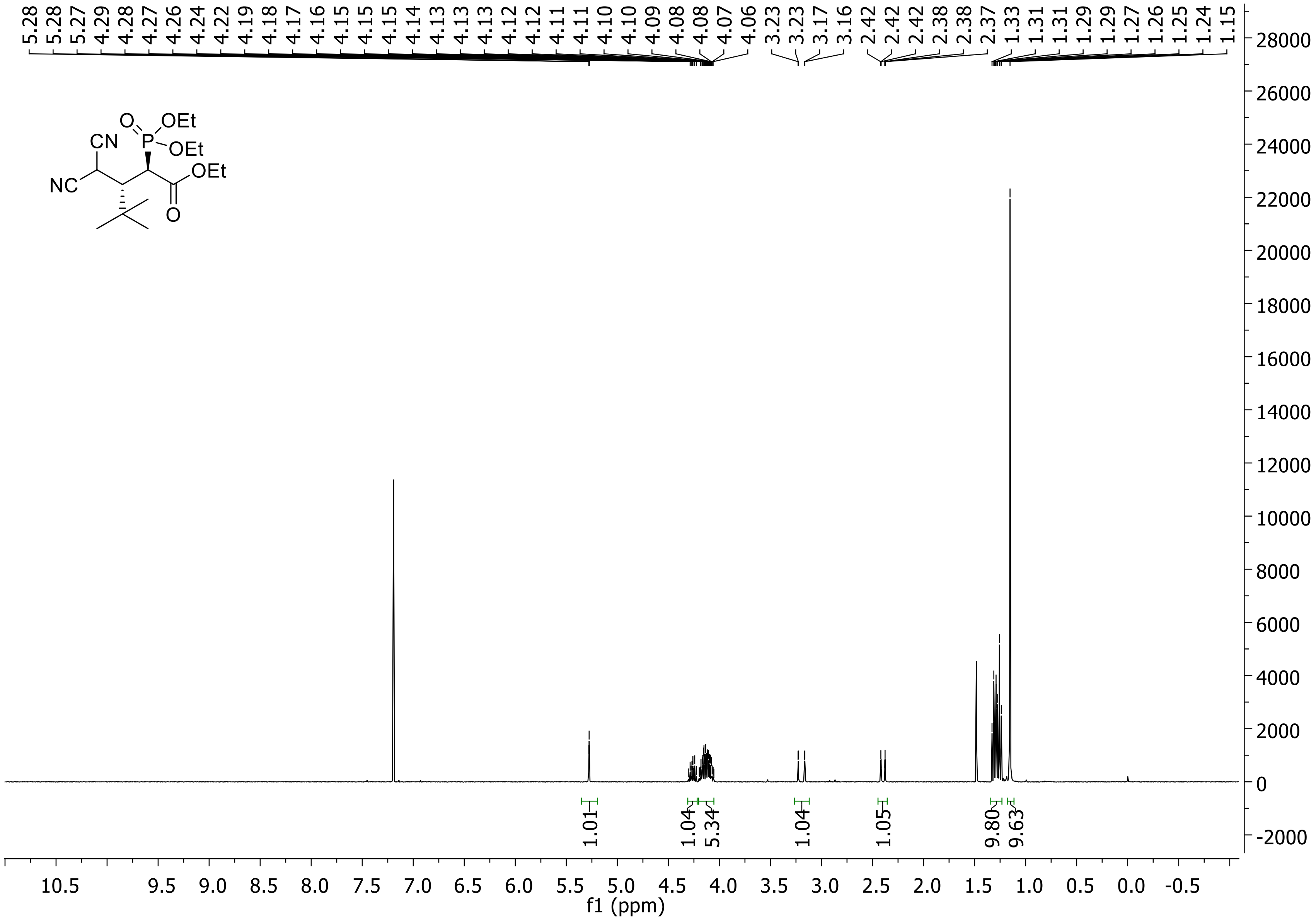

Figure S169. ^{1}H NMR spectrum of **3m** ($CDCl_3$, 400 MHz).

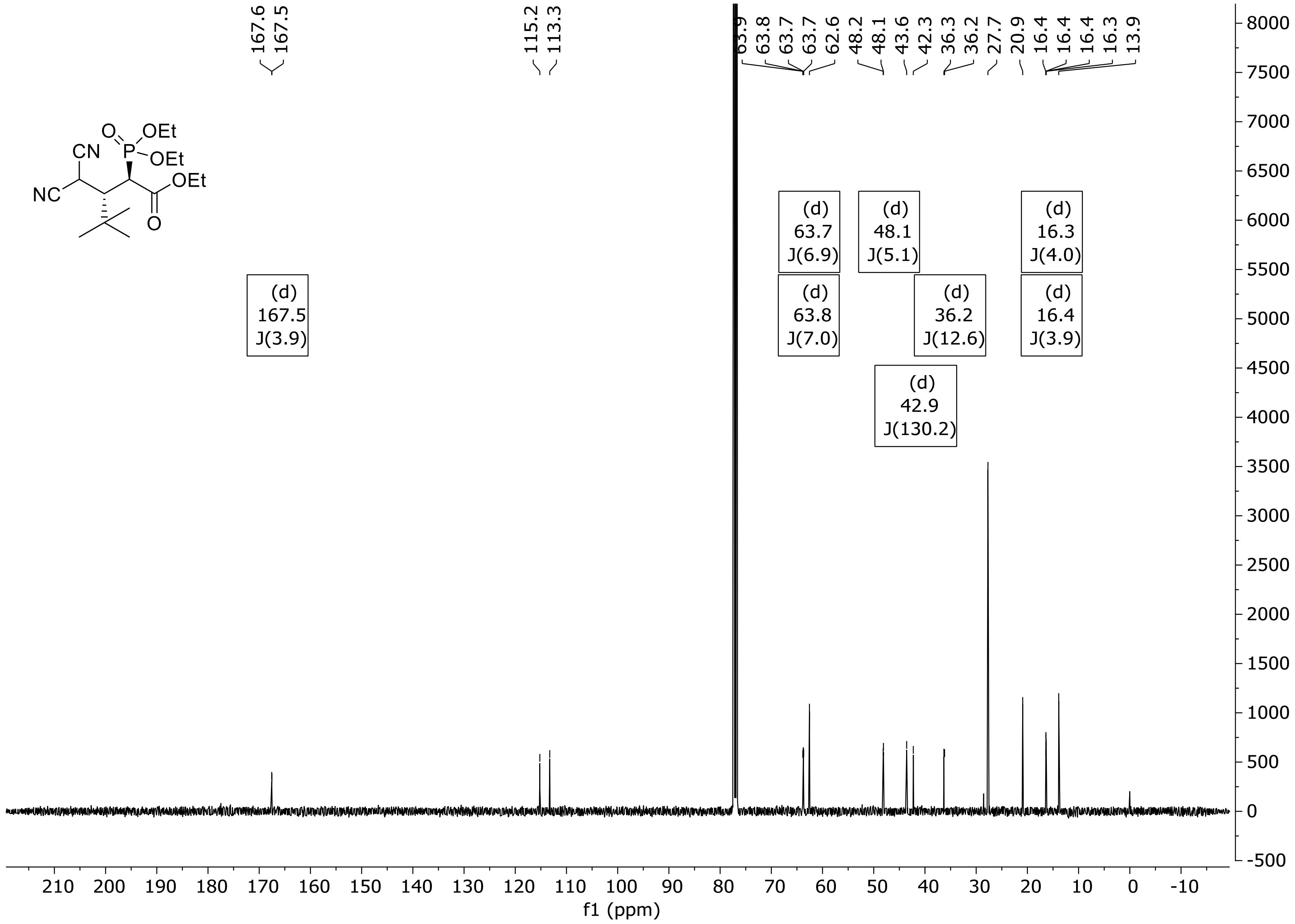

Figure S170. $^{13}C\{^{1}H\}$ NMR spectrum of **3m** ($CDCl_3$, 101 MHz).

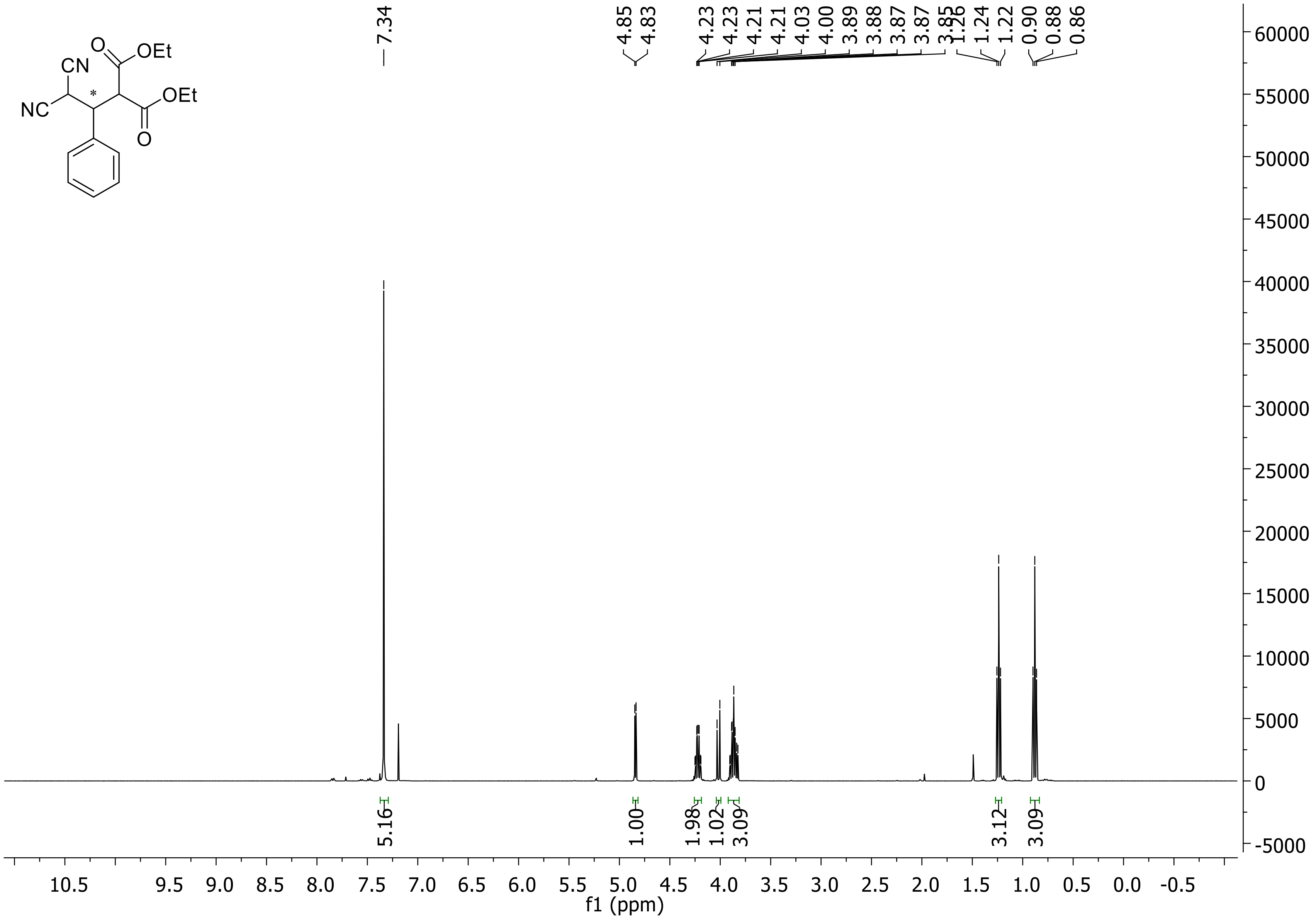

Figure S171. ^{1}H NMR spectrum of **7** ($CDCl_3$, 400 MHz).

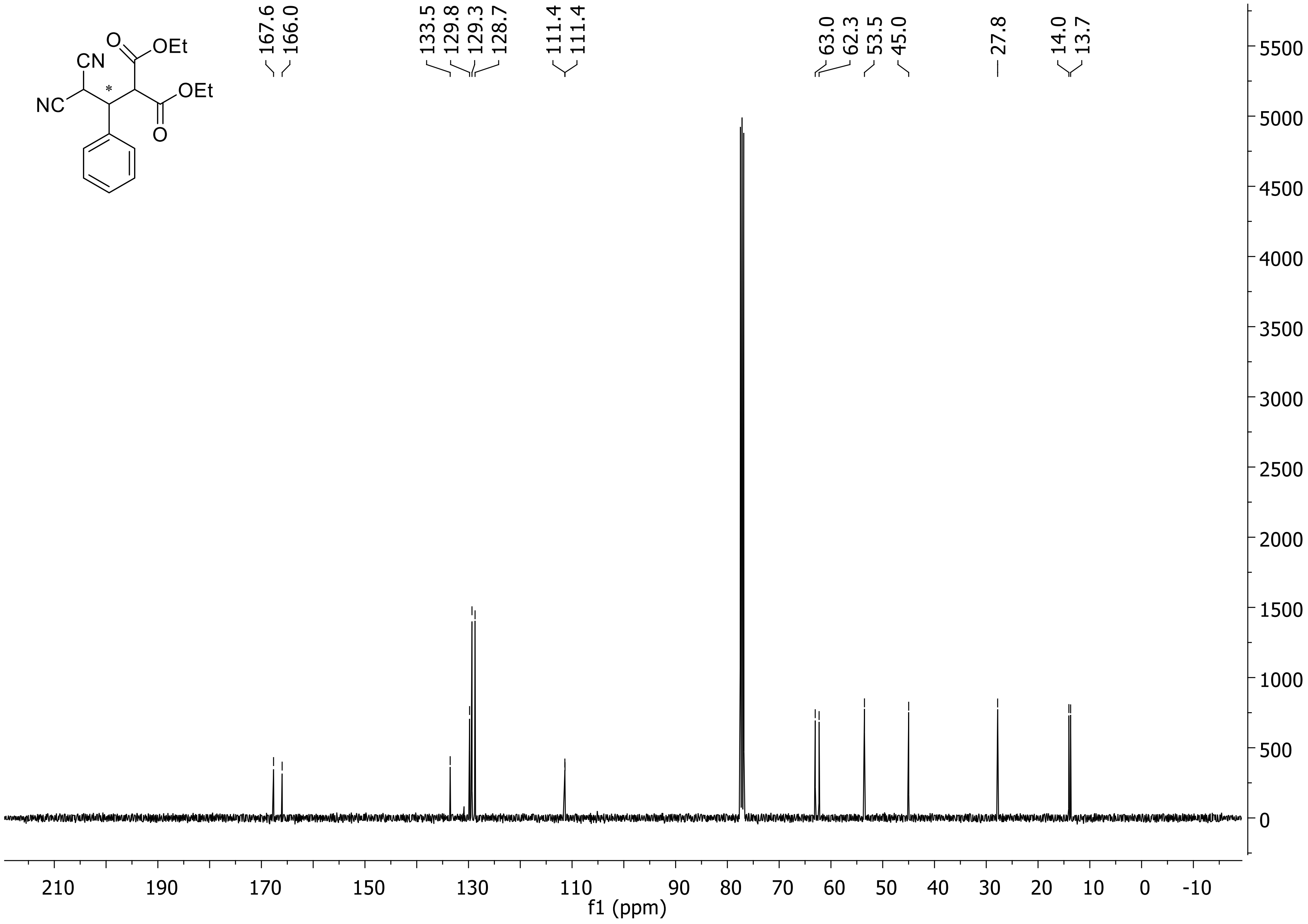

Figure S172. $^{13}C\{^{1}H\}$ NMR spectrum of **7** ($CDCl_3$, 101 MHz).

13 HPLC chromatograms of Michael reaction products

3a-major

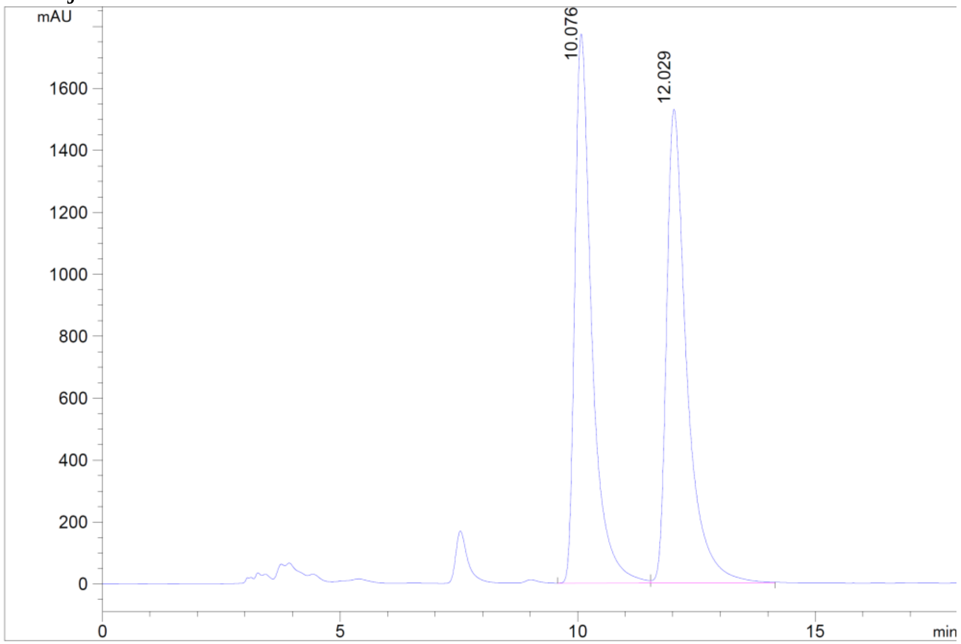

Signal 1: VWD1 A, Wavelength=210 nm

Peak #	RT [min]	Type	Width [min]	Area	Area %	Name
1	10.076	BV	0.370	44076.816	49.311	
2	12.029	MF	0.494	45308.855	50.689	

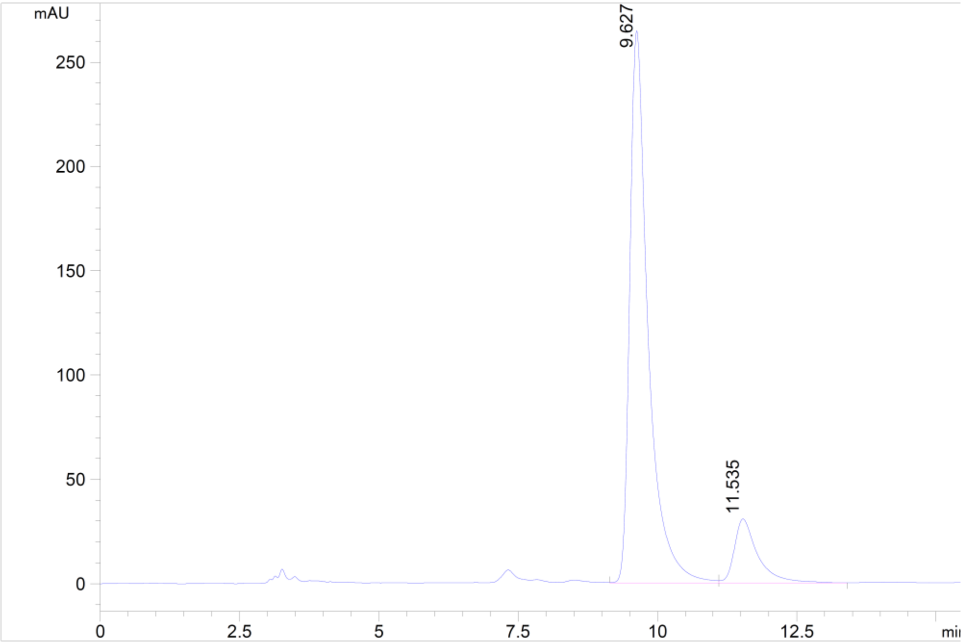

Signal 1: VWD1 A, Wavelength=210 nm

Peak #	RT [min]	Type	Width [min]	Area	Area %	Name
1	9.627	MF	0.381	6048.209	87.152	
2	11.535	FM	0.486	891.608	12.848	

3a-minor

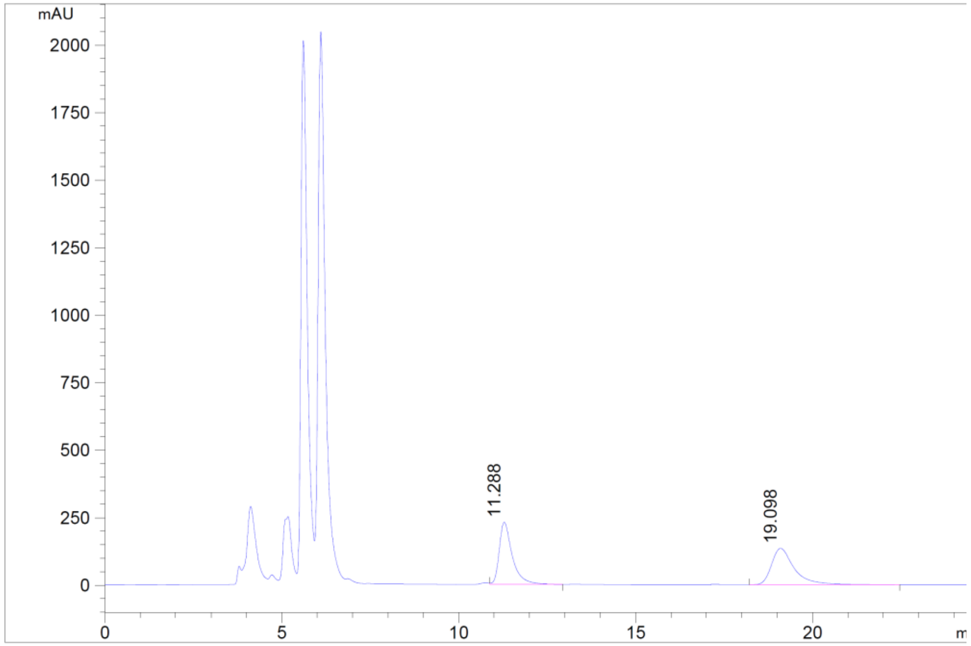

Signal 1: VWD1 A, Wavelength=210 nm

Peak #	RT [min]	Type	Width [min]	Area	Area %	Name
1	11.288	VB	0.381	5949.540	49.673	
2	19.098	BB	0.663	6027.797	50.327	

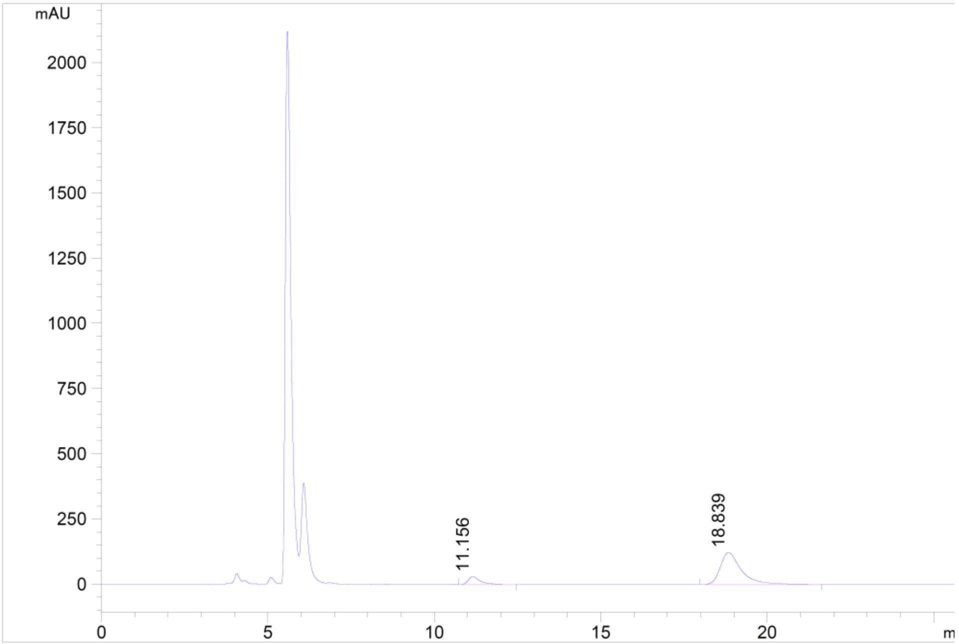

Signal 1: VWD1 A, Wavelength=210 nm

Peak #	RT [min]	Type	Width [min]	Area	Area %	Name
1	11.156	VB	0.358	733.666	12.069	
2	18.839	BB	0.630	5345.410	87.931	

3b-major

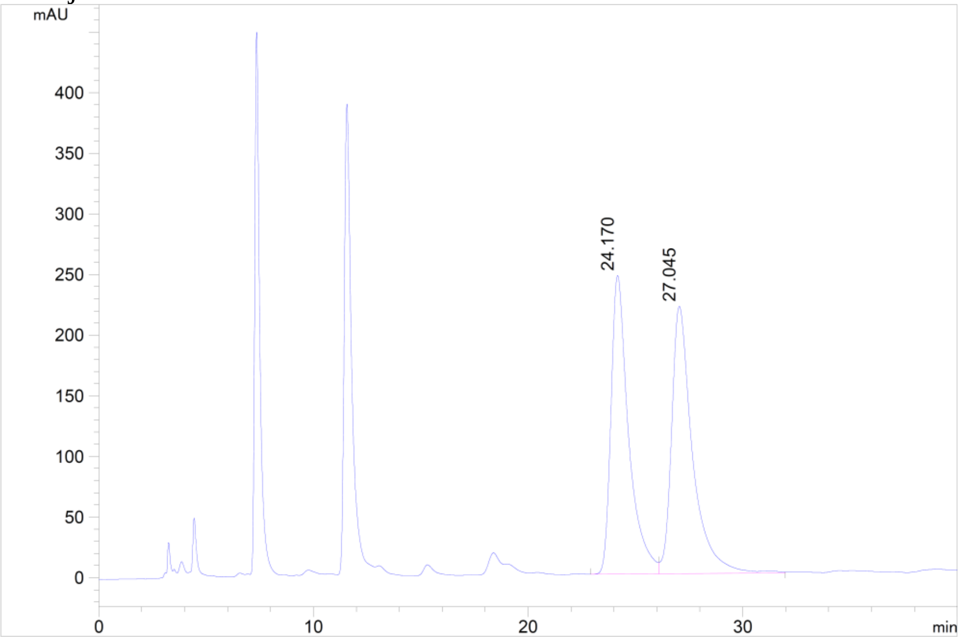

Signal 1: VWD1 A, Wavelength=210 nm

Peak #	RT [min]	Type	Width [min]	Area	Area %	Name
1	24.170	MF	0.953	14077.498	48.509	
2	27.045	FM	1.130	14943.086	51.491	

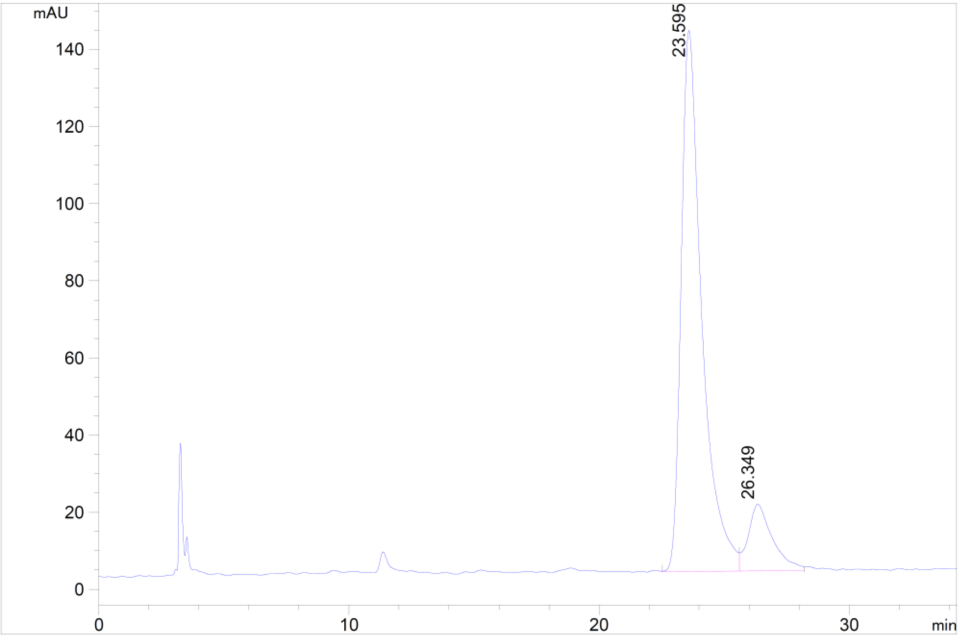

Signal 1: VWD1 A, Wavelength=210 nm

Peak #	RT [min]	Type	Width [min]	Area	Area %	Name
1	23.595	MF	0.962	8095.447	87.334	
2	26.349	MF	1.130	1174.034	12.666	

3b-minor

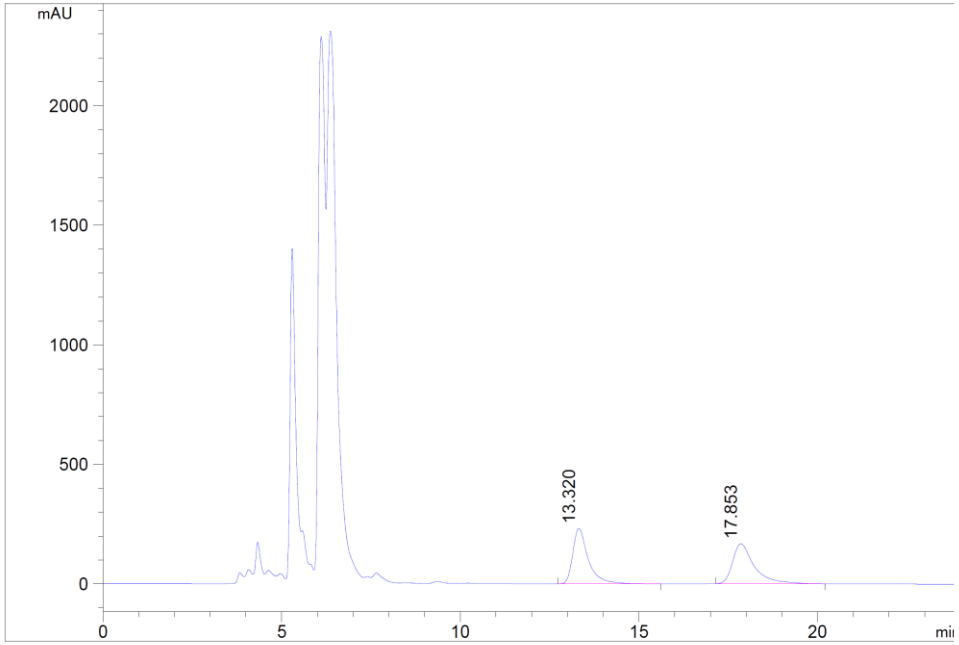

Signal 1: VWD1 A, Wavelength=210 nm

Peak #	RT [min]	Type	Width [min]	Area	Area %	Name
1	13.320	MM	0.502	6994.562	49.200	
2	17.853	MF	0.714	7222.111	50.800	

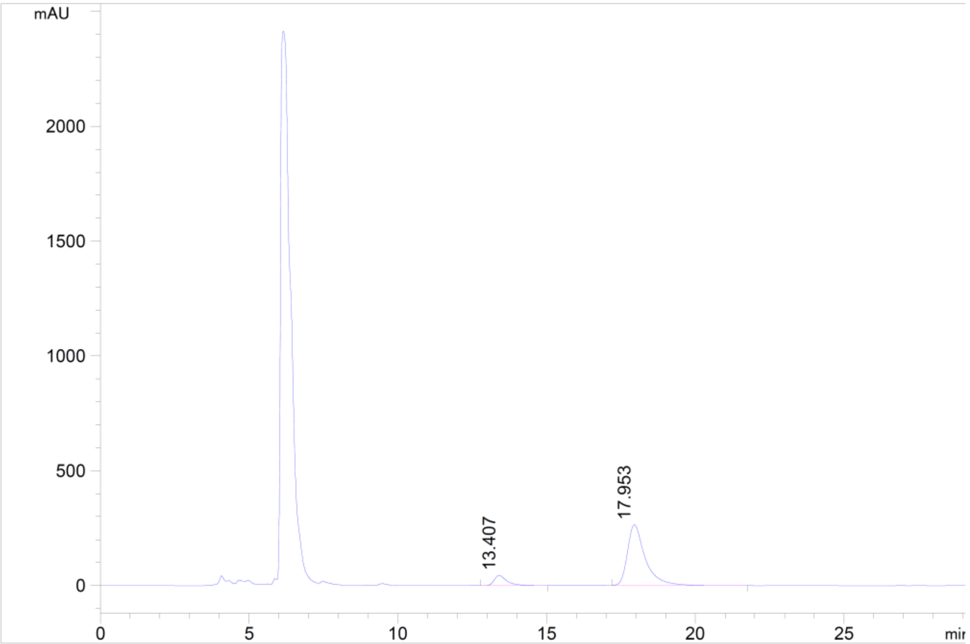

Signal 1: VWD1 A, Wavelength=210 nm

Peak #	RT [min]	Type	Width [min]	Area	Area %	Name
1	13.407	FM	0.498	1325.464	10.643	
2	17.953	FM	0.700	11128.658	89.357	

3c-major

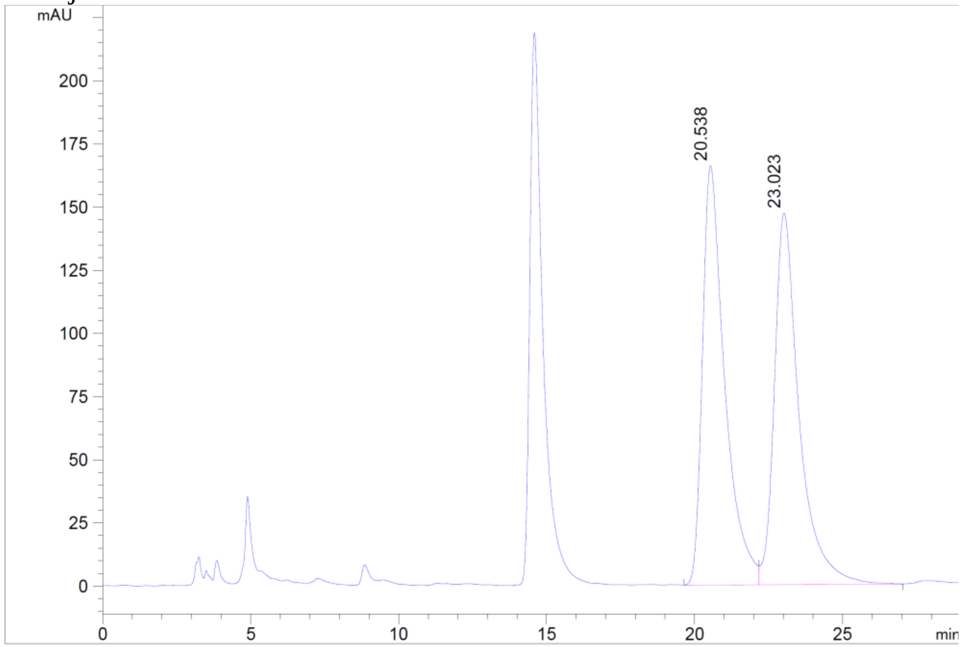

Signal 1: VWD1 A, Wavelength=210 nm

Peak #	RT [min]	Type	Width [min]	Area	Area %	Name
1	20.538	MF	0.874	8709.450	50.265	
2	23.023	FM	0.977	8617.520	49.735	

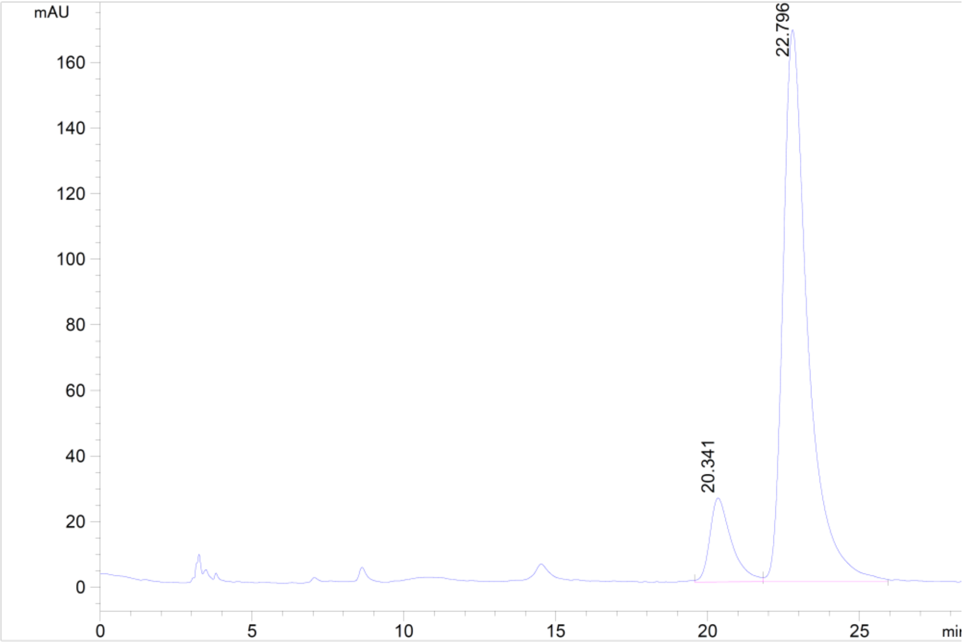

Signal 1: VWD1 A, Wavelength=210 nm

Peak #	RT [min]	Type	Width [min]	Area	Area %	Name
1	20.341	MF	0.829	1271.515	11.743	
2	22.796	MF	0.947	9556.321	88.257	

3c-minor

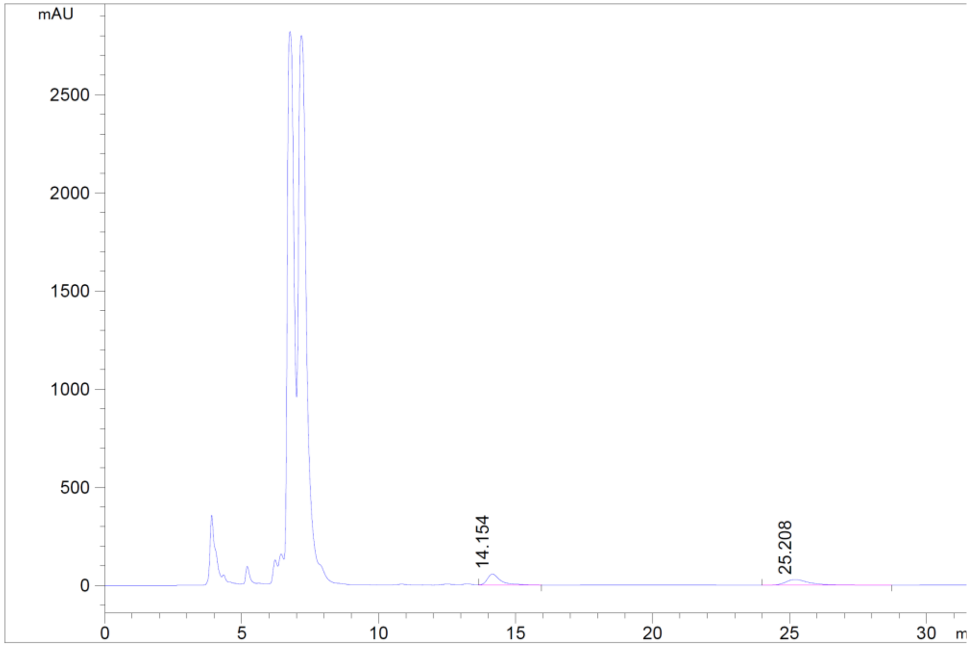

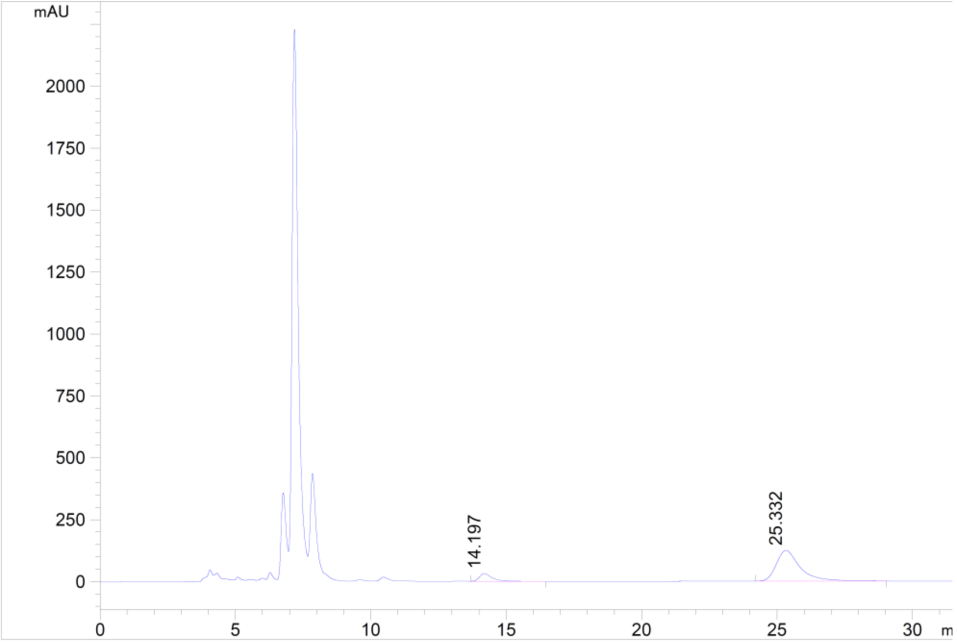

Signal 1: VWD1 A, Wavelength=210 nm

Peak #	RT [min]	Type	Width [min]	Area	Area %	Name
1	14.154	MF	0.579	1976.343	52.822	
2	25.208	MM	1.007	1765.159	47.178	

Signal 1: VWD1 A, Wavelength=210 nm

Peak #	RT [min]	Type	Width [min]	Area	Area %	Name
1	14.197	FM	0.554	1048.319	12.121	
2	25.332	MM	1.020	7600.195	87.879	

3d-major

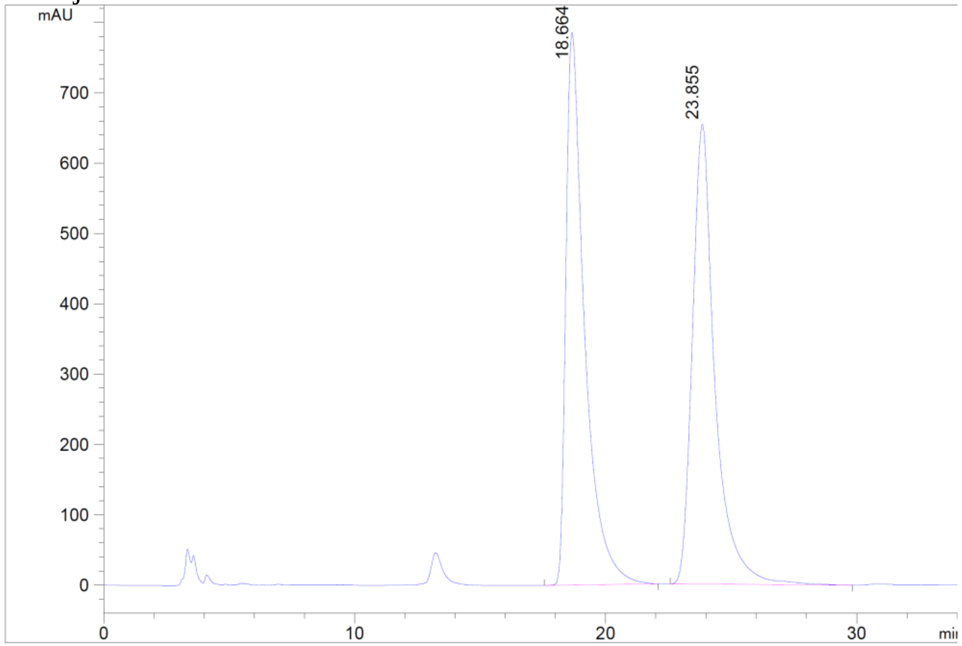

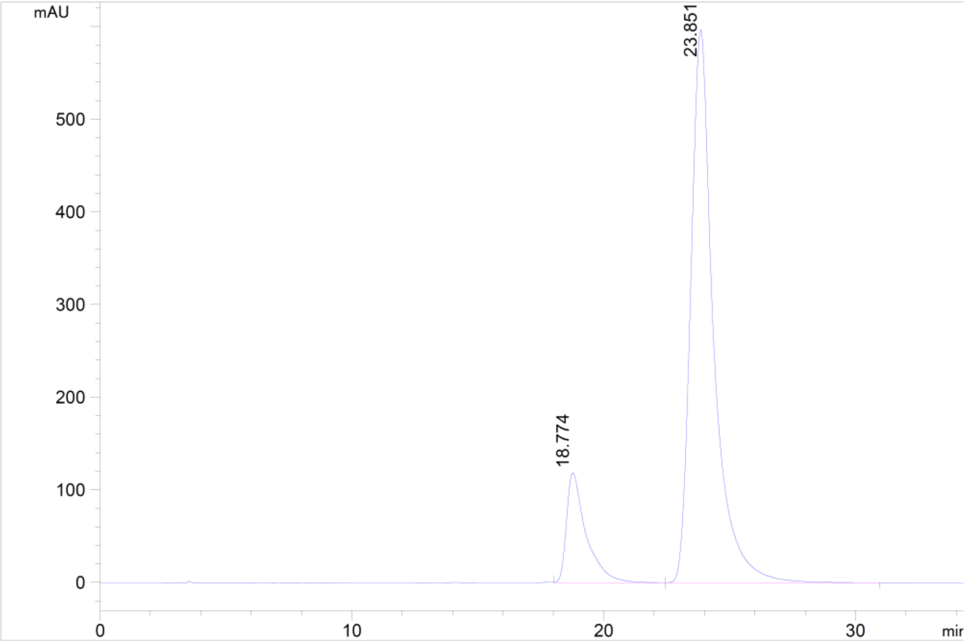

Signal 1: VWD1 A, Wavelength=254 nm

Peak #	RT [min]	Type	Width [min]	Area	Area %	Name
1	18.664	BB	0.758	40692.008	49.990	
2	23.855	BB	0.911	40707.973	50.010	

Signal 1: VWD1 A, Wavelength=254 nm

Peak #	RT [min]	Type	Width [min]	Area	Area %	Name
1	18.774	VB	0.777	6408.984	14.524	
2	23.851	BB	0.928	37718.797	85.476	

3d-minor

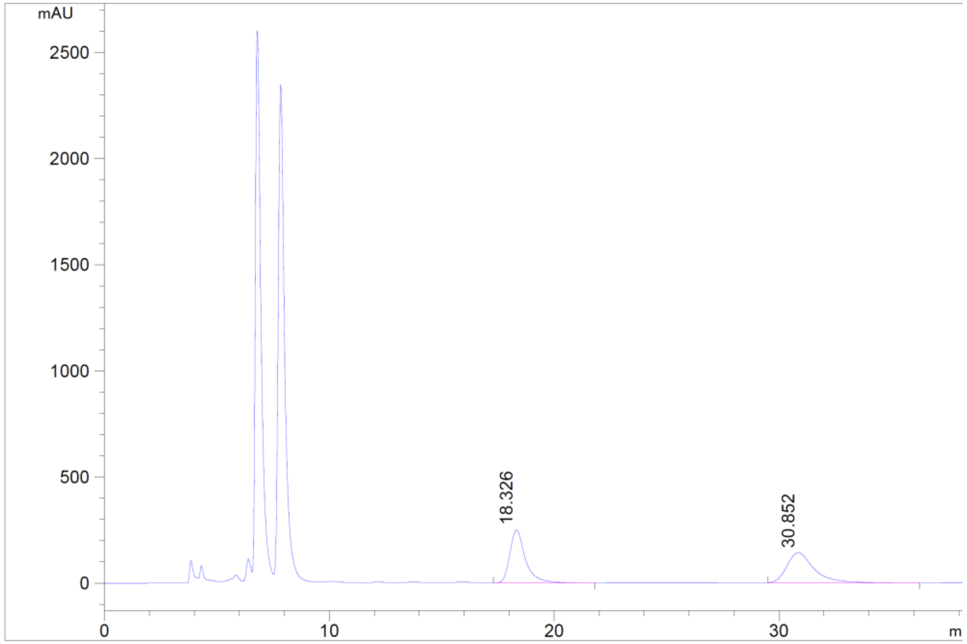

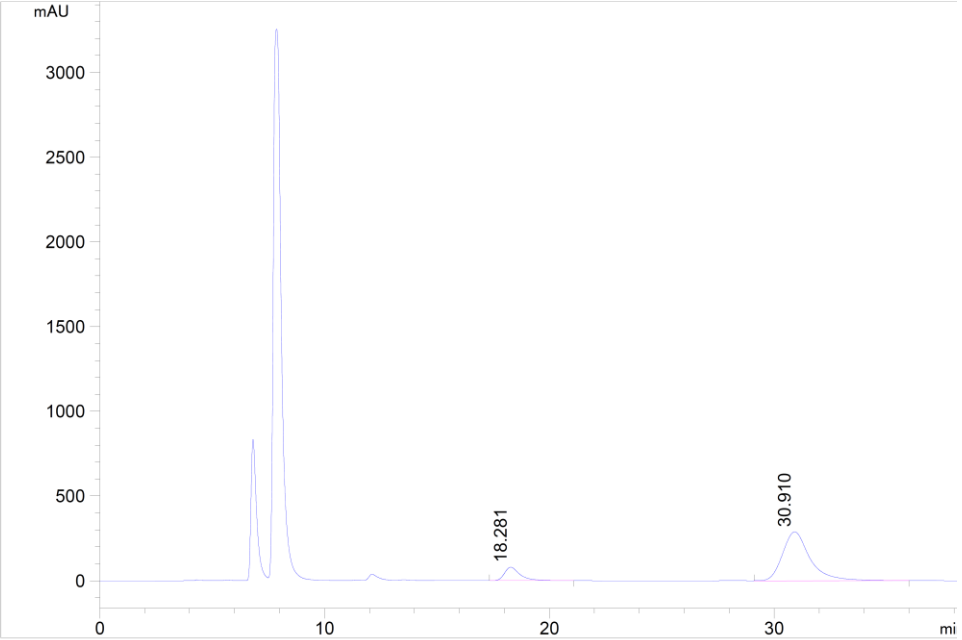

Signal 1: VWD1 A, Wavelength=254 nm

Peak #	RT [min]	Type	Width [min]	Area	Area %	Name
1	18.326	MM	0.803	12017.086	49.442	
2	30.852	FM	1.431	12288.468	50.558	

Signal 1: VWD1 A, Wavelength=254 nm

Peak #	RT [min]	Type	Width [min]	Area	Area %	Name
1	18.281	MM	0.809	3873.087	13.685	
2	30.910	MM	1.412	24427.666	86.315	

3e-major

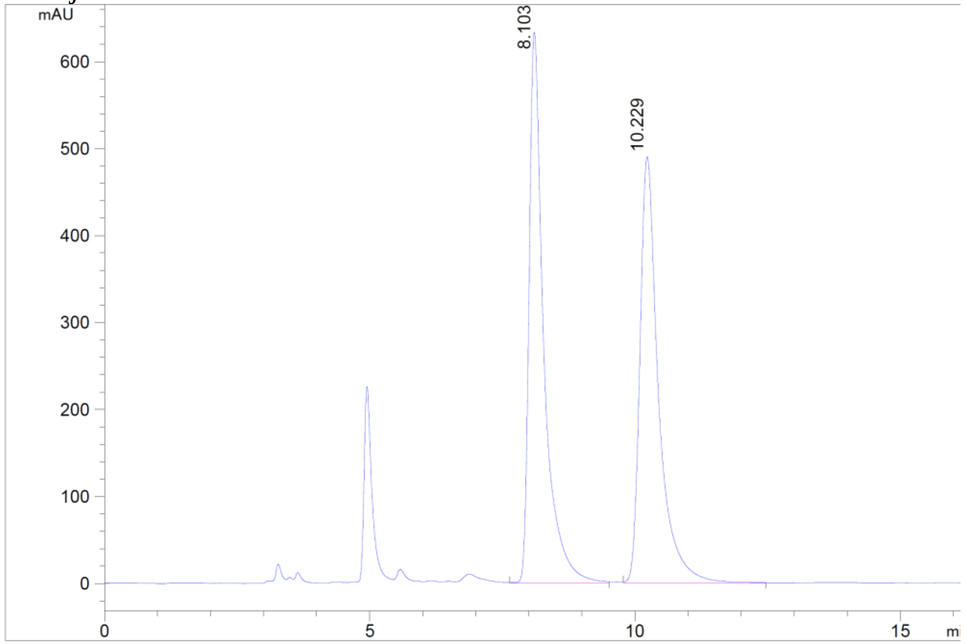

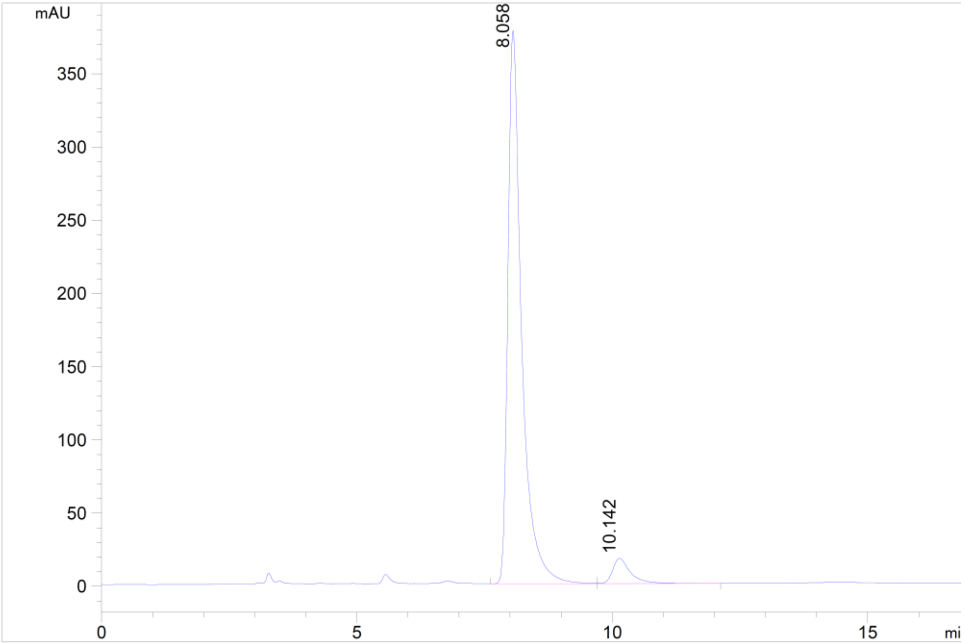

Signal 1: VWD1 A, Wavelength=210 nm

Peak #	RT [min]	Type	Width [min]	Area	Area %	Name
1	8.103	MF	0.321	12186.115	50.649	
2	10.229	FM	0.404	11873.958	49.351	

Signal 1: VWD1 A, Wavelength=210 nm

Peak #	RT [min]	Type	Width [min]	Area	Area %	Name
1	8.058	MF	0.311	7048.322	94.355	
2	10.142	FM	0.408	421.721	5.645	

3e-minor

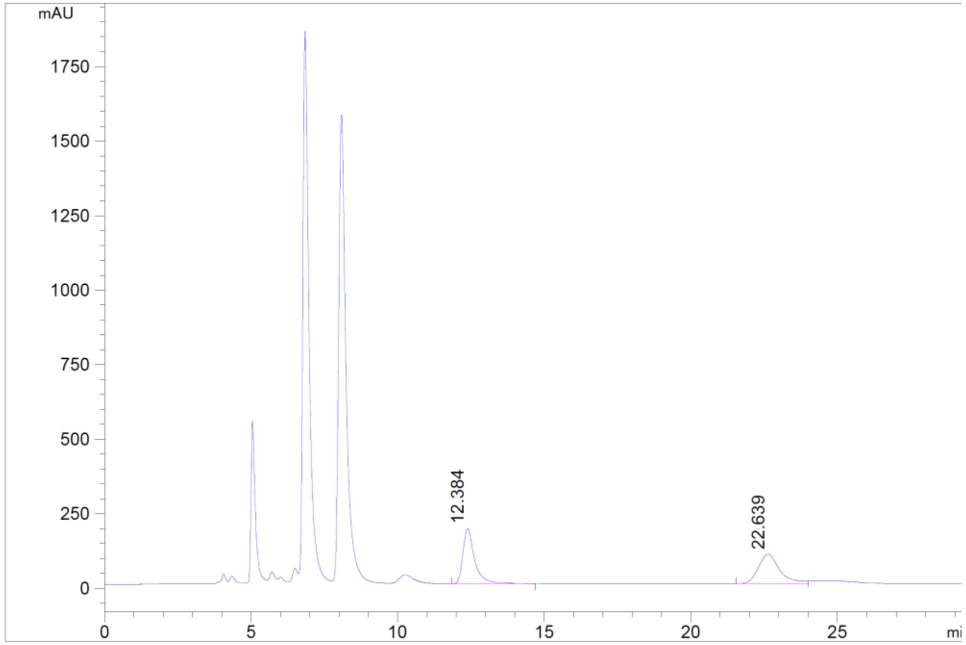

Signal 1: VWD1 A, Wavelength=210 nm

Peak #	RT [min]	Type	Width [min]	Area	Area %	Name
1	12.384	BB	0.406	5172.836	50.376	
2	22.639	MF	0.859	5095.676	49.624	

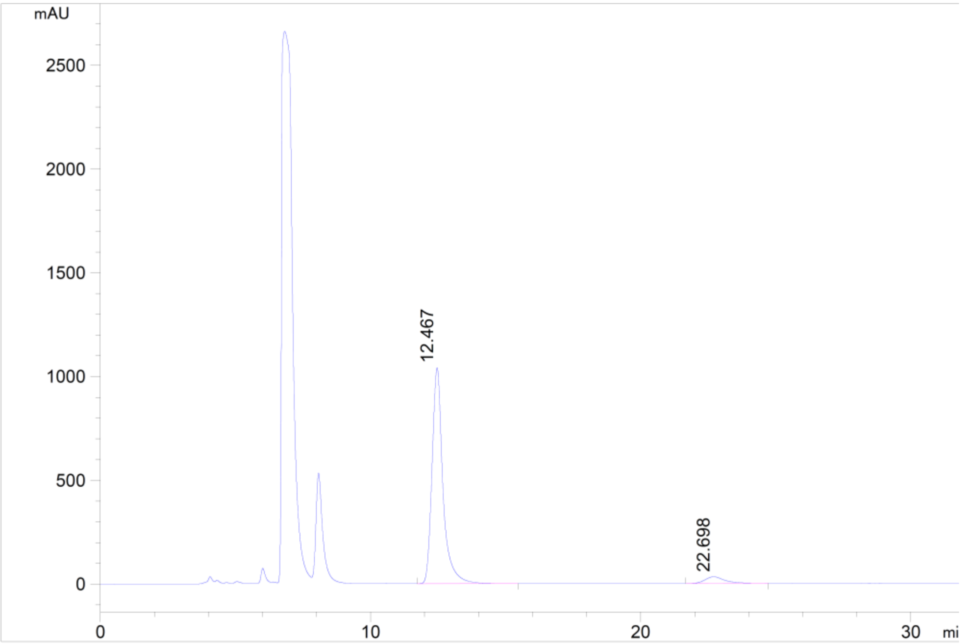

Signal 1: VWD1 A, Wavelength=210 nm

Peak #	RT [min]	Type	Width [min]	Area	Area %	Name
1	12.467	MM	0.469	29253.467	94.552	
2	22.698	MM	0.867	1685.465	5.448	

3f-major

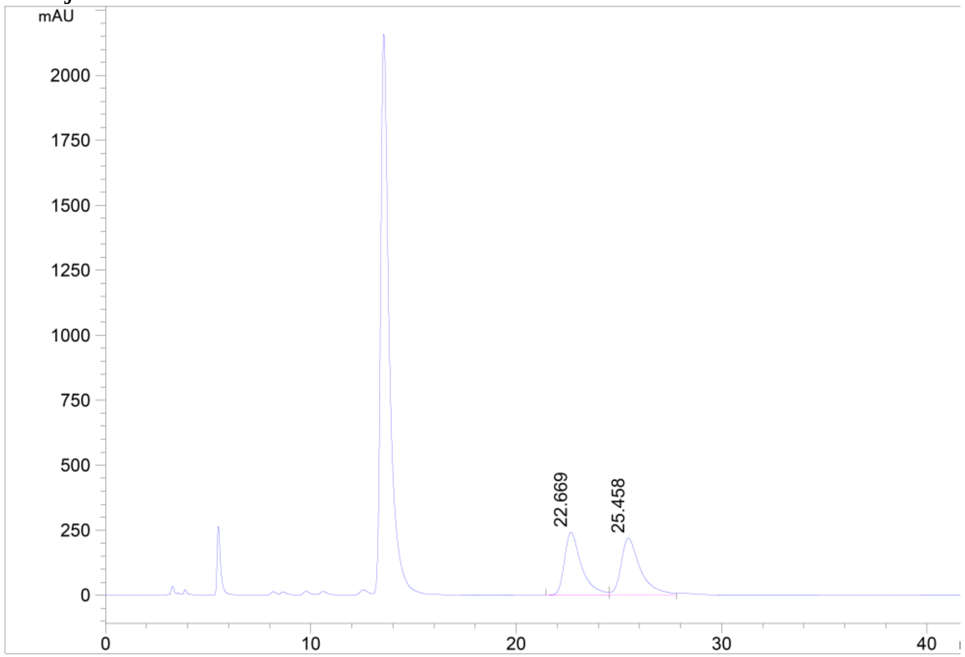

Signal 1: VWD1 A, Wavelength=210 nm

Peak #	RT [min]	Type	Width [min]	Area	Area %	Name
1	22.669	MF	0.958	13929.934	49.421	
2	25.458	MF	1.084	14256.069	50.579	

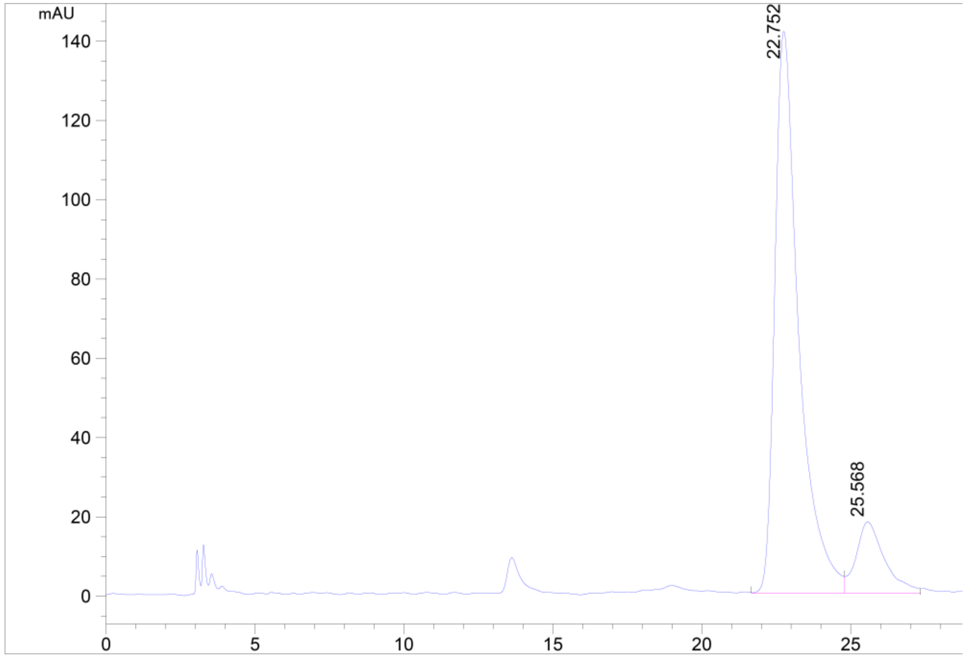

Signal 1: VWD1 A, Wavelength=210 nm

Peak #	RT [min]	Type	Width [min]	Area	Area %	Name
1	22.752	MF	0.935	7945.864	86.931	
2	25.568	MF	1.106	1194.579	13.069	

3f-major and minor

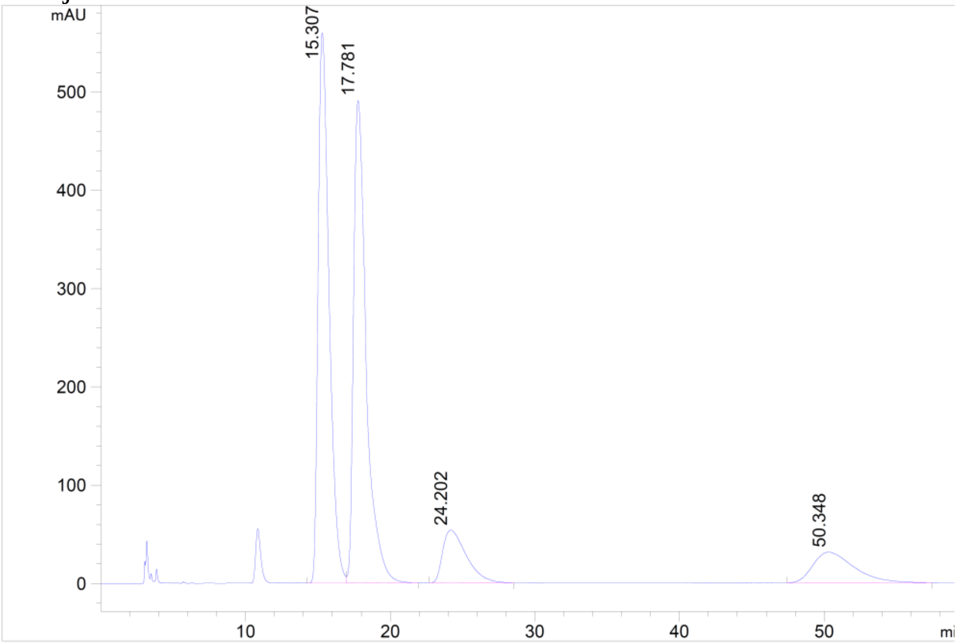

Signal 1: MWD1 C, Sig=210,8 Ref=360,100

Peak #	RT [min]	Type	Width [min]	Area	Area %	Name
1	15.307	MF	0.877	29470.990	41.269	
2	17.781	FM	1.010	29725.453	41.625	
3	24.202	MM	1.889	6077.395	8.510	
4	50.348	MM	3.279	6138.611	8.596	

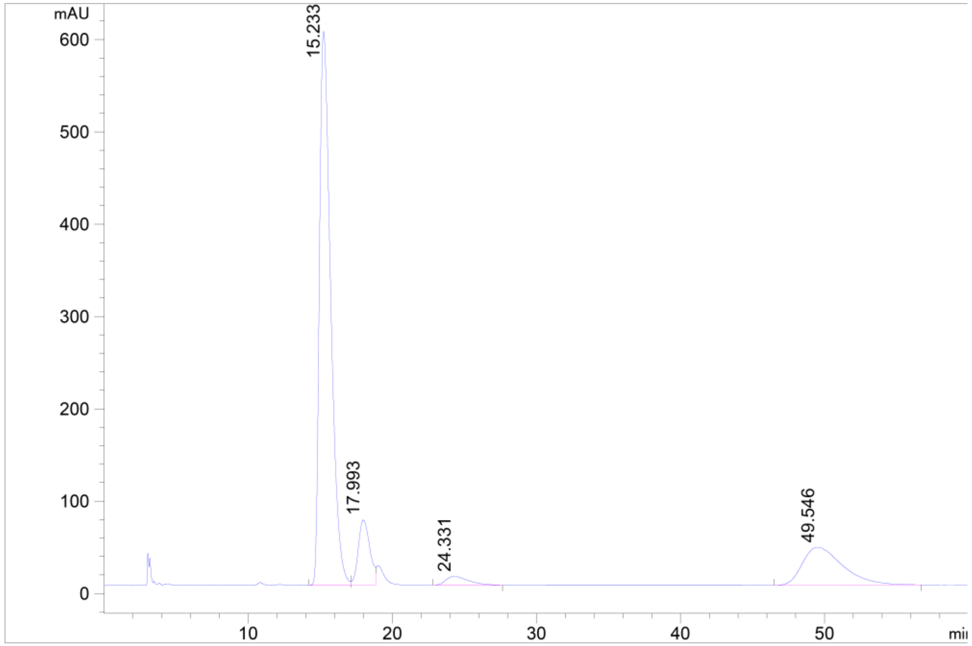

Signal 1: MWD1 C, Sig=210,8 Ref=360,100

Peak #	RT [min]	Type	Width [min]	Area	Area %	Name
1	15.233	MF	0.904	32532.064	71.466	
2	17.993	MF	0.939	3980.783	8.745	
3	24.331	MM	1.806	1017.035	2.234	
4	49.546	MM	3.250	7991.043	17.555	

3g-major

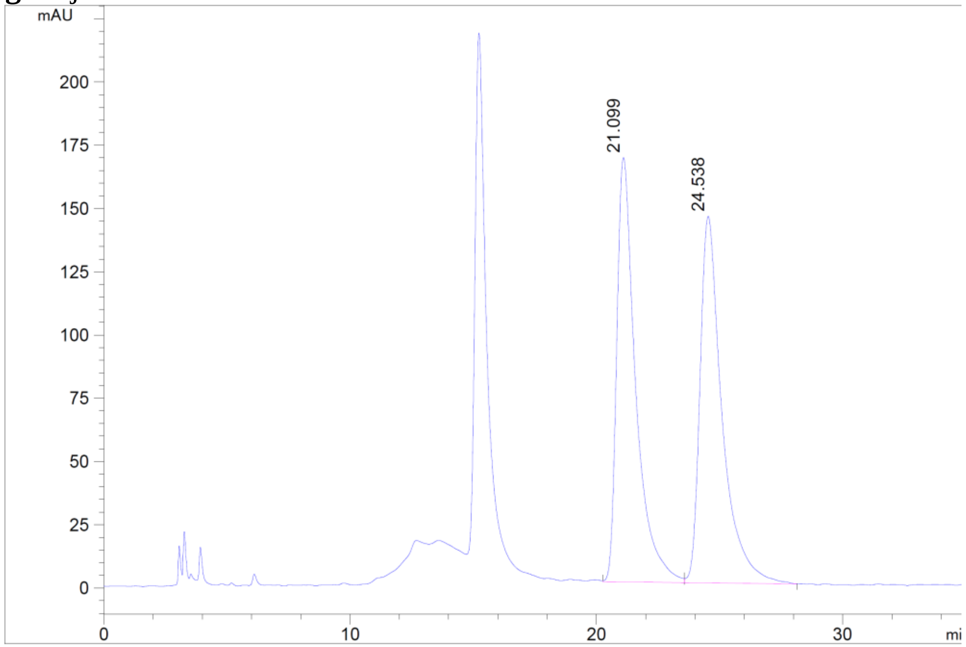

Signal 1: VWD1 A, Wavelength=210 nm

Peak #	RT [min]	Type	Width [min]	Area	Area %	Name
1	21.099	MF	0.892	8973.889	49.838	
2	24.538	FM	1.039	9032.273	50.162	

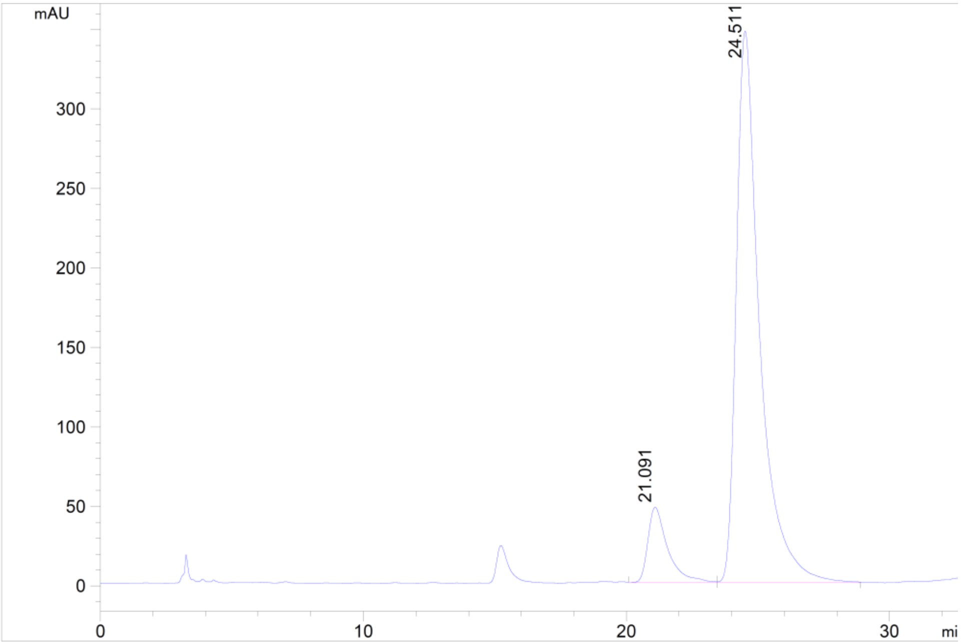

Signal 1: VWD1 A, Wavelength=210 nm

Peak #	RT [min]	Type	Width [min]	Area	Area %	Name
1	21.091	MF	0.871	2464.599	10.315	
2	24.511	FM	1.030	21428.115	89.685	

3g-minor

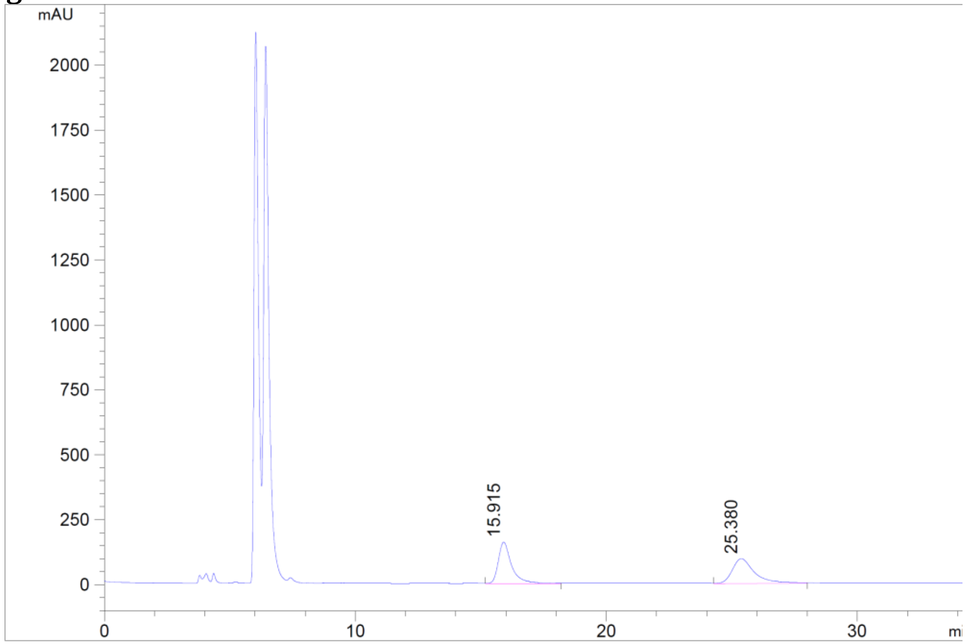

Signal 1: VWD1 A, Wavelength=210 nm

Peak #	RT [min]	Type	Width [min]	Area	Area %	Name
1	15.915	MM	0.616	5903.063	49.936	
2	25.380	MF	1.031	5918.186	50.064	

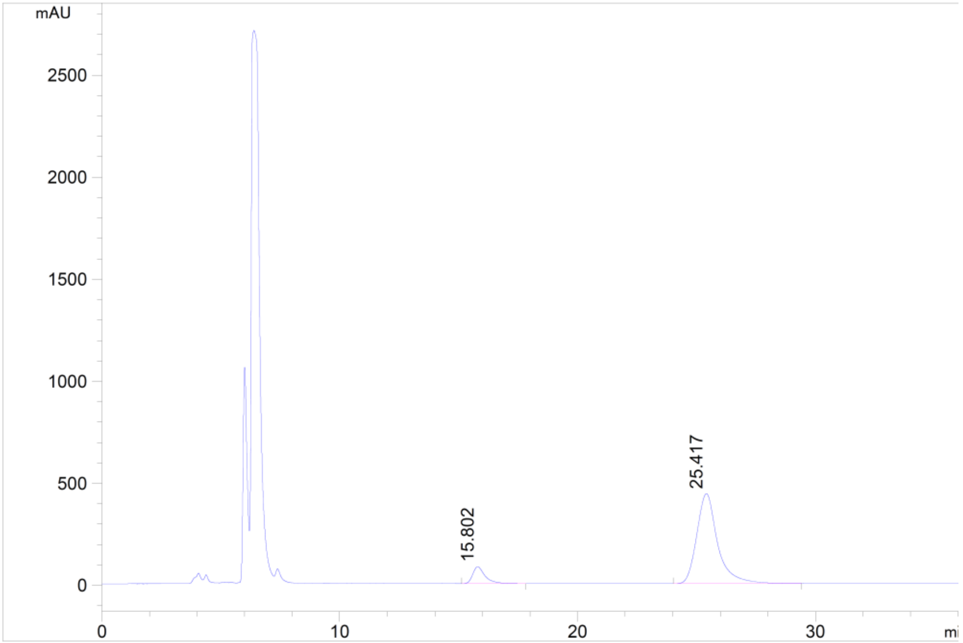

Signal 1: VWD1 A, Wavelength=210 nm

Peak #	RT [min]	Type	Width [min]	Area	Area %	Name
1	15.802	MF	0.608	3018.966	10.040	
2	25.417	MF	1.022	27051.256	89.960	

3h- major and minor

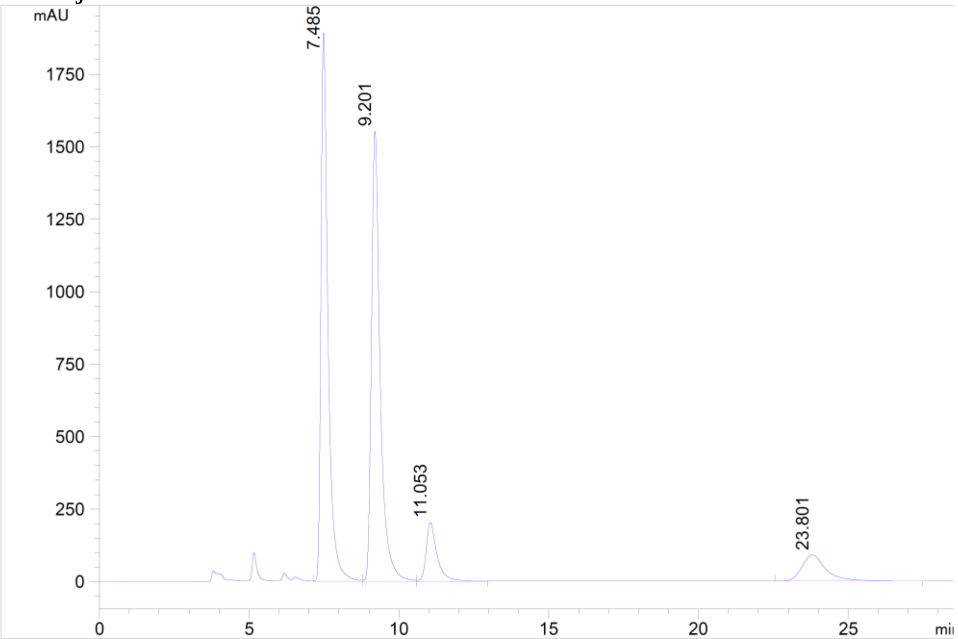

Signal 1: VWD1 A, Wavelength=210 nm

Peak #	RT [min]	Type	Width [min]	Area	Area %	Name
1	7.485	MF	0.275	31221.715	42.765	
2	9.201	MF	0.338	31504.777	43.153	
3	11.053	FM	0.428	5186.632	7.104	
4	23.801	MM	0.938	5093.688	6.977	

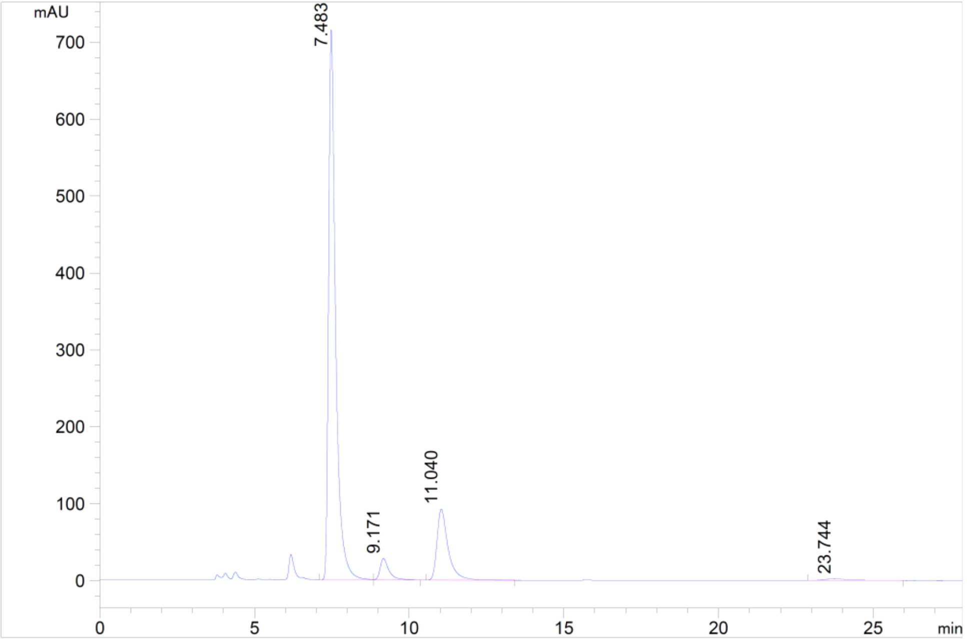

Signal 1: VWD1 A, Wavelength=210 nm

Peak #	RT [min]	Type	Width [min]	Area	Area %	Name
1	7.483	MF	0.264	11328.243	79.197	
2	9.171	MF	0.333	560.002	3.915	
3	11.040	MM	0.417	2304.149	16.109	
4	23.744	MM	0.917	111.400	0.779	

3i- major and minor

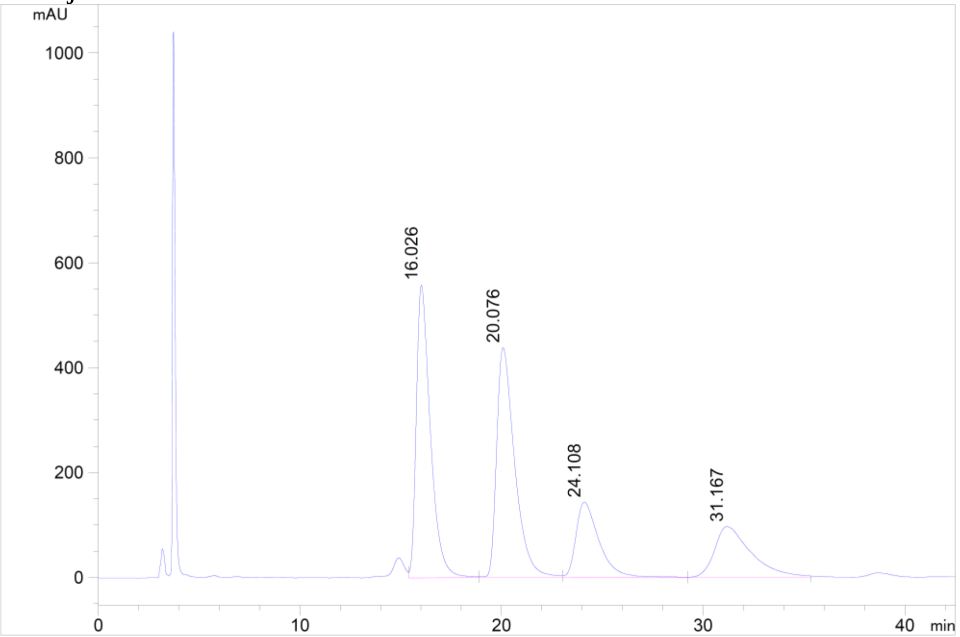

Signal 1: VWD1 A, Wavelength=210 nm

Peak #	RT [min]	Type	Width [min]	Area	Area %	Name
1	16.026	FM	0.810	27133.293	34.598	
2	20.076	FM	1.039	27376.523	34.908	
3	24.108	MF	1.339	11575.291	14.760	
4	31.167	MF	2.118	12340.450	15.735	

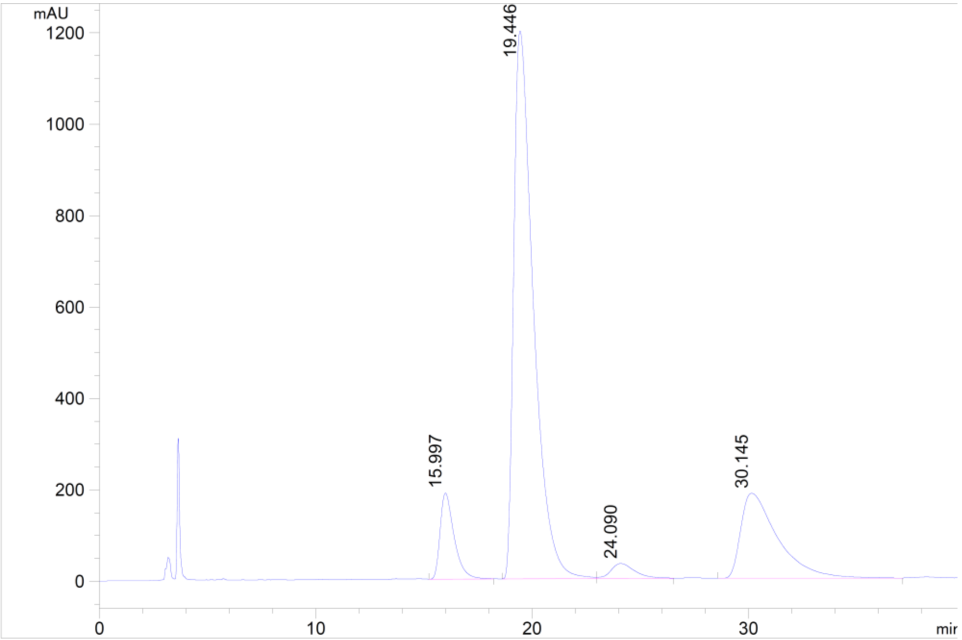

Signal 1: VWD1 A, Wavelength=210 nm

Peak #	RT [min]	Type	Width [min]	Area	Area %	Name
1	15.997	MF	0.749	8458.366	7.787	
2	19.446	MF	1.052	75627.391	69.623	
3	24.090	MF	1.291	2561.618	2.358	
4	30.145	MM	1.972	21977.127	20.232	

3j-major

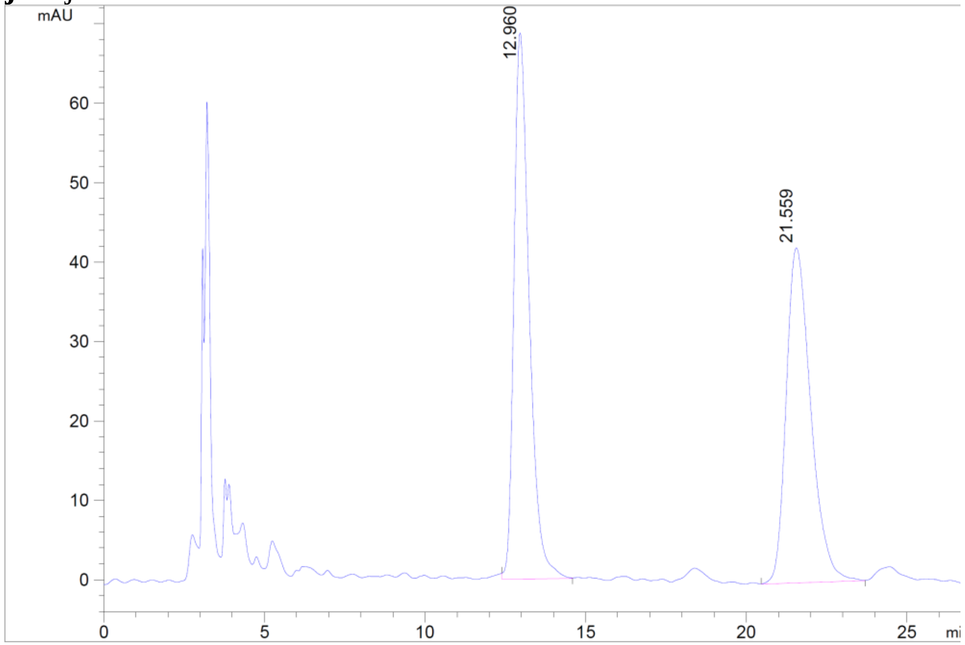

Signal 1: VWD1 A, Wavelength=210 nm

Peak #	RT [min]	Type	Width [min]	Area	Area %	Name
1	12.960	FM	0.550	2269.390	50.256	
2	21.559	MM	0.888	2246.293	49.744	

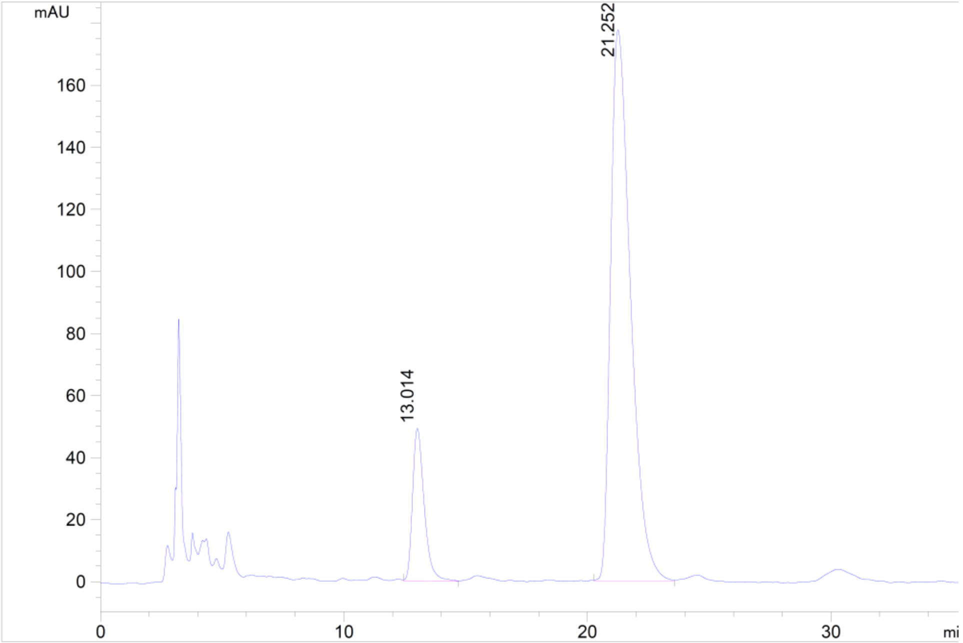

Signal 1: VWD1 A, Wavelength=210 nm

Peak #	RT [min]	Type	Width [min]	Area	Area %	Name
1	13.014	FM	0.555	1635.121	13.883	
2	21.252	MF	0.951	10143.022	86.117	

3j-minor

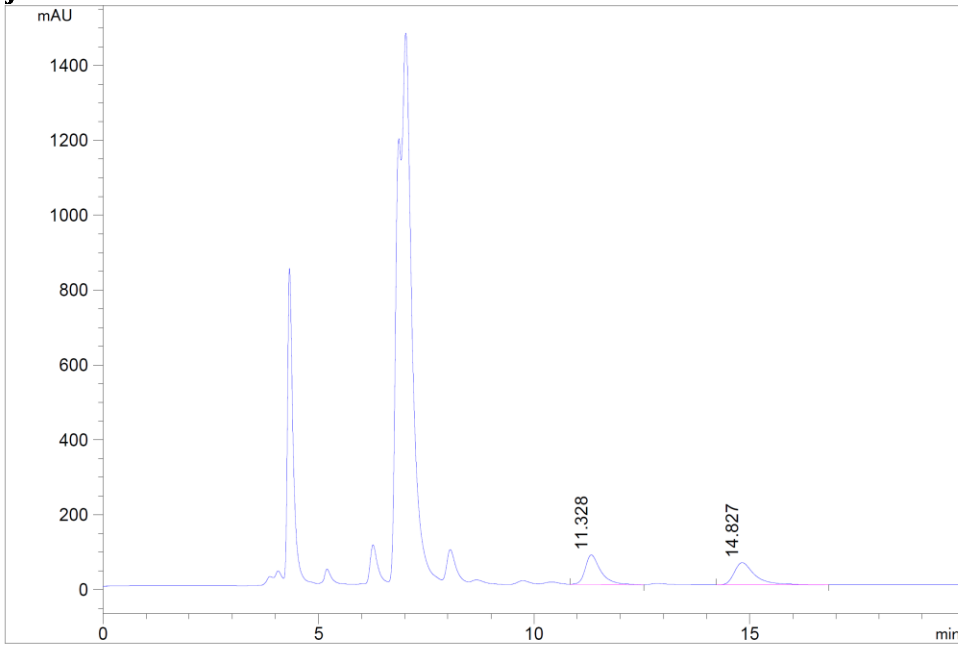

Signal 1: VWD1 A, Wavelength=210 nm

Peak #	RT [min]	Type	Width [min]	Area	Area %	Name
1	11.328	VB	0.371	2007.206	49.973	
2	14.827	BB	0.486	2009.400	50.027	

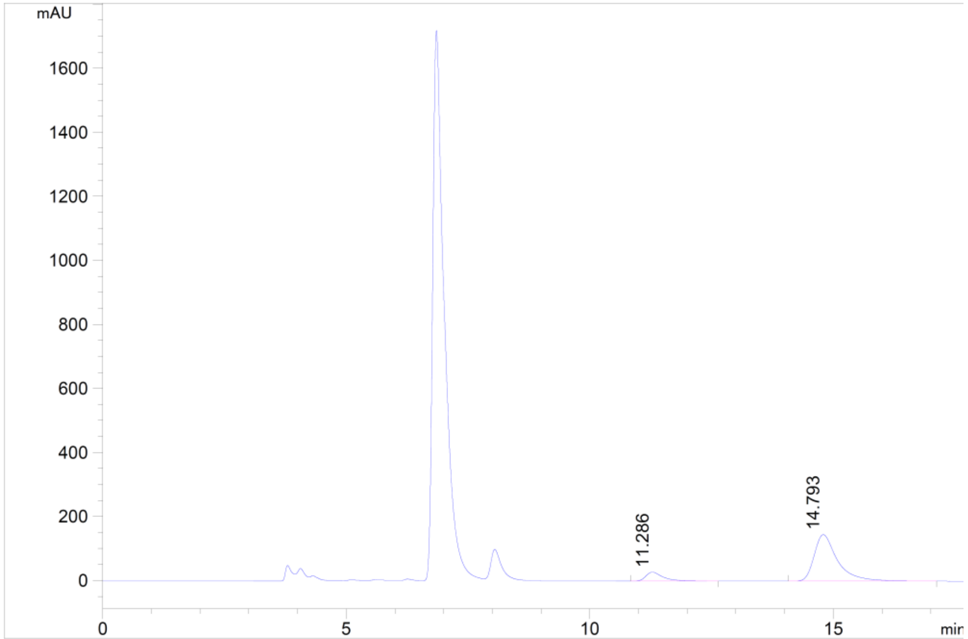

Signal 1: VWD1 A, Wavelength=210 nm

Peak #	RT [min]	Type	Width [min]	Area	Area %	Name
1	11.286	BV	0.355	698.226	12.573	
2	14.793	BB	0.490	4855.063	87.427	

3kmajor

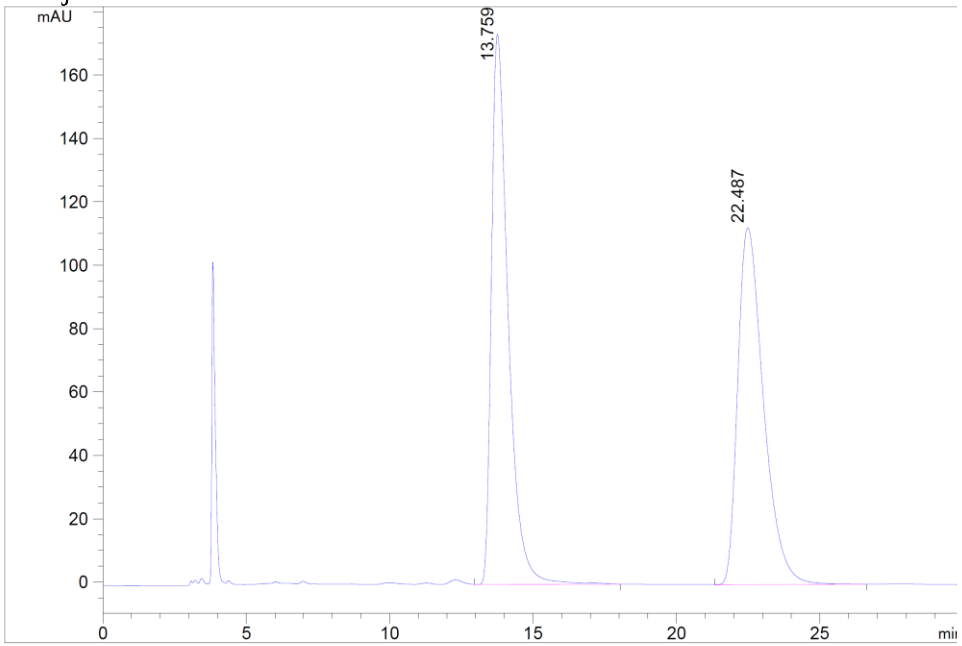

Signal 1: VWD1 A, Wavelength=254 nm

Peak #	RT [min]	Type	Width [min]	Area	Area %	Name
1	13.759	MM	0.677	7049.949	50.055	
2	22.487	MM	1.041	7034.564	49.945	

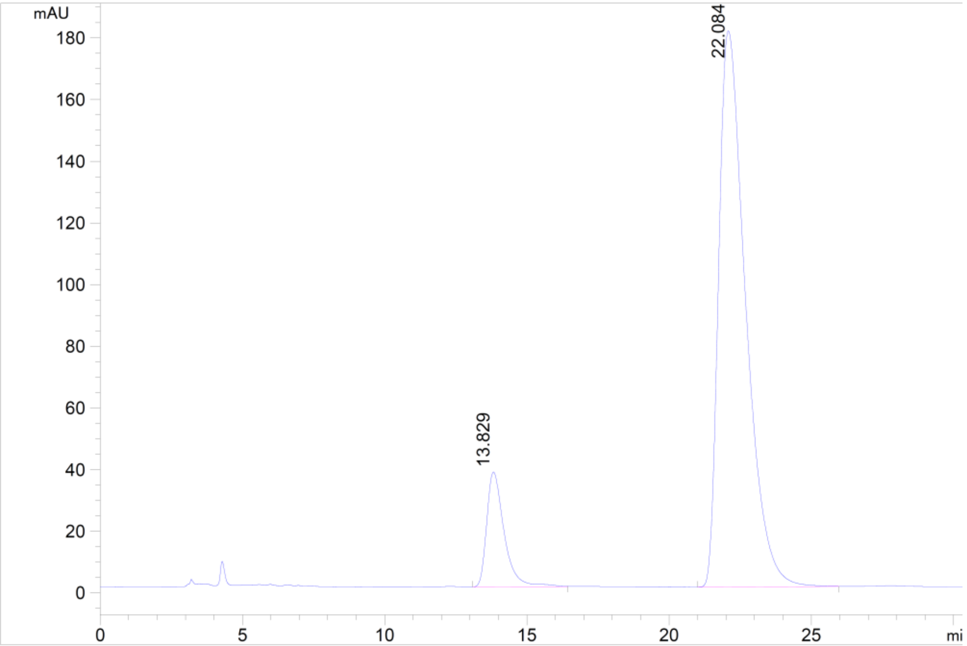

Signal 1: VWD1 A, Wavelength=254 nm

Peak #	RT [min]	Type	Width [min]	Area	Area %	Name
1	13.829	MM	0.711	1588.040	11.802	
2	22.084	BB	0.987	11867.390	88.198	

3k-minor

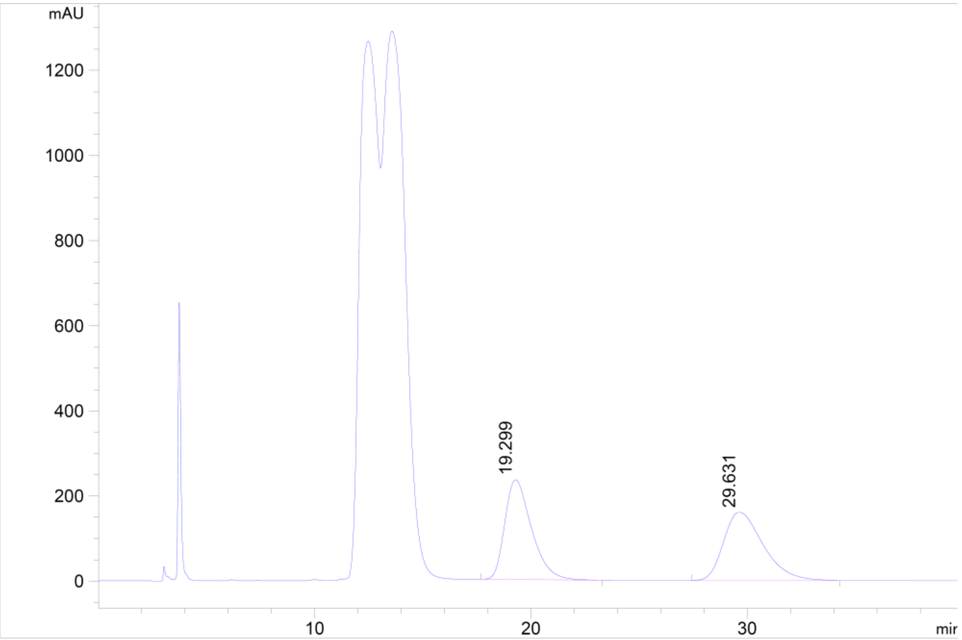

Signal 1: MWD1 D, Sig=230,16 Ref=360,100

Peak #	RT [min]	Type	Width [min]	Area	Area %	Name
1	19.299	BB	1.298	20130.627	49.628	
2	29.631	BB	1.927	20432.406	50.372	

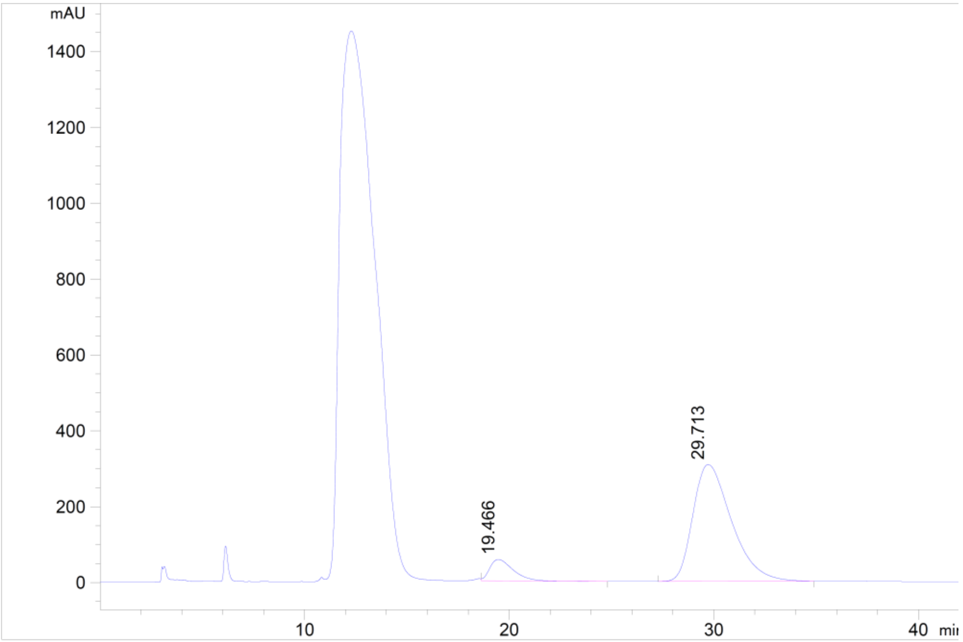

Signal 1: MWD1 D, Sig=230,16 Ref=360,100

Peak #	RT [min]	Type	Width [min]	Area	Area %	Name
1	19.466	FM	1.332	4509.781	10.034	
2	29.713	BB	1.930	40437.371	89.966	

3l-major

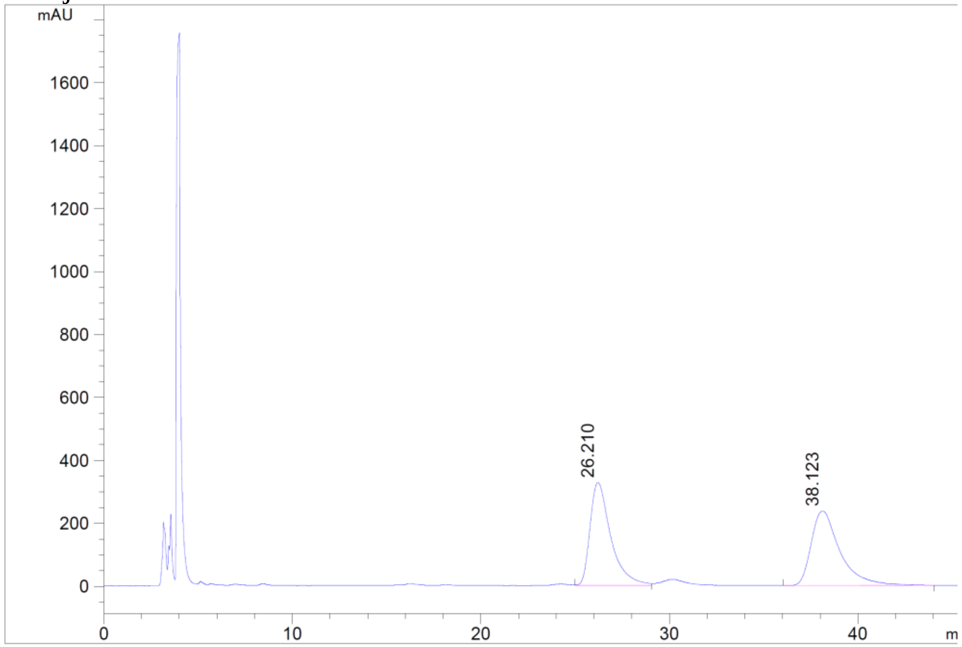

Signal 1: VWD1 A, Wavelength=210 nm

Peak #	RT [min]	Type	Width [min]	Area	Area %	Name
1	26.210	VV	1.114	24947.119	49.809	
2	38.123	BB	1.249	25138.127	50.191	

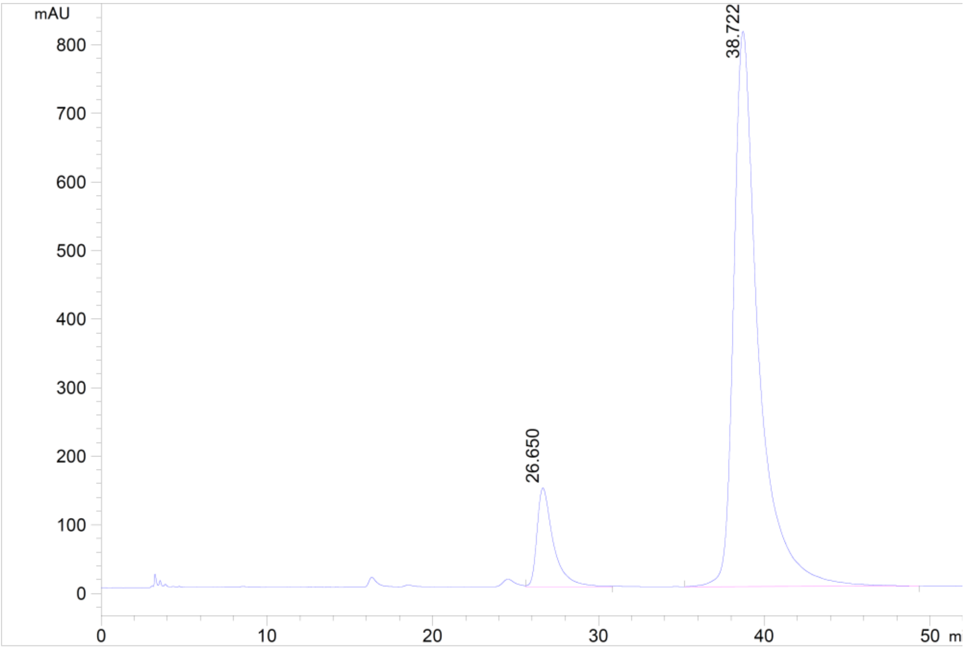

Signal 1: VWD1 A, Wavelength=210 nm

Peak #	RT [min]	Type	Width [min]	Area	Area %	Name
1	26.650	MF	1.147	9914.471	10.496	
2	38.722	MM	1.741	84546.875	89.504	

3l-minor

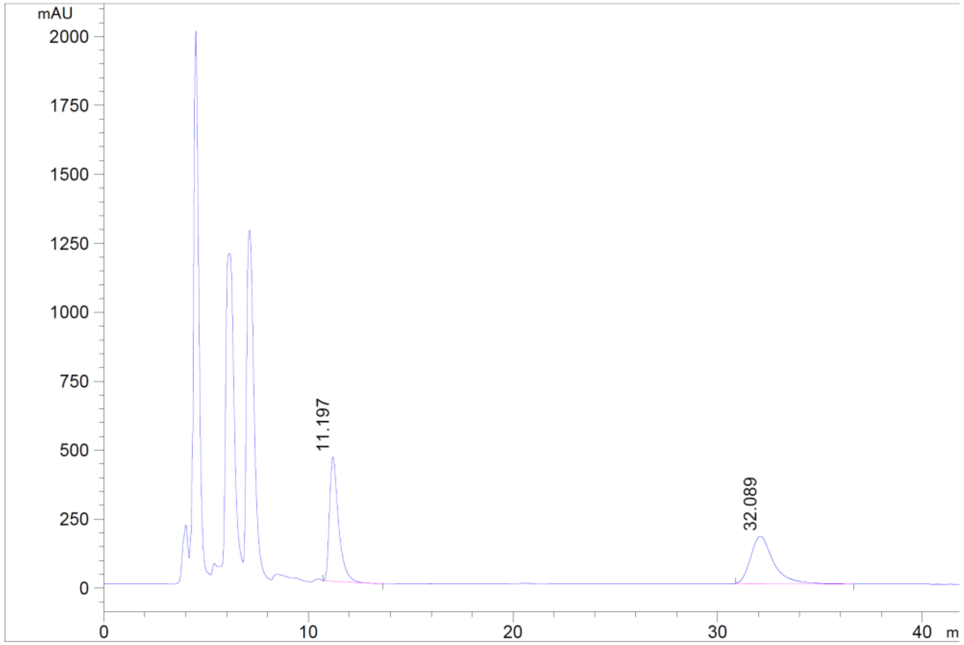

Signal 1: VWD1 A, Wavelength=210 nm

Peak #	RT [min]	Type	Width [min]	Area	Area %	Name
1	11.197	MM	0.510	13793.314	50.967	
2	32.089	BB	1.047	13269.653	49.033	

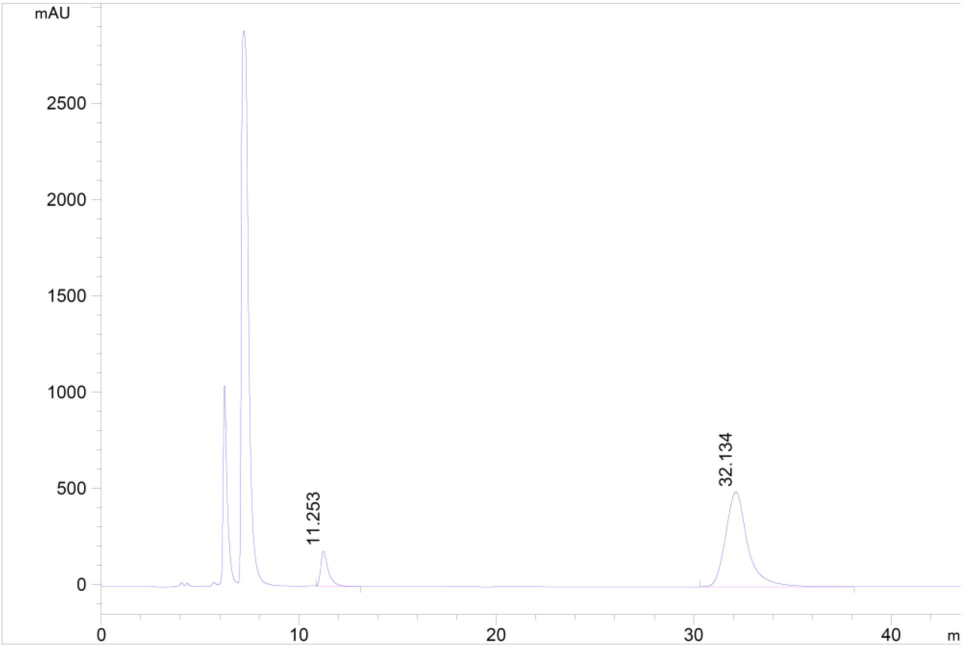

Signal 1: VWD1 A, Wavelength=210 nm

Peak #	RT [min]	Type	Width [min]	Area	Area %	Name
1	11.253	FM	0.455	5059.516	11.605	
2	32.134	MM	1.299	38536.883	88.395	

3m-major

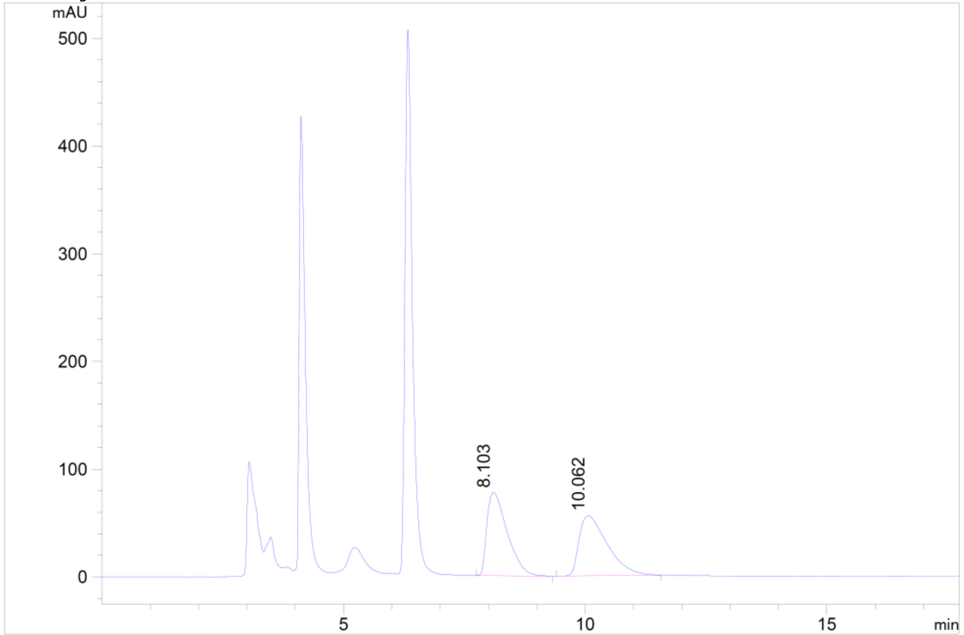

Signal 1: MWD1 C, Sig=210,8 Ref=360,100

Peak #	RT [min]	Type	Width [min]	Area	Area %	Name
1	8.103	BB	0.452	2256.465	49.856	
2	10.062	BB	0.617	2269.493	50.144	

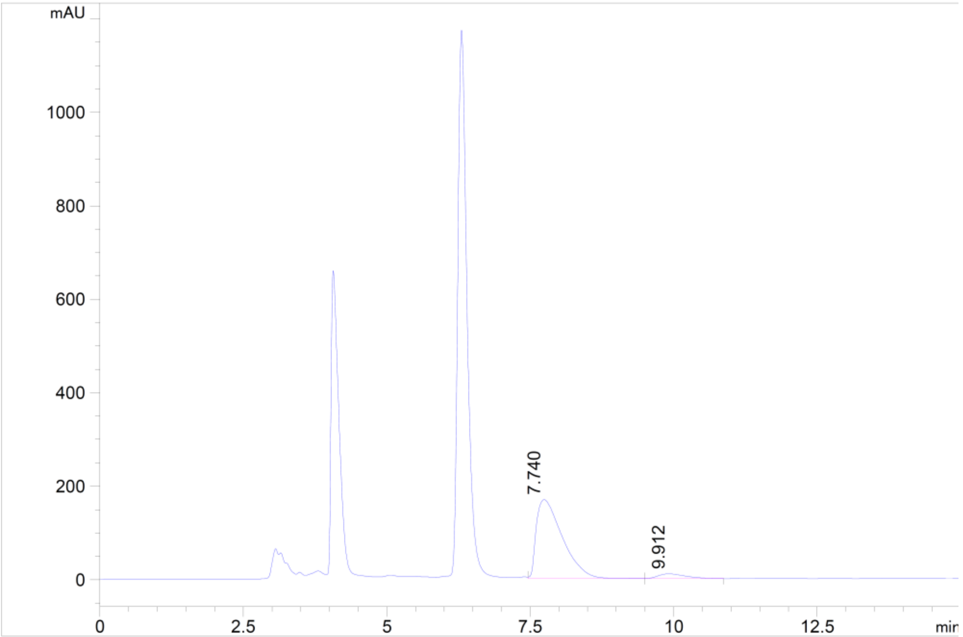

Signal 1: MWD1 C, Sig=210,8 Ref=360,100

Peak #	RT [min]	Type	Width [min]	Area	Area %	Name
1	7.740	FM	0.532	5414.088	93.688	
2	9.912	MF	0.580	364.763	6.312	

Compound **7**

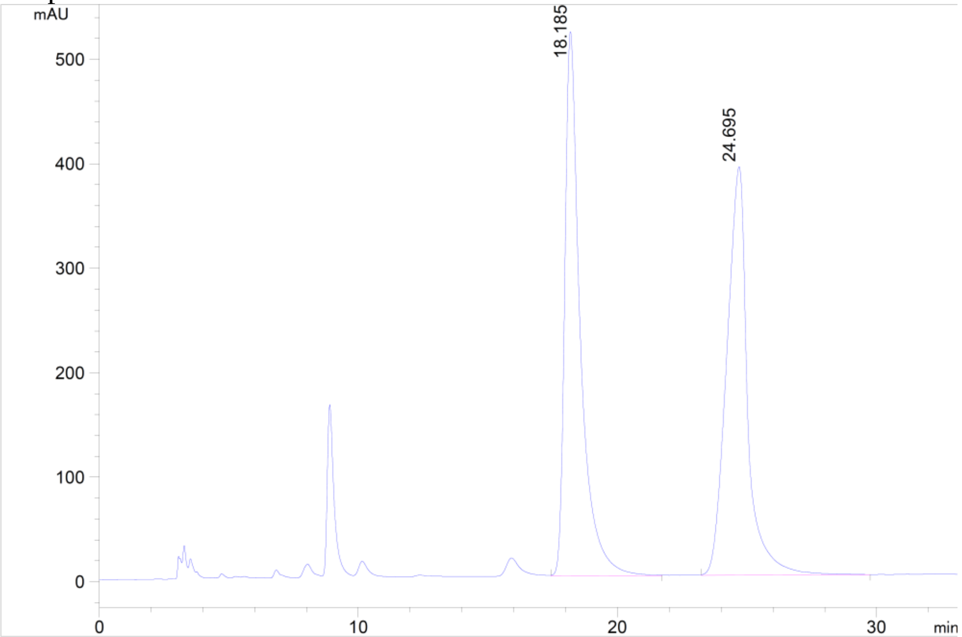

Signal 1: VWD1 A, Wavelength=210 nm

Peak #	RT [min]	Type	Width [min]	Area	Area %	Name
1	18.185	MF	0.699	21856.307	49.833	
2	24.695	FM	0.938	22002.408	50.167	

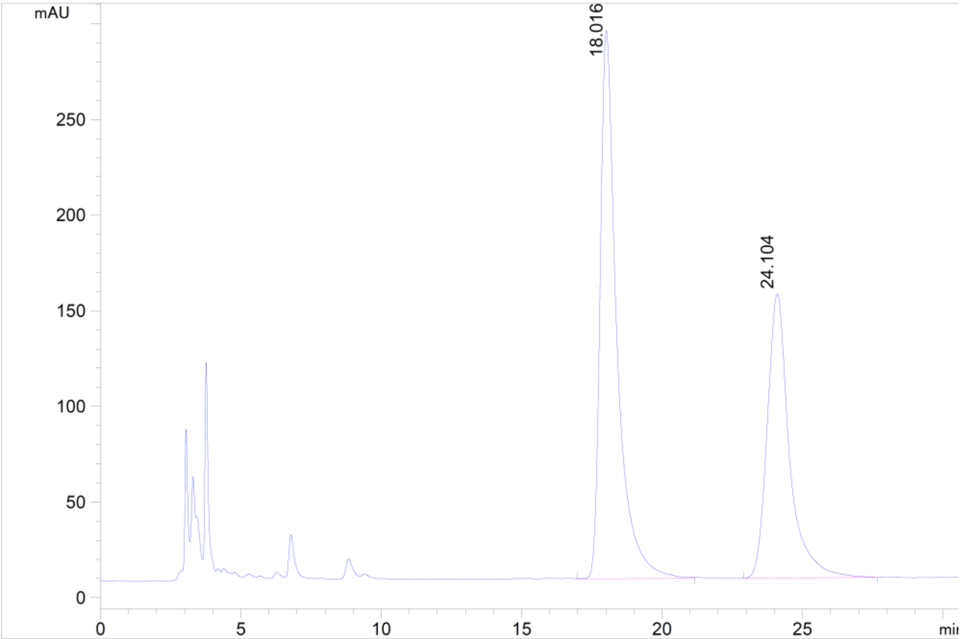

Signal 1: VWD1 A, Wavelength=210 nm

Peak #	RT [min]	Type	Width [min]	Area	Area %	Name
1	18.016	MF	0.690	11867.984	59.306	
2	24.104	BB	0.814	8143.290	40.694	

14 References

1 C. Cassani, R. Martín-Rapún, E. Arceo, F. Bravo and P. Melchiorre, *Nat. Protoc.*, 2013, **8**, 325–344.

2 A. Pedretti, L. Villa and G. Vistoli, *J. Mol. Graph. Model.*, 2002, **21**, 47–49.

3 Drug Design Laboratory - VEGA ZZ, https://www.ddl.unimi.it/cms/index.php?Software_projects:VEGA_ZZ, (accessed 9 February 2021).

4 T. Yanai, D. P. Tew and N. C. Handy, *Chem. Phys. Lett.*, 2004, **393**, 51–57.

5 F. Weigend and R. Ahlrichs, *Phys. Chem. Chem. Phys.*, 2005, **7**, 3297–3305.

6 M. J. Frisch, G. W. Trucks, H. B. Schlegel, G. E. Scuseria, M. A. Robb, J. R. Cheeseman, G. Scalmani, V. Barone, B. Mennucci, G. A. Petersson, H. Nakatsuji, M. Caricato, X. Li, H. P. Hratchian, A. F. Izmaylov, J. Bloino, G. Zheng, J. L. Sonnenberg, M. Hada, M. Ehara, K. Toyota, R. Fukuda, J. Hasegawa, M. Ishida, T. Nakajima, Y. Honda, O. Kitao, H. Nakai, T. Vreven, J. A. J. Montgomery, J. E. Peralta, F. Ogliaro, M. Bearpark, J. J. Heyd, E. Brothers, K. N. Kudin, V. N., K. R. Staroverov, J. Normand, K. Raghavachari, A. Rendell, J. C. Burant, S. S. Iyengar, J. Tomasi, M. Cossi, N. Rega, J. M. Millam, M. Klene, J. E. Knox, J. B. Cross, V. Bakken, C. Adamo, J. Jaramillo, R. Gomperts, R. E. Stratmann, O. Yazyev, A. J. Austin, R. Cammi, C. Pomelli, J. W. Ochterski, R. L. Martin, K. Morokuma, V. G. Zakrzewski, G. A. Voth, P. Salvador, J. J. Dannenberg, S. Dapprich, A. D. Daniels, O. Farkas, J. B. Foresman, J. V. Ortiz, J. Cioslowski and D. J. Fox, Gaussian 09, Revision D.01, Gaussian, Inc., Wallingford CT, 2009.

7 J. Tomasi, B. Mennucci and R. Cammi, *Chem. Rev.*, 2005, 105, 2999–3093.

8 T. Lu and F. Chen, *J. Comput. Chem.*, 2012, **33**, 580–592.

9 U. Varetto, Molekel 5.4.

10 J. Contreras-García, E. R. Johnson, S. Keinan, R. Chaudret, J. P. Piquemal, D. N. Beratan and W. Yang, *J. Chem. Theory Comput.*, 2011, **7**, 625–632.

11 W. Minor, M. Cymborowski, Z. Otwinowski and M. Chruszcz, *Acta Crystallogr. Sect. D Biol. Crystallogr.*, 2006, **D62**, 859–866.

12 CrysAlisPro, 2014, Agilent Technologies. Version 1.171.38.41.

13 R. C. Clark and J. S. Reid, *Acta Crystallogr. Sect. A*, 1995, **A51**, 887–897.

14 G. M. Sheldrick, *Acta Crystallogr. Sect. A Found. Crystallogr.*, 2015, **A71**, 3–8.

15 G. M. Sheldrick, *Acta Crystallogr. Sect. C Struct. Chem.*, 2015, **C71**, 3–8.

16 O. V. Dolomanov, L. J. Bourhis, R. J. Gildea, J. A. K. Howard and H. Puschmann, *J. Appl. Crystallogr.*, 2009, **42**, 339–341.

17 C. F. Macrae, I. J. Bruno, J. A. Chisholm, P. R. Edgington, P. McCabe, E. Pidcock, L. Rodriguez-Monge, R. Taylor, J. Van De Streek and P. A. Wood, *J. Appl. Crystallogr.*, 2008, 41, 466–470.

18 D. B. Hibbert and P. Thordarson, *Chem. Commun.*, 2016, **52**, 12792–12805.

19 Supramolecular.org - Binding Constant Calculators | Supramolecular, http://supramolecular.org/, (accessed 26 January 2021).

20 R. D. Richardson, J. M. Zayed, S. Altermann, D. Smith and T. Wirth, *Angew. Chem., Int. Ed.*, 2007, **46**, 6529–6532.

21 S. Kuwano, T. Suzuki, Y. Hosaka and T. Arai, *Chem. Commun.*, 2018, **54**, 3847–3850.

22 M. Katayama, R. Nagase, K. Mitarai, T. Misaki and Y. Tanabe, *Synlett*, 2006, **2006**, 129–132.

23 D. Tasgin and C. Unaleroglu, *Synthesis*, 2013, **45**, 193–198.
